# Thermophysical Properties and Phase Behavior of CO_2_ with Impurities: Insight from Molecular Simulations

**DOI:** 10.1021/acs.jced.4c00268

**Published:** 2024-07-12

**Authors:** D. Raju, M. Ramdin, T. J. H. Vlugt

**Affiliations:** Engineering Thermodynamics, Process & Energy Department, Faculty of Mechanical Engineering, Delft University of Technology, Leeghwaterstraat 39, Delft 2628CB, The Netherlands

## Abstract

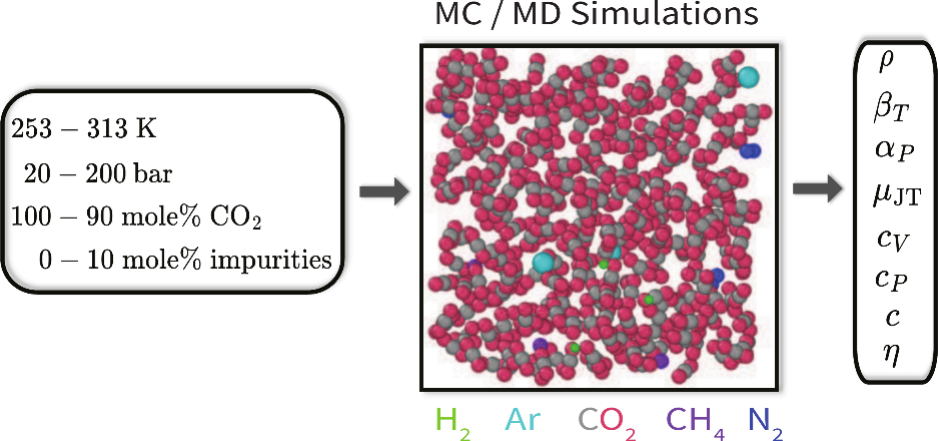

Experimentally determining thermophysical properties for various compositions commonly found in CO_2_ transportation systems is extremely challenging. To overcome this challenge, we performed Monte Carlo (MC) and Molecular Dynamics (MD) simulations of CO_2_ rich mixtures to compute thermophysical properties such as densities, thermal expansion coefficients, isothermal compressibilities, heat capacities, Joule–Thomson coefficients, speed of sound, and viscosities at temperatures of (235–313) K and pressures of (20–200) bar. We computed thermophysical properties of pure CO_2_ and CO_2_ rich mixtures with N_2_, Ar, H_2_, and CH_4_ as impurities of (1–10) mol % and showed good agreement with available Equations of State (EoS). We showed that impurities decrease the values of thermal expansion coefficients, isothermal compressibilities, heat capacities, and Joule–Thomson coefficients in the gas phase, while these values increase in the liquid and supercritical phases. In contrast, impurities increase the value of speed of sound in the gas phase and decrease it in the liquid and supercritical phases. We present an extensive data set of thermophysical properties for CO_2_ rich mixtures with various impurities, which will help to design the safe and efficient operation of CO_2_ transportation systems.

## 1 Introduction

Climate change is being driven mostly by CO_2_ emissions from the combustion of fossil fuels (oil, natural gas, and coal) for electricity production, transportation, heating, and other industrial applications.^1−3^ The 2023 statistical review of world energy indicates that more than 80% of world energy consumption comes from fossil fuels.^2,4^ It is unfeasible to shift entirely to renewable energy resources on a very short time scale.^5^ Carbon Capture and Storage (CCS) is the most popular and large-scale process used in industries to meet the anthropogenic CO_2_ emission targets.^6^ CCS is carried out in three different stages, namely, capture, transportation, and sequestration.^7^ Carbon capture and sequestration depends upon safe and economical transportation of CO_2_.^8^ In the past four decades, pipelines have been used to successfully inject pure CO_2_ into depleted or nearly oil/gas fields for Enhanced Oil/gas Recovery (EOR).^3^ The injection of CO_2_ captured from the flue gas stack is significantly different from pure CO_2_ injection for EOR.^9^ The difference is the presence of impurities, since no gas separation process is 100% efficient.^10^ Impure CO_2_ also differs in composition depending on the source and technology of capture.^11^ It is possible to obtain pure CO_2_ from impure CO_2_, but this will result in additional costs and carbon footprint.^11,12^ The most efficient and preferred phase of transporting CO_2_ in the pipeline is the dense supercritical or liquid phase.^8^ The presence of impurities, especially noncondensable gases (for example, Ar, N_2_, H_2_, O_2_, and CH_4_ which have a low boiling point compared to CO_2_), reduces the density of impure CO_2_ mixtures and is likely to introduce two-phase flow behavior.^13^ Two-phase flows during transportation lead to numerous challenges, mainly pressure surge,^14^ which will sequentially lead to possible pipeline failure.^15^ The recommended level of major impurities in CO_2_ rich stream for safe and efficient pipeline transportation and sequestration from different standards (NETL,^16^ Dynamis,^17^ and ISO^18^) and projects (Porthos^19^ and Teeside^13^) are summarized in Tables 1 and 2. For quality standards of additional minor impurities in CO_2_ transportation systems, the reader is referred to the review article of Simonsen et al.^18^ CO_2_ rich stream with impurities significantly alters the thermodynamic and transport properties of CO_2_, which will, in turn, impact the overall flow behavior, pipeline capacity, and operating window in CO_2_ pipeline systems.^20,21^ Therefore, knowledge on thermodynamic and transport properties is indispensable to model the flow and phase behavior of impure CO_2_ rich mixture within the operating window for the safe design and efficient operation of CO_2_ transportation systems.^9^

**Table 1:** Description of All Components Used in This Work^a^

Chemical name	Chemical formula	CAS number	Force field
Carbon dioxide	CO_2_	124-38-9	TraPPE^60^
Nitrogen	N_2_	7727-37-9	TraPPE^60^
Argon	Ar	7440-37-1	García-Pérez^74^
Hydrogen	H_2_	1333-74-0	Köster^65^
Methane	CH_4_	74-82-8	TraPPE^60^

a Long-range tail corrections for Lennard-Jones (LJ) interactions are used for all components.

**Table 2:** CO_2_ Quality Standards from the National Energy Technology Laboratory (NETL),^16^ Dynamis,^17^ International Standard Organization (ISO),^18^ Porthos,^19^ and Teesside^13^ a

Reference		Concentration (in mol %)				
Component	CAS number	NETL^16^	Dynamis^17^	ISO 27913:2016^18^	Porthos^19^	Teesside^13^
CO_2_	124-38-9	≥95%	>95.5%	≥95%	≥95%	≥95%
Ar	7440-37-1	4%*	<4%		≤0.4%	1%
N_2_	7727-37-9	4%*	<4%		≤2.4%	1%
H_2_	1333-74-0	4%*	<4%		≤0.75%	1%
CH_4_	74-82-8	4%*	<4%		≤1%	1%
O_2_	7782-44-7	4%*	<4%		40 ppm	10 ppm
CO	630-08-0	35 ppm	200 ppm	<2%	≤750 ppm	0.2%
Total (Ar, N_2_, H_2_, CH_4_, O_2_, CO)		≤4%		≤4%	≤4%	

a The impurity percentages with an asterisk (*) in the NETL^16^ advised limits imply that the total impurity concentration should be ≤4%.

CO_2_ captured from the stationary sources is compressed to a pressure higher than the critical pressure to avoid two-phase flows.^8^ Due to planned maintenance or failure, transient processes such as startup, shutdown, and depressurization are anticipated in CO_2_ transportation systems, which plausibly lead to two-phase flows.^8^ Hence, the operational window and conditions of the CO_2_ pipeline systems span a broad range of temperatures and pressures, encompassing transient processes from the wellhead to the bottom of the well. Operating conditions vary based on the geological location and reservoir characteristics.^9^ High temperatures are limited considering the temperature limit of the pipeline coating material (<50 °C) and cooling after compression stages.^3^ The discharge pressure from the compressor to the pipeline is generally in the range of 100 to 200 bar.^3^ The lowest temperature and pressure limit depends on geological conditions and phase behavior of CO_2_ rich stream to maintain a dense phase.^11^ Therefore, the temperature and pressure ranges expected in CO_2_ pipeline systems are assumed as −20 to 40 °C and 0 to 200 bar, respectively. The priori operational conditions considered in this study incorporate conditions at which transient events such as startup, shutdown, and depressurization are anticipated to occur.^22^ Consequently, to ensure accurate modeling of transient processes, it is crucial to know the thermodynamic and transport properties for a wide range of temperatures and pressures expected within the CO_2_ transportation systems.

The thermodynamic and transport properties of impure CO_2_ can be computed from thermodynamic models such as Equations of State (EoS) or other empirical correlations available in the literature.^23−25^ The validity of EoS predictions majorly depends on the interaction parameters that are obtained by fitting Vapor–Liquid Equilibrium (VLE) data obtained from experiments and assumptions used to develop EoS.^26,27^ Most EoS models accurately predict the thermodynamic properties related to first-order derivatives of the thermodynamic potentials (Gibbs energy, Helmholtz energy, enthalpy, and internal energy), i.e., the phase equilibria.^28,29^ The second-order derivative properties, such as isothermal compressibility, thermal expansion coefficient, Joule–Thomson coefficient, heat capacity, and speed of sound, are not predicted accurately by the majority of EoS models.^28,29^ These properties serve as a basis for the design and modeling of pipeline transportation systems. Especially, knowledge on speed of sound is crucial in characterizing the state and structure of the fluid in pipeline transportation systems.^30,31^ Many literature studies predict the thermodynamic and transport properties of impure CO_2_ using either a simple or advanced EoS,^32,33^ but no general agreement has been made to use a particular EoS with specific interaction parameters for CO_2_ mixtures with small amount of impurities.^21,34^

Determination of the thermodynamic and transport properties from experiments is difficult due to low concentration limits of impurities in CO_2_ transportation systems (Table 2). Performing experiments for a wide range of compositions and conditions of multicomponent CO_2_ mixtures is costly and time-consuming.^35^ Molecular simulations with classical force fields are widely used to compute the thermodynamic and transport properties of multicomponent gas systems.^36−38^ Densities, viscosities, and phase equilibria calculated from molecular simulations provide reasonable and sometimes better predictions than EoS since calculations are based on accurate interaction potentials between atoms and molecules.^37,39,40^ Simulations can efficiently compute heat capacities and speed of sound, both of which are essential for modeling transient phenomena like vapor collapse accurately.^21,30,36,41^ Using classical force-field-based Monte Carlo (MC) simulations, Cresswell et al.^21^ computed phase equilibria and densities of binary mixtures of CO_2_ with Ar, N_2_, H_2_, and O_2_ for a range of temperatures from 0 to 50 °C and pressures up to 200 bar. Aimoli et al.^42^ evaluated the performance of different force fields for computing density, isothermal compressibility, thermal expansion coefficient, heat capacity at constant volume and pressure, Joule–Thomson coefficient, viscosity, and speed of sound for pure CO_2_ and CH_4_ for a range of temperatures from −20 to 100 °C and pressures up to 1000 bar using Molecular Dynamics (MD) simulation. To the best of our knowledge, most molecular simulation studies are limited to binary CO_2_ mixtures.^21,42^ Molecular simulations dedicated to multicomponent CO_2_ mixtures are extremely limited^43,44^ or nonexistent.

In this work, we compute the thermodynamic and transport properties of pure and impure CO_2_ streams for a range of impurity levels ranging from (1–10) mol % (which includes 12 CO_2_ binary mixtures with 1 mol %, 5 mol %, and 10 mol % impurities which are shown in Section S14 of the Supporting Information and 36 multicomponent CO_2_ mixtures with impurities ≤4 mol % which are shown in Sections S17 and S18 of the Supporting Information) using MC and MD simulation techniques. Simulations were carried out for a range of temperatures from −20 to 40 °C and pressures up to 200 bar. The main impurities, N_2_, Ar, H_2_, and CH_4_, are selected to investigate the effect of impurities on the thermodynamic and transport properties. A comprehensive list of chemical components, CAS numbers, and force fields of all components used in this work is shown in Table 1. Properties calculated within the operating window include density, isothermal compressibility, thermal expansion coefficient, heat capacity at constant volume and pressure, Joule–Thomson coefficient, shear viscosity, and speed of sound. We showed that, in comparison to pure CO_2_ density, a CO_2_ rich mixture with molecular weight lower than pure CO_2_ at a condition had lower densities. We also showed that impurities decrease the value of thermal expansion coefficients, isothermal compressibilities, heat capacities, and Joule–Thomson coefficients in the gas phase and increase the value of these properties in the liquid and supercritical phases. Conversely, impurities tend to increase the speed of sound in the gas phase and decrease the speed of sound in the liquid and supercritical phases. Our results show that the order of influence of a particular impurity on a thermodynamic property other than density correlates with the critical temperature of that impurity.

This article is organized as follows: The methodology used to compute thermodynamics and transport properties is explained in Section 2, followed by the simulation details in Section 3. In Section 4, we present the validation and results of the computed thermodynamic and transport properties. In Section 5, our key findings are summarized.

## 2 Theoretical Background

To compute the speed of sound (*c*), one requires other properties, which include heat capacity at constant pressure (*C*_*P*_), heat capacity at constant volume (*C*_*V*_), and isothermal compressibility (β_*T*_)^30,31^
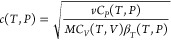
1where *v* is the molar volume of the pure component or the mixture and *M* is the molar mass of the pure component or the mixture. For a mixture, *M* can be calculated from the pure component molar mass
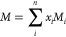
2where *n* is the number of components present in the mixture and *x*_*i*_ and *M*_*i*_ are the mole fraction and molar mass of each component present in the mixture. To calculate *C*_*V*_, *C*_*P*_, and β_*T*_, the derivatives of internal energy, volume, and enthalpy with respect to temperature and pressure have to be determined^36,45^
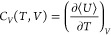
3
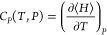
4
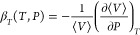
5where *U* and *H* are the internal energy and enthalpy of the system, respectively, and ⟨···⟩ denotes the ensemble average of an ensemble. The internal energy (*U* = *U*^internal^ + *U*^external^) and enthalpy (*H* = *U*^internal^ + *U*^external^ + *K* + *PV*) in eq 3 and eq 4 include the kinetic energy term (*K*) in addition to the potential energy contribution from intramolecular molecular interaction (interaction inside molecules which is denoted as *U*^internal^) and intermolecular interactions (interaction between molecules which is denoted as *U*^external^). Hence, *C*_*V*_ and *C*_*P*_ have been split into ideal and residual contributions. Following the work of Lagache et al.^45^ we can write
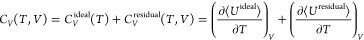
6
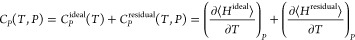
7Heat capacities *C*_*V*_^ideal^ =  and *C*_*P*_^ideal^ =  can be obtained from the standard thermodynamic databases^46,47^ or from quantum mechanical calculations. In this study, the latter approach was used to calculate *C*_*V*_^ideal^(*T*) and *C*_*P*_^ideal^(*T*) using the Gaussian 09 software^48^ with the B3LYP theory and a 6-31G(d,p) basis set. The derivatives  and  required to calculate *C*_*V*_ and *C*_*P*_ are computed from fluctuations in *NVT* and *NPT* ensembles, respectively^30,31,36,45^
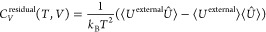
8
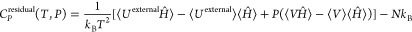
9where *k*_B_ is the Boltzmann constant, *N* is the number of molecules in the system, *Û* (*Û* = *U*^internal^ + *U*^external^) is the configurational energy, and *Ĥ* (*Ĥ* = *U*^internal^ + *U*^internal^ + *PV*) is the configurational enthalpy of the system. The molar heat capacities (*c*_*P*_ and *c*_*V*_) can be obtained from
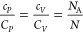
10where *N*_A_ is Avogadro’s number and *N* is the number of molecules in the system. The derivative  is required to calculate β_*T*_ defined in eq 5, and is computed using the following fluctuation formula,^49^
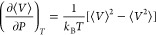
11Analogous to the computation of the speed of sound, the calculation of the Joule–Thompson coefficient requires heat capacity at constant pressure (*c*_*P*_) and thermal expansion coefficient (α_*P*_).
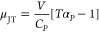
12The value of α_*P*_ is computed from the derivative of volume with respect to temperature
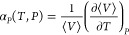
13The derivative  which is required to calculate α_*T*_ is computed using^36,42,45^
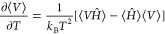
14It is important to note that *C*_*V*_ and α_*P*_ can be computed indirectly using the thermodynamic relations^50^
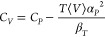
15
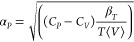
16The proofs of the mathematical derivation of equations to compute *C*_*P*_, *C*_*V*_, β_*T*_, *c*, α_*P*_, and μ_JT_ from simulations are provided in Sections S3–S8 of the Supporting Information.

## 3 Simulation Details

All force-field-based MC simulations were performed using the open-source software Brick, which uses the Continuous Fractional Component Monte Carlo (CFCMC) method^51−55^ to calculate thermodynamics properties. Force-field-based MD simulations were performed in the Large-scale Atomic/Molecular Massively Parallel Simulator (LAMMPS: version August 2023) package^56^ to compute viscosities. All molecules were described with site-based conventional intermolecular potentials with point charges centered at the atom or a dummy site. The pairwise-additive 12-6 Lennard-Jones (LJ) interaction potentials are used to model interactions:
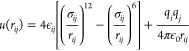
17The Lorentz–Berthelot mixing rules were used to compute LJ interactions for dissimilar atoms:^49,57^
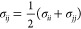
18
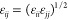
19Different force fields for CO_2_ are available in the literature. These range from simple single-site force fields like the Higashi model^58^ and Statistical Associating Fluid Theory (SAFT)-γ^59^ to more complex three-site force fields such as the Transferable Potentials for Phase Equilibria (TraPPE)-rigid,^60^ TraPPE-flex,^61^ Elementary Physical Model 2 (EPM2),^62^ Zhang model,^63^ and Cygan model.^64^ Aimoli et al.^42^ investigated the performance of seven CO_2_ force fields (TraPPE-rigid, TraPPE-flex, EPM2, Zhang model, Cygan model, Higashi model, and SAFT-γ) on density and second derivative thermodynamic properties of CO_2_ up to 900 K and 1000 bar. Alimoli et al.^42^ found that TrappE-rigid, EPM2, the Zhang model, and SAFT-γ produced nearly identical densities of CO_2_, but the SAFT-γ force field predicted second derivative properties less accurately than TrappE-rigid, EPM2, and Zhang model compared to reference data from National Institute of Standards and Technology (NIST). Aimoli et al.^42^ also investigated the TraPPE and SAFT-γ single-site force fields for CH_4_ and found that the TraPPE force field performed the best compared to NIST reference data. Similar to CO_2_, several force fields for N_2_ can be found in the literature, such as TraPPE,^60^ Köster et al. model,^65^ Murthy et al. model,^66^ and Galassi and Tildesley model.^67^ Force fields from Galassi and Tildesley^67^ and Murthy et al.^66^ were not optimized for VLE calculations.^60^ The force fields TraPPE^60^ and Köster et al.^65^ are three-site models with quadrupole moment, but they differ in their parametrization. Rahbari et al.^36^ compared the performance of five different force fields for H_2_ (Cracknell,^68^ Buch,^69^ Hirschfelder et al.,^70^ Marx and Nielaba,^71^ and Köster et al.^65^). These authors found that the force field developed by Köster et al.^65^ best predicted the second derivative thermodynamic properties with the least deviation compared to data obtained from REFPROP.^72^ The TraPPE^60,73^ force field was used in this study for CO_2_, N_2_, and CH_4_ molecules since the primary objective in developing the TraPPE force field is to predict the thermophysical properties for a wide range of state conditions and compositions. Hydrogen is simulated using the Köster et al.^65^ model. The force field from García-Pérez et al.^74^ commensurate with tail corrections was used for the monatomic nonpolar argon molecule. Molecular models of H_2_, Ar, and CH_4_ are single-site models consisting of a single Lennard-Jones (LJ) interaction site with no point charges, whereas those of CO_2_ and N_2_ are three-site models (including the dummy charge site for N_2_) with point charges. The force field parameters of CO_2_, Ar, N_2_, H_2_, and CH_4_ are listed in Section S1 of the Supporting Information. All molecules were treated as rigid objects. LJ interactions in a simulation box have a cutoff radius of 12 Å with analytic tail corrections.^57^ Periodic boundary conditions were imposed in all directions. The Ewald summation is used to compute the electrostatic energy due to point charges. To minimize computation expense, the cutoff radius of real space electrostatic interactions is chosen to limit the *k*-vectors (to a maximum of *k* = 8) in Fourier space, with an accuracy of 10^–6^. For instance, we chose a cutoff radius in real space as 12 Å with a damping parameter of α = 0.2650 Å^–1^ for a box of size 30 Å, whereas for a box of size 40 Å we chose a cutoff radius in real space as 16 Å with a damping parameter of α = 0.1960 Å^–1^. For each condition (concentration, temperature, and pressure), 10 independent simulations are performed, and each simulation started with a different initial configuration. These 10 simulations are divided into 5 blocks from which average values and uncertainties of thermodynamic and transport properties are calculated. The mean and standard deviation of 5 blocks are the average value and uncertainty of a thermodynamic or transport property. The aforementioned force fields and simulation details used for the MC and MD simulations are exactly the same.

MC simulation of Gibbs Ensemble (GE) in *NVT* and *NPT* version is the most convenient way to perform phase equilibria calculations.^75−77^ In GE, two simulation boxes are considered: one represents the liquid phase, and the other represents the gas phase. The simulation boxes are allowed to exchange energy, volume, and molecules. In a dense liquid simulation box, the insertion of a molecule in a single step is impeded due to the low probability of finding a cavity to accommodate a molecule, and the deletion of a molecule in a single step leaves the simulation box with a high energy penalty to form a new configuration.^53^ The CFCMC method^51−55^ overcomes this drawback by gradual insertion and removal of so-called fractional molecules by which the surrounding whole molecules can adapt simultaneously by performing trial moves related to fractional molecules besides thermalization trial moves such as translations, rotations, and, volume changes. In CFCGE, two simulation boxes with indistinguishable whole molecules and fractional molecules (fractional molecules can be in either of the simulation boxes, but one per component type) are used for simulating the phase coexistence. The interaction of the distinguishable fractional molecule of a component type *i* with a whole molecule is scaled with a coupling parameter λ_*i*_ ∈ [0, 1].^78^ The trial moves related to fractional molecules are randomly changing the value of λ while keeping the orientation and position of all the molecules constant, insertion of a fractional molecule in another simulation box at a randomly selected orientation and position while keeping the orientation and position of the whole molecules constant, and changing the identity of a fractional molecule in a simulation box to a whole molecule while simultaneously transforming the randomly selected whole molecule to a fraction molecule in another simulation box.^79^ Further details specific to phase equilibria calculation in the CFCGE can be found elsewhere.^53,78^ The VLE of pure component (unary) systems, CO_2_, Ar, N_2_, H_2_, and CH_4_, were computed in the *NVT* version of the CFCGE. The phase equilibria (*Pxy* diagram) of binary mixtures which include CO_2_/Ar, CO_2_/N_2_, CO_2_/H_2_, and CO_2_/CH_4_ are computed in the *NPT* version of the CFCGE. The simulation box sizes and initial distribution of whole molecules between the simulation boxes were specified based on the experimental data of the state point for both the unary and binary systems. For example, for phase equilibria computation of the CO_2_ and Ar binary system at 105 bar, we chose 360 CO_2_ molecules and 140 Ar molecules for the intended liquid box of size 30 Å and 180 CO_2_ molecules and 320 Ar molecules for the intended gas box of size 40 Å. For a pressure of 35 bar, we chose 470 CO_2_ molecules and 30 Ar molecules for the intended liquid box of size 30 Å and 325 CO_2_ molecules and 175 Ar molecules for the intended gas box of size 40 Å. For simulating the phase coexistence of unary and binary systems, an equilibration run of 5 × 10^4^ and 1 × 10^5^ MC cycles was performed, respectively. The number of trial moves in an MC cycle in Brick-CFCMC equals the total number of molecules in the simulation box, with a minimum of 20. Following the equilibration run, a production run of 1 × 10^5^ cycles was performed for unary systems and 2 × 10^5^ cycles for binary systems to compute the coexistence densities and mole fractions of the components, respectively.

To calculate thermodynamic properties, density (ρ), isothermal compressibility (β_*T*_), thermal expansion coefficient (α_*P*_), molar heat capacity at constant volume (*c*_*V*_), molar heat capacity at constant pressure (*c*_*P*_), Joule–Thompson coefficient (μ_JT_), and speed of sound (*c*) MC simulations were performed without fractional molecules. The values of *c*_*P*_, β_*T*_, μ_JT_, and α_*P*_ were computed using the fluctuation equations in the *NPT* ensemble defined in eqs 4, 5, 12, and 13, respectively. To compute *c*_*V*_ and consequently the speed of sound (*c*), it is important to extract the ensemble-averaged volume (⟨*V*⟩) from the *NPT* ensemble that reflects the same state for performing a simulation in the *NVT* ensemble with the same number of molecules. The simulations were performed with 300 molecules irrespective of the state and system. An equilibration of 5 × 10^4^ MC cycles is performed to equilibrate the system successively, and 10^6^ production runs are performed for each simulation.

In MD simulations, initial configurations of molecules in the cubic simulation boxes are constructed using PACKMOL^80^ and fftool.^81^ Periodic boundary conditions are applied to simulation boxes in all directions. A cutoff radius of 12 Å is used for LJ interactions with analytic tail corrections. All molecules are treated as rigid bodies, and Newton’s equations of motion are integrated using the velocity-Verlet algorithm with a time step of 0.5 fs. To thermostat and barostat the system, the Nosé–Hoover type is used with coupling constants of 0.1 and 1 ps, respectively. The Particle–Particle Particle–Mesh (PPPM) method is used to handle long-range electrostatic interactions with a cutoff radius of 12 Å and 10^–6^ accuracy. Shear viscosities (η) are calculated by performing an Equilibrium Molecular Dynamics (EMD) simulation by using the On-the-fly Computation of Transport Properties (OCTP) plugin in LAMMPS. The OCTP combines the Einstein relations with an order-*n* algorithm to calculate viscosity. Additional details about the OCTP’s computation of transport properties can be found elsewhere.^82^ System sizes chosen to evaluate η of pure and multicomponent mixtures were 100 molecules for the very diluted gas phase, 300 molecules for the gas phase (40 to 80 bar), and 400 molecules for the liquid and supercritical phases (≥100 bar). The various steps involved in computing η for one independent simulation are as follows: First, a simulation is carried out in the *NPT* ensemble (0.5 ns equilibration run and 1 ns production run) to compute the ensemble average volume (⟨*V*⟩). Next, the simulation box is scaled according to the computed value of ⟨*V*⟩, and this system is then used to perform a simulation in the *NVT* ensemble (0.5 ns equilibration run and 1 ns production run) to compute the average total energy of the system (⟨*E*⟩). Finally, the ensemble average total energy is used to scale to the kinetic energy of the system to perform simulations in the *NVE* ensemble. Viscosities are calculated in the *NVE* ensemble, ensuring that the thermostat and barostat have no effect on the results. In the *NVE* ensemble, a production run of 5 ns is simulated to compute η.

## 4 Results and Discussion

This section shows and discusses the thermodynamic and transport properties of single-component and multicomponent CO_2_ systems. All properties of interest computed in this work from molecular simulations are compared with data sets generated from the NIST REFPROP database version-10.0^72^ except when stated otherwise. EoS and correlation models used by REFPROP for computing pure component (CO_2_, N_2_, Ar, H_2_, and CH_4_) thermodynamic and transport properties are listed in Table S11 of the Supporting Information. For multicomponent systems, the Groupe Europèen de Recherches Gazières (GERG-2008) EoS^24^ is chosen in REFPROP. The GERG-2008 EoS is less accurate for unary systems.^72^ Therefore, it is used only to compute the thermodynamic properties of multicomponent systems. The GERG-2008 EoS was originally developed for natural gas mixtures containing 21 components, including CO_2_, N_2_, Ar, H_2_, and CH_4_. Multicomponent mixtures containing high CO_2_ concentrations and low levels of impurities were not the main focus for the development of the GERG-2008 EoS.^24,83^ The quality and quantity of experimental data used in developing the GERG-2008 EoS limit its accuracy.^24^ Thermophysical properties computed from molecular simulations will help in optimizing the EoS.^26^ Viscosities computed from MD simulations of multicomponent systems are compared with those obtained from the Extending Corresponding States (ECS) model available in REFPROP.^84^ Further details specific to the ECS model can be found elsewhere.^84^ Since the experimental data for multicomponent systems are scarce and simulating an exact composition as experiments is impossible with a small system of only 300 molecules, we opted to compare results from molecular simulations with those obtained from EoS. The numerical data used to generate all plots is provided in Sections S3, S4, and S14–S17 of the Supporting Information. The deviations of properties computed from simulations with respect to REFPROP data sets are computed using
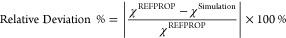
20where χ^Simulation^ and χ^REFPROP^ are the properties of interest computed from simulations and REFPROP, respectively. For the sake of clarity, plots for temperatures of 253 and 313 K are shown for binary systems, and plots for a temperature of 253 K are shown for multicomponent systems despite that properties of interest were estimated for four different temperatures: 253, 273, 293, and 313 K.

### 4.1 Thermodynamic Properties

#### 4.1.1 Phase Equilibria

The VLE of pure components (CO_2_, Ar, N_2_, H_2_, and CH_4_) computed from the CFCMC simulations in the *NVT* version are compared to the REFPROP database. The computed VLE curves compared to the REFPROP database are shown in Section S2 of the Supporting Information. Critical temperatures (*T*_c_) and densities (ρ_c_) of all pure components computed from simulations using the law of rectilinear diameters and EoS models are listed in Table 3. Our results show that the computed liquid and vapor densities are in excellent agreement with respect to the REFPROP data set, except for the liquid densities of hydrogen. Deviations of the computed liquid densities and, in turn, the *T*_c_ and ρ_c_ of hydrogen with respect to REFPROP are due to the domination of quantum effects at low temperatures.^65^ This work focuses on temperatures significantly higher (>250 K) than H_2_ VLE temperatures; hence, only the gas densities were considered for the validation of H_2_ force field.

**Table 3:** Comparison of Critical Temperatures (*T*_c_^SIM^) and Densities (ρ_c_^SIM^) of Pure Components Computed from Simulations Using the Law of Rectilinear Diameters with Critical Temperatures (*T*_c_^REFP^) and Densities (ρ_c_^REFP^) Obtained from the REFPROP^72^ Database

Components	CAS number	*T*_c_^REFP^/[K]	*T*_c_^SIM^/[K]	ρ_c_^REFP^/[kg m^–3^]	ρ_c_^SIM^/[kg m^–3^]
CO_2_	124-38-9	304.1	306.05	467.6	466.53
N_2_	7727-37-9	126.20	126.30	314.40	308.73
Ar	7440-37-1	150.65	148.93	536	539.70
H_2_	1333-74-0	33.18	33.36	31.04	37.39
CH_4_	74-82-8	190.6	191.51	162.1	160.67

**Figure 1:**
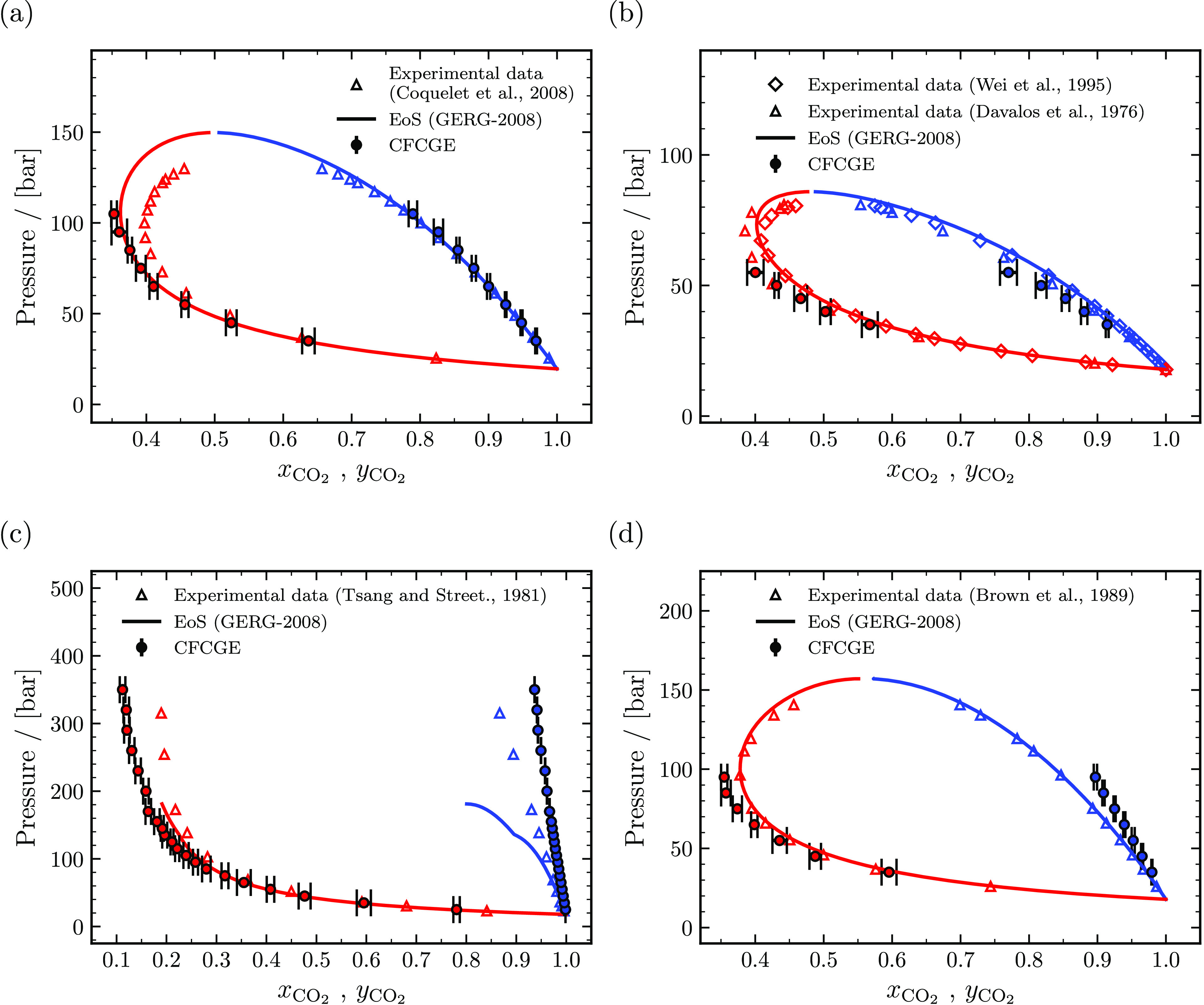
Comparison of binary VLE (*Pxy* diagram), i.e., the bubble points (blue symbols and lines) and dew points (red symbols and lines) of (a) CO_2_/Ar, (b) CO_2_/CH_4_, (c) CO_2_/H_2_, and (d) CO_2_/N_2_ mixtures computed from CFCGE simulations with experimental data^85−89^ and the GERG-2008 EoS.^24^ The simulations are performed at 253.28 K for CO_2_/Ar mixtures and at 250 K for CO_2_/CH_4_, CO_2_/H_2_, and CO_2_/N_2_ mixtures.

Figure 1 shows the phase equilibria (*Pxy* diagram) of four binary mixtures comprised of CO_2_, Ar, N_2_, H_2_, and CH_4_ computed from CFCGE simulations (in the *NPT* version) compared with experimental data and the GERG-2008 EoS. The *Pxy* diagram, i.e., the bubble and dew points of CO_2_/Ar mixtures, is computed at 253.28 K and compared with experimental data of Coquelet et al.,^85^ and for CO_2_/CH_4_ mixtures, simulations were performed at 250 K and compared to experimental data of Wei et al.^86^ and Davalos et al.^87^ Similarly, the *Pxy* diagrams of CO_2_/H_2_ and CO_2_/N_2_ mixtures are computed at 250 K and validated with experimental data of Tsang and Street^88^ and Brown et al.,^89^ respectively. Figure 1a shows CO_2_ liquid and gas mole fractions of CO_2_/Ar mixtures, computed from the CFCGE simulations compared to experimental data of Coquelet et al.^85^ and the GERG-2008 EoS. Our results match well with the GERG-2008 EoS compared to the experimental data of Coquelet et al.^85^ Bubble and dew points of CO_2_/CH_4_ mixtures (shown in Figure 1b) computed from simulations agree well with experimental data of Davalos et al.^87^ when compared to experimental data of Wei et al.^86^ and the GERG-2008 EoS at high pressures. The phase equilibria of the CO_2_/H_2_ binary system are shown in Figure 1c. REFPROP fails to converge for pressures larger than 170 bar when using the GERG-2008 EoS. This discrepancy seen in the GERG-2008 EoS was also reported in the work of Shin et al.^34^ The bubble points obtained from the GERG-2008 EoS using REFPROP provide poor estimates when compared to the experimental data of Tsang and Street.^88^ Mole fractions computed from simulations agree fairly with experimental data of Tsang and Street,^88^ and less than 5% relative deviation was noticed at high pressures. Dew points of CO_2_/N_2_ mixtures shown in Figure 1d have a reasonable agreement with EoS and experimental data of Brown et al.,^89^ but the computed bubble points have a maximum relative deviation of 3% at high pressures. At high pressures, the dew and bubble points computed from CFCGE simulations agree moderately with the GERG-2008 EoS and experiments for all systems shown in Figure 1. This is because the mixing rules used in the simulations did not include binary interaction parameters. Computing bubble and dew points close to the critical pressure is challenging, even with a system size of 1000 molecules (the system size chosen in this study to compute the phase equilibria). In principle, one can perform simulations with large system sizes for pressures in the neighborhood of the critical pressure at the cost of much larger computations. Nevertheless, it is impossible to compute accurate bubble and dew points very close to critical pressure since simulation boxes may switch identities, which complicates ensemble averaging. Similarly, the GERG-2008 EoS model fails to predict reliable results for pressures close to the critical pressure due to the unavailability of experimental data to develop the GERG-2008 EoS.^83^

#### 4.1.2 Densities (ρ)

**Figure 2:**
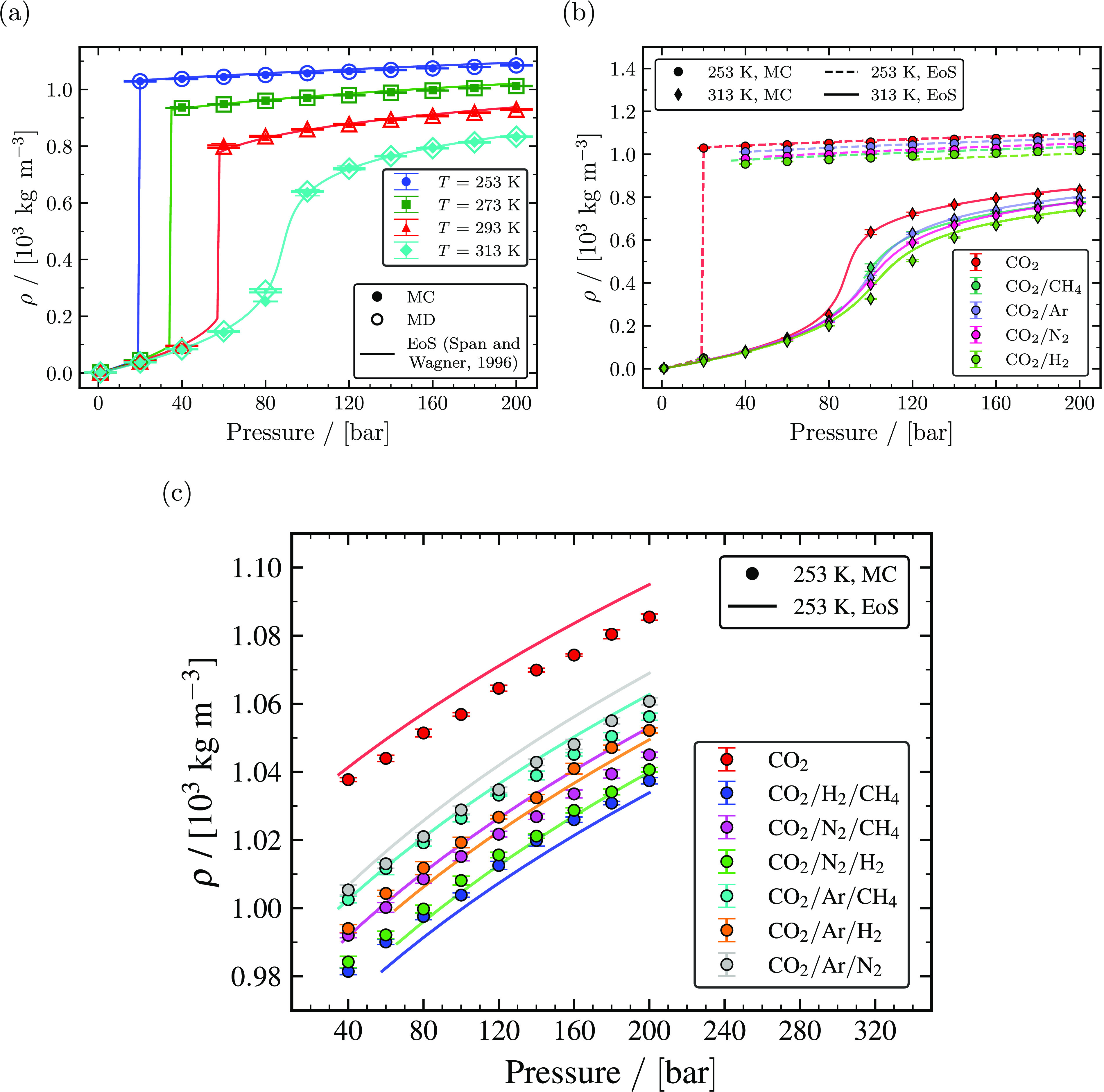
Computed densities as a function of temperature and pressure. (a) The calculated densities of pure CO_2_ from MC simulations (closed symbols), MD simulations (open symbols), and the Span and Wagner EoS^23^ (solid lines) for temperatures of 253, 273, 293, and 313 K. (b) Densities of binary mixtures with 95 mol % CO_2_ and 5 mol % impurities (CH_4_, Ar, N_2_, and H_2_) computed from MC simulations (closed symbols) and the GERG-2008 EoS^24^ (lines) compared with densities of pure CO_2_ computed from MC simulations and the Span and Wagner EoS^23^ for temperatures of 253 and 313 K. (c) Densities of ternary mixtures with 96 mol % CO_2_ and 2 mol % impurities for each of two components (CH_4_, Ar, N_2_, and H_2_) computed from MC simulations (closed symbols) and the GERG-2008 EoS^24^ (lines) compared with densities of pure CO_2_ computed from MC simulations and the Span and Wagner EoS^23^ at 253 K.

Densities of pure CO_2_ computed from MC and MD simulations for temperatures of 253, 273, 293, and 313 K and pressures ranging from 20 to 200 bar are shown in Figure 2a. MC and MD simulations have an excellent agreement with the Span and Wagner EoS for all temperatures. The computed densities from MC simulations have a maximum relative deviation of ca. 0.93% at 313 K and 200 bar, and MD simulations have a maximum relative deviation of ca. 0.89% at 293 K and 200 bar, excluding conditions close to the critical point. As expected, the density decreases with temperature to a large extent after the transition from gas to liquid or supercritical fluid. Figure 2b shows densities of different binary mixtures computed from MC simulations compared with densities obtained from the GERG-2008 EoS for 95 mol % CO_2_ and 5 mol % of one of the impurities (CH_4_, Ar, N_2_, and H_2_) for temperatures of 253 and 313 K. The computed densities of CO_2_/N_2_, CO_2_/CH_4_, CO_2_/Ar, and CO_2_/H_2_ binary mixtures from MC and MD are in close agreement with densities obtained from the GERG-2008 EoS and have a maximum relative deviation of ca. 4.4% (in MC simulation at 313 K and 60 bar), ca. 4.3% (in MD simulations at 253 K and 80 bar), ca. 2.1% (in MC simulations at 253 K and 40 bar), and ca. 6.6% (in MD simulations at 313 K and 60 bar), respectively, excluding state points close to the critical point. A comprehensive analysis of the gas phase densities of binary mixtures with 5 mol % of one of the impurities found that impurities do not significantly change the gas phase densities. The influence of impurities on densities is consistent with the molecular weight of the mixtures. A mixture with H_2_ as an impurity decreases the density to a large extent, followed by Ar, N_2_, and CH_4_. Densities were also computed for 1 and 10 mol % impurities at temperatures of 253, 273, 293, and 313 K from MC simulations and MD simulations. The computed densities are provided in Tables S73–S167 of the Supporting Information. Densities of all binary mixtures are shown in Section S15 of the Supporting Information; densities decrease with increasing mol % of impurity compared to densities of pure CO_2_ irrespective of the temperature. Figure 2c shows the liquid densities of ternary mixtures with 96 mol % CO_2_ and 2 mol % impurities for each of the two components (CH_4_, Ar, N_2_, and H_2_) at 253 K. The liquid densities of ternary mixtures may appear to have some deviation when compared to the GERG-2008 EoS due to the reduced axis range seen in Figure 2c. The maximum relative deviation of ternary mixture liquid densities in Figure 2c was ca. 0.78% for the CO_2_/Ar/N_2_ and CO_2_/N_2_/CH_4_ mixtures at 200 bar. The liquid densities of ternary mixtures were observed to have good agreement with the GERG-2008 EoS. Similar to binary mixtures, ternary mixtures with the least molecular weight tend to have lesser densities when compared to other ternary mixtures. For example, a ternary mixture with 2 mol % H_2_ and 2 mol % CH_4_ as impurities, which have the lowest molecular weight among other ternary mixtures, have the lowest densities when compared to densities of other ternary mixtures.

#### 4.1.3 Thermal Expansion Coefficients (α_*P*_)

**Figure 3:**
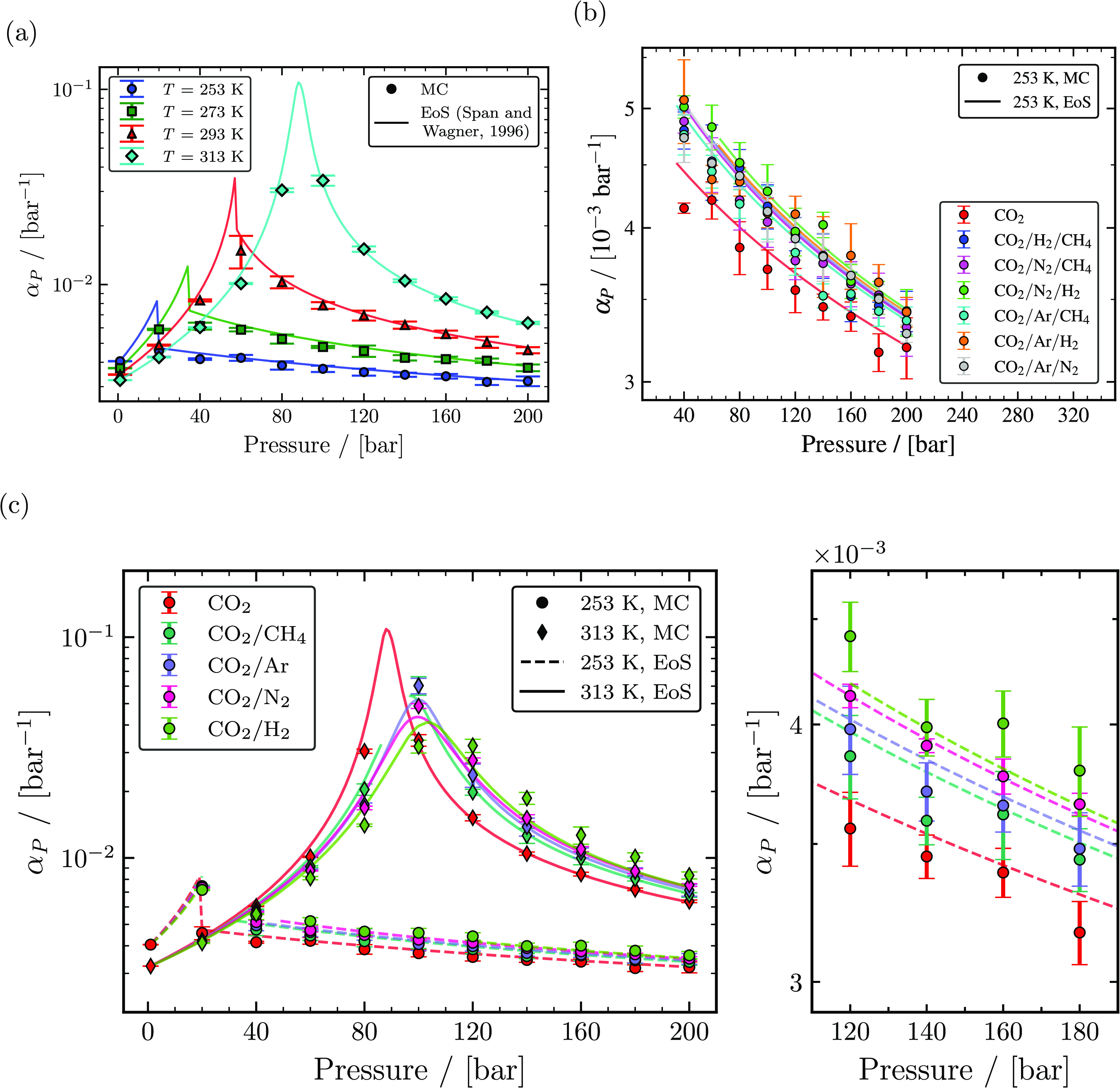
Computed thermal expansion coefficients as a function of temperature and pressure. (a) The calculated thermal expansion coefficients of pure CO_2_ from MC simulations (closed symbols) and the Span and Wagner EoS^23^ (solid lines) for temperatures of 253, 273, 293, and 313 K. (b) Thermal expansion coefficients of ternary mixtures with 96 mol % CO_2_ and 2 mol % impurities for each of two components (CH_4_, Ar, N_2_, and H_2_) computed from MC simulations (closed symbols) and the GERG-2008 EoS^24^ (lines) compared with thermal expansion coefficients of pure CO_2_ computed from MC simulations and the Span and Wagner EoS^23^ for temperatures of 253 and 313 K. (c) Thermal expansion coefficients of binary mixtures with 95 mol % CO_2_ and 5 mol % impurities (CH_4_, Ar, N_2_, and H_2_) computed from MC simulations (closed symbols) and the GERG-2008 EoS^24^ (lines) compared with thermal expansion coefficients of pure CO_2_ computed from MC simulations and the Span and Wagner EoS^23^ at 253 K.

Thermal expansion coefficients (α_*P*_) computed from MC simulations using eq 13 in the *NPT* ensemble for pure CO_2_, binary CO_2_ mixtures, and ternary CO_2_ rich mixtures with different impurities are shown as a function of temperature and pressure in Figure 3. α_*P*_ values of pure CO_2_ computed at temperatures of 253, 273, 293, and 313 K are shown in Figure 3a. α_*P*_ of pure CO_2_ increases with temperature, whereas, with increasing pressure, the value of α_*P*_ increases until it reaches a maximum value close to its saturation pressure and then decreases with increasing pressure. MC simulations closely predicted the peak of α_*P*_. Thermal expansion coefficients of pure CO_2_ computed at conditions close to its saturation pressure were found to have large uncertainties and deviations when compared to the Span and Wagner EoS. Excluding conditions close to the critical point, α_*P*_ of pure CO_2_ computed from MC simulations was in good agreement with the Span and Wagner EoS for all temperatures with a maximum relative deviation of ca. 6.8% at 293 K and 100 bar. In binary mixtures containing 95 mol % CO_2_ and 5 mol % of one of the impurities (CH_4_, Ar, N_2_, and H_2_), a similar pattern of α_*P*_ with a peak value near its saturation pressure was found, as shown in Figure 3c at temperatures of 253 and 313 K. The computed α_*P*_ of binary mixtures CO_2_/N_2_, CO_2_/CH_4_, CO_2_/Ar, and CO_2_/H_2_ agrees fairly with α_*P*_ obtained from the GERG-2008 EoS and has a maximum relative deviation of ca. 13.9% at 313 K and 120 bar, ca. 10.2% at 313 K and 60 bar, ca. 8.1% at 313 K and 60 bar, and ca. 20.4% at 313 K and 120 bar, respectively, excluding state points close to the critical point. State points of binary mixtures close to the critical point were identified based on large uncertainties observed in simulations and large relative deviation with the GERG-2008 EoS. The uncertainties and relative deviation of CO_2_/H_2_ at 313 K decrease with increasing pressure. This suggests that the maximum relative deviation for the CO_2_/H_2_ mixture observed in simulations at 313 K and 120 bar may be near its saturation/Widom line. The binary mixture containing 5 mol % H_2_ increases the value of α_*P*_ the most in liquid and supercritical phases when compared to α_*P*_ of pure CO_2_, followed by binary mixtures containing 5 mol % N_2_, Ar, and CH_4_. In the gas phase, the presence of H_2_ as an impurity decreases the value of α_*P*_ followed by CH_4_, N_2_, and Ar compared to α_*P*_ of pure CO_2_. Thermal expansion coefficients were also computed for 1 mol % and 10 mol % impurities at temperatures of 253, 273, 293, and 313 K from MC simulations. The computed thermal expansion coefficients provided in Tables S73–S167 of the Supporting Information show that the value of α_*P*_ is affected based on impurities and concentration level of impurities. Thermal expansion coefficients of ternary mixtures in the liquid phase with 96 mol % CO_2_ and 2 mol % for each of the two impurities (CH_4_, Ar, N_2_, and H_2_) are shown in Figure 3b. The effect of a particular mixture on α_*P*_ was not discernible since the thermal expansion coefficients of all of the ternary mixtures computed at 253 K were within the limits of computed uncertainty of other ternary mixtures. Similarly, no significant differences were found between the thermal expansion coefficients of ternary mixtures obtained from the GERG-2008 EoS.

#### 4.1.4 Isothermal Compressibilities (β_*T*_)

**Figure 4:**
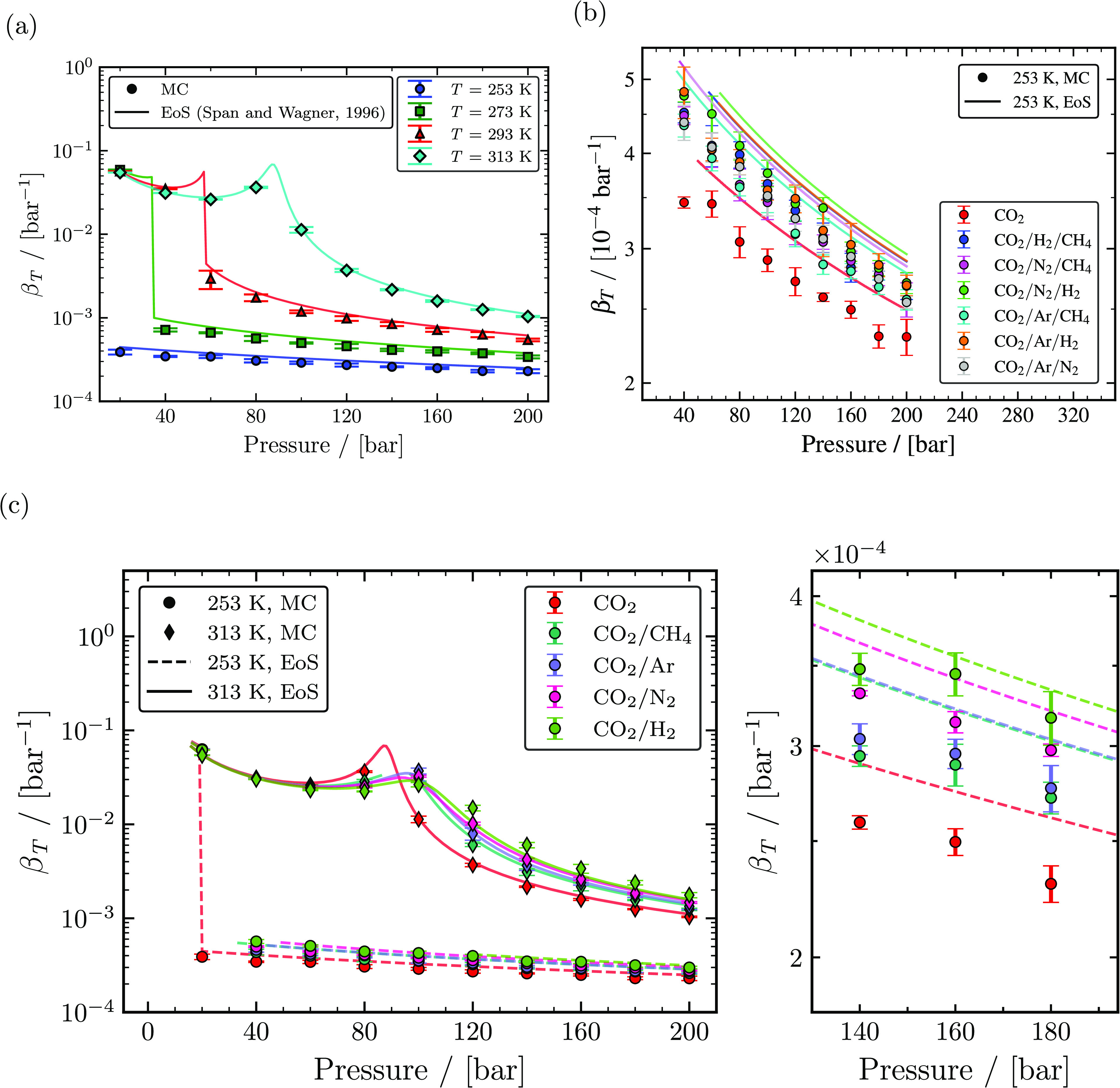
Computed isothermal compressibilities as a function of temperature and pressure. (a) The calculated isothermal compressibilities of pure CO_2_ from MC simulations (closed symbols) and the Span and Wagner EoS^23^ (solid lines) for temperatures of 253, 273, 293, and 313 K. (b) Isothermal compressibilities of ternary mixtures with 96 mol % CO_2_ and 2 mol % impurities for each of two components (CH_4_, Ar, N_2_, and H_2_) computed from MC simulations (closed symbols) and the GERG-2008 EoS^24^ (lines) compared with isothermal compressibilities of pure CO_2_ computed from MC simulations and the Span and Wagner EoS^23^ for temperatures of 253 and 313 K. (c) Isothermal compressibilities of binary mixtures with 95 mol % CO_2_ and 5 mol % impurities (CH_4_, Ar, N_2_, and H_2_) computed from MC simulations (closed symbols) and the GERG-2008 EoS^24^ (lines) compared with isothermal compressibilities of pure CO_2_ computed from MC simulations and the Span and Wagner EoS^23^ at 253 K.

The isothermal compressibility (β_*T*_) computed from MC simulations using eq 5 in the *NPT* ensemble for pure CO_2_, binary CO_2_ mixtures, and ternary CO_2_ rich mixtures with different impurities are shown as a function of temperature and pressure in Figure 4. β_*T*_ values of pure CO_2_ at temperatures of 253, 293, 293, and 313 K are shown in Figure 4a. The maximum value of β_*T*_ was observed in the gas phase. This implies that the volume change rate in response to the change in pressure is maximum when the fluid acts like an ideal gas. β_*T*_ rapidly decreases with an increase in pressure for pressures lower than the saturation pressure of a particular temperature. For pressures away from the saturation/Widom line, the change in the value of β_*T*_ was insignificant for all temperatures, as seen in Figure 4a. Figure 4a also shows that β_*T*_ is dependent on temperature and β_*T*_ increases with increasing temperature. β_*T*_ of pure CO_2_ computed from MC simulations agrees qualitatively with the Span and Wagner EoS with a maximum relative deviation of ca. 19.9% at 293 K and 100 bar. β_*T*_ values of binary mixtures with 95 mol % CO_2_ and 5 mol % of one of the impurities (CH_4_, Ar, N_2_, and H_2_) at temperatures of 253 and 313 K are shown in Figure 4c. Isothermal compressibilities of binary mixtures computed from MC simulations agree fairly with the GERG-2008 EoS. The maximum relative deviations of CO_2_/N_2_, CO_2_/CH_4_, CO_2_/Ar, and CO_2_/H_2_ binary mixtures were ca. 14.1% at 253 and 80 bar, ca. 17.5% at 313 and 120 bar, ca. 12.9% at 253 and 120 bar, and ca. 22.5% at 313 and 140 bar, respectively, excluding state points close to the critical point. Figure 4c shows that the presence of impurity increases β_*T*_ in the liquid and supercritical phases. In contrast, the presence of an impurity decreases β_*T*_ in the gas phase. Comparing the β_*T*_ of all binary mixtures shown in Figure 4c, a binary mixture with H_2_ as an impurity increases the value of β_*T*_ the most in the liquid and supercritical phases, followed by N_2_, Ar, and CH_4_. In the gas phase, a binary mixture with H_2_ as an impurity decreases the value of β_*T*_ followed by the values for N_2_, Ar, and CH_4_. This pattern of β_*T*_ with respect to phases remained the same for both higher (10 mol %) and lower (1 mol %) concentrations of impurities, which is provided in Tables S73–S167 of the Supporting Information. The liquid phase β_*T*_ of ternary mixtures with 96 mol % CO_2_ and 2 mol % for each of the two impurities (CH_4_, Ar, N_2_, and H_2_) at 253 K are shown in Figure 4b. From Figure 4b, it is obvious that the presence of impurities tends to increase the value of β_*T*_. Similar to thermal expansion coefficients, the effect of a particular type of impurity on β_*T*_ was unclear since the change in the value of β_*T*_ due to the presence of impurities was limited. However, β_*T*_ computed from MC simulations for CO_2_ rich ternary mixtures with different impurities agrees fairly with the GERG-2008 EoS. For instance, the CO_2_ rich ternary mixture with Ar and CH_4_ as impurities results in the smallest increase in β_*T*_, while the CO_2_ rich ternary mixture with N_2_ and H_2_ as impurities leads to the largest increase in β_*T*_. The difference in the value of β_*T*_ due to the presence of different impurity combinations estimated from MC simulations was consistent with the GERG-2008 EoS.

#### 4.1.5 Isobaric and Isochoric Heat Capacities

**Figure 5:**
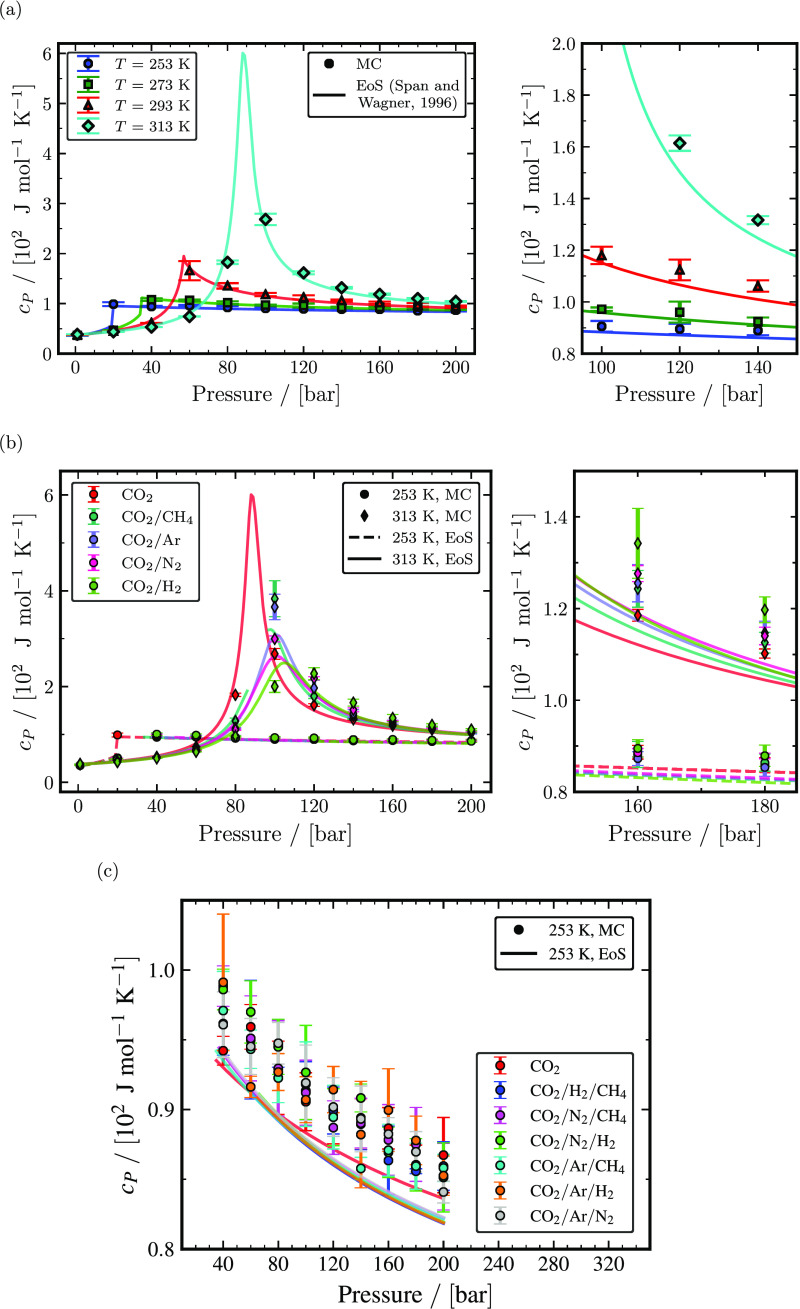
Computed isobaric heat capacities as a function of temperature and pressure. (a) shows the calculated isobaric heat capacities of pure CO_2_ from MC simulations (closed symbols) and the Span and Wagner EoS^23^ (solid lines) for temperatures: 253, 273, 293, and 313 K. (b) shows isobaric heat capacities of binary mixtures with 95 mol % CO_2_ and 5 mol % impurities (CH_4_, Ar, N_2_, and H_2_) computed from MC simulations (closed symbols) and the GERG-2008 EoS^24^ (lines) compared with isobaric heat capacities of pure CO_2_ computed from MC simulations and the Span and Wagner EoS^23^ for temperatures: 253 and 313 K. (c) shows isobaric heat capacities of ternary mixtures with 96 mol % CO_2_ and 2 mol % impurities for each of two components (CH_4_, Ar, N_2_, and H_2_) computed from MC simulations (closed symbols) and the GERG-2008 EoS^24^ (lines) compared with isobaric heat capacities of pure CO_2_ computed from MC simulations and the Span and Wagner EoS^23^ at 253 K.

The constant pressure and volume heat capacities are computed using eqs 3 and 4, respectively. Residual heat capacities *C*_*P*_^residual^ and *C*_*V*_^residual^ are computed from MC simulations in the *NPT* and *NVT* ensemble, respectively, to obtain *c*_*P*_ and *c*_*V*_. Figure 5 shows the *c*_*P*_ of pure CO_2_ and binary CO_2_ mixtures and ternary CO_2_ mixtures as a function of temperature and pressure. *c*_*P*_ of pure CO_2_ computed from MC simulations compared to the Span and Wagner EoS at temperatures of 253, 293, 293, and 313 K are shown in Figure 5a. Heat capacities at a constant pressure of pure CO_2_ are sensitive to pressures closer to the saturation/Widom line, where a sudden surge in *c*_*P*_ is noticed. Figure 5a also shows that *c*_*P*_ increases with temperature. The maximum value of *c*_*P*_ observed in Figure 5a at 313 K is calculated by using the Span and Wagner EoS. However, this peak value of *c*_*P*_ cannot be predicted by MC simulations unless one considers an extremely large system. This is because the correlation length, which measures the spatial extent of spontaneous density fluctuations, diverges near the critical point.^57^*c*_*P*_ of pure CO_2_ computed from MC simulations resulted in minor systematic overprediction with a maximum relative deviation of ca. 6.9% at 313 K and 120 bar. At 20 bar, relative deviations of *c*_*P*_ with the Span and Wagner EoS are less than 1% for all temperatures shown in Figure 5a, except at 253 K. At 253 K and 20 bar, *c*_*P*_ has a relative deviation of ca. 3.8%, which is attributed to the close distance to the vapor–liquid phase transition. *c*_*P*_ of binary mixtures with 95 mol % CO_2_ and 5 mol % of one of the impurities (CH_4_, Ar, N_2_, and H_2_) at temperatures of 253 and 313 K are shown in Figure 5b. Similar to *c*_*P*_ of pure CO_2_, systematic overprediction of *c*_*P*_ is observed in binary mixtures when compared to the GERG - 2008 EoS. The maximum relative deviations of CO_2_/N_2_, CO_2_/CH_4_, CO_2_/Ar, and CO_2_/H_2_ binary mixtures were ca. 8.4% at 313 and 140 bar, ca. 7.3% at 313 and 160 bar, ca. 6.9% at 313 and 180 bar, and ca. 15.7% at 313 and 140 bar, respectively, excluding state points close to the saturation/Widom line. Although *c*_*P*_ values of different binary mixtures may appear to be identical in Figure 5b, impurities altered the value of heat capacities significantly compared to *c*_*P*_ of pure CO_2_. The presence of impurities tends to increase the value of *c*_*P*_ for pressures larger than the pressure at which the heat capacity peaks. Conversely, when the pressure is lower than the pressure at which the heat capacity peaks, the presence of impurities tends to decrease the value of *c*_*P*_. For instance, *c*_*P*_ values of the CO_2_/H_2_ binary mixture at 313 K and 160 bar were ca. 13.2% larger than *c*_*P*_ of pure CO_2_. *c*_*P*_ of the CO_2_/H_2_ binary mixture at 313 K and 60 bar were ca. 13.7% smaller than *c*_*P*_ of pure CO_2_. At conditions away from the saturation/Widom line, a particular type of impurity did not influence *c*_*P*_ to a great extent. However, at conditions close to the saturation/Widom line, the impact of impurities on *c*_*P*_ becomes significant based on the type of impurity where a binary mixture with H_2_ as an impurity increases *c*_*P*_ to a great extent followed by N_2_, Ar, and CH_4_. Similar to binary mixtures with 5 mol % impurities, binary mixtures with 1 and 10 mol % impurities decreased and increased the value of *c*_*P*_ compared to *c*_*P*_ of pure CO_2_ before and after the maximum heat capacity, respectively; see Tables S73–S167 of the Supporting Information. *c*_*P*_ of ternary mixtures with 96 mol % CO_2_ and 2 mol % for each of the two impurities (CH_4_, Ar, N_2_, and H_2_) at 253 K are shown in Figure 5c. From Figure 5c, it is clear that the presence of impurities tends to decrease the *c*_*P*_ at 253 K. Figure 5c also confirms that the effect of a particular type of impurity combination on *c*_*P*_ is insignificant.

**Figure 6:**
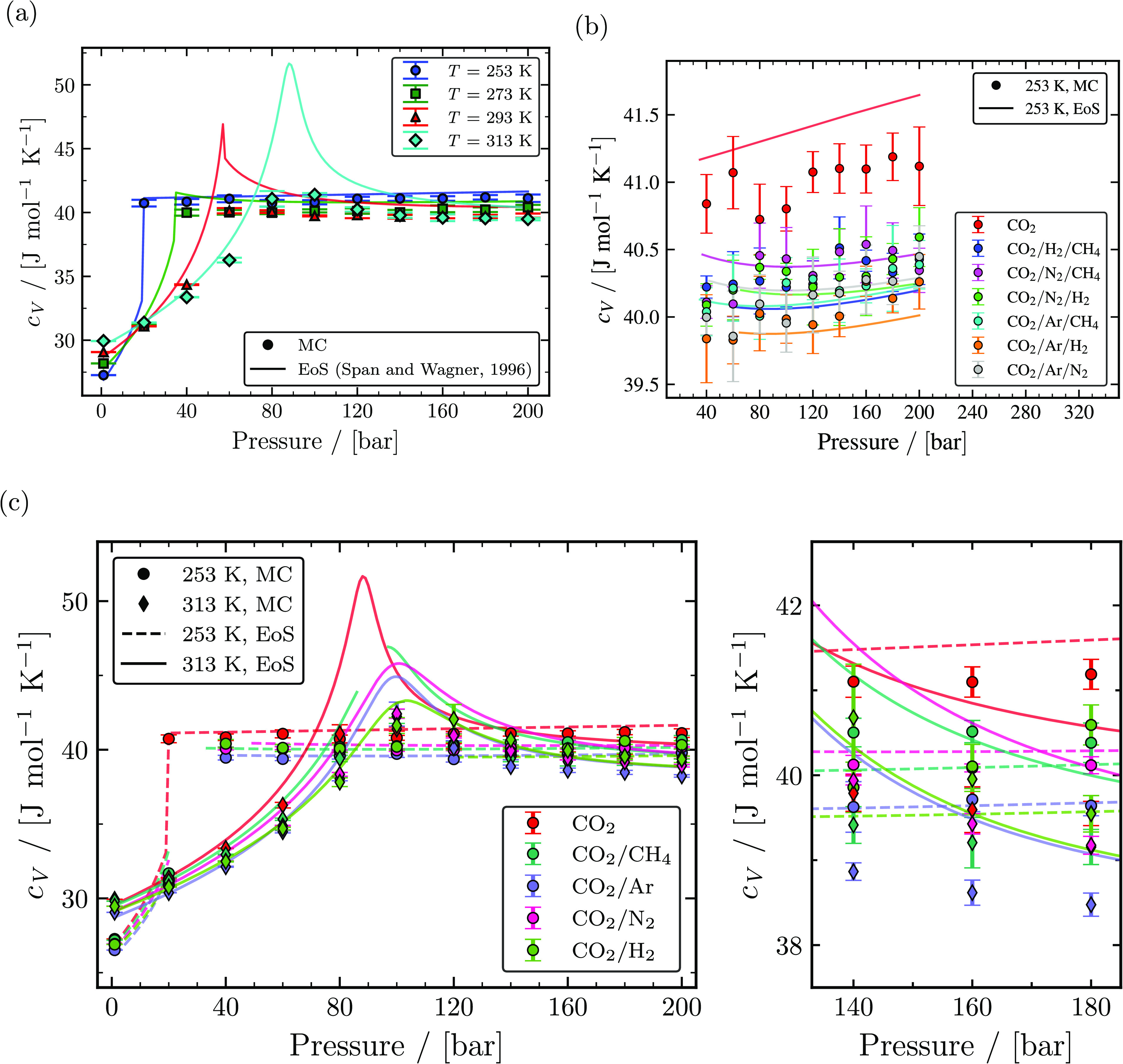
Computed isochoric heat capacities as a function of temperature and pressure. (a) The calculated isochoric heat capacities of pure CO_2_ from MC simulations (closed symbols) and the Span and Wagner EoS^23^ (solid lines) for temperatures of 253, 273, 293, and 313 K. (c) Isochoric heat capacities of binary mixtures with 95 mol % CO_2_ and 5 mol % impurities (CH_4_, Ar, N_2_, and H_2_) computed from MC simulations (closed symbols) and the GERG-2008 EoS^24^ (lines) compared with isochoric heat capacities of pure CO_2_ computed from MC simulations and the Span and Wagner EoS^23^ for temperatures of 253 and 313 K. (b) Isochoric heat capacities of ternary mixtures with 96 mol % CO_2_ and 2 mol % impurities for each of two components (CH_4_, Ar, N_2_, and H_2_) computed from MC simulations (closed symbols) and the GERG-2008 EoS^24^ (lines) compared with isochoric heat capacities of pure CO_2_ computed from MC simulations and the Span and Wagner EoS^23^ at 253 K.

Heat capacities at constant volume of pure CO_2_ at temperatures of 253, 293, 293, and 313 K are compared with the Span and Wagner EoS in Figure 6a. The computed *c*_*V*_ is in excellent agreement with *c*_*V*_ obtained from the Span and Wagner EoS with a maximum relative deviation of ca. 5% at 313 K and 120 bar considering conditions away from the saturation/Widom line. *c*_*V*_ values of pure CO_2_ vary insignificantly for pressures larger than its saturation pressure irrespective of the temperature. Similarly, *c*_*V*_ of binary mixtures with 95 mol % CO_2_ and 5 mol % of one of the impurities (CH_4_, Ar, N_2_, and H_2_) at temperatures of 253 and 313 K seen in Figure 6c have no significant difference in the value of *c*_*V*_ between 253 and 313 K for pressures larger than its saturation pressure. The presence of an impurity tends to decrease *c*_*V*_ hardly, irrespective of the type of impurity. *c*_*V*_ of ternary mixtures with 96 mol % CO_2_ and 2 mol % for each of the two impurities (CH_4_, Ar, N_2_, and H_2_) at 253 K are shown in Figure 6b. In the reduced axis range seen in Figure 6c, it is clear that the presence of impurities (in CO_2_/Ar/H_2_) decreases the *c*_*V*_ maximum by ca. 3%. It is important to mention that *c*_*V*_ can be calculated from *c*_*P*_ using eq 15. However, the indirect computation of *c*_*V*_ using eq 15 is prone to high statistical uncertainties,^90,91^ which will subsequently affect the calculations of speed of sound. Hence, constant volume heat capacities are computed by sampling eq 3 in the *NVT* ensemble with a volume of the state obtained from the *NPT* simulation.

#### 4.1.6 Joule–Thomson Coefficients (μ_JT_)

**Figure 7:**
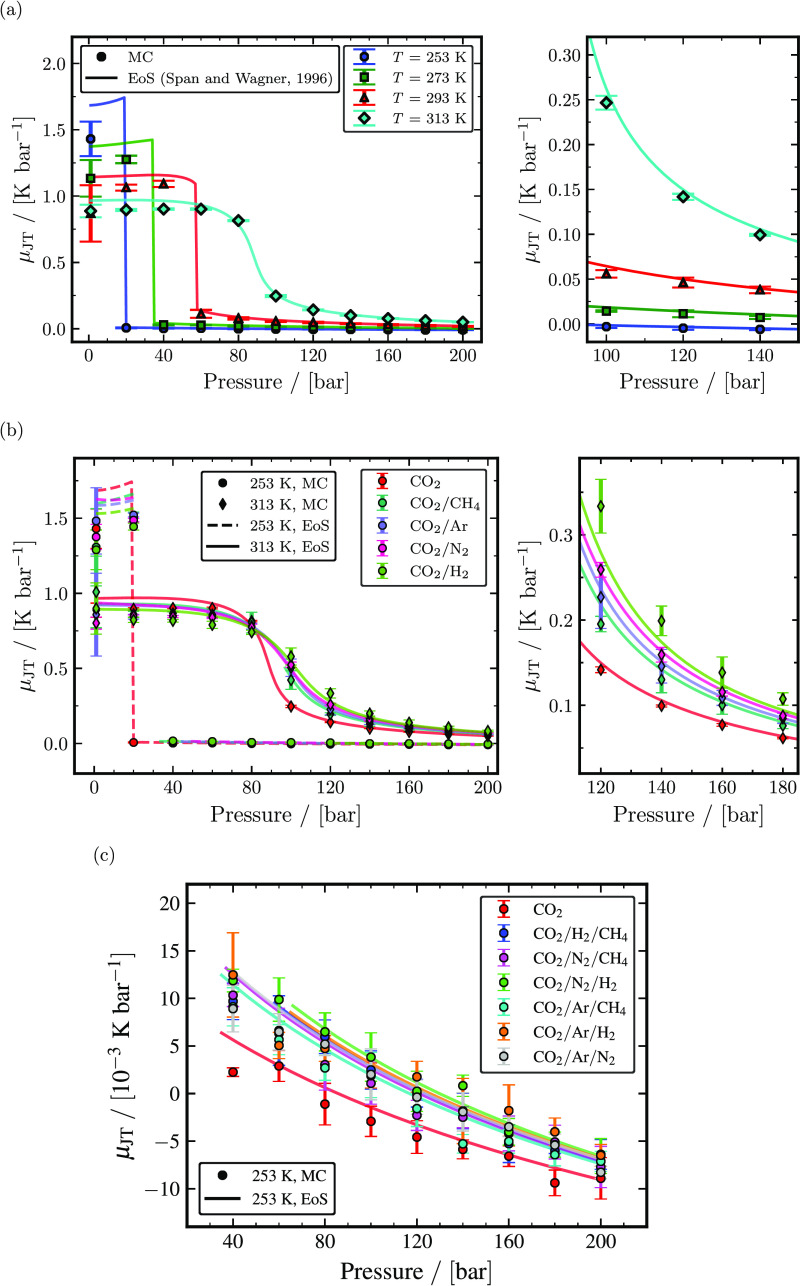
Computed Joule–Thomson coefficients as a function of temperature and pressure. (a) The calculated Joule–Thomson coefficients of pure CO_2_ from MC simulations (closed symbols) and the Span and Wagner EoS^23^ (solid lines) for temperatures of 253, 273, 293, and 313 K. (b) Joule–Thomson coefficients of binary mixtures with 95 mol % CO_2_ and 5 mol % impurities (CH_4_, Ar, N_2_, and H_2_) computed from MC simulations (closed symbols) and the GERG-2008 EoS^24^ (lines) compared with Joule–Thomson coefficients of pure CO_2_ computed from MC simulations and the Span and Wagner EoS^23^ for temperatures of 253 and 313 K. (c) Joule–Thomson coefficients of ternary mixtures with 96 mol % CO_2_ and 2 mol % impurities for each of two components (CH_4_, Ar, N_2_, and H_2_) computed from MC simulations (closed symbols) and the GERG-2008 EoS^24^ (lines) compared with Joule–Thomson coefficients of pure CO_2_ computed from MC simulations and the Span and Wagner EoS^23^ at 253 K.

Joule–Thomson coefficients (μ_JT_) of pure CO_2_, binary CO_2_ mixtures, and ternary CO_2_ rich mixtures computed using eq 12 as a function of the temperature and pressure are shown in Figure 7. μ_JT_ decreases as the temperature increases in the gas phase. Conversely, μ_JT_ increases with the temperature in the liquid and supercritical phases. The computed μ_JT_ of pure CO_2_ at temperatures of 253, 293, 293, and 313 K seen in Figure 7a agrees decently with μ_JT_ obtained from the Span and Wagner EoS. The deviations of μ_JT_ in the gas phase are larger when compared to the liquid and supercritical phases, and uncertainties of μ_JT_ are quite significant for extremely low pressures. This is because of eq 12 used for computing μ_JT_. Joule–Thomson coefficients are computed indirectly using eq 12, and its uncertainties are computed using eq S84 presented in Section S11 of the Supporting Information. The uncertainties computed using eq S84 depend on the precision and accuracy of α_*P*_ and *c*_*P*_. In particular, computation of μ_JT_ is highly sensitive to α_*P*_. Even an insignificant error in the α_*P*_ computation might result in high uncertainties and deviations in μ_JT_. A similar drawback of using eq 12 is also reported in ref (92). μ_JT_ of binary mixtures with 95 mol % CO_2_ and 5 mol % of one of the impurities (CH_4_, Ar, N_2_, and H_2_) at temperatures of 253 and 313 K are shown in Figure 7b. The value of μ_JT_ decreases due to the presence of impurities in the gas phase and increases in the liquid and supercritical phases. H_2_ has the most significant effect on the values of μ_JT_, followed by those of N_2_, Ar, and CH_4_. μ_JT_ computed from MC simulations for binary mixtures rich in CO_2_ with 1 mol %, 5 mol %, and 10 mol % impurities (CH_4_, Ar, N_2_, and H_2_) at 253, 273, 293, and 313 K listed in Tables S73–S167 of the Supporting Information also shows the same qualitative trend with respect to impurity type. μ_JT_ listed in Tables S73–S167 of the Supporting Information also indicates that the impact of impurities increases with increasing concentration of impurities. μ_JT_ of ternary mixtures with 96 mol % CO_2_ and 2 mol % for each of the two impurities (CH_4_, Ar, N_2_, and H_2_) at 253 K are shown in Figure 7c. μ_JT_ of ternary mixtures computed from MC simulations at 253 K agrees well with the μ_JT_ obtained from the GERG-2008 EoS, considering the uncertainty range. Figure 7c also shows that the presence of impurities tends to increase the μ_JT_ of ternary mixtures significantly. The impact of impurities is crucial and should be considered in the initial computations, especially at conditions where the inversion of μ_JT_ takes place. For instance, μ_JT_ inversion occurs close to 80 bar for pure CO_2_ at 253 K, but for impure CO_2_ ternary mixtures at 253 K, μ_JT_ inversion occurs at pressures larger than 120 bar.

#### 4.1.7 Speed of Sound (*c*)

**Figure 8:**
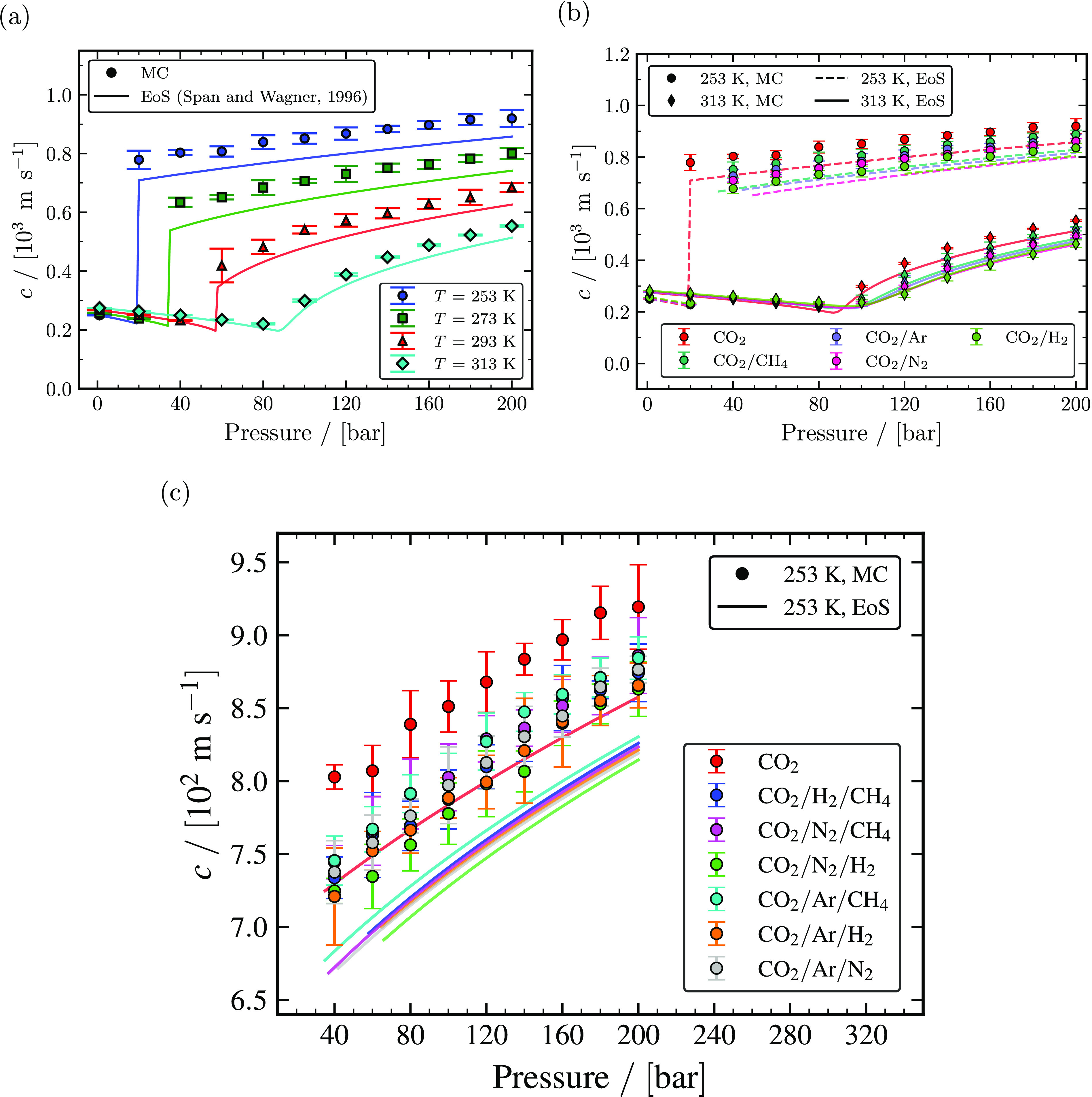
Computed speed of sound as a function of temperature and pressure. (a) The calculated speed of sound of pure CO_2_ from MC simulations (closed symbols) and the Span and Wagner EoS^23^ (solid lines) for temperatures of 253, 273, 293, and 313 K. (b) The speed of sound of binary mixtures with 95 mol % CO_2_ and 5 mol % impurities (CH_4_, Ar, N_2_, and H_2_) computed from MC simulations (closed symbols) and the GERG-2008 EoS^24^ (lines) compared with the speed of sound of pure CO_2_ computed from MC simulations and the Span and Wagner EoS^23^ for temperatures of 253 and 313 K. (c) The speed of sound of ternary mixtures with 96 mol % CO_2_ and 2 mol % impurities for each of two components (CH_4_, Ar, N_2_, and H_2_) computed from MC simulations (closed symbols) and the GERG-2008 EoS^24^ (lines) compared with the speed of sound of pure CO_2_ computed from MC simulations and the Span and Wagner EoS^23^ at 253 K.

The speed of sound (*c*) of pure CO_2_, binary CO_2_ mixtures, and ternary CO_2_ rich mixtures computed as a function of temperature and pressure using eq 1 from MC simulations is shown in Figure 8. Since *c* is a function of ρ, β_*T*_, *c*_*P*_, and *c*_*V*_, uncertainties associated with *c* are computed using eq S78 derived in Section S11 of the Supporting Information. The speed of sound of pure CO_2_ computed at temperatures of 253, 293, 293, and 313 K results in overprediction compared to *c* obtained using the Span and Wagner EoS. Since *c* depends on ρ, β_*T*_, *c*_*P*_, and *c*_*V*_, small deviations in these dependent properties will overestimate *c*. The overestimation of *c* is majorly due to the minor overestimation of *c*_*P*_ and underestimation of β_*T*_. For example, *c* of pure CO_2_ is overestimated by a relative deviation of ca. 6.7% at 253 K and 200 bar, while the overestimation of *c*_*P*_ is ca. 3.6% and the underestimation of β_*T*_ is ca. 8.4%. The magnitude of the overprediction was found to be less than 10% for all temperatures and pressures. Figure 8a shows that *c* decreases marginally until the pressure reaches its critical pressure and increases significantly for the pressure larger than the saturation pressure for a particular temperature. Figure 8a also shows that *c* is temperature dependent and decreases with increasing temperature. The impacts of impurities on *c* are evaluated by comparing *c* computed from MC simulation and the GERG-2008 EoS for binary mixtures consisting of 95 mol % CO_2_ and 5 mol % of one of the impurities (CH_4_, Ar, N_2_, and H_2_) as shown in Figure 8b. In the liquid and supercritical phases, the presence of H_2_ as an impurity is observed to have the largest impact on decreasing the value of *c*, followed by those of CH_4_, Ar, and N_2_. In the gas phase, the presence of H_2_ as an impurity is observed to have the largest impact on increasing the value of *c* followed by that of CH_4_, Ar, and N_2_. The computed *c* of binary mixtures rich in CO_2_ with 1 mol % and 10 mol % impurity (CH_4_, Ar, N_2_, and H_2_) for temperatures of 253, 273, 293, and 313 K from MC simulations and the GERG-2008 EoS, as listed in Tables S73–S167 of the Supporting Information, shows the same behavior with respect to type of impurity. Speeds of sound of ternary mixtures with 96 mol % CO_2_ and 2 mol % for each of the two impurities (CH_4_, Ar, N_2_, and H_2_) at 253 K are shown in Figure 8c. MC simulations overpredicted the value of *c* compared to the GERG-2008 EoS. However, MC simulations closely predicted the decrease in the value of *c* due to the presence of impurities. For instance, the difference in the value of *c* at 200 bar between pure CO_2_ and a ternary CO_2_ mixture with N_2_ and H_2_ as impurities is ca. 56 m/s from MC simulations and ca. 46 m/s from the GERG-2008 EoS.

### 4.2 Transport Property

#### 4.2.1 Viscosities (η)

**Figure 9:**
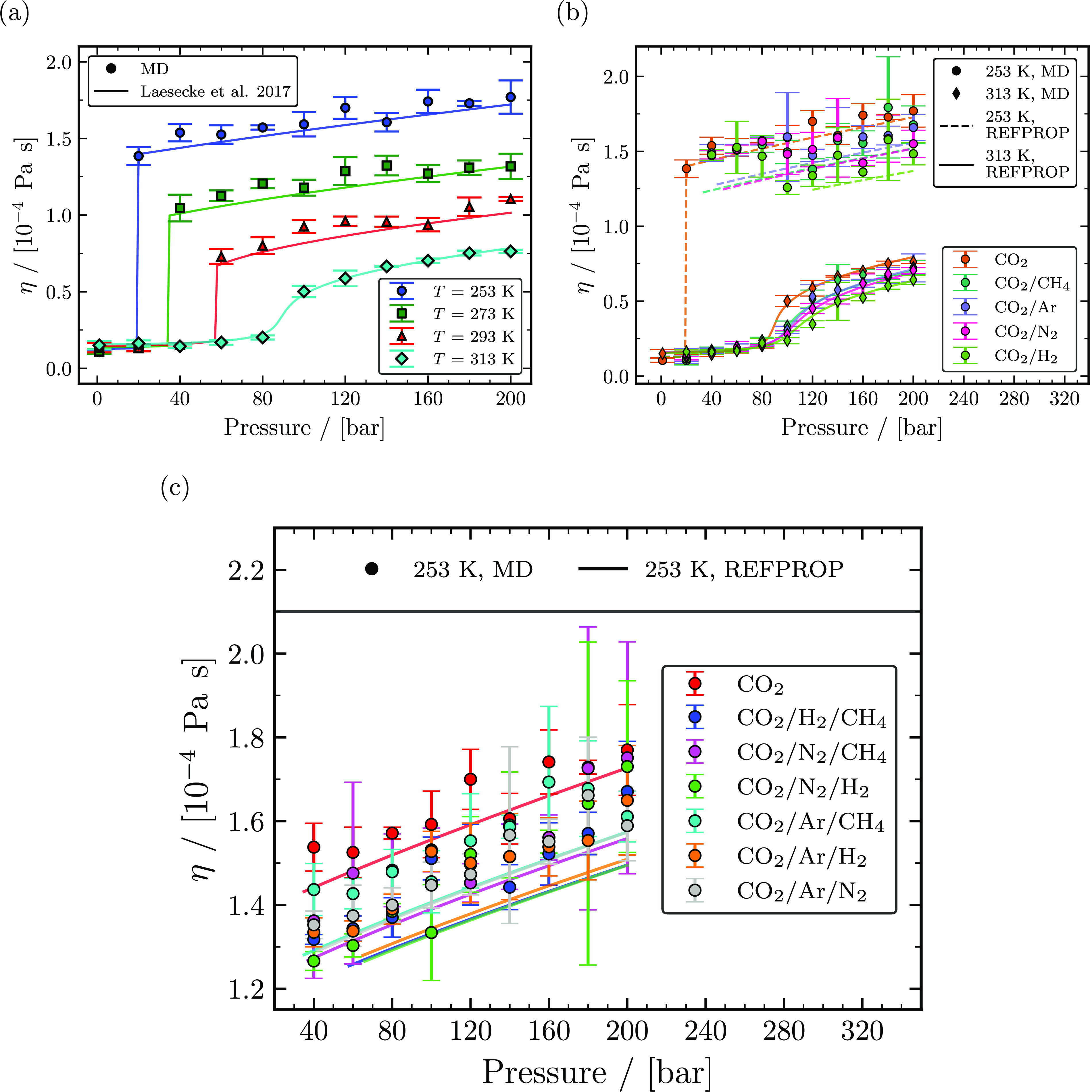
Computed viscosities as a function of temperature and pressure. (a) The calculated viscosities of pure CO_2_ computed from MD simulations (closed symbols) compared with the correlation of Laesecke et al. (2017)^25^ (solid lines) for temperatures of 253, 273, 293, and 313 K. (b) Viscosities of binary mixtures with 95 mol % CO_2_ and 5 mol % impurities (CH_4_, Ar, N_2_, and H_2_) computed from MD simulations (closed symbols) and REFPROP^72^ (lines) compared with viscosities of pure CO_2_ computed from MD simulations and REFPROP^72^ for temperatures of 253 and 313 K. (c) Viscosities of ternary mixtures with 96 mol % CO_2_ and 2 mol % impurities for each of two components (CH_4_, Ar, N_2_, and H_2_) computed from MD simulations (closed symbols) and REFPROP^72^ (lines) compared with viscosities of pure CO_2_ computed from MD simulations and REFPROP^72^ at 253 K.

Viscosities (η) of pure CO_2_, binary CO_2_ mixtures, and ternary CO_2_ rich mixtures computed from MD simulations as a function of temperature and pressure are shown in Figure 9. Viscosities of pure CO_2_ were computed at temperatures of 253, 273, 293, and 313 K. The computed viscosities are compared with those calculated from the correlation of Laesecke et al.^25^ Our results show a good agreement with the model for all temperatures as seen in Figure 9a with a maximum relative deviation of ca. 14.6% in the gas phase (at 313 K and 40 bar), excluding state points close to the saturation/Widom line. η at pressures below the saturation pressure for a temperature remains relatively constant, but for pressures far away from the saturation/Widom line, η increases with an increasing pressure at a constant temperature. η is observed to be highly dependent on temperature compared to pressure as seen in Figure 9a, where η decreases with increasing temperature. To analyze the effect of impurities, η of binary mixtures with 95 mol % CO_2_ and 5 mol % of one of the impurities (CH_4_, Ar, N_2_, and H_2_) computed from MD simulations at 253 and 313 K are compared with viscosities obtained from REFPROP in Figure 9b. Computed η of binary CO_2_ mixtures qualitatively correlate well with η obtained from REFPROP. The existence of an impurity in a CO_2_ mixture tends to reduce η compared to η of pure CO_2_. Based on η computed from MD simulations and REFPROP, it is clear that a binary mixture with H_2_ as an impurity decreases η the most in liquid and supercritical phases. The uncertainty range of computed viscosities from MD simulations made it difficult to interpret a particular type of impurity that impacts η the most next to H_2_. However, viscosities obtained from REFPROP indicate that a binary mixture with N_2_ as an impurity decreases η after H_2_ followed by CH_4_ and Ar for pressure away from the saturation/Widom line at 253 and 313 K. In addition to viscosity data of binary mixtures with 5 mol % impurities, binary mixtures with 1 mol % and 10 mol % impurities (CH_4_, Ar, N_2_, and H_2_) at temperatures of 253, 273, 293, and 313 K computed from MD simulations are listed along with data obtained from REFPROP in Tables S73–S167 of the Supporting Information. To analyze the effect on η due to the presence of a particular combination of impurities in CO_2_ rich mixtures, η of ternary mixtures were computed from MD simulations and compared with η obtained from REFPROP at 253 K, as shown in Figure 9c. η of all ternary mixtures shown in Figure 9c has a concentration of 96 mol % CO_2_ and 2 mol % for each of the two impurities (CH_4_, Ar, N_2_, and H_2_). Comparing η of ternary mixtures obtained from REFPROP, it is clear that mixtures with H_2_ as an impurity reduce the liquid viscosities of CO_2_ mixtures to a larger extent compared to mixtures without H_2_. A similar qualitative trend of η was also observed from simulations at 40, 60, and 80 bar. For higher pressures, considering the computed uncertainties, the effect of a particular combination of impurities was difficult to evaluate, with a marginal decrease in the liquid viscosities. Uncertainties associated with viscosities can be reduced by performing multiple independent simulations, but this will increase computational costs.^93^ We refrained from conducting additional simulations due to the marginal difference in liquid viscosities observed between ternary mixtures in Figure 9c.

In summary, we performed molecule simulations of CO_2_ rich mixtures with N_2_, Ar, CH_4_, and H_2_ as impurities using the force fields mentioned in Section S1 of the Supporting Information. The thermodynamic and transport properties were computed at temperatures of 253, 273, 239, and 313 K and pressures up to 200 bar for pure CO_2_ and binary mixtures rich in CO_2_ with 1 mol %, 5 mol %, and 10 mol % impurities. The computed thermodynamic and transport properties were found to be in good agreement with the EoS for pure and binary systems, except at conditions close to the saturation/Widom line. The thermodynamic and transport properties were also computed for 24 ternary and 12 quaternary CO_2_ rich mixtures for various concentrations of impurities listed in Tables S169 and S362 of the Supporting Information, respectively, for temperatures of 253, 273, 239, and 313 K and pressures up to 200 bar. Results of the thermodynamic and transport properties of ternary and quaternary mixtures are provided in Sections S16 and S17 of the Supporting Information, respectively. The thermodynamic and transport properties of multicomponent CO_2_ mixtures were compared with the GERG-2008 EoS^24^ and the ECS model,^84^ respectively, and showed good agreement. We show that molecular simulations are a powerful tool to compute the thermodynamic and transport properties of multicomponent mixtures seen in the transportation of CO_2_ with a smaller system of 300 molecules. These thermophysical properties will help in modeling and designing pipelines for CO_2_ transportation, which will be the focus of further work.

## 5 Conclusions

In this study, the effect of impurities in CO_2_ rich mixtures on the value of thermodynamic and transport properties such as densities, thermal expansion coefficients, isothermal compressibilities, heat capacities at constant pressure, heat capacities at constant volume, Joule–Thomson coefficients, speed of sound, and viscosities were investigated using molecular simulations. The CFCMC method was used to compute the VLE of pure components, such as CO_2_, CH_4_, Ar, N_2_, and H_2_, to validate force fields used in molecular simulations. The computed VLE of pure components showed an excellent agreement with the EoS.^23,94−97^ The phase equilibria of CO_2_/Ar, CO_2_/CH_4_, CO_2_/N_2_, and CO_2_/H_2_ binary mixtures were also computed using the CFCMC method and compared with the GERG-2008 EoS^24^ and data from the literature,^85−89^ showing a good agreement. The thermodynamic and transport properties were computed for pure CO_2_ and binary and ternary mixtures rich in CO_2_ at temperatures of 253, 273, 293, and 313 K and for pressures ranging from 20 to 200 bar using MC and MD simulations. The computed thermodynamic and transport properties of pure CO_2_ are in excellent agreement with corresponding values obtained from the Span and Wagner EoS.^23^ Thermodynamic and transport properties of CO_2_ rich binary mixtures with 1 mol %, 5 mol %, and 10 mol % concentrations of noncondensable impurities such as CH_4_, Ar, N_2_, and H_2_ computed from simulations showed a good agreement with the GERG-2008 EoS.^24^ The computed thermodynamic and transport properties of CO_2_ rich ternary mixtures with various impurities were compared with the GERG-2008 EoS,^24^ showing a good agreement. The effect of different types of impurities on specific thermodynamic and transport properties was evaluated. Our findings show that CO_2_ rich mixtures with impurities have low densities compared to densities of pure CO_2_. The magnitude of reduction in densities of a CO_2_ rich mixture depends strongly on the molecular weight of impurities present in a mixture. Mixtures with molecular weight lower than pure CO_2_ were observed to have reduced densities compared to pure CO_2_. CO_2_ rich mixtures containing H_2_ as an impurity led to the most significant decrease in the value of thermal expansion coefficients, isothermal compressibilities, heat capacities at constant pressure, and Joule–Thomson coefficients followed by N_2_, Ar, and CH_4_ in the gas phase. In the liquid and supercritical phases, the presence of H_2_ as an impurity led to the most significant increase in the values of thermal expansion coefficients, isothermal compressibilities, heat capacities at constant pressure, and Joule–Thomson coefficients followed by N_2_, Ar, and CH_4_. In contrast, the presence of H_2_ as an impurity in the CO_2_ rich mixture increased the value of speed of sound in the gas phase and decreased it in the liquid and supercritical phases. The order of effect due to a particular impurity on thermal expansion coefficients, isothermal compressibilities, heat capacities at constant pressure, Joule–Thomson coefficients, and speed of sound correlates with the critical temperature of impurities. In our investigation of heat capacities at constant volume, we found that the presence of impurities did not have a significant impact. Finally, differences in the values of viscosities in CO_2_ rich mixtures due to the presence of impurities were evaluated. Our findings showed that mixtures containing H_2_ as an impurity significantly reduced viscosities in liquid and supercritical phases.

## Data Availability

Data will be made available on reasonable request.

## Supporting Information Available

Force field parameters of the molecules used in both MC and MD simulations, Vapor Liquid Equilibrium (VLE) curves of unary systems computed from MC simulations, derivation of the heat capacity at constant volume, derivation of the heat capacity at constant pressure, derivation of the isothermal compressibility, derivation of the thermal expansivity, derivation of the speed of sound, derivation of the Joule–Thomson coefficient, computation of the uncertainty in the speed of sound and Joule–Thomson coefficient, data of the ideal gas heat capacity at constant volume, plots of thermodynamic and transport properties of unary systems, plots of thermodynamic and transport properties of binary systems, data of thermodynamics and transport properties of binary systems, data of thermodynamics and transport properties of ternary systems, and data of thermodynamic and transport properties of quaternary systems (PDF)
