# Supplementary material for: Thermophysical Properties and Phase Behavior of CO2 with Impurities: Insight from Molecular Simulations
Source: J Chem Eng Data. 2024 Jul 12;69(8):2735–55. doi: 10.1021/acs.jced.4c00268 (PMC11318637; doi:10.1021/acs.jced.4c00268)
Supplement: Supplementary file 1 — je4c00268_si_001.pdf [file je4c00268_si_001.pdf]

Supporting Information for: "Thermophysical Properties and Phase Behavior of CO_2 with Impurities: Insight from Molecular Simulations"

D. Raju, M. Ramdin, and T.J.H. Vlugt*

Engineering Thermodynamics, Process & Energy Department, Faculty of Mechanical Engineering, Delft University of Technology, Leeghwaterstraat 39, Delft 2628CB, The Netherlands

E-mail: T.J.H.Vlugt@tudelft.nl

The following items are presented in this Supplementary Information:

- Force field parameters of the molecules used in both MC and MD simulations (Tables S1 to S7)
- Vapor Liquid Equilibrium (VLE) curves of unary systems computed from MC simulations
- Derivation of the heat capacity at constant volume (C_V)
- Derivation of the heat capacity at constant pressure (C_P)
- Derivation of the isothermal compressibility (β_T)
- Derivation of the thermal expansivity (α_P)
- Derivation of the speed of sound (c)
- Derivation of Joule-Thomson Coefficient (μ_{JT})
- Computation of the uncertainty in the speed of sound (Δc) and Joule-Thomson coefficient ($\Delta \mu_{\mathrm{JT}}$)
- Data of the ideal gas heat capacity at constant volume (c_V^{ideal})
- Plots of thermodynamic and transport properties of unary systems
- Plots of thermodynamic and transport properties of binary systems
- Data of thermodynamics and transport properties of binary systems
- Data of thermodynamics and transport properties of ternary systems
- Data of thermodynamic and transport properties of quaternary systems

S1 Force field parameters of the molecules used in molecular simulations

Table S1: The atom types and coordinates of the TraPPE CO_2 molecule.[1]

Atom type	x/[Å]	y/[Å]	z/[Å]
C	1.16	0.00	0.00
O	0.00	0.00	0.00
O	2.32	0.00	0.00

Table S2: The TraPPE force field parameters for CO_2.[1]

Atom type	ε/k_B / [K]	σ/[Å]	q/[e]
O	79.0	3.05	-0.35
C	27.0	2.80	0.70

Table S3: The atom types and coordinates of the TraPPE N_2 molecule.[1]

Atom type	x/[Å]	y/[Å]	z/[Å]
N	0.00	0.00	0.00
N-M[a]	0.55	0.00	0.00
N	1.10	0.00	0.00

N-M[a] represent the dummy charge site in the TraPPE force field of N_2.[1]

Table S4: The TraPPE force field parameters of N_2.[1]

Atom type	ε/k_B / [K]	σ/[Å]	q/[e]
N	36.0	3.310	-0.482
N-M[a]	0.00	0.00	0.964

N-M[a] represent a point charge (0.964e) is placed at the centre of mass of N_2.

Table S5: Force field parameters of single-site argon. The force field parameters of García-Pérez et al.[2] commensurate with tail corrections have been used for Ar.

Atom type	ε/k_B / [K]	σ/[Å]	q/[e]
Ar	115.00	3.38	0.00

Table S6: Force field parameters of hydrogen. The single-site force field parameters of Köster et al.[3] have been used for H_2.

Atom type	ε/k_B / [K]	σ/[Å]	q/[e]
H_2	25.84	3.0366	0.00

Table S7: Force field parameters of methane. The single-site force field parameters from TraPPE were used for CH_4.[4]

Atom type	ε/k_B / [K]	σ/[Å]	q/[e]
CH_4	148.00	3.73	0.00

S2 Vapor Liquid Equilibria (VLE) of unary systems

The VLE of the unary systems listed in Table S8 are computed from the NVT version of the Continuous Fractional Component Gibbs Ensemble (CFCGE)[5–9] and compared with the VLE obtained form REFPROP[10] which uses a particular EoS model for pure components. The EoS models specific to the component are listed in Table S8. Using the coexistence densities computed from CFCMC simulations, critical temperatures of components were calculated following the scaling law[11] .

$$\rho_l - \rho_g = \quad A(T - T_c)^\beta \tag{S1}$$

where ρ_l is the liquid density, ρ_g is the gas density, T_c is the critical temperature, A is a fit parameter, and β is the critical exponent ($\beta = 0.32$ for three-dimensional systems[12]). The critical density was calculated using the law of rectilinear diameters.[13]

$$\frac{\rho_l - \rho_g}{2} = \quad \rho_c + B(T - T_c) \tag{S2}$$

where ρ_c is the critical density and B is a fit parameter.

Table S8: VLEs of the unary systems listed are computed in the NVT version of the CFCGE and validated with the corresponding EoS and correlation models using REFPROP.[10]

Component	CAS number	EoS	Correlation models
CO_2	124-38-9	Span and Wagner (1996)[14]	Laesecke et al. (2017)[15]
N_2	7727-37-9	Span et al. (2000)[16]	Lemmon and Jacobsen (2004)[17]
Ar	7440-37-1	Tegeler et al. (1999)[18]	Lemmon and Jacobsen (2004)[17]
H_2	1333-74-0	Leachman et al. (2009)[19]	Munzy et al. (2013)[20]
CH_4	74-82-8	Setzmann and Wagner (1991)[21]	Quinones-Cisneros et al. (2011)[22]

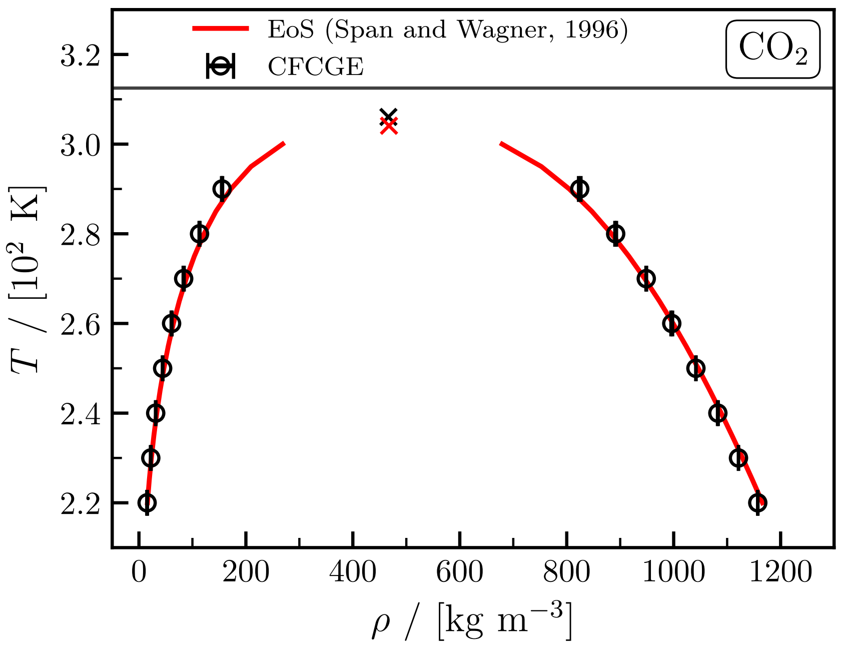

Fig. S1: The VLE of carbon dioxide computed from MC simulations in the *NVT* version of the CFCGE using the TraPPE forcefield for CO_2.[1] The circles represent MC simulations, and the line represents the EoS data from Span and Wagner (1996).[14]

From all the VLE curves of the molecules shown in Figs. S1 to S3, it is evident that MC simulations are in excellent agreement with the EoS models, except the VLE of H_2 seen in Fig. S3 (a). This can be attributed to the domination of the quantum effects at low temperatures.[3] To obtain the liquid densities correct at low temperatures, quantum effects have to be considered.[23] However, temperatures considered in this study ($> 250\,\mathrm{K}$) are much higher than the coexistence temperatures of H_2 so quantum effects are not relevant. Therefore, only the gas densities were considered for the validation of H_2 force field.

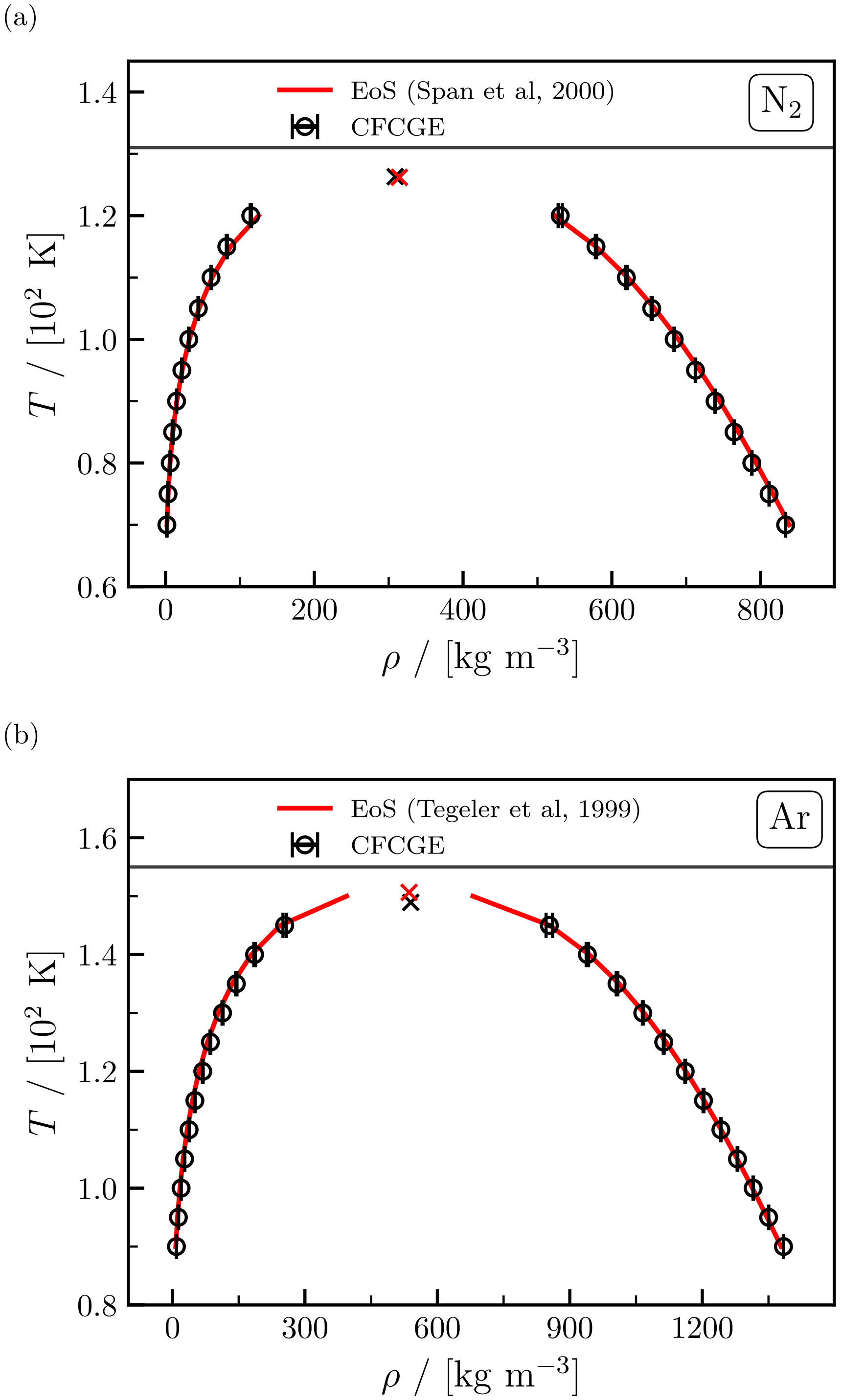

Fig. S2: The VLE of (a) nitrogen and (b) argon computed from MC simulations in the *NVT* version of the CFCGE using the TraPPE forcefield for N_2[1] and the García-Pérez et al.[2] model for Ar. The circles represent MC simulations, and the line represents the EoS data from Span et al. (2000)[16] for N_2 and Tegeler et al. (1999)[18] for Ar.

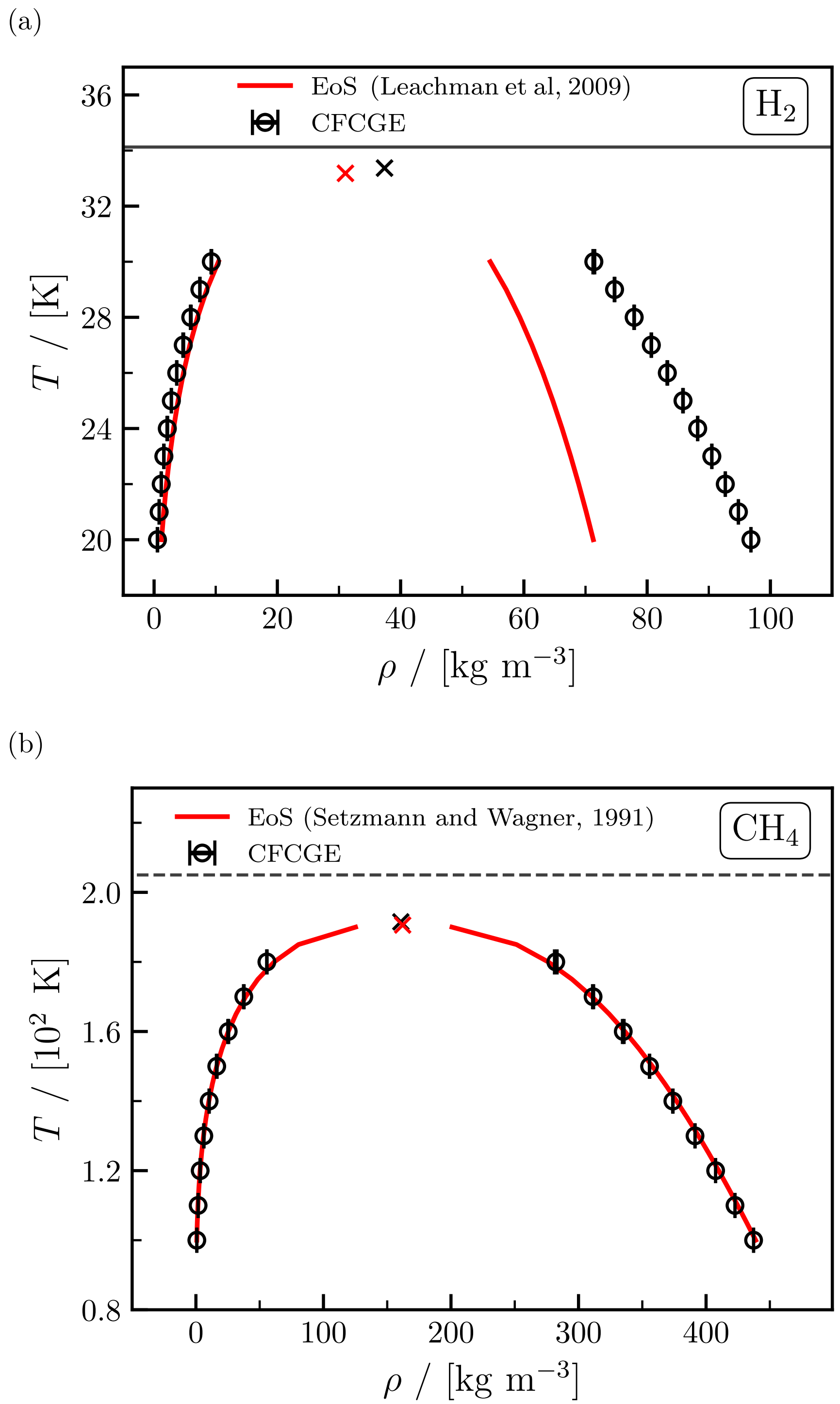

Fig. S3: The VLE of (a) hydrogen and (b) methane computed from MC simulations in the *NVT* version of the CFCGE using the Köster model for H_2[3] and TraPPE force field[4] for CH_4. The circles represent MC simulations, and the line represents the EoS data from Leachman et al. (2009)[19] for H_2 and Setzmann and Wagner (1991)[21] for CH_4.

S3 VLE data of unary systems

The raw VLE data, computed from the *NVT* version of the Continuous Fractional Component Gibbs Ensemble (CFCGE)[5–9,24] with uncertainties and VLE obtained from REFPROP[10] for the unary systems listed in Table S8 are shown in this section.

Table S9: Coexistence densities of carbon dioxide computed from CFCGE simulations ($\rho_{\text{liquid}}^{\text{CFCGE}}$ and $\rho_{\text{gas}}^{\text{CFCGE}}$) using the TraPPE model[1] and coexistence densities of carbon dioxide obtained from REFPROP[10] ($\rho_{\text{liquid}}^{\text{REFP}}$ and $\rho_{\text{gas}}^{\text{REFP}}$).

T/[K]	$\rho_{\text{liquid}}^{\text{REFP}}$/[kg m^{-3}]	$\rho_{\text{liquid}}^{\text{CFCGE}}$/[kg m^{-3}]	$\rho_{\text{gas}}^{\text{REFP}}$/[kg m^{-3}]	$\rho_{\text{gas}}^{\text{CFCGE}}$/[kg m^{-3}]
220	1166.1	1157.3 ± 0.7	15.817	15.3 ± 0.3
230	1128.7	1121.2 ± 0.7	23.271	22.1 ± 0.3
240	1088.9	1082.6 ± 0.5	33.295	31.3 ± 0.8
250	1046.0	1041.6 ± 0.6	46.644	44.3 ± 0.4
260	998.89	996.4 ± 0.9	64.417	61 ± 2
270	945.83	949 ± 1	88.374	83.6 ± 0.7
280	883.58	891.5 ± 0.9	121.74	113 ± 2
290	804.67	824 ± 1	171.96	155 ± 2

Table S10: Coexistence densities of nitrogen computed from CFCGE simulations ($\rho_{\text{liquid}}^{\text{CFCGE}}$ and $\rho_{\text{gas}}^{\text{CFCGE}}$) using TraPPE model[1] and coexistence densities of nitrogen obtained from REFPROP[10] ($\rho_{\text{liquid}}^{\text{REFP}}$ and $\rho_{\text{gas}}^{\text{REFP}}$).

T/[K]	$\rho_{\text{liquid}}^{\text{REFP}}$/[kg m^{-3}]	$\rho_{\text{liquid}}^{\text{CFCGE}}$/[kg m^{-3}]	$\rho_{\text{gas}}^{\text{REFP}}$/[kg m^{-3}]	$\rho_{\text{gas}}^{\text{CFCGE}}$/[kg m^{-3}]
70	838.51	833.6 ± 0.3	1.8960	1.8 ± 0.1
75	816.67	811.4 ± 0.4	3.5404	3.37 ± 0.09
80	793.94	788.2 ± 0.3	6.0894	6.3 ± 0.3
85	770.13	764.3 ± 0.6	9.8241	9.5 ± 0.3
90	745.02	738.8 ± 0.2	15.079	14.9 ± 0.2
95	718.26	712.4 ± 0.3	22.272	22.0 ± 0.3
100	689.35	683.8 ± 0.4	31.961	31.3 ± 0.4
105	657.52	653.6 ± 0.4	44.959	44.1 ± 0.8
110	621.45	619.5 ± 0.2	62.579	61 ± 1
115	578.70	578.7 ± 0.7	87.294	82.4 ± 0.9
120	523.36	530.6 ± 0.8	125.09	115 ± 3

Table S11: Coexistence densities of argon computed from CFCGE simulations ($\rho_{\text{liquid}}^{\text{CFCGE}}$ and $\rho_{\text{gas}}^{\text{CFCGE}}$) using García-Pérez et al.[2] model and coexistence densities of argon obtained from REFPROP[10] ($\rho_{\text{liquid}}^{\text{REFP}}$ and $\rho_{\text{gas}}^{\text{REFP}}$).

$T/[\text{K}]$	$\rho_{\text{liquid}}^{\text{REFP}}/[\text{kg m}^{-3}]$	$\rho_{\text{liquid}}^{\text{CFCGE}}/[\text{kg m}^{-3}]$	$\rho_{\text{gas}}^{\text{REFP}}/[\text{kg m}^{-3}]$	$\rho_{\text{gas}}^{\text{CFCGE}}/[\text{kg m}^{-3}]$
90	1378.6	1384.0 ± 0.3	7.4362	8.9 ± 0.1
95	1346.8	1350.4 ± 0.6	11.435	13.3 ± 0.3
100	1313.7	1315.3 ± 0.4	16.859	19.5 ± 0.4
105	1279.1	1279.7 ± 0.6	24.019	27.4 ± 0.7
110	1242.8	1242.2 ± 0.4	33.287	37.6 ± 0.4
115	1204.2	1202.8 ± 0.7	45.126	51 ± 1
120	1162.8	1161.3 ± 0.9	60.144	68.6 ± 0.8
125	1117.9	1112.7 ± 0.9	79.194	86 ± 1
130	1068.1	1065 ± 1	103.56	113 ± 1
135	1011.5	1007 ± 2	135.39	145 ± 2
140	943.71	940 ± 2	178.86	186 ± 3
145	854.28	854 ± 4	244.44	254 ± 7

Table S12: Coexistence densities of hydrogen computed from CFCGE simulations ($\rho_{\text{liquid}}^{\text{CFCGE}}$ and $\rho_{\text{gas}}^{\text{CFCGE}}$) using Köster et al.[3] model and coexistence densities of hydrogen obtained from REFPROP[10] ($\rho_{\text{liquid}}^{\text{REFP}}$ and $\rho_{\text{gas}}^{\text{REFP}}$).

$T/[\text{K}]$	$\rho_{\text{liquid}}^{\text{REFP}}/[\text{kg m}^{-3}]$	$\rho_{\text{liquid}}^{\text{CFCGE}}/[\text{kg m}^{-3}]$	$\rho_{\text{gas}}^{\text{REFP}}/[\text{kg m}^{-3}]$	$\rho_{\text{gas}}^{\text{CFCGE}}/[\text{kg m}^{-3}]$
20	71.265	96.84 ± 0.03	1.2059	0.55 ± 0.02
21	70.115	94.81 ± 0.01	1.5701	0.82 ± 0.02
22	68.893	92.68 ± 0.02	2.0090	1.16 ± 0.02
23	67.592	90.50 ± 0.03	2.5334	1.59 ± 0.03
24	66.199	88.20 ± 0.05	3.1562	2.14 ± 0.02
25	64.701	85.82 ± 0.02	3.8938	2.80 ± 0.05
26	63.079	83.28 ± 0.03	4.7674	3.68 ± 0.05
27	61.305	80.68 ± 0.06	5.8055	4.73 ± 0.04
28	59.339	77.90 ± 0.06	7.0489	5.95 ± 0.04
29	57.119	74.71 ± 0.03	8.5601	7.43 ± 0.05
30	54.538	71.35 ± 0.05	10.445	9.3 ± 0.1

Table S13: Coexistence densities of methane computed from CFCGE simulations ($\rho_{\text{liquid}}^{\text{CFCGE}}$ and $\rho_{\text{gas}}^{\text{CFCGE}}$) using TraPPE model[4] and coexistence densities of methane obtained from REFPROP[10] ($\rho_{\text{liquid}}^{\text{REFP}}$ and $\rho_{\text{gas}}^{\text{REFP}}$).

T/[K]	$\rho_{\text{liquid}}^{\text{REFP}}$/[kg m^{-3}]	$\rho_{\text{liquid}}^{\text{CFCGE}}$/[kg m^{-3}]	$\rho_{\text{gas}}^{\text{REFP}}$/[kg m^{-3}]	$\rho_{\text{gas}}^{\text{CFCGE}}$/[kg m^{-3}]
100	438.89	437.27 ± 0.08	0.674 57	0.73 ± 0.03
110	424.78	422.69 ± 0.07	1.5982	1.73 ± 0.03
120	409.90	407.5 ± 0.2	3.2619	3.35 ± 0.03
130	394.04	391.3 ± 0.2	5.9804	6.21 ± 0.09
140	376.87	374.0 ± 0.1	10.152	10.4 ± 0.2
150	357.90	355.7 ± 0.3	16.328	16.3 ± 0.1
160	336.31	335.1 ± 0.4	25.382	25.3 ± 0.7
170	310.50	311.4 ± 0.3	38.974	37.5 ± 0.4
180	276.23	282.2 ± 0.2	61.375	56 ± 1

S4 VLE data of binary systems

The phase equilibria (Pxy) of binary systems listed in Table S14 are computed in the NPT version of the Continuous Fractional Component Gibbs Ensemble (CFCGE).[5–9,24] The computed raw Pxy data with uncertainties of binary systems from simulations are shown in this section. The Pxy data from experiments and REFPROP[10] of the binary systems used for comparing the computed Pxy data are also shown in this section.

Table S14: Binary systems for which the phase equilibria are computed in the NPT version of the CFCGE. The computed Pxy data of binary systems were validated with experimental data and the GERG-2008 EoS model[30] obtained from REFPROP.[10]

No.	Binary system	Experimental data (Reference)
1	CO_2/N_2	Brown et al., 1989[25]
2	CO_2/Ar	Coquelet et al., 2008[26]
3	CO_2/H_2	Tsang and Street., 1981[27]
4	CO_2/CH_4	Davalos et al., 1976[28]
		Wei et al., 1995[29]

S4.1 VLE data of CO_2-N_2 system

Table S15: Pxy data of the CO_2-N_2 binary system computed from the NPT version of the CFCGE simulations at 250 K.

P/[bar]	x_{CO_2}	y_{CO_2}
35	0.9795 ± 0.0008	0.60 ± 0.01
45	0.965 ± 0.002	0.488 ± 0.008
55	0.952 ± 0.002	0.44 ± 0.01
65	0.939 ± 0.002	0.399 ± 0.005
75	0.925 ± 0.002	0.374 ± 0.007
85	0.909 ± 0.002	0.357 ± 0.007
95	0.897 ± 0.002	0.355 ± 0.005

Table S16: Pxy data of the CO_2-N_2 binary system obtained from REFPROP[10] using the GERG-2008 EoS[30] at 250 K.

Bubble points		Dew points	
P/[bar]	x_{CO_2}	P/[bar]	y_{CO_2}
17.88	1.0000	17.85	1.0000
17.90	0.9999	17.86	0.9995
17.93	0.9999	17.90	0.9978
17.98	0.9998	17.93	0.9964
18.04	0.9997	17.98	0.9943
18.12	0.9996	18.04	0.9916
18.21	0.9994	18.12	0.9883
18.31	0.9993	18.21	0.9841
18.41	0.9991	18.31	0.9798
18.51	0.9990	18.41	0.9756
18.61	0.9988	18.51	0.9714
18.71	0.9987	18.61	0.9673
18.81	0.9985	18.71	0.9632
18.91	0.9984	18.81	0.9592
19.01	0.9982	18.91	0.9552
19.11	0.9981	19.01	0.9512
19.21	0.9979	19.11	0.9473
19.31	0.9978	19.21	0.9435
19.71	0.9971	19.31	0.9396
20.11	0.9965	19.51	0.9321
20.51	0.9959	19.71	0.9247
20.91	0.9952	19.91	0.9175
21.31	0.9946	20.11	0.9105
21.71	0.9940	20.31	0.9035
22.11	0.9933	20.51	0.8967
22.51	0.9927	20.71	0.8901
22.91	0.9921	20.91	0.8835
23.31	0.9914	21.11	0.8771
23.71	0.9908	21.31	0.8708
24.11	0.9901	21.51	0.8646
24.51	0.9895	21.71	0.8586
24.91	0.9888	21.91	0.8526
25.31	0.9882	22.11	0.8468
25.71	0.9875	22.31	0.8410
26.11	0.9869	22.51	0.8354
26.71	0.9859	22.71	0.8298
27.31	0.9849	22.91	0.8244
27.91	0.9839	23.11	0.8190
28.51	0.9829	23.31	0.8137

Table S16: Pxy data of the CO_2-N_2 binary system obtained from REFPROP[10] using the GERG-2008 EoS[30] at 250 K.

Bubble points		Dew points	
P/[bar]	x_{CO_2}	P/[bar]	y_{CO_2}
29.11	0.9819	23.51	0.8085
29.71	0.9809	23.71	0.8034
30.31	0.9799	23.91	0.7984
30.91	0.9789	24.21	0.7910
31.51	0.9779	24.51	0.7838
32.11	0.9769	24.81	0.7767
32.71	0.9758	25.11	0.7699
33.31	0.9748	25.41	0.7631
33.91	0.9738	25.71	0.7566
34.51	0.9728	26.01	0.7501
35.11	0.9717	26.31	0.7438
35.71	0.9707	26.61	0.7377
36.31	0.9697	26.91	0.7316
36.91	0.9686	27.21	0.7257
37.51	0.9676	27.51	0.7200
38.11	0.9665	27.81	0.7143
38.71	0.9655	28.41	0.7033
39.31	0.9644	28.71	0.6980
39.91	0.9634	29.01	0.6928
40.51	0.9623	29.31	0.6877
41.11	0.9613	29.61	0.6827
41.71	0.9602	30.01	0.6761
42.31	0.9591	30.81	0.6636
42.91	0.9581	31.21	0.6575
43.51	0.9570	31.61	0.6516
44.11	0.9559	32.01	0.6458
44.71	0.9549	32.41	0.6402
45.31	0.9538	32.81	0.6347
45.91	0.9527	33.61	0.6241
46.51	0.9516	34.01	0.6190
47.11	0.9505	34.51	0.6128
47.71	0.9494	35.01	0.6067
48.31	0.9483	35.51	0.6009
48.91	0.9472	36.01	0.5951
49.51	0.9461	36.51	0.5896
50.11	0.9450	37.01	0.5842
50.71	0.9439	37.51	0.5789
51.31	0.9428	38.01	0.5738
51.91	0.9417	38.61	0.5678

Table S16: Pxy data of the CO_2-N_2 binary system obtained from REFPROP[10] using the GERG-2008 EoS[30] at 250 K.

Bubble points		Dew points	
P/[bar]	x_{CO_2}	P/[bar]	y_{CO_2}
52.51	0.9406	39.21	0.5620
53.11	0.9394	39.81	0.5564
53.71	0.9383	41.01	0.5457
54.31	0.9372	41.61	0.5406
54.91	0.9360	42.21	0.5356
55.51	0.9349	42.81	0.5308
56.11	0.9338	43.41	0.5262
56.71	0.9326	44.01	0.5216
57.31	0.9315	44.61	0.5172
57.91	0.9303	45.21	0.5129
58.51	0.9292	45.81	0.5088
59.11	0.9280	46.41	0.5047
59.71	0.9269	47.01	0.5008
60.31	0.9257	47.61	0.4970
60.91	0.9245	48.21	0.4933
61.51	0.9233	48.81	0.4897
62.11	0.9222	49.41	0.4862
62.71	0.9210	50.01	0.4828
63.31	0.9198	50.61	0.4795
63.91	0.9186	51.21	0.4762
64.51	0.9174	51.81	0.4731
65.11	0.9162	52.41	0.4700
65.71	0.9150	53.01	0.4670
66.31	0.9138	53.61	0.4641
66.91	0.9126	54.21	0.4613
67.51	0.9114	54.81	0.4586
68.11	0.9102	55.41	0.4559
68.71	0.9090	56.01	0.4533
69.31	0.9077	56.61	0.4508
69.91	0.9065	57.21	0.4483
70.51	0.9053	57.81	0.4459
71.11	0.9040	58.41	0.4436
71.71	0.9028	59.01	0.4413
72.31	0.9016	59.61	0.4391
72.91	0.9003	60.21	0.4369
73.51	0.8991	60.81	0.4348
74.11	0.8978	61.41	0.4328
74.71	0.8965	62.01	0.4308
75.31	0.8953	62.61	0.4288

Table S16: *Pxy* data of the CO_2-N_2 binary system obtained from REFPROP[10] using the GERG-2008 EoS[30] at 250 K.

Bubble points		Dew points	
P/[bar]	x_{CO_2}	P/[bar]	y_{CO_2}
75.91	0.8940	63.21	0.4269
76.51	0.8927	63.81	0.4251
77.11	0.8914	64.41	0.4233
77.71	0.8901	65.01	0.4216
78.31	0.8888	65.61	0.4199
78.91	0.8875	66.21	0.4182
79.51	0.8862	66.81	0.4166
80.11	0.8849	67.41	0.4151
80.71	0.8836	68.01	0.4135
81.31	0.8823	68.61	0.4121
81.91	0.8810	69.21	0.4106
82.51	0.8797	69.81	0.4092
83.11	0.8783	70.41	0.4079
83.71	0.8770	71.01	0.4065
84.31	0.8756	71.61	0.4053
84.91	0.8743	72.21	0.4040
85.51	0.8729	72.81	0.4028
86.11	0.8716	73.41	0.4016
86.71	0.8702	74.01	0.4005
87.31	0.8688	74.61	0.3994
87.91	0.8675	75.21	0.3983
88.51	0.8661	75.81	0.3973
89.11	0.8647	76.41	0.3963
89.71	0.8633	77.61	0.3944
90.31	0.8619	78.81	0.3926
90.91	0.8605	79.41	0.3917
91.51	0.8591	80.01	0.3909
92.11	0.8576	81.21	0.3893
92.71	0.8562	82.41	0.3879
93.31	0.8548	83.01	0.3872
93.91	0.8533	83.61	0.3865
94.51	0.8519	84.21	0.3859
95.11	0.8504	84.81	0.3853
95.71	0.8490	85.41	0.3847
96.31	0.8475	86.01	0.3842
96.91	0.8460	86.61	0.3836
97.51	0.8446	87.21	0.3831
98.11	0.8431	87.81	0.3827
98.71	0.8416	88.41	0.3822

Table S16: *Pxy* data of the CO_2-N_2 binary system obtained from REFPROP[10] using the GERG-2008 EoS[30] at 250 K.

Bubble points		Dew points	
P/[bar]	x_{CO_2}	P/[bar]	y_{CO_2}
99.31	0.8401	89.01	0.3818
99.91	0.8386	89.61	0.3814
100.51	0.8370	90.21	0.3810
101.11	0.8355	90.81	0.3806
101.71	0.8340	91.41	0.3803
102.31	0.8324	92.01	0.3800
102.91	0.8309	92.61	0.3797
103.51	0.8293	93.01	0.3795
104.11	0.8278	93.41	0.3793
104.71	0.8262	93.81	0.3792
105.31	0.8246	94.21	0.3790
105.91	0.8230	94.61	0.3789
106.51	0.8214	95.01	0.3788
107.11	0.8198	95.41	0.3786
107.71	0.8182	95.81	0.3785
108.31	0.8165	96.21	0.3784
108.91	0.8149	96.61	0.3783
109.51	0.8132	97.01	0.3782
110.11	0.8116	98.21	0.3781
110.71	0.8099	98.61	0.3780
111.31	0.8082	99.41	0.3780
111.91	0.8065	99.71	0.3779
112.51	0.8048	101.71	0.3780
113.11	0.8031	102.11	0.3780
113.71	0.8014	103.31	0.3782
114.31	0.7997	104.51	0.3784
114.91	0.7979	105.31	0.3787
115.51	0.7962	106.11	0.3789
116.11	0.7944	106.91	0.3792
116.71	0.7926	107.51	0.3794
117.31	0.7908	108.71	0.3800
117.91	0.7890	109.91	0.3806
118.51	0.7872	110.51	0.3810
119.11	0.7853	111.71	0.3817
119.71	0.7835	112.31	0.3821
120.31	0.7816	113.51	0.3830
120.91	0.7797	114.11	0.3835
121.51	0.7778	115.31	0.3845
122.11	0.7759	116.51	0.3856

Table S16: *Pxy* data of the CO_2-N_2 binary system obtained from REFPROP[10] using the GERG-2008 EoS[30] at 250 K.

Bubble points		Dew points	
P/[bar]	x_{CO_2}	P/[bar]	y_{CO_2}
122.71	0.7740	117.11	0.3862
123.31	0.7721	118.31	0.3875
123.91	0.7701	119.51	0.3888
124.51	0.7681	120.11	0.3895
125.11	0.7661	120.71	0.3903
125.71	0.7641	121.31	0.3910
126.31	0.7621	123.11	0.3935
127.01	0.7597	124.31	0.3952
127.71	0.7573	125.51	0.3971
128.41	0.7549	126.11	0.3980
129.11	0.7524	127.31	0.4001
129.81	0.7499	128.51	0.4022
130.51	0.7474	129.11	0.4033
131.21	0.7448	130.31	0.4057
131.91	0.7422	131.51	0.4081
132.61	0.7396	132.11	0.4094
133.31	0.7369	132.71	0.4108
134.01	0.7342	133.31	0.4121
134.71	0.7314	133.91	0.4135
135.41	0.7287	134.51	0.4149
136.11	0.7258	135.11	0.4164
136.81	0.7230	135.71	0.4179
137.51	0.7200	136.31	0.4195
138.21	0.7171	136.91	0.4211
138.91	0.7140	137.51	0.4227
139.61	0.7110	138.11	0.4244
140.31	0.7078	138.71	0.4261
141.01	0.7046	139.31	0.4279
141.71	0.7014	139.91	0.4297
142.41	0.6980	140.61	0.4319
143.11	0.6946	141.31	0.4342
143.81	0.6911	142.01	0.4366
144.51	0.6875	142.71	0.4390
145.21	0.6838	143.41	0.4415
145.91	0.6800	144.11	0.4441
146.61	0.6761	144.81	0.4468
147.31	0.6721	145.51	0.4496
148.01	0.6679	146.21	0.4525
148.71	0.6636	146.91	0.4556

Table S16: *Pxy* data of the CO_2-N_2 binary system obtained from REFPROP[10] using the GERG-2008 EoS[30] at 250 K.

Bubble points		Dew points	
P/[bar]	x_{CO_2}	P/[bar]	y_{CO_2}
149.41	0.6591	147.61	0.4587
150.11	0.6544	148.31	0.4621
150.81	0.6495	149.01	0.4655
151.51	0.6442	149.71	0.4692
152.21	0.6387	150.41	0.4731
152.81	0.6337	151.11	0.4772
153.41	0.6283	151.81	0.4816
154.01	0.6224	152.51	0.4864
154.51	0.6171	153.21	0.4916
155.01	0.6113	153.91	0.4973
155.41	0.6062	154.51	0.5028
155.81	0.6005	155.51	0.5138
156.21	0.5940	155.91	0.5191
156.47	0.5890	156.30	0.5254
156.71	0.5835	156.60	0.5312
156.91	0.5780	156.83	0.5365
157.01	0.5745	156.97	0.5410
157.04	0.5731	157.03	0.5431
157.05	0.5727	157.05	0.5438
157.09	0.5729	157.09	0.5505

Table S17: Experimental *Pxy* data of the CO_2-N_2 binary system from Brown et al., 1989[25] at 250 K.

P/[bar]	x_{CO_2}	y_{CO_2}
25.99	0.9864	0.7439
36.88	0.9669	0.5761
45.89	0.9512	0.4999
55.41	0.9336	0.4506
65.97	0.9130	0.4151
75.32	0.8931	0.3951
96.32	0.8468	0.3777
111.51	0.8069	0.3837
119.36	0.7827	0.3937
134.16	0.7292	0.4273
140.66	0.6992	0.4563

S4.2 VLE data of CO_2-Ar system

Table S18: *Pxy* data of the CO_2-Ar binary system computed from the *NPT* version of the CFCGE simulations at 253 K.

P/[bar]	x_{CO_2}	y_{CO_2}
35.00	0.969 ± 0.001	0.637 ± 0.009
45.00	0.948 ± 0.002	0.524 ± 0.008
55.00	0.925 ± 0.001	0.456 ± 0.005
65.00	0.900 ± 0.002	0.411 ± 0.006
75.00	0.879 ± 0.003	0.392 ± 0.007
85.00	0.855 ± 0.002	0.376 ± 0.003
95.00	0.827 ± 0.007	0.36 ± 0.01
105.00	0.790 ± 0.006	0.353 ± 0.004

Table S19: *Pxy* data of the CO_2-Ar binary system obtained from REFPROP[10] using the GERG-2008 EoS[30] at 253 K.

Bubble points		Dew points	
P/[bar]	x_{CO_2}	P/[bar]	y_{CO_2}
19.62	1.0000	19.61	1.0000
19.64	0.9999	19.64	0.9988
19.66	0.9999	19.66	0.9977
19.70	0.9998	19.70	0.9962
19.75	0.9997	19.75	0.9941
19.82	0.9996	19.82	0.9913
19.91	0.9994	19.91	0.9877
20.01	0.9992	20.01	0.9837
20.11	0.9990	20.11	0.9798
20.21	0.9988	20.21	0.9759
20.31	0.9986	20.31	0.9721
20.41	0.9984	20.41	0.9683
20.51	0.9982	20.51	0.9645
20.61	0.9980	20.61	0.9607
20.71	0.9978	20.71	0.9570
20.81	0.9976	20.81	0.9533
20.91	0.9974	20.91	0.9496
21.01	0.9972	21.01	0.9460
21.11	0.9970	21.11	0.9424
21.51	0.9962	21.51	0.9282
22.11	0.9950	22.11	0.9078
22.71	0.9938	22.71	0.8884
23.31	0.9925	23.31	0.8698

Table S19: Pxy data of the CO_2-Ar binary system obtained from REFPROP[10] using the GERG-2008 EoS[30] at 253 K.

Bubble points		Dew points	
P/[bar]	x_{CO_2}	P/[bar]	y_{CO_2}
23.91	0.9913	23.91	0.8522
24.51	0.9901	24.51	0.8353
25.11	0.9889	25.11	0.8191
25.71	0.9877	25.71	0.8037
26.31	0.9865	26.31	0.7889
26.91	0.9853	26.91	0.7748
27.51	0.9841	27.51	0.7612
28.11	0.9828	28.11	0.7482
28.71	0.9816	28.71	0.7358
29.31	0.9804	29.31	0.7238
29.91	0.9792	29.91	0.7123
30.51	0.9779	30.51	0.7013
31.11	0.9767	31.11	0.6906
31.71	0.9755	31.71	0.6804
32.31	0.9742	32.51	0.6674
32.91	0.9730	32.91	0.6611
33.51	0.9717	33.31	0.6550
34.11	0.9705	33.71	0.6490
34.71	0.9692	34.11	0.6432
35.31	0.9680	34.51	0.6375
35.91	0.9667	34.91	0.6319
36.51	0.9655	35.31	0.6265
37.11	0.9642	35.71	0.6212
37.71	0.9629	36.11	0.6160
38.31	0.9616	36.51	0.6109
38.91	0.9604	37.51	0.5987
39.51	0.9591	38.01	0.5929
40.11	0.9578	38.51	0.5872
40.71	0.9565	40.01	0.5711
41.31	0.9552	40.51	0.5659
41.91	0.9539	41.11	0.5600
42.51	0.9526	41.71	0.5542
43.11	0.9513	42.31	0.5486
43.71	0.9500	42.91	0.5432
44.31	0.9487	43.51	0.5380
44.91	0.9474	44.11	0.5328
45.51	0.9461	44.71	0.5279
46.11	0.9447	45.31	0.5231
46.71	0.9434	45.91	0.5184

Table S19: *Pxy* data of the CO_2-Ar binary system obtained from REFPROP[10] using the GERG-2008 EoS[30] at 253 K.

Bubble points		Dew points	
P/[bar]	x_{CO_2}	P/[bar]	y_{CO_2}
47.31	0.9421	46.51	0.5139
47.91	0.9407	47.11	0.5094
48.51	0.9394	47.71	0.5051
49.11	0.9381	48.31	0.5010
49.71	0.9367	48.91	0.4969
50.31	0.9354	49.51	0.4930
50.91	0.9340	50.11	0.4891
51.51	0.9326	50.71	0.4854
52.11	0.9313	51.31	0.4818
52.71	0.9299	51.91	0.4782
53.31	0.9285	52.51	0.4748
53.91	0.9271	53.11	0.4714
54.51	0.9257	53.71	0.4682
55.11	0.9244	54.31	0.4650
55.71	0.9230	54.91	0.4619
56.31	0.9216	55.51	0.4588
56.91	0.9201	56.11	0.4559
57.51	0.9187	56.71	0.4530
58.11	0.9173	57.31	0.4502
58.71	0.9159	57.91	0.4475
59.31	0.9145	58.51	0.4449
59.91	0.9130	59.11	0.4423
60.51	0.9116	59.71	0.4398
61.11	0.9101	60.31	0.4373
61.71	0.9087	60.91	0.4349
62.31	0.9072	61.51	0.4326
62.91	0.9058	62.11	0.4303
63.51	0.9043	62.71	0.4280
64.11	0.9028	63.31	0.4259
64.71	0.9014	63.91	0.4238
65.31	0.8999	64.51	0.4217
65.91	0.8984	65.11	0.4197
66.51	0.8969	65.71	0.4177
67.11	0.8954	66.31	0.4158
67.71	0.8939	66.91	0.4139
68.31	0.8924	67.51	0.4121
68.91	0.8908	68.11	0.4104
69.51	0.8893	68.71	0.4086
70.11	0.8878	69.31	0.4069

Table S19: Pxy data of the CO_2-Ar binary system obtained from REFPROP[10] using the GERG-2008 EoS[30] at 253 K.

Bubble points		Dew points	
P/[bar]	x_{CO_2}	P/[bar]	y_{CO_2}
70.71	0.8863	69.91	0.4053
71.31	0.8847	70.51	0.4037
71.91	0.8832	71.11	0.4021
72.51	0.8816	71.71	0.4006
73.11	0.8800	72.31	0.3991
73.71	0.8785	72.91	0.3977
74.31	0.8769	73.51	0.3963
74.91	0.8753	74.11	0.3949
75.51	0.8737	74.71	0.3936
76.11	0.8721	75.31	0.3923
76.71	0.8705	75.91	0.3910
77.31	0.8689	76.51	0.3898
77.91	0.8673	77.11	0.3886
78.51	0.8656	77.71	0.3874
79.11	0.8640	78.31	0.3863
79.71	0.8624	78.91	0.3852
80.31	0.8607	79.51	0.3841
80.91	0.8590	80.11	0.3830
81.51	0.8574	80.71	0.3820
82.11	0.8557	81.31	0.3810
82.71	0.8540	81.91	0.3801
83.31	0.8523	82.51	0.3792
83.91	0.8506	83.11	0.3783
84.51	0.8489	83.71	0.3774
85.11	0.8472	84.31	0.3766
85.71	0.8455	84.91	0.3757
86.31	0.8437	85.51	0.3749
86.91	0.8420	86.11	0.3742
87.51	0.8402	86.71	0.3735
88.11	0.8385	87.31	0.3727
88.71	0.8367	87.91	0.3721
89.31	0.8349	88.51	0.3714
89.91	0.8331	89.11	0.3708
90.51	0.8314	89.71	0.3701
91.11	0.8295	90.31	0.3696
91.71	0.8277	90.91	0.3690
92.31	0.8259	91.51	0.3685
92.91	0.8241	92.11	0.3679
93.51	0.8222	92.71	0.3674

Table S19: Pxy data of the CO_2-Ar binary system obtained from REFPROP[10] using the GERG-2008 EoS[30] at 253 K.

Bubble points		Dew points	
P/[bar]	x_{CO_2}	P/[bar]	y_{CO_2}
94.11	0.8204	93.31	0.3670
94.71	0.8185	93.91	0.3665
95.31	0.8166	94.51	0.3661
95.91	0.8147	95.11	0.3657
96.51	0.8128	95.71	0.3653
97.11	0.8109	96.31	0.3650
97.71	0.8090	96.91	0.3646
98.31	0.8070	97.51	0.3643
98.91	0.8051	98.11	0.3640
99.51	0.8031	98.71	0.3637
100.11	0.8012	99.11	0.3636
100.71	0.7992	99.51	0.3634
101.31	0.7972	99.91	0.3633
101.91	0.7952	100.31	0.3631
102.51	0.7932	100.71	0.3630
103.11	0.7911	101.11	0.3629
103.71	0.7891	101.51	0.3628
104.31	0.7870	101.91	0.3627
104.91	0.7849	102.31	0.3626
105.51	0.7829	102.71	0.3625
106.11	0.7807	103.51	0.3624
106.71	0.7786	103.91	0.3623
107.31	0.7765	108.61	0.3624
107.91	0.7743	109.41	0.3625
108.51	0.7722	110.21	0.3627
109.11	0.7700	110.61	0.3628
109.71	0.7678	111.41	0.3630
110.31	0.7656	112.21	0.3633
110.91	0.7634	112.81	0.3636
111.51	0.7611	113.41	0.3638
112.11	0.7589	114.01	0.3641
112.71	0.7566	114.61	0.3644
113.41	0.7539	115.21	0.3648
114.11	0.7512	115.81	0.3651
114.81	0.7484	116.41	0.3655
115.51	0.7457	117.01	0.3659
116.21	0.7429	117.61	0.3663
116.91	0.7401	118.21	0.3668
117.61	0.7372	118.81	0.3673

Table S19: Pxy data of the CO_2-Ar binary system obtained from REFPROP[10] using the GERG-2008 EoS[30] at 253 K.

Bubble points		Dew points	
P/[bar]	x_{CO_2}	P/[bar]	y_{CO_2}
118.31	0.7343	119.41	0.3678
119.01	0.7314	120.01	0.3683
119.71	0.7285	120.61	0.3689
120.41	0.7255	121.21	0.3694
121.11	0.7225	121.81	0.3701
121.81	0.7195	122.41	0.3707
122.51	0.7164	123.01	0.3714
123.21	0.7133	123.61	0.3721
123.91	0.7101	124.21	0.3728
124.61	0.7069	124.81	0.3736
125.31	0.7037	125.41	0.3744
126.01	0.7004	126.01	0.3752
126.71	0.6971	126.61	0.3761
127.41	0.6937	127.21	0.3770
128.11	0.6903	127.81	0.3779
128.81	0.6868	128.41	0.3789
129.51	0.6833	129.01	0.3799
130.21	0.6797	129.61	0.3810
130.91	0.6760	130.21	0.3821
131.61	0.6723	130.81	0.3832
132.31	0.6686	131.41	0.3844
133.01	0.6647	132.01	0.3857
133.71	0.6608	132.61	0.3870
134.41	0.6568	133.21	0.3883
135.11	0.6528	133.81	0.3897
135.81	0.6486	134.41	0.3911
136.51	0.6444	135.01	0.3927
137.21	0.6400	135.61	0.3942
137.91	0.6356	136.21	0.3959
138.61	0.6310	136.81	0.3976
139.31	0.6263	137.41	0.3994
140.01	0.6215	138.11	0.4016
140.71	0.6165	138.81	0.4039
141.41	0.6114	139.51	0.4063
142.11	0.6060	140.21	0.4089
142.81	0.6005	140.91	0.4116
143.51	0.5947	141.61	0.4145
144.11	0.5895	142.31	0.4176
144.71	0.5841	143.01	0.4209

Table S19: Pxy data of the CO_2-Ar binary system obtained from REFPROP[10] using the GERG-2008 EoS[30] at 253 K.

Bubble points		Dew points	
P/[bar]	x_{CO_2}	P/[bar]	y_{CO_2}
145.31	0.5784	143.71	0.4245
145.81	0.5733	144.41	0.4284
146.31	0.5680	145.11	0.4326
146.81	0.5624	145.81	0.4372
147.31	0.5563	146.51	0.4424
147.71	0.5511	147.11	0.4475
148.11	0.5453	147.71	0.4532
148.51	0.5389	148.21	0.4588
148.81	0.5335	148.61	0.4641
149.06	0.5284	148.99	0.4700
149.31	0.5225	149.25	0.4752
149.48	0.5173	149.48	0.4811
149.61	0.5122	149.61	0.4857
149.67	0.5092	149.67	0.4885
149.70	0.5073	149.70	0.4903

Table S20: Experimental Pxy data of the CO_2-Ar binary system from Coquelet et al., 2008[26] at 253 K.

P/[bar]	x_{CO_2}	y_{CO_2}
25.50	0.9885	0.8235
37.00	0.9650	0.6270
49.05	0.9388	0.5224
61.38	0.9099	0.4584
73.02	0.8805	0.4232
83.06	0.8540	0.4062
91.92	0.8277	0.3985
100.02	0.8014	0.3974
107.07	0.7766	0.4017
112.01	0.7564	0.4061
117.17	0.7335	0.4121
122.12	0.7088	0.4237
124.03	0.6977	0.4280
126.88	0.6799	0.4398
129.87	0.6567	0.4554

S4.3 VLE data of CO_2-H_2 system

Table S21: *Pxy* data of the CO_2-H_2 binary system computed from the *NPT* version of the CFCGE simulations at 250 K.

P/[bar]	x_{CO_2}	y_{CO_2}
25	0.9985	0.781 ± 0.006
35	0.9962 ± 0.0002	0.59 ± 0.01
45	0.9939 ± 0.0002	0.48 ± 0.01
55	0.9917 ± 0.0002	0.408 ± 0.008
65	0.9895 ± 0.0003	0.35 ± 0.01
75	0.9873 ± 0.0002	0.317 ± 0.007
85	0.9851 ± 0.0004	0.280 ± 0.009
95	0.9830 ± 0.0004	0.2579 ± 0.0005
105	0.9805 ± 0.0003	0.239 ± 0.005
115	0.9782 ± 0.0004	0.221 ± 0.004
125	0.9762 ± 0.0004	0.211 ± 0.003
135	0.9741 ± 0.0002	0.196 ± 0.005
145	0.9721 ± 0.0003	0.192 ± 0.003
155	0.9702 ± 0.0003	0.181 ± 0.006
170	0.9666 ± 0.0003	0.164 ± 0.006
200	0.9615 ± 0.0003	0.159 ± 0.006
230	0.9577 ± 0.0003	0.143 ± 0.006
260	0.9494 ± 0.0003	0.130 ± 0.006
290	0.9434 ± 0.0003	0.121 ± 0.006
320	0.9415 ± 0.0003	0.119 ± 0.006

Table S22: *Pxy* data of the CO_2-H_2 binary system obtained from REFPROP[10] using the GERG-2008 EoS[30] at 250 K.

Bubble points		Dew points	
P/[bar]	x_{CO_2}	P/[bar]	y_{CO_2}
17.94	1.0000	17.85	1.0000
18.00	0.9999	17.86	0.9996
18.09	0.9999	17.87	0.9990
18.19	0.9998	17.90	0.9979
18.29	0.9998	17.94	0.9961
18.38	0.9997	18.00	0.9934
18.48	0.9997	18.09	0.9898
18.58	0.9996	18.19	0.9855
18.68	0.9995	18.29	0.9813
18.78	0.9995	18.48	0.9731
18.88	0.9994	18.58	0.9690

Table S22: Pxy data of the CO_2-H_2 binary system obtained from REFPROP[10] using the GERG-2008 EoS[30] at 250 K.

Bubble points		Dew points	
P/[bar]	x_{CO_2}	P/[bar]	y_{CO_2}
18.98	0.9994	18.68	0.9650
19.08	0.9993	18.88	0.9571
19.17	0.9993	18.98	0.9532
19.27	0.9992	19.08	0.9493
19.37	0.9992	19.27	0.9417
19.47	0.9991	19.37	0.9380
19.86	0.9989	19.47	0.9343
20.25	0.9987	19.66	0.9270
20.37	0.9986	19.86	0.9198
20.40	0.9986	20.05	0.9128
20.42	0.9986	20.25	0.9059
20.42	0.9986	20.39	0.9009
20.74	0.9984	20.42	0.8997
21.13	0.9982	20.64	0.8922
21.52	0.9980	20.84	0.8856
21.91	0.9978	21.03	0.8791
22.30	0.9975	21.23	0.8727
22.70	0.9973	21.42	0.8663
23.09	0.9971	21.62	0.8601
23.49	0.9969	21.81	0.8539
23.89	0.9967	22.01	0.8478
24.28	0.9964	22.21	0.8418
24.68	0.9962	22.40	0.8359
25.08	0.9960	22.60	0.8301
25.48	0.9958	22.80	0.8243
25.89	0.9956	22.99	0.8186
26.29	0.9954	23.19	0.8130
26.69	0.9951	23.39	0.8074
27.10	0.9949	23.59	0.8019
27.51	0.9947	23.79	0.7965
27.91	0.9945	23.98	0.7911
28.32	0.9942	24.18	0.7858
28.73	0.9940	24.38	0.7806
29.14	0.9938	24.58	0.7754
29.56	0.9936	24.78	0.7703
29.97	0.9934	24.98	0.7653
30.39	0.9931	25.28	0.7578
30.80	0.9929	25.58	0.7505
31.22	0.9927	25.89	0.7433

Table S22: Pxy data of the CO_2-H_2 binary system obtained from REFPROP[10] using the GERG-2008 EoS[30] at 250 K.

Bubble points		Dew points	
P/[bar]	x_{CO_2}	P/[bar]	y_{CO_2}
31.64	0.9925	26.19	0.7362
32.06	0.9923	26.49	0.7292
32.48	0.9920	26.79	0.7224
32.90	0.9918	27.10	0.7156
33.33	0.9916	27.40	0.7090
33.75	0.9914	27.71	0.7025
34.18	0.9911	28.02	0.6961
34.61	0.9909	28.32	0.6898
35.04	0.9907	28.63	0.6836
35.47	0.9905	28.94	0.6775
35.90	0.9902	29.25	0.6715
36.33	0.9900	29.56	0.6656
36.77	0.9898	29.87	0.6598
37.20	0.9896	30.18	0.6541
37.64	0.9893	30.49	0.6485
38.08	0.9891	30.80	0.6430
38.52	0.9889	31.12	0.6376
38.96	0.9886	31.43	0.6322
39.40	0.9884	31.74	0.6270
39.85	0.9882	32.06	0.6218
40.29	0.9880	32.38	0.6167
40.74	0.9877	32.69	0.6116
41.19	0.9875	33.12	0.6051
41.64	0.9873	33.54	0.5986
42.09	0.9870	33.97	0.5923
42.55	0.9868	34.39	0.5861
43.00	0.9866	34.82	0.5801
43.46	0.9864	35.25	0.5741
43.92	0.9861	35.68	0.5683
44.37	0.9859	36.12	0.5626
44.84	0.9857	36.55	0.5570
45.30	0.9854	36.99	0.5515
45.76	0.9852	37.42	0.5461
44.99	0.9850	37.86	0.5408
45.39	0.9847	38.30	0.5356
45.80	0.9845	38.74	0.5305
46.21	0.9843	39.29	0.5243
46.61	0.9840	39.85	0.5182
47.02	0.9838	40.41	0.5123

Table S22: Pxy data of the CO_2-H_2 binary system obtained from REFPROP[10] using the GERG-2008 EoS[30] at 250 K.

Bubble points		Dew points	
P/[bar]	x_{CO_2}	P/[bar]	y_{CO_2}
47.42	0.9835	40.97	0.5065
47.83	0.9833	41.53	0.5008
48.24	0.9831	42.09	0.4953
48.64	0.9828	42.66	0.4899
49.05	0.9826	43.23	0.4846
49.46	0.9824	43.80	0.4794
49.87	0.9821	44.37	0.4743
50.27	0.9819	45.07	0.4683
50.68	0.9816	45.76	0.4625
51.09	0.9814	44.99	0.4697
51.50	0.9812	45.60	0.4646
51.90	0.9809	46.21	0.4597
52.31	0.9807	46.81	0.4549
52.72	0.9804	47.42	0.4502
53.13	0.9802	48.03	0.4456
53.54	0.9800	48.64	0.4412
53.94	0.9797	49.26	0.4368
54.35	0.9795	49.87	0.4326
54.76	0.9792	50.48	0.4285
55.17	0.9790	51.09	0.4244
55.58	0.9787	51.70	0.4205
55.99	0.9785	52.31	0.4166
56.40	0.9782	52.92	0.4129
56.80	0.9780	53.54	0.4092
57.21	0.9777	54.15	0.4056
57.62	0.9775	54.76	0.4021
58.03	0.9772	55.37	0.3987
58.44	0.9770	55.99	0.3953
58.85	0.9767	56.60	0.3920
59.26	0.9765	57.21	0.3888
59.67	0.9762	57.83	0.3857
60.08	0.9760	58.44	0.3826
60.49	0.9757	59.06	0.3796
60.90	0.9755	59.67	0.3766
61.31	0.9752	60.29	0.3737
61.72	0.9750	60.90	0.3709
62.13	0.9747	61.52	0.3681
62.54	0.9745	62.13	0.3654
62.96	0.9742	62.75	0.3627

Table S22: Pxy data of the CO_2-H_2 binary system obtained from REFPROP[10] using the GERG-2008 EoS[30] at 250 K.

Bubble points		Dew points	
P/[bar]	x_{CO_2}	P/[bar]	y_{CO_2}
63.37	0.9739	63.37	0.3601
63.78	0.9737	63.98	0.3576
64.19	0.9734	64.60	0.3551
64.60	0.9732	65.22	0.3526
65.01	0.9729	65.84	0.3502
65.42	0.9726	66.45	0.3478
65.84	0.9724	67.07	0.3455
66.25	0.9721	67.69	0.3432
66.66	0.9719	68.31	0.3409
67.07	0.9716	68.93	0.3387
67.48	0.9713	69.55	0.3366
68.10	0.9709	70.17	0.3345
68.72	0.9705	70.79	0.3324
69.34	0.9701	71.41	0.3303
69.96	0.9697	72.03	0.3283
70.58	0.9693	72.65	0.3263
71.20	0.9689	73.28	0.3244
71.82	0.9685	73.90	0.3225
72.45	0.9681	74.52	0.3206
73.07	0.9677	75.14	0.3188
73.69	0.9672	75.77	0.3169
74.31	0.9668	76.39	0.3152
74.94	0.9664	77.01	0.3134
75.56	0.9660	77.64	0.3117
76.18	0.9656	78.26	0.3100
76.81	0.9651	78.89	0.3083
77.43	0.9647	79.51	0.3066
78.06	0.9643	80.14	0.3050
78.68	0.9638	80.77	0.3034
79.31	0.9634	81.40	0.3019
79.93	0.9630	82.02	0.3003
80.56	0.9625	82.65	0.2988
81.19	0.9621	83.28	0.2973
81.81	0.9616	83.91	0.2958
82.44	0.9612	84.54	0.2944
83.07	0.9607	85.17	0.2929
83.70	0.9603	85.80	0.2915
84.33	0.9598	86.43	0.2901
84.96	0.9593	87.06	0.2888

Table S22: Pxy data of the CO_2-H_2 binary system obtained from REFPROP[10] using the GERG-2008 EoS[30] at 250 K.

Bubble points		Dew points	
P/[bar]	x_{CO_2}	P/[bar]	y_{CO_2}
85.59	0.9589	87.69	0.2874
86.22	0.9584	88.33	0.2861
86.85	0.9579	88.96	0.2848
87.48	0.9575	89.59	0.2835
88.11	0.9570	90.23	0.2822
88.75	0.9565	90.86	0.2809
89.38	0.9560	91.50	0.2797
90.02	0.9555	92.13	0.2785
90.65	0.9550	92.77	0.2773
91.29	0.9545	93.41	0.2761
91.92	0.9540	94.05	0.2749
92.56	0.9535	94.69	0.2738
93.20	0.9530	95.33	0.2726
93.83	0.9525	95.97	0.2715
94.47	0.9520	96.61	0.2704
95.11	0.9515	97.25	0.2693
95.75	0.9510	97.89	0.2682
96.39	0.9504	98.53	0.2671
97.03	0.9499	99.18	0.2661
97.68	0.9493	99.82	0.2650
98.32	0.9488	100.47	0.2640
98.96	0.9482	101.12	0.2630
99.61	0.9477	101.76	0.2620
100.25	0.9471	102.41	0.2610
100.90	0.9466	103.06	0.2600
101.55	0.9460	103.71	0.2591
102.20	0.9454	104.37	0.2581
102.85	0.9448	105.02	0.2572
103.50	0.9442	105.67	0.2562
104.15	0.9436	106.33	0.2553
104.80	0.9430	106.98	0.2544
105.45	0.9424	107.64	0.2535
106.11	0.9418	108.30	0.2526
106.77	0.9411	108.96	0.2517
107.42	0.9405	109.62	0.2509
108.08	0.9399	110.29	0.2500
108.74	0.9392	110.95	0.2492
109.40	0.9385	111.62	0.2483
110.07	0.9379	112.29	0.2475

Table S22: Pxy data of the CO_2-H_2 binary system obtained from REFPROP[10] using the GERG-2008 EoS[30] at 250 K.

Bubble points		Dew points	
P/[bar]	x_{CO_2}	P/[bar]	y_{CO_2}
110.73	0.9372	112.96	0.2467
111.40	0.9365	113.63	0.2459
112.06	0.9358	114.30	0.2451
112.73	0.9350	114.98	0.2443
113.40	0.9343	115.65	0.2435
114.08	0.9336	116.33	0.2427
114.75	0.9328	117.02	0.2420
115.43	0.9320	117.70	0.2412
116.11	0.9312	118.39	0.2404
116.79	0.9304	119.08	0.2397
117.47	0.9296	119.77	0.2390
118.16	0.9288	120.47	0.2382
118.85	0.9279	121.17	0.2375
119.54	0.9270	121.87	0.2368
120.24	0.9261	122.58	0.2361
120.94	0.9252	123.30	0.2354
121.64	0.9243	124.01	0.2347
122.35	0.9233	124.74	0.2340
123.06	0.9223	125.46	0.2333
123.77	0.9212	126.20	0.2327
124.49	0.9201	126.94	0.2320
125.22	0.9190	127.69	0.2313
125.95	0.9179	128.45	0.2307
126.69	0.9166	129.22	0.2300
127.44	0.9154	130.01	0.2294
128.20	0.9140	130.80	0.2287
128.96	0.9126	131.62	0.2281
129.74	0.9112	132.46	0.2275
130.54	0.9096	133.33	0.2269
131.35	0.9079	134.24	0.2262
132.18	0.9060	135.22	0.2256
133.04	0.9039	135.76	0.2253
133.93	0.9016	135.77	0.2253
134.88	0.8989	135.77	0.2253
135.75	0.8961	135.77	0.2253
135.77	0.8961	135.83	0.2252
135.77	0.8961	136.43	0.2246
135.77	0.8961	137.03	0.2240
136.03	0.8958	137.63	0.2234

Table S22: Pxy data of the CO_2-H_2 binary system obtained from REFPROP[10] using the GERG-2008 EoS[30] at 250 K.

Bubble points		Dew points	
P/[bar]	x_{CO_2}	P/[bar]	y_{CO_2}
136.63	0.8951	138.23	0.2228
137.23	0.8944	138.83	0.2222
137.83	0.8937	139.43	0.2216
138.43	0.8930	140.03	0.2211
139.03	0.8923	140.63	0.2205
139.63	0.8916	141.23	0.2199
140.23	0.8909	141.83	0.2194
140.83	0.8902	142.43	0.2188
141.43	0.8895	143.03	0.2182
142.03	0.8888	143.63	0.2177
142.63	0.8880	144.23	0.2172
143.23	0.8873	144.83	0.2166
143.83	0.8866	145.43	0.2161
144.43	0.8858	146.03	0.2156
145.03	0.8851	146.63	0.2150
145.63	0.8843	147.23	0.2145
146.23	0.8836	147.83	0.2140
146.83	0.8828	148.43	0.2135
147.43	0.8821	149.03	0.2130
148.03	0.8813	149.63	0.2125
148.63	0.8805	150.23	0.2120
149.23	0.8797	150.83	0.2115
149.83	0.8789	151.43	0.2110
150.43	0.8782	152.03	0.2105
151.03	0.8774	152.63	0.2101
151.63	0.8765	153.23	0.2096
152.23	0.8757	153.83	0.2091
152.83	0.8749	154.43	0.2086
153.43	0.8741	155.03	0.2082
154.03	0.8733	155.63	0.2077
154.63	0.8724	156.23	0.2073
155.23	0.8716	156.83	0.2068
155.83	0.8707	157.43	0.2064
156.43	0.8698	158.03	0.2059
157.03	0.8690	158.63	0.2055
157.63	0.8681	159.23	0.2050
158.23	0.8672	159.83	0.2046
158.83	0.8663	160.43	0.2042
159.43	0.8653	161.03	0.2037

Table S22: Pxy data of the CO_2-H_2 binary system obtained from REFPROP[10] using the GERG-2008 EoS[30] at 250 K.

Bubble points		Dew points	
P/[bar]	x_{CO_2}	P/[bar]	y_{CO_2}
160.03	0.8644	161.63	0.2033
160.63	0.8635	162.23	0.2029
161.23	0.8625	162.83	0.2025
161.83	0.8615	163.43	0.2021
162.43	0.8606	164.03	0.2016
163.03	0.8596	164.63	0.2012
163.63	0.8586	165.23	0.2008
164.23	0.8575	165.83	0.2004
164.83	0.8565	166.43	0.2000
165.43	0.8554	167.03	0.1996
166.03	0.8544	167.63	0.1992
166.63	0.8533	168.23	0.1988
167.23	0.8521	168.83	0.1985
167.83	0.8510	169.43	0.1981
168.43	0.8498	170.03	0.1977
169.03	0.8486	170.63	0.1973
169.63	0.8474	171.23	0.1969
170.23	0.8462	171.83	0.1965
170.83	0.8449	172.43	0.1962
171.43	0.8436	173.03	0.1958
172.03	0.8422	173.63	0.1954
172.63	0.8408	174.23	0.1951
173.23	0.8393	174.83	0.1947
173.93	0.8376	175.43	0.1943
174.63	0.8357	176.03	0.1940
175.33	0.8338	176.63	0.1936
176.03	0.8317	177.23	0.1932
176.73	0.8295	177.83	0.1929
177.43	0.8271	178.43	0.1925
178.13	0.8245	179.03	0.1922
178.83	0.8215	179.63	0.1918
179.53	0.8181	180.23	0.1915
180.23	0.8138	180.75	0.1912
180.81	0.8086	180.99	0.1910
181.04	0.8052	181.09	0.1910
181.11	0.8031	181.13	0.1909

Table S23: Experimental Pxy data of the CO_2-H_2 binary system from Tsang and Street., 1981[27] at 250 K.

P/[bar]	x_{CO_2}	y_{CO_2}
2.31	0.9956	0.8411
3.04	0.9914	0.6804
3.63	0.9876	0.5910
5.20	0.9814	0.4499
6.90	0.9738	0.3629
10.29	0.9604	0.2821
13.85	0.9455	0.2415
17.28	0.9305	0.2178
25.44	0.8942	0.1956
31.54	0.8668	0.1895

S4.4 VLE data of CO_2-CH_4 system

Table S24: Pxy data of the CO_2-CH_4 binary system computed from the NPT version of the CFCGE simulations at 250 K.

P/[bar]	x_{CO_2}	y_{CO_2}
35	0.914 ± 0.002	0.57 ± 0.01
40	0.881 ± 0.005	0.503 ± 0.008
45	0.853 ± 0.006	0.466 ± 0.009
50	0.818 ± 0.008	0.431 ± 0.003
55	0.77 ± 0.01	0.40 ± 0.01

Table S25: Pxy data of the CO_2-CH_4 binary system obtained from REFPROP[10] using the GERG-2008 EoS[30] at 250 K.

Bubble points		Dew points	
P/[bar]	x_{CO_2}	P/[bar]	y_{CO_2}
17.86	1.0000	17.85	1.0000
17.87	0.9999	17.86	0.9997
17.89	0.9999	17.87	0.9992
17.92	0.9997	17.89	0.9982
17.97	0.9996	17.92	0.9967
18.04	0.9993	17.97	0.9944
18.13	0.9990	18.04	0.9912
18.23	0.9986	18.13	0.9869
18.33	0.9982	18.23	0.9824
18.43	0.9979	18.33	0.9779
18.53	0.9975	18.43	0.9734
18.63	0.9971	18.53	0.9691
18.73	0.9968	18.63	0.9647
18.83	0.9964	18.73	0.9604
18.93	0.9960	18.83	0.9562
19.03	0.9956	18.93	0.9520
19.13	0.9953	19.03	0.9478
19.23	0.9949	19.13	0.9437
19.33	0.9945	19.23	0.9397
19.43	0.9942	19.33	0.9356
20.03	0.9919	19.43	0.9316
20.63	0.9897	19.63	0.9238
21.23	0.9874	19.83	0.9161
21.83	0.9851	20.03	0.9085
22.43	0.9828	20.23	0.9011
23.03	0.9805	20.43	0.8939
23.63	0.9781	20.63	0.8868
24.23	0.9758	20.83	0.8798
24.83	0.9734	21.03	0.8729
25.43	0.9710	21.23	0.8662
26.03	0.9686	21.43	0.8596
26.63	0.9662	21.63	0.8531
27.23	0.9637	21.83	0.8467
27.83	0.9613	22.03	0.8405
28.43	0.9588	22.23	0.8343
29.03	0.9563	22.43	0.8283
29.63	0.9538	22.63	0.8224
30.23	0.9512	22.83	0.8165
30.83	0.9487	23.03	0.8108

Table S25: Pxy data of the CO_2-CH_4 binary system obtained from REFPROP[10] using the GERG-2008 EoS[30] at 250 K.

Bubble points		Dew points	
P/[bar]	x_{CO_2}	P/[bar]	y_{CO_2}
31.43	0.9461	23.23	0.8052
32.03	0.9435	23.43	0.7996
32.63	0.9409	23.63	0.7942
33.23	0.9383	23.83	0.7888
33.83	0.9356	24.03	0.7835
34.43	0.9330	24.23	0.7784
35.03	0.9303	24.43	0.7733
35.63	0.9275	24.63	0.7682
36.23	0.9248	24.93	0.7608
36.83	0.9220	25.23	0.7536
37.43	0.9193	25.53	0.7466
38.03	0.9165	25.83	0.7397
38.63	0.9136	26.13	0.7330
39.23	0.9108	26.43	0.7264
39.83	0.9079	26.73	0.7200
40.43	0.9050	27.03	0.7137
41.03	0.9021	27.33	0.7076
41.63	0.8991	27.63	0.7015
42.23	0.8961	27.93	0.6957
42.83	0.8931	28.23	0.6899
43.43	0.8901	28.53	0.6843
44.03	0.8870	28.83	0.6787
44.63	0.8839	29.13	0.6733
45.23	0.8808	29.43	0.6680
45.83	0.8777	29.73	0.6628
46.43	0.8745	30.03	0.6578
47.03	0.8713	30.43	0.6511
47.63	0.8680	30.83	0.6447
48.23	0.8647	31.23	0.6384
48.83	0.8614	31.63	0.6323
49.43	0.8581	32.03	0.6264
50.03	0.8547	32.43	0.6206
50.63	0.8513	32.83	0.6149
51.23	0.8479	33.23	0.6094
51.83	0.8444	33.63	0.6040
52.43	0.8408	34.03	0.5988
53.03	0.8373	34.43	0.5936
53.63	0.8337	34.93	0.5874
54.23	0.8300	35.43	0.5814

Table S25: Pxy data of the CO_2-CH_4 binary system obtained from REFPROP[10] using the GERG-2008 EoS[30] at 250 K.

Bubble points		Dew points	
P/[bar]	x_{CO_2}	P/[bar]	y_{CO_2}
54.83	0.8264	35.93	0.5755
55.43	0.8226	36.43	0.5698
56.03	0.8189	36.93	0.5643
56.63	0.8150	37.43	0.5589
57.23	0.8112	37.93	0.5537
57.83	0.8073	38.43	0.5487
58.43	0.8033	39.03	0.5428
59.03	0.7993	39.63	0.5371
59.63	0.7953	40.23	0.5316
60.23	0.7912	41.43	0.5211
60.83	0.7870	42.03	0.5161
61.53	0.7821	43.23	0.5066
62.23	0.7771	44.43	0.4977
62.93	0.7720	45.03	0.4935
63.63	0.7668	46.23	0.4854
64.33	0.7616	47.43	0.4778
65.03	0.7563	48.03	0.4742
65.73	0.7508	49.23	0.4673
66.43	0.7453	50.43	0.4609
67.13	0.7397	51.03	0.4578
67.83	0.7340	52.23	0.4520
68.53	0.7281	53.43	0.4465
69.13	0.7230	54.03	0.4439
69.73	0.7179	55.23	0.4390
70.33	0.7126	56.43	0.4344
70.93	0.7072	57.03	0.4322
71.53	0.7018	58.23	0.4281
72.13	0.6962	59.43	0.4243
72.73	0.6906	60.03	0.4225
73.33	0.6848	61.23	0.4191
73.93	0.6789	62.43	0.4161
74.43	0.6739	63.03	0.4146
74.93	0.6688	64.23	0.4120
75.43	0.6636	65.43	0.4097
75.93	0.6583	66.03	0.4086
76.43	0.6529	67.23	0.4067
76.93	0.6474	68.43	0.4051
77.43	0.6417	69.03	0.4045
77.93	0.6359	70.23	0.4034

Table S25: Pxy data of the CO_2-CH_4 binary system obtained from REFPROP[10] using the GERG-2008 EoS[30] at 250 K.

Bubble points		Dew points	
P/[bar]	x_{CO_2}	P/[bar]	y_{CO_2}
78.43	0.6300	71.23	0.4027
78.93	0.6240	72.03	0.4023
79.43	0.6177	72.43	0.4022
79.83	0.6126	72.83	0.4021
80.23	0.6074	73.83	0.4022
80.63	0.6020	74.63	0.4024
81.03	0.5965	75.03	0.4026
81.43	0.5908	75.63	0.4029
81.83	0.5850	76.23	0.4035
82.23	0.5789	76.83	0.4041
82.63	0.5726	77.43	0.4049
83.03	0.5660	78.03	0.4059
83.33	0.5608	79.23	0.4084
83.63	0.5554	79.83	0.4100
83.93	0.5497	80.43	0.4119
84.20	0.5443	81.03	0.4141
84.48	0.5382	81.63	0.4166
84.78	0.5311	82.33	0.4202
84.98	0.5260	83.03	0.4246
85.22	0.5190	83.73	0.4302
85.41	0.5129	84.24	0.4354
85.55	0.5076	84.68	0.4408
85.68	0.5018	85.07	0.4468
85.78	0.4967	85.35	0.4525
85.84	0.4918	85.59	0.4586
85.87	0.4894	85.74	0.4642
85.88	0.4884	85.83	0.4687
85.88	0.4881	85.87	0.4713
85.88	0.4881	85.88	0.4725

Table S26: Experimental Pxy data of the CO_2-CH_4 binary system from Wei et al., 1995[29] at 250 K.

P/[bar]	x_{CO_2}	y_{CO_2}
17.83	0.0000	0.0000
19.67	0.0065	0.0782
20.71	0.0106	0.1174
23.15	0.0201	0.1952
24.88	0.0269	0.2408
27.59	0.0379	0.3001
29.64	0.0465	0.3381
31.31	0.0536	0.3655
34.46	0.0676	0.4090
38.36	0.0863	0.4533
42.00	0.1041	0.4855
47.92	0.1368	0.5262
53.78	0.1715	0.5559
61.50	0.2246	0.5812
67.15	0.2711	0.5914
73.99	0.3366	0.5856
76.84	0.3718	0.5761
79.83	0.4163	0.5523
80.53	0.4252	0.5408

Table S27: Experimental Pxy data of the CO_2-CH_4 binary system from Davalos et al., 1976[28] at 250 K.

P/[bar]	x_{CO_2}	y_{CO_2}
17.62	0.0000	0.0000
20.00	0.1040	0.0100
30.00	0.3610	0.0530
40.00	0.4910	0.1050
50.00	0.5750	0.1660
60.00	0.6050	0.2370
70.00	0.6150	0.3260
77.00	0.6050	0.4000
78.50	0.5640	0.4050
79.88	0.5580	0.4460

S5 Derivation of the heat capacity at constant volume (C_V)

This section presents the derivation of an equation to compute the heat capacity at constant volume (C_V) from molelcular simulation. To calculate C_V from molecular simulations, one needs to define C_V in terms of the internal energy U of the system with respect to temperature T.

$$C_V(T,V) = \left(\frac{\partial \langle U \rangle}{\partial \beta}\right)_V \times \left(\frac{\partial \beta}{\partial T}\right)_V \tag{S3}$$

$$U = U^{\text{internal}} + U^{\text{external}} + K \tag{S4}$$

Where $\beta = 1/(k_{\text{B}}T)$, k_{B} is the Boltzmann constant. The internal energy U of the system is split into U^{internal}, U^{external}, and K, which are the contributions from intramolecular interactions, intermolecular interactions, and the contribution from the kinetic energy, respectively. To calculate the heat capacity in MC simulations, the heat capacity has to be split into two parts.[31–33] One part is the ideal part, which includes the contribution from the kinetic energy, and the other is the residual part, which includes contributions from intermolecular interactions, as shown below:

$$C_V(T,V) = C_V^{\text{ideal}}(T) + C_V^{\text{residual}}(T,V) = \left(\frac{\partial \langle U^{\text{ideal}} \rangle}{\partial T}\right)_V + \left(\frac{\partial \langle U^{\text{residual}} \rangle}{\partial T}\right)_V \tag{S5}$$

Where $C_V^{\text{ideal}}(T)$ is defined as,

$$C_V^{\text{ideal}}(T) = \frac{\partial}{\partial T}\langle U^{\text{ideal}} \rangle = \frac{\partial}{\partial T}\langle U^{\text{internal}} + K \rangle = C_V^{\text{QM}}(T) \tag{S6}$$

$C_V^{\mathrm{ideal}}(T)$ of the molecules should be calculated separately from the tabulated thermodynamic reference tables[34,35] or from the quantum mechanical calculation, which computes $C_V^{\mathrm{ideal}}(T)$ from the translation, rotation, vibration, and electronic energy levels of a single isolated molecule. We calculated the $C_V^{\mathrm{ideal}}(T)$ from the Gaussian09 software[36] with the B3LYP/6-31G(d,p) basis set, where 6-31G(d,p) represents the basis set and B3LYP represents the hybrid density functional method used to calculate the electronic structure of the molecule. $C_V^{\mathrm{residual}}(T,V)$ can be calculated from MC simulations,

$$C_V^{\mathrm{residual}}(T,V) = \left(\frac{\partial \langle U^{\mathrm{residual}} \rangle}{\partial T}\right)_V \equiv \left(\frac{\partial \langle U^{\mathrm{external}} \rangle}{\partial T}\right)_V \tag{S7}$$

$$\frac{\partial}{\partial T}\langle U^{\mathrm{external}} \rangle = -\frac{1}{k_{\mathrm{B}}T^2} \times \frac{\partial}{\partial \beta}\langle U^{\mathrm{external}} \rangle \tag{S8}$$

To find the derivative of $\langle U^{\mathrm{external}} \rangle$ with respect to β, we start form the partition function of a mixture of S components in the NVT ensemble,

$$Q_{NVT} = \prod_{i=1}^{S} \frac{V^N}{\Lambda_i^{3N_i} N_i!} \int \mathrm{d}s^N \exp\left[-\beta \hat{U}\left(s^N\right)\right] \tag{S9}$$

where Λ_i is the thermal wavelength of component i, N_i is the number of molecules of the component i, and s^N are the scaled coordinates. N is the total number of molecules in the system, and $\hat{U}$ is the configuration energy of the system, which includes both intramolecular and intermolecular interactions,

$$\hat{U} = U^{\mathrm{internal}} + U^{\mathrm{external}} \tag{S10}$$

To calculate the $C_V^{\text{residual}}(T,V)$ of the molecules one requires the ensemble average of the U^{external} in the NVT ensemble,

$$\langle U^{\text{external}}\rangle = \frac{\prod_{i=1}^{S} \frac{V_i^N}{\Lambda^{3N_i} N_i!} \int \mathrm{d}s^N (U^{\text{external}}) \exp\left[-\beta \hat{U}\left(s^N\right)\right]}{Q_{NVT}} \tag{S11}$$

$$= \frac{\int \mathrm{d}s^N (U^{\text{external}}) \exp\left[-\beta \hat{U}\left(s^N\right)\right]}{\int \mathrm{d}s^N \exp\left[-\beta \hat{U}\left(s^N\right)\right]} \tag{S12}$$

The derivative of $\langle U^{\text{external}}\rangle$ with respect to β can be written as[37]

$$\frac{\partial \langle U^{\text{external}}\rangle}{\partial \beta} = \frac{\partial}{\partial \beta}\left(\frac{\int \mathrm{d}s^N (U^{\text{external}}) \exp\left[-\beta \hat{U}\left(s^N\right)\right]}{\int \mathrm{d}s^N \exp\left[-\beta \hat{U}\left(s^N\right)\right]}\right) \tag{S13}$$

$$\begin{aligned} &= \left[\frac{\frac{\partial}{\partial \beta}\left(\int \mathrm{d}s^N (U^{\text{external}}) \exp\left[-\beta \hat{U}\left(s^N\right)\right]\right) \times \int \mathrm{d}s^N \exp\left[-\beta \hat{U}\left(s^N\right)\right]}{\left[\int \mathrm{d}s^N \exp\left[-\beta \hat{U}\left(s^N\right)\right]\right]^2}\right] \\ &(-)\left[\frac{\frac{\partial}{\partial \beta}\left(\int \mathrm{d}s^N \exp\left[-\beta \hat{U}\left(s^N\right)\right]\right)}{\int \mathrm{d}s^N \exp\left[-\beta \hat{U}\left(s^N\right)\right]} \times \frac{\int \mathrm{d}s^N (U^{\text{external}}) \exp\left[-\beta \hat{U}\left(s^N\right)\right]}{\int \mathrm{d}s^N \exp\left[-\beta \hat{U}\left(s^N\right)\right]}\right] \end{aligned} \tag{S14}$$

$$\begin{aligned} &= -\left[\frac{\int \mathrm{d}s^N (U^{\text{external}} \hat{U}) \exp\left[-\beta \hat{U}\left(s^N\right)\right]}{\int \mathrm{d}s^N \exp\left[-\beta \hat{U}\left(s^N\right)\right]}\right] \\ &+ \left[\frac{\int \mathrm{d}s^N (\hat{U}) \exp\left[-\beta \hat{U}\left(s^N\right)\right]}{\int \mathrm{d}s^N \exp\left[-\beta \hat{U}\left(s^N\right)\right]} \times \langle U^{\text{external}}\rangle\right] \end{aligned} \tag{S15}$$

$$= \frac{\int \mathrm{d}s^N (U^{\text{external}} \hat{U}) \exp\left[-\beta \hat{U}\left(s^N\right)\right]}{\int \mathrm{d}s^N \exp\left[-\beta \hat{U}\left(s^N\right)\right]} - \langle \hat{U}\rangle\langle U^{\text{external}}\rangle \tag{S16}$$

$$= \langle \hat{U}\rangle\langle U^{\text{external}}\rangle - \langle U^{\text{external}} \hat{U}\rangle \tag{S17}$$

Substituting Eq. S17 in Eq. S8 leads to

$$C_V^{\text{residual}}(T,V) = (-)\frac{1}{k_\text{B}T^2}\left(\langle U^{\text{external}}\rangle\langle\hat{U}\rangle - \langle U^{\text{external}}\hat{U}\rangle\right) \tag{S18}$$

$$C_V^{\text{residual}}(T,V) = \frac{1}{k_\text{B}T^2}\left(\langle U^{\text{external}}\hat{U}\rangle - \langle U^{\text{external}}\rangle\langle\hat{U}\rangle\right) \tag{S19}$$

The same expression for C_V^{residual} is also reported by Refs.[32,33]

S6 Derivation of the heat capacity at constant pressure (C_P)

This section shows the derivation of an equation to compute the heat capacity at constant pressure (C_P). To calculate C_P from molecular simulation, one needs to define C_P in terms of the enthalpy (H) of the system with respect to temperature.

$$C_P(T,P) = \left(\frac{\partial\langle H\rangle}{\partial T}\right)_P \tag{S20}$$

The enthalpy H in the above equation is defined as,

$$H = U^{\text{internal}} + U^{\text{external}} + K + PV \tag{S21}$$

Similar to the split performed for C_{V} in Section S3, the heat capacity at constant pressure (C_P) is also split into two parts:[31–33]

$$C_P(T,P) = C_P^{\text{ideal}}(T) + C_P^{\text{residual}}(T,P) \tag{S22}$$

$$= \left(\frac{\partial\langle H^{\text{ideal}}\rangle}{\partial T}\right)_P + \left(\frac{\partial\langle H^{\text{residual}}\rangle}{\partial T}\right)_P \tag{S23}$$

$$H^{\text{ideal}}(T) = U^{\text{internal}} + K + Nk_{\text{B}}T \tag{S24}$$

$$H^{\text{residual}}(T,P) = U^{\text{external}} + PV - Nk_{\text{B}}T \tag{S25}$$

$$C_P^{\text{ideal}}(T) = \left(\frac{\partial\langle H^{\text{ideal}}\rangle}{\partial T}\right)_P \tag{S26}$$

$$= \left(\frac{\partial\langle U^{\text{internal}} + K\rangle}{\partial T}\right)_P + Nk_{\text{B}} \tag{S27}$$

where N is the total number of molecules. The first term on the right side of Eq. S27 is equal to Eq. S6 in Section S3. So, Eq. S27 is equivalent to

$$C_P^{\text{ideal}}(T) = C_{\text{V}}^{\text{ideal}}(T) + R \tag{S28}$$

where R is the universal gas constant. The expression to calculate $C_P^{\text{residual}}(T, P)$ is,

$$C_P^{\text{residual}}(T, P) = \left(\frac{\partial \langle H^{\text{residual}} \rangle}{\partial T}\right)_P \tag{S29}$$

$$= \left(\frac{\partial \langle U^{\text{external}} \rangle}{\partial T}\right)_P + P\left(\frac{\partial \langle V \rangle}{\partial T}\right)_P - Nk_{\text{B}} \tag{S30}$$

$$= (-)\frac{1}{k_{\text{B}}T^2}\left[\left(\frac{\partial \langle U^{\text{external}} \rangle}{\partial \beta}\right)_P + P\left(\frac{\partial \langle V \rangle}{\partial \beta}\right)_P\right] - Nk_{\text{B}} \tag{S31}$$

To obtain the partial derivatives of $\langle U^{\text{external}} \rangle$ and V with respect to β, we start from the partition function in NPT ensemble.

$$Q_{N,P,T} = \beta P \prod_{i=1}^{S} \frac{1}{\Lambda_i^{3N_i} N_i!} \int \mathrm{d}V\, V^N \int \mathrm{d}s^N \exp\left[-\beta \hat{H}\left(s^N, V\right)\right] \tag{S32}$$

where $\hat{H}$ is the configuration enthalpy of the system, defined as $\hat{H} = U^{\text{internal}} + U^{\text{external}} + PV$.

$$\langle U^{\text{external}} \rangle = \frac{\beta P \prod_{i=1}^{S} \frac{V_i^N}{\Lambda^{3N_i} N_i!} \int \mathrm{d}V\, V^N (U^{\text{external}}) \int \mathrm{d}s^N \exp\left[-\beta \hat{H}\left(s^N, V\right)\right]}{\beta P \prod_{i=1}^{S} \frac{1}{\Lambda_i^{3N_i} N_i!} \int \mathrm{d}V\, V^N \int \mathrm{d}s^N \exp\left[-\beta \hat{H}\left(s^N, V\right)\right]} \tag{S33}$$

$$= \frac{\int \mathrm{d}V\, V^N (U^{\text{external}}) \int \mathrm{d}s^N \exp\left[-\beta \hat{H}\left(s^N, V\right)\right]}{\int \mathrm{d}V\, V^N \int \mathrm{d}s^N \exp\left[-\beta \hat{H}\left(s^N, V\right)\right]} \tag{S34}$$

$$\frac{\partial \langle U^{\text{external}} \rangle}{\partial \beta} = \frac{\partial}{\partial \beta}\left(\frac{\int \mathrm{d}V\, V^N (U^{\text{external}}) \int \mathrm{d}s^N \exp\left[-\beta \hat{H}\left(s^N, V\right)\right]}{\int \mathrm{d}V\, V^N \int \mathrm{d}s^N \exp\left[-\beta \hat{H}\left(s^N, V\right)\right]}\right) \tag{S35}$$

Then, the quotient rule is applied to the derivative in Eq. S35

$$\frac{\partial\langle U^{\text{external}}\rangle}{\partial\beta} = \left[\frac{\frac{\partial}{\partial\beta}\left(\int \mathrm{d}V\, V^N (U^{\text{external}}) \int \mathrm{d}s^N \exp\left[-\beta\hat{H}\left(s^N, V\right)\right]\right)_P}{\left[\int \mathrm{d}V\, V^N \int \mathrm{d}s^N \exp\left[-\beta\hat{H}\left(s^N, V\right)\right]\right]^2} \times \left(\int \mathrm{d}V\, V^N \int \mathrm{d}s^N \exp\left[-\beta\hat{H}\left(s^N, V\right)\right]\right)\right] - \left[\frac{\frac{\partial}{\partial\beta} \times \left(\int \mathrm{d}V\, V^N \int \mathrm{d}s^N \exp\left[-\beta\hat{H}\left(s^N, V\right)\right]\right)}{\int \mathrm{d}V\, V^N \int \mathrm{d}s^N \exp\left[-\beta\hat{H}\left(s^N, V\right)\right]} \times \frac{\int \mathrm{d}V\, V^N (U^{\text{external}}) \int \mathrm{d}s^N \exp\left[-\beta\hat{H}\left(s^N, V\right)\right]}{\int \mathrm{d}V\, V^N \int \mathrm{d}s^N \exp\left[-\beta\hat{H}\left(s^N, V\right)\right]}\right] \tag{S36}$$

$$= \left[\frac{\frac{\partial}{\partial\beta}\left(\int \mathrm{d}V\, V^N (U^{\text{external}}) \int \mathrm{d}s^N \exp\left[-\beta\hat{H}\left(s^N, V\right)\right]\right)_P}{\left[\int \mathrm{d}V\, V^N \int \mathrm{d}s^N \exp\left[-\beta\hat{H}\left(s^N, V\right)\right]\right]}\right] - \left[\frac{\frac{\partial}{\partial\beta} \times \left(\int \mathrm{d}V\, V^N \int \mathrm{d}s^N \exp\left[-\beta\hat{H}\left(s^N, V\right)\right]\right)}{\int \mathrm{d}V\, V^N \int \mathrm{d}s^N \exp\left[-\beta\hat{H}\left(s^N, V\right)\right]} \times \frac{\int \mathrm{d}V\, V^N (U^{\text{external}}) \int \mathrm{d}s^N \exp\left[-\beta\hat{H}\left(s^N, V\right)\right]}{\int \mathrm{d}V\, V^N \int \mathrm{d}s^N \exp\left[-\beta\hat{H}\left(s^N, V\right)\right]}\right] \tag{S37}$$

$$= \left[(-)\frac{\int \mathrm{d}V\, V^N (U^{\text{external}} \times \hat{H}) \int \mathrm{d}s^N \exp\left[-\beta\hat{H}\left(s^N, V\right)\right]}{\int \mathrm{d}V\, V^N \int \mathrm{d}s^N \exp\left[-\beta\hat{H}\left(s^N, V\right)\right]}\right] - \left[(-)\frac{\int \mathrm{d}V\, V^N (\hat{H}) \int \mathrm{d}s^N \exp\left[-\beta\hat{H}\left(s^N, V\right)\right]}{\int \mathrm{d}V\, V^N \int \mathrm{d}s^N \exp\left[-\beta\hat{H}\left(s^N, V\right)\right]} \times \langle U^{\text{external}}\rangle\right] \tag{S38}$$

$$= -\langle U^{\text{external}}\hat{H}\rangle + \langle\hat{H}\rangle \times \langle U^{\text{external}}\rangle \tag{S39}$$

Next, we need the partial derivative of $\langle V\rangle$ with respect to β to obtain $C_P^{\text{residual}}(T,P)$ in Eq. S31

$$\langle V\rangle = \frac{\int \mathrm{d}V\, V^{N+1} \int \mathrm{d}s^N \exp\left[-\beta \hat{H}\left(s^N, V\right)\right]}{\int \mathrm{d}V\, V^{N} \int \mathrm{d}s^N \exp\left[-\beta \hat{H}\left(s^N, V\right)\right]} \tag{S40}$$

$$\frac{\partial\langle V\rangle}{\partial\beta} = \frac{\partial}{\partial\beta}\left(\frac{\int \mathrm{d}V\, V^{N+1} \int \mathrm{d}s^N \exp\left[-\beta \hat{H}\left(s^N, V\right)\right]}{\int \mathrm{d}V\, V^{N} \int \mathrm{d}s^N \exp\left[-\beta \hat{H}\left(s^N, V\right)\right]}\right)_P \tag{S41}$$

$$= \left[\frac{\frac{\partial}{\partial\beta}\left(\int \mathrm{d}V\, V^{N+1} \int \mathrm{d}s^N \exp\left[-\beta \hat{H}\left(s^N, V\right)\right]\right)_P \left(\int \mathrm{d}V\, V^{N} \int \mathrm{d}s^N \exp\left[-\beta \hat{H}\left(s^N, V\right)\right]\right)}{\left[\int \mathrm{d}V\, V^{N} \int \mathrm{d}s^N \exp\left[-\beta \hat{H}\left(s^N, V\right)\right]\right]^2}\right]$$
$$- \left[\frac{\frac{\partial}{\partial\beta} \times \left(\int \mathrm{d}V\, V^{N} \int \mathrm{d}s^N \exp\left[-\beta \hat{H}\left(s^N, V\right)\right]\right)}{\int \mathrm{d}V\, V^{N} \int \mathrm{d}s^N \exp\left[-\beta \hat{H}\left(s^N, V\right)\right]} \times \frac{\int \mathrm{d}V\, V^{N+1} \int \mathrm{d}s^N \exp\left[-\beta \hat{H}\left(s^N, V\right)\right]}{\int \mathrm{d}V\, V^{N} \int \mathrm{d}s^N \exp\left[-\beta \hat{H}\left(s^N, V\right)\right]}\right] \tag{S42}$$

$$= -\frac{\int \mathrm{d}V\, V^{N} (\hat{H}V) \int \mathrm{d}s^N \exp\left[-\beta \hat{H}\left(s^N, V\right)\right]}{\int \mathrm{d}V\, V^{N} \int \mathrm{d}s^N \exp\left[-\beta \hat{H}\left(s^N, V\right)\right]} + \langle\hat{H}\rangle\langle V\rangle \tag{S43}$$

$$= \langle\hat{H}\rangle\langle V\rangle - \langle V\hat{H}\rangle \tag{S44}$$

By substituting the result from Eq. S44 in Eq. S31 we obtain

$$C_P^{\text{residual}}(T,P) = \frac{1}{k_{\text{B}}T^2}\left[\left\langle U^{\text{external}}\hat{H}\right\rangle - \left\langle U^{\text{external}}\right\rangle\langle\hat{H}\rangle + P(\langle V\hat{H}\rangle - \langle V\rangle\langle\hat{H}\rangle)\right] - Nk_{\text{B}} \tag{S45}$$

The same expression for C_P^{residual} is also reported by Refs.[31,37–39]

S7 Derivation of the isothermal compressibility (β_T)

In this section, the derivation of an equation to compute the isothermal compressibility is presented. The isothermal compressibility of a fluid is defined as the measure of the relative volume change of a fluid with respect to pressure changes at constant temperature.

$$\beta_T(T,P) = -\frac{1}{\langle V\rangle}\left(\frac{\partial\langle V\rangle}{\partial P}\right)_T \tag{S46}$$

From this definition, it is clear that one requires the partial derivative of $\langle V\rangle$ with respect to P. The partial derivative of $\langle V\rangle$ with respect to P is

$$\left(\frac{\partial\langle V\rangle}{\partial P}\right)_T = \frac{\partial}{\partial P}\left(\frac{\int \mathrm{d}V\, V^{N+1}\int \mathrm{d}s^N \exp\left[-\beta\hat{H}\left(s^N,V\right)\right]}{\int \mathrm{d}V\, V^{N}\int \mathrm{d}s^N \exp\left[-\beta\hat{H}\left(s^N,V\right)\right]}\right)_T \tag{S47}$$

Applying the quotient rule, we obtain

$$\begin{aligned}\left(\frac{\partial\langle V\rangle}{\partial P}\right)_T = &\left[\frac{\left(\int \mathrm{d}V\, V^{N}\int \mathrm{d}s^N \exp\left[-\beta\hat{H}\left(s^N,V\right)\right]\right)}{\int \mathrm{d}V\, V^{N}\int \mathrm{d}s^N \exp\left[-\beta\hat{H}\left(s^N,V\right)\right]}\right.\\
&\left.\times\frac{\frac{\partial}{\partial P}\left(\int \mathrm{d}V\, V^{N+1}\int \mathrm{d}s^N \exp\left[-\beta\hat{H}\left(s^N,V\right)\right]\right)}{\int \mathrm{d}V\, V^{N}\int \mathrm{d}s^N \exp\left[-\beta\hat{H}\left(s^N,V\right)\right]}\right]\\
&(-)\left[\frac{\frac{\partial}{\partial P}\left(\int \mathrm{d}V\, V^{N}\int \mathrm{d}s^N \exp\left[-\beta\hat{H}\left(s^N,V\right)\right]\right)}{\int \mathrm{d}V\, V^{N}\int \mathrm{d}s^N \exp\left[-\beta\hat{H}\left(s^N,V\right)\right]}\right.\\
&\left.\times\frac{\int \mathrm{d}V\, V^{N+1}\int \mathrm{d}s^N \exp\left[-\beta\hat{H}\left(s^N,V\right)\right]}{\int \mathrm{d}V\, V^{N}\int \mathrm{d}s^N \exp\left[-\beta\hat{H}\left(s^N,V\right)\right]}\right]\end{aligned} \tag{S48}$$

$$\begin{aligned}= &\left[\frac{\frac{\partial}{\partial P}\times\left(\int \mathrm{d}V\, V^{N+1}\int \mathrm{d}s^N \exp\left[-\beta\hat{H}\left(s^N,V\right)\right]\right)}{\int \mathrm{d}V\, V^{N}\int \mathrm{d}s^N \exp\left[-\beta\hat{H}\left(s^N,V\right)\right]}\right]\\
&(-)\left[\frac{\frac{\partial}{\partial P}\times\left(\int \mathrm{d}V\, V^{N}\int \mathrm{d}s^N \exp\left[-\beta\hat{H}\left(s^N,V\right)\right]\right)}{\int \mathrm{d}V\, V^{N}\int \mathrm{d}s^N \exp\left[-\beta\hat{H}\left(s^N,V\right)\right]}\times\langle V\rangle\right]\end{aligned} \tag{S49}$$

$$\left(\frac{\partial\langle V\rangle}{\partial P}\right)_T = \left[\frac{\int \mathrm{d}V\, V^{N+1}(-\beta V)\int \mathrm{d}s^N \exp\left[-\beta\hat{H}\left(s^N,V\right)\right]}{\int \mathrm{d}V\, V^N \int \mathrm{d}s^N \exp\left[-\beta\hat{H}\left(s^N,V\right)\right]}\right]$$
$$(-)\left[\frac{\int \mathrm{d}V\, V^{N}(-\beta V)\int \mathrm{d}s^N \exp\left[-\beta\hat{H}\left(s^N,V\right)\right]}{\int \mathrm{d}V\, V^N \int \mathrm{d}s^N \exp\left[-\beta\hat{H}\left(s^N,V\right)\right]}\times\langle V\rangle\right] \tag{S50}$$

$$= \left[\frac{\int \mathrm{d}V\, V^{N+2}(-\beta)\int \mathrm{d}s^N \exp\left[-\beta\hat{H}\left(s^N,V\right)\right]}{\int \mathrm{d}V\, V^N \int \mathrm{d}s^N \exp\left[-\beta\hat{H}\left(s^N,V\right)\right]}\right]$$
$$(-)\left[\frac{\int \mathrm{d}V\, V^{N+1}(-\beta)\int \mathrm{d}s^N \exp\left[-\beta\hat{H}\left(s^N,V\right)\right]}{\int \mathrm{d}V\, V^N \int \mathrm{d}s^N \exp\left[-\beta\hat{H}\left(s^N,V\right)\right]}\times\langle V\rangle\right] \tag{S51}$$

$$= \beta\langle V\rangle \times \langle V\rangle - \beta\langle V\rangle^2 \tag{S52}$$

$$= \beta\left[\langle V\rangle^2 - \langle V^2\rangle\right] \tag{S53}$$

Substituting Eq. S53 in Eq. S46 yields

$$\beta_T(T,P) = \frac{1}{\langle V\rangle k_\mathrm{B}T}\left[\langle V^2\rangle - \langle V\rangle^2\right] \tag{S54}$$

The same expression for β_T is also reported by Refs.[31,39,40]

S8 Derivation of the thermal expansivity coefficient at constant pressure (α_P)

In this section, the derivation of an equation to compute the thermal expansivity of a fluid is presented. The thermal expansivity of a fluid is defined as the measure of the relative volume change of a fluid with respect to temperature changes at constant pressure.

$$\alpha_P = \frac{1}{\langle V \rangle} \left(\frac{\partial \langle V \rangle}{\partial T} \right)_P \tag{S55}$$

Where,

$$\frac{\partial \langle V \rangle}{\partial T} = \frac{\partial \langle V \rangle}{\partial \beta} \frac{\partial \beta}{\partial T} = -\frac{1}{k_\mathrm{B} T^2} \frac{\partial \langle V \rangle}{\partial \beta} \tag{S56}$$

From Eq. S44, we know that

$$\frac{\partial \langle V \rangle}{\partial \beta} = \langle \hat{H} \rangle \langle V \rangle - \langle V \hat{H} \rangle \tag{S57}$$

Substituting the above equation in Eq. S56, we obtain

$$\frac{\partial \langle V \rangle}{\partial T} = -\frac{1}{k_\mathrm{B} T^2} \left[\langle \hat{H} \rangle \langle V \rangle - \langle V \hat{H} \rangle \right] \tag{S58}$$

Substituting the above equation in Eq. S55, we obtain

$$\alpha_P = \frac{1}{\langle V \rangle k_\mathrm{B} T^2} \left[\langle V \hat{H} \rangle - \langle \hat{H} \rangle \langle V \rangle \right] \tag{S59}$$

The exact same expression for α_P is also reported by Refs.[31,37,39]

S9 Derivation of the speed of sound (c)

The speed of sound of a fluid is defined as[11]

$$c^2(T,P) = \frac{v}{M\beta_S} \tag{S60}$$

where M and v are the molar mass and molar volume of the pure component or mixture respectively. For a mixture, M can be calculated from the pure component molar mass:

$$M = \sum_i^n x_i M_i \tag{S61}$$

where n is the number of components present in the mixture, x_i and M_i are the mole fraction and molar mass of each component present in the mixture. The molar volume of the mixture is calculated from,

$$v = \frac{VN_A}{N} \tag{S62}$$

where V is the volume of the system, N_A is Avagadro's number, and N is the number of molecules in the system. β_S is the isentropic compressibility. β_S is defined as

$$\beta_S(T,P) = -\frac{1}{V}\left(\frac{\partial V}{\partial P}\right)_S \tag{S63}$$

The term $\left(\frac{\partial V}{\partial P}\right)_S$ can also be rewritten using the Maxwell relations:

$$\left(\frac{\partial V}{\partial P}\right)_S = \frac{\left(\frac{\partial T}{\partial P}\right)_S}{\left(\frac{\partial T}{\partial V}\right)_S} = -\frac{\left(\frac{\partial V}{\partial S}\right)_P}{\left(\frac{\partial P}{\partial S}\right)_V} \tag{S64}$$

$$= -\frac{\left(\frac{\partial V}{\partial T}\right)_P \left(\frac{\partial T}{\partial S}\right)_P}{\left(\frac{\partial P}{\partial T}\right)_V \left(\frac{\partial T}{\partial S}\right)_V} = -\frac{\left(\frac{\partial V}{\partial T}\right)_P \left(\frac{\partial S}{\partial T}\right)_V}{\left(\frac{\partial P}{\partial T}\right)_V \left(\frac{\partial S}{\partial T}\right)_P} \tag{S65}$$

The change in entropy with respect to temperature can be written in terms of heat capacities using

$$C_V(T,V) = \left(\frac{\partial U}{\partial T}\right)_V = T\left(\frac{\partial S}{\partial T}\right)_V \tag{S66}$$

$$C_P(T,P) = \left(\frac{\partial H}{\partial T}\right)_P = T\left(\frac{\partial S}{\partial T}\right)_P \tag{S67}$$

After substituting the heat capacities in Eq. S65, we obtain

$$\left(\frac{\partial V}{\partial P}\right)_S = -\frac{C_V(T,V)\left(\frac{\partial V}{\partial T}\right)_P}{C_P(T,P)\left(\frac{\partial P}{\partial T}\right)_V} \tag{S68}$$

Using the Maxwell relations, we obtain

$$\left(\frac{\partial V}{\partial P}\right)_S = \frac{C_V(T,V)\left(\frac{\partial S}{\partial P}\right)_T}{C_P(T,P)\left(\frac{\partial S}{\partial V}\right)_T} = \frac{C_V(T,V)}{C_P(T,P)}\left(\frac{\partial V}{\partial P}\right)_T \tag{S69}$$

Substituting Eq. S69 in Eq. S63 yields

$$\beta_S(T,P) = \frac{C_V(T,V)}{C_P(T,P)}\left(-\frac{1}{V}\left(\frac{\partial V}{\partial P}\right)_T\right) \tag{S70}$$

We know that

$$\beta_T(T,P) = -\frac{1}{V}\left(\frac{\partial V}{\partial P}\right)_T \tag{S71}$$

Hence, $\beta_S(T,P)$ can be written as

$$\beta_S(T,P) = \frac{\beta_{\mathrm{T}}(T,P)C_V(T,V)}{C_P(T,P)} \tag{S72}$$

Substituting the above equation in Eq. S60, we obtain

$$c^2(T, P) = \frac{vC_P(T, P)}{MC_V(T, V)\beta_{\mathrm{T}}(T, P)} \tag{S73}$$

The same expression for c is also reported by Refs.[32,33]

S10 Derivation of the Joule-Thomson coefficient (μ_{JT})

The Joule-Thomson coefficient (μ_{JT}) is defined as the rate of change of temperature with pressure during an isenthalpic process.

$$\mu_{\mathrm{JT}} = \left(\frac{\partial T}{\partial P}\right)_H = -\frac{\left(\frac{\partial H}{\partial P}\right)_T}{C_P} \tag{S74}$$

The term $\left(\frac{\partial H}{\partial P}\right)_T$ can be obtained from

$$\left(\frac{\partial H}{\partial P}\right)_T = T\left(\frac{\partial S}{\partial P}\right)_T + V \tag{S75}$$

$$= V - T\left(\frac{\partial V}{\partial T}\right)_P \tag{S76}$$

Substituting the above equation in Eq. S74, we obtain

$$\mu_{\mathrm{JT}} = -\frac{1}{C_P}\left[-T\left(\frac{\partial V}{\partial T}\right)_P + V\right] \tag{S77}$$

We know that thermal expansivity at constant P is defined as

$$\alpha_P = \frac{1}{V}\left(\frac{\partial V}{\partial T}\right)_P \tag{S78}$$

Substituting the thermal expansivity in Eq. S77, we obtain the Joule-Thomson coefficient in terms of the thermal expansivity:

$$\mu_{\mathrm{JT}} = \frac{V}{C_P}\left[T\alpha_P - 1\right] \tag{S79}$$

The same expression for μ_{JT} is also reported by Ref.[31,37]

S11 Computation of the uncertainty in the speed of sound (Δc) and Joule-Thomson coefficient ($\Delta \mu_{\mathrm{JT}}$)

The heat capacity at constant volume, heat capacity at constant pressure, isothermal compressibility, and thermal expansivity are calculated from the volume, internal energy, and enthalpy derivatives as shown in Eqs. (S19), (S45), (S54) and (S59). Uncertainties calculated from block averaging, as mentioned in Section 3 of the main text for the speed of sound and the Joule-Thomson coefficient do not account for error propagation. In this section, the expressions to calculate the uncertainty of the speed of sound and the Joule-Thomson coefficient, which takes the error propagation into account, are shown. The uncertainty in Eq. (S73) (Δc^2) is calculated using error propagation as,

$$\Delta c^2 = \sqrt{\left(\frac{\partial c^2}{\partial v} \cdot \Delta v\right)^2 + \left(\frac{\partial c^2}{\partial C_P} \cdot \Delta C_P\right)^2 + \left(\frac{\partial c^2}{\partial \beta_t} \cdot \Delta \beta_t\right)^2 + \left(\frac{\partial c^2}{\partial C_V} \cdot \Delta C_V\right)^2} \tag{S80}$$

Where:

$$\frac{\partial c^2}{\partial v} = \frac{C_P}{M \beta_t C_V} \tag{S81}$$

$$\frac{\partial c^2}{\partial C_P} = \frac{v}{M \beta_t C_V} \tag{S82}$$

$$\frac{\partial c^2}{\partial \beta_t} = -\frac{v C_P}{M \beta_t^2 C_V} \tag{S83}$$

$$\frac{\partial c^2}{\partial C_V} = -\frac{v C_P}{M \beta_t C_V^2} \tag{S84}$$

To find the uncertainty in c (Δc), we can write

$$\Delta c = \frac{C}{2} \left[\frac{\Delta c^2}{c^2}\right] \tag{S85}$$

Similarly, the uncertainty in μ_{JT} ($\Delta\mu_{JT}$) is calculated using error propagation equation

$$\Delta\mu_{JT} = \mu_{JT}\sqrt{\left(\frac{\partial\mu_{JT}}{\partial V}\cdot\Delta V\right)^2 + \left(\frac{\partial\mu_{JT}}{\partial\alpha_p}\cdot\Delta\alpha_p\right)^2 + \left(\frac{\partial\mu_{JT}}{\partial C_P}\cdot\Delta C_P\right)^2} \tag{S86}$$

where

$$\frac{\partial\mu_{JT}}{\partial V} = \frac{T\alpha_p - 1}{C_P} \tag{S87}$$

$$\frac{\partial\mu_{JT}}{\partial\alpha_p} = \frac{VT}{C_P} \tag{S88}$$

$$\frac{\partial\mu_{JT}}{\partial C_P} = -\frac{V\left(T\alpha_p - 1\right)}{C_P^2} \tag{S89}$$

S12 Data of ideal heat capacities at constant volume (c_V^{ideal})

c_V^{ideal} computed from REFPROP[10] and the Gaussian09 software[36] for different pure components are listed in the Tables S28 to S32. The ideal capacities at constant volume were calculated using the B3LYP theory and a 6-31G(d,p) basis set in Gaussian.

Table S28: c_V^{ideal} of CO_2 computed from REFPROP[10] and the Gaussian09 software.[36]

T [K]	c_V^{ideal} - Gaussian [J mol^{-1} K^{-1}]	c_V^{ideal} - REFPROP [J mol^{-1} K^{-1}]
253	27.095	26.672
273	28.057	27.645
293	28.982	28.589
313	29.869	29.497

Table S29: c_V^{ideal} of N_2 computed from REFPROP[10] and the Gaussian09 software.[36]

T [K]	c_V^{ideal} - Gaussian [J mol^{-1} K^{-1}]	c_V^{ideal} - REFPROP [J mol^{-1} K^{-1}]
253	20.786	20.798
273	20.790	20.802
293	20.794	20.808
313	20.798	20.819

Table S30: c_V^{ideal} of Ar computed from REFPROP[10] and the Gaussian09 software.[36]

T [K]	c_V^{ideal} - Gaussian [J mol^{-1} K^{-1}]	c_V^{ideal} - REFPROP [J mol^{-1} K^{-1}]
253	12.472	12.472
273	12.472	12.472
293	12.472	12.472
313	12.472	12.472

Table S31: c_V^{ideal} of H_2 computed from the the Gaussian09 software.[36]

T [K]	c_V^{ideal} - Gaussian [J mol^{-1} K^{-1}]	c_V^{ideal} - REFPROP [J mol^{-1} K^{-1}]
253	20.054	20.051
273	20.297	20.298
293	20.477	20.481
313	20.609	20.615

Table S32: c_V^{ideal} of CH_4 computed from REFPROP[10] and the Gaussian09 software.[36]

T [K]	c_V^{ideal} - Gaussian [J mol^{-1} K^{-1}]	c_V^{ideal} - REFPROP [J mol^{-1} K^{-1}]
253	25.777	26.022
273	26.229	26.553
293	26.794	27.207
313	27.472	27.974

S13 Thermodynamic and transport properties of unary systems

In this section, plots of the thermodynamic and transport properties such as (1) densities (ρ), (2) isothermal compressibilities at constant temperature (β_T), (3) thermal expansion coefficients (α_P), (4) heat capacities at constant volume (c_V), (5) heat capacities at constant pressure (c_P), (6) Joule-Thomson Coefficients (μ_{JT}), (7) speed of sound (c), and (8) viscosities (η) of unary systems at temperatures 253 K, 273 K, 293 K, and 313 K and pressure up to 200 bar are shown. Thermodynamic properties (β_T, α_P, c_V, c_P, μ_{JT}, and c) computed from Monte Carlo (MC) simulations are compared with data calculated from EoS specific to a molecule, as listed in Table S8, which are obtained from REFPROP.[10] Viscosities computed from Molecular Dynamics (MD) simulations are compared with data obtained from REFPROP.[10]

S13.1 Thermodynamic and transport properties of N_2

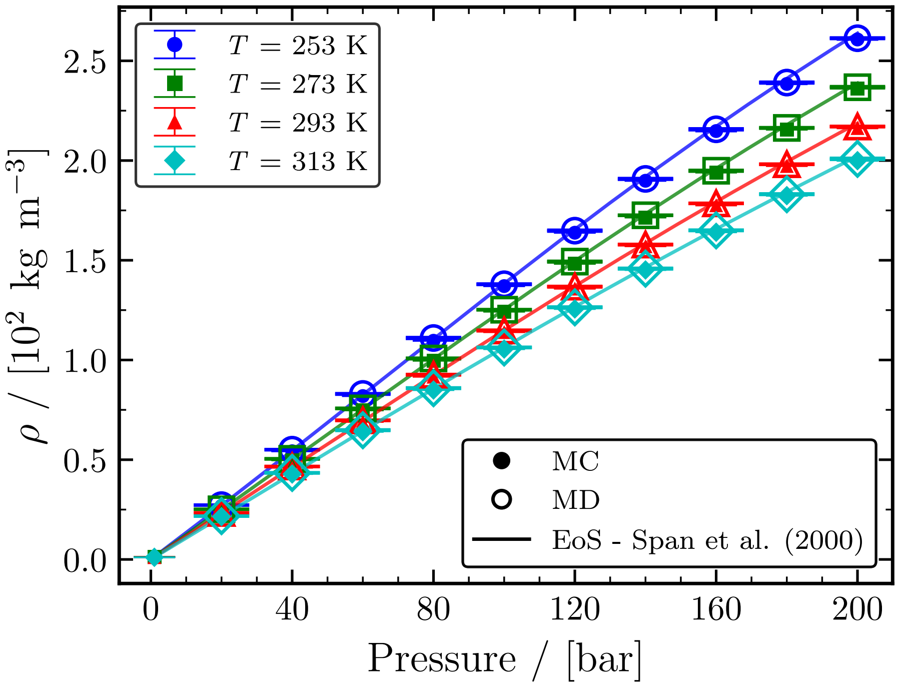

Fig. S4: Densities of N_2 computed from MC and MD simulations, compared with the Span et al. (2000)[16] EoS. Closed symbols represent MC simulations, open symbols represent MD simulations, and dotted lines represent the EoS.

(a)

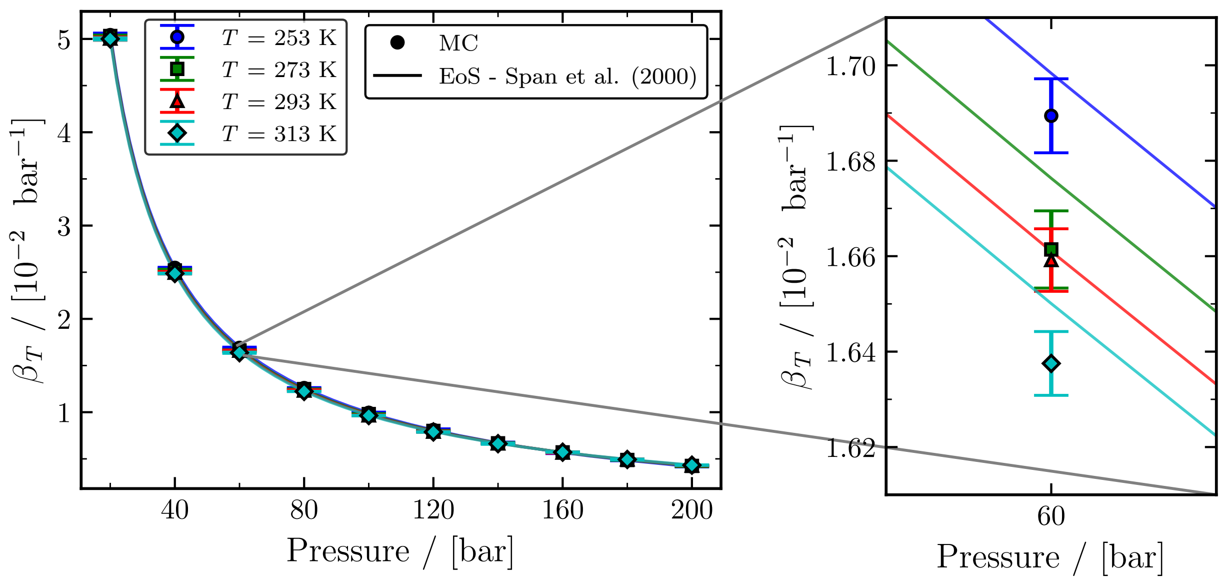

(b)

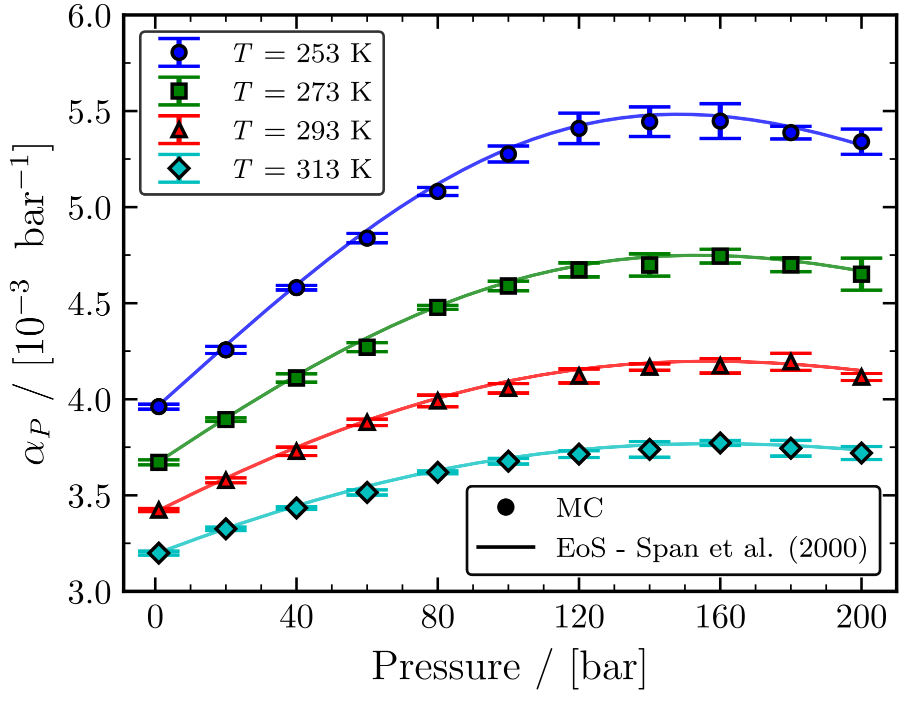

Fig. S5: Computed values of (a) isothermal compressibilities and (b) thermal expansivities of N_2 computed from MC simulations (symbols), compared with the Span et al., (2000)[16] EoS (dotted line).

(a)

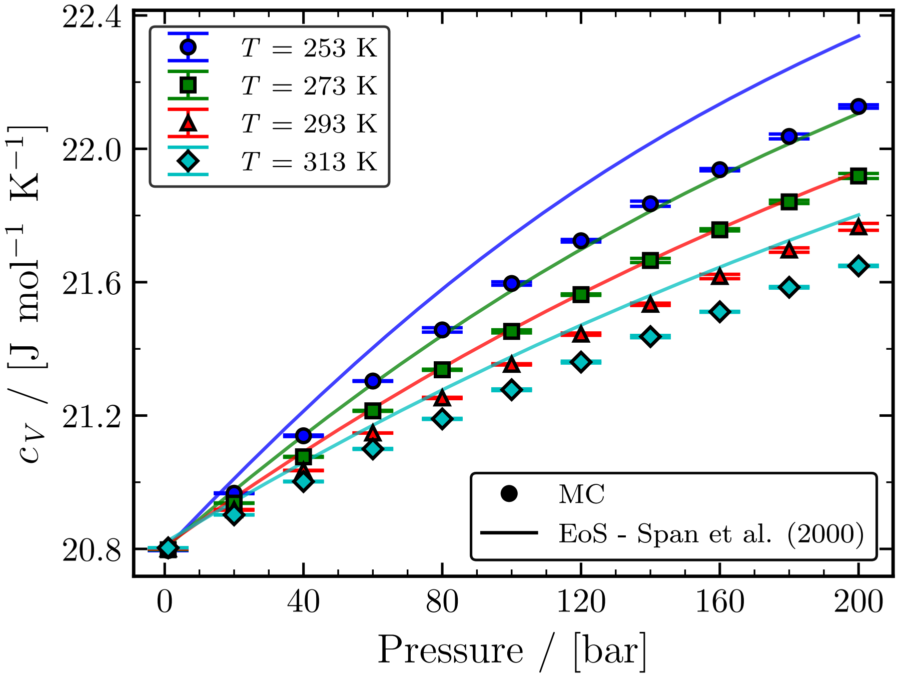

(b)

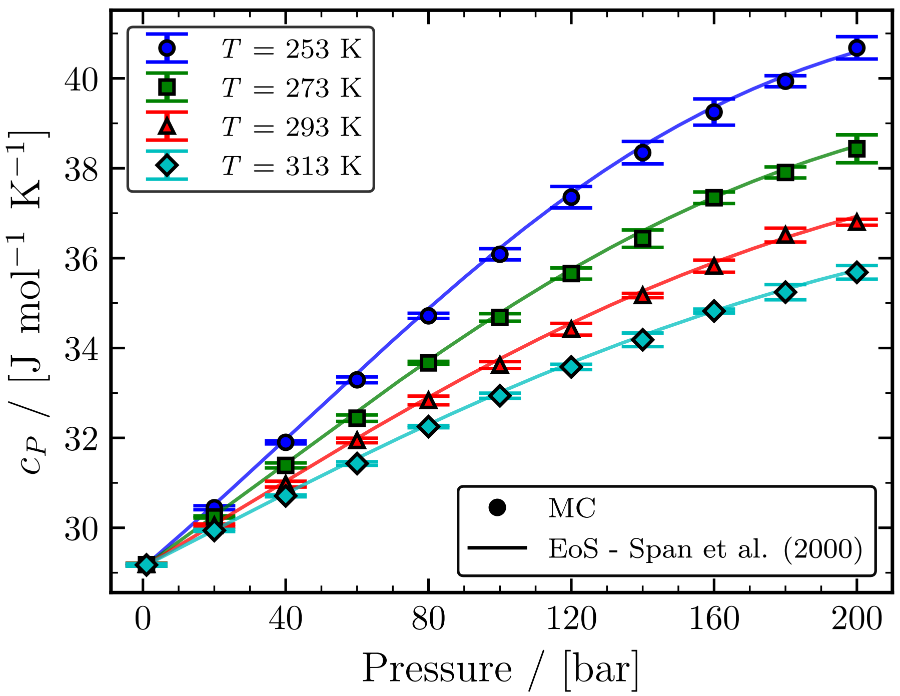

Fig. S6: Computed values of (a) heat capacities at constant volume and (b) heat capacities at constant pressure of N_2 computed from MC simulations (symbols), compared with the Span et al., (2000)[16] EoS (dotted line).

(a)

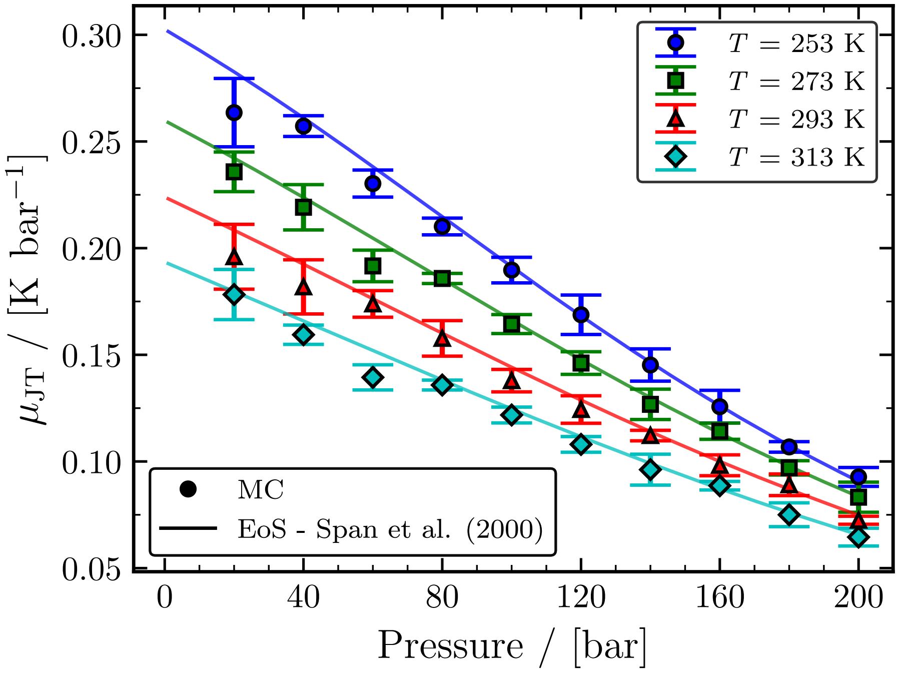

(b)

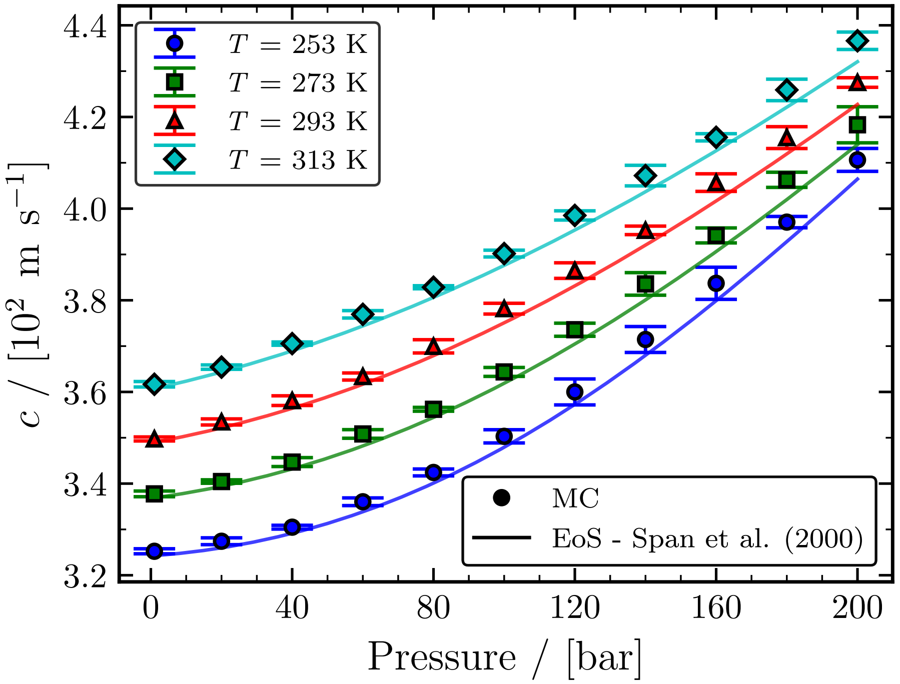

Fig. S7: Computed values of (a) Joule-Thomson coefficients and (b) speed of sound of N_2 computed from MC simulations (symbols), compared with the Span et al., (2000)[16] EoS (dotted line).

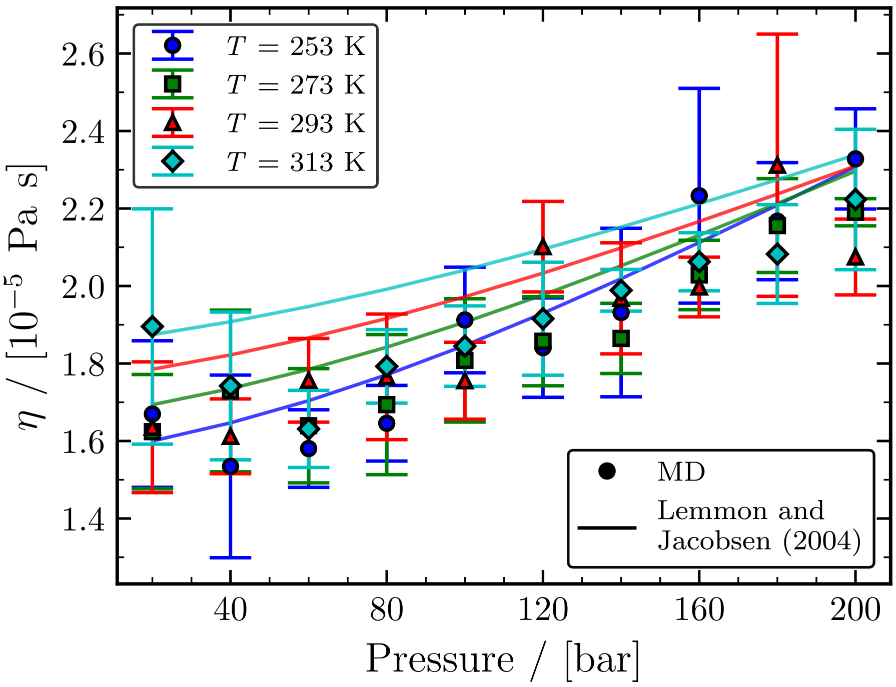

Fig. S8: Viscosities of N_2 computed from MD simulations, compared with the correlation of Lemmon and Jacobsen, (2004),[17] which is obtained from REFPROP.[10] The symbols represent MD simulations and dotted lines represent data from REFPROP.[10]

S13.2 Thermodynamic and transport properties of Ar

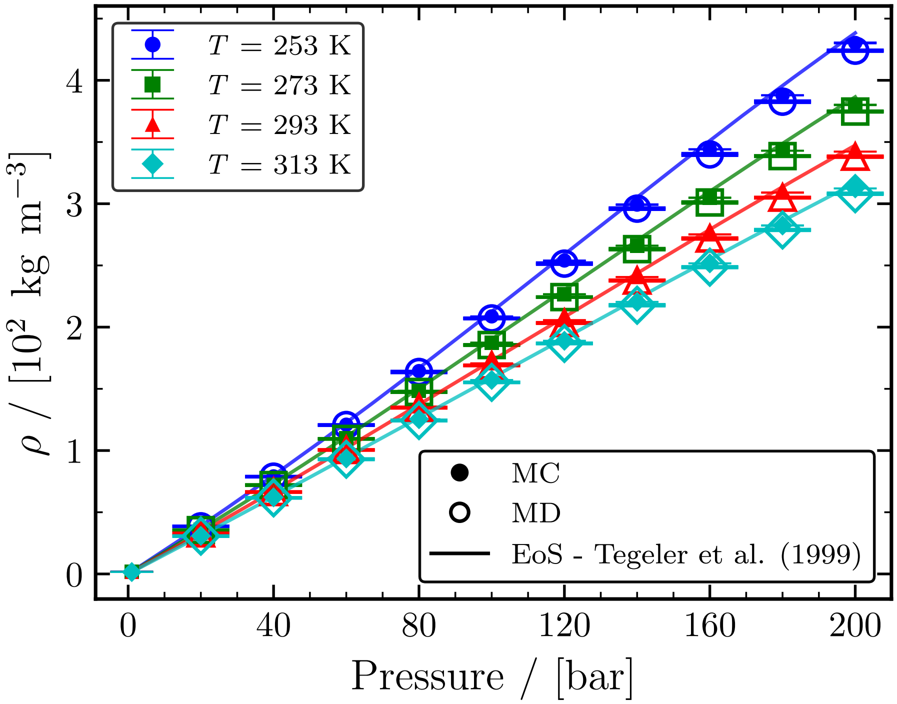

Fig. S9: Densities of argon computed from MC and MD simulations, compared with the Tegeler et al. (1999)[18] EoS. Closed symbols represent MC simulations, open symbols represent MD simulations and dotted lines represent the EoS.

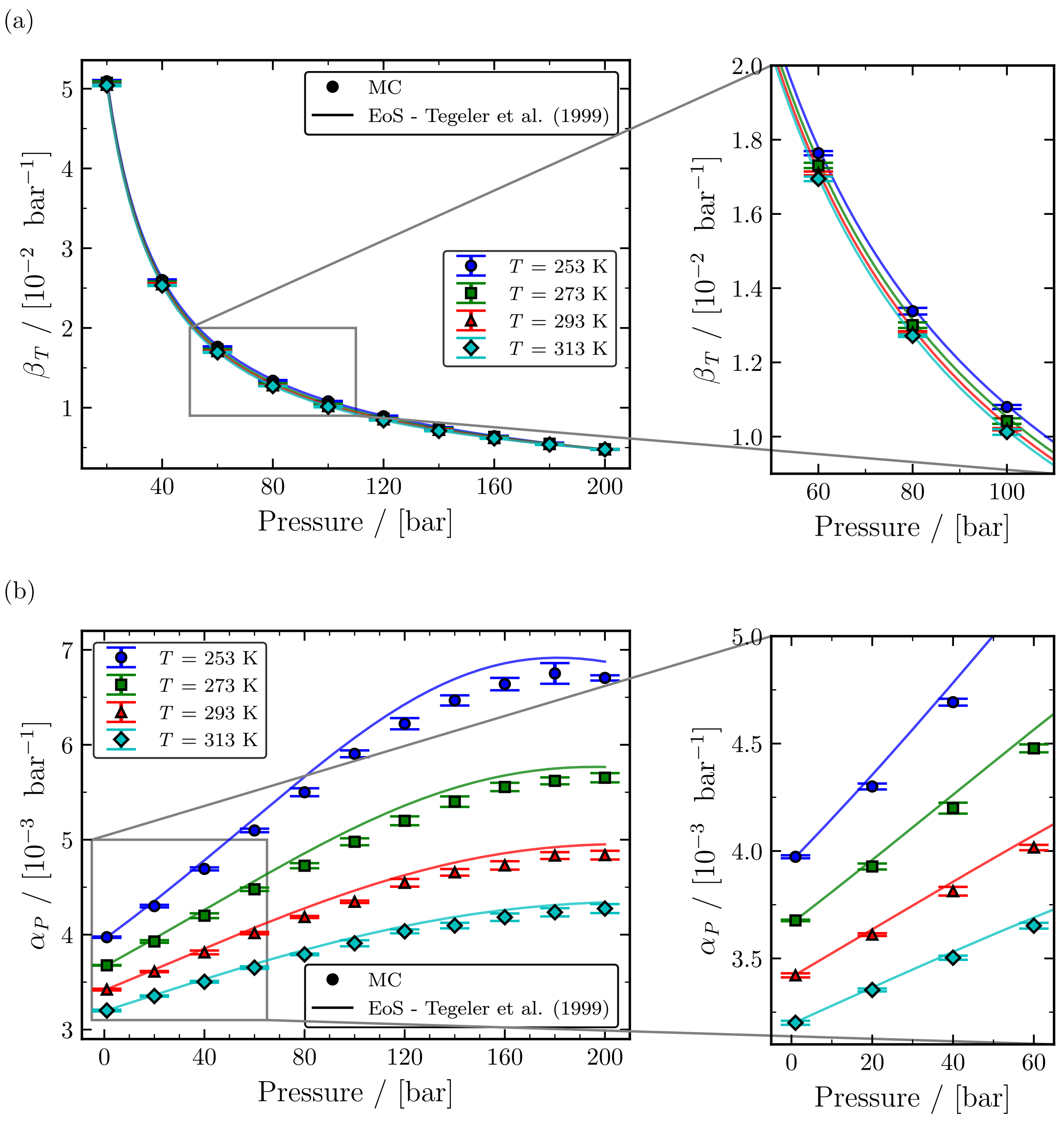

Fig. S10: Computed values of (a) isothermal compressibilities and thermal expansivities (b) of Ar computed from MC simulations (symbols), compared with the Tegeler et al. (1999)[18] EoS (dotted line).

(a)

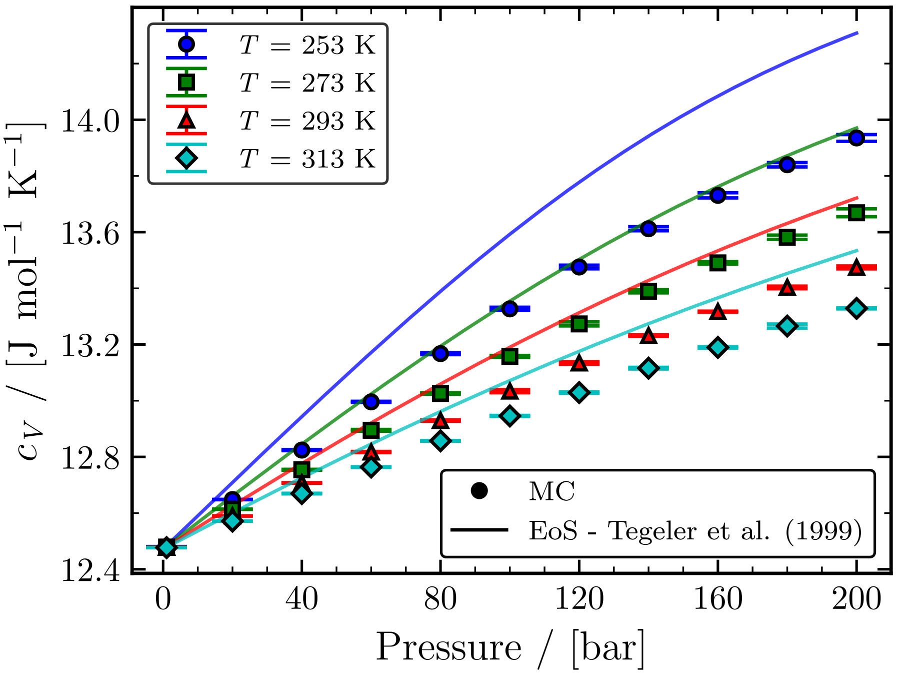

(b)

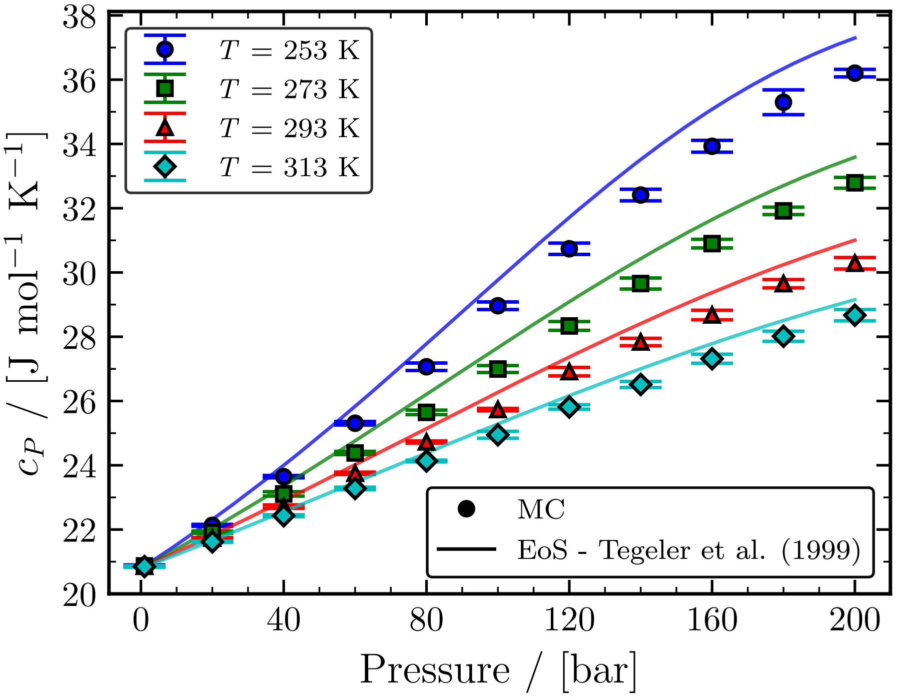

Fig. S11: Computed values of (a) heat capacities at constant volume and (b) heat capacities at constant pressure of Ar computed from MC simulations (symbols), compared with the Tegeler et al. (1999)[18] EoS (dotted line).

(a)

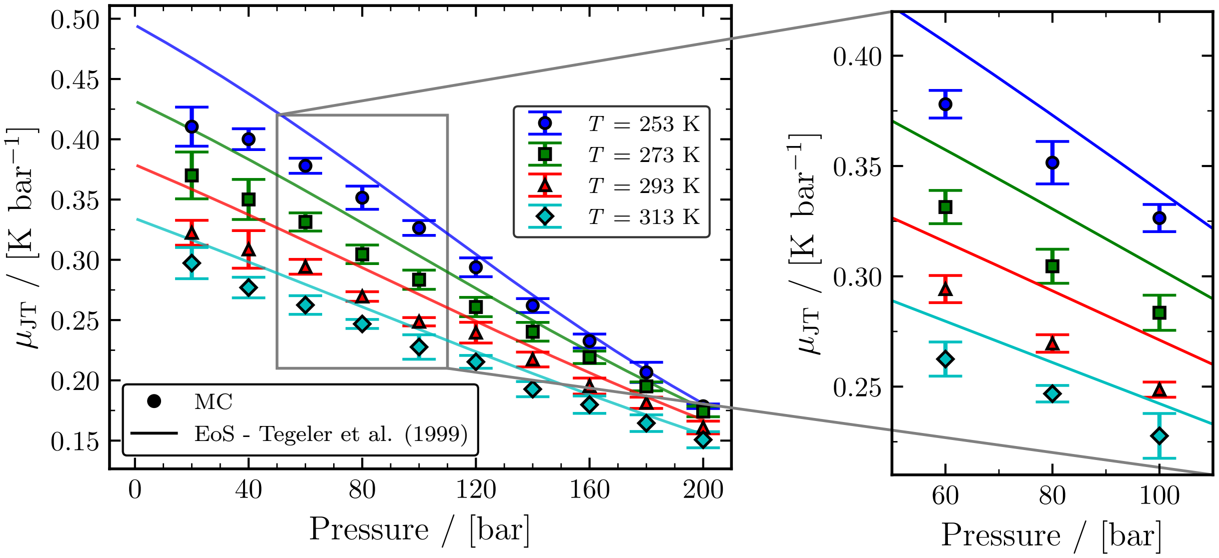

(b)

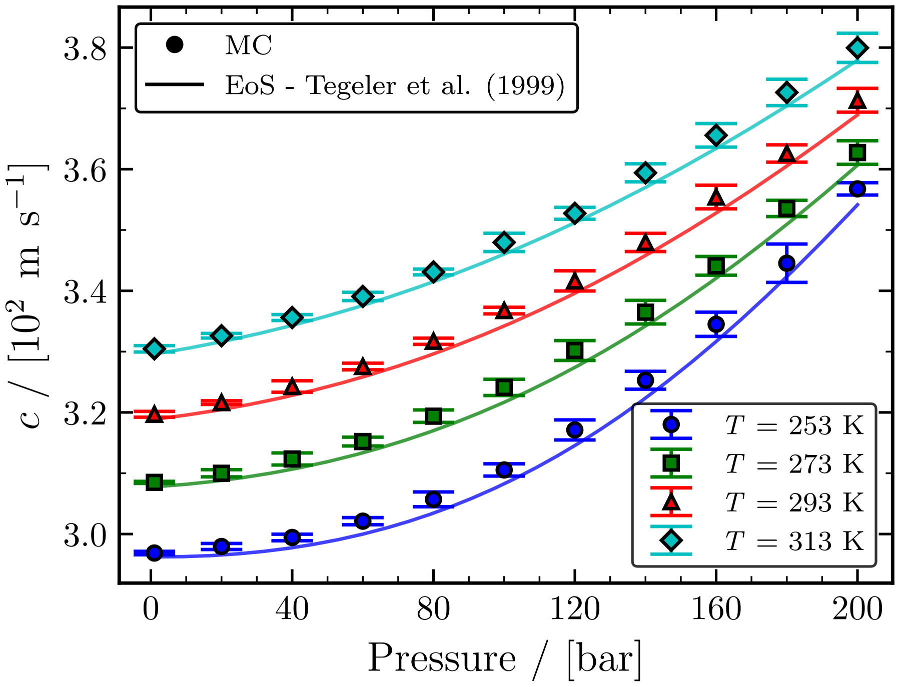

Fig. S12: Computed values of (a) Joule-Thomson coefficients and (b) speed of sound of Ar computed from MC simulations (symbols), compared with the Tegeler et al. (1999)[18] EoS (dotted line).

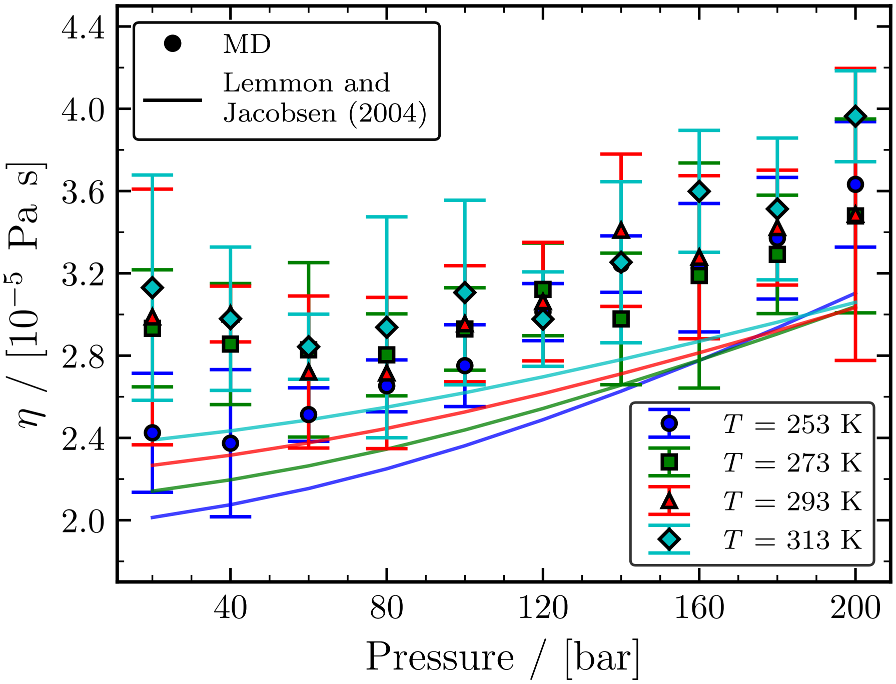

Fig. S13: Viscosities of Ar computed from MD simulations, compared with the correlation of Lemmon and Jacobsen, (2004),[17] which is obtained from REFPROP.[10] The symbols represent MD simulations and dotted lines represent data from REFPROP.[10]

S13.3 Thermodynamic and transport properties of H_2

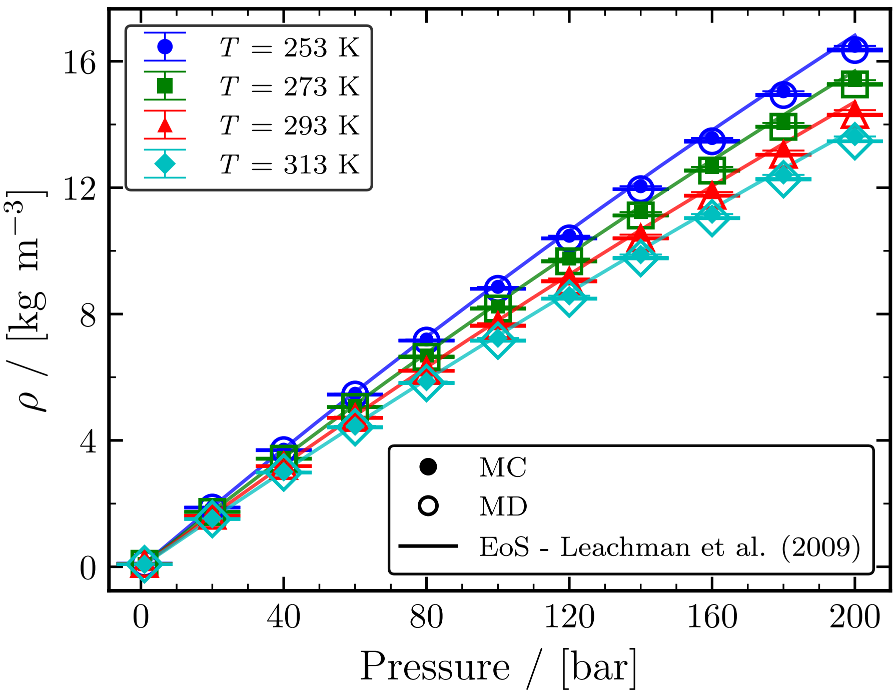

Fig. S14: Densities of H_2 computed from MC and MD simulations, compared with the Leachman et al. (2009)[19] EoS. Closed symbols represent MC simulations, open symbols represent MD simulations, and dotted lines represent the EoS.

(a)

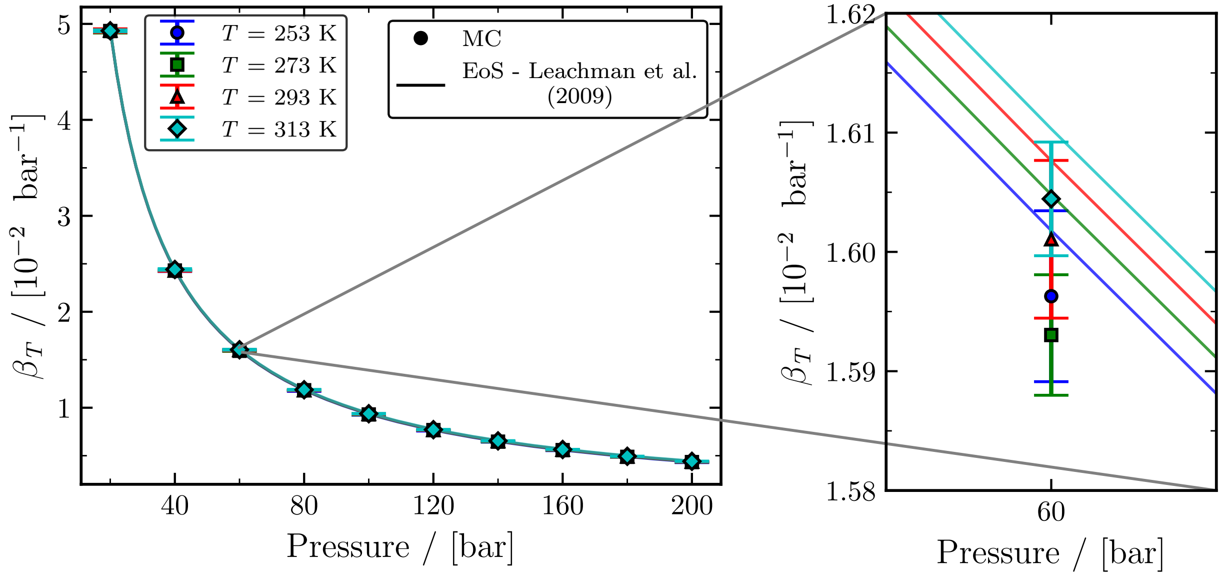

(b)

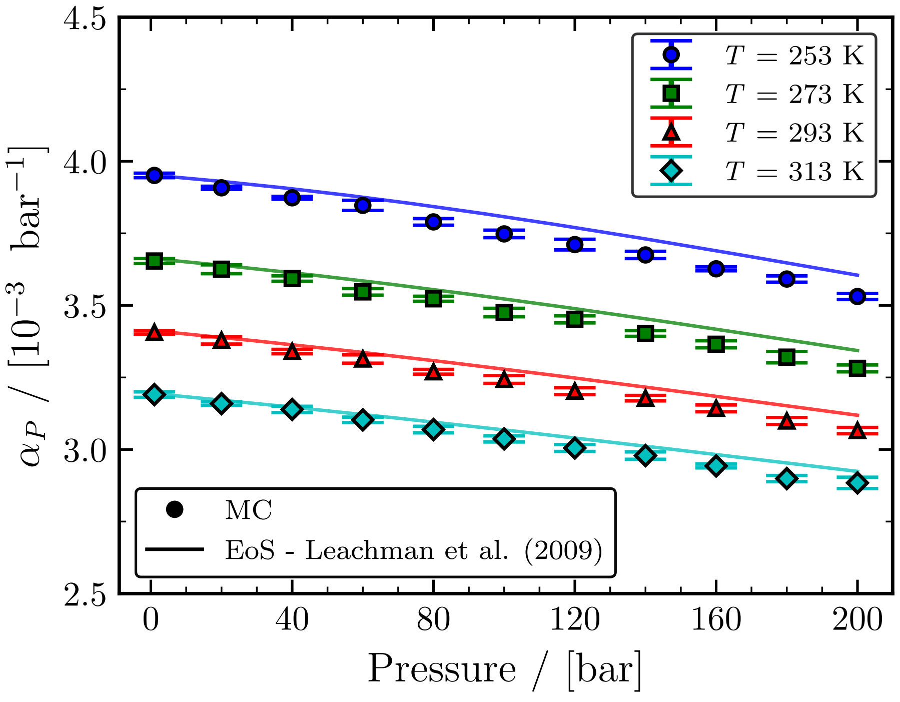

Fig. S15: Computed values of (a) isothermal compressibilities and (b) thermal expansivities of H_2 computed from MC simulations (symbols), compared with the Leachman et al., (2009)[19] EoS (dotted line).

(a)

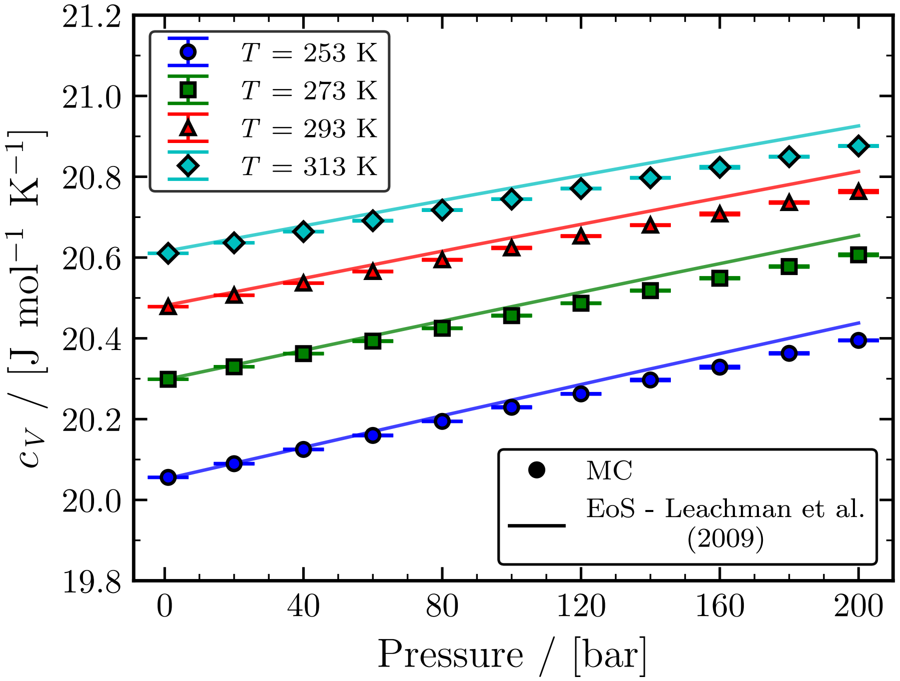

(b)

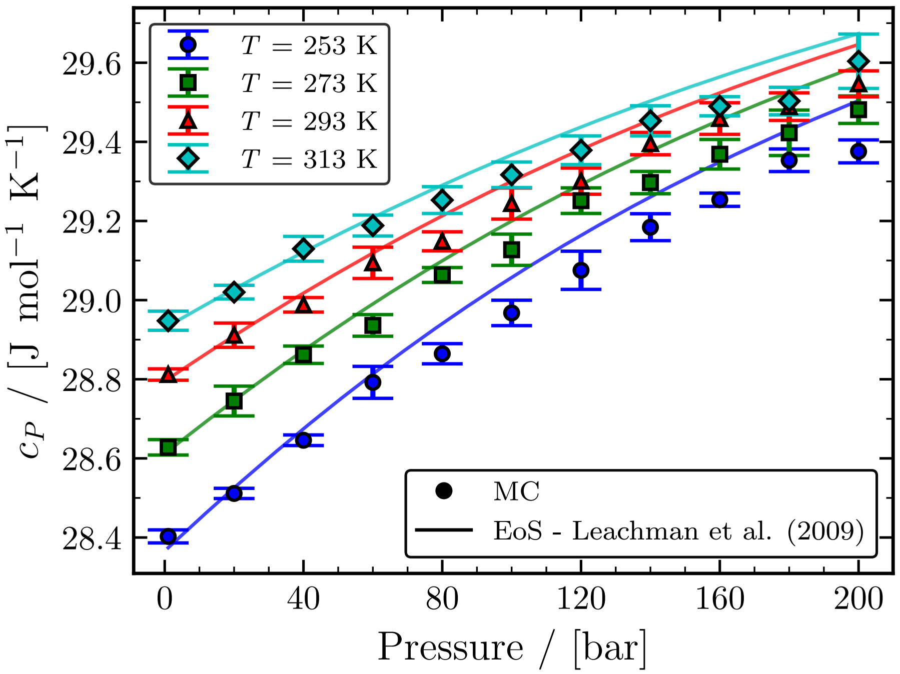

Fig. S16: Computed values of (a) heat capacities at constant volume and (b) heat capacities at constant pressure of H_2 computed from MC simulations (symbols), compared with the Leachman et al., (2009)[19] EoS (dotted line).

(a)

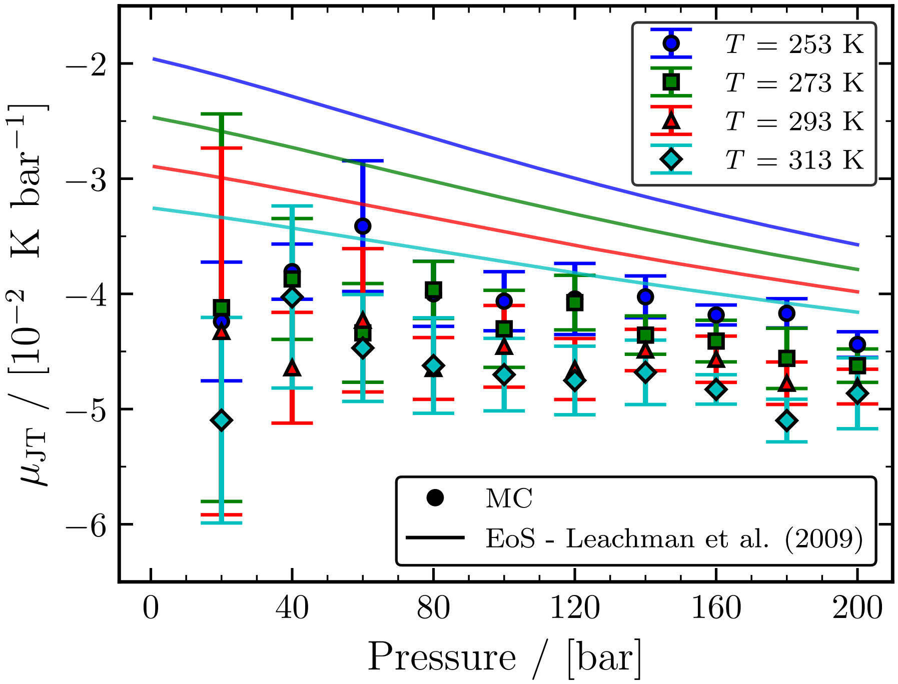

(b)

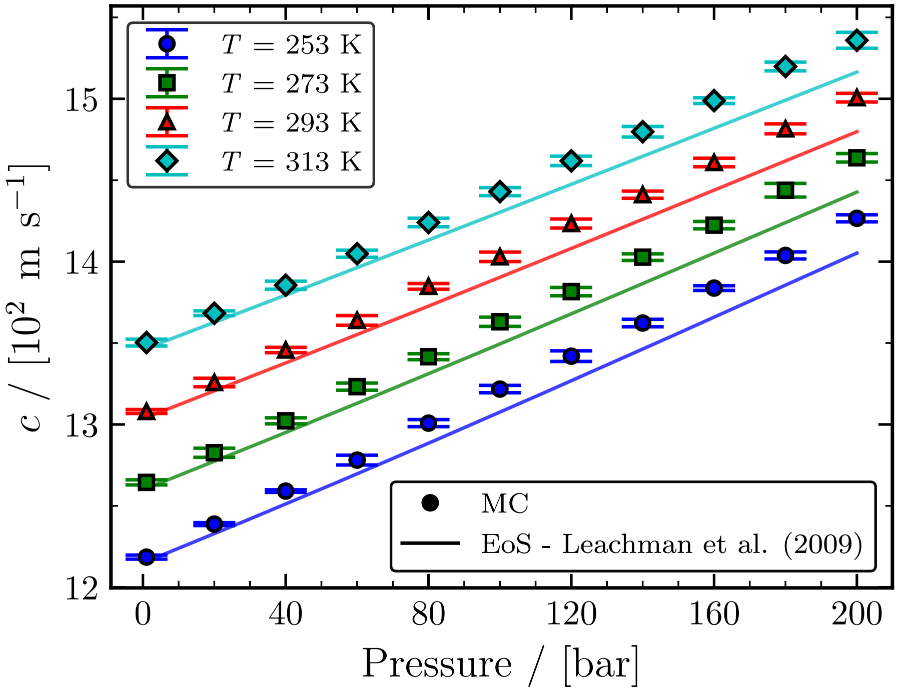

Fig. S17: Computed values of (a) Joule-Thomson coefficients and (b) speed of sound of H_2 computed from MC simulations (symbols), compared with the Tegeler et al. (1999)[19] EoS (dotted line).

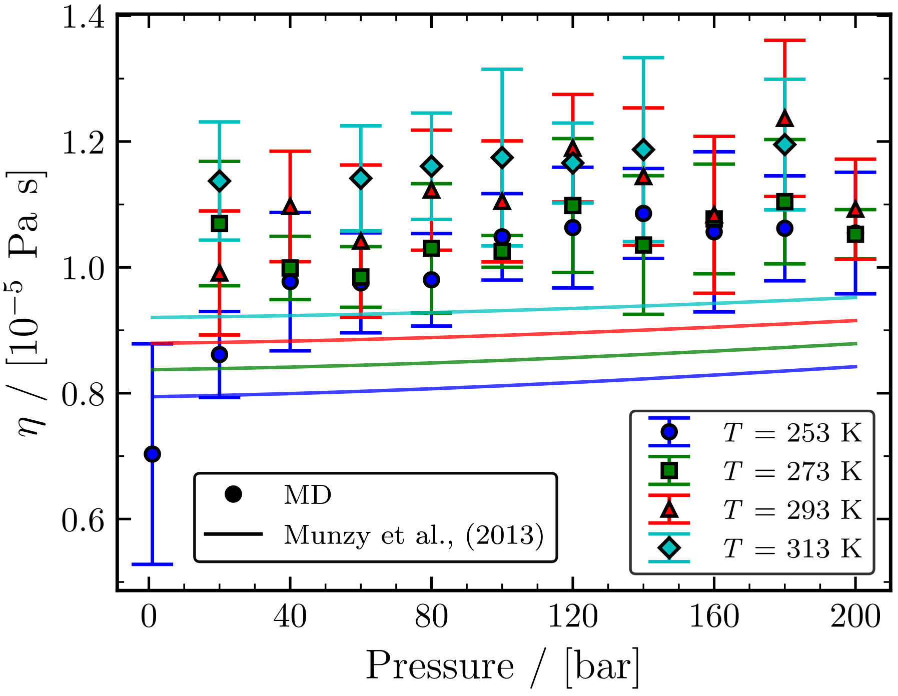

Fig. S18: Viscosities of H_2 computed from MD simulations, compared with the correlation of Munzy et al. (2013),[20] which is obtained from REFPROP.[10] The symbols represent MD simulations and dotted lines represent data from REFPROP.[10]

S13.4 Thermodynamic and transport properties of CH_4

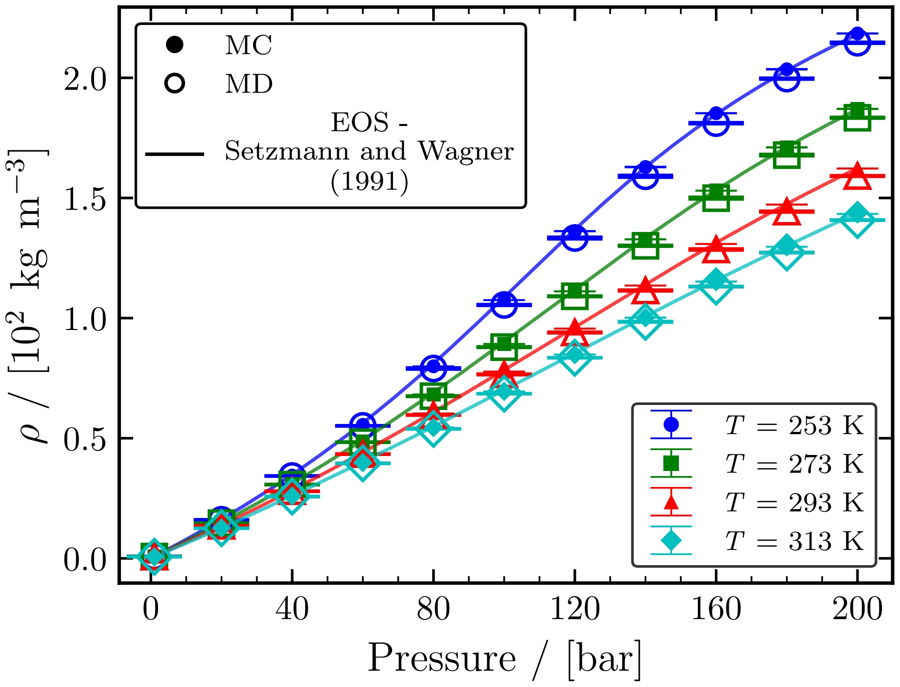

Fig. S19: Densities of CH_4 computed from MC and MD simulations, compared with the Setzmann and Wagner (1991)[21] EoS. Closed symbols represent MC simulations, open symbols represent MD simulations, and dotted lines represent the EoS.

(a)

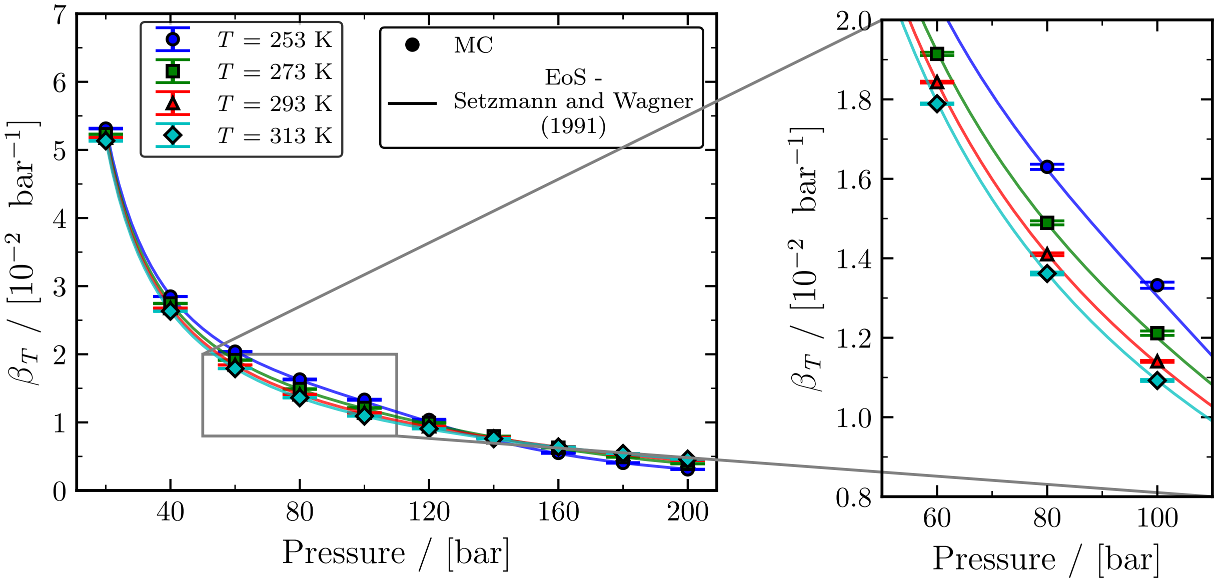

(b)

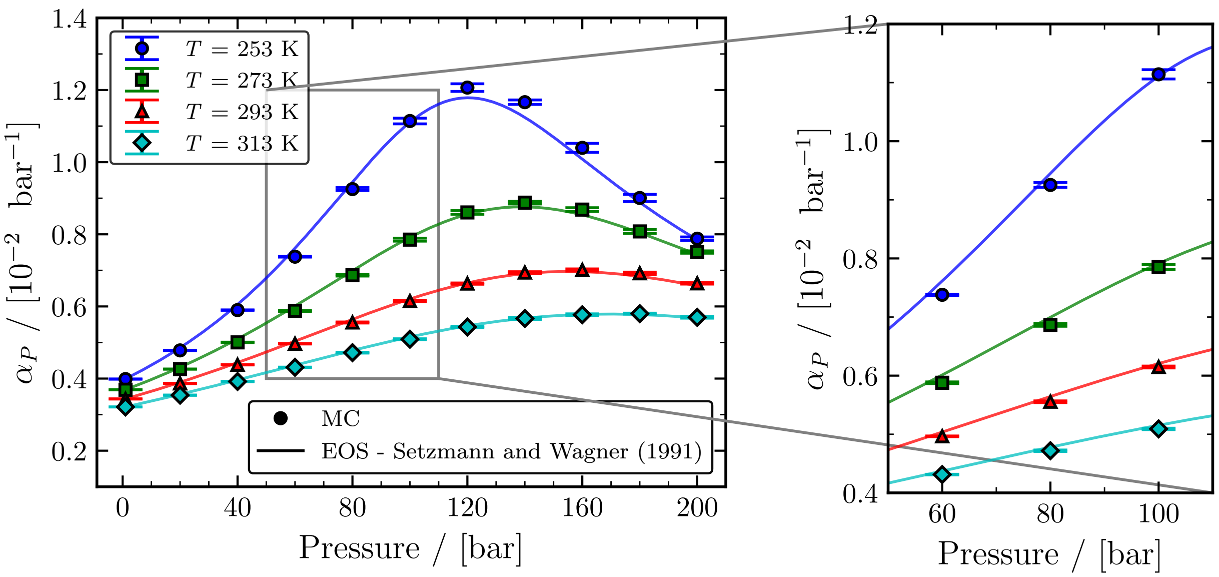

Fig. S20: Computed values of (a) isothermal compressibilities and (b) thermal expansivities of CH_4 computed from MC simulations (symbols), compared with the Setzmann and Wagner (1991)[21] EoS (dotted line).

(a)

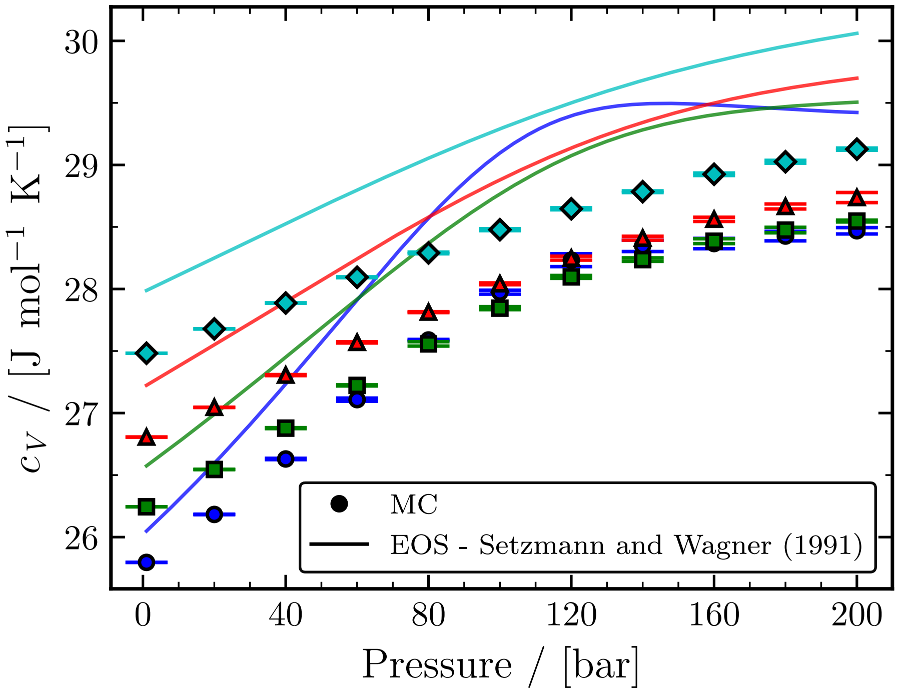

(b)

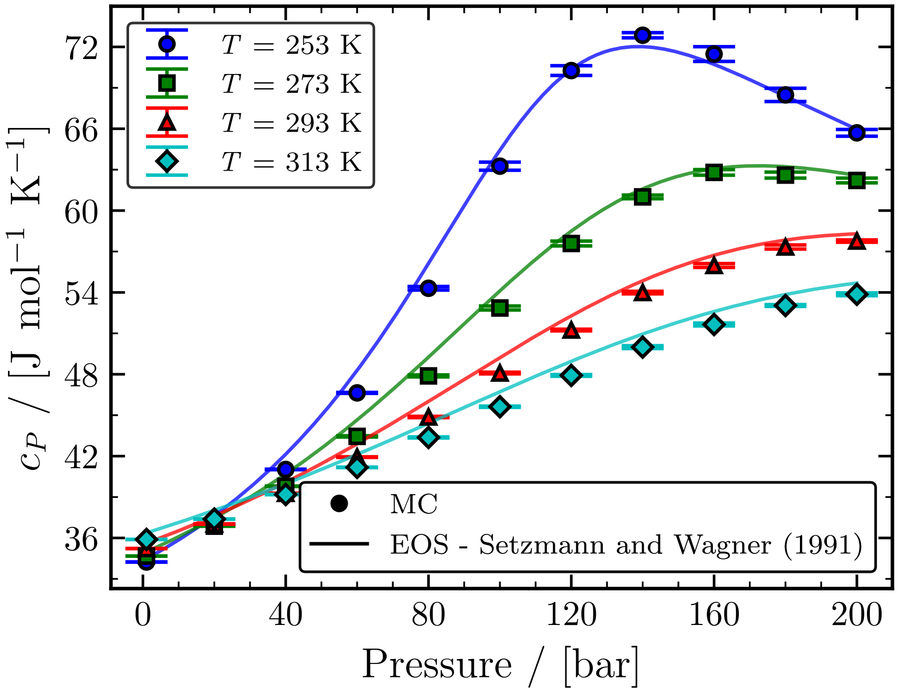

Fig. S21: Computed values of (a) heat capacities at constant volume and (b) heat capacities at constant pressure of CH_4 computed from MC simulations (symbols), compared with the Setzmann and Wagner (1991)[21] EoS (dotted line).

(a)

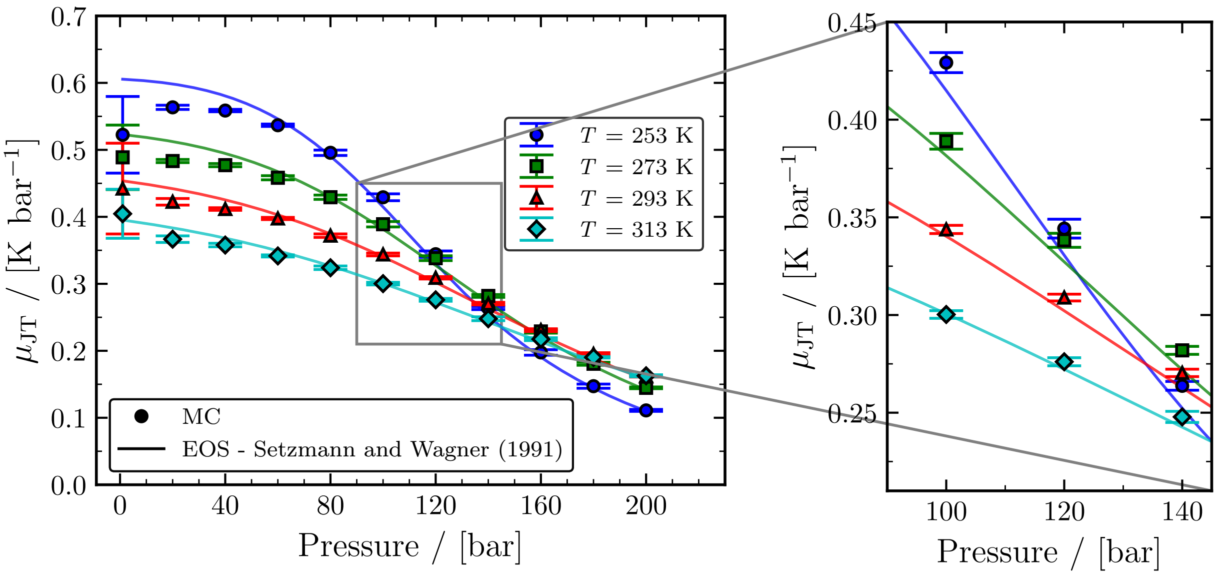

(b)

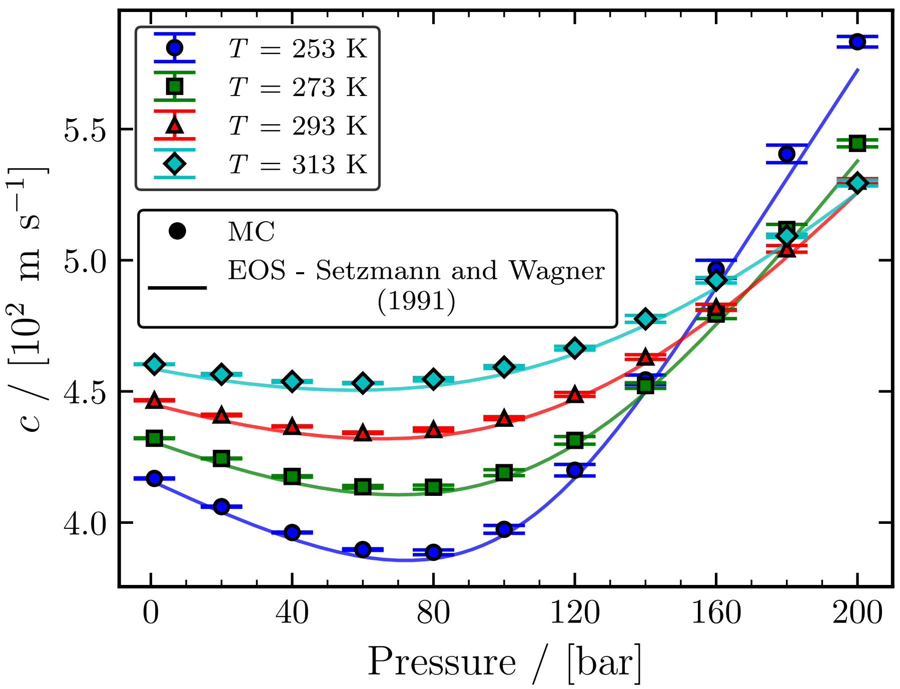

Fig. S22: Computed values of (a) Joule-Thomson coefficients and (b) speed of sound of CH_4 computed from MC simulations (symbols), compared with the Setzmann and Wagner (1991)[21] EoS (dotted line).

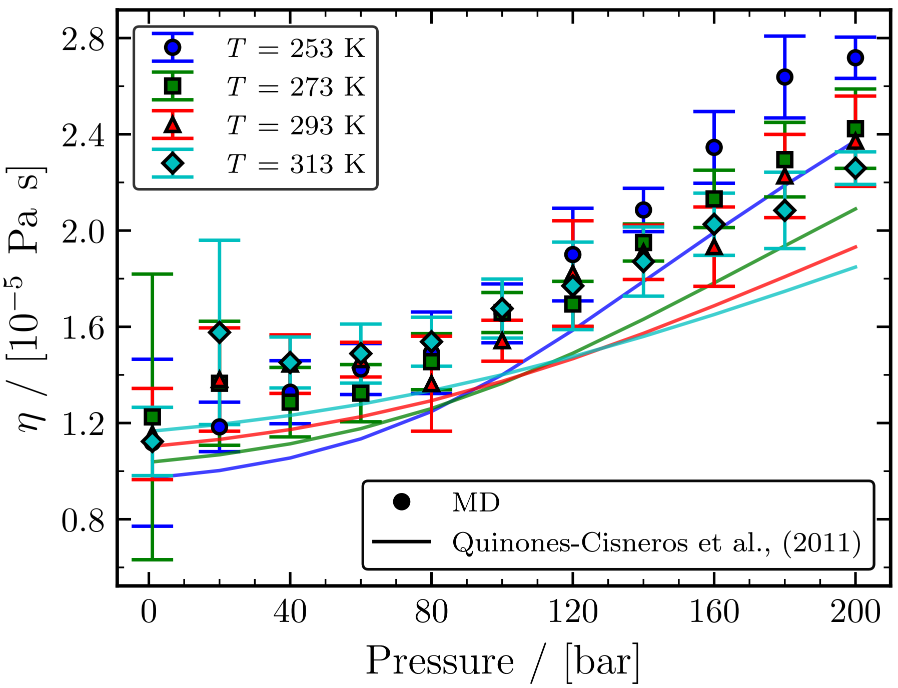

Fig. S23: Viscosities of CH_4 computed from MD simulations, compared with data obtained from REFPROP (Quinones-Cisneros et al., 2011).[10] The symbols represent MD simulations and dotted lines represent data from REFPROP.[10]

S14 Data of thermodynamic and transport properties of unary systems

This section shows the thermodynamic and transport properties with uncertainties computed from Monte Carlo (MC) and Molecular Dynamics (MD) simulations, respectively, for unary systems listed in Table S8. The thermodynamic properties computed from MC simulations are (1) densities (ρ), (2) isothermal compressibilities at constant temperature (β_T), (3) thermal expansion coefficients (α_P), (4) heat capacities at constant volume (c_V), (5) heat capacities at constant pressure (c_P), (6) Joule-Thomson Coefficients (μ_{JT}), and (7) speed of sound (c). Transport property, (8) viscosities (η), are computed from MD simulations. Densities are also computed from MD simulations. The thermodynamic and transport properties are computed at temperatures 253 K, 273 K, 293 K, and 313K, and pressure ranging from 20 bar to 200 bar in intervals of 20 bar. For each condition (concentration, temperature, and pressure), 10 independent simulations are performed, and each simulation starts with a different initial configuration. These 10 simulations are divided into 5 blocks from which average values and uncertainties of thermodynamic and transport properties are calculated as the standard deviation of 5 blocks. The thermodynamic and transport properties uncertainty values with more than one significant decimal figure are not shown. The thermodynamic and transport properties computed from molecular simulations are validated with data obtained from REFPROP[10] which uses the Equation of State (EoS) specific to the component listed in Table S8 are also shown in this section.

S14.1 Data of thermodynamic and transport properties of CO_2

Table S33: Densities computed from MC and MD simulations (ρ^{MC} and ρ^{MD}), densities obtained from REFPROP[10] (ρ^{REFP}), isothermal compressibilities computed from MC simulations (β_T^{MC}), isothermal compressibilities obtained from REFPROP[10] (β_T^{REFP}), thermal expansion coefficients computed from MC simulations (α_P^{MC}), thermal expansion coefficients obtained from REFPROP[10] (α_P^{REFP}), Joule Thomson coefficients computed from MC simulations (μ_{JT}^{MC}), and Joule Thomson coefficients obtained from REFPROP[10] (μ_{JT}^{REFP}) of pure CO_2 at 253 K and pressures ranging from 20 bar to 200 bar.

P/ [bar]	ρ^{MC}/ [kg/m^3]	ρ^{MD}/ [kg/m^3]	ρ^{REFP}/ [kg/m^3]	β_T^{MC}/ [10^{-4}/bar]	β_T^{REFP}/ [10^{-4}/bar]	α_P^{MC}/ [10^{-3}/K]	α_P^{REFP}/ [10^{-3}/K]	μ_{JT}^{MC}/ [10^{-3} K/bar]	μ_{JT}^{REFP}/ [10^{-3} K/bar]
20	1029.0 ± 1.4	1028.8 ± 1.1	1032.5	3.9 ± 0.3	4.5	4.6 ± 0.3	4.7	6.7 ± 3.4	8.8
40	1037.7 ± 0.6	1037.2 ± 0.9	1041.4	3.4 ± 0.1	4.1	4.2	4.4	2.3 ± 0.5	5.6
60	1044.0 ± 0.9	1044.8 ± 0.4	1049.5	3.4 ± 0.1	3.8	4.2 ± 0.1	4.2	2.9 ± 1.6	3.0
80	1051.4 ± 1.1	1051.0 ± 0.2	1057.2	3.1 ± 0.1	3.5	3.9 ± 0.2	4.0	-1.1 ± 2.2	0.6
100	1056.9 ± 0.5	1057.3 ± 0.1	1064.3	2.9 ± 0.1	3.3	3.7 ± 0.1	3.8	-2.9 ± 1.6	−1.5
120	1064.6 ± 0.9	1062.7 ± 0.6	1071.1	2.7 ± 0.1	3.1	3.6 ± 0.1	3.7	-4.6 ± 1.7	−3.3
140	1069.9 ± 0.5	1068.7 ± 0.8	1077.5	2.6	2.9	3.5 ± 0.1	3.5	-5.9 ± 1.0	−4.9
160	1074.3 ± 0.4	1074.2 ± 0.1	1083.6	2.5 ± 0.1	2.7	3.4 ± 0.1	3.4	-6.6 ± 1.1	−6.4
180	1080.4 ± 1.3	1079.6 ± 0.2	1089.4	2.3 ± 0.1	2.6	3.2 ± 0.1	3.3	-9.4 ± 1.4	−7.8
200	1085.5 ± 0.9	1084.9 ± 0.4	1095.0	2.3 ± 0.1	2.5	3.2 ± 0.2	3.2	-8.9 ± 2.2	−9.1

Table S34: Heat capacities at constant volume computed from MC simulations (c_V^{MC}), heat capacities at constant volume obtained from REFPROP[10] (c_V^{REFP}), heat capacities at constant pressure computed from MC simulations (c_P^{MC}), heat capacities at constant pressure obtained from REFPROP[10] (c_P^{REFP}), speed of sound computed from MC simulations (c^{MC}), speed of sound obtained from REFPROP[10] (c^{REFP}), viscosities computed from MD simulations (η^{MD}), and viscosities obtained from REFPROP[10] (η^{REFP}) of pure CO_2 at 253 K and pressures ranging from 20 bar to 200 bar.

P/ [bar]	c_V^{MC}/ [J/mol K]	c_V^{REFP}/ [J/mol K]	c_P^{MC}/ [J/mol K]	c_P^{REFP}/ [J/mol K]	c^{MC}/ [m/s]	c^{REFP}/ [m/s]	η^{MD}/ [μPa s]	η^{REFP}/ [μPa s]
20	40.7 ± 0.3	41.1	98.9 ± 4.0	95.2	778.8 ± 30.7	709.1	138.5 ± 5.8	139.8
40	40.8 ± 0.2	41.2	94.2 ± 1.0	93.0	802.9 ± 8.3	729.8	153.8 ± 5.7	143.8
60	41.1 ± 0.3	41.2	95.9 ± 1.6	91.2	807.1 ± 17.5	748.9	152.6 ± 6.0	147.8
80	40.7 ± 0.3	41.3	92.3 ± 2.6	89.7	839.0 ± 23.1	766.8	157.1 ± 1.4	151.5
100	40.8 ± 0.2	41.4	90.6 ± 2.1	88.3	851.2 ± 17.5	783.7	159.2 ± 7.9	155.2
120	41.1 ± 0.2	41.4	89.5 ± 2.0	87.2	867.9 ± 20.7	799.8	170.0 ± 7.2	158.8
140	41.1 ± 0.2	41.5	89.0 ± 1.8	86.1	883.6 ± 10.9	815.1	160.6 ± 6.0	162.2
160	41.1 ± 0.2	41.5	88.7 ± 1.5	85.2	897.0 ± 13.8	829.8	174.1 ± 7.6	165.6
180	41.2 ± 0.2	41.6	85.9 ± 1.6	84.4	915.4 ± 18.2	844.0	172.9 ± 1.6	168.9
200	41.1 ± 0.3	41.6	86.7 ± 2.7	83.6	919.4 ± 29.0	857.6	177.0 ± 10.8	172.2

Table S35: Densities computed from MC and MD simulations (ρ^{MC} and ρ^{MD}), densities obtained from REFPROP[10] (ρ^{REFP}), isothermal compressibilities computed from MC simulations (β_T^{MC}), isothermal compressibilities obtained from REFPROP[10] (β_T^{REFP}), thermal expansion coefficients computed from MC simulations (α_P^{MC}), thermal expansion coefficients obtained from REFPROP[10] (α_P^{REFP}), Joule Thomson coefficients computed from MC simulations (μ_{JT}^{MC}), and Joule Thomson coefficients obtained from REFPROP[10] (μ_{JT}^{REFP}) of pure CO_2 at 273 K and pressures ranging from 20 bar to 200 bar.

P/ [bar]	ρ^{MC}/ [kg/m^3]	ρ^{MD}/ [kg/m^3]	ρ^{REFP}/ [kg/m^3]	β_T^{MC}/ [10^{-4}/bar]	β_T^{REFP}/ [10^{-4}/bar]	α_P^{MC}/ [10^{-3}/K]	α_P^{REFP}/ [10^{-3}/K]	μ_{JT}^{MC}/ [10^{-3} K/bar]	μ_{JT}^{REFP}/ [10^{-3} K/bar]
20	44.7	45.8 ± 0.1	45.7	590.1 ± 4.9	603.0	5.9	6.3	1276.2 ± 28.2	1399.6
40	936.7 ± 1.9	934.8 ± 0.6	933.1	7.2 ± 0.3	9.4	6.1 ± 0.3	7.1	29.3 ± 3.1	39.8
60	948.5 ± 1.2	947.2 ± 0.6	949.1	6.6 ± 0.1	7.7	5.9 ± 0.1	6.2	26.3 ± 1.5	30.7
80	960.7 ± 2.1	960.2 ± 1.3	962.7	5.7 ± 0.4	6.6	5.3 ± 0.3	5.6	19.7 ± 3.5	23.9
100	970.3 ± 1.2	971.2 ± 0.7	974.8	5.0 ± 0.1	5.8	4.8 ± 0.1	5.1	14.4 ± 0.8	18.6
120	980.3 ± 1.8	979.6 ± 0.8	985.6	4.6 ± 0.3	5.2	4.6 ± 0.3	4.7	11.5 ± 3.9	14.2
140	990.1 ± 1.0	988.6 ± 1.3	995.5	4.1 ± 0.1	4.8	4.2 ± 0.1	4.5	7.3 ± 1.8	10.5
160	997.2 ± 1.5	996.2 ± 0.2	1004.7	4.0 ± 0.1	4.4	4.1 ± 0.1	4.2	6.3 ± 1.6	7.3
180	1005.0 ± 0.8	1004.5 ± 1.0	1013.1	3.8 ± 0.1	4.0	4.1 ± 0.1	4.0	5.3 ± 1.4	4.6
200	1012.1 ± 0.8	1012.2 ± 0.6	1021.1	3.4 ± 0.1	3.8	3.7 ± 0.1	3.8	1.1 ± 2.0	2.2

Table S36: Heat capacities at constant volume computed from MC simulations (c_V^{MC}), heat capacities at constant volume obtained from REFPROP[10] (c_V^{REFP}), heat capacities at constant pressure computed from MC simulations (c_P^{MC}), heat capacities at constant pressure obtained from REFPROP[10] (c_P^{REFP}), speed of sound computed from MC simulations (c^{MC}), speed of sound obtained from REFPROP[10] (c^{REFP}), viscosities computed from MD simulations (η^{MD}), and viscosities obtained from REFPROP[10] (η^{REFP}) of pure CO_2 at 273 K and pressures ranging from 20 bar to 200 bar.

P/ [bar]	c_V^{MC}/ [J/mol K]	c_V^{REFP}/ [J/mol K]	c_P^{MC}/ [J/mol K]	c_P^{REFP}/ [J/mol K]	c^{MC}/ [m/s]	c^{REFP}/ [m/s]	η^{MD}/ [μPa s]	η^{REFP}/ [μPa s]
20	31.2	31.7	46.9 ± 0.2	48.8	238.8 ± 1.1	236.5	13.1 ± 1.9	13.9
40	40.0 ± 0.2	41.4	107.8 ± 2.6	109.6	633.5 ± 16.2	549.6	104.6 ± 8.7	101.0
60	40.0 ± 0.2	41.0	106.6 ± 1.8	103.4	651.1 ± 7.4	586.4	112.6 ± 3.5	105.8
80	40.0 ± 0.1	40.9	101.7 ± 2.7	99.1	684.2 ± 25.0	616.2	120.6 ± 3.1	110.2
100	40.1 ± 0.1	40.8	97.2 ± 0.7	95.8	707.0 ± 6.7	642.0	117.9 ± 5.2	114.2
120	40.1 ± 0.2	40.8	96.1 ± 4.1	93.2	730.8 ± 27.3	665.1	128.6 ± 9.1	118.0
140	40.0 ± 0.2	40.8	92.4 ± 1.6	91.1	751.7 ± 14.0	686.2	132.3 ± 6.4	121.6
160	40.3 ± 0.3	40.8	92.7 ± 1.8	89.3	762.9 ± 14.9	705.8	127.1 ± 5.5	125.0
180	40.2 ± 0.2	40.8	93.1 ± 1.5	87.8	782.8 ± 11.5	724.1	131.0 ± 4.7	129.4
200	40.4 ± 0.2	40.9	89.2 ± 2.0	86.5	800.1 ± 18.6	741.5	131.8 ± 8.2	131.6

Table S37: Densities computed from MC and MD simulations (ρ^{MC} and ρ^{MD}), densities obtained from REFPROP[10] (ρ^{REFP}), isothermal compressibilities computed from MC simulations (β_T^{MC}), isothermal compressibilities obtained from REFPROP[10] (β_T^{REFP}), thermal expansion coefficients computed from MC simulations (α_P^{MC}), thermal expansion coefficients obtained from REFPROP[10] (α_P^{REFP}), Joule Thomson coefficients computed from MC simulations ($\mu_{\mathrm{JT}}^{\mathrm{MC}}$), and Joule Thomson coefficients obtained from REFPROP[10] ($\mu_{\mathrm{JT}}^{\mathrm{REFP}}$) of pure CO_2 at 293 K and pressures ranging from 20 bar to 200 bar.

P/ [bar]	ρ^{MC}/ [kg/m^3]	ρ^{MD}/ [kg/m^3]	ρ^{REFP}/ [kg/m^3]	β_T^{MC}/ [10^{-4}/bar]	β_T^{REFP}/ [10^{-4}/bar]	α_P^{MC}/ [10^{-3}/K]	α_P^{REFP}/ [10^{-3}/K]	$\mu_{\mathrm{JT}}^{\mathrm{MC}}$/ [$10^{-3}$ K/bar]	$\mu_{\mathrm{JT}}^{\mathrm{REFP}}$/ [$10^{-3}$ K/bar]
20	40.2	40.9 ± 0.1	40.8	564.4 ± 3.6	571.2	4.9	5.1	1063.5 ± 22.3	1154.2
40	94.0 ± 0.1	96.0 ± 0.5	97.6	350.4 ± 4.6	366.4	8.3 ± 0.1	9.2	1091.9 ± 23.9	1157.4
60	802.0 ± 7.6	798.7 ± 4.1	784.7	29.4 ± 7.3	39.5	14.9 ± 2.8	17.5	111.7 ± 30.4	135.1
80	833.8 ± 2.1	834.8 ± 1.8	829.1	17.4 ± 1.6	20.3	10.3 ± 0.7	10.7	78.6 ± 8.9	87.4
100	860.2 ± 2.1	859.6 ± 1.3	857.4	11.8 ± 0.4	14.2	7.8 ± 0.3	8.3	55.9 ± 4.2	64.5
120	878.1 ± 1.8	877.4 ± 1.9	879.0	9.8 ± 0.6	11.1	6.9 ± 0.4	7.0	46.2 ± 5.6	49.9
140	892.5 ± 1.9	893.9 ± 1.0	896.8	8.4 ± 0.5	9.1	6.2 ± 0.3	6.2	38.0 ± 3.7	39.6
160	907.3 ± 1.4	906.7 ± 1.7	912.1	7.2 ± 0.4	7.8	5.6 ± 0.2	5.6	30.2 ± 3.4	31.8
180	917.9 ± 1.1	918.0 ± 1.0	925.6	6.3 ± 0.4	6.9	5.1 ± 0.3	5.1	23.9 ± 4.9	25.5
200	930.7 ± 1.9	929.5 ± 1.6	937.7	5.4 ± 0.2	6.1	4.6 ± 0.2	4.8	17.6 ± 2.5	20.4

Table S38: Heat capacities at constant volume computed from MC simulations (c_V^{MC}), heat capacities at constant volume obtained from REFPROP[10] (c_V^{REFP}), heat capacities at constant pressure computed from MC simulations (c_P^{MC}), heat capacities at constant pressure obtained from REFPROP[10] (c_P^{REFP}), speed of sound computed from MC simulations (c^{MC}), speed of sound obtained from REFPROP[10] (c^{REFP}), viscosities computed from MD simulations (η^{MD}), and viscosities obtained from REFPROP[10] (η^{REFP}) of pure CO_2 at 293 K and pressures ranging from 20 bar to 200 bar.

P/ [bar]	c_V^{MC}/ [J/mol K]	c_V^{REFP}/ [J/mol K]	c_P^{MC}/ [J/mol K]	c_P^{REFP}/ [J/mol K]	c^{MC}/ [m/s]	c^{REFP}/ [m/s]	η^{MD}/ [μPa s]	η^{REFP}/ [μPa s]
20	31.1	31.3	44.7 ± 0.1	45.6	251.8 ± 0.8	249.9	13.4 ± 2.2	15.0
40	34.3	35.6	61.3 ± 0.3	66.3	232.7 ± 1.6	228.0	15.1 ± 1.6	15.8
60	40.1 ± 0.2	43.8	165.8 ± 19.0	171.7	418.9 ± 57.4	355.7	72.9 ± 4.8	68.0
80	40.1 ± 0.1	41.8	135.4 ± 5.4	130.4	482.4 ± 24.5	430.8	80.1 ± 5.4	76.0
100	39.7	41.1	118.0 ± 3.4	115.1	540.6 ± 12.8	480.2	92.6 ± 4.4	81.7
120	39.8 ± 0.2	40.8	112.4 ± 4.0	106.6	572.4 ± 21.1	518.5	96.0 ± 3.0	86.5
140	39.7 ± 0.2	40.6	106.1 ± 2.2	100.9	596.7 ± 17.4	550.6	95.7 ± 3.3	90.7
160	39.7 ± 0.2	40.5	101.7 ± 2.3	96.9	628.0 ± 17.4	578.5	93.7 ± 4.3	94.6
180	39.7 ± 0.1	40.5	97.3 ± 3.6	93.8	651.0 ± 26.0	603.4	105.4 ± 6.0	98.2
200	39.8 ± 0.2	40.4	94.2 ± 2.1	91.3	684.6 ± 14.7	626.2	110.4 ± 1.3	101.5

Table S39: Densities computed from MC and MD simulations (ρ^{MC} and ρ^{MD}), densities obtained from REFPROP[10] (ρ^{REFP}), isothermal compressibilities computed from MC simulations (β_T^{MC}), isothermal compressibilities obtained from REFPROP[10] (β_T^{REFP}), thermal expansion coefficients computed from MC simulations (α_P^{MC}), thermal expansion coefficients obtained from REFPROP[10] (α_P^{REFP}), Joule Thomson coefficients computed from MC simulations (μ_{JT}^{MC}), and Joule Thomson coefficients obtained from REFPROP[10] (μ_{JT}^{REFP}) of pure CO_2 at 313 K and pressures ranging from 20 bar to 200 bar.

P/ [bar]	ρ^{MC}/ [kg/m^3]	ρ^{MD}/ [kg/m^3]	ρ^{REFP}/ [kg/m^3]	β_T^{MC}/ [10^{-4}/bar]	β_T^{REFP}/ [10^{-4}/bar]	α_P^{MC}/ [10^{-3}/K]	α_P^{REFP}/ [10^{-3}/K]	μ_{JT}^{MC}/ [10^{-3} K/bar]	μ_{JT}^{REFP}/ [10^{-3} K/bar]
20	36.7	37.4 ± 0.1	37.2	547.6 ± 1.3	552.5	4.2	4.3	896.2 ± 7.8	970.1
40	81.7	83.3 ± 0.1	83.8	311.7 ± 0.8	320.1	6.0	6.4	901.9 ± 4.5	964.8
60	142.9 ± 0.3	147.3 ± 1.3	149.5	261.3 ± 1.7	275.8	10.1 ± 0.1	11.4	901.6 ± 5.9	932.0
80	252.2 ± 1.3	289.6 ± 5.5	279.5	363.8 ± 7.4	414.3	30.4 ± 0.7	38.5	815.2 ± 4.7	783.6
100	636.2 ± 11.5	640.7 ± 1.4	631.7	113.2 ± 9.0	116.6	34.1 ± 2.1	32.7	246.6 ± 7.6	262.2
120	722.9 ± 7.4	720.6 ± 2.4	719.4	36.9 ± 1.6	39.6	15.2 ± 0.5	14.9	141.7 ± 3.4	149.7
140	765.2 ± 2.6	764.8 ± 0.9	764.5	21.7 ± 0.4	23.9	10.5 ± 0.2	10.5	99.4 ± 1.2	105.7
160	794.2 ± 2.9	793.0 ± 0.9	795.9	15.9 ± 0.4	17.2	8.5 ± 0.2	8.4	77.1 ± 1.6	80.5
180	814.7 ± 1.8	813.8 ± 1.4	820.4	12.5 ± 0.2	13.4	7.2 ± 0.1	7.1	61.5 ± 0.9	63.6
200	832.8 ± 1.3	833.2 ± 0.9	840.6	10.4 ± 0.2	11.1	6.4 ± 0.1	6.3	50.1 ± 1.1	51.3

Table S40: Heat capacities at constant volume computed from MC simulations (c_V^{MC}), heat capacities at constant volume obtained from REFPROP[10] (c_V^{REFP}), heat capacities at constant pressure computed from MC simulations (c_P^{MC}), heat capacities at constant pressure obtained from REFPROP[10] (c_P^{REFP}), speed of sound computed from MC simulations (c^{MC}), speed of sound obtained from REFPROP[10] (c^{REFP}), viscosities computed from MD simulations (η^{MD}), and viscosities obtained from REFPROP[10] (η^{REFP}) of pure CO_2 at 313 K and pressures ranging from 20 bar to 200 bar.

P/ [bar]	c_V^{MC}/ [J/mol K]	c_V^{REFP}/ [J/mol K]	c_P^{MC}/ [J/mol K]	c_P^{REFP}/ [J/mol K]	c^{MC}/ [m/s]	c^{REFP}/ [m/s]	η^{MD}/ [μPa s]	η^{REFP}/ [μPa s]
20	31.4	31.4	43.7	44.1	263.2 ± 0.2	261.5	16.3 ± 1.9	15.9
40	33.4	33.9	53.0 ± 0.1	55.2	249.8 ± 0.1	246.2	14.4 ± 1.1	16.5
60	36.3 ± 0.2	37.7	74.2 ± 0.3	80.9	234.3 ± 0.9	228.3	16.9 ± 1.5	17.9
80	41.1 ± 0.6	45.6	182.6 ± 3.4	222.3	220.2 ± 1.8	205.2	20.2 ± 1.3	22.0
100	41.4 ± 0.1	45.2	268.4 ± 11.3	245.8	299.0 ± 4.5	271.6	50.1 ± 3.7	48.2
120	40.2 ± 0.2	42.2	161.4 ± 3.0	150.2	388.0 ± 4.3	353.4	58.7 ± 5.2	58.7
140	39.8 ± 0.2	41.3	131.7 ± 1.6	124.7	447.1 ± 3.1	406.5	66.5 ± 0.4	65.3
160	39.6 ± 0.3	40.8	118.5 ± 1.3	112.1	488.1 ± 3.6	448.2	70.3 ± 1.4	70.3
180	39.5 ± 0.1	40.6	110.2 ± 1.0	104.4	522.8 ± 1.7	483.2	75.2 ± 1.6	74.6
200	39.5 ± 0.1	40.4	104.4 ± 0.8	99.1	553.3 ± 3.1	513.6	76.3 ± 1.1	78.5

S14.2 Data of thermodynamic and transport properties of N_2

Table S41: Densities computed from MC and MD simulations (ρ^{MC} and ρ^{MD}), densities obtained from REFPROP[10] (ρ^{REFP}), isothermal compressibilities computed from MC simulations (β_T^{MC}), isothermal compressibilities obtained from REFPROP[10] (β_T^{REFP}), thermal expansion coefficients computed from MC simulations (α_P^{MC}), thermal expansion coefficients obtained from REFPROP[10] (α_P^{REFP}), Joule Thomson coefficients computed from MC simulations ($\mu_{\mathrm{JT}}^{\mathrm{MC}}$), and Joule Thomson coefficients obtained from REFPROP[10] ($\mu_{\mathrm{JT}}^{\mathrm{REFP}}$) of pure N_2 at 253 K and pressures ranging from 20 bar to 200 bar.

P/ [bar]	ρ^{MC}/ [kg/m^3]	ρ^{MD}/ [kg/m^3]	ρ^{REFP}/ [kg/m^3]	β_T^{MC}/ [10^{-4}/bar]	β_T^{REFP}/ [10^{-4}/bar]	α_P^{MC}/ [10^{-3}/K]	α_P^{REFP}/ [10^{-3}/K]	$\mu_{\mathrm{JT}}^{\mathrm{MC}}$/ [$10^{-3}$ K/bar]	$\mu_{\mathrm{JT}}^{\mathrm{REFP}}$/ [$10^{-3}$ K/bar]
20	26.9	27.2	27.0	504.1 ± 2.2	506.2	4.3	4.3	263.5 ± 16.0	282.6
40	54.2	55.0 ± 0.1	54.6	254.7 ± 0.6	254.8	4.6	4.6	257.2 ± 4.9	261.1
60	81.9	83.0 ± 0.1	82.6	168.9 ± 0.8	169.8	4.8	4.9	230.3 ± 6.3	238.2
80	109.6	111.0 ± 0.2	110.6	125.8 ± 0.5	126.4	5.1	5.1	210.2 ± 3.9	214.7
100	137.0	138.0 ± 0.1	138.3	99.4 ± 0.7	99.5	5.3	5.3	189.7 ± 6.0	191.2
120	163.8 ± 0.1	164.8 ± 0.2	165.5	81.0 ± 1.2	81.0	5.4 ± 0.1	5.4	168.8 ± 9.2	168.2
140	189.9 ± 0.1	190.8 ± 0.2	191.9	67.0 ± 0.9	67.3	5.4 ± 0.1	5.5	145.2 ± 7.6	146.4
160	214.8 ± 0.2	215.7 ± 0.2	217.1	56.6 ± 0.9	56.8	5.4 ± 0.1	5.5	125.7 ± 7.7	126.1
180	238.4 ± 0.2	239.1 ± 0.2	241.1	48.2 ± 0.3	48.4	5.4	5.4	106.8 ± 2.5	107.5
200	260.7 ± 0.2	261.3 ± 0.2	263.8	41.8 ± 0.4	41.7	5.3 ± 0.1	5.3	92.8 ± 4.4	90.7

Table S42: Heat capacities at constant volume computed from MC simulations (c_V^{MC}), heat capacities at constant volume obtained from REFPROP[10] (c_V^{REFP}), heat capacities at constant pressure computed from MC simulations (c_P^{MC}), heat capacities at constant pressure obtained from REFPROP[10] (c_P^{REFP}), speed of sound computed from MC simulations (c^{MC}), speed of sound obtained from REFPROP[10] (c^{REFP}), viscosities computed from MD simulations (η^{MD}), and viscosities obtained from REFPROP[10] (η^{REFP}) of pure N_2 at 253 K and pressures ranging from 20 bar to 200 bar.

P/ [bar]	c_V^{MC}/ [J/mol K]	c_V^{REFP}/ [J/mol K]	c_P^{MC}/ [J/mol K]	c_P^{REFP}/ [J/mol K]	c^{MC}/ [m/s]	c^{REFP}/ [m/s]	η^{MD}/ [μPa s]	η^{REFP}/ [μPa s]
20	21.0	21.0	30.4	30.5	327.4 ± 0.7	326.0	16.7 ± 1.9	16.0
40	21.1	21.2	31.9	32.0	330.5 ± 0.4	329.1	15.3 ± 2.4	16.5
60	21.3	21.4	33.3 ± 0.1	33.4	336.0 ± 0.8	333.8	15.8 ± 1.0	17.0
80	21.5	21.6	34.7 ± 0.1	34.9	342.4 ± 0.8	340.1	16.5 ± 1.0	17.7
100	21.6	21.7	36.1 ± 0.1	36.2	350.3 ± 1.4	347.9	19.1 ± 1.4	18.5
120	21.7	21.9	37.4 ± 0.2	37.4	360.0 ± 2.8	357.3	18.4 ± 1.3	19.3
140	21.8	22.0	38.3 ± 0.2	38.5	371.4 ± 2.8	368.0	19.3 ± 2.2	20.2
160	21.9	22.1	39.3 ± 0.3	39.4	383.7 ± 3.5	379.9	22.3 ± 2.8	21.1
180	22.0	22.2	39.9 ± 0.1	40.1	397.1 ± 1.2	392.8	21.7 ± 1.5	22.1
200	22.1	22.3	40.7 ± 0.2	40.6	410.6 ± 2.5	406.5	23.3 ± 1.3	23.1

Table S43: Densities computed from MC and MD simulations (ρ^{MC} and ρ^{MD}), densities obtained from REFPROP[10] (ρ^{REFP}), isothermal compressibilities computed from MC simulations (β_T^{MC}), isothermal compressibilities obtained from REFPROP[10] (β_T^{REFP}), thermal expansion coefficients computed from MC simulations (α_P^{MC}), thermal expansion coefficients obtained from REFPROP[10] (α_P^{REFP}), Joule Thomson coefficients computed from MC simulations (μ_{JT}^{MC}), and Joule Thomson coefficients obtained from REFPROP[10] (μ_{JT}^{REFP}) of pure N_2 at 273 K and pressures ranging from 20 bar to 200 bar.

P/ [bar]	ρ^{MC}/ [kg/m^3]	ρ^{MD}/ [kg/m^3]	ρ^{REFP}/ [kg/m^3]	β_T^{MC}/ [10^{-4}/bar]	β_T^{REFP}/ [10^{-4}/bar]	α_P^{MC}/ [10^{-3}/K]	α_P^{REFP}/ [10^{-3}/K]	μ_{JT}^{MC}/ [10^{-3} K/bar]	μ_{JT}^{REFP}/ [10^{-3} K/bar]
20	24.8	25.1	24.9	503.1 ± 1.0	503.5	3.9	3.9	235.8 ± 9.3	242.2
40	49.8	50.4 ± 0.1	50.1	251.9 ± 1.3	252.3	4.1	4.1	219.2 ± 10.6	223.8
60	74.8	75.7 ± 0.1	75.3	166.1 ± 0.8	167.6	4.3	4.3	191.7 ± 7.4	204.7
80	99.7 ± 0.1	100.8 ± 0.1	100.5	124.8 ± 0.3	124.6	4.5	4.5	185.8 ± 2.4	185.4
100	124.3	125.2 ± 0.1	125.3	98.0 ± 0.5	98.2	4.6	4.6	164.4 ± 4.4	166.3
120	148.2	149.3 ± 0.2	149.7	79.9 ± 0.5	80.2	4.7	4.7	146.1 ± 5.3	147.7
140	171.5 ± 0.1	172.6 ± 0.1	173.3	66.7 ± 0.8	67.0	4.7 ± 0.1	4.7	126.8 ± 7.1	129.9
160	194.0 ± 0.1	194.9 ± 0.2	196.1	56.9 ± 0.4	57.0	4.7	4.7	114.2 ± 3.8	113.2
180	215.5 ± 0.1	216.4 ± 0.1	217.9	48.8 ± 0.4	49.0	4.7	4.7	97.0 ± 3.3	97.7
200	236.1	236.8 ± 0.3	238.8	42.4 ± 0.7	42.6	4.7 ± 0.1	4.7	83.3 ± 7.1	83.5

Table S44: Heat capacities at constant volume computed from MC simulations (c_V^{MC}), heat capacities at constant volume obtained from REFPROP[10] (c_V^{REFP}), heat capacities at constant pressure computed from MC simulations (c_P^{MC}), heat capacities at constant pressure obtained from REFPROP[10] (c_P^{REFP}), speed of sound computed from MC simulations (c^{MC}), speed of sound obtained from REFPROP[10] (c^{REFP}), viscosities computed from MD simulations (η^{MD}), and viscosities obtained from REFPROP[10] (η^{REFP}) of pure N_2 at 273 K and pressures ranging from 20 bar to 200 bar.

P/ [bar]	c_V^{MC}/ [J/mol K]	c_V^{REFP}/ [J/mol K]	c_P^{MC}/ [J/mol K]	c_P^{REFP}/ [J/mol K]	c^{MC}/ [m/s]	c^{REFP}/ [m/s]	η^{MD}/ [μPa s]	η^{REFP}/ [μPa s]
20	20.9	21.0	30.2	30.3	340.4 ± 0.4	339.4	16.2 ± 1.5	16.9
40	21.1	21.1	31.4 ± 0.1	31.4	344.7 ± 1.0	343.2	17.3 ± 2.1	17.4
60	21.2	21.3	32.4 ± 0.1	32.6	350.8 ± 0.9	348.2	16.4 ± 1.5	17.8
80	21.3	21.4	33.7	33.7	356.2 ± 0.4	354.4	16.9 ± 1.8	18.4
100	21.5	21.6	34.7 ± 0.1	34.8	364.4 ± 1.0	361.9	18.1 ± 1.6	19.1
120	21.6	21.7	35.7 ± 0.1	35.7	373.6 ± 1.4	370.4	18.6 ± 1.1	19.8
140	21.7	21.8	36.4 ± 0.2	36.6	383.6 ± 2.5	380.1	18.6 ± 0.9	20.5
160	21.8	21.9	37.3 ± 0.1	37.4	394.2 ± 1.6	390.6	20.3 ± 0.9	21.3
180	21.8	22.0	37.9 ± 0.1	38.0	406.3 ± 1.7	402.0	21.6 ± 1.2	22.1
200	21.9	22.1	38.4 ± 0.3	38.5	418.3 ± 3.9	414.0	21.9 ± 0.4	23.0

Table S45: Densities computed from MC and MD simulations (ρ^{MC} and ρ^{MD}), densities obtained from REFPROP[10] (ρ^{REFP}), isothermal compressibilities computed from MC simulations (β_T^{MC}), isothermal compressibilities obtained from REFPROP[10] (β_T^{REFP}), thermal expansion coefficients computed from MC simulations (α_P^{MC}), thermal expansion coefficients obtained from REFPROP[10] (α_P^{REFP}), Joule Thomson coefficients computed from MC simulations ($\mu_{\mathrm{JT}}^{\mathrm{MC}}$), and Joule Thomson coefficients obtained from REFPROP[10] ($\mu_{\mathrm{JT}}^{\mathrm{REFP}}$) of pure N_2 at 293 K and pressures ranging from 20 bar to 200 bar.

P/ [bar]	ρ^{MC}/ [kg/m^3]	ρ^{MD}/ [kg/m^3]	ρ^{REFP}/ [kg/m^3]	β_T^{MC}/ [10^{-4}/bar]	β_T^{REFP}/ [10^{-4}/bar]	α_P^{MC}/ [10^{-3}/K]	α_P^{REFP}/ [10^{-3}/K]	$\mu_{\mathrm{JT}}^{\mathrm{MC}}$/ [$10^{-3}$ K/bar]	$\mu_{\mathrm{JT}}^{\mathrm{REFP}}$/ [$10^{-3}$ K/bar]
20	23.0	23.3	23.1	500.4 ± 1.8	501.5	3.6	3.6	196.0 ± 15.2	208.4
40	46.0	46.6	46.3	249.5 ± 1.4	250.5	3.7	3.7	181.8 ± 12.7	192.5
60	69.0	69.7 ± 0.1	69.4	165.9 ± 0.7	166.1	3.9	3.9	173.9 ± 6.2	176.3
80	91.6	92.6 ± 0.1	92.3	123.2 ± 0.9	123.4	4.0	4.0	157.7 ± 8.3	160.1
100	114.0	114.8 ± 0.2	114.9	96.6 ± 0.6	97.3	4.1	4.1	137.9 ± 5.3	144.2
120	135.8	136.8 ± 0.1	137.0	79.1 ± 0.6	79.7	4.1	4.2	124.4 ± 6.4	128.7
140	157.0	157.9 ± 0.1	158.5	66.6 ± 0.3	66.8	4.2	4.2	112.1 ± 2.5	114.0
160	177.6 ± 0.1	178.5 ± 0.1	179.4	56.7 ± 0.5	57.0	4.2	4.2	98.2 ± 4.9	100.0
180	197.3 ± 0.1	198.1 ± 0.1	199.4	49.4 ± 0.5	49.3	4.2	4.2	89.1 ± 5.1	86.9
200	216.3 ± 0.2	217.0 ± 0.1	218.7	42.8 ± 0.2	43.1	4.1	4.1	72.5 ± 1.9	74.8

Table S46: Heat capacities at constant volume computed from MC simulations (c_V^{MC}), heat capacities at constant volume obtained from REFPROP[10] (c_V^{REFP}), heat capacities at constant pressure computed from MC simulations (c_P^{MC}), heat capacities at constant pressure obtained from REFPROP[10] (c_P^{REFP}), speed of sound computed from MC simulations (c^{MC}), speed of sound obtained from REFPROP[10] (c^{REFP}), viscosities computed from MD simulations (η^{MD}), and viscosities obtained from REFPROP[10] (η^{REFP}) of pure N_2 at 293 K and pressures ranging from 20 bar to 200 bar.

P/ [bar]	c_V^{MC}/ [J/mol K]	c_V^{REFP}/ [J/mol K]	c_P^{MC}/ [J/mol K]	c_P^{REFP}/ [J/mol K]	c^{MC}/ [m/s]	c^{REFP}/ [m/s]	η^{MD}/ [μPa s]	η^{REFP}/ [μPa s]
20	20.9	21.0	30.1	30.1	353.5 ± 0.7	352.2	16.4 ± 1.7	17.9
40	21.0	21.1	31.0 ± 0.1	31.0	358.1 ± 1.1	356.4	16.1 ± 1.0	18.2
60	21.1	21.2	31.9 ± 0.1	32.0	363.4 ± 0.8	361.7	17.6 ± 1.1	18.7
80	21.3	21.3	32.8 ± 0.1	32.9	369.9 ± 1.4	367.9	17.7 ± 1.6	19.2
100	21.4	21.5	33.6 ± 0.1	33.8	378.2 ± 1.2	375.1	17.6 ± 1.0	19.7
120	21.4	21.6	34.4 ± 0.1	34.6	386.5 ± 1.7	383.1	21.0 ± 1.2	20.3
140	21.5	21.7	35.2	35.3	395.3 ± 0.9	392.0	19.7 ± 1.4	21.0
160	21.6	21.8	35.8 ± 0.1	35.9	405.7 ± 1.9	401.7	20.0 ± 0.8	21.7
180	21.7	21.8	36.5 ± 0.2	36.5	415.5 ± 2.4	411.9	23.1 ± 3.4	22.4
200	21.8	21.9	36.8 ± 0.1	36.9	427.5 ± 1.0	422.7	20.7 ± 1.0	23.1

Table S47: Densities computed from MC and MD simulations (ρ^{MC} and ρ^{MD}), densities obtained from REFPROP[10] (ρ^{REFP}), isothermal compressibilities computed from MC simulations (β_T^{MC}), isothermal compressibilities obtained from REFPROP[10] (β_T^{REFP}), thermal expansion coefficients computed from MC simulations (α_P^{MC}), thermal expansion coefficients obtained from REFPROP[10] (α_P^{REFP}), Joule Thomson coefficients computed from MC simulations ($\mu_{\mathrm{JT}}^{\mathrm{MC}}$), and Joule Thomson coefficients obtained from REFPROP[10] ($\mu_{\mathrm{JT}}^{\mathrm{REFP}}$) of pure N_2 at 313 K and pressures ranging from 20 bar to 200 bar.

P/ [bar]	ρ^{MC}/ [kg/m^3]	ρ^{MD}/ [kg/m^3]	ρ^{REFP}/ [kg/m^3]	β_T^{MC}/ [10^{-4}/bar]	β_T^{REFP}/ [10^{-4}/bar]	α_P^{MC}/ [10^{-3}/K]	α_P^{REFP}/ [10^{-3}/K]	$\mu_{\mathrm{JT}}^{\mathrm{MC}}$/ [$10^{-3}$ K/bar]	$\mu_{\mathrm{JT}}^{\mathrm{REFP}}$/ [$10^{-3}$ K/bar]
20	21.5	21.7	21.5	500.0 ± 1.2	500.0	3.3	3.3	178.2 ± 11.8	179.7
40	42.8	43.4	43.1	248.7 ± 0.4	249.2	3.4	3.4	159.4 ± 4.5	165.8
60	64.0	64.8 ± 0.1	64.4	163.8 ± 0.7	165.0	3.5	3.5	139.4 ± 5.9	151.9
80	84.9 ± 0.1	85.9 ± 0.1	85.6	122.2 ± 0.2	122.5	3.6	3.6	135.8 ± 2.3	138.1
100	105.5	106.3 ± 0.1	106.3	96.4 ± 0.3	96.7	3.7	3.7	121.8 ± 3.7	124.6
120	125.6	126.4 ± 0.1	126.6	78.8 ± 0.4	79.3	3.7	3.7	108.0 ± 3.7	111.6
140	145.1	145.8 ± 0.2	146.4	66.3 ± 0.7	66.6	3.7	3.8	96.2 ± 7.2	99.1
160	164.1 ± 0.1	165.0 ± 0.1	165.7	57.1 ± 0.2	57.0	3.8	3.8	88.6 ± 2.0	87.2
180	182.4 ± 0.1	183.1 ± 0.1	184.2	49.4 ± 0.5	49.5	3.7	3.8	75.0 ± 5.6	76.1
200	199.9 ± 0.1	200.9 ± 0.3	202.1	43.2 ± 0.3	43.4	3.7	3.7	64.5 ± 4.2	65.6

Table S48: Heat capacities at constant volume computed from MC simulations (c_V^{MC}), heat capacities at constant volume obtained from REFPROP[10] (c_V^{REFP}), heat capacities at constant pressure computed from MC simulations (c_P^{MC}), heat capacities at constant pressure obtained from REFPROP[10] (c_P^{REFP}), speed of sound computed from MC simulations (c^{MC}), speed of sound obtained from REFPROP[10] (c^{REFP}), viscosities computed from MD simulations (η^{MD}), and viscosities obtained from REFPROP[10] (η^{REFP}) of pure N_2 at 313 K and pressures ranging from 20 bar to 200 bar.

P/ [bar]	c_V^{MC}/ [J/mol K]	c_V^{REFP}/ [J/mol K]	c_P^{MC}/ [J/mol K]	c_P^{REFP}/ [J/mol K]	c^{MC}/ [m/s]	c^{REFP}/ [m/s]	η^{MD}/ [μPa s]	η^{REFP}/ [μPa s]
20	20.9	20.9	29.9	30.0	365.4 ± 0.5	364.3	19.0 ± 3.0	18.7
40	21.0	21.1	30.7	30.8	370.5 ± 0.4	369.0	17.4 ± 1.9	19.1
60	21.1	21.2	31.4	31.5	376.9 ± 0.8	374.4	16.3 ± 1.0	19.5
80	21.2	21.3	32.3	32.3	382.8 ± 0.3	380.6	17.9 ± 0.9	19.9
100	21.3	21.4	32.9 ± 0.1	33.0	390.2 ± 0.7	387.6	18.4 ± 1.0	20.4
120	21.4	21.5	33.6 ± 0.1	33.7	398.5 ± 1.0	395.3	19.2 ± 1.5	20.9
140	21.4	21.6	34.2 ± 0.2	34.3	407.2 ± 2.2	403.7	19.9 ± 0.5	21.5
160	21.5	21.6	34.8	34.8	415.6 ± 0.8	412.6	20.6 ± 0.7	22.1
180	21.6	21.7	35.2 ± 0.2	35.3	425.9 ± 2.3	422.1	20.8 ± 1.3	22.7
200	21.6	21.8	35.7 ± 0.2	35.7	436.6 ± 1.9	432.1	22.2 ± 1.8	23.4

S14.3 Data of thermodynamic and transport properties of argon

Table S49: Densities computed from MC and MD simulations (ρ^{MC} and ρ^{MD}), densities obtained from REFPROP[10] (ρ^{REFP}), isothermal compressibilities computed from MC simulations (β_T^{MC}), isothermal compressibilities obtained from REFPROP[10] (β_T^{REFP}), thermal expansion coefficients computed from MC simulations (α_P^{MC}), thermal expansion coefficients obtained from REFPROP[10] (α_P^{REFP}), Joule Thomson coefficients computed from MC simulations ($\mu_{\text{JT}}^{\text{MC}}$), and Joule Thomson coefficients obtained from REFPROP[10] ($\mu_{\text{JT}}^{\text{REFP}}$) of pure Ar at 253 K and pressures ranging from 20 bar to 200 bar.

P/ [bar]	ρ^{MC}/ [kg/m^3]	ρ^{MD}/ [kg/m^3]	ρ^{REFP}/ [kg/m^3]	β_T^{MC}/ [10^{-4}/bar]	β_T^{REFP}/ [10^{-4}/bar]	α_P^{MC}/ [10^{-3}/K]	α_P^{REFP}/ [10^{-3}/K]	$\mu_{\text{JT}}^{\text{MC}}$/ [$10^{-3}$ K/bar]	$\mu_{\text{JT}}^{\text{REFP}}$/ [$10^{-3}$ K/bar]
20	38.7	78.8 ± 0.1	39.0	509.3 ± 1.5	512.5	4.3	4.4	410.5 ± 16.2	467.4
40	79.1	120.7 ± 0.2	79.8	260.1 ± 0.8	262.0	4.7	4.8	400.1 ± 8.7	437.9
60	121.0	163.7 ± 0.4	122.6	176.4 ± 0.6	177.8	5.1	5.2	378.0 ± 6.3	406.3
80	164.4 ± 0.1	207.1 ± 0.2	166.9	133.8 ± 0.9	135.0	5.5	5.7	351.5 ± 9.6	373.0
100	208.7 ± 0.1	251.5 ± 0.4	212.5	108.0 ± 0.5	108.4	5.9	6.1	326.4 ± 6.2	338.8
120	254.0 ± 0.1	296.0 ± 0.5	258.9	89.3 ± 0.8	89.9	6.2 ± 0.1	6.4	293.8 ± 7.8	304.4
140	299.3 ± 0.3	339.9 ± 0.6	305.4	75.2 ± 0.5	75.8	6.5 ± 0.1	6.7	262.0 ± 5.8	270.7
160	344.1 ± 0.2	382.7 ± 0.5	351.2	64.2 ± 0.7	64.5	6.6 ± 0.1	6.9	232.6 ± 5.8	238.4
180	387.9 ± 0.2	424.0 ± 0.4	395.7	55.4 ± 0.8	55.2	6.8 ± 0.1	6.9	206.6 ± 8.4	208.3

Table S50: Heat capacities at constant volume computed from MC simulations (c_V^{MC}), heat capacities at constant volume obtained from REFPROP[10] (c_V^{REFP}), heat capacities at constant pressure computed from MC simulations (c_P^{MC}), heat capacities at constant pressure obtained from REFPROP[10] (c_P^{REFP}), speed of sound computed from MC simulations (c^{MC}), speed of sound obtained from REFPROP[10] (c^{REFP}), viscosities computed from MD simulations (η^{MD}), and viscosities obtained from REFPROP[10] (η^{REFP}) of pure Ar at 253 K and pressures ranging from 20 bar to 200 bar.

P/ [bar]	c_V^{MC}/ [J/mol K]	c_V^{REFP}/ [J/mol K]	c_P^{MC}/ [J/mol K]	c_P^{REFP}/ [J/mol K]	c^{MC}/ [m/s]	c^{REFP}/ [m/s]	η^{MD}/ [μPa s]	η^{REFP}/ [μPa s]
20	12.6	12.7	22.1	22.3	298.0 ± 0.5	296.6	23.7 ± 3.6	20.8
40	12.8	12.9	23.6	24.0	299.4 ± 0.5	297.7	25.1 ± 1.3	21.5
60	13.0	13.2	25.3 ± 0.1	25.8	302.1 ± 0.6	300.0	26.5 ± 1.3	22.5
80	13.2	13.4	27.1 ± 0.1	27.8	305.7 ± 1.2	303.4	27.5 ± 2.0	23.6
100	13.3	13.6	29.0 ± 0.1	29.8	310.5 ± 1.0	308.3	30.1 ± 1.4	24.9
120	13.5	13.8	30.7 ± 0.2	31.7	317.1 ± 1.6	314.5	32.4 ± 1.4	26.3
140	13.6	13.9	32.4 ± 0.2	33.5	325.3 ± 1.5	322.4	32.3 ± 3.1	27.8
160	13.7	14.1	33.9 ± 0.2	35.1	334.5 ± 2.0	331.7	33.7 ± 3.0	29.4
180	13.8	14.2	35.3 ± 0.4	36.3	344.5 ± 3.2	342.3	36.3 ± 3.0	31.0

Table S51: Densities computed from MC and MD simulations (ρ^{MC} and ρ^{MD}), densities obtained from REFPROP[10] (ρ^{REFP}), isothermal compressibilities computed from MC simulations (β_T^{MC}), isothermal compressibilities obtained from REFPROP[10] (β_T^{REFP}), thermal expansion coefficients computed from MC simulations (α_P^{MC}), thermal expansion coefficients obtained from REFPROP[10] (α_P^{REFP}), Joule Thomson coefficients computed from MC simulations (μ_{JT}^{MC}), and Joule Thomson coefficients obtained from REFPROP[10] (μ_{JT}^{REFP}) of pure Ar at 273 K and pressures ranging from 20 bar to 200 bar.

P/ [bar]	ρ^{MC}/ [kg/m^3]	ρ^{MD}/ [kg/m^3]	ρ^{REFP}/ [kg/m^3]	β_T^{MC}/ [10^{-4}/bar]	β_T^{REFP}/ [10^{-4}/bar]	α_P^{MC}/ [10^{-3}/K]	α_P^{REFP}/ [10^{-3}/K]	μ_{JT}^{MC}/ [10^{-3} K/bar]	μ_{JT}^{REFP}/ [10^{-3} K/bar]
20	35.6	72.0 ± 0.1	35.8	507.4 ± 1.8	508.9	3.9	4.0	370.0 ± 19.4	408.1
40	72.3	109.4 ± 0.1	72.9	256.8 ± 1.4	258.3	4.2	4.3	350.1 ± 16.6	383.3
60	110.0	147.5 ± 0.2	111.2	173.1 ± 0.7	174.2	4.5	4.6	331.4 ± 7.5	357.4
80	148.4 ± 0.1	185.5 ± 0.2	150.3	130.0 ± 0.8	131.5	4.7	4.9	304.6 ± 7.7	330.6
100	187.5 ± 0.1	224.3 ± 0.1	190.1	104.2 ± 0.8	105.3	5.0	5.1	283.5 ± 8.0	303.4
120	226.7 ± 0.1	263.2 ± 0.4	230.3	86.4 ± 0.7	87.3	5.2	5.4	260.8 ± 8.0	276.2
140	266.0 ± 0.1	301.0 ± 0.4	270.4	73.5 ± 0.7	73.9	5.4 ± 0.1	5.5	240.3 ± 7.8	249.6
160	304.9 ± 0.2	338.6 ± 0.2	310.1	63.5 ± 0.5	63.4	5.6	5.7	219.1 ± 5.2	223.9
180	343.0 ± 0.1	374.7 ± 0.3	348.9	54.8 ± 0.4	54.9	5.6	5.8	195.0 ± 3.7	199.5

Table S52: Heat capacities at constant volume computed from MC simulations (c_V^{MC}), heat capacities at constant volume obtained from REFPROP[10] (c_V^{REFP}), heat capacities at constant pressure computed from MC simulations (c_P^{MC}), heat capacities at constant pressure obtained from REFPROP[10] (c_P^{REFP}), speed of sound computed from MC simulations (c^{MC}), speed of sound obtained from REFPROP[10] (c^{REFP}), viscosities computed from MD simulations (η^{MD}), and viscosities obtained from REFPROP[10] (η^{REFP}) of pure Ar at 273 K and pressures ranging from 20 bar to 200 bar.

P/ [bar]	c_V^{MC}/ [J/mol K]	c_V^{REFP}/ [J/mol K]	c_P^{MC}/ [J/mol K]	c_P^{REFP}/ [J/mol K]	c^{MC}/ [m/s]	c^{REFP}/ [m/s]	η^{MD}/ [μPa s]	η^{REFP}/ [μPa s]
20	12.6	12.7	21.9	22.0	310.0 ± 0.6	308.8	28.6 ± 2.9	22.0
40	12.8	12.8	23.1 ± 0.1	23.4	312.3 ± 1.0	310.7	28.3 ± 4.2	22.6
60	12.9	13.0	24.4 ± 0.1	24.8	315.2 ± 0.7	313.4	28.0 ± 2.0	23.5
80	13.0	13.2	25.6 ± 0.1	26.2	319.4 ± 1.0	317.0	29.3 ± 2.0	24.4
100	13.2	13.4	27.0 ± 0.1	27.7	324.1 ± 1.3	321.7	31.2 ± 2.3	25.4
120	13.3	13.5	28.3 ± 0.1	29.1	330.2 ± 1.6	327.4	29.8 ± 3.2	26.6
140	13.4	13.6	29.7 ± 0.2	30.4	336.5 ± 1.9	334.2	31.9 ± 5.5	27.8
160	13.5	13.8	30.9 ± 0.1	31.6	344.1 ± 1.5	342.1	32.9 ± 2.9	29.1
180	13.6	13.9	31.9 ± 0.1	32.7	353.5 ± 1.3	350.9	34.8 ± 4.7	30.4

Table S53: Densities computed from MC and MD simulations (ρ^{MC} and ρ^{MD}), densities obtained from REFPROP[10] (ρ^{REFP}), isothermal compressibilities computed from MC simulations (β_T^{MC}), isothermal compressibilities obtained from REFPROP[10] (β_T^{REFP}), thermal expansion coefficients computed from MC simulations (α_P^{MC}), thermal expansion coefficients obtained from REFPROP[10] (α_P^{REFP}), Joule Thomson coefficients computed from MC simulations ($\mu_{\mathrm{JT}}^{\mathrm{MC}}$), and Joule Thomson coefficients obtained from REFPROP[10] ($\mu_{\mathrm{JT}}^{\mathrm{REFP}}$) of pure Ar at 293 K and pressures ranging from 20 bar to 200 bar.

P/ [bar]	ρ^{MC}/ [kg/m^3]	ρ^{MD}/ [kg/m^3]	ρ^{REFP}/ [kg/m^3]	β_T^{MC}/ [10^{-4}/bar]	β_T^{REFP}/ [10^{-4}/bar]	α_P^{MC}/ [10^{-3}/K]	α_P^{REFP}/ [10^{-3}/K]	$\mu_{\mathrm{JT}}^{\mathrm{MC}}$/ [$10^{-3}$ K/bar]	$\mu_{\mathrm{JT}}^{\mathrm{REFP}}$/ [$10^{-3}$ K/bar]
20	33.1	66.4 ± 0.1	33.2	504.9 ± 0.8	506.3	3.6	3.6	322.4 ± 10.3	358.6
40	66.8	100.5 ± 0.1	67.3	254.6 ± 1.3	255.7	3.8	3.9	308.6 ± 15.6	337.4
60	101.0	134.8 ± 0.2	102.0	170.9 ± 0.5	171.7	4.0	4.1	294.2 ± 6.2	315.6
80	135.8	169.0 ± 0.1	137.2	128.0 ± 0.4	129.1	4.2	4.3	269.6 ± 4.0	293.4
100	170.7	203.3 ± 0.2	172.8	102.0 ± 0.3	103.2	4.3	4.5	248.6 ± 3.4	271.1
120	205.7 ± 0.1	237.8 ± 0.3	208.5	85.4 ± 0.7	85.5	4.5	4.6	239.6 ± 8.6	249.0
140	240.6 ± 0.1	271.8 ± 0.4	244.0	72.2 ± 0.5	72.5	4.7	4.8	217.3 ± 6.1	227.3
160	275.2 ± 0.2	304.9 ± 0.2	279.2	62.0 ± 0.6	62.5	4.7	4.9	195.3 ± 6.6	206.3
180	309.0 ± 0.2	338.1 ± 0.4	313.7	54.5 ± 0.4	54.4	4.8	4.9	181.4 ± 4.7	186.2

Table S54: Heat capacities at constant volume computed from MC simulations (c_V^{MC}), heat capacities at constant volume obtained from REFPROP[10] (c_V^{REFP}), heat capacities at constant pressure computed from MC simulations (c_P^{MC}), heat capacities at constant pressure obtained from REFPROP[10] (c_P^{REFP}), speed of sound computed from MC simulations (c^{MC}), speed of sound obtained from REFPROP[10] (c^{REFP}), viscosities computed from MD simulations (η^{MD}), and viscosities obtained from REFPROP[10] (η^{REFP}) of pure Ar at 293 K and pressures ranging from 20 bar to 200 bar.

P/ [bar]	c_V^{MC}/ [J/mol K]	c_V^{REFP}/ [J/mol K]	c_P^{MC}/ [J/mol K]	c_P^{REFP}/ [J/mol K]	c^{MC}/ [m/s]	c^{REFP}/ [m/s]	η^{MD}/ [μPa s]	η^{REFP}/ [μPa s]
20	12.6	12.6	21.7	21.8	321.6 ± 0.3	320.5	30.0 ± 1.4	23.2
40	12.7	12.8	22.7 ± 0.1	22.9	324.3 ± 0.9	322.8	27.2 ± 3.7	23.8
60	12.8	12.9	23.7	24.0	327.6 ± 0.5	325.8	27.2 ± 3.7	24.5
80	12.9	13.1	24.7	25.1	331.7 ± 0.5	329.6	29.5 ± 2.8	25.3
100	13.0	13.2	25.7	26.3	336.8 ± 0.5	334.2	30.6 ± 2.9	26.2
120	13.1	13.3	26.9 ± 0.1	27.4	341.6 ± 1.7	339.6	34.1 ± 3.7	27.1
140	13.2	13.4	27.8 ± 0.1	28.4	348.0 ± 1.5	345.8	32.8 ± 4.0	28.1
160	13.3	13.5	28.7 ± 0.1	29.4	355.4 ± 1.9	352.8	34.2 ± 2.8	29.2
180	13.4	13.6	29.7 ± 0.1	30.2	362.6 ± 1.4	360.5	34.9 ± 7.1	30.3

Table S55: Densities computed from MC and MD simulations (ρ^{MC} and ρ^{MD}), densities obtained from REFPROP[10] (ρ^{REFP}), isothermal compressibilities computed from MC simulations (β_T^{MC}), isothermal compressibilities obtained from REFPROP[10] (β_T^{REFP}), thermal expansion coefficients computed from MC simulations (α_P^{MC}), thermal expansion coefficients obtained from REFPROP[10] (α_P^{REFP}), Joule Thomson coefficients computed from MC simulations ($\mu_{\mathrm{JT}}^{\mathrm{MC}}$), and Joule Thomson coefficients obtained from REFPROP[10] ($\mu_{\mathrm{JT}}^{\mathrm{REFP}}$) of pure Ar at 313 K and pressures ranging from 20 bar to 200 bar.

P/ [bar]	ρ^{MC}/ [kg/m^3]	ρ^{MD}/ [kg/m^3]	ρ^{REFP}/ [kg/m^3]	β_T^{MC}/ [10^{-4}/bar]	β_T^{REFP}/ [10^{-4}/bar]	α_P^{MC}/ [10^{-3}/K]	α_P^{REFP}/ [10^{-3}/K]	$\mu_{\mathrm{JT}}^{\mathrm{MC}}$/ [$10^{-3}$ K/bar]	$\mu_{\mathrm{JT}}^{\mathrm{REFP}}$/ [$10^{-3}$ K/bar]
20	30.8	61.6 ± 0.1	31.0	504.1 ± 1.0	504.4	3.4	3.4	297.3 ± 12.9	316.5
40	62.1	93.0 ± 0.2	62.5	253.2 ± 0.7	253.8	3.5	3.5	277.0 ± 8.5	298.2
60	93.6	124.3 ± 0.2	94.4	169.5 ± 0.6	169.8	3.7	3.7	262.5 ± 7.7	279.7
80	125.4	155.2 ± 0.1	126.6	127.2 ± 0.3	127.5	3.8	3.8	246.8 ± 3.7	260.9
100	157.2	186.8 ± 0.1	158.9	101.2 ± 0.8	101.7	3.9	4.0	227.7 ± 10.2	242.3
120	188.9 ± 0.1	217.8 ± 0.4	191.1	84.3 ± 0.4	84.2	4.0	4.1	215.3 ± 5.3	223.8
140	220.5 ± 0.1	248.6 ± 0.3	223.2	71.0 ± 0.5	71.5	4.1	4.2	192.7 ± 6.3	205.7
160	251.7 ± 0.1	278.7 ± 0.3	254.9	61.6 ± 0.6	61.7	4.2	4.3	179.8 ± 7.2	188.1
180	282.4 ± 0.2	308.1 ± 0.4	286.1	53.9 ± 0.5	54.0	4.2	4.3	164.5 ± 6.9	171.3

Table S56: Heat capacities at constant volume computed from MC simulations (c_V^{MC}), heat capacities at constant volume obtained from REFPROP[10] (c_V^{REFP}), heat capacities at constant pressure computed from MC simulations (c_P^{MC}), heat capacities at constant pressure obtained from REFPROP[10] (c_P^{REFP}), speed of sound computed from MC simulations (c^{MC}), speed of sound obtained from REFPROP[10] (c^{REFP}), viscosities computed from MD simulations (η^{MD}), and viscosities obtained from REFPROP[10] (η^{REFP}) of pure Ar at 313 K and pressures ranging from 20 bar to 200 bar.

P/ [bar]	c_V^{MC}/ [J/mol K]	c_V^{REFP}/ [J/mol K]	c_P^{MC}/ [J/mol K]	c_P^{REFP}/ [J/mol K]	c^{MC}/ [m/s]	c^{REFP}/ [m/s]	η^{MD}/ [μPa s]	η^{REFP}/ [μPa s]
20	12.6	12.6	21.6	21.7	332.6 ± 0.4	331.6	29.8 ± 3.5	24.3
40	12.7	12.7	22.4	22.6	335.6 ± 0.5	334.3	28.4 ± 1.6	24.9
60	12.8	12.8	23.3	23.5	339.1 ± 0.7	337.6	29.4 ± 5.4	25.5
80	12.9	13.0	24.1	24.4	343.1 ± 0.5	341.5	31.1 ± 4.5	26.2
100	12.9	13.1	24.9 ± 0.1	25.3	348.0 ± 1.5	346.0	29.8 ± 2.3	27.0
120	13.0	13.2	25.8 ± 0.1	26.2	352.7 ± 1.0	351.2	32.5 ± 3.9	27.8
140	13.1	13.3	26.5 ± 0.1	27.0	359.4 ± 1.5	357.0	36.0 ± 3.0	28.7
160	13.2	13.4	27.3 ± 0.1	27.8	365.6 ± 1.9	363.4	35.1 ± 3.4	29.6
180	13.3	13.5	28.0 ± 0.2	28.5	372.6 ± 2.2	370.4	39.6 ± 2.2	30.6

S14.4 Data of thermodynamic and transport properties of H_2

Table S57: Densities computed from MC and MD simulations (ρ^{MC} and ρ^{MD}), densities obtained from REFPROP[10] (ρ^{REFP}), isothermal compressibilities computed from MC simulations (β_T^{MC}), isothermal compressibilities obtained from REFPROP[10] (β_T^{REFP}), thermal expansion coefficients computed from MC simulations (α_P^{MC}), thermal expansion coefficients obtained from REFPROP[10] (α_P^{REFP}), Joule Thomson coefficients computed from MC simulations (μ_{JT}^{MC}), and Joule Thomson coefficients obtained from REFPROP[10] (μ_{JT}^{REFP}) of pure H_2 at 253 K and pressures ranging from 20 bar to 200 bar.

P/ [bar]	ρ^{MC}/ [kg/m^3]	ρ^{MD}/ [kg/m^3]	ρ^{REFP}/ [kg/m^3]	β_T^{MC}/ [10^{-4}/bar]	β_T^{REFP}/ [10^{-4}/bar]	α_P^{MC}/ [10^{-3}/K]	α_P^{REFP}/ [10^{-3}/K]	μ_{JT}^{MC}/ [10^{-3} K/bar]	μ_{JT}^{REFP}/ [10^{-3} K/bar]
20	1.9	1.9	1.9	491.5 ± 0.7	493.6	3.9	3.9	-42.4 ± 5.2	-21.1
40	3.7	3.7	3.7	242.2 ± 0.3	243.6	3.9	3.9	-38.1 ± 2.4	-22.9
60	5.5	5.5	5.5	159.6 ± 0.7	160.2	3.8	3.9	-34.1 ± 5.7	-24.7
80	7.2	7.2	7.3	117.4 ± 0.4	118.5	3.8	3.8	-40.0 ± 2.8	-26.5
100	8.9	8.8	9.0	92.5 ± 0.3	93.4	3.7	3.8	-40.6 ± 2.6	-28.3
120	10.5	10.4	10.6	76.0 ± 0.3	76.8	3.7	3.8	-40.4 ± 3.1	-30.0
140	12.0	12.0	12.2	64.3 ± 0.2	64.8	3.7	3.7	-40.3 ± 1.8	-31.6
160	13.6	13.5	13.8	55.4 ± 0.1	55.9	3.6	3.7	-41.8 ± 0.9	-33.1
180	15.1	14.9	15.3	48.6 ± 0.1	49.0	3.6	3.6	-41.7 ± 1.3	-34.4
200	16.5	16.4	16.8	42.9 ± 0.1	43.5	3.5	3.6	-44.4 ± 1.1	-35.7

Table S58: Heat capacities at constant volume computed from MC simulations (c_V^{MC}), heat capacities at constant volume obtained from REFPROP[10] (c_V^{REFP}), heat capacities at constant pressure computed from MC simulations (c_P^{MC}), heat capacities at constant pressure obtained from REFPROP[10] (c_P^{REFP}), speed of sound computed from MC simulations (c^{MC}), speed of sound obtained from REFPROP[10] (c^{REFP}), viscosities computed from MD simulations (η^{MD}), and viscosities obtained from REFPROP[10] (η^{REFP}) of pure H_2 at 253 K and pressures ranging from 20 bar to 200 bar.

P/ [bar]	c_V^{MC}/ [J/mol K]	c_V^{REFP}/ [J/mol K]	c_P^{MC}/ [J/mol K]	c_P^{REFP}/ [J/mol K]	c^{MC}/ [m/s]	c^{REFP}/ [m/s]	η^{MD}/ [μPa s]	η^{REFP}/ [μPa s]
20	20.1	20.1	28.5	28.5	1238.8 ± 0.9	1232.9	8.6 ± 0.7	8.0
40	20.1	20.1	28.6	28.7	1259.1 ± 0.8	1251.1	9.8 ± 1.1	8.0
60	20.2	20.2	28.8	28.8	1278.1 ± 3.0	1269.7	9.8 ± 0.8	8.0
80	20.2	20.2	28.9	28.9	1300.8 ± 2.2	1288.5	9.8 ± 0.7	8.1
100	20.2	20.2	29.0	29.1	1321.7 ± 2.3	1307.5	10.5 ± 0.7	8.1
120	20.3	20.3	29.1	29.2	1341.9 ± 3.3	1326.8	10.6 ± 1.0	8.2
140	20.3	20.3	29.2	29.3	1362.3 ± 2.3	1346.3	10.9 ± 0.7	8.2
160	20.3	20.4	29.3	29.3	1383.8 ± 1.4	1365.9	10.6 ± 1.3	8.3
180	20.4	20.4	29.4	29.4	1403.8 ± 2.2	1385.5	10.6 ± 0.8	8.4
200	20.4	20.4	29.4	29.5	1426.6 ± 2.2	1405.2	10.5 ± 1.0	8.4

Table S59: Densities computed from MC and MD simulations (ρ^{MC} and ρ^{MD}), densities obtained from REFPROP[10] (ρ^{REFP}), isothermal compressibilities computed from MC simulations (β_T^{MC}), isothermal compressibilities obtained from REFPROP[10] (β_T^{REFP}), thermal expansion coefficients computed from MC simulations (α_P^{MC}), thermal expansion coefficients obtained from REFPROP[10] (α_P^{REFP}), Joule Thomson coefficients computed from MC simulations ($\mu_{\mathrm{JT}}^{\mathrm{MC}}$), and Joule Thomson coefficients obtained from REFPROP[10] ($\mu_{\mathrm{JT}}^{\mathrm{REFP}}$) of pure H_2 at 273 K and pressures ranging from 20 bar to 200 bar.

P/ [bar]	ρ^{MC}/ [kg/m^3]	ρ^{MD}/ [kg/m^3]	ρ^{REFP}/ [kg/m^3]	β_T^{MC}/ [10^{-4}/bar]	β_T^{REFP}/ [10^{-4}/bar]	α_P^{MC}/ [10^{-3}/K]	α_P^{REFP}/ [10^{-3}/K]	$\mu_{\mathrm{JT}}^{\mathrm{MC}}$/ [$10^{-3}$ K/bar]	$\mu_{\mathrm{JT}}^{\mathrm{REFP}}$/ [$10^{-3}$ K/bar]
20	1.7	1.7	1.8	492.6 ± 2.0	493.8	3.6	3.6	-41.2 ± 16.8	-25.9
40	3.4	3.4	3.5	243.0 ± 0.7	243.8	3.6	3.6	-38.7 ± 5.2	-27.3
60	5.1	5.1	5.1	159.3 ± 0.5	160.5	3.5	3.6	-43.4 ± 4.3	-28.8
80	6.7	6.6	6.8	118.2 ± 0.3	118.8	3.5	3.6	-39.7 ± 2.5	-30.2
100	8.2	8.2	8.3	93.0 ± 0.4	93.8	3.5	3.5	-43.0 ± 3.3	-31.7
120	9.8	9.7	9.9	76.7 ± 0.3	77.1	3.5	3.5	-40.8 ± 2.4	-33.1
140	11.2	11.1	11.4	64.6 ± 0.2	65.2	3.4	3.5	-43.6 ± 1.7	-34.4
160	12.7	12.5	12.9	55.8 ± 0.2	56.3	3.4	3.4	-44.1 ± 1.8	-35.6
180	14.1	13.9	14.3	48.8 ± 0.3	49.4	3.3	3.4	-45.6 ± 2.6	-36.8
200	15.4	15.3	15.7	43.4 ± 0.1	43.9	3.3	3.3	-46.2 ± 1.4	-37.9

Table S60: Heat capacities at constant volume computed from MC simulations (c_V^{MC}), heat capacities at constant volume obtained from REFPROP[10] (c_V^{REFP}), heat capacities at constant pressure computed from MC simulations (c_P^{MC}), heat capacities at constant pressure obtained from REFPROP[10] (c_P^{REFP}), speed of sound computed from MC simulations (c^{MC}), speed of sound obtained from REFPROP[10] (c^{REFP}), viscosities computed from MD simulations (η^{MD}), and viscosities obtained from REFPROP[10] (η^{REFP}) of pure H_2 at 273 K and pressures ranging from 20 bar to 200 bar.

P/ [bar]	c_V^{MC}/ [J/mol K]	c_V^{REFP}/ [J/mol K]	c_P^{MC}/ [J/mol K]	c_P^{REFP}/ [J/mol K]	c^{MC}/ [m/s]	c^{REFP}/ [m/s]	η^{MD}/ [μPa s]	η^{REFP}/ [μPa s]
20	20.3	20.3	28.7	28.7	1282.6 ± 2.8	1277.4	10.7 ± 1.0	8.4
40	20.4	20.4	28.9	28.9	1302.2 ± 1.8	1295.1	10.0 ± 0.5	8.4
60	20.4	20.4	28.9	29.0	1323.2 ± 2.2	1313.0	9.8 ± 0.5	8.4
80	20.4	20.4	29.1	29.1	1341.6 ± 1.8	1331.1	10.3 ± 1.0	8.5
100	20.5	20.5	29.1	29.2	1363.1 ± 2.8	1349.4	10.3 ± 0.3	8.5
120	20.5	20.5	29.3	29.3	1381.6 ± 2.5	1367.9	11.0 ± 1.1	8.6
140	20.5	20.5	29.3	29.4	1402.8 ± 2.0	1386.5	10.4 ± 1.1	8.6
160	20.5	20.6	29.4	29.5	1422.4 ± 2.3	1405.2	10.8 ± 0.9	8.7
180	20.6	20.6	29.4 ± 0.1	29.5	1443.8 ± 4.2	1423.9	11.0 ± 1.0	8.7
200	20.6	20.7	29.5	29.6	1463.8 ± 2.6	1442.7	10.5 ± 0.4	8.8

Table S61: Densities computed from MC and MD simulations (ρ^{MC} and ρ^{MD}), densities obtained from REFPROP[10] (ρ^{REFP}), isothermal compressibilities computed from MC simulations (β_T^{MC}), isothermal compressibilities obtained from REFPROP[10] (β_T^{REFP}), thermal expansion coefficients computed from MC simulations (α_P^{MC}), thermal expansion coefficients obtained from REFPROP[10] (α_P^{REFP}), Joule Thomson coefficients computed from MC simulations ($\mu_{\mathrm{JT}}^{\mathrm{MC}}$), and Joule Thomson coefficients obtained from REFPROP[10] ($\mu_{\mathrm{JT}}^{\mathrm{REFP}}$) of pure H_2 at 293 K and pressures ranging from 20 bar to 200 bar.

P/ [bar]	ρ^{MC}/ [kg/m^3]	ρ^{MD}/ [kg/m^3]	ρ^{REFP}/ [kg/m^3]	β_T^{MC}/ [10^{-4}/bar]	β_T^{REFP}/ [10^{-4}/bar]	α_P^{MC}/ [10^{-3}/K]	α_P^{REFP}/ [10^{-3}/K]	$\mu_{\mathrm{JT}}^{\mathrm{MC}}$/ [$10^{-3}$ K/bar]	$\mu_{\mathrm{JT}}^{\mathrm{REFP}}$/ [$10^{-3}$ K/bar]
20	1.6	1.6	1.6	493.0 ± 1.9	494.1	3.4	3.4	-43.3 ± 15.9	-29.9
40	3.2	3.2	3.2	242.8 ± 0.6	244.1	3.3	3.4	-46.4 ± 4.8	-31.0
60	4.8	4.7	4.8	160.1 ± 0.7	160.8	3.3	3.3	-42.3 ± 6.2	-32.2
80	6.2	6.2	6.3	118.1 ± 0.3	119.1	3.3	3.3	-46.5 ± 2.7	-33.5
100	7.7	7.6	7.8	93.5 ± 0.4	94.1	3.2	3.3	-44.6 ± 3.5	-34.6
120	9.1	9.0	9.3	76.7 ± 0.3	77.4	3.2	3.2	-46.5 ± 2.6	-35.8
140	10.5	10.4	10.7	65.1 ± 0.2	65.6	3.2	3.2	-44.9 ± 1.8	-36.9
160	11.9	11.7	12.0	56.2 ± 0.2	56.7	3.1	3.2	-45.7 ± 2.0	-37.9
180	13.2	13.0	13.4	49.2 ± 0.2	49.7	3.1	3.2	-47.8 ± 1.8	-38.9
200	14.5	14.3	14.7	43.7 ± 0.1	44.2	3.1	3.1	-48.0 ± 1.5	-39.8

Table S62: Heat capacities at constant volume computed from MC simulations (c_V^{MC}), heat capacities at constant volume obtained from REFPROP[10] (c_V^{REFP}), heat capacities at constant pressure computed from MC simulations (c_P^{MC}), heat capacities at constant pressure obtained from REFPROP[10] (c_P^{REFP}), speed of sound computed from MC simulations (c^{MC}), speed of sound obtained from REFPROP[10] (c^{REFP}), viscosities computed from MD simulations (η^{MD}), and viscosities obtained from REFPROP[10] (η^{REFP}) of pure H_2 at 293 K and pressures ranging from 20 bar to 200 bar.

P/ [bar]	c_V^{MC}/ [J/mol K]	c_V^{REFP}/ [J/mol K]	c_P^{MC}/ [J/mol K]	c_P^{REFP}/ [J/mol K]	c^{MC}/ [m/s]	c^{REFP}/ [m/s]	η^{MD}/ [μPa s]	η^{REFP}/ [μPa s]
20	20.5	20.5	28.9	28.9	1325.8 ± 2.6	1320.6	9.9 ± 1.0	8.8
40	20.5	20.5	29.0	29.0	1345.7 ± 1.6	1337.8	11.0 ± 0.9	8.8
60	20.6	20.6	29.1	29.1	1363.9 ± 3.0	1355.2	10.4 ± 1.2	8.9
80	20.6	20.6	29.1	29.2	1384.8 ± 1.7	1372.7	11.2 ± 1.0	8.9
100	20.6	20.6	29.2	29.3	1403.0 ± 2.9	1390.3	11.0 ± 1.0	8.9
120	20.7	20.7	29.3	29.4	1423.4 ± 2.8	1408.1	11.9 ± 0.9	9.0
140	20.7	20.7	29.4	29.5	1441.1 ± 2.2	1425.9	11.4 ± 1.1	9.0
160	20.7	20.7	29.5	29.5	1460.9 ± 2.6	1443.9	10.8 ± 1.2	9.0
180	20.7	20.8	29.5	29.6	1481.5 ± 3.0	1461.8	12.4 ± 1.2	9.1
200	20.8	20.8	29.5	29.6	1500.7 ± 2.7	1479.8	10.9 ± 0.8	9.2

Table S63: Densities computed from MC and MD simulations (ρ^{MC} and ρ^{MD}), densities obtained from REFPROP[10] (ρ^{REFP}), isothermal compressibilities computed from MC simulations (β_T^{MC}), isothermal compressibilities obtained from REFPROP[10] (β_T^{REFP}), thermal expansion coefficients computed from MC simulations (α_P^{MC}), thermal expansion coefficients obtained from REFPROP[10] (α_P^{REFP}), Joule Thomson coefficients computed from MC simulations ($\mu_{\mathrm{JT}}^{\mathrm{MC}}$), and Joule Thomson coefficients obtained from REFPROP[10] ($\mu_{\mathrm{JT}}^{\mathrm{REFP}}$) of pure H_2 at 313 K and pressures ranging from 20 bar to 200 bar.

P/ [bar]	ρ^{MC}/ [kg/m^3]	ρ^{MD}/ [kg/m^3]	ρ^{REFP}/ [kg/m^3]	β_T^{MC}/ [10^{-4}/bar]	β_T^{REFP}/ [10^{-4}/bar]	α_P^{MC}/ [10^{-3}/K]	α_P^{REFP}/ [10^{-3}/K]	$\mu_{\mathrm{JT}}^{\mathrm{MC}}$/ [$10^{-3}$ K/bar]	$\mu_{\mathrm{JT}}^{\mathrm{REFP}}$/ [$10^{-3}$ K/bar]
20	1.5	1.5	1.5	492.8 ± 1.0	494.3	3.2	3.2	-51.0 ± 8.9	-33.4
40	3.0	3.0	3.0	244.1 ± 0.8	244.4	3.1	3.1	-40.3 ± 7.9	-34.3
60	4.5	4.4	4.5	160.4 ± 0.5	161.0	3.1	3.1	-44.7 ± 4.6	-35.3
80	5.9	5.8	5.9	118.7 ± 0.4	119.4	3.1	3.1	-46.2 ± 4.1	-36.3
100	7.2	7.2	7.3	93.8 ± 0.3	94.4	3.0	3.1	-47.0 ± 3.1	-37.2
120	8.6	8.5	8.7	77.1 ± 0.3	77.7	3.0	3.0	-47.5 ± 3.0	-38.2
140	9.9	9.8	10.0	65.4 ± 0.3	65.9	3.0	3.0	-46.8 ± 2.8	-39.1
160	11.2	11.0	11.3	56.5 ± 0.1	57.0	2.9	3.0	-48.3 ± 1.3	-40.0
180	12.4	12.3	12.6	49.4 ± 0.2	50.0	2.9	3.0	-51.0 ± 1.9	-40.8
200	13.6	13.5	13.9	44.1 ± 0.3	44.5	2.9	2.9	-48.6 ± 3.1	-41.6

Table S64: Heat capacities at constant volume computed from MC simulations (c_V^{MC}), heat capacities at constant volume obtained from REFPROP[10] (c_V^{REFP}), heat capacities at constant pressure computed from MC simulations (c_P^{MC}), heat capacities at constant pressure obtained from REFPROP[10] (c_P^{REFP}), speed of sound computed from MC simulations (c^{MC}), speed of sound obtained from REFPROP[10] (c^{REFP}), viscosities computed from MD simulations (η^{MD}), and viscosities obtained from REFPROP[10] (η^{REFP}) of pure H_2 at 313 K and pressures ranging from 20 bar to 200 bar.

P/ [bar]	c_V^{MC}/ [J/mol K]	c_V^{REFP}/ [J/mol K]	c_P^{MC}/ [J/mol K]	c_P^{REFP}/ [J/mol K]	c^{MC}/ [m/s]	c^{REFP}/ [m/s]	η^{MD}/ [μPa s]	η^{REFP}/ [μPa s]
20	20.6	20.6	29.0	29.0	1368.3 ± 1.4	1362.6	11.4 ± 0.9	9.2
40	20.7	20.7	29.1	29.1	1385.5 ± 2.5	1379.4	10.7 ± 1.4	9.2
60	20.7	20.7	29.2	29.2	1404.8 ± 2.2	1396.3	11.4 ± 0.8	9.3
80	20.7	20.7	29.3	29.3	1424.0 ± 2.6	1413.2	11.6 ± 0.8	9.3
100	20.7	20.8	29.3	29.4	1443.0 ± 2.5	1430.3	11.7 ± 1.4	9.3
120	20.8	20.8	29.4	29.4	1461.9 ± 2.9	1447.4	11.7 ± 0.6	9.3
140	20.8	20.8	29.5	29.5	1479.8 ± 3.2	1464.6	11.9 ± 1.5	9.4
160	20.8	20.9	29.5	29.6	1498.9 ± 1.8	1481.9	12.5 ± 1.0	9.4
180	20.8	20.9	29.5	29.6	1519.8 ± 2.7	1499.1	12.0 ± 1.0	9.5
200	20.9	20.9	29.6 ± 0.1	29.7	1535.9 ± 4.9	1516.4	12.3 ± 0.9	9.5

S14.5 Data of thermodynamic and transport properties of CH_4

Table S65: Densities computed from MC and MD simulations (ρ^{MC} and ρ^{MD}), densities obtained from REFPROP[10] (ρ^{REFP}), isothermal compressibilities computed from MC simulations (β_T^{MC}), isothermal compressibilities obtained from REFPROP[10] (β_T^{REFP}), thermal expansion coefficients computed from MC simulations (α_P^{MC}), thermal expansion coefficients obtained from REFPROP[10] (α_P^{REFP}), Joule Thomson coefficients computed from MC simulations ($\mu_{\mathrm{JT}}^{\mathrm{MC}}$), and Joule Thomson coefficients obtained from REFPROP[10] ($\mu_{\mathrm{JT}}^{\mathrm{REFP}}$) of pure CH_4 at 253 K and pressures ranging from 20 bar to 200 bar.

P/ [bar]	ρ^{MC}/ [kg/m^3]	ρ^{MD}/ [kg/m^3]	ρ^{REFP}/ [kg/m^3]	β_T^{MC}/ [10^{-4}/bar]	β_T^{REFP}/ [10^{-4}/bar]	α_P^{MC}/ [10^{-3}/K]	α_P^{REFP}/ [10^{-3}/K]	$\mu_{\mathrm{JT}}^{\mathrm{MC}}$/ [$10^{-3}$ K/bar]	$\mu_{\mathrm{JT}}^{\mathrm{REFP}}$/ [$10^{-3}$ K/bar]
20	16.1	16.1	16.3	531.3 ± 0.5	533.3	4.8	4.9	563.5 ± 3.1	598.4
40	34.5	34.3	34.9	284.8 ± 0.2	286.3	5.9	6.1	558.7 ± 1.8	580.6
60	55.6	55.1 ± 0.1	56.4	203.9 ± 0.2	205.0	7.4	7.6	536.8 ± 1.8	546.2
80	79.9	79.0 ± 0.3	81.1	163.0 ± 0.7	162.3	9.3	9.5	495.6 ± 4.0	490.5
100	107.5	105.5 ± 0.2	108.6	133.2 ± 0.8	130.5	11.1 ± 0.1	11.1	429.2 ± 5.1	414.9
120	136.2 ± 0.1	133.3 ± 0.3	136.8	103.6 ± 0.9	100.6	12.1 ± 0.1	11.8	344.2 ± 4.8	330.3
140	162.9 ± 0.2	159.0 ± 0.4	162.8	76.4 ± 0.6	74.1	11.7 ± 0.1	11.2	263.7 ± 2.2	252.3
160	185.3 ± 0.1	181.1 ± 0.2	184.9	55.2 ± 0.6	54.1	10.4 ± 0.1	10.1	197.5 ± 4.1	190.4
180	203.6 ± 0.1	199.7 ± 0.2	203.0	40.5 ± 0.4	40.5	9.0 ± 0.1	8.9	147.2 ± 3.1	144.2
200	218.5 ± 0.1	214.6 ± 0.2	218.0	31.0 ± 0.2	31.4	7.9	7.8	111.0 ± 1.4	109.9

Table S66: Heat capacities at constant volume computed from MC simulations (c_V^{MC}), heat capacities at constant volume obtained from REFPROP[10] (c_V^{REFP}), heat capacities at constant pressure computed from MC simulations (c_P^{MC}), heat capacities at constant pressure obtained from REFPROP[10] (c_P^{REFP}), speed of sound computed from MC simulations (c^{MC}), speed of sound obtained from REFPROP[10] (c^{REFP}), viscosities computed from MD simulations (η^{MD}), and viscosities obtained from REFPROP[10] (η^{REFP}) of pure CH_4 at 253 K and pressures ranging from 20 bar to 200 bar.

P/ [bar]	c_V^{MC}/ [J/mol K]	c_V^{REFP}/ [J/mol K]	c_P^{MC}/ [J/mol K]	c_P^{REFP}/ [J/mol K]	c^{MC}/ [m/s]	c^{REFP}/ [m/s]	η^{MD}/ [μPa s]	η^{REFP}/ [μPa s]
20	26.2	26.6	37.0	37.6	406.1 ± 0.2	404.0	11.8 ± 1.0	10.0
40	26.6	27.2	41.0	42.1	396.2 ± 0.2	393.7	13.3 ± 1.3	10.5
60	27.1	27.9	46.6	48.3	389.7 ± 0.3	386.9	14.2 ± 1.1	11.3
80	27.6	28.6	54.3 ± 0.1	56.1	388.7 ± 0.9	386.2	14.9 ± 1.7	12.5
100	28.0	29.1	63.3 ± 0.3	64.4	397.4 ± 1.5	395.3	16.6 ± 1.2	14.0
120	28.2 ± 0.1	29.4	70.3 ± 0.4	70.4	420.0 ± 2.2	416.9	19.0 ± 1.9	15.9
140	28.3	29.5	72.9 ± 0.2	72.0	454.4 ± 1.8	450.0	20.9 ± 0.9	17.9
160	28.4	29.5	71.5 ± 0.5	70.7	496.5 ± 3.4	489.5	23.5 ± 1.5	19.9
180	28.4	29.4	68.5 ± 0.5	68.4	540.5 ± 3.3	531.2	26.4 ± 1.7	21.9
200	28.5	29.4	65.7 ± 0.2	66.0	583.3 ± 2.0	572.5	27.2 ± 0.9	23.7

Table S67: Densities computed from MC and MD simulations (ρ^{MC} and ρ^{MD}), densities obtained from REFPROP[10] (ρ^{REFP}), isothermal compressibilities computed from MC simulations (β_T^{MC}), isothermal compressibilities obtained from REFPROP[10] (β_T^{REFP}), thermal expansion coefficients computed from MC simulations (α_P^{MC}), thermal expansion coefficients obtained from REFPROP[10] (α_P^{REFP}), Joule Thomson coefficients computed from MC simulations ($\mu_{\mathrm{JT}}^{\mathrm{MC}}$), and Joule Thomson coefficients obtained from REFPROP[10] ($\mu_{\mathrm{JT}}^{\mathrm{REFP}}$) of pure CH_4 at 273 K and pressures ranging from 20 bar to 200 bar.

P/ [bar]	ρ^{MC}/ [kg/m^3]	ρ^{MD}/ [kg/m^3]	ρ^{REFP}/ [kg/m^3]	β_T^{MC}/ [10^{-4}/bar]	β_T^{REFP}/ [10^{-4}/bar]	α_P^{MC}/ [10^{-3}/K]	α_P^{REFP}/ [10^{-3}/K]	$\mu_{\mathrm{JT}}^{\mathrm{MC}}$/ [$10^{-3}$ K/bar]	$\mu_{\mathrm{JT}}^{\mathrm{REFP}}$/ [$10^{-3}$ K/bar]
20	14.7	14.7	14.8	523.0 ± 0.3	524.7	4.3	4.3	483.3 ± 2.4	512.4
40	30.9	30.7	31.2	274.6 ± 0.3	275.5	5.0	5.1	477.1 ± 2.4	494.6
60	48.7	48.4 ± 0.1	49.3	191.4 ± 0.4	192.1	5.9	6.0	458.3 ± 3.1	467.6
80	68.2	67.5 ± 0.1	69.0	148.9 ± 0.5	148.8	6.9	7.0	429.3 ± 3.2	429.6
100	89.2	87.9 ± 0.1	90.1	121.1 ± 0.6	120.0	7.9	7.9	388.9 ± 4.0	381.6
120	111.1	109.1 ± 0.1	112.0	99.1 ± 0.6	97.3	8.6 ± 0.1	8.6	338.3 ± 3.6	327.2
140	132.8 ± 0.1	130.1 ± 0.2	133.3	79.6 ± 0.3	78.0	8.9	8.8	281.9 ± 2.0	271.9
160	153.0	149.9 ± 0.4	153.3	62.9 ± 0.4	61.9	8.7 ± 0.1	8.5	228.9 ± 2.5	220.9
180	171.1 ± 0.1	167.8 ± 0.3	171.2	49.1 ± 0.3	49.0	8.1 ± 0.1	8.0	180.6 ± 2.2	177.2
200	187.1 ± 0.1	183.4 ± 0.2	186.9	39.3 ± 0.2	39.2	7.5	7.4	144.7 ± 1.3	141.5

Table S68: Heat capacities at constant volume computed from MC simulations (c_V^{MC}), heat capacities at constant volume obtained from REFPROP[10] (c_V^{REFP}), heat capacities at constant pressure computed from MC simulations (c_P^{MC}), heat capacities at constant pressure obtained from REFPROP[10] (c_P^{REFP}), speed of sound computed from MC simulations (c^{MC}), speed of sound obtained from REFPROP[10] (c^{REFP}), viscosities computed from MD simulations (η^{MD}), and viscosities obtained from REFPROP[10] (η^{REFP}) of pure CH_4 at 273 K and pressures ranging from 20 bar to 200 bar.

P/ [bar]	c_V^{MC}/ [J/mol K]	c_V^{REFP}/ [J/mol K]	c_P^{MC}/ [J/mol K]	c_P^{REFP}/ [J/mol K]	c^{MC}/ [m/s]	c^{REFP}/ [m/s]	η^{MD}/ [μPa s]	η^{REFP}/ [μPa s]
20	26.5	27.0	36.9	37.5	424.4 ± 0.1	422.2	13.7 ± 2.6	10.7
40	26.9	27.4	39.8	40.7	417.6 ± 0.3	415.3	12.9 ± 1.4	11.1
60	27.2	27.9	43.4	44.6	413.6 ± 0.5	411.2	13.2 ± 1.2	11.8
80	27.6	28.4	47.9 ± 0.1	49.3	413.5 ± 0.8	411.2	14.5 ± 1.2	12.6
100	27.8	28.8	52.9 ± 0.1	54.1	419.0 ± 1.1	417.0	16.6 ± 0.8	13.6
120	28.1	29.1	57.6 ± 0.2	58.5	431.3 ± 1.5	429.7	16.9 ± 0.9	14.9
140	28.2	29.3	61.0 ± 0.1	61.6	452.2 ± 1.1	449.6	19.5 ± 0.8	16.3
160	28.4	29.4	62.8 ± 0.2	63.1	479.5 ± 1.7	475.5	21.3 ± 1.2	17.8
180	28.5	29.5	62.6 ± 0.2	63.2	511.7 ± 2.0	505.6	22.9 ± 1.5	19.4
200	28.5	29.5	62.2 ± 0.2	62.6	544.6 ± 1.3	537.8	24.2 ± 1.6	20.9

Table S69: Densities computed from MC and MD simulations (ρ^{MC} and ρ^{MD}), densities obtained from REFPROP[10] (ρ^{REFP}), isothermal compressibilities computed from MC simulations (β_T^{MC}), isothermal compressibilities obtained from REFPROP[10] (β_T^{REFP}), thermal expansion coefficients computed from MC simulations (α_P^{MC}), thermal expansion coefficients obtained from REFPROP[10] (α_P^{REFP}), Joule Thomson coefficients computed from MC simulations ($\mu_{\mathrm{JT}}^{\mathrm{MC}}$), and Joule Thomson coefficients obtained from REFPROP[10] ($\mu_{\mathrm{JT}}^{\mathrm{REFP}}$) of pure CH_4 at 293 K and pressures ranging from 20 bar to 200 bar.

P/ [bar]	ρ^{MC}/ [kg/m^3]	ρ^{MD}/ [kg/m^3]	ρ^{REFP}/ [kg/m^3]	β_T^{MC}/ [10^{-4}/bar]	β_T^{REFP}/ [10^{-4}/bar]	α_P^{MC}/ [10^{-3}/K]	α_P^{REFP}/ [10^{-3}/K]	$\mu_{\mathrm{JT}}^{\mathrm{MC}}$/ [$10^{-3}$ K/bar]	$\mu_{\mathrm{JT}}^{\mathrm{REFP}}$/ [$10^{-3}$ K/bar]
20	13.6	13.5	13.7	517.8 ± 0.7	518.6	3.9	3.9	422.4 ± 4.9	442.1
40	28.2	27.9 ± 0.1	28.4	267.8 ± 0.2	268.5	4.4	4.4	411.7 ± 1.9	425.4
60	43.8	43.4 ± 0.1	44.1	184.3 ± 0.2	184.4	5.0	5.0	397.5 ± 1.6	403.0
80	60.3	59.7	60.9	141.0 ± 0.4	141.1	5.6	5.6	371.9 ± 2.6	374.5
100	77.7	76.6 ± 0.1	78.4	114.1 ± 0.2	113.5	6.1	6.2	343.8 ± 2.1	340.3
120	95.6	94.0 ± 0.1	96.3	94.2 ± 0.3	93.3	6.6	6.7	308.9 ± 1.7	302.2
140	113.5	111.5 ± 0.2	114.1	78.1 ± 0.3	77.1	6.9	6.9	270.4 ± 1.9	262.6
160	130.9	128.6 ± 0.2	131.4	64.4 ± 0.3	63.7	7.0	7.0	230.8 ± 2.1	224.0
180	147.2 ± 0.1	144.3 ± 0.2	147.5	53.4 ± 0.2	52.7	6.9	6.8	195.2 ± 1.9	188.4
200	162.3 ± 0.1	159.1 ± 0.1	162.4	44.1 ± 0.1	43.7	6.6	6.6	161.8 ± 1.0	156.8

Table S70: Heat capacities at constant volume computed from MC simulations (c_V^{MC}), heat capacities at constant volume obtained from REFPROP[10] (c_V^{REFP}), heat capacities at constant pressure computed from MC simulations (c_P^{MC}), heat capacities at constant pressure obtained from REFPROP[10] (c_P^{REFP}), speed of sound computed from MC simulations (c^{MC}), speed of sound obtained from REFPROP[10] (c^{REFP}), viscosities computed from MD simulations (η^{MD}), and viscosities obtained from REFPROP[10] (η^{REFP}) of pure CH_4 at 293 K and pressures ranging from 20 bar to 200 bar.

P/ [bar]	c_V^{MC}/ [J/mol K]	c_V^{REFP}/ [J/mol K]	c_P^{MC}/ [J/mol K]	c_P^{REFP}/ [J/mol K]	c^{MC}/ [m/s]	c^{REFP}/ [m/s]	η^{MD}/ [μPa s]	η^{REFP}/ [μPa s]
20	27.0	27.6	37.0	37.6	441.0 ± 0.3	438.9	13.8 ± 2.1	11.3
40	27.3	27.9	39.3	40.1	436.7 ± 0.2	434.3	14.4 ± 1.2	11.7
60	27.6	28.2	41.9	42.9	434.2 ± 0.3	432.0	14.6 ± 0.7	12.3
80	27.8	28.6	44.9	46.0	435.5 ± 0.6	432.9	13.6 ± 2.0	12.9
100	28.0	28.9	48.1 ± 0.1	49.2	439.8 ± 0.6	437.7	15.4 ± 0.9	13.7
120	28.2	29.1	51.2 ± 0.1	52.3	448.8 ± 0.8	447.0	18.2 ± 2.2	14.7
140	28.4	29.3	54.0 ± 0.1	54.9	463.1 ± 0.9	460.9	19.1 ± 1.1	15.7
160	28.6	29.5	56.0 ± 0.1	56.7	482.1 ± 1.2	479.2	19.3 ± 1.6	16.9
180	28.7	29.6	57.3 ± 0.2	57.8	504.3 ± 1.3	501.2	22.3 ± 1.7	18.1
200	28.7	29.7	57.8 ± 0.1	58.3	530.1 ± 1.0	525.8	23.7 ± 1.9	19.3

Table S71: Densities computed from MC and MD simulations (ρ^{MC} and ρ^{MD}), densities obtained from REFPROP[10] (ρ^{REFP}), isothermal compressibilities computed from MC simulations (β_T^{MC}), isothermal compressibilities obtained from REFPROP[10] (β_T^{REFP}), thermal expansion coefficients computed from MC simulations (α_P^{MC}), thermal expansion coefficients obtained from REFPROP[10] (α_P^{REFP}), Joule Thomson coefficients computed from MC simulations ($\mu_{\text{JT}}^{\text{MC}}$), and Joule Thomson coefficients obtained from REFPROP[10] ($\mu_{\text{JT}}^{\text{REFP}}$) of pure CH_4 at 313 K and pressures ranging from 20 bar to 200 bar.

P/ [bar]	ρ^{MC}/ [kg/m^3]	ρ^{MD}/ [kg/m^3]	ρ^{REFP}/ [kg/m^3]	β_T^{MC}/ [10^{-4}/bar]	β_T^{REFP}/ [10^{-4}/bar]	α_P^{MC}/ [10^{-3}/K]	α_P^{REFP}/ [10^{-3}/K]	$\mu_{\text{JT}}^{\text{MC}}$/ [$10^{-3}$ K/bar]	$\mu_{\text{JT}}^{\text{REFP}}$/ [$10^{-3}$ K/bar]
20	12.6	12.5	12.7	513.4 ± 0.7	514.2	3.5	3.6	366.7 ± 4.9	383.5
40	25.9	25.7	26.1	263.2 ± 0.4	263.6	3.9	4.0	357.9 ± 2.6	368.2
60	39.9	39.6 ± 0.1	40.2	178.9 ± 0.2	179.3	4.3	4.4	341.6 ± 1.6	349.3
80	54.5	53.9	54.9	136.2 ± 0.4	136.2	4.7	4.8	324.0 ± 2.6	326.7
100	69.5	68.5	70.0	109.2 ± 0.2	109.3	5.1	5.2	300.3 ± 2.0	300.7
120	84.8	83.5 ± 0.1	85.4	90.6 ± 0.2	90.1	5.4	5.5	276.1 ± 2.1	272.2
140	100.2	98.5 ± 0.1	100.7	76.0 ± 0.4	75.5	5.7	5.7	247.8 ± 2.9	242.6
160	115.2	113.1 ± 0.1	115.7	63.9 ± 0.2	63.6	5.8	5.8	217.4 ± 2.1	213.1
180	129.7	127.3 ± 0.1	130.1	54.3 ± 0.1	53.9	5.8	5.8	190.2 ± 1.0	184.9
200	143.4	140.8 ± 0.1	143.7	46.0 ± 0.2	45.8	5.7	5.7	162.7 ± 1.5	158.9

Table S72: Heat capacities at constant volume computed from MC simulations (c_V^{MC}), heat capacities at constant volume obtained from REFPROP[10] (c_V^{REFP}), heat capacities at constant pressure computed from MC simulations (c_P^{MC}), heat capacities at constant pressure obtained from REFPROP[10] (c_P^{REFP}), speed of sound computed from MC simulations (c^{MC}), speed of sound obtained from REFPROP[10] (c^{REFP}), viscosities computed from MD simulations (η^{MD}), and viscosities obtained from REFPROP[10] (η^{REFP}) of pure CH_4 at 313 K and pressures ranging from 20 bar to 200 bar.

P/ [bar]	c_V^{MC}/ [J/mol K]	c_V^{REFP}/ [J/mol K]	c_P^{MC}/ [J/mol K]	c_P^{REFP}/ [J/mol K]	c^{MC}/ [m/s]	c^{REFP}/ [m/s]	η^{MD}/ [μPa s]	η^{REFP}/ [μPa s]
20	27.7	28.3	37.4	38.0	456.5 ± 0.3	454.2	15.8 ± 3.8	11.9
40	27.9	28.5	39.2	40.0	453.8 ± 0.3	451.3	14.5 ± 1.1	12.3
60	28.1	28.8	41.2	42.1	453.2 ± 0.2	450.5	14.9 ± 1.2	12.8
80	28.3	29.1	43.4	44.4	454.6 ± 0.6	452.1	15.4 ± 1.0	13.3
100	28.5	29.3	45.6	46.7	459.3 ± 0.6	456.6	16.8 ± 1.2	14.0
120	28.6	29.5	47.9 ± 0.1	49.0	466.5 ± 0.7	464.3	17.7 ± 1.8	14.8
140	28.8	29.7	50.0 ± 0.1	51.0	477.6 ± 1.3	475.2	18.7 ± 1.4	15.6
160	28.9	29.8	51.6 ± 0.1	52.6	492.4 ± 1.0	489.4	20.3 ± 1.3	16.5
180	29.0	30.0	53.0 ± 0.1	53.9	509.3 ± 0.7	506.4	20.8 ± 1.6	17.5
200	29.1	30.1	53.9 ± 0.1	54.7	529.5 ± 1.1	525.8	22.6 ± 0.7	18.5

S15 Data of thermodynamic and transport properties of binary systems

This section shows the thermodynamic and transport properties with uncertainties computed from Monte Carlo (MC) and Molecular Dynamics (MD) simulations, respectively, for binary systems listed in Table S14 with 1, 5, and 10 mole% of impurities. The thermodynamic properties computed from MC simulations are (1) densities (ρ), (2) isothermal compressibilities at constant temperature (β_T), (3) thermal expansion coefficients (α_P), (4) heat capacities at constant volume (c_V), (5) heat capacities at constant pressure (c_P), (6) Joule-Thomson Coefficients (μ_{JT}), and (7) speed of sound (c). Transport property, (8) viscosities (η) are computed from MD simulations. Densities computed from MD simulations are also reported in this section. The thermodynamic and transport properties are computed at temperatures 253 K, 273 K, 293 K, and 313K, and pressure ranging from 20 bar to 200 bar in intervals of 20 bar. For each condition (concentration, temperature, and pressure), 10 independent simulations are performed, and each simulation starts with a different initial configuration. These 10 simulations are divided into 5 blocks from which average values and uncertainties of thermodynamic and transport properties are calculated as the standard deviation of 5 blocks. The thermodynamic and transport properties uncertainty values with more than one significant decimal figure are not shown. Properties computed from molecular simulations are compared with data calculated from the GERG-2008 EoS,[30] which are obtained from REFPROP.[10] At conditions close to the critical point, REFPROP[10] fails to calculate heat capacities at constant volume (c_V), heat capacities at constant pressure (c_P), Joule-Thomson Coefficients (μ_{JT}), and speed of sound (c). A similar issue was also encountered during simulations, so the unavailable EoS data and simulation data are represented by ”-”.

S15.1 Thermodynamic and transport properties data of binary CO_2 mixture with 1 mole% impurity of N_2

Table S73: Densities computed from MC and MD simulations (ρ^{MC} and ρ^{MD}), densities obtained from REFPROP[10] (ρ^{REFP}), isothermal compressibilities computed from MC simulations (β_T^{MC}), isothermal compressibilities obtained from REFPROP[10] (β_T^{REFP}), thermal expansion coefficients computed from MC simulations (α_P^{MC}), thermal expansion coefficients obtained from REFPROP[10] (α_P^{REFP}), Joule Thomson coefficients computed from MC simulations ($\mu_{\mathrm{JT}}^{\mathrm{MC}}$), and Joule Thomson coefficients obtained from REFPROP[10] ($\mu_{\mathrm{JT}}^{\mathrm{REFP}}$) of CO_2-N_2 binary mixture with 1 mole% impurity of N_2 at 253 K and pressures ranging from 20 bar to 200 bar.

P/ [bar]	ρ^{MC}/ [kg/m^3]	ρ^{MD}/ [kg/m^3]	ρ^{REFP}/ [kg/m^3]	β_T^{MC}/ [10^{-5}/bar]	β_T^{REFP}/ [10^{-5}/bar]	α_P^{MC}/ [10^{-4}/K]	α_P^{REFP}/ [10^{-4}/K]	$\mu_{\mathrm{JT}}^{\mathrm{MC}}$/ [$10^{-3}$ K/bar]	$\mu_{\mathrm{JT}}^{\mathrm{REFP}}$/ [$10^{-3}$ K/bar]
20	50.8	52.2 ± 0.2	80.6	6410.7 ± 46.1	6862.2	77.4 ± 0.6	167.8	1576.2 ± 26.7	–
40	1026.9 ± 0.4	1025.6 ± 1.3	1030.4	37.2 ± 0.6	43.4	43.6 ± 0.8	45.8	4.6 ± 0.9	7.3
60	1034.0 ± 0.2	1032.6 ± 0.6	1039.0	35.0 ± 0.4	39.8	41.9 ± 0.6	43.2	2.7 ± 0.7	4.3
80	1041.3 ± 0.2	1039.6 ± 0.8	1047.0	32.5 ± 0.4	36.8	39.8 ± 0.4	41.0	0.3 ± 0.4	1.8
100	1047.6 ± 0.4	1046.6 ± 0.4	1054.4	30.3 ± 0.4	34.2	37.9 ± 0.5	39.1	-1.9 ± 0.5	-0.5
120	1053.8 ± 0.2	1053.4 ± 0.6	1061.4	29.2 ± 0.6	32.1	37.3 ± 1.0	37.5	-2.6 ± 1.1	-2.5
140	1059.8 ± 0.4	1059.5 ± 0.3	1068.1	27.4 ± 0.6	30.2	35.6 ± 0.6	36.0	-4.6 ± 0.7	-4.2
160	1065.4 ± 0.5	1065.3 ± 0.3	1074.3	25.7 ± 0.4	28.5	34.0 ± 0.5	34.8	-6.5 ± 0.6	-5.8
180	1070.7 ± 0.3	1070.7 ± 0.5	1080.3	24.8 ± 0.2	27.1	33.4 ± 0.2	33.6	-7.2 ± 0.3	-7.2
200	1076.2 ± 0.5	1075.8 ± 0.4	1086.1	23.3 ± 0.3	25.8	31.8 ± 0.4	32.6	-9.3 ± 0.5	-8.5

Table S74: Heat capacities at constant volume computed from MC simulations (c_V^{MC}), heat capacities at constant volume obtained from REFPROP[10] (c_V^{REFP}), heat capacities at constant pressure computed from MC simulations (c_P^{MC}), heat capacities at constant pressure obtained from REFPROP[10] (c_P^{REFP}), speed of sound computed from MC simulations (c^{MC}), speed of sound obtained from REFPROP[10] (c^{REFP}), viscosities computed from MD simulations (η^{MD}), and viscosities obtained from REFPROP[10] (η^{REFP}) of CO_2-N_2 binary mixture with 1 mole% impurity of N_2 at 253 K and pressures ranging from 20 bar to 200 bar.

P/ [bar]	c_V^{MC}/ [J/mol K]	c_V^{REFP}/ [J/mol K]	c_P^{MC}/ [J/mol K]	c_P^{REFP}/ [J/mol K]	c^{MC}/ [m/s]	c^{REFP}/ [m/s]	η^{MD}/ [μPa s]	η^{REFP}/ [μPa s]
20	32.0	–	52.5 ± 0.2	–	224.4 ± 0.9	–	12.2 ± 1.0	13.2
40	40.4	41.1	95.8 ± 1.2	93.2	788.2 ± 8.0	712.0	147.8 ± 2.9	139.8
60	40.5 ± 0.1	41.1	94.7 ± 0.9	91.2	803.7 ± 5.8	733.2	151.4 ± 2.8	143.8
80	40.6 ± 0.1	41.1	92.5 ± 0.5	89.5	820.8 ± 5.4	752.8	156.8 ± 1.8	147.6
100	40.7 ± 0.1	41.1	90.7 ± 0.6	88.1	837.7 ± 5.7	771.2	168.2 ± 9.8	151.2
120	40.5 ± 0.1	41.1	90.8 ± 1.5	86.9	853.2 ± 11.5	788.4	165.0 ± 15.1	154.8
140	40.7 ± 0.1	41.1	89.2 ± 0.7	85.8	869.7 ± 9.5	804.8	163.1 ± 22.0	158.3
160	40.7 ± 0.1	41.1	87.8 ± 0.8	84.9	887.8 ± 7.7	820.3	180.2 ± 21.1	161.7
180	40.8 ± 0.1	41.2	87.5 ± 0.4	84.0	897.9 ± 4.1	835.1	181.8 ± 17.6	165.0
200	40.9 ± 0.1	41.2	85.5 ± 0.7	83.2	912.9 ± 6.7	849.2	198.7 ± 42.4	168.3

Table S75: Densities computed from MC and MD simulations (ρ^{MC} and ρ^{MD}), densities obtained from REFPROP[10] (ρ^{REFP}), isothermal compressibilities computed from MC simulations (β_T^{MC}), isothermal compressibilities obtained from REFPROP[10] (β_T^{REFP}), thermal expansion coefficients computed from MC simulations (α_P^{MC}), thermal expansion coefficients obtained from REFPROP[10] (α_P^{REFP}), Joule Thomson coefficients computed from MC simulations ($\mu_{\mathrm{JT}}^{\mathrm{MC}}$), and Joule Thomson coefficients obtained from REFPROP[10] ($\mu_{\mathrm{JT}}^{\mathrm{REFP}}$) of CO_2-N_2 binary mixture with 1 mole% impurity of N_2 at 273 K and pressures ranging from 20 bar to 200 bar.

P/ [bar]	ρ^{MC}/ [kg/m^3]	ρ^{MD}/ [kg/m^3]	ρ^{REFP}/ [kg/m^3]	β_T^{MC}/ [10^{-5}/bar]	β_T^{REFP}/ [10^{-5}/bar]	α_P^{MC}/ [10^{-4}/K]	α_P^{REFP}/ [10^{-4}/K]	$\mu_{\mathrm{JT}}^{\mathrm{MC}}$/ [$10^{-3}$ K/bar]	$\mu_{\mathrm{JT}}^{\mathrm{REFP}}$/ [$10^{-3}$ K/bar]
20	44.5	45.5	45.4	5889.7 ± 18.7	6005.9	58.6 ± 0.2	62.0	1265.7 ± 13.2	1384.3
40	921.8 ± 1.2	920.1 ± 0.8	873.1	84.5 ± 2.8	208.8	68.7 ± 1.7	124.4	36.8 ± 2.1	–
60	936.3 ± 0.6	933.8 ± 1.4	935.7	71.5 ± 1.2	85.4	61.2 ± 0.7	65.4	29.3 ± 0.9	35.1
80	948.4 ± 0.8	946.9 ± 0.4	950.5	59.8 ± 1.2	72.1	53.7 ± 0.9	58.2	21.3 ± 1.1	27.2
100	960.0 ± 0.6	959.2 ± 0.4	963.3	55.6 ± 1.4	62.7	51.8 ± 1.2	53.0	18.9 ± 1.5	21.1
120	969.6 ± 0.4	969.5 ± 0.9	974.8	48.8 ± 1.7	55.7	47.1 ± 1.6	48.9	13.4 ± 2.1	16.2
140	979.4 ± 0.7	978.4 ± 0.9	985.1	44.2 ± 0.9	50.3	44.2 ± 0.7	45.7	9.8 ± 0.9	12.1
160	987.4 ± 0.4	987.0 ± 0.4	994.6	42.1 ± 0.8	45.9	43.1 ± 0.9	43.1	8.3 ± 1.1	8.7
180	995.2 ± 0.6	994.4 ± 0.5	1003.4	38.9 ± 0.7	42.2	40.8 ± 0.8	40.8	5.5 ± 1.1	5.7
200	1002.4 ± 0.5	1002.6 ± 0.4	1011.6	36.1 ± 0.7	39.2	38.7 ± 0.8	38.9	2.8 ± 1.1	3.1

Table S76: Heat capacities at constant volume computed from MC simulations (c_V^{MC}), heat capacities at constant volume obtained from REFPROP[10] (c_V^{REFP}), heat capacities at constant pressure computed from MC simulations (c_P^{MC}), heat capacities at constant pressure obtained from REFPROP[10] (c_P^{REFP}), speed of sound computed from MC simulations (c^{MC}), speed of sound obtained from REFPROP[10] (c^{REFP}), viscosities computed from MD simulations (η^{MD}), and viscosities obtained from REFPROP[10] (η^{REFP}) of CO_2-N_2 binary mixture with 1 mole% impurity of N_2 at 273 K and pressures ranging from 20 bar to 200 bar.

P/ [bar]	c_V^{MC}/ [J/mol K]	c_V^{REFP}/ [J/mol K]	c_P^{MC}/ [J/mol K]	c_P^{REFP}/ [J/mol K]	c^{MC}/ [m/s]	c^{REFP}/ [m/s]	η^{MD}/ [μPa s]	η^{REFP}/ [μPa s]
20	31.0	31.5	46.7 ± 0.1	48.4	239.9 ± 0.4	237.5	3.3 ± 4.6	14.0
40	40.2 ± 0.1	–	113.1 ± 1.5	–	601.1 ± 10.8	–	105.3 ± 2.1	86.7
60	40.0 ± 0.1	40.9	107.3 ± 0.9	104.9	633.2 ± 5.7	566.4	112.3 ± 3.4	103.2
80	40.0 ± 0.1	40.7	101.2 ± 0.9	99.9	668.4 ± 7.5	598.3	117.4 ± 8.4	107.7
100	39.9 ± 0.1	40.6	100.4 ± 1.4	96.2	686.6 ± 9.9	626.1	117.3 ± 3.4	111.8
120	40.0 ± 0.1	40.6	96.2 ± 1.9	93.4	713.1 ± 14.4	651.0	121.2 ± 4.8	115.7
140	40.0 ± 0.1	40.5	94.0 ± 0.9	91.1	736.8 ± 8.0	673.7	126.0 ± 6.8	119.3
160	40.0 ± 0.1	40.5	93.5 ± 1.2	89.2	749.3 ± 8.7	694.6	126.2 ± 4.2	122.8
180	40.0 ± 0.1	40.5	91.7 ± 1.2	87.6	769.2 ± 8.8	714.1	131.2 ± 6.1	126.1
200	40.0 ± 0.1	40.5	89.7 ± 1.0	86.2	786.7 ± 9.4	732.4	143.2 ± 21.4	129.3

Table S77: Densities computed from MC and MD simulations (ρ^{MC} and ρ^{MD}), densities obtained from REFPROP[10] (ρ^{REFP}), isothermal compressibilities computed from MC simulations (β_T^{MC}), isothermal compressibilities obtained from REFPROP[10] (β_T^{REFP}), thermal expansion coefficients computed from MC simulations (α_P^{MC}), thermal expansion coefficients obtained from REFPROP[10] (α_P^{REFP}), Joule Thomson coefficients computed from MC simulations ($\mu_{\mathrm{JT}}^{\mathrm{MC}}$), and Joule Thomson coefficients obtained from REFPROP[10] ($\mu_{\mathrm{JT}}^{\mathrm{REFP}}$) of CO_2-N_2 binary mixture with 1 mole% impurity of N_2 at 293 K and pressures ranging from 20 bar to 200 bar.

P/ [bar]	ρ^{MC}/ [kg/m^3]	ρ^{MD}/ [kg/m^3]	ρ^{REFP}/ [kg/m^3]	β_T^{MC}/ [10^{-5}/bar]	β_T^{REFP}/ [10^{-5}/bar]	α_P^{MC}/ [10^{-4}/K]	α_P^{REFP}/ [10^{-4}/K]	$\mu_{\mathrm{JT}}^{\mathrm{MC}}$/ [$10^{-3}$ K/bar]	$\mu_{\mathrm{JT}}^{\mathrm{REFP}}$/ [$10^{-3}$ K/bar]
20	40.0	40.8 ± 0.1	40.6	5628.1 ± 21.2	5698.0	48.7 ± 0.2	50.5	1050.2 ± 15.8	1143.2
40	93.2	95.7 ± 0.5	96.6	3478.2 ± 17.4	3620.4	81.6 ± 0.4	90.0	1081.1 ± 10.3	1142.6
60	652.6 ± 150.9	650.9 ± 123.6	491.8	1087.2 ± 874.5	28 394.9	180.2 ± 9.0	4311.4	253.4 ± 137.7	–
80	817.6 ± 1.2	814.6 ± 1.8	808.7	179.8 ± 5.4	244.3	102.5 ± 2.6	121.0	80.9 ± 3.3	100.5
100	844.1 ± 0.8	843.2 ± 1.2	841.1	134.0 ± 4.5	161.2	84.2 ± 2.6	90.2	63.0 ± 3.5	72.4
120	863.5 ± 1.1	861.9 ± 0.9	864.9	108.2 ± 2.3	122.4	72.5 ± 1.3	74.6	50.7 ± 1.8	55.5
140	881.4 ± 1.1	881.0 ± 1.5	884.2	87.1 ± 1.5	99.4	62.8 ± 1.0	64.8	39.4 ± 1.4	43.8
160	895.0 ± 0.4	895.2 ± 0.9	900.5	75.6 ± 0.8	84.1	56.9 ± 0.5	58.0	32.3 ± 0.7	35.0
180	908.2 ± 0.4	907.4 ± 0.5	914.7	66.2 ± 2.4	73.1	52.2 ± 1.8	52.9	26.1 ± 2.6	28.0
200	920.1 ± 0.8	919.2 ± 0.8	927.4	60.1 ± 0.5	64.8	49.3 ± 0.4	48.9	22.0 ± 0.6	22.4

Table S78: Heat capacities at constant volume computed from MC simulations (c_V^{MC}), heat capacities at constant volume obtained from REFPROP[10] (c_V^{REFP}), heat capacities at constant pressure computed from MC simulations (c_P^{MC}), heat capacities at constant pressure obtained from REFPROP[10] (c_P^{REFP}), speed of sound computed from MC simulations (c^{MC}), speed of sound obtained from REFPROP[10] (c^{REFP}), viscosities computed from MD simulations (η^{MD}), and viscosities obtained from REFPROP[10] (η^{REFP}) of CO_2-N_2 binary mixture with 1 mole% impurity of N_2 at 293 K and pressures ranging from 20 bar to 200 bar.

P/ [bar]	c_V^{MC}/ [J/mol K]	c_V^{REFP}/ [J/mol K]	c_P^{MC}/ [J/mol K]	c_P^{REFP}/ [J/mol K]	c^{MC}/ [m/s]	c^{REFP}/ [m/s]	η^{MD}/ [μPa s]	η^{REFP}/ [μPa s]
20	30.9	31.1	44.5 ± 0.1	45.3	252.8 ± 0.5	250.8	8.0 ± 4.9	15.0
40	34.1	35.3	60.6 ± 0.2	65.0	234.1 ± 0.7	229.5	15.3 ± 0.7	15.7
60	49.1 ± 0.9	–	168.8 ± 15.7	–	268.2 ± 130.0	–	50.8 ± 18.8	33.9
80	40.2 ± 0.1	42.1	132.9 ± 2.1	137.2	474.2 ± 8.0	406.4	77.1 ± 1.6	73.2
100	39.9 ± 0.1	41.2	120.9 ± 2.4	118.2	517.7 ± 10.2	460.0	82.1 ± 2.6	79.6
120	39.8 ± 0.1	40.8	112.5 ± 1.3	108.3	550.4 ± 6.8	501.0	86.5 ± 4.3	84.7
140	39.7	40.5	105.9 ± 1.1	101.9	589.8 ± 6.1	535.1	93.6 ± 4.2	89.1
160	39.7 ± 0.1	40.3	101.4 ± 0.5	97.4	614.4 ± 3.5	564.6	95.6 ± 5.6	93.1
180	39.6 ± 0.1	40.3	98.0 ± 1.9	94.0	641.3 ± 13.2	591.0	114.0 ± 14.9	96.8
200	39.5	40.2	96.3 ± 0.6	91.3	663.8 ± 3.6	614.9	112.2 ± 7.6	100.2

Table S79: Densities computed from MC and MD simulations (ρ^{MC} and ρ^{MD}), densities obtained from REFPROP[10] (ρ^{REFP}), isothermal compressibilities computed from MC simulations (β_T^{MC}), isothermal compressibilities obtained from REFPROP[10] (β_T^{REFP}), thermal expansion coefficients computed from MC simulations (α_P^{MC}), thermal expansion coefficients obtained from REFPROP[10] (α_P^{REFP}), Joule Thomson coefficients computed from MC simulations ($\mu_{\mathrm{JT}}^{\mathrm{MC}}$), and Joule Thomson coefficients obtained from REFPROP[10] ($\mu_{\mathrm{JT}}^{\mathrm{REFP}}$) of CO_2-N_2 binary mixture with 1 mole% impurity of N_2 at 313 K and pressures ranging from 20 bar to 200 bar.

P/ [bar]	ρ^{MC}/ [kg/m^3]	ρ^{MD}/ [kg/m^3]	ρ^{REFP}/ [kg/m^3]	β_T^{MC}/ [10^{-5}/bar]	β_T^{REFP}/ [10^{-5}/bar]	α_P^{MC}/ [10^{-4}/K]	α_P^{REFP}/ [10^{-4}/K]	$\mu_{\mathrm{JT}}^{\mathrm{MC}}$/ [$10^{-3}$ K/bar]	$\mu_{\mathrm{JT}}^{\mathrm{REFP}}$/ [$10^{-3}$ K/bar]
20	36.5	37.3 ± 0.1	37.0	5465.3 ± 10.4	5515.4	42.2 ± 0.1	43.3	884.9 ± 6.8	961.9
40	81.1	83.1 ± 0.3	83.2	3099.4 ± 8.7	3182.7	59.6 ± 0.1	63.5	890.3 ± 4.9	953.6
60	141.0	145.8 ± 1.1	147.5	2554.3 ± 15.2	2704.9	97.7 ± 0.5	109.8	883.8 ± 7.7	920.6
80	238.4 ± 0.6	273.2 ± 7.0	267.3	3399.3 ± 61.9	3644.5	264.7 ± 5.7	320.2	833.7 ± 26.3	–
100	593.2 ± 6.6	588.4 ± 8.5	589.0	1825.7 ± 167.9	1631.5	470.2 ± 29.8	405.0	305.7 ± 24.5	–
120	699.4 ± 2.0	699.7 ± 2.9	696.4	440.4 ± 23.8	469.6	168.9 ± 6.9	165.1	158.6 ± 8.9	167.2
140	747.8 ± 1.4	746.6 ± 1.5	746.8	241.9 ± 6.0	267.8	110.9 ± 2.7	111.9	108.1 ± 4.1	115.4
160	777.0 ± 0.7	777.7 ± 1.7	780.9	173.1 ± 4.5	188.0	88.2 ± 2.1	88.2	83.0 ± 3.3	87.1
180	802.0 ± 0.6	799.9 ± 0.8	807.1	134.1 ± 2.1	145.2	74.6 ± 1.0	74.4	65.8 ± 1.7	68.6
200	821.8 ± 0.7	820.8 ± 0.6	828.5	110.9 ± 1.9	118.5	65.7 ± 0.9	65.1	53.8 ± 1.6	55.2

Table S80: Heat capacities at constant volume computed from MC simulations (c_V^{MC}), heat capacities at constant volume obtained from REFPROP[10] (c_V^{REFP}), heat capacities at constant pressure computed from MC simulations (c_P^{MC}), heat capacities at constant pressure obtained from REFPROP[10] (c_P^{REFP}), speed of sound computed from MC simulations (c^{MC}), speed of sound obtained from REFPROP[10] (c^{REFP}), viscosities computed from MD simulations (η^{MD}), and viscosities obtained from REFPROP[10] (η^{REFP}) of CO_2-N_2 binary mixture with 1 mole% impurity of N_2 at 313 K and pressures ranging from 20 bar to 200 bar.

P/ [bar]	c_V^{MC}/ [J/mol K]	c_V^{REFP}/ [J/mol K]	c_P^{MC}/ [J/mol K]	c_P^{REFP}/ [J/mol K]	c^{MC}/ [m/s]	c^{REFP}/ [m/s]	η^{MD}/ [μPa s]	η^{REFP}/ [μPa s]
20	31.3	31.3	43.5	44.0	264.1 ± 0.3	262.4	13.8 ± 3.0	15.9
40	33.2	33.7	52.6	54.7	251.2 ± 0.4	247.4	16.9 ± 0.6	16.5
60	35.9	37.2	72.4 ± 0.2	78.7	236.5 ± 0.8	230.2	19.6 ± 3.5	17.8
80	40.3 ± 0.1	–	160.7 ± 3.2	–	222.0 ± 3.0	–	21.1 ± 0.4	21.5
100	42.1 ± 0.2	–	331.8 ± 13.6	–	269.8 ± 13.7	–	43.5 ± 3.6	43.5
120	40.5 ± 0.1	42.6	169.4 ± 4.1	156.9	368.6 ± 10.9	335.7	58.6 ± 1.8	56.1
140	39.9 ± 0.2	41.3	134.1 ± 2.3	127.2	431.0 ± 6.5	392.2	64.3 ± 2.5	63.3
160	39.7 ± 0.1	40.7	119.6 ± 1.8	113.4	473.6 ± 7.1	435.4	71.5 ± 4.2	68.8
180	39.4	40.4	110.9 ± 0.9	105.2	511.2 ± 4.6	471.2	77.0 ± 3.8	73.4
200	39.4	40.2	104.8 ± 1.0	99.5	540.5 ± 5.2	502.2	78.9 ± 2.2	77.4

S15.2 Thermodynamic and transport properties data of binary CO_2 mixture with 5 mole% impurity of N_2

Table S81: Densities computed from MC and MD simulations (ρ^{MC} and ρ^{MD}), densities obtained from REFPROP[10] (ρ^{REFP}), isothermal compressibilities computed from MC simulations (β_T^{MC}), isothermal compressibilities obtained from REFPROP[10] (β_T^{REFP}), thermal expansion coefficients computed from MC simulations (α_P^{MC}), thermal expansion coefficients obtained from REFPROP[10] (α_P^{REFP}), Joule Thomson coefficients computed from MC simulations (μ_{JT}^{MC}), and Joule Thomson coefficients obtained from REFPROP[10] (μ_{JT}^{REFP}) of CO_2-N_2 binary mixture with 5 mole% impurity of N_2 at 253 K and pressures ranging from 20 bar to 200 bar.

P/ [bar]	ρ^{MC}/ [kg/m^3]	ρ^{MD}/ [kg/m^3]	ρ^{REFP}/ [kg/m^3]	β_T^{MC}/ [10^{-5}/bar]	β_T^{REFP}/ [10^{-5}/bar]	α_P^{MC}/ [10^{-4}/K]	α_P^{REFP}/ [10^{-4}/K]	μ_{JT}^{MC}/ [10^{-3} K/bar]	μ_{JT}^{REFP}/ [10^{-3} K/bar]
20	49.3	52.2 ± 0.2	50.8	6247.2 ± 19.5	6478.0	73.3 ± 0.3	80.8	1487.3 ± 12.4	1637.2
40	980.2 ± 0.7	1025.6 ± 1.3	729.2	49.9 ± 0.9	-142.0	51.2 ± 1.0	11.2	13.1 ± 1.2	–
60	990.2 ± 0.7	1032.6 ± 0.6	994.5	44.5 ± 0.8	52.0	47.0 ± 0.8	49.6	8.7 ± 0.9	12.0
80	998.4 ± 0.2	1039.6 ± 0.8	1004.3	41.1 ± 0.8	46.8	44.8 ± 0.8	46.3	6.1 ± 1.0	8.2
100	1006.0 ± 0.4	1004.9 ± 0.9	1013.4	38.1 ± 0.3	42.7	42.6 ± 0.5	43.6	3.7 ± 0.5	5.0
120	1014.1 ± 0.3	1013.3 ± 0.8	1021.7	35.8 ± 0.4	39.4	41.3 ± 0.5	41.3	2.1 ± 0.6	2.2
140	1020.7 ± 0.3	1020.6 ± 0.2	1029.5	33.2 ± 0.2	36.6	39.1 ± 0.3	39.3	-0.6 ± 0.4	-0.2
160	1027.2 ± 0.2	1025.8 ± 0.6	1036.8	31.4 ± 0.6	34.2	37.7 ± 0.7	37.7	-2.1 ± 0.9	-2.3
180	1033.4 ± 0.3	1032.7 ± 0.8	1043.7	29.8 ± 0.4	32.1	36.6 ± 0.5	36.2	-3.5 ± 0.5	-4.2
200	1039.2 ± 0.3	1038.8 ± 0.3	1050.2	27.9 ± 0.5	30.3	35.0 ± 0.8	34.9	-5.5 ± 0.9	-5.9

Table S82: Heat capacities at constant volume computed from MC simulations (c_V^{MC}), heat capacities at constant volume obtained from REFPROP[10] (c_V^{REFP}), heat capacities at constant pressure computed from MC simulations (c_P^{MC}), heat capacities at constant pressure obtained from REFPROP[10] (c_P^{REFP}), speed of sound computed from MC simulations (c^{MC}), speed of sound obtained from REFPROP[10] (c^{REFP}), viscosities computed from MD simulations (η^{MD}), and viscosities obtained from REFPROP[10] (η^{REFP}) of CO_2-N_2 binary mixture with 5 mole% impurity of N_2 at 253 K and pressures ranging from 20 bar to 200 bar.

P/ [bar]	c_V^{MC}/ [J/mol K]	c_V^{REFP}/ [J/mol K]	c_P^{MC}/ [J/mol K]	c_P^{REFP}/ [J/mol K]	c^{MC}/ [m/s]	c^{REFP}/ [m/s]	η^{MD}/ [μPa s]	η^{REFP}/ [μPa s]
20	31.3	32.6	50.4 ± 0.1	54.3	228.7 ± 0.4	225.0	12.2 ± 1.0	13.1
40	40.1 ± 0.1	–	98.8 ± 1.3	–	709.7 ± 8.2	–	147.8 ± 2.9	63.8
60	40.1 ± 0.1	40.4	95.0 ± 0.9	92.5	733.4 ± 7.8	665.8	151.4 ± 2.8	126.9
80	39.9 ± 0.1	40.3	93.6 ± 1.1	90.2	756.2 ± 8.8	689.4	156.8 ± 1.8	130.9
100	40.0 ± 0.1	40.3	92.0 ± 0.7	88.2	775.4 ± 4.3	711.1	148.2 ± 13.8	134.7
120	40.0	40.3	91.6 ± 0.8	86.6	794.3 ± 5.2	731.2	151.2 ± 11.5	138.3
140	40.1 ± 0.1	40.3	89.4 ± 0.6	85.2	810.9 ± 3.6	749.9	159.1 ± 26.2	141.9
160	40.1	40.3	88.5 ± 0.9	84.0	827.4 ± 9.5	767.6	142.2 ± 1.6	145.3
180	40.1 ± 0.1	40.3	87.9 ± 0.6	83.0	843.8 ± 5.9	784.3	158.5 ± 15.7	148.6
200	40.2	40.3	86.4 ± 1.1	82.1	861.1 ± 9.6	800.2	155.1 ± 9.1	151.8

Table S83: Densities computed from MC and MD simulations (ρ^{MC} and ρ^{MD}), densities obtained from REFPROP[10] (ρ^{REFP}), isothermal compressibilities computed from MC simulations (β_T^{MC}), isothermal compressibilities obtained from REFPROP[10] (β_T^{REFP}), thermal expansion coefficients computed from MC simulations (α_P^{MC}), thermal expansion coefficients obtained from REFPROP[10] (α_P^{REFP}), Joule Thomson coefficients computed from MC simulations (μ_{JT}^{MC}), and Joule Thomson coefficients obtained from REFPROP[10] (μ_{JT}^{REFP}) of CO_2-N_2 binary mixture with 5 mole% impurity of N_2 at 273 K and pressures ranging from 20 bar to 200 bar.

P/ [bar]	ρ^{MC}/ [kg/m^3]	ρ^{MD}/ [kg/m^3]	ρ^{REFP}/ [kg/m^3]	β_T^{MC}/ [10^{-5}/bar]	β_T^{REFP}/ [10^{-5}/bar]	α_P^{MC}/ [10^{-4}/K]	α_P^{REFP}/ [10^{-4}/K]	μ_{JT}^{MC}/ [10^{-3} K/bar]	μ_{JT}^{REFP}/ [10^{-3} K/bar]
20	43.4	45.5	44.3	5804.6 ± 20.3	5923.0	56.8 ± 0.3	60.1	1201.2 ± 15.7	1323.5
40	108.5 ± 0.1	120.9 ± 0.9	194.1	4189.8 ± 36.1	42 654.7	127.0 ± 1.4	3334.8	1253.0 ± 21.3	–
60	878.2 ± 1.5	933.8 ± 1.4	859.7	111.1 ± 3.4	170.7	79.3 ± 2.2	101.2	49.2 ± 2.7	–
80	896.4 ± 0.8	946.9 ± 0.4	896.1	89.0 ± 3.5	105.9	68.1 ± 2.2	72.3	38.1 ± 2.7	44.5
100	909.8 ± 1.2	908.8 ± 0.5	913.4	74.7 ± 2.2	86.6	60.3 ± 1.6	63.1	29.8 ± 2.0	34.3
120	922.3 ± 0.8	923.2 ± 0.8	928.1	65.2 ± 1.7	73.8	55.0 ± 1.5	56.7	23.7 ± 2.0	26.7
140	933.8 ± 0.9	933.8 ± 0.6	941.0	58.1 ± 1.2	64.5	51.1 ± 0.9	51.8	18.9 ± 1.2	20.7
160	944.2 ± 0.5	943.7 ± 0.7	952.6	52.5 ± 0.6	57.5	47.9 ± 0.4	48.1	14.9 ± 0.6	15.8
180	953.7 ± 0.3	953.4 ± 0.8	963.0	47.9 ± 0.4	52.0	44.9 ± 0.4	45.0	11.2 ± 0.5	11.7
200	962.3 ± 0.4	961.0 ± 0.7	972.6	44.3 ± 0.9	47.5	42.9 ± 1.0	42.4	8.5 ± 1.3	8.2

Table S84: Heat capacities at constant volume computed from MC simulations (c_V^{MC}), heat capacities at constant volume obtained from REFPROP[10] (c_V^{REFP}), heat capacities at constant pressure computed from MC simulations (c_P^{MC}), heat capacities at constant pressure obtained from REFPROP[10] (c_P^{REFP}), speed of sound computed from MC simulations (c^{MC}), speed of sound obtained from REFPROP[10] (c^{REFP}), viscosities computed from MD simulations (η^{MD}), and viscosities obtained from REFPROP[10] (η^{REFP}) of CO_2-N_2 binary mixture with 5 mole% impurity of N_2 at 273 K and pressures ranging from 20 bar to 200 bar.

P/ [bar]	c_V^{MC}/ [J/mol K]	c_V^{REFP}/ [J/mol K]	c_P^{MC}/ [J/mol K]	c_P^{REFP}/ [J/mol K]	c^{MC}/ [m/s]	c^{REFP}/ [m/s]	η^{MD}/ [μPa s]	η^{REFP}/ [μPa s]
20	30.5	30.9	45.6 ± 0.1	47.2	243.7 ± 0.5	241.1	3.3 ± 4.6	14.1
40	36.1 ± 0.1	–	78.4 ± 0.6	–	218.4 ± 1.3	–	17.4 ± 3.0	17.0
60	39.9 ± 0.1	–	116.6 ± 2.2	–	547.3 ± 9.9	–	112.3 ± 3.4	84.8
80	39.7 ± 0.1	40.5	108.7 ± 1.9	105.6	585.9 ± 12.5	524.2	117.4 ± 8.4	93.7
100	39.6 ± 0.1	40.2	103.0 ± 1.6	99.6	618.5 ± 10.3	559.3	102.6 ± 5.0	98.3
120	39.5 ± 0.2	40.0	99.0 ± 1.7	95.3	645.4 ± 10.1	589.5	103.9 ± 3.1	102.5
140	39.4 ± 0.1	39.9	96.4 ± 1.0	92.1	671.0 ± 7.6	616.3	109.8 ± 4.0	106.4
160	39.4 ± 0.1	39.9	94.2 ± 0.5	89.6	694.0 ± 4.6	640.5	113.0 ± 4.5	110.0
180	39.4 ± 0.1	39.8	91.8 ± 0.4	87.5	714.2 ± 3.5	662.7	116.2 ± 10.9	113.4
200	39.5 ± 0.1	39.8	90.6 ± 1.3	85.8	734.1 ± 9.0	683.3	116.6 ± 3.9	116.7

Table S85: Densities computed from MC and MD simulations (ρ^{MC} and ρ^{MD}), densities obtained from REFPROP[10] (ρ^{REFP}), isothermal compressibilities computed from MC simulations (β_T^{MC}), isothermal compressibilities obtained from REFPROP[10] (β_T^{REFP}), thermal expansion coefficients computed from MC simulations (α_P^{MC}), thermal expansion coefficients obtained from REFPROP[10] (α_P^{REFP}), Joule Thomson coefficients computed from MC simulations ($\mu_{\mathrm{JT}}^{\mathrm{MC}}$), and Joule Thomson coefficients obtained from REFPROP[10] ($\mu_{\mathrm{JT}}^{\mathrm{REFP}}$) of CO_2-N_2 binary mixture with 5 mole% impurity of N_2 at 293 K and pressures ranging from 20 bar to 200 bar.

P/ [bar]	ρ^{MC}/ [kg/m^3]	ρ^{MD}/ [kg/m^3]	ρ^{REFP}/ [kg/m^3]	β_T^{MC}/ [10^{-5}/bar]	β_T^{REFP}/ [10^{-5}/bar]	α_P^{MC}/ [10^{-4}/K]	α_P^{REFP}/ [10^{-4}/K]	$\mu_{\mathrm{JT}}^{\mathrm{MC}}$/ [$10^{-3}$ K/bar]	$\mu_{\mathrm{JT}}^{\mathrm{REFP}}$/ [$10^{-3}$ K/bar]
20	39.1	40.8 ± 0.1	39.7	5580.3 ± 11.0	5647.6	47.6 ± 0.1	49.4	999.3 ± 7.6	1097.2
40	89.8	95.7 ± 0.5	92.9	3340.5 ± 18.7	3471.4	75.3 ± 0.5	82.7	1014.6 ± 12.7	1083.1
60	170.4 ± 0.3	457.6 ± 199.7	185.9	3457.2 ± 31.9	4037.9	180.1 ± 1.6	236.8	995.6 ± 12.8	1009.1
80	722.6 ± 2.3	814.6 ± 1.8	700.4	493.2 ± 18.7	751.8	200.4 ± 5.3	257.5	156.1 ± 5.7	197.8
100	771.9 ± 1.5	771.9 ± 1.4	766.2	240.8 ± 10.8	294.2	120.3 ± 4.0	129.7	101.2 ± 5.1	115.9
120	802.5 ± 1.1	803.1 ± 1.1	802.8	166.4 ± 1.8	189.1	93.1 ± 0.7	95.4	76.0 ± 1.0	82.8
140	826.3 ± 0.6	826.5 ± 0.7	829.3	123.3 ± 2.8	140.9	75.1 ± 1.5	78.2	57.1 ± 2.3	63.2
160	844.0 ± 0.7	843.6 ± 1.1	850.4	101.1 ± 1.9	113.0	65.9 ± 1.2	67.5	45.9 ± 1.8	49.7
180	860.1 ± 0.8	860.1 ± 1.1	868.2	87.4 ± 2.8	94.6	60.3 ± 1.4	60.1	38.2 ± 2.1	39.7
200	874.6 ± 0.9	874.1 ± 0.5	883.6	76.5 ± 1.6	81.6	55.2 ± 1.0	54.6	31.4 ± 1.5	31.9

Table S86: Heat capacities at constant volume computed from MC simulations (c_V^{MC}), heat capacities at constant volume obtained from REFPROP[10] (c_V^{REFP}), heat capacities at constant pressure computed from MC simulations (c_P^{MC}), heat capacities at constant pressure obtained from REFPROP[10] (c_P^{REFP}), speed of sound computed from MC simulations (c^{MC}), speed of sound obtained from REFPROP[10] (c^{REFP}), viscosities computed from MD simulations (η^{MD}), and viscosities obtained from REFPROP[10] (η^{REFP}) of CO_2-N_2 binary mixture with 5 mole% impurity of N_2 at 293 K and pressures ranging from 20 bar to 200 bar.

P/ [bar]	c_V^{MC}/ [J/mol K]	c_V^{REFP}/ [J/mol K]	c_P^{MC}/ [J/mol K]	c_P^{REFP}/ [J/mol K]	c^{MC}/ [m/s]	c^{REFP}/ [m/s]	η^{MD}/ [μPa s]	η^{REFP}/ [μPa s]
20	30.5	30.7	43.6	44.5	256.1 ± 0.3	254.2	8.0 ± 4.9	15.1
40	33.2	34.3	57.3 ± 0.2	61.1	239.7 ± 0.8	235.1	15.3 ± 0.7	15.8
60	38.6 ± 0.1	42.2	108.9 ± 0.7	136.8	218.9 ± 1.3	207.8	31.4 ± 18.7	17.9
80	41.0 ± 0.2	44.7	186.6 ± 3.2	204.2	357.5 ± 7.5	294.4	77.1 ± 1.6	56.6
100	40.0 ± 0.1	41.8	139.7 ± 2.8	136.2	433.6 ± 10.7	380.3	73.8 ± 8.4	66.6
120	39.7	40.8	122.4 ± 0.7	116.6	480.6 ± 2.9	434.2	79.3 ± 6.5	73.0
140	39.3 ± 0.1	40.2	110.1 ± 1.4	106.4	524.0 ± 6.8	475.7	81.7 ± 7.0	78.2
160	39.2 ± 0.1	39.9	104.0 ± 1.2	100.0	557.5 ± 6.2	510.3	84.1 ± 4.0	82.6
180	39.1 ± 0.1	39.8	100.6 ± 1.1	95.4	585.4 ± 10.0	540.4	88.6 ± 5.1	86.6
200	39.0 ± 0.1	39.6	96.9 ± 1.0	91.9	609.6 ± 7.1	567.3	102.2 ± 19.7	90.2

Table S87: Densities computed from MC and MD simulations (ρ^{MC} and ρ^{MD}), densities obtained from REFPROP[10] (ρ^{REFP}), isothermal compressibilities computed from MC simulations (β_T^{MC}), isothermal compressibilities obtained from REFPROP[10] (β_T^{REFP}), thermal expansion coefficients computed from MC simulations (α_P^{MC}), thermal expansion coefficients obtained from REFPROP[10] (α_P^{REFP}), Joule Thomson coefficients computed from MC simulations (μ_{JT}^{MC}), and Joule Thomson coefficients obtained from REFPROP[10] (μ_{JT}^{REFP}) of CO_2-N_2 binary mixture with 5 mole% impurity of N_2 at 313 K and pressures ranging from 20 bar to 200 bar.

P/ [bar]	ρ^{MC}/ [kg/m^3]	ρ^{MD}/ [kg/m^3]	ρ^{REFP}/ [kg/m^3]	β_T^{MC}/ [10^{-5}/bar]	β_T^{REFP}/ [10^{-5}/bar]	α_P^{MC}/ [10^{-4}/K]	α_P^{REFP}/ [10^{-4}/K]	μ_{JT}^{MC}/ [10^{-3} K/bar]	μ_{JT}^{REFP}/ [10^{-3} K/bar]
20	35.8	37.3 ± 0.1	36.2	5435.6 ± 10.9	5480.4	41.5 ± 0.1	42.7	847.8 ± 8.4	925.2
40	78.8	83.1 ± 0.3	80.8	3037.0 ± 10.2	3114.0	57.0 ± 0.2	60.6	844.9 ± 6.6	908.6
60	134.1 ± 0.1	145.8 ± 1.1	140.0	2424.2 ± 9.0	2529.0	87.5 ± 0.3	96.7	838.2 ± 5.6	872.0
80	216.6 ± 0.3	275.5 ± 6.9	232.9	2488.6 ± 49.8	2692.6	168.4 ± 3.1	198.1	774.7 ± 20.1	770.9
100	392.6 ± 1.0	411.8 ± 4.6	421.8	3247.9 ± 78.5	2790.9	487.6 ± 12.2	434.9	523.9 ± 18.1	491.1
120	588.0 ± 2.8	590.7 ± 2.3	592.0	1017.2 ± 36.4	917.9	276.7 ± 7.1	238.3	259.1 ± 8.6	255.2
140	669.2 ± 1.6	668.9 ± 3.5	670.7	420.1 ± 15.1	432.2	151.4 ± 5.6	144.4	159.3 ± 8.7	163.3
160	712.2 ± 1.1	712.2 ± 1.6	717.9	260.9 ± 10.0	271.5	109.6 ± 3.0	106.2	115.6 ± 4.7	117.7
180	745.3 ± 0.3	744.1 ± 2.1	751.8	184.8 ± 4.8	196.7	87.1 ± 2.2	86.1	87.8 ± 3.8	90.3
200	769.8 ± 1.0	769.4 ± 1.8	778.3	147.4 ± 3.0	153.8	75.4 ± 1.1	73.5	71.3 ± 1.9	71.6

Table S88: Heat capacities at constant volume computed from MC simulations (c_V^{MC}), heat capacities at constant volume obtained from REFPROP[10] (c_V^{REFP}), heat capacities at constant pressure computed from MC simulations (c_P^{MC}), heat capacities at constant pressure obtained from REFPROP[10] (c_P^{REFP}), speed of sound computed from MC simulations (c^{MC}), speed of sound obtained from REFPROP[10] (c^{REFP}), viscosities computed from MD simulations (η^{MD}), and viscosities obtained from REFPROP[10] (η^{REFP}) of CO_2-N_2 binary mixture with 5 mole% impurity of N_2 at 313 K and pressures ranging from 20 bar to 200 bar.

P/ [bar]	c_V^{MC}/ [J/mol K]	c_V^{REFP}/ [J/mol K]	c_P^{MC}/ [J/mol K]	c_P^{REFP}/ [J/mol K]	c^{MC}/ [m/s]	c^{REFP}/ [m/s]	η^{MD}/ [μPa s]	η^{REFP}/ [μPa s]
20	30.8	30.9	42.8	43.3	267.3 ± 0.3	265.7	13.8 ± 3.0	16.1
40	32.5	33.1	50.9 ± 0.1	52.9	255.8 ± 0.5	252.0	16.9 ± 0.6	16.7
60	34.9	36.0	66.8 ± 0.2	71.8	242.7 ± 0.6	237.2	19.6 ± 3.5	17.8
80	38.3 ± 0.1	40.5	110.0 ± 1.4	125.1	230.7 ± 2.7	221.9	20.6 ± 0.6	20.5
100	42.4 ± 0.3	45.8	299.6 ± 6.4	263.1	235.3 ± 3.9	220.9	28.0 ± 2.3	29.9
120	40.9 ± 0.2	43.4	217.3 ± 3.4	184.7	297.9 ± 5.9	279.9	45.2 ± 3.3	44.2
140	39.9 ± 0.1	41.5	151.5 ± 4.2	138.8	367.3 ± 8.3	339.5	49.8 ± 1.7	53.3
160	39.4 ± 0.1	40.6	127.6 ± 1.8	118.8	417.4 ± 8.5	387.4	61.8 ± 6.4	59.7
180	39.2 ± 0.1	40.1	114.0 ± 1.9	107.9	459.7 ± 7.1	426.6	68.2 ± 3.1	64.8
200	38.9 ± 0.1	39.8	107.2 ± 0.8	100.9	492.7 ± 5.4	460.3	70.7 ± 2.0	69.1

S15.3 Thermodynamic and transport properties data of binary CO_2 mixture with 10 mole% impurity of N_2

Table S89: Densities computed from MC and MD simulations (ρ^{MC} and ρ^{MD}), densities obtained from REFPROP[10] (ρ^{REFP}), isothermal compressibilities computed from MC simulations (β_T^{MC}), isothermal compressibilities obtained from REFPROP[10] (β_T^{REFP}), thermal expansion coefficients computed from MC simulations (α_P^{MC}), thermal expansion coefficients obtained from REFPROP[10] (α_P^{REFP}), Joule Thomson coefficients computed from MC simulations (μ_{JT}^{MC}), and Joule Thomson coefficients obtained from REFPROP[10] (μ_{JT}^{REFP}) of CO_2-N_2 binary mixture with 10 mole% impurity of N_2 at 253 K and pressures ranging from 20 bar to 200 bar.

P/ [bar]	ρ^{MC}/ [kg/m^3]	ρ^{MD}/ [kg/m^3]	ρ^{REFP}/ [kg/m^3]	β_T^{MC}/ [10^{-5}/bar]	β_T^{REFP}/ [10^{-5}/bar]	α_P^{MC}/ [10^{-4}/K]	α_P^{REFP}/ [10^{-4}/K]	μ_{JT}^{MC}/ [10^{-3} K/bar]	μ_{JT}^{REFP}/ [10^{-3} K/bar]
20	47.6	52.2 ± 0.2	48.9	6098.9 ± 42.6	6281.1	69.1 ± 0.6	75.2	1387.1 ± 27.1	1532.1
40	916.2 ± 0.8	1025.6 ± 1.3	386.4	76.7 ± 2.0	2094.1	64.8 ± 1.5	2667.8	28.4 ± 1.7	–
60	928.8 ± 0.5	1032.6 ± 0.6	729.6	66.2 ± 2.1	−431.0	58.7 ± 1.5	−85.7	22.1 ± 1.8	–
80	940.4 ± 0.5	1039.6 ± 0.8	946.4	58.6 ± 1.5	66.9	54.2 ± 1.4	55.9	17.2 ± 1.7	20.1
100	950.5 ± 0.2	950.3 ± 0.7	958.3	52.7 ± 1.4	58.7	50.4 ± 1.2	51.2	13.0 ± 1.5	14.7
120	959.8 ± 0.5	959.6 ± 0.7	969.0	47.2 ± 0.5	52.5	46.6 ± 0.6	47.6	8.6 ± 0.7	10.3
140	969.9 ± 0.5	969.2 ± 0.2	978.7	43.2 ± 0.5	47.6	44.5 ± 0.5	44.6	6.1 ± 0.6	6.6
160	977.2 ± 0.6	976.3 ± 0.5	987.7	39.9 ± 0.5	43.6	42.2 ± 0.6	42.2	3.3 ± 0.7	3.4
180	985.0 ± 0.4	983.6 ± 0.8	996.0	37.0 ± 1.0	40.3	40.0 ± 0.9	40.1	0.6 ± 1.2	0.7
200	991.9 ± 0.4	991.6 ± 0.9	1003.8	35.0 ± 0.8	37.5	38.8 ± 0.8	38.3	-1.0 ± 1.0	−1.6

Table S90: Heat capacities at constant volume computed from MC simulations (c_V^{MC}), heat capacities at constant volume obtained from REFPROP[10] (c_V^{REFP}), heat capacities at constant pressure computed from MC simulations (c_P^{MC}), heat capacities at constant pressure obtained from REFPROP[10] (c_P^{REFP}), speed of sound computed from MC simulations (c^{MC}), speed of sound obtained from REFPROP[10] (c^{REFP}), viscosities computed from MD simulations (η^{MD}), and viscosities obtained from REFPROP[10] (η^{REFP}) of CO_2-N_2 binary mixture with 10 mole% impurity of N_2 at 253 K and pressures ranging from 20 bar to 200 bar.

P/ [bar]	c_V^{MC}/ [J/mol K]	c_V^{REFP}/ [J/mol K]	c_P^{MC}/ [J/mol K]	c_P^{REFP}/ [J/mol K]	c^{MC}/ [m/s]	c^{REFP}/ [m/s]	η^{MD}/ [μPa s]	η^{REFP}/ [μPa s]
20	30.4	31.3	48.1 ± 0.2	51.1	233.5 ± 0.9	230.3	12.2 ± 1.0	13.3
40	40.0 ± 0.1	–	104.3 ± 1.3	–	609.4 ± 8.9	–	147.8 ± 2.9	24.9
60	39.7 ± 0.1	–	100.2 ± 1.3	–	640.6 ± 11.0	–	151.4 ± 2.8	62.5
80	39.6 ± 0.2	39.7	97.1 ± 1.6	92.6	667.2 ± 10.2	607.3	156.8 ± 1.8	111.6
100	39.6 ± 0.1	39.5	94.3 ± 1.2	89.6	689.8 ± 10.4	634.6	117.2 ± 6.9	115.7
120	39.5 ± 0.1	39.4	91.1 ± 0.9	87.2	714.3 ± 5.2	659.3	129.4 ± 14.5	119.6
140	39.4	39.4	90.2 ± 0.6	85.2	739.1 ± 5.3	681.8	123.6 ± 3.7	123.2
160	39.5 ± 0.1	39.3	88.6 ± 1.0	83.6	758.4 ± 6.4	702.7	133.7 ± 7.7	126.7
180	39.4 ± 0.1	39.3	86.8 ± 1.1	82.2	777.2 ± 11.4	722.1	140.2 ± 18.0	130.0
200	39.5 ± 0.1	39.3	86.2 ± 0.8	81.0	793.5 ± 10.1	740.4	143.5 ± 14.3	133.3

Table S91: Densities computed from MC and MD simulations (ρ^{MC} and ρ^{MD}), densities obtained from REFPROP[10] (ρ^{REFP}), isothermal compressibilities computed from MC simulations (β_T^{MC}), isothermal compressibilities obtained from REFPROP[10] (β_T^{REFP}), thermal expansion coefficients computed from MC simulations (α_P^{MC}), thermal expansion coefficients obtained from REFPROP[10] (α_P^{REFP}), Joule Thomson coefficients computed from MC simulations ($\mu_{\mathrm{JT}}^{\mathrm{MC}}$), and Joule Thomson coefficients obtained from REFPROP[10] ($\mu_{\mathrm{JT}}^{\mathrm{REFP}}$) of CO_2-N_2 binary mixture with 10 mole% impurity of N_2 at 273 K and pressures ranging from 20 bar to 200 bar.

P/ [bar]	ρ^{MC}/ [kg/m^3]	ρ^{MD}/ [kg/m^3]	ρ^{REFP}/ [kg/m^3]	β_T^{MC}/ [10^{-5}/bar]	β_T^{REFP}/ [10^{-5}/bar]	α_P^{MC}/ [10^{-4}/K]	α_P^{REFP}/ [10^{-4}/K]	$\mu_{\mathrm{JT}}^{\mathrm{MC}}$/ [$10^{-3}$ K/bar]	$\mu_{\mathrm{JT}}^{\mathrm{REFP}}$/ [$10^{-3}$ K/bar]
20	42.1	45.5	42.9	5713.0 ± 10.8	5828.3	54.7 ± 0.1	57.8	1121.7 ± 6.8	1248.3
40	101.7 ± 0.1	120.9 ± 0.9	107.2	3792.0 ± 35.4	4069.9	105.9 ± 1.1	123.3	1156.3 ± 19.9	1221.8
60	785.2 ± 1.0	933.8 ± 1.4	449.3	276.0 ± 20.2	2887.3	141.4 ± 7.2	461.1	103.2 ± 7.7	–
80	818.7 ± 1.4	946.9 ± 0.4	787.2	158.7 ± 3.5	300.3	95.3 ± 1.7	141.2	68.2 ± 2.1	–
100	840.9 ± 1.5	840.2 ± 2.3	842.3	121.0 ± 3.9	142.9	79.2 ± 1.9	83.7	52.6 ± 2.4	60.2
120	858.7 ± 1.0	857.5 ± 0.9	863.8	97.0 ± 1.8	111.5	67.8 ± 1.2	70.8	40.5 ± 1.6	45.8
140	873.9 ± 0.8	874.2 ± 1.6	881.4	83.5 ± 2.5	92.0	61.9 ± 1.5	62.3	33.4 ± 2.0	35.5
160	886.9 ± 0.8	887.7 ± 1.6	896.5	71.8 ± 1.5	78.6	55.9 ± 1.0	56.2	26.2 ± 1.3	27.7
180	899.7 ± 0.7	898.8 ± 0.5	909.8	63.2 ± 0.6	68.8	51.4 ± 0.3	51.5	20.4 ± 0.5	21.5
200	910.0 ± 0.5	909.1 ± 0.9	921.7	57.1 ± 1.3	61.4	48.0 ± 0.9	47.8	16.0 ± 1.3	16.4

Table S92: Heat capacities at constant volume computed from MC simulations (c_V^{MC}), heat capacities at constant volume obtained from REFPROP[10] (c_V^{REFP}), heat capacities at constant pressure computed from MC simulations (c_P^{MC}), heat capacities at constant pressure obtained from REFPROP[10] (c_P^{REFP}), speed of sound computed from MC simulations (c^{MC}), speed of sound obtained from REFPROP[10] (c^{REFP}), viscosities computed from MD simulations (η^{MD}), and viscosities obtained from REFPROP[10] (η^{REFP}) of CO_2-N_2 binary mixture with 10 mole% impurity of N_2 at 273 K and pressures ranging from 20 bar to 200 bar.

P/ [bar]	c_V^{MC}/ [J/mol K]	c_V^{REFP}/ [J/mol K]	c_P^{MC}/ [J/mol K]	c_P^{REFP}/ [J/mol K]	c^{MC}/ [m/s]	c^{REFP}/ [m/s]	η^{MD}/ [μPa s]	η^{REFP}/ [μPa s]
20	29.8	30.3	44.2	45.7	248.3 ± 0.3	245.7	3.3 ± 4.6	14.3
40	34.5	36.3	68.2 ± 0.4	76.6	226.5 ± 1.2	220.0	17.4 ± 3.0	15.2
60	40.5 ± 0.3	–	149.7 ± 4.2	–	413.2 ± 16.2	–	112.3 ± 3.4	30.1
80	39.7 ± 0.1	–	121.5 ± 1.4	–	485.3 ± 6.0	–	117.4 ± 8.4	71.1
100	39.4	40.1	111.4 ± 1.3	107.5	526.9 ± 9.1	472.1	87.9 ± 7.6	82.2
120	39.1 ± 0.1	39.6	103.5 ± 1.0	99.8	563.8 ± 6.0	511.6	86.9 ± 2.7	87.1
140	39.0 ± 0.1	39.3	100.4 ± 1.2	94.7	593.6 ± 9.7	544.9	93.6 ± 4.6	91.5
160	38.8 ± 0.1	39.2	96.1 ± 1.0	91.0	623.5 ± 7.2	574.0	97.9 ± 5.3	95.4
180	38.9 ± 0.1	39.0	92.7 ± 0.3	88.1	647.2 ± 3.2	600.2	102.6 ± 7.1	99.0
200	38.8 ± 0.1	39.0	90.5 ± 0.8	85.8	670.0 ± 8.2	624.0	102.7 ± 4.2	102.4

Table S93: Densities computed from MC and MD simulations (ρ^{MC} and ρ^{MD}), densities obtained from REFPROP[10] (ρ^{REFP}), isothermal compressibilities computed from MC simulations (β_T^{MC}), isothermal compressibilities obtained from REFPROP[10] (β_T^{REFP}), thermal expansion coefficients computed from MC simulations (α_P^{MC}), thermal expansion coefficients obtained from REFPROP[10] (α_P^{REFP}), Joule Thomson coefficients computed from MC simulations (μ_{JT}^{MC}), and Joule Thomson coefficients obtained from REFPROP[10] (μ_{JT}^{REFP}) of CO_2-N_2 binary mixture with 10 mole% impurity of N_2 at 293 K and pressures ranging from 20 bar to 200 bar.

P/ [bar]	ρ^{MC}/ [kg/m^3]	ρ^{MD}/ [kg/m^3]	ρ^{REFP}/ [kg/m^3]	β_T^{MC}/ [10^{-5}/bar]	β_T^{REFP}/ [10^{-5}/bar]	α_P^{MC}/ [10^{-4}/K]	α_P^{REFP}/ [10^{-4}/K]	μ_{JT}^{MC}/ [10^{-3} K/bar]	μ_{JT}^{REFP}/ [10^{-3} K/bar]
20	38.1	40.8 ± 0.1	38.6	5520.3 ± 11.2	5588.0	46.4 ± 0.1	48.2	938.0 ± 8.0	1039.1
40	85.9	95.7 ± 0.5	88.7	3219.0 ± 20.5	3322.3	69.6 ± 0.4	75.5	948.2 ± 12.0	1012.7
60	154.7 ± 0.2	457.6 ± 199.7	165.1	2892.2 ± 13.8	3132.0	132.4 ± 1.0	156.1	926.0 ± 10.6	949.1
80	336.7 ± 6.5	815.1 ± 1.9	389.5	7163.8 ± 495.7	5849.0	948.6 ± 86.2	857.5	646.4 ± 85.1	–
100	643.5 ± 3.6	645.1 ± 1.1	637.3	823.6 ± 61.4	845.2	265.2 ± 13.8	248.5	212.7 ± 14.5	223.9
120	712.7 ± 1.2	713.6 ± 3.6	710.3	322.9 ± 4.9	364.5	136.9 ± 1.9	138.5	126.0 ± 2.7	136.9
140	748.8 ± 1.3	748.7 ± 2.6	752.3	209.9 ± 7.7	230.5	101.5 ± 3.1	101.8	92.1 ± 4.7	97.6
160	776.6 ± 1.5	775.9 ± 1.7	782.4	149.4 ± 1.8	168.7	80.4 ± 1.2	82.8	68.3 ± 1.8	74.3
180	798.1 ± 0.9	797.0 ± 0.5	806.2	121.4 ± 1.7	133.3	70.3 ± 0.8	71.1	55.0 ± 1.3	58.3
200	815.9 ± 0.7	815.5 ± 1.6	825.9	102.8 ± 1.8	110.2	63.3 ± 1.2	62.9	45.3 ± 1.9	46.6

Table S94: Heat capacities at constant volume computed from MC simulations (c_V^{MC}), heat capacities at constant volume obtained from REFPROP[10] (c_V^{REFP}), heat capacities at constant pressure computed from MC simulations (c_P^{MC}), heat capacities at constant pressure obtained from REFPROP[10] (c_P^{REFP}), speed of sound computed from MC simulations (c^{MC}), speed of sound obtained from REFPROP[10] (c^{REFP}), viscosities computed from MD simulations (η^{MD}), and viscosities obtained from REFPROP[10] (η^{REFP}) of CO_2-N_2 binary mixture with 10 mole% impurity of N_2 at 293 K and pressures ranging from 20 bar to 200 bar.

P/ [bar]	c_V^{MC}/ [J/mol K]	c_V^{REFP}/ [J/mol K]	c_P^{MC}/ [J/mol K]	c_P^{REFP}/ [J/mol K]	c^{MC}/ [m/s]	c^{REFP}/ [m/s]	η^{MD}/ [μPa s]	η^{REFP}/ [μPa s]
20	29.9	30.1	42.6	43.5	260.5 ± 0.3	258.5	8.0 ± 4.9	15.3
40	32.3	33.2	54.1 ± 0.1	57.2	246.1 ± 0.8	241.8	15.3 ± 0.7	16.0
60	36.2 ± 0.1	38.2	85.3 ± 0.5	96.7	229.4 ± 0.9	221.4	31.4 ± 18.7	17.6
80	45.2 ± 0.4	–	522.6 ± 47.0	–	219.0 ± 12.6	–	76.7 ± 3.4	27.1
100	41.0 ± 0.2	44.2	209.7 ± 6.8	186.6	310.6 ± 12.7	280.0	51.6 ± 2.3	49.1
120	39.7 ± 0.2	41.3	142.2 ± 1.5	133.4	394.3 ± 3.8	353.2	59.3 ± 2.3	58.7
140	39.1 ± 0.1	40.2	121.4 ± 2.3	114.4	444.2 ± 9.2	405.2	67.2 ± 3.4	65.2
160	38.8 ± 0.2	39.6	108.5 ± 1.0	104.2	490.9 ± 3.8	446.5	71.5 ± 4.0	70.3
180	38.7 ± 0.1	39.2	102.5 ± 0.8	97.6	522.9 ± 4.1	481.3	74.9 ± 4.4	74.7
200	38.5 ± 0.1	39.0	98.1 ± 1.3	93.0	550.9 ± 6.0	511.8	81.1 ± 3.4	78.6

Table S95: Densities computed from MC and MD simulations (ρ^{MC} and ρ^{MD}), densities obtained from REFPROP[10] (ρ^{REFP}), isothermal compressibilities computed from MC simulations (β_T^{MC}), isothermal compressibilities obtained from REFPROP[10] (β_T^{REFP}), thermal expansion coefficients computed from MC simulations (α_P^{MC}), thermal expansion coefficients obtained from REFPROP[10] (α_P^{REFP}), Joule Thomson coefficients computed from MC simulations (μ_{JT}^{MC}), and Joule Thomson coefficients obtained from REFPROP[10] (μ_{JT}^{REFP}) of CO_2-N_2 binary mixture with 10 mole% impurity of N_2 at 313 K and pressures ranging from 20 bar to 200 bar.

P/ [bar]	ρ^{MC}/ [kg/m^3]	ρ^{MD}/ [kg/m^3]	ρ^{REFP}/ [kg/m^3]	β_T^{MC}/ [10^{-5}/bar]	β_T^{REFP}/ [10^{-5}/bar]	α_P^{MC}/ [10^{-4}/K]	α_P^{REFP}/ [10^{-4}/K]	μ_{JT}^{MC}/ [10^{-3} K/bar]	μ_{JT}^{REFP}/ [10^{-3} K/bar]
20	34.9	37.3 ± 0.1	35.3	5380.6 ± 4.6	5438.4	40.6 ± 0.1	41.9	789.1 ± 4.9	878.5
40	76.1	83.1 ± 0.3	77.9	2979.6 ± 12.7	3038.5	54.3 ± 0.2	57.4	796.4 ± 8.5	854.2
60	127.0	145.8 ± 1.1	132.0	2273.7 ± 19.1	2367.7	77.4 ± 0.7	85.0	775.0 ± 11.5	814.8
80	195.4 ± 0.1	275.5 ± 6.9	207.6	2083.7 ± 33.7	2223.3	123.9 ± 1.9	141.8	721.3 ± 16.1	733.5
100	299.9 ± 1.2	314.5 ± 2.0	325.0	2169.8 ± 29.7	2202.6	228.5 ± 4.1	249.9	592.0 ± 14.9	565.6
120	447.9 ± 1.2	455.9 ± 4.0	468.9	1630.7 ± 43.8	1354.9	295.6 ± 7.2	249.5	387.6 ± 13.2	357.1
140	560.0 ± 1.1	562.7 ± 5.6	570.3	739.2 ± 19.8	697.1	193.4 ± 5.3	175.4	237.5 ± 9.2	231.8
160	627.1 ± 0.8	626.6 ± 1.8	635.0	423.2 ± 12.9	414.6	138.4 ± 3.8	128.2	165.9 ± 6.6	163.3
180	670.4 ± 1.2	670.2 ± 2.2	679.7	278.0 ± 9.5	281.6	105.3 ± 2.8	100.8	122.8 ± 5.1	122.1
200	703.4 ± 0.8	701.4 ± 1.7	713.5	199.0 ± 4.8	210.1	84.9 ± 1.7	84.1	93.3 ± 3.2	95.2

Table S96: Heat capacities at constant volume computed from MC simulations (c_V^{MC}), heat capacities at constant volume obtained from REFPROP[10] (c_V^{REFP}), heat capacities at constant pressure computed from MC simulations (c_P^{MC}), heat capacities at constant pressure obtained from REFPROP[10] (c_P^{REFP}), speed of sound computed from MC simulations (c^{MC}), speed of sound obtained from REFPROP[10] (c^{REFP}), viscosities computed from MD simulations (η^{MD}), and viscosities obtained from REFPROP[10] (η^{REFP}) of CO_2-N_2 binary mixture with 10 mole% impurity of N_2 at 313 K and pressures ranging from 20 bar to 200 bar.

P/ [bar]	c_V^{MC}/ [J/mol K]	c_V^{REFP}/ [J/mol K]	c_P^{MC}/ [J/mol K]	c_P^{REFP}/ [J/mol K]	c^{MC}/ [m/s]	c^{REFP}/ [m/s]	η^{MD}/ [μPa s]	η^{REFP}/ [μPa s]
20	30.2	30.3	41.9	42.5	271.7 ± 0.1	269.9	13.8 ± 3.0	16.3
40	31.7	32.3	49.0 ± 0.1	50.8	261.1 ± 0.6	257.8	16.9 ± 0.6	16.8
60	33.7	34.8	61.3 ± 0.2	65.4	250.9 ± 1.2	245.4	19.6 ± 3.5	17.9
80	36.3 ± 0.2	37.9	86.6 ± 0.7	95.8	242.0 ± 2.3	233.9	20.6 ± 0.6	19.9
100	39.4 ± 0.2	41.6	146.9 ± 2.0	157.4	239.5 ± 2.4	230.0	25.1 ± 4.4	24.6
120	40.6 ± 0.1	42.4	201.6 ± 4.1	172.5	260.7 ± 4.4	253.0	30.5 ± 3.0	33.6
140	39.9 ± 0.1	41.3	161.1 ± 3.3	144.1	312.2 ± 5.3	296.1	41.3 ± 2.4	42.5
160	39.0 ± 0.1	40.3	135.9 ± 2.5	123.2	362.1 ± 6.5	340.6	49.5 ± 3.3	49.5
180	38.7 ± 0.1	39.7	118.4 ± 1.8	110.1	405.4 ± 7.7	380.8	54.2 ± 2.4	55.1
200	38.4 ± 0.1	39.2	107.1 ± 1.2	101.8	446.2 ± 5.9	416.0	61.2 ± 9.0	59.8

S15.4 Thermodynamic and Transport Properties Data of binary CO_2 mixture with 1 mole% Impurity of Ar

Table S97: Densities computed from MC and MD simulations (ρ^{MC} and ρ^{MD}), densities obtained from REFPROP[10] (ρ^{REFP}), isothermal compressibilities computed from MC simulations (β_T^{MC}), isothermal compressibilities obtained from REFPROP[10] (β_T^{REFP}), thermal expansion coefficients computed from MC simulations (α_P^{MC}), thermal expansion coefficients obtained from REFPROP[10] (α_P^{REFP}), Joule Thomson coefficients computed from MC simulations (μ_{JT}^{MC}), and Joule Thomson coefficients obtained from REFPROP[10] (μ_{JT}^{REFP}) of CO_2-Ar binary mixture with 1 mole% impurity of Ar at 253 K and pressures ranging from 20 bar to 200 bar.

P/ [bar]	ρ^{MC}/ [kg/m^3]	ρ^{MD}/ [kg/m^3]	ρ^{REFP}/ [kg/m^3]	β_T^{MC}/ [10^{-5}/bar]	β_T^{REFP}/ [10^{-5}/bar]	α_P^{MC}/ [10^{-4}/K]	α_P^{REFP}/ [10^{-4}/K]	μ_{JT}^{MC}/ [10^{-3} K/bar]	μ_{JT}^{REFP}/ [10^{-3} K/bar]
20	50.9 ± 0.1	52.2 ± 0.1	82.6	6394.5 ± 17.6	6954.2	77.0 ± 0.4	176.2	1569.2 ± 17.0	–
40	1032.1 ± 0.7	1030.2 ± 0.7	1035.7	37.4 ± 1.6	42.6	43.7 ± 1.7	45.5	4.7 ± 1.9	6.9
60	1039.8 ± 1.2	1037.2 ± 1.2	1044.2	34.4 ± 0.9	39.1	41.4 ± 1.0	42.9	2.1 ± 1.1	4.0
80	1046.6 ± 1.6	1045.3 ± 0.8	1052.1	31.5 ± 0.9	36.2	38.8 ± 1.0	40.8	-0.9 ± 1.2	1.5
100	1054.6 ± 0.4	1052.1 ± 0.6	1059.5	29.5 ± 1.1	33.8	37.2 ± 1.4	38.9	-2.7 ± 1.7	-0.7
120	1060.3 ± 0.9	1058.1 ± 0.6	1066.4	28.1 ± 1.2	31.6	36.2 ± 1.6	37.3	-3.9 ± 1.9	-2.7
140	1065.2 ± 0.7	1064.4 ± 0.4	1073.0	26.6 ± 1.3	29.8	34.7 ± 1.9	35.9	-5.7 ± 2.3	-4.4
160	1071.2 ± 0.4	1069.4 ± 0.5	1079.3	25.7 ± 0.6	28.2	34.1 ± 0.9	34.6	-6.4 ± 1.1	-5.9
180	1076.1 ± 1.3	1075.4 ± 0.8	1085.2	24.5 ± 1.0	26.8	33.0 ± 1.2	33.5	-7.8 ± 1.4	-7.4
200	1082.0 ± 0.8	1080.7 ± 0.3	1090.9	23.7 ± 0.4	25.5	32.8 ± 0.6	32.5	-7.9 ± 0.7	-8.6

Table S98: Heat capacities at constant volume computed from MC simulations (c_V^{MC}), heat capacities at constant volume obtained from REFPROP[10] (c_V^{REFP}), heat capacities at constant pressure computed from MC simulations (c_P^{MC}), heat capacities at constant pressure obtained from REFPROP[10] (c_P^{REFP}), speed of sound computed from MC simulations (c^{MC}), speed of sound obtained from REFPROP[10] (c^{REFP}), viscosities computed from MD simulations (η^{MD}), and viscosities obtained from REFPROP[10] (η^{REFP}) of CO_2-Ar binary mixture with 1 mole% impurity of Ar at 253 K and pressures ranging from 20 bar to 200 bar.

P/ [bar]	c_V^{MC}/ [J/mol K]	c_V^{REFP}/ [J/mol K]	c_P^{MC}/ [J/mol K]	c_P^{REFP}/ [J/mol K]	c^{MC}/ [m/s]	c^{REFP}/ [m/s]	η^{MD}/ [μPa s]	η^{REFP}/ [μPa s]
20	31.9 ± 0.1	–	52.2 ± 0.1	–	224.1 ± 0.5	–	12.1 ± 2.4	13.3
40	40.4 ± 0.2	40.9	95.7 ± 2.2	93.0	783.2 ± 18.9	717.4	148.9 ± 5.5	140.7
60	40.7 ± 0.1	40.9	93.7 ± 1.2	91.1	802.5 ± 11.8	738.2	150.8 ± 3.9	144.6
80	40.6 ± 0.2	40.9	91.3 ± 1.3	89.4	826.2 ± 13.1	757.5	157.2 ± 3.0	148.4
100	40.6 ± 0.2	40.9	90.0 ± 2.1	88.1	844.2 ± 18.3	775.6	167.0 ± 15.9	152.1
120	40.7 ± 0.2	41.0	89.5 ± 2.2	86.8	858.4 ± 21.7	792.6	161.2 ± 11.2	155.7
140	40.6 ± 0.1	41.0	88.2 ± 2.8	85.8	876.2 ± 25.8	808.8	164.3 ± 4.1	159.1
160	40.9 ± 0.3	41.0	87.8 ± 1.6	84.8	882.9 ± 13.9	824.1	196.9 ± 34.2	162.5
180	40.9 ± 0.1	41.1	86.6 ± 1.5	84.0	896.3 ± 19.7	838.7	175.7 ± 5.8	165.8
200	40.9 ± 0.3	41.1	87.5 ± 1.2	83.2	913.0 ± 10.5	852.7	173.0 ± 14.5	169.1

Table S99: Densities computed from MC and MD simulations (ρ^{MC} and ρ^{MD}), densities obtained from REFPROP[10] (ρ^{REFP}), isothermal compressibilities computed from MC simulations (β_T^{MC}), isothermal compressibilities obtained from REFPROP[10] (β_T^{REFP}), thermal expansion coefficients computed from MC simulations (α_P^{MC}), thermal expansion coefficients obtained from REFPROP[10] (α_P^{REFP}), Joule Thomson coefficients computed from MC simulations ($\mu_{\mathrm{JT}}^{\mathrm{MC}}$), and Joule Thomson coefficients obtained from REFPROP[10] ($\mu_{\mathrm{JT}}^{\mathrm{REFP}}$) of CO_2-Ar binary mixture with 1 mole% impurity of Ar at 273 K and pressures ranging from 20 bar to 200 bar.

P/ [bar]	ρ^{MC}/ [kg/m^3]	ρ^{MD}/ [kg/m^3]	ρ^{REFP}/ [kg/m^3]	β_T^{MC}/ [10^{-5}/bar]	β_T^{REFP}/ [10^{-5}/bar]	α_P^{MC}/ [10^{-4}/K]	α_P^{REFP}/ [10^{-4}/K]	$\mu_{\mathrm{JT}}^{\mathrm{MC}}$/ [$10^{-3}$ K/bar]	$\mu_{\mathrm{JT}}^{\mathrm{REFP}}$/ [$10^{-3}$ K/bar]
20	44.6	45.4 ± 0.1	45.5	5871.3 ± 23.9	6001.6	58.5 ± 0.4	61.8	1261.0 ± 23.8	1381.2
40	927.4 ± 1.5	924.7 ± 1.9	924.2	84.5 ± 8.6	102.6	68.7 ± 5.7	74.6	36.8 ± 6.7	44.2
60	942.4 ± 1.1	940.0 ± 0.9	941.4	64.5 ± 2.4	83.1	56.4 ± 1.9	64.4	24.5 ± 2.4	34.0
80	955.0 ± 1.1	951.2 ± 1.6	955.9	60.4 ± 3.4	70.5	54.7 ± 2.8	57.6	22.2 ± 3.4	26.4
100	966.0 ± 1.4	963.2 ± 1.0	968.6	54.7 ± 2.8	61.5	51.7 ± 2.5	52.5	18.6 ± 3.1	20.5
120	976.0 ± 1.1	973.7 ± 0.9	979.9	48.2 ± 1.5	54.8	46.7 ± 1.3	48.6	13.0 ± 1.7	15.7
140	984.5 ± 1.7	983.5 ± 0.8	990.1	44.8 ± 1.8	49.5	44.7 ± 1.8	45.4	10.4 ± 2.4	11.7
160	993.2 ± 0.9	991.3 ± 1.1	999.5	39.2 ± 1.5	45.2	40.7 ± 1.6	42.8	5.4 ± 2.1	8.3
180	1001.8 ± 0.4	999.4 ± 0.6	1008.3	36.7 ± 0.4	41.7	38.8 ± 0.4	40.6	3.0 ± 0.6	5.4
200	1008.1 ± 0.5	1006.4 ± 0.8	1016.4	35.2 ± 1.2	38.7	37.8 ± 1.3	38.7	1.6 ± 1.7	2.9

Table S100: Heat capacities at constant volume computed from MC simulations (c_V^{MC}), heat capacities at constant volume obtained from REFPROP[10] (c_V^{REFP}), heat capacities at constant pressure computed from MC simulations (c_P^{MC}), heat capacities at constant pressure obtained from REFPROP[10] (c_P^{REFP}), speed of sound computed from MC simulations (c^{MC}), speed of sound obtained from REFPROP[10] (c^{REFP}), viscosities computed from MD simulations (η^{MD}), and viscosities obtained from REFPROP[10] (η^{REFP}) of CO_2-Ar binary mixture with 1 mole% impurity of Ar at 273 K and pressures ranging from 20 bar to 200 bar.

P/ [bar]	c_V^{MC}/ [J/mol K]	c_V^{REFP}/ [J/mol K]	c_P^{MC}/ [J/mol K]	c_P^{REFP}/ [J/mol K]	c^{MC}/ [m/s]	c^{REFP}/ [m/s]	η^{MD}/ [μPa s]	η^{REFP}/ [μPa s]
20	30.9	31.3	46.6 ± 0.2	48.2	239.9 ± 0.7	237.3	4.7 ± 3.1	14.0
40	39.9 ± 0.3	41.0	113.0 ± 5.1	111.4	601.2 ± 33.5	535.5	105.5 ± 2.9	99.1
60	39.9 ± 0.1	40.7	103.0 ± 2.2	104.4	651.5 ± 13.9	572.4	111.4 ± 2.7	104.0
80	39.9 ± 0.1	40.5	102.2 ± 2.9	99.6	666.0 ± 21.0	603.6	118.9 ± 7.8	108.5
100	39.7 ± 0.1	40.5	100.7 ± 3.0	96.0	692.8 ± 20.4	630.9	114.8 ± 3.6	112.6
120	39.8 ± 0.2	40.4	95.5 ± 1.6	93.2	713.4 ± 12.9	655.4	114.6 ± 22.2	116.4
140	39.9 ± 0.1	40.4	94.4 ± 2.4	90.9	732.5 ± 17.6	677.8	129.0 ± 6.8	120.0
160	40.1 ± 0.1	40.4	91.2 ± 2.1	89.0	764.3 ± 17.0	698.5	147.9 ± 29.5	123.5
180	40.2 ± 0.1	40.4	89.2 ± 0.9	87.5	776.3 ± 6.1	717.7	139.7 ± 8.9	126.8
200	40.2 ± 0.1	40.4	88.4 ± 1.7	86.1	786.9 ± 15.3	735.8	139.8 ± 6.7	130.0

Table S101: Densities computed from MC and MD simulations (ρ^{MC} and ρ^{MD}), densities obtained from REFPROP[10] (ρ^{REFP}), isothermal compressibilities computed from MC simulations (β_T^{MC}), isothermal compressibilities obtained from REFPROP[10] (β_T^{REFP}), thermal expansion coefficients computed from MC simulations (α_P^{MC}), thermal expansion coefficients obtained from REFPROP[10] (α_P^{REFP}), Joule Thomson coefficients computed from MC simulations ($\mu_{\mathrm{JT}}^{\mathrm{MC}}$), and Joule Thomson coefficients obtained from REFPROP[10] ($\mu_{\mathrm{JT}}^{\mathrm{REFP}}$) of CO_2-Ar binary mixture with 1 mole% impurity of Ar at 293 K and pressures ranging from 20 bar to 200 bar.

P/ [bar]	ρ^{MC}/ [kg/m^3]	ρ^{MD}/ [kg/m^3]	ρ^{REFP}/ [kg/m^3]	β_T^{MC}/ [10^{-5}/bar]	β_T^{REFP}/ [10^{-5}/bar]	α_P^{MC}/ [10^{-4}/K]	α_P^{REFP}/ [10^{-4}/K]	$\mu_{\mathrm{JT}}^{\mathrm{MC}}$/ [$10^{-3}$ K/bar]	$\mu_{\mathrm{JT}}^{\mathrm{REFP}}$/ [$10^{-3}$ K/bar]
20	40.1	40.9 ± 0.1	40.7	5652.0 ± 56.5	5695.9	48.8 ± 0.5	50.4	1061.9 ± 34.2	1140.9
40	93.4 ± 0.1	96.2 ± 0.5	96.8	3455.7 ± 34.6	3616.0	80.9 ± 0.5	89.6	1071.6 ± 11.3	1142.2
60	787.9 ± 4.8	775.1 ± 4.0	562.7	320.4 ± 43.3	−3895.4	158.5 ± 14.6	−546.2	118.7 ± 15.3	–
80	826.3 ± 3.8	815.2 ± 2.0	815.8	176.3 ± 23.7	232.4	102.4 ± 11.7	117.3	79.6 ± 14.8	97.1
100	851.2 ± 1.7	847.1 ± 1.7	847.2	124.8 ± 6.7	156.0	80.0 ± 3.6	88.5	58.9 ± 4.8	70.5
120	870.4 ± 2.6	868.0 ± 1.2	870.5	105.4 ± 6.3	119.4	72.1 ± 3.6	73.6	49.7 ± 4.9	54.3
140	886.9 ± 0.6	885.9 ± 1.4	889.4	82.6 ± 3.6	97.4	60.3 ± 1.8	64.2	36.7 ± 2.6	42.9
160	901.1 ± 1.2	898.5 ± 0.9	905.5	75.2 ± 2.4	82.7	57.3 ± 1.8	57.5	32.4 ± 2.5	34.3
180	912.6 ± 1.5	911.7 ± 1.7	919.6	66.3 ± 3.4	72.0	52.5 ± 2.5	52.6	26.3 ± 3.7	27.5
200	923.7 ± 0.8	922.6 ± 0.7	932.1	59.4 ± 2.4	63.9	48.6 ± 1.8	48.6	21.2 ± 2.7	22.0

Table S102: Heat capacities at constant volume computed from MC simulations (c_V^{MC}), heat capacities at constant volume obtained from REFPROP[10] (c_V^{REFP}), heat capacities at constant pressure computed from MC simulations (c_P^{MC}), heat capacities at constant pressure obtained from REFPROP[10] (c_P^{REFP}), speed of sound computed from MC simulations (c^{MC}), speed of sound obtained from REFPROP[10] (c^{REFP}), viscosities computed from MD simulations (η^{MD}), and viscosities obtained from REFPROP[10] (η^{REFP}) of CO_2-Ar binary mixture with 1 mole% impurity of Ar at 293 K and pressures ranging from 20 bar to 200 bar.

P/ [bar]	c_V^{MC}/ [J/mol K]	c_V^{REFP}/ [J/mol K]	c_P^{MC}/ [J/mol K]	c_P^{REFP}/ [J/mol K]	c^{MC}/ [m/s]	c^{REFP}/ [m/s]	η^{MD}/ [μPa s]	η^{REFP}/ [μPa s]
20	30.9	31.0	44.4 ± 0.1	45.1	252.0 ± 1.3	250.6	9.0 ± 2.8	15.0
40	34.0	35.1	60.2 ± 0.1	64.6	234.1 ± 1.2	229.4	15.9 ± 2.0	15.7
60	40.2 ± 0.5	–	171.3 ± 9.0	–	410.7 ± 29.9	–	68.9 ± 1.3	40.2
80	39.7 ± 0.1	41.7	133.7 ± 9.6	135.3	480.5 ± 36.6	413.5	73.1 ± 2.9	74.1
100	39.6 ± 0.2	40.9	117.7 ± 2.8	117.3	529.0 ± 15.7	465.6	86.9 ± 4.3	80.3
120	39.5 ± 0.2	40.5	113.0 ± 3.3	107.8	558.5 ± 18.6	505.9	89.9 ± 2.2	85.4
140	39.4 ± 0.1	40.3	103.7 ± 1.2	101.6	599.3 ± 13.5	539.4	95.5 ± 7.2	89.8
160	39.5 ± 0.1	40.2	102.1 ± 2.0	97.2	617.2 ± 11.6	568.6	96.4 ± 5.2	93.7
180	39.4	40.1	98.4 ± 2.9	93.8	642.7 ± 19.0	594.6	102.6 ± 4.0	97.4
200	39.5 ± 0.2	40.0	95.1 ± 2.1	91.2	662.4 ± 15.3	618.3	119.3 ± 18.4	100.8

Table S103: Densities computed from MC and MD simulations (ρ^{MC} and ρ^{MD}), densities obtained from REFPROP[10] (ρ^{REFP}), isothermal compressibilities computed from MC simulations (β_T^{MC}), isothermal compressibilities obtained from REFPROP[10] (β_T^{REFP}), thermal expansion coefficients computed from MC simulations (α_P^{MC}), thermal expansion coefficients obtained from REFPROP[10] (α_P^{REFP}), Joule Thomson coefficients computed from MC simulations (μ_{JT}^{MC}), and Joule Thomson coefficients obtained from REFPROP[10] (μ_{JT}^{REFP}) of CO_2-Ar binary mixture with 1 mole% impurity of Ar at 313 K and pressures ranging from 20 bar to 200 bar.

P/ [bar]	ρ^{MC}/ [kg/m^3]	ρ^{MD}/ [kg/m^3]	ρ^{REFP}/ [kg/m^3]	β_T^{MC}/ [10^{-5}/bar]	β_T^{REFP}/ [10^{-5}/bar]	α_P^{MC}/ [10^{-4}/K]	α_P^{REFP}/ [10^{-4}/K]	μ_{JT}^{MC}/ [10^{-3} K/bar]	μ_{JT}^{REFP}/ [10^{-3} K/bar]
20	36.6	37.3 ± 0.1	37.1	5468.7 ± 20.7	5514.3	42.2 ± 0.2	43.3	889.7 ± 18.0	960.3
40	81.4	83.4 ± 0.1	83.4	3091.3 ± 19.7	3181.4	59.4 ± 0.4	63.4	886.5 ± 14.2	953.4
60	141.5 ± 0.2	146.6 ± 1.0	147.7	2551.1 ± 37.5	2702.4	97.6 ± 2.1	109.3	883.9 ± 31.0	921.4
80	245.3 ± 1.7	269.4 ± 4.3	267.7	3253.2 ± 145.2	3654.7	259.9 ± 12.8	319.9	807.7 ± 56.8	–
100	611.7 ± 9.2	598.7 ± 4.8	599.6	1407.7 ± 257.4	1555.8	385.7 ± 50.7	397.0	276.6 ± 46.8	–
120	699.4 ± 4.1	703.7 ± 4.2	703.5	428.0 ± 33.2	452.3	164.6 ± 9.8	161.9	156.5 ± 12.9	163.3
140	752.5 ± 1.3	750.0 ± 1.0	752.8	225.4 ± 17.3	260.7	104.5 ± 7.8	110.4	102.6 ± 12.2	113.3
160	782.9 ± 3.4	780.8 ± 1.9	786.3	172.4 ± 10.8	184.1	87.9 ± 4.8	87.3	82.7 ± 7.6	85.7
180	809.1 ± 1.6	805.0 ± 1.3	812.1	132.0 ± 10.8	142.6	73.6 ± 5.6	73.8	64.7 ± 9.2	67.6
200	826.5 ± 1.7	825.3 ± 0.5	833.3	108.7 ± 5.4	116.6	65.0 ± 2.7	64.7	52.7 ± 4.5	54.5

Table S104: Heat capacities at constant volume computed from MC simulations (c_V^{MC}), heat capacities at constant volume obtained from REFPROP[10] (c_V^{REFP}), heat capacities at constant pressure computed from MC simulations (c_P^{MC}), heat capacities at constant pressure obtained from REFPROP[10] (c_P^{REFP}), speed of sound computed from MC simulations (c^{MC}), speed of sound obtained from REFPROP[10] (c^{REFP}), viscosities computed from MD simulations (η^{MD}), and viscosities obtained from REFPROP[10] (η^{REFP}) of CO_2-Ar binary mixture with 1 mole% impurity of Ar at 313 K and pressures ranging from 20 bar to 200 bar.

P/ [bar]	c_V^{MC}/ [J/mol K]	c_V^{REFP}/ [J/mol K]	c_P^{MC}/ [J/mol K]	c_P^{REFP}/ [J/mol K]	c^{MC}/ [m/s]	c^{REFP}/ [m/s]	η^{MD}/ [μPa s]	η^{REFP}/ [μPa s]
20	31.2	31.2	43.4 ± 0.1	43.8	263.6 ± 0.6	262.1	13.6 ± 5.0	16.0
40	33.1	33.6	52.4 ± 0.2	54.4	251.0 ± 0.9	247.2	16.9 ± 1.8	16.6
60	35.8 ± 0.1	37.0	72.3 ± 1.1	78.2	236.8 ± 2.5	230.0	19.2 ± 2.3	17.8
80	40.5 ± 0.2	–	158.3 ± 6.6	–	221.5 ± 6.8	–	20.2 ± 0.8	21.6
100	41.9 ± 0.4	–	288.0 ± 25.5	–	282.6 ± 28.8	–	42.0 ± 1.2	44.5
120	40.4 ± 0.2	42.3	166.7 ± 6.0	155.6	371.4 ± 16.0	340.2	58.0 ± 2.4	56.8
140	39.6 ± 0.2	41.1	129.3 ± 6.6	126.6	438.9 ± 20.3	396.2	63.5 ± 2.4	63.9
160	39.3 ± 0.1	40.5	118.8 ± 4.0	113.0	473.4 ± 16.8	439.0	72.4 ± 4.6	69.3
180	39.4 ± 0.2	40.2	109.6 ± 5.1	104.9	510.2 ± 24.0	474.5	72.8 ± 1.7	73.9
200	39.1 ± 0.1	40.0	104.3 ± 2.4	99.3	544.9 ± 14.9	505.3	87.4 ± 13.2	77.9

S15.5 Thermodynamic and Transport Properties Data of binary CO_2 mixture with 5 mole% Impurity of Ar

Table S105: Densities computed from MC and MD simulations (ρ^{MC} and ρ^{MD}), densities obtained from REFPROP[10] (ρ^{REFP}), isothermal compressibilities computed from MC simulations (β_T^{MC}), isothermal compressibilities obtained from REFPROP[10] (β_T^{REFP}), thermal expansion coefficients computed from MC simulations (α_P^{MC}), thermal expansion coefficients obtained from REFPROP[10] (α_P^{REFP}), Joule Thomson coefficients computed from MC simulations ($\mu_{\mathrm{JT}}^{\mathrm{MC}}$), and Joule Thomson coefficients obtained from REFPROP[10] ($\mu_{\mathrm{JT}}^{\mathrm{REFP}}$) of CO_2-Ar binary mixture with 5 mole% impurity of Ar at 253 K and pressures ranging from 20 bar to 200 bar.

P/ [bar]	ρ^{MC}/ [kg/m^3]	ρ^{MD}/ [kg/m^3]	ρ^{REFP}/ [kg/m^3]	β_T^{MC}/ [10^{-5}/bar]	β_T^{REFP}/ [10^{-5}/bar]	α_P^{MC}/ [10^{-4}/K]	α_P^{REFP}/ [10^{-4}/K]	$\mu_{\mathrm{JT}}^{\mathrm{MC}}$/ [$10^{-3}$ K/bar]	$\mu_{\mathrm{JT}}^{\mathrm{REFP}}$/ [$10^{-3}$ K/bar]
20	50.0	52.2 ± 0.1	51.3	6297.1 ± 32.0	6437.6	73.9 ± 0.3	79.1	1521.6 ± 12.7	1620.4
40	1011.6 ± 1.0	1030.2 ± 0.7	858.5	46.2 ± 2.6	-1829.0	49.5 ± 2.7	-838.4	11.2 ± 3.1	–
60	1020.0 ± 0.8	1037.2 ± 1.2	1021.2	41.8 ± 1.8	47.3	46.2 ± 2.2	47.6	7.6 ± 2.6	9.6
80	1027.4 ± 1.6	1045.3 ± 0.8	1030.5	40.3 ± 2.4	43.1	45.1 ± 2.7	44.7	6.4 ± 3.1	6.2
100	1035.5 ± 1.5	1034.6 ± 0.4	1039.0	35.5 ± 2.2	39.7	40.7 ± 2.6	42.3	1.4 ± 3.1	3.3
120	1043.1 ± 0.4	1041.6 ± 0.8	1047.0	33.6 ± 1.6	36.8	39.8 ± 2.0	40.2	0.3 ± 2.3	0.9
140	1050.0 ± 0.7	1049.0 ± 0.6	1054.5	30.4 ± 0.9	34.3	37.1 ± 1.2	38.5	-2.9 ± 1.5	-1.3
160	1056.2 ± 0.7	1054.1 ± 1.0	1061.5	29.6 ± 0.8	32.2	36.5 ± 1.1	36.9	-3.6 ± 1.3	-3.3
180	1063.4 ± 0.7	1061.0 ± 0.5	1068.2	27.7 ± 1.2	30.4	34.8 ± 1.4	35.5	-5.8 ± 1.7	-5.0
200	1067.5 ± 0.8	1066.8 ± 0.8	1074.5	27.3 ± 1.4	28.8	35.0 ± 2.0	34.3	-5.5 ± 2.4	-6.6

Table S106: Heat capacities at constant volume computed from MC simulations (c_V^{MC}), heat capacities at constant volume obtained from REFPROP[10] (c_V^{REFP}), heat capacities at constant pressure computed from MC simulations (c_P^{MC}), heat capacities at constant pressure obtained from REFPROP[10] (c_P^{REFP}), speed of sound computed from MC simulations (c^{MC}), speed of sound obtained from REFPROP[10] (c^{REFP}), viscosities computed from MD simulations (η^{MD}), and viscosities obtained from REFPROP[10] (η^{REFP}) of CO_2-Ar binary mixture with 5 mole% impurity of Ar at 253 K and pressures ranging from 20 bar to 200 bar.

P/ [bar]	c_V^{MC}/ [J/mol K]	c_V^{REFP}/ [J/mol K]	c_P^{MC}/ [J/mol K]	c_P^{REFP}/ [J/mol K]	c^{MC}/ [m/s]	c^{REFP}/ [m/s]	η^{MD}/ [μPa s]	η^{REFP}/ [μPa s]
20	30.9 ± 0.1	31.8	50.1 ± 0.1	52.8	226.9 ± 0.6	224.4	12.1 ± 2.4	13.3
40	39.5 ± 0.2	–	97.6 ± 3.3	–	727.2 ± 24.0	–	148.9 ± 5.5	83.9
60	39.4 ± 0.1	39.6	94.9 ± 3.1	91.5	752.0 ± 20.5	691.3	150.8 ± 3.9	131.1
80	39.6 ± 0.2	39.6	94.1 ± 3.6	89.4	757.9 ± 26.9	713.0	157.2 ± 3.0	135.0
100	39.7 ± 0.2	39.6	89.7 ± 3.6	87.6	784.0 ± 28.8	733.1	159.6 ± 29.5	138.7
120	39.4 ± 0.2	39.6	89.5 ± 2.5	86.2	805.5 ± 22.1	751.9	147.3 ± 4.2	142.3
140	39.6 ± 0.3	39.6	87.4 ± 1.6	84.9	830.9 ± 14.8	769.5	159.5 ± 19.7	145.8
160	39.7 ± 0.1	39.6	87.3 ± 1.5	83.8	839.0 ± 13.9	786.1	159.6 ± 7.7	149.2
180	39.6 ± 0.1	39.7	85.3 ± 1.9	82.8	855.4 ± 21.3	801.9	160.6 ± 6.3	152.5
200	39.8 ± 0.2	39.7	86.2 ± 2.8	81.9	862.5 ± 26.4	816.9	165.9 ± 8.6	155.7

Table S107: Densities computed from MC and MD simulations (ρ^{MC} and ρ^{MD}), densities obtained from REFPROP[10] (ρ^{REFP}), isothermal compressibilities computed from MC simulations (β_T^{MC}), isothermal compressibilities obtained from REFPROP[10] (β_T^{REFP}), thermal expansion coefficients computed from MC simulations (α_P^{MC}), thermal expansion coefficients obtained from REFPROP[10] (α_P^{REFP}), Joule Thomson coefficients computed from MC simulations ($\mu_{\mathrm{JT}}^{\mathrm{MC}}$), and Joule Thomson coefficients obtained from REFPROP[10] ($\mu_{\mathrm{JT}}^{\mathrm{REFP}}$) of CO_2-Ar binary mixture with 5 mole% impurity of Ar at 273 K and pressures ranging from 20 bar to 200 bar.

P/ [bar]	ρ^{MC}/ [kg/m^3]	ρ^{MD}/ [kg/m^3]	ρ^{REFP}/ [kg/m^3]	β_T^{MC}/ [10^{-5}/bar]	β_T^{REFP}/ [10^{-5}/bar]	α_P^{MC}/ [10^{-4}/K]	α_P^{REFP}/ [10^{-4}/K]	$\mu_{\mathrm{JT}}^{\mathrm{MC}}$/ [$10^{-3}$ K/bar]	$\mu_{\mathrm{JT}}^{\mathrm{REFP}}$/ [$10^{-3}$ K/bar]
20	44.0	45.4 ± 0.1	44.8	5816.5 ± 52.4	5906.1	56.9 ± 0.5	59.3	1219.3 ± 32.1	1310.9
40	110.1 ± 0.3	121.3 ± 0.5	209.1	4244.7 ± 157.0	−40 837.1	129.0 ± 6.0	−3596.4	1277.8 ± 92.0	–
60	911.4 ± 1.1	940.0 ± 0.9	905.9	90.8 ± 3.0	115.9	69.1 ± 2.2	78.7	39.3 ± 2.8	50.4
80	925.7 ± 1.9	951.2 ± 1.6	924.8	77.6 ± 4.3	92.5	62.3 ± 3.1	67.3	31.9 ± 4.0	38.6
100	939.1 ± 2.1	937.4 ± 1.0	940.6	72.0 ± 2.0	77.6	59.7 ± 1.3	59.7	28.6 ± 1.7	30.1
120	951.1 ± 1.5	950.3 ± 0.7	954.2	60.2 ± 2.8	67.2	52.9 ± 2.4	54.2	20.9 ± 3.1	23.5
140	962.7 ± 1.6	961.7 ± 1.1	966.4	53.9 ± 3.0	59.5	49.3 ± 2.6	50.0	16.6 ± 3.5	18.2
160	973.3 ± 1.7	971.4 ± 0.8	977.3	49.5 ± 2.2	53.5	46.7 ± 1.9	46.6	13.3 ± 2.5	13.8
180	982.8 ± 1.5	980.9 ± 1.0	987.4	46.4 ± 1.4	48.7	45.3 ± 1.2	43.8	11.3 ± 1.6	10.0
200	991.0 ± 0.5	989.0 ± 0.8	996.6	39.9 ± 1.5	44.7	39.7 ± 1.7	41.5	4.3 ± 2.3	6.8

Table S108: Heat capacities at constant volume computed from MC simulations (c_V^{MC}), heat capacities at constant volume obtained from REFPROP[10] (c_V^{REFP}), heat capacities at constant pressure computed from MC simulations (c_P^{MC}), heat capacities at constant pressure obtained from REFPROP[10] (c_P^{REFP}), speed of sound computed from MC simulations (c^{MC}), speed of sound obtained from REFPROP[10] (c^{REFP}), viscosities computed from MD simulations (η^{MD}), and viscosities obtained from REFPROP[10] (η^{REFP}) of CO_2-Ar binary mixture with 5 mole% impurity of Ar at 273 K and pressures ranging from 20 bar to 200 bar.

P/ [bar]	c_V^{MC}/ [J/mol K]	c_V^{REFP}/ [J/mol K]	c_P^{MC}/ [J/mol K]	c_P^{REFP}/ [J/mol K]	c^{MC}/ [m/s]	c^{REFP}/ [m/s]	η^{MD}/ [μPa s]	η^{REFP}/ [μPa s]
20	30.1	30.3	45.2 ± 0.1	46.2	242.4 ± 1.2	240.2	4.7 ± 3.1	14.3
40	35.9 ± 0.2	–	78.5 ± 2.4	–	216.1 ± 5.2	–	14.5 ± 0.7	17.5
60	39.1 ± 0.3	39.8	108.6 ± 2.5	110.4	579.5 ± 11.7	514.2	111.4 ± 2.7	92.6
80	38.7 ± 0.2	39.5	104.0 ± 2.9	102.8	611.6 ± 19.2	552.0	118.9 ± 7.8	97.5
100	38.9 ± 0.1	39.3	102.7 ± 1.4	97.7	624.5 ± 9.8	583.9	112.0 ± 5.6	101.9
120	38.9 ± 0.1	39.2	97.5 ± 2.5	94.0	662.2 ± 17.6	611.7	110.1 ± 4.2	106.0
140	38.9 ± 0.1	39.1	95.1 ± 3.2	91.1	686.4 ± 22.4	636.6	119.4 ± 4.9	109.7
160	38.8 ± 0.2	39.1	93.4 ± 2.0	88.8	706.4 ± 17.8	659.3	122.3 ± 7.4	113.3
180	39.0 ± 0.1	39.1	92.8 ± 1.5	86.9	722.2 ± 12.5	680.3	122.9 ± 9.2	116.6
200	39.1 ± 0.3	39.1	86.8 ± 2.1	85.3	749.1 ± 17.4	699.8	128.5 ± 11.7	119.9

Table S109: Densities computed from MC and MD simulations (ρ^{MC} and ρ^{MD}), densities obtained from REFPROP[10] (ρ^{REFP}), isothermal compressibilities computed from MC simulations (β_T^{MC}), isothermal compressibilities obtained from REFPROP[10] (β_T^{REFP}), thermal expansion coefficients computed from MC simulations (α_P^{MC}), thermal expansion coefficients obtained from REFPROP[10] (α_P^{REFP}), Joule Thomson coefficients computed from MC simulations (μ_{JT}^{MC}), and Joule Thomson coefficients obtained from REFPROP[10] (μ_{JT}^{REFP}) of CO_2-Ar binary mixture with 5 mole% impurity of Ar at 293 K and pressures ranging from 20 bar to 200 bar.

P/ [bar]	ρ^{MC}/ [kg/m^3]	ρ^{MD}/ [kg/m^3]	ρ^{REFP}/ [kg/m^3]	β_T^{MC}/ [10^{-5}/bar]	β_T^{REFP}/ [10^{-5}/bar]	α_P^{MC}/ [10^{-4}/K]	α_P^{REFP}/ [10^{-4}/K]	μ_{JT}^{MC}/ [10^{-3} K/bar]	μ_{JT}^{REFP}/ [10^{-3} K/bar]
20	39.7	40.9 ± 0.1	40.2	5578.5 ± 42.1	5639.2	47.6 ± 0.4	49.0	1008.1 ± 27.4	1088.0
40	91.1 ± 0.1	96.2 ± 0.5	93.9	3336.6 ± 30.9	3457.1	75.5 ± 0.7	81.3	1023.0 ± 19.2	1082.4
60	174.7 ± 0.6	666.0 ± 152.2	186.6	3600.0 ± 49.3	3968.1	187.3 ± 3.7	227.4	1018.9 ± 31.2	1017.1
80	762.0 ± 3.2	815.2 ± 2.0	749.1	346.8 ± 70.6	482.7	156.8 ± 25.0	192.0	128.4 ± 29.1	156.4
100	807.6 ± 0.9	805.0 ± 2.7	800.3	202.8 ± 18.2	239.2	109.6 ± 7.8	115.1	89.5 ± 9.8	100.6
120	835.0 ± 2.1	831.3 ± 1.4	832.3	143.8 ± 12.3	163.8	86.0 ± 6.7	88.5	67.3 ± 9.3	74.0
140	857.6 ± 2.6	852.4 ± 1.3	856.5	111.4 ± 11.1	126.0	72.1 ± 6.0	74.0	52.2 ± 8.7	57.3
160	872.3 ± 1.8	872.3 ± 0.9	876.2	92.9 ± 5.2	103.0	62.8 ± 3.2	64.7	41.6 ± 4.9	45.4
180	889.1 ± 1.8	886.6 ± 0.6	892.9	78.6 ± 2.7	87.4	56.5 ± 1.6	58.0	33.2 ± 2.5	36.4
200	902.4 ± 0.6	899.5 ± 0.8	907.6	71.2 ± 2.5	76.0	53.6 ± 1.8	53.0	28.8 ± 2.7	29.3

Table S110: Heat capacities at constant volume computed from MC simulations (c_V^{MC}), heat capacities at constant volume obtained from REFPROP[10] (c_V^{REFP}), heat capacities at constant pressure computed from MC simulations (c_P^{MC}), heat capacities at constant pressure obtained from REFPROP[10] (c_P^{REFP}), speed of sound computed from MC simulations (c^{MC}), speed of sound obtained from REFPROP[10] (c^{REFP}), viscosities computed from MD simulations (η^{MD}), and viscosities obtained from REFPROP[10] (η^{REFP}) of CO_2-Ar binary mixture with 5 mole% impurity of Ar at 293 K and pressures ranging from 20 bar to 200 bar.

P/ [bar]	c_V^{MC}/ [J/mol K]	c_V^{REFP}/ [J/mol K]	c_P^{MC}/ [J/mol K]	c_P^{REFP}/ [J/mol K]	c^{MC}/ [m/s]	c^{REFP}/ [m/s]	η^{MD}/ [μPa s]	η^{REFP}/ [μPa s]
20	30.1	30.1	43.2 ± 0.1	43.7	254.7 ± 1.0	253.1	9.0 ± 2.8	15.3
40	32.9 ± 0.1	33.4	56.9 ± 0.3	59.6	238.6 ± 1.3	234.3	15.9 ± 2.0	16.0
60	38.1 ± 0.2	41.1	110.4 ± 2.1	130.7	214.7 ± 2.6	207.3	56.5 ± 21.0	18.0
80	39.7 ± 0.2	42.1	161.0 ± 15.9	173.0	392.0 ± 44.3	337.2	73.1 ± 2.9	62.1
100	39.1 ± 0.3	40.3	134.1 ± 5.0	129.2	457.8 ± 22.3	409.3	73.2 ± 5.5	70.4
120	38.6 ± 0.2	39.6	118.4 ± 6.0	113.2	505.5 ± 25.2	458.1	77.5 ± 7.3	76.4
140	38.7 ± 0.2	39.2	108.7 ± 5.2	104.4	541.8 ± 30.1	496.6	83.0 ± 6.5	81.2
160	38.6 ± 0.1	39.0	101.5 ± 3.2	98.5	569.6 ± 18.4	529.1	90.8 ± 6.2	85.5
180	38.4 ± 0.1	38.9	97.2 ± 1.9	94.3	601.5 ± 11.8	557.6	90.8 ± 5.8	89.3
200	38.4 ± 0.1	38.8	96.2 ± 1.9	91.1	623.8 ± 12.7	583.2	94.8 ± 3.1	92.8

Table S111: Densities computed from MC and MD simulations (ρ^{MC} and ρ^{MD}), densities obtained from REFPROP[10] (ρ^{REFP}), isothermal compressibilities computed from MC simulations (β_T^{MC}), isothermal compressibilities obtained from REFPROP[10] (β_T^{REFP}), thermal expansion coefficients computed from MC simulations (α_P^{MC}), thermal expansion coefficients obtained from REFPROP[10] (α_P^{REFP}), Joule Thomson coefficients computed from MC simulations ($\mu_{\mathrm{JT}}^{\mathrm{MC}}$), and Joule Thomson coefficients obtained from REFPROP[10] ($\mu_{\mathrm{JT}}^{\mathrm{REFP}}$) of CO_2-Ar binary mixture with 5 mole% impurity of Ar at 313 K and pressures ranging from 20 bar to 200 bar.

P/ [bar]	ρ^{MC}/ [kg/m^3]	ρ^{MD}/ [kg/m^3]	ρ^{REFP}/ [kg/m^3]	β_T^{MC}/ [10^{-5}/bar]	β_T^{REFP}/ [10^{-5}/bar]	α_P^{MC}/ [10^{-4}/K]	α_P^{REFP}/ [10^{-4}/K]	$\mu_{\mathrm{JT}}^{\mathrm{MC}}$/ [$10^{-3}$ K/bar]	$\mu_{\mathrm{JT}}^{\mathrm{REFP}}$/ [$10^{-3}$ K/bar]
20	36.3	37.3 ± 0.1	36.7	5420.4 ± 23.7	5476.0	41.4 ± 0.2	42.4	844.3 ± 16.3	918.9
40	80.0 ± 0.1	83.4 ± 0.1	81.7	3034.9 ± 31.0	3109.6	57.0 ± 0.7	60.0	850.9 ± 24.3	908.4
60	136.3 ± 0.2	146.6 ± 1.0	141.5	2444.6 ± 23.0	2523.1	88.2 ± 0.8	95.3	850.4 ± 13.6	876.4
80	222.0 ± 0.8	279.0 ± 7.9	235.4	2542.3 ± 39.0	2708.2	174.4 ± 2.9	196.6	781.2 ± 19.6	782.1
100	424.3 ± 12.0	434.1 ± 5.2	444.0	3698.5 ± 267.5	3195.6	602.5 ± 48.8	520.9	504.0 ± 58.3	491.6
120	630.5 ± 5.3	622.1 ± 4.3	627.4	794.4 ± 116.6	824.5	237.5 ± 28.7	233.0	226.9 ± 36.8	236.5
140	697.2 ± 2.7	695.6 ± 3.4	701.0	365.0 ± 42.3	384.3	138.5 ± 13.0	137.7	145.9 ± 19.7	151.2
160	740.0 ± 3.0	740.2 ± 1.8	745.1	240.1 ± 14.8	246.2	105.6 ± 5.4	102.1	108.7 ± 8.7	110.0
180	771.8 ± 1.9	770.3 ± 1.3	777.1	176.2 ± 9.4	180.9	86.7 ± 3.6	83.3	84.8 ± 5.9	84.9
200	796.9 ± 2.6	794.8 ± 1.5	802.4	133.7 ± 10.1	143.0	71.5 ± 4.4	71.5	65.1 ± 7.6	67.6

Table S112: Heat capacities at constant volume computed from MC simulations (c_V^{MC}), heat capacities at constant volume obtained from REFPROP[10] (c_V^{REFP}), heat capacities at constant pressure computed from MC simulations (c_P^{MC}), heat capacities at constant pressure obtained from REFPROP[10] (c_P^{REFP}), speed of sound computed from MC simulations (c^{MC}), speed of sound obtained from REFPROP[10] (c^{REFP}), viscosities computed from MD simulations (η^{MD}), and viscosities obtained from REFPROP[10] (η^{REFP}) of CO_2-Ar binary mixture with 5 mole% impurity of Ar at 313 K and pressures ranging from 20 bar to 200 bar.

P/ [bar]	c_V^{MC}/ [J/mol K]	c_V^{REFP}/ [J/mol K]	c_P^{MC}/ [J/mol K]	c_P^{REFP}/ [J/mol K]	c^{MC}/ [m/s]	c^{REFP}/ [m/s]	η^{MD}/ [μPa s]	η^{REFP}/ [μPa s]
20	30.4	30.3	42.3 ± 0.1	42.6	266.1 ± 0.6	264.4	13.6 ± 5.0	16.3
40	32.1	32.4	50.5 ± 0.3	51.8	254.3 ± 1.5	250.9	16.9 ± 1.8	16.9
60	34.5 ± 0.1	35.2	66.5 ± 0.4	70.1	240.6 ± 1.3	236.2	19.2 ± 2.3	18.0
80	38.0 ± 0.1	39.5	112.7 ± 1.6	122.6	229.1 ± 2.5	220.7	20.7 ± 0.5	20.7
100	42.4 ± 0.8	44.9	366.2 ± 26.6	307.1	234.7 ± 12.7	219.5	30.9 ± 2.4	31.2
120	40.1 ± 0.5	41.9	197.0 ± 16.2	185.8	313.1 ± 26.5	292.8	53.1 ± 7.9	47.3
140	38.9 ± 0.1	40.2	143.6 ± 8.2	136.8	381.1 ± 24.6	355.2	57.6 ± 6.2	56.1
160	38.6 ± 0.1	39.5	125.5 ± 4.1	117.4	427.8 ± 14.9	402.5	62.0 ± 2.6	62.2
180	38.5 ± 0.1	39.1	114.7 ± 2.6	106.8	468.1 ± 13.6	440.9	67.3 ± 5.4	67.1
200	38.2 ± 0.1	38.8	104.5 ± 3.4	100.0	506.3 ± 20.9	473.7	72.2 ± 9.3	71.4

S15.6 Thermodynamic and Transport Properties Data of binary CO_2 mixture with 10 mole% Impurity of Ar

Table S113: Densities computed from MC and MD simulations (ρ^{MC} and ρ^{MD}), densities obtained from REFPROP[10] (ρ^{REFP}), isothermal compressibilities computed from MC simulations (β_T^{MC}), isothermal compressibilities obtained from REFPROP[10] (β_T^{REFP}), thermal expansion coefficients computed from MC simulations (α_P^{MC}), thermal expansion coefficients obtained from REFPROP[10] (α_P^{REFP}), Joule Thomson coefficients computed from MC simulations ($\mu_{\mathrm{JT}}^{\mathrm{MC}}$), and Joule Thomson coefficients obtained from REFPROP[10] ($\mu_{\mathrm{JT}}^{\mathrm{REFP}}$) of CO_2-Ar binary mixture with 10 mole% impurity of Ar at 253 K and pressures ranging from 20 bar to 200 bar.

P/ [bar]	ρ^{MC}/ [kg/m^3]	ρ^{MD}/ [kg/m^3]	ρ^{REFP}/ [kg/m^3]	β_T^{MC}/ [10^{-5}/bar]	β_T^{REFP}/ [10^{-5}/bar]	α_P^{MC}/ [10^{-4}/K]	α_P^{REFP}/ [10^{-4}/K]	$\mu_{\mathrm{JT}}^{\mathrm{MC}}$/ [$10^{-3}$ K/bar]	$\mu_{\mathrm{JT}}^{\mathrm{REFP}}$/ [$10^{-3}$ K/bar]
20	44.9	52.2 ± 0.1	49.9	6085.4 ± 52.4	6225.3	68.8 ± 0.6	72.7	1458.5 ± 31.8	1506.8
40	898.0 ± 0.9	1030.2 ± 0.7	435.8	62.0 ± 3.7	180.9	57.7 ± 2.6	205.2	21.0 ± 3.1	–
60	908.5 ± 1.7	1037.2 ± 1.2	874.2	56.0 ± 1.9	426.0	53.8 ± 1.2	233.8	16.9 ± 1.5	–
80	917.9 ± 1.1	1045.3 ± 0.8	1000.2	51.6 ± 1.8	55.2	51.4 ± 1.8	51.1	14.0 ± 2.1	14.2
100	926.8 ± 0.6	1008.4 ± 1.6	1010.8	47.3 ± 2.7	49.6	48.4 ± 2.9	47.6	10.7 ± 3.5	10.0
120	933.4 ± 1.3	1016.9 ± 0.8	1020.4	42.6 ± 0.9	45.2	45.1 ± 0.9	44.7	6.8 ± 1.1	6.5
140	942.0 ± 0.4	1025.6 ± 0.8	1029.3	37.6 ± 1.8	41.6	40.5 ± 2.1	42.3	1.3 ± 2.7	3.6
160	949.6 ± 0.8	1033.0 ± 0.6	1037.5	36.9 ± 1.3	38.5	41.2 ± 1.3	40.3	2.1 ± 1.6	1.0
180	956.6 ± 1.0	1040.3 ± 0.8	1045.3	32.9 ± 1.0	36.0	37.8 ± 1.3	38.5	-2.2 ± 1.6	-1.3
200	961.7 ± 0.6	1047.7 ± 0.8	1052.6	32.0 ± 1.4	33.7	37.8 ± 1.7	37.0	-2.1 ± 2.1	-3.4

Table S114: Heat capacities at constant volume computed from MC simulations (c_V^{MC}), heat capacities at constant volume obtained from REFPROP[10] (c_V^{REFP}), heat capacities at constant pressure computed from MC simulations (c_P^{MC}), heat capacities at constant pressure obtained from REFPROP[10] (c_P^{REFP}), speed of sound computed from MC simulations (c^{MC}), speed of sound obtained from REFPROP[10] (c^{REFP}), viscosities computed from MD simulations (η^{MD}), and viscosities obtained from REFPROP[10] (η^{REFP}) of CO_2-Ar binary mixture with 10 mole% impurity of Ar at 253 K and pressures ranging from 20 bar to 200 bar.

P/ [bar]	c_V^{MC}/ [J/mol K]	c_V^{REFP}/ [J/mol K]	c_P^{MC}/ [J/mol K]	c_P^{REFP}/ [J/mol K]	c^{MC}/ [m/s]	c^{REFP}/ [m/s]	η^{MD}/ [μPa s]	η^{REFP}/ [μPa s]
20	28.5	29.9	45.3 ± 0.2	48.7	241.1 ± 1.1	228.8	12.1 ± 2.4	13.6
40	37.3 ± 0.3	–	97.4 ± 2.1	–	684.5 ± 22.0	–	148.9 ± 5.5	27.6
60	37.0 ± 0.3	–	94.3 ± 1.1	–	707.4 ± 12.8	–	150.8 ± 3.9	83.5
80	37.1 ± 0.3	38.0	93.1 ± 2.2	90.1	728.2 ± 15.7	655.4	157.2 ± 3.0	119.5
100	37.2 ± 0.1	38.0	90.8 ± 3.5	87.7	746.6 ± 25.9	678.7	137.4 ± 15.4	123.3
120	37.0 ± 0.2	37.9	88.5 ± 0.8	85.8	775.0 ± 9.4	700.0	133.8 ± 4.7	127.0
140	37.2 ± 0.3	38.0	83.5 ± 2.8	84.1	795.5 ± 23.3	719.8	142.6 ± 14.4	130.5
160	37.2 ± 0.1	38.0	85.5 ± 1.7	82.7	810.5 ± 15.9	738.2	137.9 ± 4.7	133.9
180	37.5 ± 0.2	38.0	82.5 ± 2.0	81.5	835.7 ± 15.9	755.6	146.2 ± 11.1	137.2
200	37.2 ± 0.2	38.0	83.5 ± 2.4	80.4	854.1 ± 21.9	772.0	143.5 ± 6.3	140.4

Table S115: Densities computed from MC and MD simulations (ρ^{MC} and ρ^{MD}), densities obtained from REFPROP[10] (ρ^{REFP}), isothermal compressibilities computed from MC simulations (β_T^{MC}), isothermal compressibilities obtained from REFPROP[10] (β_T^{REFP}), thermal expansion coefficients computed from MC simulations (α_P^{MC}), thermal expansion coefficients obtained from REFPROP[10] (α_P^{REFP}), Joule Thomson coefficients computed from MC simulations (μ_{JT}^{MC}), and Joule Thomson coefficients obtained from REFPROP[10] (μ_{JT}^{REFP}) of CO_2-Ar binary mixture with 10 mole% impurity of Ar at 273 K and pressures ranging from 20 bar to 200 bar.

P/ [bar]	ρ^{MC}/ [kg/m^3]	ρ^{MD}/ [kg/m^3]	ρ^{REFP}/ [kg/m^3]	β_T^{MC}/ [10^{-5}/bar]	β_T^{REFP}/ [10^{-5}/bar]	α_P^{MC}/ [10^{-4}/K]	α_P^{REFP}/ [10^{-4}/K]	μ_{JT}^{MC}/ [10^{-3} K/bar]	μ_{JT}^{REFP}/ [10^{-3} K/bar]
20	39.7	45.4 ± 0.1	44.0	5711.3 ± 41.1	5803.9	54.6 ± 0.4	56.6	1189.7 ± 30.1	1229.9
40	96.3 ± 0.1	121.3 ± 0.5	108.9	3792.2 ± 29.9	3998.3	105.9 ± 1.1	117.0	1199.8 ± 21.1	1222.4
60	785.8 ± 3.4	940.0 ± 0.9	515.0	181.0 ± 34.5	3057.0	108.3 ± 14.6	215.7	77.7 ± 16.8	–
80	809.9 ± 2.6	951.2 ± 1.6	878.0	123.9 ± 14.7	141.2	83.0 ± 8.3	86.8	55.9 ± 10.5	61.5
100	827.4 ± 1.5	900.2 ± 1.1	900.0	97.1 ± 4.8	109.3	69.2 ± 2.5	72.7	42.3 ± 3.3	46.8
120	842.5 ± 1.8	915.3 ± 1.2	917.9	78.6 ± 3.8	89.9	60.1 ± 3.3	63.6	31.7 ± 4.6	36.4
140	854.6 ± 1.2	929.0 ± 0.9	933.3	70.4 ± 1.7	76.8	56.1 ± 1.6	57.3	26.7 ± 2.3	28.6
160	866.6 ± 1.8	943.1 ± 1.0	946.8	61.2 ± 3.2	67.2	51.0 ± 2.0	52.5	20.2 ± 2.8	22.4
180	875.9 ± 2.1	953.7 ± 1.2	958.9	56.6 ± 1.8	59.9	48.7 ± 1.5	48.7	17.1 ± 2.2	17.2
200	886.4 ± 0.4	964.4 ± 1.0	969.9	50.6 ± 2.1	54.2	45.5 ± 2.0	45.6	12.7 ± 2.9	13.0

Table S116: Heat capacities at constant volume computed from MC simulations (c_V^{MC}), heat capacities at constant volume obtained from REFPROP[10] (c_V^{REFP}), heat capacities at constant pressure computed from MC simulations (c_P^{MC}), heat capacities at constant pressure obtained from REFPROP[10] (c_P^{REFP}), speed of sound computed from MC simulations (c^{MC}), speed of sound obtained from REFPROP[10] (c^{REFP}), viscosities computed from MD simulations (η^{MD}), and viscosities obtained from REFPROP[10] (η^{REFP}) of CO_2-Ar binary mixture with 10 mole% impurity of Ar at 273 K and pressures ranging from 20 bar to 200 bar.

P/ [bar]	c_V^{MC}/ [J/mol K]	c_V^{REFP}/ [J/mol K]	c_P^{MC}/ [J/mol K]	c_P^{REFP}/ [J/mol K]	c^{MC}/ [m/s]	c^{REFP}/ [m/s]	η^{MD}/ [μPa s]	η^{REFP}/ [μPa s]
20	27.9	29.0	41.5 ± 0.2	43.9	256.0 ± 1.1	243.7	4.7 ± 3.1	14.6
40	32.6 ± 0.1	34.5	65.4 ± 0.5	71.9	234.5 ± 1.4	219.0	14.5 ± 0.7	15.5
60	37.1 ± 0.2	–	128.3 ± 8.9	–	493.0 ± 50.0	–	111.4 ± 2.7	34.5
80	37.0 ± 0.1	38.3	112.1 ± 6.6	110.6	549.8 ± 36.5	482.5	118.9 ± 7.8	84.2
100	36.6 ± 0.3	37.9	101.6 ± 1.6	101.8	587.6 ± 15.4	522.5	103.1 ± 11.5	89.3
120	36.6 ± 0.2	37.7	95.8 ± 3.5	96.2	629.0 ± 19.3	555.7	95.7 ± 3.0	93.7
140	36.6 ± 0.2	37.6	93.4 ± 2.0	92.1	651.4 ± 10.4	584.6	108.3 ± 11.8	97.7
160	36.6 ± 0.2	37.5	89.7 ± 1.7	89.0	679.9 ± 18.7	610.3	113.2 ± 20.4	101.5
180	36.6 ± 0.2	37.5	88.5 ± 1.9	86.5	698.4 ± 13.3	633.6	111.1 ± 4.1	104.9
200	36.7 ± 0.3	37.5	86.2 ± 2.5	84.5	723.4 ± 18.5	655.1	118.3 ± 5.3	108.2

Table S117: Densities computed from MC and MD simulations (ρ^{MC} and ρ^{MD}), densities obtained from REFPROP[10] (ρ^{REFP}), isothermal compressibilities computed from MC simulations (β_T^{MC}), isothermal compressibilities obtained from REFPROP[10] (β_T^{REFP}), thermal expansion coefficients computed from MC simulations (α_P^{MC}), thermal expansion coefficients obtained from REFPROP[10] (α_P^{REFP}), Joule Thomson coefficients computed from MC simulations ($\mu_{\mathrm{JT}}^{\mathrm{MC}}$), and Joule Thomson coefficients obtained from REFPROP[10] ($\mu_{\mathrm{JT}}^{\mathrm{REFP}}$) of CO_2-Ar binary mixture with 10 mole% impurity of Ar at 293 K and pressures ranging from 20 bar to 200 bar.

P/ [bar]	ρ^{MC}/ [kg/m^3]	ρ^{MD}/ [kg/m^3]	ρ^{REFP}/ [kg/m^3]	β_T^{MC}/ [10^{-5}/bar]	β_T^{REFP}/ [10^{-5}/bar]	α_P^{MC}/ [10^{-4}/K]	α_P^{REFP}/ [10^{-4}/K]	$\mu_{\mathrm{JT}}^{\mathrm{MC}}$/ [$10^{-3}$ K/bar]	$\mu_{\mathrm{JT}}^{\mathrm{REFP}}$/ [$10^{-3}$ K/bar]
20	35.9	40.9 ± 0.1	39.6	5520.4 ± 23.7	5576.0	46.4 ± 0.2	47.5	1003.3 ± 15.7	1026.3
40	81.3 ± 0.1	96.2 ± 0.5	90.8	3243.4 ± 51.0	3306.1	70.3 ± 1.0	73.5	1010.2 ± 29.7	1014.2
60	147.1 ± 0.3	666.0 ± 152.2	168.1	2936.4 ± 52.5	3096.8	135.2 ± 2.8	149.6	965.7 ± 30.7	962.6
80	422.8 ± 20.9	816.1 ± 2.0	441.9	$10\,922.3 \pm 1191.1$	13 960.3	1861.8 ± 178.1	2267.2	526.6 ± 79.9	–
100	666.6 ± 6.8	720.6 ± 2.7	720.4	429.3 ± 40.5	511.7	170.2 ± 11.7	188.0	151.7 ± 14.6	167.8
120	709.8 ± 1.7	772.4 ± 2.8	774.3	276.8 ± 27.0	265.4	128.3 ± 9.5	117.8	114.5 ± 12.5	110.6
140	743.4 ± 1.6	803.4 ± 0.9	808.9	164.4 ± 12.3	181.9	88.9 ± 5.2	90.9	76.9 ± 7.8	81.8
160	762.2 ± 1.4	829.9 ± 1.5	835.0	135.8 ± 9.5	139.4	78.6 ± 4.6	76.0	64.5 ± 7.0	63.4
180	782.5 ± 2.6	850.2 ± 0.9	856.2	108.3 ± 8.0	113.3	67.6 ± 4.7	66.4	50.5 ± 7.4	50.4
200	798.9 ± 1.2	867.7 ± 1.0	874.2	90.7 ± 4.6	95.7	60.0 ± 2.8	59.4	40.4 ± 4.5	40.6

Table S118: Heat capacities at constant volume computed from MC simulations (c_V^{MC}), heat capacities at constant volume obtained from REFPROP[10] (c_V^{REFP}), heat capacities at constant pressure computed from MC simulations (c_P^{MC}), heat capacities at constant pressure obtained from REFPROP[10] (c_P^{REFP}), speed of sound computed from MC simulations (c^{MC}), speed of sound obtained from REFPROP[10] (c^{REFP}), viscosities computed from MD simulations (η^{MD}), and viscosities obtained from REFPROP[10] (η^{REFP}) of CO_2-Ar binary mixture with 10 mole% impurity of Ar at 293 K and pressures ranging from 20 bar to 200 bar.

P/ [bar]	c_V^{MC}/ [J/mol K]	c_V^{REFP}/ [J/mol K]	c_P^{MC}/ [J/mol K]	c_P^{REFP}/ [J/mol K]	c^{MC}/ [m/s]	c^{REFP}/ [m/s]	η^{MD}/ [μPa s]	η^{REFP}/ [μPa s]
20	27.9	29.0	39.9 ± 0.1	42.0	268.3 ± 0.6	256.2	9.0 ± 2.8	15.7
40	30.4	31.7	51.6 ± 0.3	54.7	253.7 ± 2.1	239.9	15.9 ± 2.0	16.3
60	34.7 ± 0.4	36.3	83.4 ± 1.4	91.2	236.0 ± 3.2	219.8	56.5 ± 21.0	17.9
80	44.2 ± 1.1	–	965.5 ± 102.1	–	217.8 ± 17.6	–	76.4 ± 1.2	30.2
100	37.5 ± 0.4	40.1	157.7 ± 6.7	162.7	383.7 ± 20.0	331.5	64.8 ± 8.5	57.3
120	37.2 ± 0.2	38.6	135.9 ± 5.8	124.9	431.2 ± 23.0	396.7	66.9 ± 3.2	65.2
140	36.6 ± 0.3	38.0	112.2 ± 3.8	109.7	500.6 ± 20.7	443.2	71.2 ± 1.9	71.0
160	36.5 ± 0.3	37.6	106.2 ± 3.6	101.1	530.3 ± 20.6	480.6	75.8 ± 2.6	75.7
180	36.2 ± 0.2	37.4	99.1 ± 4.3	95.4	568.4 ± 24.4	512.5	80.7 ± 4.0	79.9
200	36.1 ± 0.2	37.3	94.0 ± 2.7	91.2	599.9 ± 17.4	540.6	88.3 ± 4.4	83.6

Table S119: Densities computed from MC and MD simulations (ρ^{MC} and ρ^{MD}), densities obtained from REFPROP[10] (ρ^{REFP}), isothermal compressibilities computed from MC simulations (β_T^{MC}), isothermal compressibilities obtained from REFPROP[10] (β_T^{REFP}), thermal expansion coefficients computed from MC simulations (α_P^{MC}), thermal expansion coefficients obtained from REFPROP[10] (α_P^{REFP}), Joule Thomson coefficients computed from MC simulations (μ_{JT}^{MC}), and Joule Thomson coefficients obtained from REFPROP[10] (μ_{JT}^{REFP}) of CO_2-Ar binary mixture with 10 mole% impurity of Ar at 313 K and pressures ranging from 20 bar to 200 bar.

P/ [bar]	ρ^{MC}/ [kg/m^3]	ρ^{MD}/ [kg/m^3]	ρ^{REFP}/ [kg/m^3]	β_T^{MC}/ [10^{-5}/bar]	β_T^{REFP}/ [10^{-5}/bar]	α_P^{MC}/ [10^{-4}/K]	α_P^{REFP}/ [10^{-4}/K]	μ_{JT}^{MC}/ [10^{-3} K/bar]	μ_{JT}^{REFP}/ [10^{-3} K/bar]
20	32.9	37.3 ± 0.1	36.3	5390.4 ± 25.7	5432.4	40.7 ± 0.2	41.5	851.7 ± 20.1	870.1
40	71.9	83.4 ± 0.1	79.9	2975.7 ± 25.1	3033.9	54.3 ± 0.5	56.5	839.7 ± 18.9	856.3
60	120.7 ± 0.1	146.6 ± 1.0	135.3	2324.9 ± 50.9	2364.4	79.5 ± 1.9	83.2	831.5 ± 34.2	824.4
80	186.8 ± 0.4	279.0 ± 7.9	212.9	2164.3 ± 57.8	2241.0	130.3 ± 3.4	140.1	760.1 ± 29.7	751.5
100	293.8 ± 2.3	330.2 ± 3.5	339.5	2512.8 ± 136.0	2429.6	277.8 ± 17.3	277.5	617.9 ± 53.9	590.5
120	458.3 ± 4.9	499.1 ± 3.2	514.6	1607.4 ± 178.8	1456.1	325.1 ± 34.2	294.1	364.6 ± 51.8	–
140	565.2 ± 3.5	611.4 ± 4.7	624.5	684.1 ± 16.7	635.2	199.1 ± 6.2	179.6	222.6 ± 10.3	215.1
160	622.3 ± 1.7	678.6 ± 2.2	686.9	374.3 ± 18.9	361.3	133.5 ± 6.7	124.5	153.9 ± 11.6	149.0
180	664.3 ± 1.4	720.1 ± 1.8	728.9	236.2 ± 18.3	246.3	98.3 ± 7.3	97.1	110.6 ± 13.5	111.6
200	691.7 ± 2.2	750.0 ± 2.0	760.6	186.0 ± 7.7	185.7	85.5 ± 3.5	81.0	90.8 ± 6.5	87.4

Table S120: Heat capacities at constant volume computed from MC simulations (c_V^{MC}), heat capacities at constant volume obtained from REFPROP[10] (c_V^{REFP}), heat capacities at constant pressure computed from MC simulations (c_P^{MC}), heat capacities at constant pressure obtained from REFPROP[10] (c_P^{REFP}), speed of sound computed from MC simulations (c^{MC}), speed of sound obtained from REFPROP[10] (c^{REFP}), viscosities computed from MD simulations (η^{MD}), and viscosities obtained from REFPROP[10] (η^{REFP}) of CO_2-Ar binary mixture with 10 mole% impurity of Ar at 313 K and pressures ranging from 20 bar to 200 bar.

P/ [bar]	c_V^{MC}/ [J/mol K]	c_V^{REFP}/ [J/mol K]	c_P^{MC}/ [J/mol K]	c_P^{REFP}/ [J/mol K]	c^{MC}/ [m/s]	c^{REFP}/ [m/s]	η^{MD}/ [μPa s]	η^{REFP}/ [μPa s]
20	28.3	29.3	39.2 ± 0.1	41.2	279.5 ± 0.7	267.2	13.6 ± 5.0	16.7
40	29.8	31.0	46.3 ± 0.2	49.0	269.4 ± 1.3	255.4	16.9 ± 1.8	17.2
60	31.8 ± 0.1	33.2	59.3 ± 0.7	62.8	257.7 ± 3.3	243.1	19.2 ± 2.3	18.3
80	34.6 ± 0.2	36.1	86.8 ± 1.5	92.2	249.1 ± 4.0	231.4	20.7 ± 0.5	20.3
100	37.6 ± 0.4	39.8	169.6 ± 8.7	167.2	247.3 ± 9.3	225.8	23.5 ± 0.9	25.4
120	38.8 ± 0.7	–	219.7 ± 17.6	–	277.4 ± 19.3	–	34.8 ± 1.8	36.6
140	37.4 ± 0.3	39.1	166.4 ± 4.5	150.0	339.5 ± 6.4	311.2	43.5 ± 3.0	46.7
160	36.4 ± 0.2	38.2	132.7 ± 4.9	123.4	395.6 ± 12.4	361.0	52.1 ± 2.2	53.8
180	36.3 ± 0.1	37.7	113.0 ± 5.7	109.4	445.3 ± 20.6	402.2	59.2 ± 4.9	59.3
200	36.1 ± 0.2	37.4	106.8 ± 2.9	100.8	479.5 ± 12.1	437.2	67.4 ± 6.2	63.8

S15.7 Thermodynamic and Transport Properties Data of binary CO_2 mixture with 1 mole% Impurity of H_2

Table S121: Densities computed from MC and MD simulations (ρ^{MC} and ρ^{MD}), densities obtained from REFPROP[10] (ρ^{REFP}), isothermal compressibilities computed from MC simulations (β_T^{MC}), isothermal compressibilities obtained from REFPROP[10] (β_T^{REFP}), thermal expansion coefficients computed from MC simulations (α_P^{MC}), thermal expansion coefficients obtained from REFPROP[10] (α_P^{REFP}), Joule Thomson coefficients computed from MC simulations (μ_{JT}^{MC}), and Joule Thomson coefficients obtained from REFPROP[10] (μ_{JT}^{REFP}) of CO_2-H_2 binary mixture with 1 mole% impurity of H_2 at 253 K and pressures ranging from 20 bar to 200 bar.

P/ [bar]	ρ^{MC}/ [kg/m^3]	ρ^{MD}/ [kg/m^3]	ρ^{REFP}/ [kg/m^3]	β_T^{MC}/ [10^{-5}/bar]	β_T^{REFP}/ [10^{-5}/bar]	α_P^{MC}/ [10^{-4}/K]	α_P^{REFP}/ [10^{-4}/K]	μ_{JT}^{MC}/ [10^{-3} K/bar]	μ_{JT}^{REFP}/ [10^{-3} K/bar]
20	50.4	51.5 ± 0.1	77.3	6401.5 ± 50.3	6608.5	77.0 ± 0.8	150.6	1569.0 ± 35.5	–
40	1020.7 ± 0.3	1020.2 ± 0.9	1020.5	39.3 ± 1.2	43.9	45.3 ± 1.5	46.0	6.5 ± 1.7	7.5
60	1029.2 ± 0.8	1026.6 ± 0.9	1029.1	34.4 ± 0.9	40.2	40.9 ± 1.1	43.4	1.5 ± 1.3	4.5
80	1036.5 ± 1.4	1034.1 ± 0.7	1037.0	33.2 ± 2.1	37.1	40.3 ± 2.4	41.1	0.9 ± 2.7	1.9
100	1044.2 ± 1.1	1041.9 ± 0.4	1044.5	30.1 ± 0.8	34.5	37.4 ± 0.7	39.2	-2.5 ± 0.8	-0.4
120	1049.5 ± 0.7	1048.8 ± 0.6	1051.5	29.9 ± 0.6	32.3	38.0 ± 0.7	37.6	-1.8 ± 0.9	-2.4
140	1055.5 ± 0.9	1054.3 ± 0.7	1058.1	28.1 ± 0.5	30.4	36.0 ± 0.7	36.1	-4.1 ± 0.8	-4.2
160	1061.4 ± 0.7	1060.5 ± 1.1	1064.4	26.1 ± 1.3	28.8	34.3 ± 1.5	34.8	-6.3 ± 1.8	-5.8
180	1067.8 ± 0.9	1066.0 ± 0.7	1070.3	25.1 ± 0.8	27.3	33.9 ± 1.1	33.7	-6.7 ± 1.3	-7.2
200	1073.1 ± 0.8	1070.9 ± 0.8	1076.1	23.4 ± 0.9	26.0	32.0 ± 1.3	32.6	-9.1 ± 1.5	-8.5

Table S122: Heat capacities at constant volume computed from MC simulations (c_V^{MC}), heat capacities at constant volume obtained from REFPROP[10] (c_V^{REFP}), heat capacities at constant pressure computed from MC simulations (c_P^{MC}), heat capacities at constant pressure obtained from REFPROP[10] (c_P^{REFP}), speed of sound computed from MC simulations (c^{MC}), speed of sound obtained from REFPROP[10] (c^{REFP}), viscosities computed from MD simulations (η^{MD}), and viscosities obtained from REFPROP[10] (η^{REFP}) of CO_2-H_2 binary mixture with 1 mole% impurity of H_2 at 253 K and pressures ranging from 20 bar to 200 bar.

P/ [bar]	c_V^{MC}/ [J/mol K]	c_V^{REFP}/ [J/mol K]	c_P^{MC}/ [J/mol K]	c_P^{REFP}/ [J/mol K]	c^{MC}/ [m/s]	c^{REFP}/ [m/s]	η^{MD}/ [μPa s]	η^{REFP}/ [μPa s]
20	32.0 ± 0.1	–	52.3 ± 0.3	–	225.2 ± 1.1	–	11.5 ± 3.6	13.2
40	40.7 ± 0.2	40.9	97.3 ± 2.2	93.0	772.2 ± 14.7	712.8	147.7 ± 3.6	136.7
60	40.7 ± 0.2	40.9	92.5 ± 2.0	91.1	800.4 ± 13.3	734.1	152.7 ± 17.4	140.6
80	40.8 ± 0.2	40.9	92.6 ± 3.1	89.4	811.6 ± 29.7	753.8	146.8 ± 13.9	144.4
100	40.6 ± 0.2	40.9	89.9 ± 0.6	88.0	839.6 ± 12.0	772.3	159.2 ± 10.4	148.0
120	40.7 ± 0.2	40.9	91.5 ± 1.1	86.7	846.3 ± 10.6	789.6	169.7 ± 13.3	151.5
140	40.8 ± 0.2	41.0	89.0 ± 1.0	85.6	857.2 ± 9.7	806.0	166.7 ± 11.2	154.9
160	40.9 ± 0.3	41.0	87.4 ± 1.7	84.7	877.7 ± 23.6	821.5	192.4 ± 35.9	158.3
180	41.0 ± 0.2	41.0	88.0 ± 1.6	83.8	894.7 ± 17.1	836.4	165.7 ± 8.1	161.6
200	41.0 ± 0.3	41.1	85.8 ± 1.8	83.1	913.2 ± 21.2	850.6	179.8 ± 15.5	164.8

Table S123: Densities computed from MC and MD simulations (ρ^{MC} and ρ^{MD}), densities obtained from REFPROP[10] (ρ^{REFP}), isothermal compressibilities computed from MC simulations (β_T^{MC}), isothermal compressibilities obtained from REFPROP[10] (β_T^{REFP}), thermal expansion coefficients computed from MC simulations (α_P^{MC}), thermal expansion coefficients obtained from REFPROP[10] (α_P^{REFP}), Joule Thomson coefficients computed from MC simulations ($\mu_{\mathrm{JT}}^{\mathrm{MC}}$), and Joule Thomson coefficients obtained from REFPROP[10] ($\mu_{\mathrm{JT}}^{\mathrm{REFP}}$) of CO_2-H_2 binary mixture with 1 mole% impurity of H_2 at 273 K and pressures ranging from 20 bar to 200 bar.

P/ [bar]	ρ^{MC}/ [kg/m^3]	ρ^{MD}/ [kg/m^3]	ρ^{REFP}/ [kg/m^3]	β_T^{MC}/ [10^{-5}/bar]	β_T^{REFP}/ [10^{-5}/bar]	α_P^{MC}/ [10^{-4}/K]	α_P^{REFP}/ [10^{-4}/K]	$\mu_{\mathrm{JT}}^{\mathrm{MC}}$/ [$10^{-3}$ K/bar]	$\mu_{\mathrm{JT}}^{\mathrm{REFP}}$/ [$10^{-3}$ K/bar]
20	44.1	45.1 ± 0.1	45.0	5875.9 ± 31.4	5995.0	58.3 ± 0.3	61.6	1255.7 ± 17.2	1373.2
40	916.7 ± 1.9	912.3 ± 1.4	620.4	85.8 ± 3.8	-172.3	69.5 ± 2.6	102.2	37.5 ± 3.1	–
60	929.9 ± 2.8	926.4 ± 1.2	926.4	76.0 ± 9.5	86.4	64.6 ± 7.0	65.7	32.4 ± 8.4	35.6
80	944.6 ± 1.6	939.7 ± 1.2	941.2	59.3 ± 2.7	72.8	52.9 ± 2.1	58.4	20.5 ± 2.6	27.6
100	953.7 ± 2.2	953.0 ± 0.6	954.0	58.6 ± 5.2	63.3	53.6 ± 4.2	53.1	20.8 ± 5.2	21.4
120	966.0 ± 1.1	963.3 ± 0.8	965.5	49.7 ± 2.1	56.2	47.5 ± 1.6	49.0	13.9 ± 2.1	16.4
140	974.2 ± 1.0	971.7 ± 1.1	975.8	45.8 ± 1.7	50.6	45.2 ± 1.5	45.8	11.0 ± 1.9	12.3
160	983.8 ± 1.6	981.3 ± 0.8	985.3	41.8 ± 2.2	46.2	42.6 ± 1.7	43.1	7.8 ± 2.2	8.8
180	991.1 ± 1.2	988.7 ± 0.9	994.1	38.1 ± 1.1	42.5	39.5 ± 1.1	40.8	3.9 ± 1.5	5.8
200	998.9 ± 0.9	996.8 ± 0.3	1002.2	37.4 ± 1.3	39.5	40.0 ± 1.1	38.9	4.4 ± 1.4	3.2

Table S124: Heat capacities at constant volume computed from MC simulations (c_V^{MC}), heat capacities at constant volume obtained from REFPROP[10] (c_V^{REFP}), heat capacities at constant pressure computed from MC simulations (c_P^{MC}), heat capacities at constant pressure obtained from REFPROP[10] (c_P^{REFP}), speed of sound computed from MC simulations (c^{MC}), speed of sound obtained from REFPROP[10] (c^{REFP}), viscosities computed from MD simulations (η^{MD}), and viscosities obtained from REFPROP[10] (η^{REFP}) of CO_2-H_2 binary mixture with 1 mole% impurity of H_2 at 273 K and pressures ranging from 20 bar to 200 bar.

P/ [bar]	c_V^{MC}/ [J/mol K]	c_V^{REFP}/ [J/mol K]	c_P^{MC}/ [J/mol K]	c_P^{REFP}/ [J/mol K]	c^{MC}/ [m/s]	c^{REFP}/ [m/s]	η^{MD}/ [μPa s]	η^{REFP}/ [μPa s]
20	31.0	31.4	46.6 ± 0.1	48.1	240.7 ± 0.7	238.3	7.1 ± 4.0	13.9
40	39.9 ± 0.2	–	113.8 ± 2.4	–	602.2 ± 14.9	–	102.4 ± 2.8	48.4
60	40.1 ± 0.2	40.7	110.5 ± 7.0	104.8	624.4 ± 43.7	567.2	106.5 ± 3.2	101.0
80	39.9 ± 0.2	40.6	100.0 ± 2.1	99.8	668.8 ± 16.6	599.2	110.8 ± 2.6	105.5
100	40.1 ± 0.2	40.5	101.6 ± 4.5	96.1	673.8 ± 33.3	627.1	116.7 ± 5.0	109.5
120	39.8 ± 0.2	40.4	96.1 ± 1.7	93.2	709.2 ± 16.5	652.1	126.5 ± 21.7	113.3
140	39.9 ± 0.2	40.4	94.6 ± 1.8	90.9	729.3 ± 15.5	674.9	165.2 ± 78.9	116.9
160	40.1 ± 0.2	40.4	92.9 ± 1.7	88.9	751.0 ± 21.1	695.9	125.3 ± 6.0	120.3
180	40.0 ± 0.1	40.4	89.3 ± 1.4	87.3	768.8 ± 12.4	715.4	142.7 ± 18.4	123.6
200	40.2 ± 0.2	40.4	91.1 ± 1.0	85.9	779.0 ± 14.4	733.7	136.0 ± 8.4	126.8

Table S125: Densities computed from MC and MD simulations (ρ^{MC} and ρ^{MD}), densities obtained from REFPROP[10] (ρ^{REFP}), isothermal compressibilities computed from MC simulations (β_T^{MC}), isothermal compressibilities obtained from REFPROP[10] (β_T^{REFP}), thermal expansion coefficients computed from MC simulations (α_P^{MC}), thermal expansion coefficients obtained from REFPROP[10] (α_P^{REFP}), Joule Thomson coefficients computed from MC simulations ($\mu_{\mathrm{JT}}^{\mathrm{MC}}$), and Joule Thomson coefficients obtained from REFPROP[10] ($\mu_{\mathrm{JT}}^{\mathrm{REFP}}$) of CO_2-H_2 binary mixture with 1 mole% impurity of H_2 at 293 K and pressures ranging from 20 bar to 200 bar.

P/ [bar]	ρ^{MC}/ [kg/m^3]	ρ^{MD}/ [kg/m^3]	ρ^{REFP}/ [kg/m^3]	β_T^{MC}/ [10^{-5}/bar]	β_T^{REFP}/ [10^{-5}/bar]	α_P^{MC}/ [10^{-4}/K]	α_P^{REFP}/ [10^{-4}/K]	$\mu_{\mathrm{JT}}^{\mathrm{MC}}$/ [$10^{-3}$ K/bar]	$\mu_{\mathrm{JT}}^{\mathrm{REFP}}$/ [$10^{-3}$ K/bar]
20	39.7	40.5 ± 0.1	40.3	5616.1 ± 40.0	5692.2	48.5 ± 0.4	50.3	1041.4 ± 28.6	1134.5
40	92.4 ± 0.1	94.4 ± 0.4	95.8	3440.0 ± 39.2	3603.5	80.5 ± 1.2	88.9	1064.0 ± 29.4	1135.8
60	473.0 ± 258.1	707.8 ± 88.0	408.2	2576.5 ± 1928.5	$-10\,943.8$	239.7 ± 40.3	-1886.5	503.2 ± 297.0	–
80	809.4 ± 2.7	803.8 ± 1.7	800.2	199.0 ± 11.0	247.7	111.2 ± 7.2	121.8	87.4 ± 9.5	101.8
100	835.9 ± 2.6	835.1 ± 2.1	832.6	134.1 ± 10.4	162.9	83.3 ± 4.9	90.5	62.7 ± 6.5	73.2
120	856.8 ± 2.1	854.4 ± 2.1	856.4	112.5 ± 3.9	123.4	74.2 ± 2.5	74.8	52.7 ± 3.7	56.1
140	873.8 ± 1.6	873.0 ± 1.1	875.7	92.0 ± 4.4	100.2	64.4 ± 2.2	64.9	41.7 ± 3.1	44.2
160	889.1 ± 1.4	888.5 ± 1.4	891.9	77.5 ± 3.4	84.7	57.6 ± 2.1	58.1	33.2 ± 3.0	35.3
180	902.4 ± 1.3	900.6 ± 0.7	906.1	67.5 ± 1.3	73.6	52.9 ± 0.9	52.9	27.0 ± 1.3	28.3
200	913.9 ± 1.5	913.5 ± 0.6	918.7	63.1 ± 2.1	65.2	50.4 ± 1.3	48.9	23.6 ± 1.9	22.6

Table S126: Heat capacities at constant volume computed from MC simulations (c_V^{MC}), heat capacities at constant volume obtained from REFPROP[10] (c_V^{REFP}), heat capacities at constant pressure computed from MC simulations (c_P^{MC}), heat capacities at constant pressure obtained from REFPROP[10] (c_P^{REFP}), speed of sound computed from MC simulations (c^{MC}), speed of sound obtained from REFPROP[10] (c^{REFP}), viscosities computed from MD simulations (η^{MD}), and viscosities obtained from REFPROP[10] (η^{REFP}) of CO_2-H_2 binary mixture with 1 mole% impurity of H_2 at 293 K and pressures ranging from 20 bar to 200 bar.

P/ [bar]	c_V^{MC}/ [J/mol K]	c_V^{REFP}/ [J/mol K]	c_P^{MC}/ [J/mol K]	c_P^{REFP}/ [J/mol K]	c^{MC}/ [m/s]	c^{REFP}/ [m/s]	η^{MD}/ [μPa s]	η^{REFP}/ [μPa s]
20	31.0	31.1	44.4 ± 0.1	45.2	253.7 ± 1.0	251.7	7.5 ± 4.2	14.9
40	34.2 ± 0.1	35.1	60.2 ± 0.5	64.3	235.4 ± 1.7	230.5	16.3 ± 0.8	15.6
60	51.8 ± 3.1	–	175.5 ± 21.0	–	210.3 ± 98.3	–	59.8 ± 8.4	27.7
80	40.1 ± 0.3	41.9	139.2 ± 7.7	137.5	464.1 ± 18.3	407.1	79.3 ± 3.3	71.8
100	39.7 ± 0.2	41.0	119.8 ± 3.6	118.1	518.7 ± 21.7	461.1	86.8 ± 6.1	78.0
120	39.7 ± 0.1	40.6	113.3 ± 3.4	108.1	543.9 ± 12.5	502.2	86.7 ± 2.2	83.1
140	39.6 ± 0.1	40.3	106.1 ± 1.7	101.7	577.1 ± 14.5	536.3	90.3 ± 2.5	87.5
160	39.7 ± 0.2	40.2	101.3 ± 2.0	97.2	608.7 ± 14.9	566.0	96.0 ± 6.0	91.4
180	39.6 ± 0.1	40.1	98.5 ± 0.8	93.8	639.2 ± 6.8	592.4	99.0 ± 2.3	95.0
200	39.6 ± 0.1	40.0	96.0 ± 1.5	91.1	648.5 ± 11.9	616.4	101.2 ± 3.0	98.4

Table S127: Densities computed from MC and MD simulations (ρ^{MC} and ρ^{MD}), densities obtained from REFPROP[10] (ρ^{REFP}), isothermal compressibilities computed from MC simulations (β_T^{MC}), isothermal compressibilities obtained from REFPROP[10] (β_T^{REFP}), thermal expansion coefficients computed from MC simulations (α_P^{MC}), thermal expansion coefficients obtained from REFPROP[10] (α_P^{REFP}), Joule Thomson coefficients computed from MC simulations ($\mu_{\mathrm{JT}}^{\mathrm{MC}}$), and Joule Thomson coefficients obtained from REFPROP[10] ($\mu_{\mathrm{JT}}^{\mathrm{REFP}}$) of CO_2-H_2 binary mixture with 1 mole% impurity of H_2 at 313 K and pressures ranging from 20 bar to 200 bar.

P/ [bar]	ρ^{MC}/ [kg/m^3]	ρ^{MD}/ [kg/m^3]	ρ^{REFP}/ [kg/m^3]	β_T^{MC}/ [10^{-5}/bar]	β_T^{REFP}/ [10^{-5}/bar]	α_P^{MC}/ [10^{-4}/K]	α_P^{REFP}/ [10^{-4}/K]	$\mu_{\mathrm{JT}}^{\mathrm{MC}}$/ [$10^{-3}$ K/bar]	$\mu_{\mathrm{JT}}^{\mathrm{REFP}}$/ [$10^{-3}$ K/bar]
20	36.3	37.0 ± 0.1	36.7	5479.8 ± 17.7	5511.9	42.3 ± 0.2	43.2	893.4 ± 16.9	955.1
40	80.4 ± 0.1	82.2 ± 0.2	82.6	3102.3 ± 28.3	3176.2	59.4 ± 0.5	63.1	886.9 ± 18.2	948.2
60	139.4 ± 0.3	143.1 ± 0.4	146.0	2531.3 ± 52.4	2687.0	96.3 ± 1.9	108.1	876.9 ± 28.0	916.6
80	237.4 ± 1.5	262.9 ± 8.8	262.3	2952.4 ± 150.5	3529.6	227.6 ± 13.5	303.5	791.2 ± 65.5	–
100	550.3 ± 8.5	559.0 ± 7.9	579.6	2778.7 ± 222.7	1716.9	626.8 ± 44.3	420.2	362.8 ± 36.1	–
120	691.2 ± 3.1	686.2 ± 2.3	688.8	423.9 ± 50.5	476.9	161.5 ± 17.1	166.5	156.4 ± 23.6	169.2
140	738.5 ± 2.6	735.0 ± 1.0	739.4	249.5 ± 9.0	270.2	112.3 ± 3.3	112.2	110.7 ± 4.8	116.5
160	769.6 ± 3.0	766.4 ± 2.7	773.4	181.6 ± 11.3	189.2	90.3 ± 5.2	88.3	86.2 ± 8.3	87.8
180	794.4 ± 2.4	793.5 ± 2.2	799.4	142.8 ± 10.4	146.0	78.2 ± 5.5	74.4	69.9 ± 9.0	69.0
200	815.9 ± 1.4	813.0 ± 2.0	820.8	116.4 ± 4.5	119.0	68.0 ± 2.3	65.1	56.7 ± 3.7	55.6

Table S128: Heat capacities at constant volume computed from MC simulations (c_V^{MC}), heat capacities at constant volume obtained from REFPROP[10] (c_V^{REFP}), heat capacities at constant pressure computed from MC simulations (c_P^{MC}), heat capacities at constant pressure obtained from REFPROP[10] (c_P^{REFP}), speed of sound computed from MC simulations (c^{MC}), speed of sound obtained from REFPROP[10] (c^{REFP}), viscosities computed from MD simulations (η^{MD}), and viscosities obtained from REFPROP[10] (η^{REFP}) of CO_2-H_2 binary mixture with 1 mole% impurity of H_2 at 313 K and pressures ranging from 20 bar to 200 bar.

P/ [bar]	c_V^{MC}/ [J/mol K]	c_V^{REFP}/ [J/mol K]	c_P^{MC}/ [J/mol K]	c_P^{REFP}/ [J/mol K]	c^{MC}/ [m/s]	c^{REFP}/ [m/s]	η^{MD}/ [μPa s]	η^{REFP}/ [μPa s]
20	31.3	31.3	43.5 ± 0.1	43.9	264.7 ± 0.5	263.2	16.2 ± 1.3	15.9
40	33.2	33.6	52.4 ± 0.2	54.3	251.6 ± 1.3	248.3	16.1 ± 1.6	16.5
60	35.8 ± 0.1	37.0	71.9 ± 0.8	77.7	238.5 ± 2.8	231.3	17.0 ± 1.4	17.7
80	40.5 ± 0.5	–	142.2 ± 6.4	–	223.8 ± 7.8	–	21.5 ± 2.3	21.3
100	42.9 ± 0.5	–	406.8 ± 26.2	–	249.0 ± 13.0	–	37.8 ± 2.5	42.6
120	40.2 ± 0.2	42.3	163.5 ± 11.9	157.5	372.7 ± 26.1	336.6	57.8 ± 6.3	55.1
140	39.8 ± 0.2	41.1	134.1 ± 2.2	127.2	427.3 ± 8.6	393.4	65.1 ± 3.3	62.2
160	39.5 ± 0.2	40.5	120.0 ± 4.5	113.2	466.3 ± 17.0	436.8	68.6 ± 2.9	67.6
180	39.4 ± 0.1	40.2	113.6 ± 5.3	104.9	504.3 ± 21.8	472.7	74.1 ± 4.9	72.1
200	39.4 ± 0.1	40.0	106.4 ± 1.9	99.2	532.9 ± 11.3	503.8	78.8 ± 2.7	76.1

S15.8 Thermodynamic and Transport Properties Data of binary CO_2 mixture with 5 mole% Impurity of H_2

Table S129: Densities computed from MC and MD simulations (ρ^{MC} and ρ^{MD}), densities obtained from REFPROP[10] (ρ^{REFP}), isothermal compressibilities computed from MC simulations (β_T^{MC}), isothermal compressibilities obtained from REFPROP[10] (β_T^{REFP}), thermal expansion coefficients computed from MC simulations (α_P^{MC}), thermal expansion coefficients obtained from REFPROP[10] (α_P^{REFP}), Joule Thomson coefficients computed from MC simulations ($\mu_{\mathrm{JT}}^{\mathrm{MC}}$), and Joule Thomson coefficients obtained from REFPROP[10] ($\mu_{\mathrm{JT}}^{\mathrm{REFP}}$) of CO_2-H_2 binary mixture with 5 mole% impurity of H_2 at 253 K and pressures ranging from 20 bar to 200 bar.

P/ [bar]	ρ^{MC}/ [kg/m^3]	ρ^{MD}/ [kg/m^3]	ρ^{REFP}/ [kg/m^3]	β_T^{MC}/ [10^{-5}/bar]	β_T^{REFP}/ [10^{-5}/bar]	α_P^{MC}/ [10^{-4}/K]	α_P^{REFP}/ [10^{-4}/K]	$\mu_{\mathrm{JT}}^{\mathrm{MC}}$/ [$10^{-3}$ K/bar]	$\mu_{\mathrm{JT}}^{\mathrm{REFP}}$/ [$10^{-3}$ K/bar]
20	47.4	51.5 ± 0.1	48.7	6183.7 ± 32.4	6364.5	71.6 ± 0.5	76.8	1444.0 ± 22.5	1565.6
40	954.3 ± 2.0	1020.2 ± 0.9	516.2	56.7 ± 2.7	70.1	55.2 ± 2.6	-390.3	17.3 ± 2.9	–
60	966.3 ± 1.6	1026.6 ± 0.9	740.9	50.8 ± 1.4	-331.2	51.7 ± 1.9	-76.3	13.6 ± 2.2	–
80	974.7 ± 0.5	1034.1 ± 0.7	860.4	44.3 ± 1.5	201.5	46.4 ± 1.6	129.3	8.0 ± 1.9	–
100	982.8 ± 1.3	981.0 ± 0.7	930.9	42.7 ± 2.1	66.6	45.8 ± 2.2	57.8	7.2 ± 2.6	–
120	992.2 ± 1.3	989.3 ± 0.8	974.7	39.7 ± 1.6	41.2	44.1 ± 1.7	41.9	5.3 ± 2.0	3.0
140	999.4 ± 1.6	997.2 ± 0.9	982.5	34.8 ± 1.1	38.2	39.9 ± 1.3	39.8	0.4 ± 1.5	0.4
160	1004.8 ± 0.9	1004.1 ± 1.0	989.7	34.5 ± 1.4	35.6	40.0 ± 1.5	38.1	0.6 ± 1.7	-1.9
180	1012.0 ± 0.4	1010.9 ± 0.5	996.6	31.7 ± 1.6	33.4	38.0 ± 1.9	36.5	-1.8 ± 2.3	-3.9
200	1018.5 ± 1.3	1017.7 ± 0.5	1003.1	29.9 ± 0.9	31.5	36.2 ± 1.5	35.2	-4.0 ± 1.8	-5.7

Table S130: Heat capacities at constant volume computed from MC simulations (c_V^{MC}), heat capacities at constant volume obtained from REFPROP[10] (c_V^{REFP}), heat capacities at constant pressure computed from MC simulations (c_P^{MC}), heat capacities at constant pressure obtained from REFPROP[10] (c_P^{REFP}), speed of sound computed from MC simulations (c^{MC}), speed of sound obtained from REFPROP[10] (c^{REFP}), viscosities computed from MD simulations (η^{MD}), and viscosities obtained from REFPROP[10] (η^{REFP}) of CO_2-H_2 binary mixture with 5 mole% impurity of H_2 at 253 K and pressures ranging from 20 bar to 200 bar.

P/ [bar]	c_V^{MC}/ [J/mol K]	c_V^{REFP}/ [J/mol K]	c_P^{MC}/ [J/mol K]	c_P^{REFP}/ [J/mol K]	c^{MC}/ [m/s]	c^{REFP}/ [m/s]	η^{MD}/ [μPa s]	η^{REFP}/ [μPa s]
20	31.2	31.7	49.8 ± 0.2	51.8	233.4 ± 0.8	229.8	11.5 ± 3.6	13.0
40	40.4 ± 0.3	–	100.5 ± 2.9	–	678.2 ± 19.1	–	147.7 ± 3.6	34.4
60	40.1 ± 0.2	–	98.3 ± 2.9	–	706.0 ± 14.1	–	152.7 ± 17.4	64.4
80	40.1 ± 0.3	–	93.0 ± 2.0	–	732.9 ± 14.8	–	146.8 ± 13.9	86.9
100	40.2 ± 0.2	–	93.3 ± 2.7	–	743.9 ± 21.0	–	125.9 ± 4.8	107.9
120	40.2 ± 0.3	39.5	92.6 ± 1.8	85.8	764.0 ± 17.1	735.0	133.8 ± 7.1	124.4
140	39.9 ± 0.3	39.5	88.9 ± 1.6	84.4	801.2 ± 14.6	754.2	147.4 ± 18.3	127.7
160	40.1 ± 0.3	39.5	89.5 ± 1.9	83.1	802.8 ± 18.8	772.1	136.2 ± 3.7	130.8
180	40.6 ± 0.2	39.6	87.9 ± 2.3	82.0	821.8 ± 23.9	789.1	157.8 ± 27.1	133.9
200	40.3 ± 0.3	39.6	85.9 ± 2.6	81.1	835.8 ± 18.1	805.1	148.4 ± 7.4	136.9

Table S131: Densities computed from MC and MD simulations (ρ^{MC} and ρ^{MD}), densities obtained from REFPROP[10] (ρ^{REFP}), isothermal compressibilities computed from MC simulations (β_T^{MC}), isothermal compressibilities obtained from REFPROP[10] (β_T^{REFP}), thermal expansion coefficients computed from MC simulations (α_P^{MC}), thermal expansion coefficients obtained from REFPROP[10] (α_P^{REFP}), Joule Thomson coefficients computed from MC simulations ($\mu_{\mathrm{JT}}^{\mathrm{MC}}$), and Joule Thomson coefficients obtained from REFPROP[10] ($\mu_{\mathrm{JT}}^{\mathrm{REFP}}$) of CO_2-H_2 binary mixture with 5 mole% impurity of H_2 at 273 K and pressures ranging from 20 bar to 200 bar.

P/ [bar]	ρ^{MC}/ [kg/m^3]	ρ^{MD}/ [kg/m^3]	ρ^{REFP}/ [kg/m^3]	β_T^{MC}/ [10^{-5}/bar]	β_T^{REFP}/ [10^{-5}/bar]	α_P^{MC}/ [10^{-4}/K]	α_P^{REFP}/ [10^{-4}/K]	$\mu_{\mathrm{JT}}^{\mathrm{MC}}$/ [$10^{-3}$ K/bar]	$\mu_{\mathrm{JT}}^{\mathrm{REFP}}$/ [$10^{-3}$ K/bar]
20	41.8	45.1 ± 0.1	42.6	5785.1 ± 53.0	5873.9	56.0 ± 0.6	58.4	1170.1 ± 33.8	1268.9
40	102.7 ± 0.2	118.8 ± 1.1	172.1	4027.4 ± 85.3	8600.2	117.7 ± 3.3	536.3	1213.7 ± 53.1	–
60	841.0 ± 3.6	926.4 ± 1.2	580.0	137.0 ± 8.8	−599.5	88.9 ± 5.4	27.9	59.3 ± 6.5	–
80	863.2 ± 3.2	939.7 ± 1.2	784.2	107.0 ± 9.3	313.3	74.6 ± 4.2	155.9	45.8 ± 5.2	–
100	881.9 ± 1.5	878.0 ± 0.9	869.4	87.0 ± 4.0	91.5	66.5 ± 2.5	64.4	36.5 ± 3.2	36.9
120	895.4 ± 2.9	894.4 ± 1.0	884.2	79.1 ± 2.8	77.5	62.9 ± 2.0	57.6	32.3 ± 2.5	28.7
140	908.0 ± 2.3	904.9 ± 0.7	897.0	65.8 ± 1.3	67.5	55.2 ± 0.8	52.5	23.8 ± 1.1	22.2
160	918.9 ± 0.5	918.0 ± 0.7	908.5	59.2 ± 1.7	60.0	51.8 ± 1.5	48.5	19.6 ± 2.0	16.9
180	929.2 ± 2.0	927.9 ± 0.9	918.9	52.4 ± 2.3	54.1	47.2 ± 1.9	45.4	14.1 ± 2.6	12.6
200	939.9 ± 1.3	938.1 ± 0.5	928.4	47.9 ± 0.5	49.3	44.5 ± 0.9	42.7	10.6 ± 1.2	8.9

Table S132: Heat capacities at constant volume computed from MC simulations (c_V^{MC}), heat capacities at constant volume obtained from REFPROP[10] (c_V^{REFP}), heat capacities at constant pressure computed from MC simulations (c_P^{MC}), heat capacities at constant pressure obtained from REFPROP[10] (c_P^{REFP}), speed of sound computed from MC simulations (c^{MC}), speed of sound obtained from REFPROP[10] (c^{REFP}), viscosities computed from MD simulations (η^{MD}), and viscosities obtained from REFPROP[10] (η^{REFP}) of CO_2-H_2 binary mixture with 5 mole% impurity of H_2 at 273 K and pressures ranging from 20 bar to 200 bar.

P/ [bar]	c_V^{MC}/ [J/mol K]	c_V^{REFP}/ [J/mol K]	c_P^{MC}/ [J/mol K]	c_P^{REFP}/ [J/mol K]	c^{MC}/ [m/s]	c^{REFP}/ [m/s]	η^{MD}/ [μPa s]	η^{REFP}/ [μPa s]
20	30.4	30.5	45.2 ± 0.1	46.0	247.9 ± 1.2	245.6	7.1 ± 4.0	14.0
40	36.0 ± 0.1	–	74.4 ± 1.2	–	223.5 ± 3.0	–	14.6 ± 1.0	16.2
60	40.4 ± 0.3	–	120.1 ± 4.9	–	508.1 ± 19.5	–	106.5 ± 3.2	42.0
80	40.3 ± 0.1	–	110.0 ± 2.3	–	543.5 ± 24.3	–	110.8 ± 2.6	69.9
100	39.5 ± 0.2	39.3	106.2 ± 2.6	99.0	591.8 ± 15.5	562.4	98.1 ± 9.5	88.8
120	39.6 ± 0.1	39.2	104.2 ± 2.3	94.5	609.4 ± 12.8	593.2	99.7 ± 2.9	92.7
140	39.7 ± 0.1	39.1	98.3 ± 1.1	91.2	643.5 ± 7.6	620.5	105.8 ± 7.1	96.3
160	39.6 ± 0.2	39.1	96.2 ± 1.7	88.6	668.5 ± 11.3	645.1	110.1 ± 3.7	99.6
180	39.6 ± 0.1	39.0	92.2 ± 2.3	86.4	692.0 ± 17.3	667.6	114.5 ± 10.3	102.8
200	39.6 ± 0.2	39.0	90.2 ± 1.7	84.7	711.9 ± 7.8	688.4	109.7 ± 2.4	105.8

Table S133: Densities computed from MC and MD simulations (ρ^{MC} and ρ^{MD}), densities obtained from REFPROP[10] (ρ^{REFP}), isothermal compressibilities computed from MC simulations (β_T^{MC}), isothermal compressibilities obtained from REFPROP[10] (β_T^{REFP}), thermal expansion coefficients computed from MC simulations (α_P^{MC}), thermal expansion coefficients obtained from REFPROP[10] (α_P^{REFP}), Joule Thomson coefficients computed from MC simulations (μ_{JT}^{MC}), and Joule Thomson coefficients obtained from REFPROP[10] (μ_{JT}^{REFP}) of CO_2-H_2 binary mixture with 5 mole% impurity of H_2 at 293 K and pressures ranging from 20 bar to 200 bar.

P/ [bar]	ρ^{MC}/ [kg/m^3]	ρ^{MD}/ [kg/m^3]	ρ^{REFP}/ [kg/m^3]	β_T^{MC}/ [10^{-5}/bar]	β_T^{REFP}/ [10^{-5}/bar]	α_P^{MC}/ [10^{-4}/K]	α_P^{REFP}/ [10^{-4}/K]	μ_{JT}^{MC}/ [10^{-3} K/bar]	μ_{JT}^{REFP}/ [10^{-3} K/bar]
20	37.8	40.5 ± 0.1	38.4	5558.3 ± 22.4	5620.1	47.1 ± 0.3	48.6	971.1 ± 19.0	1054.3
40	85.9 ± 0.1	94.4 ± 0.4	89.0	3317.3 ± 31.2	3404.9	73.5 ± 0.6	78.6	997.2 ± 16.0	1049.0
60	158.4 ± 0.4	376.2 ± 131.6	171.9	3164.9 ± 103.9	3558.2	154.2 ± 6.5	190.4	964.1 ± 61.4	993.9
80	595.8 ± 12.7	803.8 ± 1.7	544.0	3701.8 ± 808.2	11 821.4	869.5 ± 167.0	2534.1	349.1 ± 89.3	–
100	717.8 ± 1.6	717.1 ± 1.9	725.2	419.1 ± 27.5	321.3	174.7 ± 9.3	136.2	142.9 ± 11.1	124.8
120	761.8 ± 3.0	757.8 ± 1.7	762.6	217.8 ± 8.4	200.7	109.7 ± 3.3	97.9	93.5 ± 4.5	88.0
140	790.2 ± 2.3	787.3 ± 1.8	789.2	146.2 ± 11.9	147.8	81.5 ± 6.4	79.4	66.1 ± 9.4	66.7
160	813.0 ± 2.4	810.5 ± 1.2	810.2	122.7 ± 7.6	117.7	74.4 ± 3.6	68.2	56.2 ± 5.2	52.2
180	831.1 ± 2.1	829.1 ± 0.8	827.8	99.3 ± 2.4	98.2	64.5 ± 1.5	60.5	44.2 ± 2.2	41.5
200	847.5 ± 1.6	845.3 ± 1.2	843.0	87.6 ± 4.1	84.4	60.0 ± 2.0	54.8	37.7 ± 3.0	33.2

Table S134: Heat capacities at constant volume computed from MC simulations (c_V^{MC}), heat capacities at constant volume obtained from REFPROP[10] (c_V^{REFP}), heat capacities at constant pressure computed from MC simulations (c_P^{MC}), heat capacities at constant pressure obtained from REFPROP[10] (c_P^{REFP}), speed of sound computed from MC simulations (c^{MC}), speed of sound obtained from REFPROP[10] (c^{REFP}), viscosities computed from MD simulations (η^{MD}), and viscosities obtained from REFPROP[10] (η^{REFP}) of CO_2-H_2 binary mixture with 5 mole% impurity of H_2 at 293 K and pressures ranging from 20 bar to 200 bar.

P/ [bar]	c_V^{MC}/ [J/mol K]	c_V^{REFP}/ [J/mol K]	c_P^{MC}/ [J/mol K]	c_P^{REFP}/ [J/mol K]	c^{MC}/ [m/s]	c^{REFP}/ [m/s]	η^{MD}/ [μPa s]	η^{REFP}/ [μPa s]
20	30.4	30.4	43.4 ± 0.1	43.8	260.7 ± 0.6	258.7	7.5 ± 4.2	14.9
40	33.2	33.4	56.5 ± 0.1	58.5	244.4 ± 1.2	240.2	16.3 ± 0.8	15.6
60	38.0 ± 0.4	39.5	96.6 ± 3.2	112.4	225.1 ± 5.4	215.6	31.8 ± 11.8	17.3
80	45.5 ± 1.1	–	494.1 ± 78.0	–	222.2 ± 30.1	–	79.3 ± 3.3	38.5
100	41.1 ± 0.7	40.7	168.3 ± 6.8	138.5	368.8 ± 14.6	382.1	58.3 ± 1.3	60.3
120	40.4 ± 0.3	39.8	130.3 ± 2.5	116.6	440.9 ± 9.7	437.8	67.4 ± 2.6	66.4
140	39.7 ± 0.1	39.3	111.4 ± 5.5	105.7	492.8 ± 23.5	480.2	81.6 ± 12.1	71.2
160	39.5 ± 0.2	39.1	108.4 ± 2.9	99.0	524.5 ± 17.7	515.4	78.1 ± 3.0	75.3
180	39.4 ± 0.3	38.9	101.6 ± 1.5	94.2	558.7 ± 8.2	545.9	80.4 ± 2.7	79.0
200	39.3 ± 0.2	38.8	99.2 ± 1.6	90.7	582.9 ± 14.5	573.1	84.5 ± 4.1	82.3

Table S135: Densities computed from MC and MD simulations (ρ^{MC} and ρ^{MD}), densities obtained from REFPROP[10] (ρ^{REFP}), isothermal compressibilities computed from MC simulations (β_T^{MC}), isothermal compressibilities obtained from REFPROP[10] (β_T^{REFP}), thermal expansion coefficients computed from MC simulations (α_P^{MC}), thermal expansion coefficients obtained from REFPROP[10] (α_P^{REFP}), Joule Thomson coefficients computed from MC simulations ($\mu_{\text{JT}}^{\text{MC}}$), and Joule Thomson coefficients obtained from REFPROP[10] ($\mu_{\text{JT}}^{\text{REFP}}$) of CO_2-H_2 binary mixture with 5 mole% impurity of H_2 at 313 K and pressures ranging from 20 bar to 200 bar.

P/ [bar]	ρ^{MC}/ [kg/m^3]	ρ^{MD}/ [kg/m^3]	ρ^{REFP}/ [kg/m^3]	β_T^{MC}/ [10^{-5}/bar]	β_T^{REFP}/ [10^{-5}/bar]	α_P^{MC}/ [10^{-4}/K]	α_P^{REFP}/ [10^{-4}/K]	$\mu_{\text{JT}}^{\text{MC}}$/ [$10^{-3}$ K/bar]	$\mu_{\text{JT}}^{\text{REFP}}$/ [$10^{-3}$ K/bar]
20	34.6	37.0 ± 0.1	35.0	5417.1 ± 17.8	5463.2	41.2 ± 0.2	42.1	822.6 ± 16.5	891.2
40	75.7	82.2 ± 0.2	77.8	3007.3 ± 16.2	3085.0	55.7 ± 0.3	58.9	817.2 ± 9.4	881.0
60	127.5 ± 0.1	143.1 ± 0.4	133.6	2317.6 ± 34.7	2464.6	81.0 ± 1.4	90.9	790.1 ± 23.2	850.8
80	200.3 ± 0.6	259.0 ± 4.7	217.2	2238.3 ± 24.9	2498.1	140.6 ± 1.8	171.3	740.6 ± 14.2	767.2
100	325.7 ± 2.6	337.6 ± 3.9	376.4	2641.1 ± 138.7	2854.0	319.9 ± 21.5	396.2	579.3 ± 56.5	540.5
120	503.5 ± 4.3	505.7 ± 4.9	552.4	1491.8 ± 103.4	1048.3	322.5 ± 22.2	256.9	333.6 ± 31.5	279.2
140	611.9 ± 2.7	607.5 ± 3.7	634.6	601.9 ± 39.7	466.6	186.5 ± 11.6	150.2	199.1 ± 17.3	174.2
160	669.2 ± 2.5	666.2 ± 1.4	682.3	337.9 ± 36.5	285.6	126.7 ± 11.4	108.3	138.5 ± 18.4	123.9
180	704.7 ± 1.6	706.0 ± 0.9	715.9	239.3 ± 13.0	204.4	101.1 ± 4.4	87.0	107.5 ± 7.3	94.3
200	736.9 ± 3.1	734.5 ± 2.1	742.1	176.6 ± 11.2	158.8	83.4 ± 3.1	73.9	83.1 ± 5.2	74.4

Table S136: Heat capacities at constant volume computed from MC simulations (c_V^{MC}), heat capacities at constant volume obtained from REFPROP[10] (c_V^{REFP}), heat capacities at constant pressure computed from MC simulations (c_P^{MC}), heat capacities at constant pressure obtained from REFPROP[10] (c_P^{REFP}), speed of sound computed from MC simulations (c^{MC}), speed of sound obtained from REFPROP[10] (c^{REFP}), viscosities computed from MD simulations (η^{MD}), and viscosities obtained from REFPROP[10] (η^{REFP}) of CO_2-H_2 binary mixture with 5 mole% impurity of H_2 at 313 K and pressures ranging from 20 bar to 200 bar.

P/ [bar]	c_V^{MC}/ [J/mol K]	c_V^{REFP}/ [J/mol K]	c_P^{MC}/ [J/mol K]	c_P^{REFP}/ [J/mol K]	c^{MC}/ [m/s]	c^{REFP}/ [m/s]	η^{MD}/ [μPa s]	η^{REFP}/ [μPa s]
20	30.8	30.7	42.6 ± 0.1	42.8	272.0 ± 0.5	270.1	16.2 ± 1.3	15.9
40	32.5	32.6	50.3 ± 0.1	51.6	260.8 ± 0.8	256.8	16.1 ± 1.6	16.5
60	34.7 ± 0.1	35.1	64.0 ± 0.5	68.0	249.8 ± 2.1	242.6	17.0 ± 1.4	17.5
80	37.8 ± 0.3	38.7	96.1 ± 0.8	109.7	238.1 ± 1.9	228.4	22.1 ± 3.1	19.7
100	41.6 ± 0.5	43.1	200.2 ± 12.4	234.8	236.5 ± 9.8	225.1	23.7 ± 2.1	26.9
120	42.1 ± 1.0	41.8	227.0 ± 12.4	191.3	268.1 ± 12.3	281.0	34.7 ± 2.2	40.2
140	40.7 ± 0.6	40.3	166.4 ± 7.3	140.2	333.3 ± 13.5	342.7	50.0 ± 12.7	48.9
160	40.0 ± 0.1	39.5	134.2 ± 7.6	118.4	385.5 ± 23.6	392.0	52.4 ± 2.2	54.9
180	39.5 ± 0.2	39.1	119.7 ± 2.8	106.9	423.7 ± 12.6	432.1	60.3 ± 2.3	59.6
200	39.4 ± 0.2	38.9	110.1 ± 1.9	99.6	463.7 ± 15.3	466.3	64.2 ± 2.9	63.6

S15.9 Thermodynamic and Transport Properties Data of binary CO_2 mixture with 10 mole% Impurity of H_2

Table S137: Densities computed from MC and MD simulations (ρ^{MC} and ρ^{MD}), densities obtained from REFPROP[10] (ρ^{REFP}), isothermal compressibilities computed from MC simulations (β_T^{MC}), isothermal compressibilities obtained from REFPROP[10] (β_T^{REFP}), thermal expansion coefficients computed from MC simulations (α_P^{MC}), thermal expansion coefficients obtained from REFPROP[10] (α_P^{REFP}), Joule Thomson coefficients computed from MC simulations ($\mu_{\mathrm{JT}}^{\mathrm{MC}}$), and Joule Thomson coefficients obtained from REFPROP[10] ($\mu_{\mathrm{JT}}^{\mathrm{REFP}}$) of CO_2-H_2 binary mixture with 10 mole% impurity of H_2 at 253 K and pressures ranging from 20 bar to 200 bar.

P/ [bar]	ρ^{MC}/ [kg/m^3]	ρ^{MD}/ [kg/m^3]	ρ^{REFP}/ [kg/m^3]	β_T^{MC}/ [10^{-5}/bar]	β_T^{REFP}/ [10^{-5}/bar]	α_P^{MC}/ [10^{-4}/K]	α_P^{REFP}/ [10^{-4}/K]	$\mu_{\mathrm{JT}}^{\mathrm{MC}}$/ [$10^{-3}$ K/bar]	$\mu_{\mathrm{JT}}^{\mathrm{REFP}}$/ [$10^{-3}$ K/bar]
20	44.0	51.5 ± 0.1	45.0	5952.8 ± 61.9	6104.9	66.0 ± 0.7	69.1	1291.0 ± 36.2	1396.1
40	618.9 ± 290.1	1020.2 ± 0.9	302.9	1763.9 ± 2171.2	−958.8	138.9 ± 51.6	−438.3	285.7 ± 291.2	–
60	861.6 ± 4.3	1026.6 ± 0.9	489.4	109.8 ± 9.5	488.2	78.3 ± 4.5	−481.0	41.8 ± 5.0	–
80	882.2 ± 1.2	1034.1 ± 0.7	612.9	83.3 ± 7.3	−263.4	66.0 ± 5.4	7.3	29.8 ± 6.3	–
100	892.6 ± 2.6	890.6 ± 2.0	698.9	68.3 ± 1.5	−2019.6	56.8 ± 1.3	−684.4	20.7 ± 1.6	–
120	905.2 ± 1.5	902.2 ± 0.7	760.8	64.0 ± 3.1	390.4	56.5 ± 2.7	196.2	19.5 ± 3.1	–
140	915.9 ± 2.3	914.1 ± 0.8	806.7	57.5 ± 1.8	154.2	52.6 ± 1.6	96.9	15.4 ± 1.9	–
160	926.2 ± 1.8	925.0 ± 1.1	841.6	49.4 ± 2.0	93.4	47.1 ± 1.7	68.0	9.2 ± 2.1	–
180	935.0 ± 0.3	933.9 ± 0.7	868.5	45.7 ± 2.5	67.3	44.5 ± 2.1	54.3	6.1 ± 2.6	–
200	943.3 ± 1.2	942.6 ± 0.9	889.7	43.0 ± 1.1	53.3	43.8 ± 1.0	46.5	5.1 ± 1.3	–

Table S138: Heat capacities at constant volume computed from MC simulations (c_V^{MC}), heat capacities at constant volume obtained from REFPROP[10] (c_V^{REFP}), heat capacities at constant pressure computed from MC simulations (c_P^{MC}), heat capacities at constant pressure obtained from REFPROP[10] (c_P^{REFP}), speed of sound computed from MC simulations (c^{MC}), speed of sound obtained from REFPROP[10] (c^{REFP}), viscosities computed from MD simulations (η^{MD}), and viscosities obtained from REFPROP[10] (η^{REFP}) of CO_2-H_2 binary mixture with 10 mole% impurity of H_2 at 253 K and pressures ranging from 20 bar to 200 bar.

P/ [bar]	c_V^{MC}/ [J/mol K]	c_V^{REFP}/ [J/mol K]	c_P^{MC}/ [J/mol K]	c_P^{REFP}/ [J/mol K]	c^{MC}/ [m/s]	c^{REFP}/ [m/s]	η^{MD}/ [μPa s]	η^{REFP}/ [μPa s]
20	30.2	29.9	46.9 ± 0.2	47.4	243.8 ± 1.4	240.2	10.1 ± 2.2	13.0
40	82.9 ± 10.0	–	122.2 ± 2.7	–	170.7 ± 129.4	–	153.6 ± 9.6	20.3
60	42.3 ± 0.6	–	108.4 ± 2.9	–	520.5 ± 24.0	–	150.0 ± 9.0	35.0
80	40.4 ± 0.7	–	101.4 ± 5.0	–	584.2 ± 29.7	–	165.4 ± 26.5	45.9
100	40.5 ± 0.2	–	94.5 ± 1.4	–	618.6 ± 8.4	–	100.3 ± 2.7	56.5
120	40.7 ± 0.4	–	96.6 ± 3.0	–	640.2 ± 18.6	–	117.6 ± 15.6	67.1
140	40.1 ± 0.5	–	93.5 ± 1.7	–	665.7 ± 12.8	–	110.6 ± 6.9	76.9
160	40.5 ± 0.2	–	89.6 ± 1.8	–	695.5 ± 15.6	–	114.9 ± 5.9	85.6
180	40.1 ± 0.3	–	87.2 ± 2.4	–	713.5 ± 21.7	–	125.9 ± 30.7	93.2
200	40.2 ± 0.3	–	87.8 ± 1.3	–	734.4 ± 11.4	–	118.5 ± 3.1	99.8

Table S139: Densities computed from MC and MD simulations (ρ^{MC} and ρ^{MD}), densities obtained from REFPROP[10] (ρ^{REFP}), isothermal compressibilities computed from MC simulations (β_T^{MC}), isothermal compressibilities obtained from REFPROP[10] (β_T^{REFP}), thermal expansion coefficients computed from MC simulations (α_P^{MC}), thermal expansion coefficients obtained from REFPROP[10] (α_P^{REFP}), Joule Thomson coefficients computed from MC simulations ($\mu_{\mathrm{JT}}^{\mathrm{MC}}$), and Joule Thomson coefficients obtained from REFPROP[10] ($\mu_{\mathrm{JT}}^{\mathrm{REFP}}$) of CO_2-H_2 binary mixture with 10 mole% impurity of H_2 at 273 K and pressures ranging from 20 bar to 200 bar.

P/ [bar]	ρ^{MC}/ [kg/m^3]	ρ^{MD}/ [kg/m^3]	ρ^{REFP}/ [kg/m^3]	β_T^{MC}/ [10^{-5}/bar]	β_T^{REFP}/ [10^{-5}/bar]	α_P^{MC}/ [10^{-4}/K]	α_P^{REFP}/ [10^{-4}/K]	$\mu_{\mathrm{JT}}^{\mathrm{MC}}$/ [$10^{-3}$ K/bar]	$\mu_{\mathrm{JT}}^{\mathrm{REFP}}$/ [$10^{-3}$ K/bar]
20	39.1	45.1 ± 0.1	39.8	5593.7 ± 27.9	5742.2	52.5 ± 0.3	55.0	1015.5 ± 20.7	1143.0
40	91.8 ± 0.1	118.8 ± 1.1	96.1	3549.6 ± 79.9	3741.1	92.9 ± 2.1	102.3	1061.5 ± 42.3	1138.4
60	197.0 ± 1.5	926.4 ± 1.2	341.7	6163.5 ± 871.7	−10 701.0	483.5 ± 82.9	−1649.4	939.4 ± 226.5	–
80	711.0 ± 9.3	939.7 ± 1.2	513.9	450.8 ± 92.3	−4612.9	191.3 ± 31.7	−750.3	139.0 ± 32.1	–
100	755.1 ± 5.4	752.1 ± 3.6	630.1	254.7 ± 43.2	2015.0	126.4 ± 15.4	623.0	96.1 ± 17.6	–
120	786.0 ± 3.5	783.4 ± 2.6	710.5	164.2 ± 13.9	333.7	91.6 ± 3.9	148.1	66.5 ± 4.9	–
140	810.2 ± 3.1	804.7 ± 1.8	767.5	123.8 ± 6.5	149.4	78.5 ± 2.0	83.6	52.1 ± 2.6	–
160	826.5 ± 3.2	824.0 ± 1.6	808.7	101.9 ± 7.3	92.2	68.3 ± 4.2	60.3	41.4 ± 5.7	–
180	841.3 ± 2.0	839.6 ± 1.5	827.3	88.8 ± 1.8	75.5	62.6 ± 1.7	52.9	34.3 ± 2.4	24.8
200	857.9 ± 2.2	853.4 ± 1.1	839.1	71.5 ± 2.6	66.9	54.2 ± 1.5	48.9	24.3 ± 2.1	18.9

Table S140: Heat capacities at constant volume computed from MC simulations (c_V^{MC}), heat capacities at constant volume obtained from REFPROP[10] (c_V^{REFP}), heat capacities at constant pressure computed from MC simulations (c_P^{MC}), heat capacities at constant pressure obtained from REFPROP[10] (c_P^{REFP}), speed of sound computed from MC simulations (c^{MC}), speed of sound obtained from REFPROP[10] (c^{REFP}), viscosities computed from MD simulations (η^{MD}), and viscosities obtained from REFPROP[10] (η^{REFP}) of CO_2-H_2 binary mixture with 10 mole% impurity of H_2 at 273 K and pressures ranging from 20 bar to 200 bar.

P/ [bar]	c_V^{MC}/ [J/mol K]	c_V^{REFP}/ [J/mol K]	c_P^{MC}/ [J/mol K]	c_P^{REFP}/ [J/mol K]	c^{MC}/ [m/s]	c^{REFP}/ [m/s]	η^{MD}/ [μPa s]	η^{REFP}/ [μPa s]
20	29.7	29.4	43.4 ± 0.1	43.8	258.5 ± 0.7	255.2	2.7 ± 3.2	14.0
40	33.9 ± 0.1	33.6	62.7 ± 0.7	65.2	238.2 ± 3.1	232.3	14.6 ± 2.3	14.7
60	51.8 ± 2.5	–	262.6 ± 40.4	–	204.5 ± 21.9	–	103.7 ± 5.5	23.1
80	44.3 ± 1.3	–	170.1 ± 18.1	–	346.2 ± 40.3	–	109.1 ± 1.6	35.6
100	41.9 ± 0.7	–	134.4 ± 8.5	–	408.5 ± 37.2	–	72.3 ± 9.1	47.4
120	41.4 ± 0.7	–	114.2 ± 2.2	–	462.5 ± 20.5	–	73.1 ± 1.6	58.7
140	40.4 ± 0.3	–	107.7 ± 1.1	–	515.2 ± 13.8	–	77.9 ± 2.7	68.7
160	40.0 ± 0.3	–	100.7 ± 3.5	–	546.4 ± 22.0	–	82.2 ± 8.3	77.3
180	40.1 ± 0.7	37.5	97.6 ± 2.2	86.1	571.1 ± 10.0	606.3	91.1 ± 5.5	81.7
200	39.5 ± 0.2	37.4	91.9 ± 1.7	83.7	615.5 ± 12.6	630.9	90.0 ± 2.8	84.6

Table S141: Densities computed from MC and MD simulations (ρ^{MC} and ρ^{MD}), densities obtained from REFPROP[10] (ρ^{REFP}), isothermal compressibilities computed from MC simulations (β_T^{MC}), isothermal compressibilities obtained from REFPROP[10] (β_T^{REFP}), thermal expansion coefficients computed from MC simulations (α_P^{MC}), thermal expansion coefficients obtained from REFPROP[10] (α_P^{REFP}), Joule Thomson coefficients computed from MC simulations (μ_{JT}^{MC}), and Joule Thomson coefficients obtained from REFPROP[10] (μ_{JT}^{REFP}) of CO_2-H_2 binary mixture with 10 mole% impurity of H_2 at 293 K and pressures ranging from 20 bar to 200 bar.

P/ [bar]	ρ^{MC}/ [kg/m^3]	ρ^{MD}/ [kg/m^3]	ρ^{REFP}/ [kg/m^3]	β_T^{MC}/ [10^{-5}/bar]	β_T^{REFP}/ [10^{-5}/bar]	α_P^{MC}/ [10^{-4}/K]	α_P^{REFP}/ [10^{-4}/K]	μ_{JT}^{MC}/ [10^{-3} K/bar]	μ_{JT}^{REFP}/ [10^{-3} K/bar]
20	35.4	40.5 ± 0.1	36.0	5459.5 ± 13.7	5537.3	45.2 ± 0.1	46.6	864.7 ± 7.2	955.8
40	78.7 ± 0.1	94.4 ± 0.4	81.4	3119.5 ± 15.6	3219.3	64.9 ± 0.4	69.2	875.0 ± 13.1	944.9
60	136.8 ± 0.1	376.2 ± 131.6	145.9	2539.9 ± 29.6	2783.1	105.5 ± 1.1	123.0	833.4 ± 13.2	903.3
80	231.7 ± 1.4	801.7 ± 5.1	304.3	2840.7 ± 147.4	4376.9	240.3 ± 13.7	496.2	713.6 ± 56.3	–
100	431.4 ± 4.6	434.9 ± 1.6	489.2	2706.1 ± 157.5	2433.9	487.0 ± 25.9	515.2	426.2 ± 31.3	–
120	587.3 ± 1.2	584.6 ± 2.3	617.6	784.4 ± 68.8	535.9	232.7 ± 17.4	176.6	216.2 ± 22.3	–
140	655.8 ± 6.5	650.2 ± 1.8	675.9	365.3 ± 36.0	266.6	138.0 ± 11.7	109.0	137.0 ± 17.2	112.3
160	696.4 ± 1.9	694.1 ± 1.5	706.8	251.0 ± 11.1	189.0	109.3 ± 4.1	86.5	103.9 ± 6.2	83.9
180	725.6 ± 1.2	725.4 ± 1.3	730.6	175.7 ± 9.3	146.7	86.3 ± 4.9	73.2	77.4 ± 7.9	65.1
200	748.3 ± 1.6	745.5 ± 0.8	750.2	157.0 ± 6.2	120.0	81.0 ± 2.5	64.3	69.3 ± 3.9	51.5

Table S142: Heat capacities at constant volume computed from MC simulations (c_V^{MC}), heat capacities at constant volume obtained from REFPROP[10] (c_V^{REFP}), heat capacities at constant pressure computed from MC simulations (c_P^{MC}), heat capacities at constant pressure obtained from REFPROP[10] (c_P^{REFP}), speed of sound computed from MC simulations (c^{MC}), speed of sound obtained from REFPROP[10] (c^{REFP}), viscosities computed from MD simulations (η^{MD}), and viscosities obtained from REFPROP[10] (η^{REFP}) of CO_2-H_2 binary mixture with 10 mole% impurity of H_2 at 293 K and pressures ranging from 20 bar to 200 bar.

P/ [bar]	c_V^{MC}/ [J/mol K]	c_V^{REFP}/ [J/mol K]	c_P^{MC}/ [J/mol K]	c_P^{REFP}/ [J/mol K]	c^{MC}/ [m/s]	c^{REFP}/ [m/s]	η^{MD}/ [μPa s]	η^{REFP}/ [μPa s]
20	29.8	29.6	42.1	42.3	270.4 ± 0.4	267.9	10.0 ± 5.7	14.9
40	32.1	31.9	52.0 ± 0.2	53.2	257.0 ± 0.8	252.3	18.1 ± 2.4	15.5
60	35.8 ± 0.1	35.2	73.0 ± 0.4	78.6	242.4 ± 1.6	234.6	31.4 ± 12.3	16.8
80	42.4 ± 0.8	–	145.5 ± 6.2	–	228.4 ± 8.0	–	75.9 ± 6.6	22.2
100	46.9 ± 0.6	–	287.5 ± 12.9	–	229.3 ± 8.6	–	28.9 ± 2.0	34.0
120	43.0 ± 0.9	–	182.4 ± 9.9	–	303.4 ± 15.9	–	46.3 ± 3.2	46.7
140	41.1 ± 0.4	38.2	134.9 ± 7.4	115.1	370.3 ± 21.0	408.9	51.7 ± 3.7	54.2
160	40.6 ± 0.4	37.7	121.2 ± 3.1	103.1	413.6 ± 10.7	452.3	56.7 ± 2.1	58.7
180	39.7 ± 0.3	37.5	108.4 ± 4.4	95.8	462.9 ± 15.6	488.5	63.2 ± 5.4	62.5
200	39.3 ± 0.5	37.3	105.5 ± 1.8	90.8	478.2 ± 10.7	520.0	66.8 ± 3.6	65.9

Table S143: Densities computed from MC and MD simulations (ρ^{MC} and ρ^{MD}), densities obtained from REFPROP[10] (ρ^{REFP}), isothermal compressibilities computed from MC simulations (β_T^{MC}), isothermal compressibilities obtained from REFPROP[10] (β_T^{REFP}), thermal expansion coefficients computed from MC simulations (α_P^{MC}), thermal expansion coefficients obtained from REFPROP[10] (α_P^{REFP}), Joule Thomson coefficients computed from MC simulations ($\mu_{\mathrm{JT}}^{\mathrm{MC}}$), and Joule Thomson coefficients obtained from REFPROP[10] ($\mu_{\mathrm{JT}}^{\mathrm{REFP}}$) of CO_2-H_2 binary mixture with 10 mole% impurity of H_2 at 313 K and pressures ranging from 20 bar to 200 bar.

P/ [bar]	ρ^{MC}/ [kg/m^3]	ρ^{MD}/ [kg/m^3]	ρ^{REFP}/ [kg/m^3]	β_T^{MC}/ [10^{-5}/bar]	β_T^{REFP}/ [10^{-5}/bar]	α_P^{MC}/ [10^{-4}/K]	α_P^{REFP}/ [10^{-4}/K]	$\mu_{\mathrm{JT}}^{\mathrm{MC}}$/ [$10^{-3}$ K/bar]	$\mu_{\mathrm{JT}}^{\mathrm{REFP}}$/ [$10^{-3}$ K/bar]
20	32.5	37.0 ± 0.1	33.0	5364.2 ± 22.1	5405.6	40.1 ± 0.2	40.9	746.3 ± 16.0	811.7
40	70.2	82.2 ± 0.2	72.1	2917.4 ± 28.2	2987.7	51.9 ± 0.6	54.4	737.4 ± 21.7	798.9
60	115.3 ± 0.2	143.1 ± 0.4	120.6	2165.4 ± 24.3	2274.9	70.0 ± 0.8	76.6	709.7 ± 15.5	770.3
80	172.1 ± 0.3	259.0 ± 4.7	184.6	1873.8 ± 81.7	2036.9	100.9 ± 4.8	117.1	664.5 ± 50.2	710.2
100	247.7 ± 1.0	254.1 ± 1.2	275.9	1738.5 ± 45.5	1984.8	149.7 ± 4.5	191.7	571.5 ± 25.8	593.5
120	346.4 ± 2.1	350.4 ± 1.4	397.3	1538.2 ± 13.1	1533.1	208.2 ± 3.8	242.0	442.3 ± 12.8	–
140	451.6 ± 3.0	449.3 ± 2.3	500.2	1035.6 ± 64.6	824.8	202.5 ± 12.5	186.1	311.4 ± 26.1	267.9
160	528.9 ± 2.6	526.5 ± 2.6	567.0	626.8 ± 18.1	475.6	159.3 ± 7.6	135.2	218.8 ± 15.7	185.1
180	585.9 ± 5.1	583.4 ± 3.1	612.4	435.2 ± 29.8	313.5	133.4 ± 6.6	104.6	168.9 ± 12.2	136.0
200	627.8 ± 1.7	623.4 ± 2.3	646.0	287.3 ± 8.5	229.2	102.3 ± 3.6	86.0	124.3 ± 7.1	104.6

Table S144: Heat capacities at constant volume computed from MC simulations (c_V^{MC}), heat capacities at constant volume obtained from REFPROP[10] (c_V^{REFP}), heat capacities at constant pressure computed from MC simulations (c_P^{MC}), heat capacities at constant pressure obtained from REFPROP[10] (c_P^{REFP}), speed of sound computed from MC simulations (c^{MC}), speed of sound obtained from REFPROP[10] (c^{REFP}), viscosities computed from MD simulations (η^{MD}), and viscosities obtained from REFPROP[10] (η^{REFP}) of CO_2-H_2 binary mixture with 10 mole% impurity of H_2 at 313 K and pressures ranging from 20 bar to 200 bar.

P/ [bar]	c_V^{MC}/ [J/mol K]	c_V^{REFP}/ [J/mol K]	c_P^{MC}/ [J/mol K]	c_P^{REFP}/ [J/mol K]	c^{MC}/ [m/s]	c^{REFP}/ [m/s]	η^{MD}/ [μPa s]	η^{REFP}/ [μPa s]
20	30.1	29.9	41.6 ± 0.1	41.6	281.2 ± 0.6	279.3	9.4 ± 5.0	15.9
40	31.6	31.5	48.0 ± 0.2	48.6	272.3 ± 1.4	267.8	16.9 ± 2.1	16.4
60	33.5 ± 0.1	33.3	58.0 ± 0.3	60.0	263.4 ± 1.7	256.3	17.7 ± 4.4	17.3
80	35.8 ± 0.1	35.5	75.2 ± 2.1	80.9	255.0 ± 6.6	246.2	21.0 ± 4.5	18.8
100	38.7 ± 0.2	38.0	103.7 ± 2.4	121.6	249.3 ± 4.5	241.9	25.1 ± 9.6	22.0
120	40.4 ± 0.2	–	143.4 ± 2.6	–	257.9 ± 2.8	–	25.3 ± 1.4	28.3
140	41.4 ± 0.6	38.8	151.1 ± 6.1	143.3	279.5 ± 10.6	299.4	37.1 ± 3.6	35.8
160	40.7 ± 0.4	38.1	137.1 ± 5.5	122.6	318.8 ± 8.0	345.4	42.3 ± 10.4	42.0
180	39.9 ± 0.2	37.7	127.7 ± 3.8	108.6	354.6 ± 13.4	387.5	45.7 ± 2.7	46.9
200	39.5 ± 0.2	37.4	112.3 ± 3.0	99.7	396.8 ± 8.0	424.2	47.2 ± 1.1	51.0

S15.10 Thermodynamic and Transport Properties Data of binary CO_2 mixture with 1 mole% Impurity of CH_4

Table S145: Densities computed from MC and MD simulations (ρ^{MC} and ρ^{MD}), densities obtained from REFPROP[10] (ρ^{REFP}), isothermal compressibilities computed from MC simulations (β_T^{MC}), isothermal compressibilities obtained from REFPROP[10] (β_T^{REFP}), thermal expansion coefficients computed from MC simulations (α_P^{MC}), thermal expansion coefficients obtained from REFPROP[10] (α_P^{REFP}), Joule Thomson coefficients computed from MC simulations ($\mu_{\mathrm{JT}}^{\mathrm{MC}}$), and Joule Thomson coefficients obtained from REFPROP[10] ($\mu_{\mathrm{JT}}^{\mathrm{REFP}}$) of CO_2-CH_4 binary mixture with 1 mole% impurity of CH_4 at 253 K and pressures ranging from 20 bar to 200 bar.

P/ [bar]	ρ^{MC}/ [kg/m^3]	ρ^{MD}/ [kg/m^3]	ρ^{REFP}/ [kg/m^3]	β_T^{MC}/ [10^{-5}/bar]	β_T^{REFP}/ [10^{-5}/bar]	α_P^{MC}/ [10^{-4}/K]	α_P^{REFP}/ [10^{-4}/K]	$\mu_{\mathrm{JT}}^{\mathrm{MC}}$/ [$10^{-3}$ K/bar]	$\mu_{\mathrm{JT}}^{\mathrm{REFP}}$/ [$10^{-3}$ K/bar]
20	50.6	51.9 ± 0.2	87.7	6395.1 ± 70.4	7621.6	77.1 ± 0.8	219.6	1567.1 ± 34.1	–
40	1024.9 ± 1.3	1023.1 ± 1.1	1028.0	37.3 ± 1.8	42.7	43.7 ± 2.3	45.4	4.7 ± 2.6	6.8
60	1032.6 ± 0.8	1030.3 ± 0.8	1036.5	33.4 ± 1.3	39.1	40.3 ± 1.4	42.8	0.9 ± 1.6	3.9
80	1040.0 ± 0.7	1037.5 ± 1.1	1044.3	31.7 ± 0.8	36.2	39.2 ± 1.2	40.7	-0.3 ± 1.4	1.4
100	1045.7 ± 1.3	998.7 ± 0.5	1051.6	29.4 ± 0.8	33.7	37.1 ± 0.9	38.8	-2.9 ± 1.1	−0.8
120	1052.1 ± 1.6	1005.7 ± 0.6	1058.5	28.5 ± 1.3	31.6	36.6 ± 1.4	37.2	-3.4 ± 1.6	−2.8
140	1057.6 ± 0.2	1011.9 ± 0.4	1065.0	27.8 ± 1.5	29.8	36.6 ± 2.1	35.8	-3.3 ± 2.4	−4.5
160	1063.3 ± 1.2	1017.8 ± 0.5	1071.2	26.0 ± 1.0	28.2	34.5 ± 1.4	34.5	-5.9 ± 1.6	−6.1
180	1069.6 ± 0.7	1023.1 ± 0.9	1077.1	24.4 ± 1.2	26.8	32.8 ± 1.7	33.4	-8.0 ± 2.1	−7.5
200	1073.9 ± 0.4	1028.5 ± 0.4	1082.8	23.6 ± 0.2	25.5	32.7 ± 0.3	32.4	-8.0 ± 0.4	−8.8

Table S146: Heat capacities at constant volume computed from MC simulations (c_V^{MC}), heat capacities at constant volume obtained from REFPROP[10] (c_V^{REFP}), heat capacities at constant pressure computed from MC simulations (c_P^{MC}), heat capacities at constant pressure obtained from REFPROP[10] (c_P^{REFP}), speed of sound computed from MC simulations (c^{MC}), speed of sound obtained from REFPROP[10] (c^{REFP}), viscosities computed from MD simulations (η^{MD}), and viscosities obtained from REFPROP[10] (η^{REFP}) of CO_2-CH_4 binary mixture with 1 mole% impurity of CH_4 at 253 K and pressures ranging from 20 bar to 200 bar.

P/ [bar]	c_V^{MC}/ [J/mol K]	c_V^{REFP}/ [J/mol K]	c_P^{MC}/ [J/mol K]	c_P^{REFP}/ [J/mol K]	c^{MC}/ [m/s]	c^{REFP}/ [m/s]	η^{MD}/ [μPa s]	η^{REFP}/ [μPa s]
20	32.1 ± 0.1	–	52.4 ± 0.3	–	224.5 ± 1.4	–	10.4 ± 3.0	13.3
40	40.4 ± 0.2	41.0	95.6 ± 3.1	92.9	786.8 ± 23.1	719.0	147.2 ± 3.1	140.0
60	40.7 ± 0.2	41.0	92.9 ± 1.8	91.0	812.9 ± 18.2	740.0	152.0 ± 2.7	143.9
80	40.6 ± 0.3	41.0	92.4 ± 1.9	89.4	830.0 ± 14.1	759.4	154.4 ± 4.9	147.7
100	40.7 ± 0.3	41.0	90.2 ± 1.3	88.0	849.3 ± 13.5	777.6	149.6 ± 7.8	151.4
120	40.8 ± 0.1	41.0	90.2 ± 1.7	86.8	858.4 ± 20.9	794.7	138.1 ± 7.0	154.9
140	40.9 ± 0.1	41.1	91.4 ± 3.2	85.7	871.1 ± 28.0	810.9	157.3 ± 11.4	158.3
160	40.8 ± 0.2	41.1	88.4 ± 2.1	84.8	884.5 ± 20.3	826.3	155.4 ± 8.3	161.7
180	40.8 ± 0.2	41.1	86.6 ± 2.6	84.0	901.8 ± 25.9	841.0	179.3 ± 33.7	165.0
200	41.2 ± 0.3	41.2	87.7 ± 0.8	83.2	916.0 ± 7.1	855.0	167.7 ± 12.6	168.3

Table S147: Densities computed from MC and MD simulations (ρ^{MC} and ρ^{MD}), densities obtained from REFPROP[10] (ρ^{REFP}), isothermal compressibilities computed from MC simulations (β_T^{MC}), isothermal compressibilities obtained from REFPROP[10] (β_T^{REFP}), thermal expansion coefficients computed from MC simulations (α_P^{MC}), thermal expansion coefficients obtained from REFPROP[10] (α_P^{REFP}), Joule Thomson coefficients computed from MC simulations (μ_{JT}^{MC}), and Joule Thomson coefficients obtained from REFPROP[10] (μ_{JT}^{REFP}) of CO_2-CH_4 binary mixture with 1 mole% impurity of CH_4 at 273 K and pressures ranging from 20 bar to 200 bar.

P/ [bar]	ρ^{MC}/ [kg/m^3]	ρ^{MD}/ [kg/m^3]	ρ^{REFP}/ [kg/m^3]	β_T^{MC}/ [10^{-5}/bar]	β_T^{REFP}/ [10^{-5}/bar]	α_P^{MC}/ [10^{-4}/K]	α_P^{REFP}/ [10^{-4}/K]	μ_{JT}^{MC}/ [10^{-3} K/bar]	μ_{JT}^{REFP}/ [10^{-3} K/bar]
20	44.4	45.3 ± 0.1	45.3	5903.8 ± 28.1	6012.2	58.8 ± 0.3	62.1	1273.2 ± 19.8	1387.0
40	924.0 ± 1.8	917.8 ± 2.0	917.7	82.2 ± 7.1	102.3	68.6 ± 5.1	74.3	36.1 ± 6.0	44.0
60	936.2 ± 0.8	933.4 ± 2.5	934.7	68.2 ± 4.1	83.0	59.5 ± 3.6	64.2	27.4 ± 4.4	33.7
80	948.2 ± 1.4	946.1 ± 1.2	949.1	59.2 ± 1.5	70.4	53.3 ± 1.1	57.3	20.9 ± 1.4	26.2
100	960.6 ± 1.4	909.2 ± 1.9	961.6	51.8 ± 3.5	61.4	48.7 ± 2.7	52.3	15.4 ± 3.5	20.3
120	969.1 ± 0.9	920.2 ± 1.3	972.8	47.2 ± 1.5	54.7	45.6 ± 1.3	48.4	11.7 ± 1.7	15.5
140	978.5 ± 1.3	930.8 ± 0.7	983.0	42.6 ± 0.6	49.4	42.7 ± 0.8	45.3	8.0 ± 1.0	11.5
160	986.4 ± 0.8	940.0 ± 0.8	992.3	41.6 ± 2.5	45.2	43.0 ± 2.5	42.7	8.2 ± 3.3	8.2
180	995.4 ± 0.6	948.0 ± 1.0	1001.0	37.6 ± 1.0	41.6	40.1 ± 1.0	40.5	4.5 ± 1.3	5.2
200	1001.6 ± 0.3	956.3 ± 1.1	1009.0	35.7 ± 1.4	38.7	38.6 ± 1.5	38.6	2.6 ± 2.0	2.7

Table S148: Heat capacities at constant volume computed from MC simulations (c_V^{MC}), heat capacities at constant volume obtained from REFPROP[10] (c_V^{REFP}), heat capacities at constant pressure computed from MC simulations (c_P^{MC}), heat capacities at constant pressure obtained from REFPROP[10] (c_P^{REFP}), speed of sound computed from MC simulations (c^{MC}), speed of sound obtained from REFPROP[10] (c^{REFP}), viscosities computed from MD simulations (η^{MD}), and viscosities obtained from REFPROP[10] (η^{REFP}) of CO_2-CH_4 binary mixture with 1 mole% impurity of CH_4 at 273 K and pressures ranging from 20 bar to 200 bar.

P/ [bar]	c_V^{MC}/ [J/mol K]	c_V^{REFP}/ [J/mol K]	c_P^{MC}/ [J/mol K]	c_P^{REFP}/ [J/mol K]	c^{MC}/ [m/s]	c^{REFP}/ [m/s]	η^{MD}/ [μPa s]	η^{REFP}/ [μPa s]
20	31.1	31.6	46.8 ± 0.1	48.5	239.7 ± 0.6	237.6	4.9 ± 5.2	13.9
40	39.9 ± 0.2	41.1	114.6 ± 5.2	111.2	615.0 ± 30.0	536.7	104.8 ± 1.9	98.7
60	40.0 ± 0.3	40.8	106.6 ± 3.9	104.2	646.5 ± 23.0	573.7	109.1 ± 3.4	103.6
80	39.9 ± 0.2	40.7	100.6 ± 1.3	99.4	670.2 ± 9.5	605.0	115.2 ± 2.5	108.0
100	40.0 ± 0.1	40.6	97.0 ± 2.7	95.9	698.5 ± 25.4	632.5	106.3 ± 5.2	112.1
120	39.9 ± 0.2	40.5	94.4 ± 1.5	93.1	718.9 ± 12.9	657.1	111.9 ± 5.6	115.9
140	40.1 ± 0.1	40.5	92.2 ± 1.2	90.9	742.6 ± 7.1	679.5	122.9 ± 11.8	119.5
160	40.1 ± 0.2	40.5	94.0 ± 3.2	89.0	755.8 ± 26.5	700.3	114.7 ± 5.1	122.9
180	40.2 ± 0.2	40.5	91.4 ± 1.1	87.4	780.0 ± 11.5	719.6	127.9 ± 9.8	126.2
200	40.4	40.5	90.0 ± 1.9	86.1	789.5 ± 17.5	737.7	124.0 ± 9.1	129.4

Table S149: Densities computed from MC and MD simulations (ρ^{MC} and ρ^{MD}), densities obtained from REFPROP[10] (ρ^{REFP}), isothermal compressibilities computed from MC simulations (β_T^{MC}), isothermal compressibilities obtained from REFPROP[10] (β_T^{REFP}), thermal expansion coefficients computed from MC simulations (α_P^{MC}), thermal expansion coefficients obtained from REFPROP[10] (α_P^{REFP}), Joule Thomson coefficients computed from MC simulations ($\mu_{\mathrm{JT}}^{\mathrm{MC}}$), and Joule Thomson coefficients obtained from REFPROP[10] ($\mu_{\mathrm{JT}}^{\mathrm{REFP}}$) of CO_2-CH_4 binary mixture with 1 mole% impurity of CH_4 at 293 K and pressures ranging from 20 bar to 200 bar.

P/ [bar]	ρ^{MC}/ [kg/m^3]	ρ^{MD}/ [kg/m^3]	ρ^{REFP}/ [kg/m^3]	β_T^{MC}/ [10^{-5}/bar]	β_T^{REFP}/ [10^{-5}/bar]	α_P^{MC}/ [10^{-4}/K]	α_P^{REFP}/ [10^{-4}/K]	$\mu_{\mathrm{JT}}^{\mathrm{MC}}$/ [$10^{-3}$ K/bar]	$\mu_{\mathrm{JT}}^{\mathrm{REFP}}$/ [$10^{-3}$ K/bar]
20	39.9	40.7 ± 0.1	40.5	5611.5 ± 28.4	5701.8	48.6 ± 0.2	50.5	1041.6 ± 16.6	1144.5
40	93.1 ± 0.1	95.1 ± 0.3	96.5	3460.4 ± 32.1	3632.8	81.4 ± 0.6	90.5	1074.2 ± 13.5	1146.2
60	784.0 ± 5.5	728.5 ± 72.3	758.9	297.4 ± 23.4	520.1	149.3 ± 10.2	213.4	114.0 ± 11.5	157.7
80	820.2 ± 1.6	813.1 ± 1.7	810.8	172.6 ± 12.7	229.9	99.9 ± 6.4	116.1	78.3 ± 8.2	96.3
100	845.5 ± 1.3	784.1 ± 2.2	841.7	125.8 ± 7.8	154.9	81.3 ± 4.4	87.9	59.5 ± 5.9	69.9
120	865.0 ± 2.6	810.3 ± 1.1	864.7	103.0 ± 7.0	118.7	70.5 ± 4.3	73.2	48.2 ± 6.0	53.8
140	880.5 ± 1.2	830.5 ± 1.6	883.4	85.2 ± 6.7	96.9	61.6 ± 4.2	63.8	38.1 ± 6.0	42.5
160	896.5 ± 1.7	846.6 ± 1.3	899.3	72.9 ± 6.5	82.3	55.7 ± 4.7	57.3	30.6 ± 6.8	33.9
180	907.7 ± 0.8	859.8 ± 1.0	913.3	64.5 ± 1.4	71.7	51.4 ± 1.0	52.3	25.0 ± 1.5	27.2
200	917.4 ± 1.2	872.8 ± 1.1	925.7	58.6 ± 3.2	63.7	47.9 ± 2.3	48.4	20.3 ± 3.5	21.7

Table S150: Heat capacities at constant volume computed from MC simulations (c_V^{MC}), heat capacities at constant volume obtained from REFPROP[10] (c_V^{REFP}), heat capacities at constant pressure computed from MC simulations (c_P^{MC}), heat capacities at constant pressure obtained from REFPROP[10] (c_P^{REFP}), speed of sound computed from MC simulations (c^{MC}), speed of sound obtained from REFPROP[10] (c^{REFP}), viscosities computed from MD simulations (η^{MD}), and viscosities obtained from REFPROP[10] (η^{REFP}) of CO_2-CH_4 binary mixture with 1 mole% impurity of CH_4 at 293 K and pressures ranging from 20 bar to 200 bar.

P/ [bar]	c_V^{MC}/ [J/mol K]	c_V^{REFP}/ [J/mol K]	c_P^{MC}/ [J/mol K]	c_P^{REFP}/ [J/mol K]	c^{MC}/ [m/s]	c^{REFP}/ [m/s]	η^{MD}/ [μPa s]	η^{REFP}/ [μPa s]
20	31.0	31.2	44.5 ± 0.1	45.4	253.3 ± 0.7	251.0	5.1 ± 3.5	14.9
40	34.2	35.4	60.6 ± 0.2	65.3	234.5 ± 1.1	229.5	17.4 ± 1.8	15.6
60	40.3 ± 0.2	44.1	165.1 ± 7.9	191.9	419.0 ± 19.3	331.9	61.1 ± 10.7	64.8
80	40.1 ± 0.1	41.9	131.2 ± 5.3	134.6	481.0 ± 20.3	415.0	76.3 ± 1.4	73.9
100	39.6 ± 0.2	41.1	120.0 ± 4.1	117.0	533.6 ± 19.0	467.2	75.9 ± 6.0	80.1
120	39.7 ± 0.1	40.7	111.8 ± 4.2	107.5	562.1 ± 21.8	507.4	81.7 ± 6.0	85.1
140	39.7 ± 0.1	40.5	105.0 ± 4.0	101.4	593.8 ± 25.8	541.0	85.3 ± 4.3	89.4
160	39.8 ± 0.2	40.3	100.8 ± 4.9	97.1	622.5 ± 31.7	570.2	89.5 ± 7.2	93.4
180	39.6 ± 0.2	40.2	97.7 ± 1.0	93.7	648.7 ± 8.0	596.3	90.7 ± 8.1	97.0
200	39.7 ± 0.2	40.2	94.6 ± 2.7	91.1	666.4 ± 20.5	620.1	106.2 ± 24.0	100.4

Table S151: Densities computed from MC and MD simulations (ρ^{MC} and ρ^{MD}), densities obtained from REFPROP[10] (ρ^{REFP}), isothermal compressibilities computed from MC simulations (β_T^{MC}), isothermal compressibilities obtained from REFPROP[10] (β_T^{REFP}), thermal expansion coefficients computed from MC simulations (α_P^{MC}), thermal expansion coefficients obtained from REFPROP[10] (α_P^{REFP}), Joule Thomson coefficients computed from MC simulations ($\mu_{\mathrm{JT}}^{\mathrm{MC}}$), and Joule Thomson coefficients obtained from REFPROP[10] ($\mu_{\mathrm{JT}}^{\mathrm{REFP}}$) of CO_2-CH_4 binary mixture with 1 mole% impurity of CH_4 at 313 K and pressures ranging from 20 bar to 200 bar.

P/ [bar]	ρ^{MC}/ [kg/m^3]	ρ^{MD}/ [kg/m^3]	ρ^{REFP}/ [kg/m^3]	β_T^{MC}/ [10^{-5}/bar]	β_T^{REFP}/ [10^{-5}/bar]	α_P^{MC}/ [10^{-4}/K]	α_P^{REFP}/ [10^{-4}/K]	$\mu_{\mathrm{JT}}^{\mathrm{MC}}$/ [$10^{-3}$ K/bar]	$\mu_{\mathrm{JT}}^{\mathrm{REFP}}$/ [$10^{-3}$ K/bar]
20	36.4	37.2 ± 0.1	36.9	5498.6 ± 33.0	5518.1	42.5 ± 0.3	43.4	905.8 ± 23.8	962.6
40	81.1 ± 0.1	82.8 ± 0.3	83.1	3100.4 ± 36.3	3188.4	59.8 ± 0.7	63.7	891.0 ± 23.9	956.1
60	140.9 ± 0.2	146.2 ± 0.6	147.5	2605.9 ± 56.6	2719.7	99.8 ± 2.1	110.8	899.4 ± 28.7	923.5
80	246.0 ± 2.3	273.3 ± 4.3	269.4	3434.1 ± 167.3	3760.2	279.5 ± 15.1	335.0	812.5 ± 60.8	–
100	609.1 ± 10.1	467.5 ± 8.1	601.6	1383.8 ± 226.0	1451.9	382.3 ± 51.0	376.4	274.7 ± 49.6	–
120	708.6 ± 4.5	628.4 ± 3.5	700.5	391.3 ± 37.2	442.5	158.4 ± 12.8	159.0	147.7 ± 17.0	161.1
140	751.4 ± 1.7	691.6 ± 3.8	748.7	221.8 ± 16.1	257.3	104.3 ± 6.9	109.2	101.4 ± 10.5	112.1
160	779.5 ± 1.9	725.2 ± 1.0	781.7	171.5 ± 10.8	182.3	88.3 ± 4.4	86.6	82.4 ± 6.8	84.9
180	804.4 ± 2.1	753.5 ± 1.9	807.1	128.0 ± 5.2	141.6	72.4 ± 2.6	73.3	62.9 ± 4.2	66.9
200	822.5 ± 1.5	775.0 ± 0.7	828.0	110.1 ± 6.1	115.9	65.9 ± 3.5	64.3	53.6 ± 5.8	53.9

Table S152: Heat capacities at constant volume computed from MC simulations (c_V^{MC}), heat capacities at constant volume obtained from REFPROP[10] (c_V^{REFP}), heat capacities at constant pressure computed from MC simulations (c_P^{MC}), heat capacities at constant pressure obtained from REFPROP[10] (c_P^{REFP}), speed of sound computed from MC simulations (c^{MC}), speed of sound obtained from REFPROP[10] (c^{REFP}), viscosities computed from MD simulations (η^{MD}), and viscosities obtained from REFPROP[10] (η^{REFP}) of CO_2-CH_4 binary mixture with 1 mole% impurity of CH_4 at 313 K and pressures ranging from 20 bar to 200 bar.

P/ [bar]	c_V^{MC}/ [J/mol K]	c_V^{REFP}/ [J/mol K]	c_P^{MC}/ [J/mol K]	c_P^{REFP}/ [J/mol K]	c^{MC}/ [m/s]	c^{REFP}/ [m/s]	η^{MD}/ [μPa s]	η^{REFP}/ [μPa s]
20	31.3	31.4	43.6 ± 0.1	44.0	263.7 ± 0.8	262.6	12.3 ± 3.9	15.9
40	33.3	33.8	52.7 ± 0.3	54.8	251.0 ± 1.6	247.4	17.3 ± 2.1	16.5
60	36.0 ± 0.1	37.3	73.3 ± 0.7	79.3	235.4 ± 2.8	230.0	19.6 ± 2.0	17.7
80	41.0 ± 0.4	–	169.6 ± 7.2	–	221.4 ± 7.3	–	22.7 ± 3.7	21.6
100	41.8 ± 0.4	–	286.8 ± 30.2	–	285.3 ± 27.9	–	33.5 ± 3.3	44.9
120	40.1 ± 0.2	42.4	165.4 ± 9.1	154.1	385.8 ± 21.2	342.2	51.5 ± 6.0	56.8
140	39.7 ± 0.1	41.3	130.1 ± 5.4	126.0	443.6 ± 18.6	398.0	64.2 ± 10.4	63.8
160	39.4 ± 0.2	40.7	120.1 ± 3.4	112.7	477.5 ± 16.6	440.8	67.6 ± 3.7	69.2
180	39.5 ± 0.1	40.4	109.6 ± 2.2	104.7	519.2 ± 11.9	476.3	66.0 ± 1.4	73.7
200	39.5 ± 0.2	40.2	105.4 ± 3.4	99.2	542.7 ± 17.6	507.1	72.6 ± 4.6	77.6

S15.11 Thermodynamic and Transport Properties Data of binary CO_2 mixture with 5 mole% Impurity of CH_4

Table S153: Densities computed from MC and MD simulations (ρ^{MC} and ρ^{MD}), densities obtained from REFPROP[10] (ρ^{REFP}), isothermal compressibilities computed from MC simulations (β_T^{MC}), isothermal compressibilities obtained from REFPROP[10] (β_T^{REFP}), thermal expansion coefficients computed from MC simulations (α_P^{MC}), thermal expansion coefficients obtained from REFPROP[10] (α_P^{REFP}), Joule Thomson coefficients computed from MC simulations ($\mu_{\mathrm{JT}}^{\mathrm{MC}}$), and Joule Thomson coefficients obtained from REFPROP[10] ($\mu_{\mathrm{JT}}^{\mathrm{REFP}}$) of CO_2-CH_4 binary mixture with 5 mole% impurity of CH_4 at 253 K and pressures ranging from 20 bar to 200 bar.

P/ [bar]	ρ^{MC}/ [kg/m^3]	ρ^{MD}/ [kg/m^3]	ρ^{REFP}/ [kg/m^3]	β_T^{MC}/ [10^{-5}/bar]	β_T^{REFP}/ [10^{-5}/bar]	α_P^{MC}/ [10^{-4}/K]	α_P^{REFP}/ [10^{-4}/K]	$\mu_{\mathrm{JT}}^{\mathrm{MC}}$/ [$10^{-3}$ K/bar]	$\mu_{\mathrm{JT}}^{\mathrm{REFP}}$/ [$10^{-3}$ K/bar]
20	48.9 ± 0.1	51.9 ± 0.2	50.4	6295.0 ± 57.8	6545.9	74.5 ± 0.7	82.7	1509.2 ± 31.2	1661.5
40	977.6 ± 1.7	1023.1 ± 1.1	974.0	43.7 ± 3.1	52.6	47.3 ± 2.9	50.4	8.9 ± 3.3	12.9
60	985.2 ± 1.4	1030.3 ± 0.8	983.8	39.9 ± 1.9	47.2	44.5 ± 2.2	46.9	5.8 ± 2.6	8.9
80	993.0 ± 1.4	1037.5 ± 1.1	992.7	36.8 ± 2.2	43.0	41.9 ± 2.4	44.1	2.9 ± 2.8	5.5
100	1000.7 ± 1.3	998.7 ± 0.5	1000.9	35.2 ± 1.9	39.5	41.1 ± 2.2	41.7	1.8 ± 2.5	2.7
120	1006.3 ± 0.8	1005.7 ± 0.6	1008.5	32.4 ± 1.3	36.7	38.6 ± 1.8	39.7	-1.1 ± 2.2	0.2
140	1013.8 ± 0.8	1011.9 ± 0.4	1015.7	29.4 ± 0.6	34.2	35.9 ± 1.0	37.9	-4.4 ± 1.2	-2.0
160	1019.8 ± 0.8	1017.8 ± 0.5	1022.5	28.9 ± 1.2	32.1	36.2 ± 1.8	36.4	-4.0 ± 2.1	-3.9
180	1024.5 ± 0.9	1023.1 ± 0.9	1028.9	27.2 ± 0.8	30.3	34.4 ± 1.2	35.1	-6.3 ± 1.5	-5.7
200	1030.3 ± 1.3	1028.5 ± 0.4	1034.9	26.1 ± 0.8	28.7	33.8 ± 1.1	33.8	-6.9 ± 1.4	-7.2

Table S154: Heat capacities at constant volume computed from MC simulations (c_V^{MC}), heat capacities at constant volume obtained from REFPROP[10] (c_V^{REFP}), heat capacities at constant pressure computed from MC simulations (c_P^{MC}), heat capacities at constant pressure obtained from REFPROP[10] (c_P^{REFP}), speed of sound computed from MC simulations (c^{MC}), speed of sound obtained from REFPROP[10] (c^{REFP}), viscosities computed from MD simulations (η^{MD}), and viscosities obtained from REFPROP[10] (η^{REFP}) of CO_2-CH_4 binary mixture with 5 mole% impurity of CH_4 at 253 K and pressures ranging from 20 bar to 200 bar.

P/ [bar]	c_V^{MC}/ [J/mol K]	c_V^{REFP}/ [J/mol K]	c_P^{MC}/ [J/mol K]	c_P^{REFP}/ [J/mol K]	c^{MC}/ [m/s]	c^{REFP}/ [m/s]	η^{MD}/ [μPa s]	η^{REFP}/ [μPa s]
20	31.7	33.2	51.1 ± 0.3	55.6	228.8 ± 1.2	225.2	10.4 ± 3.0	12.9
40	40.1 ± 0.1	40.1	96.7 ± 3.2	93.6	751.6 ± 29.3	675.4	147.2 ± 3.1	124.0
60	40.1 ± 0.2	40.0	94.5 ± 3.1	91.1	774.6 ± 22.6	699.9	152.0 ± 2.7	128.0
80	40.2 ± 0.2	40.0	92.1 ± 2.8	89.1	792.3 ± 27.2	722.2	154.4 ± 4.9	131.8
100	40.2 ± 0.2	40.0	91.9 ± 2.9	87.4	805.5 ± 25.0	742.8	149.6 ± 7.8	135.5
120	40.4 ± 0.2	40.0	89.5 ± 2.9	85.9	824.2 ± 21.6	761.9	138.1 ± 7.0	139.0
140	40.5 ± 0.2	40.1	86.8 ± 1.8	84.7	847.4 ± 12.3	779.9	157.3 ± 11.4	142.4
160	40.5 ± 0.1	40.1	88.2 ± 2.8	83.6	858.6 ± 22.1	796.9	155.4 ± 8.3	145.7
180	40.4 ± 0.2	40.1	86.4 ± 1.9	82.6	876.6 ± 16.6	812.9	179.3 ± 33.7	148.9
200	40.7 ± 0.3	40.2	86.3 ± 1.8	81.8	887.9 ± 16.8	828.2	167.7 ± 12.6	152.0

Table S155: Densities computed from MC and MD simulations (ρ^{MC} and ρ^{MD}), densities obtained from REFPROP[10] (ρ^{REFP}), isothermal compressibilities computed from MC simulations (β_T^{MC}), isothermal compressibilities obtained from REFPROP[10] (β_T^{REFP}), thermal expansion coefficients computed from MC simulations (α_P^{MC}), thermal expansion coefficients obtained from REFPROP[10] (α_P^{REFP}), Joule Thomson coefficients computed from MC simulations (μ_{JT}^{MC}), and Joule Thomson coefficients obtained from REFPROP[10] (μ_{JT}^{REFP}) of CO_2-CH_4 binary mixture with 5 mole% impurity of CH_4 at 273 K and pressures ranging from 20 bar to 200 bar.

P/ [bar]	ρ^{MC}/ [kg/m^3]	ρ^{MD}/ [kg/m^3]	ρ^{REFP}/ [kg/m^3]	β_T^{MC}/ [10^{-5}/bar]	β_T^{REFP}/ [10^{-5}/bar]	α_P^{MC}/ [10^{-4}/K]	α_P^{REFP}/ [10^{-4}/K]	μ_{JT}^{MC}/ [10^{-3} K/bar]	μ_{JT}^{REFP}/ [10^{-3} K/bar]
20	43.0	45.3 ± 0.1	43.8	5806.1 ± 26.4	5952.2	57.1 ± 0.3	60.6	1206.0 ± 17.3	1336.1
40	108.8 ± 0.3	122.7 ± 1.6	240.0	4379.8 ± 78.4	-3878.5	136.2 ± 2.8	-539.4	1291.9 ± 39.1	–
60	885.0 ± 1.5	933.4 ± 2.5	874.8	84.1 ± 3.5	113.6	66.5 ± 2.4	76.6	36.1 ± 3.0	48.7
80	898.8 ± 2.1	946.1 ± 1.2	892.7	75.0 ± 4.6	90.9	61.7 ± 3.6	65.7	30.7 ± 4.5	37.1
100	910.1 ± 1.0	909.2 ± 1.9	907.7	64.6 ± 2.5	76.5	55.5 ± 2.3	58.4	23.9 ± 3.0	28.7
120	921.9 ± 0.7	920.2 ± 1.3	920.7	56.1 ± 3.2	66.3	50.1 ± 2.7	53.1	17.7 ± 3.6	22.2
140	932.0 ± 1.8	930.8 ± 0.7	932.2	50.2 ± 4.2	58.8	46.4 ± 3.6	49.0	13.0 ± 4.9	17.0
160	940.9 ± 1.5	940.0 ± 0.8	942.7	46.6 ± 1.7	52.9	44.3 ± 1.9	45.7	10.4 ± 2.6	12.7
180	950.0 ± 0.7	948.0 ± 1.0	952.2	42.5 ± 1.1	48.2	42.0 ± 0.6	43.0	7.2 ± 0.9	9.0
200	958.5 ± 1.5	956.3 ± 1.1	961.0	38.9 ± 1.3	44.3	39.3 ± 1.2	40.7	3.7 ± 1.6	5.9

Table S156: Heat capacities at constant volume computed from MC simulations (c_V^{MC}), heat capacities at constant volume obtained from REFPROP[10] (c_V^{REFP}), heat capacities at constant pressure computed from MC simulations (c_P^{MC}), heat capacities at constant pressure obtained from REFPROP[10] (c_P^{REFP}), speed of sound computed from MC simulations (c^{MC}), speed of sound obtained from REFPROP[10] (c^{REFP}), viscosities computed from MD simulations (η^{MD}), and viscosities obtained from REFPROP[10] (η^{REFP}) of CO_2-CH_4 binary mixture with 5 mole% impurity of CH_4 at 273 K and pressures ranging from 20 bar to 200 bar.

P/ [bar]	c_V^{MC}/ [J/mol K]	c_V^{REFP}/ [J/mol K]	c_P^{MC}/ [J/mol K]	c_P^{REFP}/ [J/mol K]	c^{MC}/ [m/s]	c^{REFP}/ [m/s]	η^{MD}/ [μPa s]	η^{REFP}/ [μPa s]
20	30.8	31.3	46.0 ± 0.1	47.6	244.7 ± 0.6	241.8	4.9 ± 5.2	13.9
40	36.7 ± 0.2	–	82.4 ± 1.0	–	217.0 ± 2.4	–	15.5 ± 1.3	18.4
60	39.5 ± 0.1	40.5	108.8 ± 2.0	109.1	607.9 ± 14.0	521.0	109.1 ± 3.4	90.8
80	39.6 ± 0.2	40.1	105.7 ± 3.7	102.0	628.9 ± 22.3	559.4	115.2 ± 2.5	95.6
100	39.6 ± 0.2	40.0	100.8 ± 2.9	97.1	658.3 ± 16.1	591.8	106.3 ± 5.2	99.9
120	39.6 ± 0.2	39.9	96.2 ± 3.1	93.5	685.1 ± 22.3	620.0	111.9 ± 5.6	103.8
140	39.6 ± 0.2	39.8	93.1 ± 4.0	90.8	708.4 ± 33.4	645.4	122.9 ± 11.8	107.4
160	39.6 ± 0.2	39.8	91.9 ± 2.9	88.5	727.8 ± 17.4	668.4	114.7 ± 5.1	110.9
180	39.8 ± 0.2	39.7	90.4 ± 0.8	86.7	749.8 ± 10.3	689.7	127.9 ± 9.8	114.1
200	39.9	39.7	87.8 ± 1.3	85.1	768.5 ± 14.5	709.5	124.0 ± 9.1	117.3

Table S157: Densities computed from MC and MD simulations (ρ^{MC} and ρ^{MD}), densities obtained from REFPROP[10] (ρ^{REFP}), isothermal compressibilities computed from MC simulations (β_T^{MC}), isothermal compressibilities obtained from REFPROP[10] (β_T^{REFP}), thermal expansion coefficients computed from MC simulations (α_P^{MC}), thermal expansion coefficients obtained from REFPROP[10] (α_P^{REFP}), Joule Thomson coefficients computed from MC simulations ($\mu_{\mathrm{JT}}^{\mathrm{MC}}$), and Joule Thomson coefficients obtained from REFPROP[10] ($\mu_{\mathrm{JT}}^{\mathrm{REFP}}$) of CO_2-CH_4 binary mixture with 5 mole% impurity of CH_4 at 293 K and pressures ranging from 20 bar to 200 bar.

P/ [bar]	ρ^{MC}/ [kg/m^3]	ρ^{MD}/ [kg/m^3]	ρ^{REFP}/ [kg/m^3]	β_T^{MC}/ [10^{-5}/bar]	β_T^{REFP}/ [10^{-5}/bar]	α_P^{MC}/ [10^{-4}/K]	α_P^{REFP}/ [10^{-4}/K]	$\mu_{\mathrm{JT}}^{\mathrm{MC}}$/ [$10^{-3}$ K/bar]	$\mu_{\mathrm{JT}}^{\mathrm{REFP}}$/ [$10^{-3}$ K/bar]
20	38.7	40.7 ± 0.1	39.2	5621.1 ± 15.0	5665.6	48.2 ± 0.1	49.7	1029.1 ± 6.1	1104.0
40	89.4 ± 0.1	95.1 ± 0.3	92.4	3394.0 ± 29.1	3522.9	77.6 ± 0.9	85.0	1037.8 ± 24.0	1100.2
60	175.4 ± 0.8	638.4 ± 137.3	190.6	3921.4 ± 139.5	4542.9	217.6 ± 7.3	283.2	1032.2 ± 47.4	1019.5
80	754.5 ± 2.6	813.1 ± 1.7	730.9	292.4 ± 30.6	427.9	141.3 ± 11.1	172.9	114.3 ± 12.8	146.2
100	789.5 ± 3.3	784.1 ± 2.2	776.7	167.4 ± 19.2	225.5	94.2 ± 9.4	109.0	76.5 ± 12.8	95.6
120	813.4 ± 1.6	810.3 ± 1.1	806.2	124.7 ± 8.8	157.2	77.0 ± 4.7	84.9	58.2 ± 6.8	70.6
140	832.4 ± 1.9	830.5 ± 1.6	828.8	104.4 ± 3.5	122.0	69.0 ± 2.3	71.6	48.3 ± 3.3	54.6
160	848.4 ± 1.5	846.6 ± 1.3	847.3	87.5 ± 4.4	100.2	61.0 ± 3.0	62.8	38.7 ± 4.5	43.2
180	862.2 ± 1.4	859.8 ± 1.0	863.1	75.6 ± 2.3	85.3	55.2 ± 1.3	56.5	31.1 ± 2.0	34.5
200	875.3 ± 1.3	872.8 ± 1.1	876.9	66.9 ± 4.3	74.4	51.0 ± 3.2	51.7	25.4 ± 4.9	27.6

Table S158: Heat capacities at constant volume computed from MC simulations (c_V^{MC}), heat capacities at constant volume obtained from REFPROP[10] (c_V^{REFP}), heat capacities at constant pressure computed from MC simulations (c_P^{MC}), heat capacities at constant pressure obtained from REFPROP[10] (c_P^{REFP}), speed of sound computed from MC simulations (c^{MC}), speed of sound obtained from REFPROP[10] (c^{REFP}), viscosities computed from MD simulations (η^{MD}), and viscosities obtained from REFPROP[10] (η^{REFP}) of CO_2-CH_4 binary mixture with 5 mole% impurity of CH_4 at 293 K and pressures ranging from 20 bar to 200 bar.

P/ [bar]	c_V^{MC}/ [J/mol K]	c_V^{REFP}/ [J/mol K]	c_P^{MC}/ [J/mol K]	c_P^{REFP}/ [J/mol K]	c^{MC}/ [m/s]	c^{REFP}/ [m/s]	η^{MD}/ [μPa s]	η^{REFP}/ [μPa s]
20	30.8	31.0	44.1	44.9	256.6 ± 0.4	255.1	5.1 ± 3.5	14.9
40	33.7	34.8	58.4 ± 0.5	62.5	239.2 ± 1.4	234.9	17.4 ± 1.8	15.5
60	39.7 ± 0.5	44.4	126.6 ± 2.9	160.0	215.3 ± 4.7	204.0	41.0 ± 15.4	17.8
80	39.9 ± 0.2	42.8	155.1 ± 6.6	162.2	420.0 ± 23.8	348.0	76.3 ± 1.4	62.0
100	39.5 ± 0.2	41.1	124.1 ± 7.7	125.9	487.6 ± 31.7	418.0	75.9 ± 6.0	69.7
120	39.2 ± 0.1	40.4	113.1 ± 4.2	111.5	533.2 ± 21.3	466.5	81.7 ± 6.0	75.3
140	39.2 ± 0.2	40.0	108.2 ± 2.3	103.3	563.5 ± 11.3	505.1	85.3 ± 4.3	79.9
160	39.1 ± 0.2	39.8	102.2 ± 3.2	97.8	593.0 ± 17.7	537.8	89.5 ± 7.2	84.0
180	39.2 ± 0.1	39.7	98.0 ± 1.2	93.8	619.5 ± 10.1	566.5	90.7 ± 8.1	87.7
200	39.1 ± 0.1	39.6	94.8 ± 3.5	90.7	643.6 ± 24.1	592.4	106.2 ± 24.0	91.1

Table S159: Densities computed from MC and MD simulations (ρ^{MC} and ρ^{MD}), densities obtained from REFPROP[10] (ρ^{REFP}), isothermal compressibilities computed from MC simulations (β_T^{MC}), isothermal compressibilities obtained from REFPROP[10] (β_T^{REFP}), thermal expansion coefficients computed from MC simulations (α_P^{MC}), thermal expansion coefficients obtained from REFPROP[10] (α_P^{REFP}), Joule Thomson coefficients computed from MC simulations (μ_{JT}^{MC}), and Joule Thomson coefficients obtained from REFPROP[10] (μ_{JT}^{REFP}) of CO_2-CH_4 binary mixture with 5 mole% impurity of CH_4 at 313 K and pressures ranging from 20 bar to 200 bar.

P/ [bar]	ρ^{MC}/ [kg/m^3]	ρ^{MD}/ [kg/m^3]	ρ^{REFP}/ [kg/m^3]	β_T^{MC}/ [10^{-5}/bar]	β_T^{REFP}/ [10^{-5}/bar]	α_P^{MC}/ [10^{-4}/K]	α_P^{REFP}/ [10^{-4}/K]	μ_{JT}^{MC}/ [10^{-3} K/bar]	μ_{JT}^{REFP}/ [10^{-3} K/bar]
20	35.4	37.2 ± 0.1	35.8	5456.2 ± 33.6	5493.3	41.8 ± 0.2	42.8	861.4 ± 19.5	929.1
40	78.3	82.8 ± 0.3	80.1	3074.4 ± 22.5	3139.7	58.2 ± 0.4	61.6	865.6 ± 13.4	920.6
60	134.3 ± 0.2	146.2 ± 0.6	139.9	2480.6 ± 22.9	2586.4	91.4 ± 0.9	100.7	857.3 ± 14.7	886.3
80	222.9 ± 1.0	271.5 ± 6.6	238.0	2844.5 ± 157.3	2912.6	204.3 ± 12.1	224.6	805.6 ± 68.8	778.3
100	471.6 ± 17.1	467.5 ± 8.1	466.2	3102.3 ± 213.4	2832.1	603.7 ± 58.3	512.1	422.4 ± 61.9	441.0
120	632.0 ± 6.7	628.4 ± 3.5	620.8	604.2 ± 24.8	710.1	198.4 ± 6.1	208.1	195.4 ± 9.1	217.7
140	692.6 ± 2.3	691.6 ± 3.8	685.0	310.8 ± 25.2	352.4	126.2 ± 9.9	128.5	130.2 ± 15.4	142.0
160	728.0 ± 3.6	725.2 ± 1.0	725.1	217.1 ± 21.0	231.9	99.9 ± 6.8	97.1	100.2 ± 10.5	104.0
180	755.5 ± 2.0	753.5 ± 1.9	754.6	156.6 ± 2.0	172.7	80.5 ± 1.8	80.0	76.1 ± 3.3	80.5
200	776.9 ± 2.1	775.0 ± 0.7	778.1	124.4 ± 3.5	137.6	68.0 ± 1.6	69.1	59.9 ± 2.9	64.2

Table S160: Heat capacities at constant volume computed from MC simulations (c_V^{MC}), heat capacities at constant volume obtained from REFPROP[10] (c_V^{REFP}), heat capacities at constant pressure computed from MC simulations (c_P^{MC}), heat capacities at constant pressure obtained from REFPROP[10] (c_P^{REFP}), speed of sound computed from MC simulations (c^{MC}), speed of sound obtained from REFPROP[10] (c^{REFP}), viscosities computed from MD simulations (η^{MD}), and viscosities obtained from REFPROP[10] (η^{REFP}) of CO_2-CH_4 binary mixture with 5 mole% impurity of CH_4 at 313 K and pressures ranging from 20 bar to 200 bar.

P/ [bar]	c_V^{MC}/ [J/mol K]	c_V^{REFP}/ [J/mol K]	c_P^{MC}/ [J/mol K]	c_P^{REFP}/ [J/mol K]	c^{MC}/ [m/s]	c^{REFP}/ [m/s]	η^{MD}/ [μPa s]	η^{REFP}/ [μPa s]
20	31.2	31.2	43.2 ± 0.1	43.6	268.1 ± 0.8	266.7	12.3 ± 3.9	15.8
40	32.9	33.5	51.8 ± 0.1	53.6	255.5 ± 1.0	252.3	17.3 ± 2.1	16.4
60	35.3 ± 0.1	36.6	68.9 ± 0.5	74.0	241.9 ± 1.4	236.4	19.6 ± 2.0	17.6
80	39.4 ± 0.2	41.7	128.0 ± 6.2	138.7	226.3 ± 8.3	219.2	23.9 ± 2.1	20.5
100	42.2 ± 0.5	46.6	383.5 ± 37.8	311.5	249.3 ± 15.7	225.0	33.5 ± 3.3	33.2
120	40.4 ± 0.2	42.8	179.8 ± 4.7	173.8	341.6 ± 8.5	303.6	51.5 ± 6.0	47.9
140	39.4 ± 0.2	41.2	139.5 ± 7.8	132.4	405.5 ± 20.0	365.0	64.2 ± 10.4	55.9
160	39.2 ± 0.3	40.4	124.3 ± 4.0	115.2	447.9 ± 22.9	411.8	67.6 ± 3.7	61.7
180	39.2 ± 0.2	40.0	112.6 ± 2.3	105.6	493.1 ± 6.1	450.0	66.0 ± 1.4	66.4
200	39.2 ± 0.2	39.7	103.2 ± 1.4	99.2	522.0 ± 8.3	482.9	72.6 ± 4.6	70.4

S15.12 Thermodynamic and Transport Properties Data of binary CO_2 mixture with 10 mole% Impurity of CH_4

Table S161: Densities computed from MC and MD simulations (ρ^{MC} and ρ^{MD}), densities obtained from REFPROP[10] (ρ^{REFP}), isothermal compressibilities computed from MC simulations (β_T^{MC}), isothermal compressibilities obtained from REFPROP[10] (β_T^{REFP}), thermal expansion coefficients computed from MC simulations (α_P^{MC}), thermal expansion coefficients obtained from REFPROP[10] (α_P^{REFP}), Joule Thomson coefficients computed from MC simulations ($\mu_{\mathrm{JT}}^{\mathrm{MC}}$), and Joule Thomson coefficients obtained from REFPROP[10] ($\mu_{\mathrm{JT}}^{\mathrm{REFP}}$) of CO_2-CH_4 binary mixture with 10 mole% impurity of CH_4 at 253 K and pressures ranging from 20 bar to 200 bar.

P/ [bar]	ρ^{MC}/ [kg/m^3]	ρ^{MD}/ [kg/m^3]	ρ^{REFP}/ [kg/m^3]	β_T^{MC}/ [10^{-5}/bar]	β_T^{REFP}/ [10^{-5}/bar]	α_P^{MC}/ [10^{-4}/K]	α_P^{REFP}/ [10^{-4}/K]	$\mu_{\mathrm{JT}}^{\mathrm{MC}}$/ [$10^{-3}$ K/bar]	$\mu_{\mathrm{JT}}^{\mathrm{REFP}}$/ [$10^{-3}$ K/bar]
20	46.8	51.9 ± 0.2	48.0	6213.3 ± 60.0	6390.5	71.6 ± 0.8	78.0	1445.4 ± 37.2	1576.0
40	916.4 ± 1.3	1023.1 ± 1.1	679.8	53.6 ± 1.7	-172.0	51.8 ± 1.1	6.8	14.4 ± 1.3	–
60	926.9 ± 0.8	1030.3 ± 0.8	917.8	48.0 ± 1.7	61.4	47.8 ± 1.3	53.6	10.0 ± 1.6	17.3
80	935.4 ± 1.1	1037.5 ± 1.1	928.5	45.4 ± 2.0	54.4	46.6 ± 2.1	49.3	8.5 ± 2.6	12.3
100	942.6 ± 0.5	941.4 ± 0.6	938.1	41.9 ± 2.0	48.9	44.3 ± 2.3	46.0	5.7 ± 2.8	8.3
120	951.0 ± 1.1	948.9 ± 0.7	946.9	38.3 ± 0.7	44.6	41.6 ± 0.9	43.3	2.5 ± 1.0	4.8
140	957.8 ± 1.4	955.6 ± 1.0	955.0	35.8 ± 0.9	41.0	40.1 ± 1.2	41.0	0.7 ± 1.4	1.9
160	963.4 ± 0.8	963.0 ± 0.7	962.6	33.4 ± 1.4	38.0	37.8 ± 1.7	39.0	-2.1 ± 2.1	-0.7
180	971.0 ± 0.9	968.7 ± 0.7	969.7	31.1 ± 0.6	35.5	36.3 ± 0.5	37.3	-4.0 ± 0.7	-2.9
200	976.5 ± 1.0	975.3 ± 0.7	976.4	29.9 ± 0.8	33.3	35.5 ± 1.1	35.8	-5.1 ± 1.4	-4.9

Table S162: Heat capacities at constant volume computed from MC simulations (c_V^{MC}), heat capacities at constant volume obtained from REFPROP[10] (c_V^{REFP}), heat capacities at constant pressure computed from MC simulations (c_P^{MC}), heat capacities at constant pressure obtained from REFPROP[10] (c_P^{REFP}), speed of sound computed from MC simulations (c^{MC}), speed of sound obtained from REFPROP[10] (c^{REFP}), viscosities computed from MD simulations (η^{MD}), and viscosities obtained from REFPROP[10] (η^{REFP}) of CO_2-CH_4 binary mixture with 10 mole% impurity of CH_4 at 253 K and pressures ranging from 20 bar to 200 bar.

P/ [bar]	c_V^{MC}/ [J/mol K]	c_V^{REFP}/ [J/mol K]	c_P^{MC}/ [J/mol K]	c_P^{REFP}/ [J/mol K]	c^{MC}/ [m/s]	c^{REFP}/ [m/s]	η^{MD}/ [μPa s]	η^{REFP}/ [μPa s]
20	31.1	32.3	49.5 ± 0.2	53.0	233.9 ± 1.3	231.2	10.4 ± 3.0	12.8
40	39.6 ± 0.3	–	96.8 ± 0.7	–	705.3 ± 11.8	–	147.2 ± 3.1	58.3
60	39.5 ± 0.1	39.1	93.3 ± 1.2	92.2	728.9 ± 14.0	646.4	152.0 ± 2.7	110.4
80	39.7 ± 0.3	39.1	93.0 ± 3.0	89.4	742.4 ± 20.4	673.3	154.4 ± 4.9	114.2
100	39.7 ± 0.2	39.0	91.2 ± 3.1	87.1	762.9 ± 22.3	697.6	119.6 ± 14.6	117.9
120	39.6 ± 0.2	39.0	89.1 ± 1.2	85.3	786.4 ± 9.4	719.8	131.9 ± 4.9	121.4
140	39.7 ± 0.2	39.0	88.5 ± 1.8	83.7	806.2 ± 13.3	740.3	133.9 ± 4.9	124.7
160	39.8 ± 0.2	39.0	86.1 ± 2.2	82.4	819.7 ± 20.4	759.5	136.1 ± 5.4	128.0
180	39.9 ± 0.2	39.0	85.4 ± 0.7	81.3	841.9 ± 8.7	777.5	144.3 ± 10.5	131.1
200	40.2 ± 0.2	39.1	84.8 ± 1.6	80.3	850.4 ± 14.5	794.6	200.3 ± 120.8	134.1

Table S163: Densities computed from MC and MD simulations (ρ^{MC} and ρ^{MD}), densities obtained from REFPROP[10] (ρ^{REFP}), isothermal compressibilities computed from MC simulations (β_T^{MC}), isothermal compressibilities obtained from REFPROP[10] (β_T^{REFP}), thermal expansion coefficients computed from MC simulations (α_P^{MC}), thermal expansion coefficients obtained from REFPROP[10] (α_P^{REFP}), Joule Thomson coefficients computed from MC simulations ($\mu_{\mathrm{JT}}^{\mathrm{MC}}$), and Joule Thomson coefficients obtained from REFPROP[10] ($\mu_{\mathrm{JT}}^{\mathrm{REFP}}$) of CO_2-CH_4 binary mixture with 10 mole% impurity of CH_4 at 273 K and pressures ranging from 20 bar to 200 bar.

P/ [bar]	ρ^{MC}/ [kg/m^3]	ρ^{MD}/ [kg/m^3]	ρ^{REFP}/ [kg/m^3]	β_T^{MC}/ [10^{-5}/bar]	β_T^{REFP}/ [10^{-5}/bar]	α_P^{MC}/ [10^{-4}/K]	α_P^{REFP}/ [10^{-4}/K]	$\mu_{\mathrm{JT}}^{\mathrm{MC}}$/ [$10^{-3}$ K/bar]	$\mu_{\mathrm{JT}}^{\mathrm{REFP}}$/ [$10^{-3}$ K/bar]
20	41.2	45.3 ± 0.1	42.0	5788.4 ± 21.0	5881.4	56.0 ± 0.2	58.8	1170.7 ± 11.4	1272.7
40	102.0 ± 0.2	122.7 ± 1.6	107.4	4056.6 ± 62.9	4355.8	118.6 ± 2.5	139.5	1223.9 ± 40.0	1264.2
60	814.1 ± 1.4	933.4 ± 2.5	794.8	128.9 ± 10.6	188.7	84.2 ± 5.5	103.6	56.5 ± 6.9	78.3
80	835.3 ± 1.6	946.1 ± 1.2	820.1	92.4 ± 1.8	132.5	67.3 ± 1.9	80.9	39.2 ± 2.6	56.5
100	850.1 ± 1.3	847.3 ± 2.0	839.5	83.8 ± 5.7	103.9	63.2 ± 3.9	68.5	34.3 ± 5.1	42.7
120	863.0 ± 1.4	860.2 ± 0.5	855.5	73.5 ± 4.7	86.1	58.5 ± 3.0	60.4	28.5 ± 3.9	32.9
140	875.6 ± 0.9	873.0 ± 0.9	869.3	60.9 ± 3.5	73.9	50.7 ± 2.9	54.6	19.4 ± 4.1	25.5
160	885.5 ± 1.3	883.5 ± 0.6	881.4	54.8 ± 1.2	65.0	47.4 ± 1.3	50.1	15.0 ± 1.8	19.5
180	895.4 ± 1.3	891.9 ± 1.2	892.3	50.1 ± 1.5	58.1	45.0 ± 1.0	46.6	11.7 ± 1.4	14.6
200	903.4 ± 1.1	901.5 ± 0.8	902.2	46.8 ± 3.2	52.6	42.9 ± 2.7	43.7	8.8 ± 3.8	10.5

Table S164: Heat capacities at constant volume computed from MC simulations (c_V^{MC}), heat capacities at constant volume obtained from REFPROP[10] (c_V^{REFP}), heat capacities at constant pressure computed from MC simulations (c_P^{MC}), heat capacities at constant pressure obtained from REFPROP[10] (c_P^{REFP}), speed of sound computed from MC simulations (c^{MC}), speed of sound obtained from REFPROP[10] (c^{REFP}), viscosities computed from MD simulations (η^{MD}), and viscosities obtained from REFPROP[10] (η^{REFP}) of CO_2-CH_4 binary mixture with 10 mole% impurity of CH_4 at 273 K and pressures ranging from 20 bar to 200 bar.

P/ [bar]	c_V^{MC}/ [J/mol K]	c_V^{REFP}/ [J/mol K]	c_P^{MC}/ [J/mol K]	c_P^{REFP}/ [J/mol K]	c^{MC}/ [m/s]	c^{REFP}/ [m/s]	η^{MD}/ [μPa s]	η^{REFP}/ [μPa s]
20	30.4	30.9	45.2 ± 0.1	46.6	249.5 ± 0.5	247.3	4.9 ± 5.2	13.8
40	35.5 ± 0.2	38.4	73.9 ± 0.9	85.2	224.2 ± 2.4	217.7	15.5 ± 1.3	14.7
60	39.6 ± 0.3	40.5	116.3 ± 4.5	121.1	529.0 ± 24.0	446.4	109.1 ± 3.4	75.9
80	39.0 ± 0.1	39.8	105.4 ± 2.6	107.5	591.3 ± 9.4	498.7	115.2 ± 2.5	81.5
100	39.0 ± 0.2	39.4	102.6 ± 3.8	99.9	607.8 ± 23.6	539.2	89.9 ± 2.8	86.2
120	39.3 ± 0.1	39.2	100.2 ± 2.4	94.8	634.0 ± 21.7	573.1	98.3 ± 4.4	90.3
140	39.0 ± 0.1	39.0	93.5 ± 3.3	91.2	670.9 ± 22.7	602.7	102.5 ± 4.4	94.0
160	39.1 ± 0.2	39.0	91.3 ± 2.1	88.3	693.6 ± 11.2	629.0	100.9 ± 1.2	97.5
180	39.3 ± 0.1	38.9	89.9 ± 0.9	86.1	713.7 ± 11.5	653.0	107.7 ± 3.7	100.8
200	39.1 ± 0.2	38.9	88.2 ± 2.9	84.2	730.2 ± 28.2	675.0	117.6 ± 10.9	103.9

Table S165: Densities computed from MC and MD simulations (ρ^{MC} and ρ^{MD}), densities obtained from REFPROP[10] (ρ^{REFP}), isothermal compressibilities computed from MC simulations (β_T^{MC}), isothermal compressibilities obtained from REFPROP[10] (β_T^{REFP}), thermal expansion coefficients computed from MC simulations (α_P^{MC}), thermal expansion coefficients obtained from REFPROP[10] (α_P^{REFP}), Joule Thomson coefficients computed from MC simulations ($\mu_{\mathrm{JT}}^{\mathrm{MC}}$), and Joule Thomson coefficients obtained from REFPROP[10] ($\mu_{\mathrm{JT}}^{\mathrm{REFP}}$) of CO_2-CH_4 binary mixture with 10 mole% impurity of CH_4 at 293 K and pressures ranging from 20 bar to 200 bar.

P/ [bar]	ρ^{MC}/ [kg/m^3]	ρ^{MD}/ [kg/m^3]	ρ^{REFP}/ [kg/m^3]	β_T^{MC}/ [10^{-5}/bar]	β_T^{REFP}/ [10^{-5}/bar]	α_P^{MC}/ [10^{-4}/K]	α_P^{REFP}/ [10^{-4}/K]	$\mu_{\mathrm{JT}}^{\mathrm{MC}}$/ [$10^{-3}$ K/bar]	$\mu_{\mathrm{JT}}^{\mathrm{REFP}}$/ [$10^{-3}$ K/bar]
20	37.2	40.7 ± 0.1	37.7	5546.3 ± 33.9	5622.1	47.0 ± 0.4	48.7	961.1 ± 27.0	1053.5
40	85.1 ± 0.1	95.1 ± 0.3	87.6	3320.3 ± 33.0	3406.7	73.5 ± 1.0	79.1	993.8 ± 26.9	1044.9
60	158.6 ± 0.4	638.4 ± 137.3	168.7	3212.2 ± 74.4	3504.6	158.3 ± 5.0	188.1	969.3 ± 46.4	978.2
80	658.8 ± 4.0	814.5 ± 1.7	587.2	687.7 ± 115.8	1935.7	247.9 ± 31.5	500.9	187.7 ± 31.5	305.4
100	713.8 ± 1.6	711.8 ± 1.6	688.2	266.6 ± 13.7	404.6	122.9 ± 5.7	154.7	109.9 ± 7.8	145.4
120	748.7 ± 2.9	744.3 ± 2.0	731.1	169.6 ± 10.9	233.0	90.0 ± 4.7	105.2	77.3 ± 6.9	98.8
140	771.1 ± 2.4	769.6 ± 1.4	760.2	141.0 ± 7.4	165.7	80.3 ± 3.1	83.5	65.0 ± 4.5	73.6
160	789.4 ± 1.6	787.6 ± 0.9	782.8	112.0 ± 8.1	129.4	68.5 ± 3.5	70.8	50.7 ± 5.4	57.1
180	806.0 ± 1.1	802.9 ± 1.3	801.3	91.4 ± 4.7	106.5	59.4 ± 2.8	62.3	39.1 ± 4.5	45.2
200	817.8 ± 0.4	818.9 ± 0.8	817.2	78.8 ± 3.1	90.7	54.0 ± 1.7	56.2	31.4 ± 2.7	36.1

Table S166: Heat capacities at constant volume computed from MC simulations (c_V^{MC}), heat capacities at constant volume obtained from REFPROP[10] (c_V^{REFP}), heat capacities at constant pressure computed from MC simulations (c_P^{MC}), heat capacities at constant pressure obtained from REFPROP[10] (c_P^{REFP}), speed of sound computed from MC simulations (c^{MC}), speed of sound obtained from REFPROP[10] (c^{REFP}), viscosities computed from MD simulations (η^{MD}), and viscosities obtained from REFPROP[10] (η^{REFP}) of CO_2-CH_4 binary mixture with 10 mole% impurity of CH_4 at 293 K and pressures ranging from 20 bar to 200 bar.

P/ [bar]	c_V^{MC}/ [J/mol K]	c_V^{REFP}/ [J/mol K]	c_P^{MC}/ [J/mol K]	c_P^{REFP}/ [J/mol K]	c^{MC}/ [m/s]	c^{REFP}/ [m/s]	η^{MD}/ [μPa s]	η^{REFP}/ [μPa s]
20	30.6	30.7	43.4 ± 0.1	44.2	262.5 ± 0.9	260.5	5.1 ± 3.5	14.8
40	33.1	34.1	56.2 ± 0.4	59.4	245.2 ± 1.6	241.8	17.4 ± 1.8	15.4
60	37.7 ± 0.3	40.4	97.5 ± 2.5	112.7	225.2 ± 3.9	217.2	41.0 ± 15.4	17.2
80	40.5 ± 0.3	47.7	208.8 ± 16.7	314.3	337.5 ± 31.5	240.6	77.7 ± 3.3	44.5
100	39.3 ± 0.1	41.7	136.8 ± 4.0	145.5	427.6 ± 12.7	354.0	63.2 ± 5.3	57.4
120	38.8 ± 0.1	40.3	116.5 ± 3.5	118.7	486.1 ± 17.2	415.9	72.3 ± 5.2	64.1
140	38.9 ± 0.2	39.7	111.2 ± 2.2	106.5	513.0 ± 14.4	461.7	74.4 ± 6.3	69.3
160	38.6 ± 0.2	39.3	103.7 ± 2.5	99.1	550.8 ± 20.9	499.1	80.3 ± 5.5	73.6
180	38.7 ± 0.2	39.1	97.0 ± 2.9	94.1	583.4 ± 17.4	531.3	83.4 ± 4.1	77.4
200	38.8 ± 0.3	38.9	93.4 ± 1.4	90.4	611.0 ± 13.0	559.7	89.8 ± 7.2	80.8

Table S167: Densities computed from MC and MD simulations (ρ^{MC} and ρ^{MD}), densities obtained from REFPROP[10] (ρ^{REFP}), isothermal compressibilities computed from MC simulations (β_T^{MC}), isothermal compressibilities obtained from REFPROP[10] (β_T^{REFP}), thermal expansion coefficients computed from MC simulations (α_P^{MC}), thermal expansion coefficients obtained from REFPROP[10] (α_P^{REFP}), Joule Thomson coefficients computed from MC simulations ($\mu_{\mathrm{JT}}^{\mathrm{MC}}$), and Joule Thomson coefficients obtained from REFPROP[10] ($\mu_{\mathrm{JT}}^{\mathrm{REFP}}$) of CO_2-CH_4 binary mixture with 10 mole% impurity of CH_4 at 313 K and pressures ranging from 20 bar to 200 bar.

P/ [bar]	ρ^{MC}/ [kg/m^3]	ρ^{MD}/ [kg/m^3]	ρ^{REFP}/ [kg/m^3]	β_T^{MC}/ [10^{-5}/bar]	β_T^{REFP}/ [10^{-5}/bar]	α_P^{MC}/ [10^{-4}/K]	α_P^{REFP}/ [10^{-4}/K]	$\mu_{\mathrm{JT}}^{\mathrm{MC}}$/ [$10^{-3}$ K/bar]	$\mu_{\mathrm{JT}}^{\mathrm{REFP}}$/ [$10^{-3}$ K/bar]
20	34.1	37.2 ± 0.1	34.5	5394.0 ± 33.4	5463.3	41.0 ± 0.2	42.2	804.3 ± 18.7	887.7
40	74.9	82.8 ± 0.3	76.5	3021.8 ± 23.7	3084.2	56.2 ± 0.5	59.0	825.7 ± 18.2	877.2
60	126.9 ± 0.1	146.2 ± 0.6	131.3	2380.8 ± 35.1	2455.9	84.2 ± 1.4	90.9	817.3 ± 23.4	842.0
80	202.7 ± 0.7	271.5 ± 6.6	212.8	2383.8 ± 56.5	2441.5	154.5 ± 4.0	167.1	766.9 ± 28.1	–
100	348.0 ± 4.3	359.2 ± 4.6	356.5	2893.1 ± 121.6	2610.0	395.7 ± 17.1	344.4	550.6 ± 33.8	543.4
120	535.5 ± 5.8	531.7 ± 5.3	519.5	1063.5 ± 121.6	1130.9	269.1 ± 25.8	255.1	276.0 ± 36.0	298.7
140	615.7 ± 1.4	613.1 ± 2.5	605.1	440.1 ± 16.1	516.5	148.8 ± 6.6	154.9	167.8 ± 11.3	187.2
160	665.5 ± 2.2	659.8 ± 1.7	655.2	292.1 ± 30.8	311.5	116.1 ± 11.2	111.2	126.1 ± 18.6	132.0
180	697.2 ± 1.6	693.0 ± 1.4	690.3	192.1 ± 10.4	220.1	87.0 ± 3.8	88.8	91.1 ± 6.7	99.8
200	720.3 ± 0.9	717.6 ± 0.9	717.4	147.1 ± 2.4	169.5	72.3 ± 1.2	75.2	70.4 ± 2.3	78.4

Table S168: Heat capacities at constant volume computed from MC simulations (c_V^{MC}), heat capacities at constant volume obtained from REFPROP[10] (c_V^{REFP}), heat capacities at constant pressure computed from MC simulations (c_P^{MC}), heat capacities at constant pressure obtained from REFPROP[10] (c_P^{REFP}), speed of sound computed from MC simulations (c^{MC}), speed of sound obtained from REFPROP[10] (c^{REFP}), viscosities computed from MD simulations (η^{MD}), and viscosities obtained from REFPROP[10] (η^{REFP}) of CO_2-CH_4 binary mixture with 10 mole% impurity of CH_4 at 313 K and pressures ranging from 20 bar to 200 bar.

P/ [bar]	c_V^{MC}/ [J/mol K]	c_V^{REFP}/ [J/mol K]	c_P^{MC}/ [J/mol K]	c_P^{REFP}/ [J/mol K]	c^{MC}/ [m/s]	c^{REFP}/ [m/s]	η^{MD}/ [μPa s]	η^{REFP}/ [μPa s]
20	30.9	31.0	42.7	43.2	274.1 ± 0.9	272.1	12.3 ± 3.9	15.7
40	32.5	33.0	50.5 ± 0.2	52.1	261.9 ± 1.1	258.6	17.3 ± 2.1	16.3
60	34.6 ± 0.1	35.7	64.9 ± 0.6	68.8	249.2 ± 2.2	244.3	19.6 ± 2.0	17.4
80	37.6 ± 0.2	–	101.7 ± 1.7	–	236.6 ± 3.5	–	23.9 ± 2.1	19.7
100	41.3 ± 0.2	43.6	245.0 ± 9.2	208.0	242.9 ± 7.0	226.4	30.1 ± 6.4	26.3
120	40.2 ± 0.4	42.6	207.0 ± 14.7	185.5	300.6 ± 20.4	272.2	42.4 ± 6.5	38.4
140	39.2 ± 0.2	41.0	145.9 ± 5.4	140.0	370.6 ± 9.7	330.6	46.4 ± 2.1	47.3
160	38.9 ± 0.3	40.1	129.4 ± 8.1	118.2	413.4 ± 25.4	380.2	54.9 ± 4.6	53.5
180	38.7 ± 0.1	39.5	111.9 ± 2.8	106.5	464.5 ± 13.9	421.1	61.5 ± 6.0	58.4
200	38.6 ± 0.1	39.2	102.6 ± 1.2	99.1	501.0 ± 5.2	456.0	68.8 ± 2.2	62.5

S16 Data of thermodynamic and transport properties of ternary systems

This section shows the thermodynamic and transport properties with uncertainties computed from Monte Carlo (MC) and Molecular Dynamics (MD) simulations, respectively, for CO_2 rich ternary mixtures with impurities (Ar, N_2, H_2, listed in Table S169, where compositions are in mole%. The thermodynamic properties computed from MC simulations are (1) densities (ρ), (2) isothermal compressibilities at constant temperature (β_T), (3) thermal expansion coefficients (α_P), (4) heat capacities at constant volume (c_V), (5) heat capacities at constant pressure (c_P), (6) Joule-Thomson Coefficients (μ_{JT}), and (7) speed of sound (c). Transport property, (8) viscosities (η) are computed from MD simulations. Densities computed from MD simulations are also reported in this section. The thermodynamic and transport properties are computed at temperatures 253 K, 273 K, 293 K, and 313K, and pressure ranging from 20 bar to 200 bar in intervals of 20 bar. For each condition (concentration, temperature, and pressure), 10 independent simulations are performed, and each simulation starts with a different initial configuration. These 10 simulations are divided into 5 blocks from which average values and uncertainties of thermodynamic and transport properties are calculated as the standard deviation of 5 blocks. The thermodynamic and transport properties uncertainty values with more than one significant decimal figure are not shown. Properties computed from molecular simulations are compared with data calculated from the GERG-2008 EoS,[30] which are obtained from REFPROP.[10] At conditions close to the critical point, REFPROP[10] fails to calculate heat capacities at constant volume (c_V), heat capacities at constant pressure (c_P), Joule-Thomson Coefficients (μ_{JT}), and speed of sound (c). A similar issue was also encountered during simulations, so the unavailable EoS data and simulation data are represented by "-".

Table S169: Compositions of CO_2 rich ternary mixtures in mole% with impurities (Ar, N_2, H_2, and CH_4) for which the thermodynamics and transport properties are computed at temperatures 253 K, 273 K, 293 K, and 313K, and pressure ranging from 20 bar to 200 bar in intervals of 20 bar. Component with 0 mole% are denoted by -.

No.	Mixture - ID	CO_2 (mole%)	Ar (mole%)	N_2 (mole%)	H_2 (mole%)	CH_4 (mole%)
1	TM-98-1	98	1	1	-	-
2	TM-98-2	98	-	1	1	-
3	TM-98-3	98	-	-	1	1
4	TM-98-4	98	1	-	-	1
5	TM-98-5	98	1	-	1	-
6	TM-98-6	98	-	1	-	1
7	TM-96-1	96	2	2	-	-
8	TM-96-2	96	-	2	2	-
9	TM-96-3	96	-	-	2	2
10	TM-96-4	96	2	-	-	2
11	TM-96-5	96	2	-	2	-
12	TM-96-6	96	-	2	-	2
13	TM-96-7	96	-	-	1	3
14	TM-96-8	96	-	-	3	1
15	TM-96-9	96	-	1	-	3
16	TM-96-10	96	-	3	-	1
17	TM-96-11	96	-	1	3	-
18	TM-96-12	96	-	3	1	-
19	TM-96-13	96	1	-	-	3
20	TM-96-14	96	3	-	-	1
21	TM-96-15	96	1	-	3	-
22	TM-96-16	96	3	-	1	-
23	TM-96-17	96	1	3	-	-
24	TM-96-18	96	3	1	-	-

S16.1 Data of thermodynamics and transport properties of CO_2 rich ternary mixture with 1 mole% impurity of Ar and 1 mole% impurity of N_2

Table S170: Densities computed from MC and MD simulations (ρ^{MC} and ρ^{MD}), densities obtained from REFPROP[10] (ρ^{REFP}), isothermal compressibilities computed from MC simulations (β_T^{MC}), isothermal compressibilities obtained from REFPROP[10] (β_T^{REFP}), thermal expansion coefficients computed from MC simulations (α_P^{MC}), thermal expansion coefficients obtained from REFPROP[10] (α_P^{REFP}), Joule Thomson coefficients computed from MC simulations ($\mu_{\mathrm{JT}}^{\mathrm{MC}}$), and Joule Thomson coefficients obtained from REFPROP[10] ($\mu_{\mathrm{JT}}^{\mathrm{REFP}}$) of CO_2 rich ternary mixture with 1% impurity of Ar and 1% impurity of N_2 at 253 K and pressures ranging from 20 bar to 200 bar.

P/ [bar]	ρ^{MC}/ [kg/m^3]	ρ^{MD}/ [kg/m^3]	ρ^{REFP}/ [kg/m^3]	β_T^{MC}/ [10^{-5}/bar]	β_T^{REFP}/ [10^{-5}/bar]	α_P^{MC}/ [10^{-4}/K]	α_P^{REFP}/ [10^{-4}/K]	$\mu_{\mathrm{JT}}^{\mathrm{MC}}$/ [$10^{-3}$ K/bar]	$\mu_{\mathrm{JT}}^{\mathrm{REFP}}$/ [$10^{-3}$ K/bar]
20	50.5	51.6 ± 0.2	52.1	6404.9 ± 71.5	6610.9	76.7 ± 0.8	84.7	1570.1 ± 33.9	1699.6
40	1022.0 ± 1.1	1018.4 ± 1.0	1024.5	39.7 ± 1.8	45.7	45.3 ± 2.1	47.1	6.5 ± 2.4	8.8
60	1028.9 ± 1.8	1026.0 ± 0.7	1033.5	35.4 ± 2.2	41.6	41.4 ± 2.7	44.3	2.2 ± 3.2	5.6
80	1035.5 ± 0.8	1034.6 ± 0.9	1041.7	35.3 ± 1.4	38.3	42.2 ± 1.5	41.9	3.0 ± 1.7	2.9
100	1043.4 ± 1.2	1041.6 ± 0.5	1049.5	31.4 ± 0.7	35.6	38.5 ± 1.3	39.9	-1.2 ± 1.5	0.5
120	1049.9 ± 1.1	1047.9 ± 0.4	1056.7	29.7 ± 0.9	33.2	37.1 ± 1.4	38.2	-2.8 ± 1.7	-1.6
140	1057.2 ± 1.0	1054.5 ± 0.7	1063.5	27.6 ± 1.0	31.2	35.4 ± 1.5	36.6	-4.9 ± 1.8	-3.5
160	1062.0 ± 0.7	1060.2 ± 1.1	1070.0	26.5 ± 1.2	29.5	34.3 ± 1.5	35.3	-6.2 ± 1.8	-5.2
180	1067.9 ± 0.9	1066.5 ± 0.1	1076.2	25.3 ± 1.1	27.9	33.7 ± 1.6	34.1	-7.0 ± 1.9	-6.7
200	1072.6 ± 1.1	1071.7 ± 0.6	1082.0	24.8 ± 0.6	26.6	33.1 ± 0.8	33.0	-7.7 ± 0.9	-8.1

Table S171: Heat capacities at constant volume computed from MC simulations (c_V^{MC}), heat capacities at constant volume obtained from REFPROP[10] (c_V^{REFP}), heat capacities at constant pressure computed from MC simulations (c_P^{MC}), heat capacities at constant pressure obtained from REFPROP[10] (c_P^{REFP}), speed of sound computed from MC simulations (c^{MC}), speed of sound obtained from REFPROP[10] (c^{REFP}), viscosities computed from MD simulations (η^{MD}), and viscosities obtained from REFPROP[10] (η^{REFP}) of CO_2 rich ternary mixture with 1% impurity of Ar and 1% impurity of N_2 at 253 K and pressures ranging from 20 bar to 200 bar.

P/ [bar]	c_V^{MC}/ [J/mol K]	c_V^{REFP}/ [J/mol K]	c_P^{MC}/ [J/mol K]	c_P^{REFP}/ [J/mol K]	c^{MC}/ [m/s]	c^{REFP}/ [m/s]	η^{MD}/ [μPa s]	η^{REFP}/ [μPa s]
20	31.8	33.4	52.0 ± 0.2	56.5	224.8 ± 1.3	221.5	10.2 ± 3.2	13.0
40	40.3 ± 0.2	40.7	96.6 ± 3.0	93.4	768.4 ± 21.0	699.7	140.7 ± 2.2	136.3
60	40.3 ± 0.2	40.7	92.5 ± 3.9	91.3	794.1 ± 29.5	721.6	146.5 ± 4.0	140.3
80	40.5 ± 0.2	40.7	94.0 ± 1.7	89.5	796.0 ± 17.6	741.8	148.3 ± 2.4	144.1
100	40.4 ± 0.1	40.7	90.5 ± 2.2	88.0	826.8 ± 14.2	760.6	153.9 ± 7.9	147.8
120	40.6 ± 0.2	40.7	89.4 ± 2.4	86.7	840.8 ± 16.7	778.3	165.3 ± 13.2	151.4
140	40.6 ± 0.2	40.8	88.2 ± 2.5	85.6	862.7 ± 20.3	795.0	177.9 ± 35.4	154.9
160	40.6 ± 0.1	40.8	86.9 ± 2.2	84.6	872.1 ± 22.0	810.8	160.8 ± 7.4	158.3
180	40.7 ± 0.2	40.8	87.0 ± 2.5	83.7	888.4 ± 23.0	825.9	170.5 ± 11.3	161.6
200	40.8 ± 0.1	40.9	86.5 ± 1.4	82.9	893.1 ± 12.5	840.3	173.7 ± 9.5	164.8

Table S172: Densities computed from MC and MD simulations (ρ^{MC} and ρ^{MD}), densities obtained from REFPROP[10] (ρ^{REFP}), isothermal compressibilities computed from MC simulations (β_T^{MC}), isothermal compressibilities obtained from REFPROP[10] (β_T^{REFP}), thermal expansion coefficients computed from MC simulations (α_P^{MC}), thermal expansion coefficients obtained from REFPROP[10] (α_P^{REFP}), Joule Thomson coefficients computed from MC simulations ($\mu_{\mathrm{JT}}^{\mathrm{MC}}$), and Joule Thomson coefficients obtained from REFPROP[10] ($\mu_{\mathrm{JT}}^{\mathrm{REFP}}$) of CO_2 rich ternary mixture with 1% impurity of Ar and 1% impurity of N_2 at 273 K and pressures ranging from 20 bar to 200 bar.

P/ [bar]	ρ^{MC}/ [kg/m^3]	ρ^{MD}/ [kg/m^3]	ρ^{REFP}/ [kg/m^3]	β_T^{MC}/ [10^{-5}/bar]	β_T^{REFP}/ [10^{-5}/bar]	α_P^{MC}/ [10^{-4}/K]	α_P^{REFP}/ [10^{-4}/K]	$\mu_{\mathrm{JT}}^{\mathrm{MC}}$/ [$10^{-3}$ K/bar]	$\mu_{\mathrm{JT}}^{\mathrm{REFP}}$/ [$10^{-3}$ K/bar]
20	44.3	45.2 ± 0.1	45.2	5841.8 ± 45.0	5980.4	57.8 ± 0.4	61.3	1236.8 ± 21.9	1366.1
40	–	119.1 ± 0.6	495.8	–	225.5	–	-64.1	–	–
60	928.7 ± 1.9	923.4 ± 1.7	927.3	72.5 ± 4.7	92.3	60.5 ± 3.6	68.5	29.2 ± 4.5	38.7
80	940.9 ± 3.1	938.4 ± 2.0	943.1	65.1 ± 4.3	76.9	56.6 ± 3.0	60.4	24.8 ± 3.7	30.0
100	954.2 ± 1.9	951.5 ± 0.7	956.6	56.8 ± 3.9	66.3	51.7 ± 2.9	54.6	19.1 ± 3.8	23.3
120	963.5 ± 1.3	962.5 ± 1.0	968.6	52.3 ± 2.6	58.5	48.8 ± 2.1	50.3	15.7 ± 2.8	18.0
140	973.3 ± 1.4	972.4 ± 1.9	979.4	48.0 ± 2.5	52.5	46.2 ± 2.0	46.8	12.5 ± 2.6	13.6
160	982.4 ± 1.2	980.7 ± 0.9	989.3	44.1 ± 0.6	47.8	44.0 ± 0.8	43.9	9.6 ± 1.1	9.9
180	990.8 ± 1.0	989.0 ± 0.7	998.3	39.8 ± 0.5	43.9	41.0 ± 0.8	41.6	5.8 ± 1.0	6.8
200	998.6 ± 0.8	996.6 ± 0.9	1006.8	36.9 ± 1.3	40.6	38.8 ± 1.5	39.5	3.0 ± 2.1	4.0

Table S173: Heat capacities at constant volume computed from MC simulations (c_V^{MC}), heat capacities at constant volume obtained from REFPROP[10] (c_V^{REFP}), heat capacities at constant pressure computed from MC simulations (c_P^{MC}), heat capacities at constant pressure obtained from REFPROP[10] (c_P^{REFP}), speed of sound computed from MC simulations (c^{MC}), speed of sound obtained from REFPROP[10] (c^{REFP}), viscosities computed from MD simulations (η^{MD}), and viscosities obtained from REFPROP[10] (η^{REFP}) of CO_2 rich ternary mixture with 1% impurity of Ar and 1% impurity of N_2 at 273 K and pressures ranging from 20 bar to 200 bar.

P/ [bar]	c_V^{MC}/ [J/mol K]	c_V^{REFP}/ [J/mol K]	c_P^{MC}/ [J/mol K]	c_P^{REFP}/ [J/mol K]	c^{MC}/ [m/s]	c^{REFP}/ [m/s]	η^{MD}/ [μPa s]	η^{REFP}/ [μPa s]
20	30.8	31.2	46.2 ± 0.1	47.8	240.8 ± 1.0	238.2	3.6 ± 3.2	14.1
40	–	–	–	–	–	–	15.1 ± 1.7	33.0
60	39.7 ± 0.2	40.7	105.0 ± 3.7	106.2	626.2 ± 23.2	552.3	105.7 ± 2.3	100.3
80	39.6 ± 0.1	40.5	102.6 ± 2.8	100.6	650.2 ± 23.2	585.6	111.7 ± 6.8	104.9
100	39.7 ± 0.3	40.3	99.0 ± 2.8	96.6	678.0 ± 25.3	614.5	116.0 ± 5.1	109.1
120	39.7 ± 0.1	40.3	96.5 ± 2.2	93.5	694.4 ± 19.0	640.1	128.5 ± 14.5	113.0
140	39.8 ± 0.2	40.2	94.5 ± 1.9	91.1	712.7 ± 20.3	663.4	129.3 ± 8.9	116.7
160	39.9 ± 0.2	40.2	93.3 ± 1.4	89.1	734.9 ± 7.8	684.9	124.2 ± 4.0	120.2
180	39.9 ± 0.1	40.2	91.0 ± 1.3	87.4	760.7 ± 7.6	704.8	131.5 ± 7.2	123.5
200	39.9 ± 0.2	40.2	88.9 ± 2.2	86.0	777.5 ± 17.0	723.4	134.4 ± 18.1	126.7

Table S174: Densities computed from MC and MD simulations (ρ^{MC} and ρ^{MD}), densities obtained from REFPROP[10] (ρ^{REFP}), isothermal compressibilities computed from MC simulations (β_T^{MC}), isothermal compressibilities obtained from REFPROP[10] (β_T^{REFP}), thermal expansion coefficients computed from MC simulations (α_P^{MC}), thermal expansion coefficients obtained from REFPROP[10] (α_P^{REFP}), Joule Thomson coefficients computed from MC simulations ($\mu_{\mathrm{JT}}^{\mathrm{MC}}$), and Joule Thomson coefficients obtained from REFPROP[10] ($\mu_{\mathrm{JT}}^{\mathrm{REFP}}$) of CO_2 rich ternary mixture with 1% impurity of Ar and 1% impurity of N_2 at 293 K and pressures ranging from 20 bar to 200 bar.

P/ [bar]	ρ^{MC}/ [kg/m^3]	ρ^{MD}/ [kg/m^3]	ρ^{REFP}/ [kg/m^3]	β_T^{MC}/ [10^{-5}/bar]	β_T^{REFP}/ [10^{-5}/bar]	α_P^{MC}/ [10^{-4}/K]	α_P^{REFP}/ [10^{-4}/K]	$\mu_{\mathrm{JT}}^{\mathrm{MC}}$/ [$10^{-3}$ K/bar]	$\mu_{\mathrm{JT}}^{\mathrm{REFP}}$/ [$10^{-3}$ K/bar]
20	39.9	40.7 ± 0.1	40.5	5599.0 ± 26.0	5683.2	48.2 ± 0.2	50.1	1030.1 ± 18.2	1129.6
40	92.6 ± 0.1	94.7 ± 0.2	95.8	3455.3 ± 54.4	3576.1	80.2 ± 1.6	87.6	1071.4 ± 38.0	1127.1
60	185.8 ± 1.0	303.5 ± 103.8	262.3	4381.4 ± 204.9	17 759.0	258.6 ± 15.5	1722.2	1051.2 ± 91.2	–
80	803.9 ± 4.5	798.4 ± 4.4	794.1	223.6 ± 31.7	284.6	120.0 ± 13.0	133.7	94.9 ± 15.4	112.1
100	834.1 ± 3.5	832.7 ± 2.3	830.2	142.5 ± 13.6	177.9	86.4 ± 6.7	95.7	66.5 ± 9.1	78.9
120	855.3 ± 2.0	854.8 ± 0.7	855.9	122.0 ± 6.7	131.9	80.2 ± 4.0	77.9	58.0 ± 5.4	60.0
140	873.4 ± 2.1	871.0 ± 2.2	876.3	95.7 ± 1.8	105.8	66.6 ± 1.2	67.1	44.2 ± 1.8	47.1
160	889.4 ± 1.7	885.6 ± 1.3	893.4	75.8 ± 4.2	88.7	56.1 ± 3.1	59.7	31.8 ± 4.7	37.6
180	899.8 ± 2.3	900.9 ± 1.5	908.2	72.5 ± 4.7	76.6	55.3 ± 3.2	54.2	30.2 ± 4.7	30.1
200	914.2 ± 0.8	912.2 ± 0.6	921.4	60.2 ± 3.3	67.6	48.2 ± 2.8	49.9	21.1 ± 4.3	24.1

Table S175: Heat capacities at constant volume computed from MC simulations (c_V^{MC}), heat capacities at constant volume obtained from REFPROP[10] (c_V^{REFP}), heat capacities at constant pressure computed from MC simulations (c_P^{MC}), heat capacities at constant pressure obtained from REFPROP[10] (c_P^{REFP}), speed of sound computed from MC simulations (c^{MC}), speed of sound obtained from REFPROP[10] (c^{REFP}), viscosities computed from MD simulations (η^{MD}), and viscosities obtained from REFPROP[10] (η^{REFP}) of CO_2 rich ternary mixture with 1% impurity of Ar and 1% impurity of N_2 at 293 K and pressures ranging from 20 bar to 200 bar.

P/ [bar]	c_V^{MC}/ [J/mol K]	c_V^{REFP}/ [J/mol K]	c_P^{MC}/ [J/mol K]	c_P^{REFP}/ [J/mol K]	c^{MC}/ [m/s]	c^{REFP}/ [m/s]	η^{MD}/ [μPa s]	η^{REFP}/ [μPa s]
20	30.8	30.9	44.1 ± 0.1	44.9	253.6 ± 0.6	251.4	9.0 ± 2.1	15.0
40	33.8 ± 0.1	34.8	59.6 ± 0.6	63.6	234.8 ± 2.2	230.8	15.0 ± 1.7	15.8
60	40.3 ± 0.3	–	147.5 ± 7.7	–	211.9 ± 7.5	–	19.3 ± 2.3	20.3
80	39.9 ± 0.3	42.1	144.5 ± 8.8	143.6	448.9 ± 34.7	388.7	72.7 ± 0.5	70.4
100	39.5 ± 0.3	41.0	121.0 ± 5.6	120.6	507.7 ± 27.1	446.3	81.0 ± 4.0	77.1
120	39.4 ± 0.2	40.5	119.0 ± 3.8	109.5	538.1 ± 17.3	489.2	85.8 ± 3.4	82.4
140	39.3 ± 0.1	40.2	108.1 ± 1.5	102.5	573.6 ± 6.7	524.4	91.9 ± 2.9	87.0
160	39.4 ± 0.1	40.1	99.5 ± 3.6	97.7	612.5 ± 20.3	554.8	99.5 ± 5.3	91.0
180	39.3 ± 0.1	39.9	99.9 ± 3.4	94.1	623.8 ± 22.8	581.7	110.8 ± 12.2	94.7
200	39.3 ± 0.1	39.9	93.7 ± 3.4	91.3	657.9 ± 21.8	606.1	101.5 ± 6.8	98.2

Table S176: Densities computed from MC and MD simulations (ρ^{MC} and ρ^{MD}), densities obtained from REFPROP[10] (ρ^{REFP}), isothermal compressibilities computed from MC simulations (β_T^{MC}), isothermal compressibilities obtained from REFPROP[10] (β_T^{REFP}), thermal expansion coefficients computed from MC simulations (α_P^{MC}), thermal expansion coefficients obtained from REFPROP[10] (α_P^{REFP}), Joule Thomson coefficients computed from MC simulations (μ_{JT}^{MC}), and Joule Thomson coefficients obtained from REFPROP[10] (μ_{JT}^{REFP}) of CO_2 rich ternary mixture with 1% impurity of Ar and 1% impurity of N_2 at 313 K and pressures ranging from 20 bar to 200 bar.

P/ [bar]	ρ^{MC}/ [kg/m^3]	ρ^{MD}/ [kg/m^3]	ρ^{REFP}/ [kg/m^3]	β_T^{MC}/ [10^{-5}/bar]	β_T^{REFP}/ [10^{-5}/bar]	α_P^{MC}/ [10^{-4}/K]	α_P^{REFP}/ [10^{-4}/K]	μ_{JT}^{MC}/ [10^{-3} K/bar]	μ_{JT}^{REFP}/ [10^{-3} K/bar]
20	36.4	37.1 ± 0.1	36.9	5456.5 ± 20.1	5505.5	42.1 ± 0.1	43.1	879.4 ± 12.3	951.3
40	80.8	82.8 ± 0.2	82.8	3093.5 ± 31.1	3163.5	59.1 ± 0.6	62.6	884.8 ± 21.6	942.0
60	139.6 ± 0.3	144.1 ± 0.7	145.8	2515.1 ± 49.8	2653.6	95.2 ± 2.1	105.7	873.4 ± 30.7	909.0
80	237.1 ± 0.8	259.5 ± 4.5	257.2	3101.9 ± 26.0	3304.5	235.8 ± 3.3	274.6	815.8 ± 19.6	–
100	549.8 ± 4.3	548.6 ± 8.0	551.4	2648.1 ± 300.8	2146.6	586.7 ± 55.0	478.5	364.5 ± 44.1	356.0
120	681.1 ± 5.7	681.0 ± 4.3	679.1	529.0 ± 69.8	541.8	187.9 ± 17.7	180.3	177.1 ± 22.2	183.0
140	737.9 ± 4.2	731.4 ± 1.2	734.6	246.2 ± 23.5	293.8	109.4 ± 8.6	117.9	110.0 ± 13.3	123.9
160	768.7 ± 4.2	765.9 ± 1.8	771.0	180.2 ± 16.0	201.8	89.2 ± 6.2	91.6	86.1 ± 9.9	92.7
180	792.7 ± 2.6	792.2 ± 1.9	798.6	143.3 ± 7.3	153.9	77.0 ± 3.1	76.6	70.0 ± 5.0	72.6
200	815.6 ± 1.1	811.9 ± 1.3	821.0	110.8 ± 3.1	124.5	64.4 ± 1.9	66.8	53.3 ± 3.3	58.3

Table S177: Heat capacities at constant volume computed from MC simulations (c_V^{MC}), heat capacities at constant volume obtained from REFPROP[10] (c_V^{REFP}), heat capacities at constant pressure computed from MC simulations (c_P^{MC}), heat capacities at constant pressure obtained from REFPROP[10] (c_P^{REFP}), speed of sound computed from MC simulations (c^{MC}), speed of sound obtained from REFPROP[10] (c^{REFP}), viscosities computed from MD simulations (η^{MD}), and viscosities obtained from REFPROP[10] (η^{REFP}) of CO_2 rich ternary mixture with 1% impurity of Ar and 1% impurity of N_2 at 313 K and pressures ranging from 20 bar to 200 bar.

P/ [bar]	c_V^{MC}/ [J/mol K]	c_V^{REFP}/ [J/mol K]	c_P^{MC}/ [J/mol K]	c_P^{REFP}/ [J/mol K]	c^{MC}/ [m/s]	c^{REFP}/ [m/s]	η^{MD}/ [μPa s]	η^{REFP}/ [μPa s]
20	31.1	31.1	43.3	43.7	264.6 ± 0.5	263.0	11.2 ± 7.5	16.0
40	32.9	33.4	52.1 ± 0.3	54.0	251.5 ± 1.4	248.3	16.9 ± 1.7	16.6
60	35.5 ± 0.1	36.7	71.1 ± 0.8	76.3	239.0 ± 2.7	231.8	18.3 ± 1.2	17.8
80	39.7 ± 0.5	–	144.5 ± 2.5	–	222.4 ± 2.6	–	21.2 ± 0.8	21.2
100	42.3 ± 0.8	46.7	379.7 ± 26.1	311.9	248.2 ± 16.7	237.6	36.5 ± 2.4	39.9
120	40.1 ± 0.2	42.5	177.3 ± 9.3	163.6	350.5 ± 24.9	323.4	61.7 ± 10.6	53.7
140	39.6 ± 0.2	41.1	130.9 ± 6.4	129.5	426.8 ± 22.9	381.9	65.5 ± 3.4	61.3
160	39.4 ± 0.2	40.5	118.7 ± 4.5	114.4	466.1 ± 22.6	426.2	72.2 ± 6.9	67.0
180	39.2 ± 0.1	40.1	111.4 ± 2.3	105.6	500.3 ± 13.8	462.7	73.1 ± 5.1	71.6
200	39.2 ± 0.2	39.9	102.6 ± 2.2	99.7	538.2 ± 9.7	494.3	75.5 ± 1.3	75.7

S16.2 Data of thermodynamics and transport properties of CO_2 rich ternary mixture with 1 mole% impurity of N_2 and 1 mole% impurity of H_2

Table S178: Densities computed from MC and MD simulations (ρ^{MC} and ρ^{MD}), densities obtained from REFPROP[10] (ρ^{REFP}), isothermal compressibilities computed from MC simulations (β_T^{MC}), isothermal compressibilities obtained from REFPROP[10] (β_T^{REFP}), thermal expansion coefficients computed from MC simulations (α_P^{MC}), thermal expansion coefficients obtained from REFPROP[10] (α_P^{REFP}), Joule Thomson coefficients computed from MC simulations ($\mu_{\text{JT}}^{\text{MC}}$), and Joule Thomson coefficients obtained from REFPROP[10] ($\mu_{\text{JT}}^{\text{REFP}}$) of CO_2 rich ternary mixture with 1% impurity of N_2 and 1% impurity of H_2 at 253 K and pressures ranging from 20 bar to 200 bar.

P/ [bar]	ρ^{MC}/ [kg/m^3]	ρ^{MD}/ [kg/m^3]	ρ^{REFP}/ [kg/m^3]	β_T^{MC}/ [10^{-5}/bar]	β_T^{REFP}/ [10^{-5}/bar]	α_P^{MC}/ [10^{-4}/K]	α_P^{REFP}/ [10^{-4}/K]	$\mu_{\text{JT}}^{\text{MC}}$/ [$10^{-3}$ K/bar]	$\mu_{\text{JT}}^{\text{REFP}}$/ [$10^{-3}$ K/bar]
20	50.0 ± 0.1	51.1 ± 0.2	51.6	6341.6 ± 74.5	6593.7	76.0 ± 1.1	84.1	1544.9 ± 48.6	1688.8
40	1011.5 ± 1.8	1008.7 ± 0.5	969.6	41.3 ± 1.4	75.4	46.3 ± 2.1	66.1	7.6 ± 2.4	–
60	1019.0 ± 1.0	1016.7 ± 0.8	1018.3	37.6 ± 1.1	42.8	43.2 ± 1.1	44.8	4.2 ± 1.3	6.2
80	1026.3 ± 0.4	1023.7 ± 1.3	1026.7	34.4 ± 1.3	39.3	40.6 ± 1.9	42.3	1.2 ± 2.2	3.3
100	1032.1 ± 1.5	1031.8 ± 0.9	1034.5	32.6 ± 1.1	36.4	39.5 ± 1.5	40.2	-0.1 ± 1.7	0.9
120	1038.8 ± 1.2	1038.3 ± 0.5	1041.8	30.9 ± 1.1	34.0	37.9 ± 1.6	38.4	-1.9 ± 1.8	-1.3
140	1046.2 ± 1.4	1045.8 ± 0.5	1048.7	29.4 ± 1.7	31.9	36.9 ± 1.9	36.9	-3.1 ± 2.3	-3.2
160	1052.3 ± 1.4	1050.5 ± 0.6	1055.2	27.2 ± 1.0	30.0	35.2 ± 1.5	35.5	-5.1 ± 1.8	-5.0
180	1057.5 ± 0.7	1056.5 ± 0.3	1061.4	26.9 ± 1.8	28.4	35.5 ± 2.4	34.3	-4.7 ± 2.8	-6.5
200	1063.5 ± 0.4	1061.7 ± 0.9	1067.3	24.3 ± 0.3	27.0	32.4 ± 0.3	33.2	-8.6 ± 0.4	-7.9

Table S179: Heat capacities at constant volume computed from MC simulations (c_V^{MC}), heat capacities at constant volume obtained from REFPROP[10] (c_V^{REFP}), heat capacities at constant pressure computed from MC simulations (c_P^{MC}), heat capacities at constant pressure obtained from REFPROP[10] (c_P^{REFP}), speed of sound computed from MC simulations (c^{MC}), speed of sound obtained from REFPROP[10] (c^{REFP}), viscosities computed from MD simulations (η^{MD}), and viscosities obtained from REFPROP[10] (η^{REFP}) of CO_2 rich ternary mixture with 1% impurity of N_2 and 1% impurity of H_2 at 253 K and pressures ranging from 20 bar to 200 bar.

P/ [bar]	c_V^{MC}/ [J/mol K]	c_V^{REFP}/ [J/mol K]	c_P^{MC}/ [J/mol K]	c_P^{REFP}/ [J/mol K]	c^{MC}/ [m/s]	c^{REFP}/ [m/s]	η^{MD}/ [μPa s]	η^{REFP}/ [μPa s]
20	31.8 ± 0.1	33.4	51.8 ± 0.4	56.2	226.8 ± 1.6	222.6	13.7 ± 1.5	13.0
40	40.4 ± 0.1	–	96.9 ± 3.3	–	757.7 ± 18.5	–	141.3 ± 6.2	116.9
60	40.7 ± 0.1	40.7	94.3 ± 1.3	91.3	778.4 ± 12.3	717.4	142.1 ± 6.4	136.4
80	40.5 ± 0.2	40.7	91.9 ± 3.2	89.5	802.2 ± 20.9	738.0	150.1 ± 2.8	140.2
100	40.6 ± 0.3	40.7	91.4 ± 2.2	87.9	817.9 ± 17.5	757.2	154.7 ± 7.5	143.8
120	40.6 ± 0.1	40.7	89.6 ± 2.2	86.6	828.7 ± 18.4	775.2	161.4 ± 8.7	147.4
140	40.6 ± 0.1	40.7	89.1 ± 2.4	85.5	844.6 ± 26.4	792.1	161.8 ± 15.5	150.8
160	40.7 ± 0.2	40.8	88.1 ± 2.3	84.4	870.5 ± 20.1	808.2	154.6 ± 5.1	154.1
180	40.8 ± 0.2	40.8	89.2 ± 3.6	83.5	876.2 ± 34.9	823.5	177.0 ± 11.4	157.4
200	40.9 ± 0.2	40.8	85.3 ± 0.6	82.7	897.8 ± 6.9	838.1	176.4 ± 20.2	160.6

Table S180: Densities computed from MC and MD simulations (ρ^{MC} and ρ^{MD}), densities obtained from REFPROP[10] (ρ^{REFP}), isothermal compressibilities computed from MC simulations (β_T^{MC}), isothermal compressibilities obtained from REFPROP[10] (β_T^{REFP}), thermal expansion coefficients computed from MC simulations (α_P^{MC}), thermal expansion coefficients obtained from REFPROP[10] (α_P^{REFP}), Joule Thomson coefficients computed from MC simulations (μ_{JT}^{MC}), and Joule Thomson coefficients obtained from REFPROP[10] (μ_{JT}^{REFP}) of CO_2 rich ternary mixture with 1% impurity of N_2 and 1% impurity of H_2 at 273 K and pressures ranging from 20 bar to 200 bar.

P/ [bar]	ρ^{MC}/ [kg/m^3]	ρ^{MD}/ [kg/m^3]	ρ^{REFP}/ [kg/m^3]	β_T^{MC}/ [10^{-5}/bar]	β_T^{REFP}/ [10^{-5}/bar]	α_P^{MC}/ [10^{-4}/K]	α_P^{REFP}/ [10^{-4}/K]	μ_{JT}^{MC}/ [10^{-3} K/bar]	μ_{JT}^{REFP}/ [10^{-3} K/bar]
20	43.9	44.9 ± 0.2	44.8	5850.8 ± 39.1	5973.9	57.9 ± 0.4	61.1	1240.2 ± 26.1	1358.0
40	–	117.3 ± 1.0	415.0	–	1290.7	–	605.5	–	–
60	915.6 ± 1.9	913.3 ± 1.1	912.3	81.0 ± 5.6	96.1	65.9 ± 4.6	69.9	34.5 ± 5.6	40.5
80	930.6 ± 1.3	926.5 ± 0.9	928.4	69.3 ± 4.4	79.5	58.9 ± 3.5	61.3	27.2 ± 4.4	31.3
100	941.9 ± 1.2	940.5 ± 1.3	942.1	58.9 ± 2.3	68.3	52.7 ± 1.8	55.3	20.3 ± 2.4	24.3
120	952.7 ± 1.1	951.1 ± 0.8	954.3	55.2 ± 4.2	60.1	50.7 ± 3.6	50.8	17.8 ± 4.6	18.8
140	964.4 ± 1.7	961.6 ± 0.4	965.2	46.9 ± 1.2	53.8	45.0 ± 1.0	47.2	11.0 ± 1.4	14.2
160	972.8 ± 0.9	970.3 ± 1.2	975.1	45.0 ± 1.9	48.8	44.2 ± 1.9	44.3	9.9 ± 2.5	10.4
180	980.7 ± 0.9	979.6 ± 1.3	984.2	41.7 ± 1.0	44.7	42.3 ± 0.9	41.8	7.5 ± 1.2	7.2
200	988.3 ± 1.5	986.6 ± 0.9	992.7	38.7 ± 1.3	41.3	40.3 ± 1.3	39.7	4.8 ± 1.7	4.3

Table S181: Heat capacities at constant volume computed from MC simulations (c_V^{MC}), heat capacities at constant volume obtained from REFPROP[10] (c_V^{REFP}), heat capacities at constant pressure computed from MC simulations (c_P^{MC}), heat capacities at constant pressure obtained from REFPROP[10] (c_P^{REFP}), speed of sound computed from MC simulations (c^{MC}), speed of sound obtained from REFPROP[10] (c^{REFP}), viscosities computed from MD simulations (η^{MD}), and viscosities obtained from REFPROP[10] (η^{REFP}) of CO_2 rich ternary mixture with 1% impurity of N_2 and 1% impurity of H_2 at 273 K and pressures ranging from 20 bar to 200 bar.

P/ [bar]	c_V^{MC}/ [J/mol K]	c_V^{REFP}/ [J/mol K]	c_P^{MC}/ [J/mol K]	c_P^{REFP}/ [J/mol K]	c^{MC}/ [m/s]	c^{REFP}/ [m/s]	η^{MD}/ [μPa s]	η^{REFP}/ [μPa s]
20	30.9	31.2	46.3 ± 0.1	47.8	241.8 ± 0.9	239.3	3.2 ± 2.7	14.0
40	–	–	–	–	–	–	15.2 ± 1.2	27.1
60	40.2 ± 0.3	40.7	109.8 ± 5.1	106.7	606.8 ± 25.3	546.8	104.5 ± 1.7	97.4
80	39.9 ± 0.1	40.5	104.2 ± 3.9	100.9	636.3 ± 23.6	581.1	107.8 ± 1.7	102.0
100	39.8 ± 0.2	40.3	99.2 ± 2.1	96.7	670.5 ± 14.7	610.6	112.5 ± 4.4	106.2
120	39.8 ± 0.2	40.3	98.0 ± 4.0	93.6	683.8 ± 29.7	636.7	112.5 ± 7.2	110.0
140	39.7 ± 0.2	40.2	93.1 ± 1.2	91.1	720.2 ± 10.7	660.4	120.4 ± 6.2	113.7
160	39.9 ± 0.2	40.2	92.9 ± 2.3	89.0	730.2 ± 18.0	682.2	127.4 ± 7.2	117.1
180	39.9 ± 0.1	40.2	91.8 ± 1.0	87.3	750.7 ± 10.2	702.4	121.4 ± 11.1	120.4
200	39.8 ± 0.1	40.2	90.4 ± 1.6	85.8	770.6 ± 15.0	721.3	134.2 ± 15.1	123.6

Table S182: Densities computed from MC and MD simulations (ρ^{MC} and ρ^{MD}), densities obtained from REFPROP[10] (ρ^{REFP}), isothermal compressibilities computed from MC simulations (β_T^{MC}), isothermal compressibilities obtained from REFPROP[10] (β_T^{REFP}), thermal expansion coefficients computed from MC simulations (α_P^{MC}), thermal expansion coefficients obtained from REFPROP[10] (α_P^{REFP}), Joule Thomson coefficients computed from MC simulations (μ_{JT}^{MC}), and Joule Thomson coefficients obtained from REFPROP[10] (μ_{JT}^{REFP}) of CO_2 rich ternary mixture with 1% impurity of N_2 and 1% impurity of H_2 at 293 K and pressures ranging from 20 bar to 200 bar.

P/ [bar]	ρ^{MC}/ [kg/m^3]	ρ^{MD}/ [kg/m^3]	ρ^{REFP}/ [kg/m^3]	β_T^{MC}/ [10^{-5}/bar]	β_T^{REFP}/ [10^{-5}/bar]	α_P^{MC}/ [10^{-4}/K]	α_P^{REFP}/ [10^{-4}/K]	μ_{JT}^{MC}/ [10^{-3} K/bar]	μ_{JT}^{REFP}/ [10^{-3} K/bar]
20	39.5	40.2	40.1	5623.7 ± 24.7	5679.5	48.4 ± 0.2	50.0	1041.2 ± 15.2	1123.1
40	91.5 ± 0.1	93.9 ± 0.4	94.8	3409.4 ± 35.8	3564.1	78.6 ± 1.1	87.0	1046.7 ± 27.4	1120.7
60	–	241.1 ± 50.2	238.4	–	8962.6	–	759.0	–	–
80	786.2 ± 4.0	777.7 ± 6.4	778.0	262.6 ± 34.1	306.3	133.7 ± 14.5	139.8	105.7 ± 17.1	117.9
100	819.4 ± 1.2	815.9 ± 2.1	815.5	157.9 ± 9.4	186.3	92.4 ± 4.3	98.1	72.6 ± 5.7	82.0
120	841.8 ± 3.3	841.0 ± 1.6	841.8	121.3 ± 3.9	136.6	77.5 ± 2.7	79.2	56.9 ± 3.8	62.0
140	861.6 ± 2.8	859.6 ± 2.2	862.6	94.3 ± 5.4	108.9	64.5 ± 3.3	67.9	42.6 ± 4.8	48.5
160	877.7 ± 2.1	875.6 ± 1.5	879.9	83.0 ± 4.9	90.9	59.8 ± 3.1	60.2	36.4 ± 4.5	38.6
180	891.2 ± 2.0	889.8 ± 1.9	894.8	72.8 ± 2.9	78.3	54.9 ± 1.7	54.6	30.0 ± 2.6	30.9
200	903.7 ± 1.7	901.9 ± 1.6	908.1	64.6 ± 2.1	69.0	51.0 ± 1.5	50.2	24.6 ± 2.2	24.8

Table S183: Heat capacities at constant volume computed from MC simulations (c_V^{MC}), heat capacities at constant volume obtained from REFPROP[10] (c_V^{REFP}), heat capacities at constant pressure computed from MC simulations (c_P^{MC}), heat capacities at constant pressure obtained from REFPROP[10] (c_P^{REFP}), speed of sound computed from MC simulations (c^{MC}), speed of sound obtained from REFPROP[10] (c^{REFP}), viscosities computed from MD simulations (η^{MD}), and viscosities obtained from REFPROP[10] (η^{REFP}) of CO_2 rich ternary mixture with 1% impurity of N_2 and 1% impurity of H_2 at 293 K and pressures ranging from 20 bar to 200 bar.

P/ [bar]	c_V^{MC}/ [J/mol K]	c_V^{REFP}/ [J/mol K]	c_P^{MC}/ [J/mol K]	c_P^{REFP}/ [J/mol K]	c^{MC}/ [m/s]	c^{REFP}/ [m/s]	η^{MD}/ [μPa s]	η^{REFP}/ [μPa s]
20	30.8	31.0	44.3 ± 0.1	45.0	254.3 ± 0.6	252.5	6.8 ± 3.4	15.0
40	33.9	34.8	59.1 ± 0.4	63.3	236.6 ± 1.5	232.0	16.9 ± 1.1	15.7
60	–	–	–	–	–	–	18.3 ± 1.3	19.4
80	40.2 ± 0.3	42.2	152.5 ± 10.7	146.6	428.7 ± 31.7	381.8	69.6 ± 1.5	68.1
100	39.8 ± 0.2	41.1	124.7 ± 3.1	121.6	492.1 ± 15.9	441.5	83.2 ± 6.9	74.9
120	39.6 ± 0.1	40.5	115.2 ± 2.8	109.9	533.8 ± 10.7	485.5	92.2 ± 6.9	80.2
140	39.5 ± 0.1	40.2	105.3 ± 3.0	102.7	572.9 ± 18.3	521.3	89.1 ± 4.9	84.7
160	39.5 ± 0.2	40.1	102.2 ± 2.9	97.7	596.1 ± 19.5	552.2	99.8 ± 10.9	88.8
180	39.5 ± 0.1	40.0	98.6 ± 1.7	94.1	620.1 ± 13.4	579.5	95.2 ± 3.3	92.4
200	39.3 ± 0.1	39.9	96.5 ± 1.6	91.2	648.5 ± 11.6	604.2	103.8 ± 6.7	95.8

Table S184: Densities computed from MC and MD simulations (ρ^{MC} and ρ^{MD}), densities obtained from REFPROP[10] (ρ^{REFP}), isothermal compressibilities computed from MC simulations (β_T^{MC}), isothermal compressibilities obtained from REFPROP[10] (β_T^{REFP}), thermal expansion coefficients computed from MC simulations (α_P^{MC}), thermal expansion coefficients obtained from REFPROP[10] (α_P^{REFP}), Joule Thomson coefficients computed from MC simulations (μ_{JT}^{MC}), and Joule Thomson coefficients obtained from REFPROP[10] (μ_{JT}^{REFP}) of CO_2 rich ternary mixture with 1% impurity of N_2 and 1% impurity of H_2 at 313 K and pressures ranging from 20 bar to 200 bar.

P/ [bar]	ρ^{MC}/ [kg/m^3]	ρ^{MD}/ [kg/m^3]	ρ^{REFP}/ [kg/m^3]	β_T^{MC}/ [10^{-5}/bar]	β_T^{REFP}/ [10^{-5}/bar]	α_P^{MC}/ [10^{-4}/K]	α_P^{REFP}/ [10^{-4}/K]	μ_{JT}^{MC}/ [10^{-3} K/bar]	μ_{JT}^{REFP}/ [10^{-3} K/bar]
20	36.1	36.7 ± 0.1	36.5	5431.2 ± 18.4	5503.0	41.8 ± 0.2	43.1	856.1 ± 13.6	946.0
40	80.0 ± 0.1	81.5 ± 0.3	82.0	3090.9 ± 31.2	3158.5	59.0 ± 0.6	62.4	881.0 ± 21.1	936.8
60	137.7 ± 0.2	141.6 ± 0.5	144.1	2512.6 ± 25.9	2639.2	94.2 ± 0.6	104.6	870.3 ± 9.3	904.2
80	229.6 ± 0.9	248.3 ± 2.2	252.1	2813.3 ± 78.8	3211.0	208.1 ± 6.5	262.4	792.3 ± 37.2	–
100	510.2 ± 15.3	507.1 ± 5.1	531.3	2823.5 ± 322.5	2318.2	588.3 ± 56.3	495.1	389.7 ± 54.0	373.6
120	661.2 ± 3.3	659.5 ± 2.6	664.3	592.1 ± 63.6	571.9	202.2 ± 16.9	185.5	188.8 ± 21.2	189.7
140	719.8 ± 2.0	714.3 ± 2.4	721.2	290.0 ± 21.4	304.7	122.4 ± 8.6	119.9	123.3 ± 13.2	127.4
160	753.2 ± 0.9	751.4 ± 0.8	758.2	210.9 ± 14.3	207.5	100.4 ± 6.0	92.6	97.6 ± 9.3	94.9
180	782.1 ± 1.7	778.2 ± 2.1	786.0	143.8 ± 8.0	157.5	76.0 ± 3.7	77.2	69.8 ± 6.1	74.1
200	803.3 ± 2.0	801.2 ± 0.5	808.5	120.4 ± 4.1	127.1	68.2 ± 1.9	67.2	58.4 ± 3.3	59.4

Table S185: Heat capacities at constant volume computed from MC simulations (c_V^{MC}), heat capacities at constant volume obtained from REFPROP[10] (c_V^{REFP}), heat capacities at constant pressure computed from MC simulations (c_P^{MC}), heat capacities at constant pressure obtained from REFPROP[10] (c_P^{REFP}), speed of sound computed from MC simulations (c^{MC}), speed of sound obtained from REFPROP[10] (c^{REFP}), viscosities computed from MD simulations (η^{MD}), and viscosities obtained from REFPROP[10] (η^{REFP}) of CO_2 rich ternary mixture with 1% impurity of N_2 and 1% impurity of H_2 at 313 K and pressures ranging from 20 bar to 200 bar.

P/ [bar]	c_V^{MC}/ [J/mol K]	c_V^{REFP}/ [J/mol K]	c_P^{MC}/ [J/mol K]	c_P^{REFP}/ [J/mol K]	c^{MC}/ [m/s]	c^{REFP}/ [m/s]	η^{MD}/ [μPa s]	η^{REFP}/ [μPa s]
20	31.1	31.2	43.2 ± 0.1	43.7	266.1 ± 0.5	264.1	12.6 ± 3.6	15.9
40	33.0	33.5	52.1 ± 0.2	53.9	252.8 ± 1.4	249.5	16.7 ± 1.7	16.5
60	35.6 ± 0.1	36.7	70.6 ± 0.4	75.8	239.4 ± 1.4	233.1	18.9 ± 1.5	17.7
80	39.7 ± 0.6	–	131.6 ± 3.8	–	226.6 ± 4.8	–	20.9 ± 0.8	21.0
100	42.7 ± 0.7	46.7	381.0 ± 34.3	317.2	249.2 ± 18.6	234.8	34.7 ± 3.0	38.1
120	40.5 ± 0.3	42.6	185.4 ± 9.7	165.7	341.8 ± 20.5	320.1	52.1 ± 4.0	52.1
140	39.9 ± 0.2	41.2	138.5 ± 7.0	130.1	407.7 ± 18.3	379.2	62.1 ± 6.1	59.7
160	39.5 ± 0.1	40.5	126.5 ± 4.7	114.6	448.8 ± 17.4	424.1	71.1 ± 8.1	65.3
180	39.4	40.1	109.8 ± 3.0	105.6	497.8 ± 15.4	461.0	73.8 ± 9.7	69.9
200	39.3 ± 0.2	39.9	105.0 ± 1.7	99.6	525.2 ± 9.9	492.8	78.6 ± 2.4	74.0

S16.3 Data of thermodynamics and transport properties of CO_2 rich ternary mixture with 1 mole% impurity of H_2 and 1 mole% impurity of CH_4

Table S186: Densities computed from MC and MD simulations (ρ^{MC} and ρ^{MD}), densities obtained from REFPROP[10] (ρ^{REFP}), isothermal compressibilities computed from MC simulations (β_T^{MC}), isothermal compressibilities obtained from REFPROP[10] (β_T^{REFP}), thermal expansion coefficients computed from MC simulations (α_P^{MC}), thermal expansion coefficients obtained from REFPROP[10] (α_P^{REFP}), Joule Thomson coefficients computed from MC simulations ($\mu_{\mathrm{JT}}^{\mathrm{MC}}$), and Joule Thomson coefficients obtained from REFPROP[10] ($\mu_{\mathrm{JT}}^{\mathrm{REFP}}$) of CO_2 rich ternary mixture with 1% impurity of H_2 and 1% impurity of CH_4 at 253 K and pressures ranging from 20 bar to 200 bar.

P/ [bar]	ρ^{MC}/ [kg/m^3]	ρ^{MD}/ [kg/m^3]	ρ^{REFP}/ [kg/m^3]	β_T^{MC}/ [10^{-5}/bar]	β_T^{REFP}/ [10^{-5}/bar]	α_P^{MC}/ [10^{-4}/K]	α_P^{REFP}/ [10^{-4}/K]	$\mu_{\mathrm{JT}}^{\mathrm{MC}}$/ [$10^{-3}$ K/bar]	$\mu_{\mathrm{JT}}^{\mathrm{REFP}}$/ [$10^{-3}$ K/bar]
20	50.0	51.1 ± 0.1	51.5	6344.8 ± 40.7	6609.1	76.2 ± 0.6	84.5	1545.2 ± 28.2	1694.0
40	1010.6 ± 1.3	1007.8 ± 0.6	1007.0	38.7 ± 1.6	46.2	43.8 ± 1.5	47.2	4.9 ± 1.7	9.0
60	1018.2 ± 1.6	1015.4 ± 0.8	1015.9	36.7 ± 1.7	42.0	42.8 ± 2.4	44.3	3.8 ± 2.7	5.7
80	1025.6 ± 1.3	1022.5 ± 1.4	1024.2	34.6 ± 1.4	38.7	41.4 ± 1.5	41.9	2.1 ± 1.7	2.9
100	1031.5 ± 1.5	1030.2 ± 0.4	1031.8	31.6 ± 1.0	35.9	38.6 ± 1.3	39.9	-1.1 ± 1.5	0.5
120	1037.9 ± 1.1	1037.6 ± 0.4	1039.0	29.8 ± 0.5	33.5	37.3 ± 0.6	38.2	-2.6 ± 0.7	-1.7
140	1043.8 ± 0.7	1043.1 ± 1.0	1045.8	28.5 ± 0.5	31.5	35.9 ± 0.6	36.6	-4.3 ± 0.7	-3.6
160	1050.5 ± 1.4	1049.3 ± 0.6	1052.2	26.3 ± 1.2	29.7	34.2 ± 1.7	35.3	-6.3 ± 2.0	-5.3
180	1056.4 ± 0.9	1054.8 ± 0.4	1058.3	26.2 ± 0.9	28.1	34.5 ± 1.5	34.1	-6.0 ± 1.7	-6.8
200	1061.6 ± 0.5	1059.7 ± 0.6	1064.1	25.0 ± 1.2	26.7	33.5 ± 1.6	33.0	-7.1 ± 2.0	-8.2

Table S187: Heat capacities at constant volume computed from MC simulations (c_V^{MC}), heat capacities at constant volume obtained from REFPROP[10] (c_V^{REFP}), heat capacities at constant pressure computed from MC simulations (c_P^{MC}), heat capacities at constant pressure obtained from REFPROP[10] (c_P^{REFP}), speed of sound computed from MC simulations (c^{MC}), speed of sound obtained from REFPROP[10] (c^{REFP}), viscosities computed from MD simulations (η^{MD}), and viscosities obtained from REFPROP[10] (η^{REFP}) of CO_2 rich ternary mixture with 1% impurity of H_2 and 1% impurity of CH_4 at 253 K and pressures ranging from 20 bar to 200 bar.

P/ [bar]	c_V^{MC}/ [J/mol K]	c_V^{REFP}/ [J/mol K]	c_P^{MC}/ [J/mol K]	c_P^{REFP}/ [J/mol K]	c^{MC}/ [m/s]	c^{REFP}/ [m/s]	η^{MD}/ [μPa s]	η^{REFP}/ [μPa s]
20	31.9 ± 0.1	33.5	52.0 ± 0.2	56.5	226.7 ± 0.9	222.6	11.8 ± 3.1	12.9
40	40.5 ± 0.2	40.7	94.4 ± 1.5	93.2	772.0 ± 16.8	702.2	143.9 ± 2.0	132.7
60	40.5 ± 0.1	40.6	94.4 ± 3.6	91.0	790.4 ± 24.2	724.3	147.0 ± 3.9	136.6
80	40.5 ± 0.2	40.6	93.4 ± 1.9	89.3	805.5 ± 18.1	744.7	146.8 ± 2.9	140.3
100	40.5 ± 0.2	40.7	90.6 ± 1.8	87.8	828.6 ± 16.0	763.7	158.4 ± 9.9	144.0
120	40.8 ± 0.2	40.7	89.9 ± 0.9	86.5	843.8 ± 8.6	781.5	170.2 ± 10.7	147.5
140	40.6 ± 0.1	40.7	88.3 ± 1.1	85.4	855.6 ± 9.4	798.3	179.5 ± 31.6	150.9
160	40.7 ± 0.1	40.7	87.2 ± 2.5	84.4	881.3 ± 24.1	814.2	164.0 ± 17.7	154.2
180	40.9 ± 0.2	40.8	87.8 ± 2.6	83.5	880.3 ± 20.4	829.4	170.6 ± 20.1	157.4
200	40.9 ± 0.2	40.8	87.2 ± 2.3	82.7	897.2 ± 24.7	843.9	175.7 ± 12.0	160.6

Table S188: Densities computed from MC and MD simulations (ρ^{MC} and ρ^{MD}), densities obtained from REFPROP[10] (ρ^{REFP}), isothermal compressibilities computed from MC simulations (β_T^{MC}), isothermal compressibilities obtained from REFPROP[10] (β_T^{REFP}), thermal expansion coefficients computed from MC simulations (α_P^{MC}), thermal expansion coefficients obtained from REFPROP[10] (α_P^{REFP}), Joule Thomson coefficients computed from MC simulations (μ_{JT}^{MC}), and Joule Thomson coefficients obtained from REFPROP[10] (μ_{JT}^{REFP}) of CO_2 rich ternary mixture with 1% impurity of H_2 and 1% impurity of CH_4 at 273 K and pressures ranging from 20 bar to 200 bar.

P/ [bar]	ρ^{MC}/ [kg/m^3]	ρ^{MD}/ [kg/m^3]	ρ^{REFP}/ [kg/m^3]	β_T^{MC}/ [10^{-5}/bar]	β_T^{REFP}/ [10^{-5}/bar]	α_P^{MC}/ [10^{-4}/K]	α_P^{REFP}/ [10^{-4}/K]	μ_{JT}^{MC}/ [10^{-3} K/bar]	μ_{JT}^{REFP}/ [10^{-3} K/bar]
20	–	44.6 ± 0.1	44.7	–	5980.0	–	61.3	–	1360.6
40	–	116.9 ± 0.8	471.1	–	257.8	–	-7.4	–	–
60	915.7 ± 1.2	913.5 ± 2.0	911.6	76.6 ± 2.2	93.1	62.9 ± 2.0	68.5	31.7 ± 2.5	39.0
80	931.1 ± 1.5	928.3 ± 1.8	927.2	66.5 ± 5.0	77.5	57.8 ± 3.7	60.3	25.9 ± 4.6	30.1
100	941.9 ± 1.6	940.4 ± 1.4	940.6	58.7 ± 2.6	66.8	52.3 ± 2.0	54.6	20.0 ± 2.5	23.4
120	953.6 ± 1.4	951.5 ± 1.0	952.5	51.4 ± 1.2	58.9	48.3 ± 1.1	50.2	15.0 ± 1.5	18.0
140	963.3 ± 1.7	960.8 ± 1.1	963.1	47.5 ± 1.1	52.9	46.0 ± 0.7	46.7	12.1 ± 0.9	13.6
160	971.3 ± 1.1	969.3 ± 0.7	972.9	43.7 ± 1.8	48.0	43.4 ± 2.0	43.9	8.9 ± 2.6	9.9
180	978.7 ± 1.4	978.2 ± 0.8	981.9	41.1 ± 1.3	44.1	41.8 ± 1.8	41.5	6.8 ± 2.3	6.7
200	987.6 ± 1.0	985.9 ± 0.9	990.2	37.9 ± 0.8	40.8	39.9 ± 0.8	39.4	4.3 ± 1.0	3.9

Table S189: Heat capacities at constant volume computed from MC simulations (c_V^{MC}), heat capacities at constant volume obtained from REFPROP[10] (c_V^{REFP}), heat capacities at constant pressure computed from MC simulations (c_P^{MC}), heat capacities at constant pressure obtained from REFPROP[10] (c_P^{REFP}), speed of sound computed from MC simulations (c^{MC}), speed of sound obtained from REFPROP[10] (c^{REFP}), viscosities computed from MD simulations (η^{MD}), and viscosities obtained from REFPROP[10] (η^{REFP}) of CO_2 rich ternary mixture with 1% impurity of H_2 and 1% impurity of CH_4 at 273 K and pressures ranging from 20 bar to 200 bar.

P/ [bar]	c_V^{MC}/ [J/mol K]	c_V^{REFP}/ [J/mol K]	c_P^{MC}/ [J/mol K]	c_P^{REFP}/ [J/mol K]	c^{MC}/ [m/s]	c^{REFP}/ [m/s]	η^{MD}/ [μPa s]	η^{REFP}/ [μPa s]
20	–	31.3	–	47.9	–	239.4	6.0 ± 4.4	13.9
40	–	–	–	–	–	–	14.5 ± 0.8	31.3
60	39.9 ± 0.1	40.6	107.0 ± 2.6	105.9	618.5 ± 11.8	554.3	103.4 ± 1.8	97.8
80	39.8 ± 0.1	40.4	104.1 ± 3.5	100.3	650.5 ± 26.8	587.9	119.4 ± 22.7	102.3
100	39.9 ± 0.2	40.3	98.7 ± 2.2	96.3	669.5 ± 16.6	617.0	112.4 ± 2.5	106.4
120	39.9 ± 0.1	40.2	96.4 ± 1.2	93.3	702.5 ± 9.6	642.9	117.7 ± 11.3	110.3
140	39.9 ± 0.1	40.2	94.7 ± 0.8	90.8	721.0 ± 8.9	666.3	128.4 ± 12.5	113.8
160	40.0 ± 0.3	40.2	92.7 ± 2.6	88.8	738.5 ± 18.5	687.9	130.4 ± 6.5	117.3
180	40.2 ± 0.2	40.2	91.4 ± 2.7	87.1	752.0 ± 16.4	707.9	129.1 ± 16.2	120.5
200	39.9 ± 0.1	40.2	90.5 ± 1.2	85.7	778.7 ± 10.0	726.6	136.1 ± 16.4	123.7

Table S190: Densities computed from MC and MD simulations (ρ^{MC} and ρ^{MD}), densities obtained from REFPROP[10] (ρ^{REFP}), isothermal compressibilities computed from MC simulations (β_T^{MC}), isothermal compressibilities obtained from REFPROP[10] (β_T^{REFP}), thermal expansion coefficients computed from MC simulations (α_P^{MC}), thermal expansion coefficients obtained from REFPROP[10] (α_P^{REFP}), Joule Thomson coefficients computed from MC simulations ($\mu_{\mathrm{JT}}^{\mathrm{MC}}$), and Joule Thomson coefficients obtained from REFPROP[10] ($\mu_{\mathrm{JT}}^{\mathrm{REFP}}$) of CO_2 rich ternary mixture with 1% impurity of H_2 and 1% impurity of CH_4 at 293 K and pressures ranging from 20 bar to 200 bar.

P/ [bar]	ρ^{MC}/ [kg/m^3]	ρ^{MD}/ [kg/m^3]	ρ^{REFP}/ [kg/m^3]	β_T^{MC}/ [10^{-5}/bar]	β_T^{REFP}/ [10^{-5}/bar]	α_P^{MC}/ [10^{-4}/K]	α_P^{REFP}/ [10^{-4}/K]	$\mu_{\mathrm{JT}}^{\mathrm{MC}}$/ [$10^{-3}$ K/bar]	$\mu_{\mathrm{JT}}^{\mathrm{REFP}}$/ [$10^{-3}$ K/bar]
20	39.4	40.2 ± 0.1	40.0	5607.0 ± 34.4	5683.2	48.3 ± 0.3	50.1	1028.7 ± 25.6	1124.5
40	91.4 ± 0.1	93.6 ± 0.2	94.7	3430.9 ± 34.9	3575.6	79.5 ± 0.8	87.5	1057.8 ± 19.9	1124.3
60	–	345.4 ± 124.3	260.7	–	17 879.4	–	1739.4	–	–
80	792.4 ± 2.4	784.3 ± 2.7	781.0	228.3 ± 11.2	285.1	120.7 ± 5.9	133.1	96.3 ± 7.5	112.5
100	822.4 ± 2.0	820.6 ± 1.6	816.6	153.7 ± 7.3	178.2	91.6 ± 3.7	95.3	71.0 ± 4.9	79.1
120	846.8 ± 1.3	842.3 ± 2.0	841.9	113.1 ± 8.0	132.2	73.8 ± 4.0	77.5	52.9 ± 5.6	60.0
140	862.3 ± 1.8	859.6 ± 1.6	862.0	96.1 ± 7.7	106.0	66.6 ± 5.0	66.8	44.3 ± 7.1	47.1
160	876.8 ± 2.4	875.8 ± 1.4	878.9	83.2 ± 3.0	88.9	60.2 ± 2.1	59.4	36.6 ± 3.0	37.5
180	890.7 ± 0.5	890.0 ± 1.2	893.5	71.4 ± 1.8	76.8	54.6 ± 1.2	54.0	29.4 ± 1.7	30.0
200	902.5 ± 1.2	901.3 ± 1.6	906.5	62.0 ± 2.7	67.8	49.3 ± 2.3	49.7	22.5 ± 3.5	24.0

Table S191: Heat capacities at constant volume computed from MC simulations (c_V^{MC}), heat capacities at constant volume obtained from REFPROP[10] (c_V^{REFP}), heat capacities at constant pressure computed from MC simulations (c_P^{MC}), heat capacities at constant pressure obtained from REFPROP[10] (c_P^{REFP}), speed of sound computed from MC simulations (c^{MC}), speed of sound obtained from REFPROP[10] (c^{REFP}), viscosities computed from MD simulations (η^{MD}), and viscosities obtained from REFPROP[10] (η^{REFP}) of CO_2 rich ternary mixture with 1% impurity of H_2 and 1% impurity of CH_4 at 293 K and pressures ranging from 20 bar to 200 bar.

P/ [bar]	c_V^{MC}/ [J/mol K]	c_V^{REFP}/ [J/mol K]	c_P^{MC}/ [J/mol K]	c_P^{REFP}/ [J/mol K]	c^{MC}/ [m/s]	c^{REFP}/ [m/s]	η^{MD}/ [μPa s]	η^{REFP}/ [μPa s]
20	30.9	31.0	44.3 ± 0.1	45.0	254.7 ± 0.9	252.7	8.5 ± 1.1	14.9
40	34.0 ± 0.1	34.9	59.6 ± 0.3	63.6	236.6 ± 1.4	231.9	14.9 ± 0.3	15.6
60	–	–	–	–	–	–	20.2 ± 2.6	20.2
80	40.2 ± 0.1	42.0	144.0 ± 5.4	143.0	444.7 ± 13.7	391.0	71.3 ± 0.4	68.8
100	39.8 ± 0.2	41.0	124.9 ± 3.1	120.1	498.5 ± 13.4	448.9	85.6 ± 7.2	75.4
120	39.5 ± 0.2	40.5	112.4 ± 3.1	109.0	545.1 ± 20.7	491.9	84.9 ± 7.7	80.6
140	39.5 ± 0.2	40.2	107.9 ± 5.0	102.2	574.2 ± 26.5	527.3	89.5 ± 2.9	85.0
160	39.4 ± 0.1	40.0	103.2 ± 2.3	97.4	599.0 ± 12.7	557.8	92.5 ± 4.6	89.0
180	39.5 ± 0.2	39.9	99.0 ± 1.1	93.8	627.7 ± 8.7	584.8	97.8 ± 7.7	92.6
200	39.6 ± 0.1	39.9	94.7 ± 2.8	91.0	653.2 ± 17.2	609.3	103.7 ± 4.8	96.0

Table S192: Densities computed from MC and MD simulations (ρ^{MC} and ρ^{MD}), densities obtained from REFPROP[10] (ρ^{REFP}), isothermal compressibilities computed from MC simulations (β_T^{MC}), isothermal compressibilities obtained from REFPROP[10] (β_T^{REFP}), thermal expansion coefficients computed from MC simulations (α_P^{MC}), thermal expansion coefficients obtained from REFPROP[10] (α_P^{REFP}), Joule Thomson coefficients computed from MC simulations ($\mu_{\mathrm{JT}}^{\mathrm{MC}}$), and Joule Thomson coefficients obtained from REFPROP[10] ($\mu_{\mathrm{JT}}^{\mathrm{REFP}}$) of CO_2 rich ternary mixture with 1% impurity of H_2 and 1% impurity of CH_4 at 313 K and pressures ranging from 20 bar to 200 bar.

P/ [bar]	ρ^{MC}/ [kg/m^3]	ρ^{MD}/ [kg/m^3]	ρ^{REFP}/ [kg/m^3]	β_T^{MC}/ [10^{-5}/bar]	β_T^{REFP}/ [10^{-5}/bar]	α_P^{MC}/ [10^{-4}/K]	α_P^{REFP}/ [10^{-4}/K]	$\mu_{\mathrm{JT}}^{\mathrm{MC}}$/ [$10^{-3}$ K/bar]	$\mu_{\mathrm{JT}}^{\mathrm{REFP}}$/ [$10^{-3}$ K/bar]
20	36.0	36.7 ± 0.1	36.5	5471.9 ± 25.1	5505.7	42.1 ± 0.2	43.1	877.6 ± 20.5	946.7
40	79.8 ± 0.1	81.4 ± 0.2	81.8	3087.2 ± 34.5	3164.0	59.0 ± 0.7	62.6	879.7 ± 22.7	939.3
60	137.7 ± 0.2	142.0 ± 0.4	144.1	2508.7 ± 36.0	2652.6	93.8 ± 1.9	105.5	866.4 ± 28.2	907.2
80	231.3 ± 2.2	249.0 ± 1.6	253.3	2960.4 ± 155.0	3281.8	221.8 ± 13.8	270.7	807.0 ± 70.9	787.5
100	535.9 ± 11.8	523.5 ± 7.9	544.2	2838.1 ± 329.9	2138.8	628.0 ± 51.7	476.4	370.1 ± 39.8	356.2
120	674.7 ± 7.1	664.5 ± 3.4	669.1	473.1 ± 53.4	537.8	173.2 ± 15.8	178.6	167.3 ± 21.5	182.8
140	723.9 ± 1.1	720.9 ± 2.6	723.5	283.9 ± 23.9	292.4	122.4 ± 9.3	117.0	121.2 ± 13.8	123.7
160	756.6 ± 1.8	753.8 ± 1.2	759.2	186.9 ± 17.2	201.1	90.3 ± 7.5	90.9	88.2 ± 12.2	92.5
180	784.1 ± 1.4	781.0 ± 1.8	786.3	153.2 ± 5.7	153.5	81.6 ± 2.9	76.1	74.6 ± 4.7	72.4
200	804.2 ± 2.2	801.3 ± 0.7	808.2	120.9 ± 3.8	124.3	69.1 ± 1.5	66.3	58.9 ± 2.5	58.1

Table S193: Heat capacities at constant volume computed from MC simulations (c_V^{MC}), heat capacities at constant volume obtained from REFPROP[10] (c_V^{REFP}), heat capacities at constant pressure computed from MC simulations (c_P^{MC}), heat capacities at constant pressure obtained from REFPROP[10] (c_P^{REFP}), speed of sound computed from MC simulations (c^{MC}), speed of sound obtained from REFPROP[10] (c^{REFP}), viscosities computed from MD simulations (η^{MD}), and viscosities obtained from REFPROP[10] (η^{REFP}) of CO_2 rich ternary mixture with 1% impurity of H_2 and 1% impurity of CH_4 at 313 K and pressures ranging from 20 bar to 200 bar.

P/ [bar]	c_V^{MC}/ [J/mol K]	c_V^{REFP}/ [J/mol K]	c_P^{MC}/ [J/mol K]	c_P^{REFP}/ [J/mol K]	c^{MC}/ [m/s]	c^{REFP}/ [m/s]	η^{MD}/ [μPa s]	η^{REFP}/ [μPa s]
20	31.2	31.2	43.4 ± 0.1	43.8	265.6 ± 0.7	264.3	7.6 ± 4.2	15.9
40	33.1	33.5	52.2 ± 0.2	54.0	253.1 ± 1.5	249.5	16.9 ± 2.1	16.5
60	35.7 ± 0.1	36.8	70.3 ± 0.9	76.3	238.7 ± 2.3	232.9	18.4 ± 1.8	17.7
80	40.3 ± 0.2	42.8	137.9 ± 6.7	162.3	223.6 ± 8.1	213.7	20.8 ± 1.3	21.0
100	42.6 ± 0.2	46.5	407.9 ± 24.4	310.8	250.9 ± 16.6	239.8	36.5 ± 3.2	39.3
120	40.6 ± 0.4	42.4	169.7 ± 10.6	162.5	362.1 ± 23.5	326.3	51.9 ± 2.9	52.8
140	39.9 ± 0.1	41.1	139.7 ± 6.8	128.8	412.8 ± 20.1	384.9	61.3 ± 3.2	60.2
160	39.3 ± 0.1	40.5	118.5 ± 6.0	113.9	461.4 ± 24.2	429.3	69.4 ± 4.6	65.7
180	39.6 ± 0.1	40.1	115.2 ± 2.7	105.1	492.4 ± 10.7	466.0	72.5 ± 4.7	70.3
200	39.2 ± 0.1	39.9	106.4 ± 1.2	99.3	528.1 ± 9.0	497.6	74.2 ± 1.9	74.2

S16.4 Data of thermodynamics and transport properties of CO_2 rich ternary mixture with 1 mole% impurity of Ar and 1 mole% impurity of CH_4

Table S194: Densities computed from MC and MD simulations (ρ^{MC} and ρ^{MD}), densities obtained from REFPROP[10] (ρ^{REFP}), isothermal compressibilities computed from MC simulations (β_T^{MC}), isothermal compressibilities obtained from REFPROP[10] (β_T^{REFP}), thermal expansion coefficients computed from MC simulations (α_P^{MC}), thermal expansion coefficients obtained from REFPROP[10] (α_P^{REFP}), Joule Thomson coefficients computed from MC simulations ($\mu_{\mathrm{JT}}^{\mathrm{MC}}$), and Joule Thomson coefficients obtained from REFPROP[10] ($\mu_{\mathrm{JT}}^{\mathrm{REFP}}$) of CO_2 rich ternary mixture with 1% impurity of Ar and 1% impurity of CH_4 at 253 K and pressures ranging from 20 bar to 200 bar.

P/ [bar]	ρ^{MC}/ [kg/m^3]	ρ^{MD}/ [kg/m^3]	ρ^{REFP}/ [kg/m^3]	β_T^{MC}/ [10^{-5}/bar]	β_T^{REFP}/ [10^{-5}/bar]	α_P^{MC}/ [10^{-4}/K]	α_P^{REFP}/ [10^{-4}/K]	$\mu_{\mathrm{JT}}^{\mathrm{MC}}$/ [$10^{-3}$ K/bar]	$\mu_{\mathrm{JT}}^{\mathrm{REFP}}$/ [$10^{-3}$ K/bar]
20	50.5	51.5 ± 0.1	52.0	6366.5 ± 89.3	6626.6	76.5 ± 1.3	85.1	1556.7 ± 56.3	1704.8
40	1020.5 ± 0.8	1018.3 ± 0.8	1022.2	38.0 ± 1.8	44.8	43.8 ± 2.1	46.6	4.8 ± 2.4	8.2
60	1028.2 ± 0.9	1025.9 ± 1.1	1031.0	35.5 ± 1.9	40.9	42.0 ± 2.5	43.9	2.8 ± 2.8	5.1
80	1034.8 ± 1.4	1033.3 ± 0.8	1039.1	33.6 ± 2.3	37.8	40.5 ± 2.8	41.6	1.1 ± 3.2	2.4
100	1041.6 ± 0.6	1040.4 ± 0.5	1046.7	31.6 ± 0.7	35.1	38.8 ± 1.3	39.6	-0.8 ± 1.5	0.1
120	1049.1 ± 1.6	1046.8 ± 0.4	1053.8	28.9 ± 0.6	32.8	36.6 ± 0.9	37.9	-3.5 ± 1.1	-2.0
140	1054.3 ± 1.1	1053.1 ± 0.6	1060.6	28.1 ± 0.9	30.8	36.0 ± 1.2	36.4	-4.2 ± 1.4	-3.8
160	1059.7 ± 1.5	1059.2 ± 0.8	1066.9	26.9 ± 1.5	29.1	34.9 ± 1.9	35.1	-5.4 ± 2.3	-5.4
180	1065.0 ± 1.3	1064.0 ± 0.7	1073.0	26.1 ± 0.4	27.6	34.8 ± 0.6	33.9	-5.6 ± 0.7	-6.9
200	1070.6 ± 0.6	1069.3 ± 0.6	1078.8	23.7 ± 0.7	26.3	32.0 ± 1.0	32.8	-9.1 ± 1.2	-8.3

Table S195: Heat capacities at constant volume computed from MC simulations (c_V^{MC}), heat capacities at constant volume obtained from REFPROP[10] (c_V^{REFP}), heat capacities at constant pressure computed from MC simulations (c_P^{MC}), heat capacities at constant pressure obtained from REFPROP[10] (c_P^{REFP}), speed of sound computed from MC simulations (c^{MC}), speed of sound obtained from REFPROP[10] (c^{REFP}), viscosities computed from MD simulations (η^{MD}), and viscosities obtained from REFPROP[10] (η^{REFP}) of CO_2 rich ternary mixture with 1% impurity of Ar and 1% impurity of CH_4 at 253 K and pressures ranging from 20 bar to 200 bar.

P/ [bar]	c_V^{MC}/ [J/mol K]	c_V^{REFP}/ [J/mol K]	c_P^{MC}/ [J/mol K]	c_P^{REFP}/ [J/mol K]	c^{MC}/ [m/s]	c^{REFP}/ [m/s]	η^{MD}/ [μPa s]	η^{REFP}/ [μPa s]
20	31.8	33.6	52.0 ± 0.4	56.8	225.5 ± 1.8	221.5	12.2 ± 2.3	13.0
40	40.3 ± 0.1	40.7	94.9 ± 2.7	93.1	779.5 ± 21.8	706.8	142.3 ± 3.5	136.5
60	40.4 ± 0.2	40.6	93.9 ± 3.6	91.0	798.3 ± 26.1	728.4	146.3 ± 5.6	140.5
80	40.4 ± 0.2	40.7	92.8 ± 3.6	89.3	812.5 ± 31.8	748.4	150.9 ± 4.9	144.3
100	40.6 ± 0.2	40.7	91.0 ± 2.4	87.9	824.9 ± 14.6	767.1	164.8 ± 30.3	147.9
120	40.4 ± 0.2	40.7	89.2 ± 1.5	86.6	853.8 ± 11.7	784.6	158.4 ± 7.5	151.5
140	40.7 ± 0.2	40.7	88.9 ± 1.8	85.5	859.1 ± 16.7	801.1	180.6 ± 21.0	154.9
160	40.6 ± 0.2	40.8	88.0 ± 2.5	84.5	872.0 ± 28.0	816.8	174.8 ± 16.8	158.3
180	40.7 ± 0.4	40.8	88.8 ± 1.1	83.6	885.8 ± 9.2	831.8	170.0 ± 12.9	161.6
200	40.6 ± 0.3	40.8	85.2 ± 1.7	82.9	908.3 ± 16.4	846.1	177.6 ± 11.8	164.8

Table S196: Densities computed from MC and MD simulations (ρ^{MC} and ρ^{MD}), densities obtained from REFPROP[10] (ρ^{REFP}), isothermal compressibilities computed from MC simulations (β_T^{MC}), isothermal compressibilities obtained from REFPROP[10] (β_T^{REFP}), thermal expansion coefficients computed from MC simulations (α_P^{MC}), thermal expansion coefficients obtained from REFPROP[10] (α_P^{REFP}), Joule Thomson coefficients computed from MC simulations (μ_{JT}^{MC}), and Joule Thomson coefficients obtained from REFPROP[10] (μ_{JT}^{REFP}) of CO_2 rich ternary mixture with 1% impurity of Ar and 1% impurity of CH_4 at 273 K and pressures ranging from 20 bar to 200 bar.

P/ [bar]	ρ^{MC}/ [kg/m^3]	ρ^{MD}/ [kg/m^3]	ρ^{REFP}/ [kg/m^3]	β_T^{MC}/ [10^{-5}/bar]	β_T^{REFP}/ [10^{-5}/bar]	α_P^{MC}/ [10^{-4}/K]	α_P^{REFP}/ [10^{-4}/K]	μ_{JT}^{MC}/ [10^{-3} K/bar]	μ_{JT}^{REFP}/ [10^{-3} K/bar]
20	44.2	45.1 ± 0.1	45.1	5903.1 ± 31.5	5986.6	58.5 ± 0.3	61.4	1269.7 ± 17.8	1368.7
40	914.1 ± 3.0	119.8 ± 0.6	630.7	89.7 ± 5.2	-184.2	71.7 ± 3.7	83.7	39.8 ± 4.4	–
60	930.4 ± 1.6	924.8 ± 0.6	926.6	72.6 ± 3.2	89.5	61.9 ± 2.9	67.1	30.1 ± 3.5	37.2
80	942.3 ± 1.3	939.2 ± 1.1	941.8	64.0 ± 3.7	75.0	56.3 ± 3.2	59.4	24.3 ± 4.0	28.9
100	952.8 ± 1.0	950.9 ± 1.0	955.0	55.2 ± 4.3	64.9	50.7 ± 3.9	53.9	17.9 ± 5.0	22.4
120	963.0 ± 1.4	961.9 ± 0.4	966.7	49.5 ± 2.6	57.4	46.8 ± 2.1	49.7	13.3 ± 2.8	17.3
140	972.2 ± 1.5	972.1 ± 0.5	977.3	45.6 ± 2.9	51.6	44.8 ± 2.5	46.3	10.6 ± 3.3	13.0
160	982.1 ± 1.7	980.0 ± 0.8	987.0	42.3 ± 1.3	47.0	42.8 ± 1.1	43.5	8.0 ± 1.5	9.4
180	990.0 ± 0.8	987.8 ± 0.7	995.9	38.0 ± 1.5	43.2	39.3 ± 1.7	41.2	3.6 ± 2.3	6.3
200	996.7 ± 0.5	995.8 ± 0.5	1004.3	37.6 ± 1.3	40.1	39.8 ± 1.5	39.2	4.2 ± 2.0	3.6

Table S197: Heat capacities at constant volume computed from MC simulations (c_V^{MC}), heat capacities at constant volume obtained from REFPROP[10] (c_V^{REFP}), heat capacities at constant pressure computed from MC simulations (c_P^{MC}), heat capacities at constant pressure obtained from REFPROP[10] (c_P^{REFP}), speed of sound computed from MC simulations (c^{MC}), speed of sound obtained from REFPROP[10] (c^{REFP}), viscosities computed from MD simulations (η^{MD}), and viscosities obtained from REFPROP[10] (η^{REFP}) of CO_2 rich ternary mixture with 1% impurity of Ar and 1% impurity of CH_4 at 273 K and pressures ranging from 20 bar to 200 bar.

P/ [bar]	c_V^{MC}/ [J/mol K]	c_V^{REFP}/ [J/mol K]	c_P^{MC}/ [J/mol K]	c_P^{REFP}/ [J/mol K]	c^{MC}/ [m/s]	c^{REFP}/ [m/s]	η^{MD}/ [μPa s]	η^{REFP}/ [μPa s]
20	30.9	31.3	46.5 ± 0.1	48.0	240.2 ± 0.7	238.3	3.2 ± 4.3	14.0
40	39.7 ± 0.2	–	115.1 ± 3.6	–	594.4 ± 19.6	–	15.3 ± 1.4	49.3
60	39.8 ± 0.2	40.6	107.7 ± 3.4	105.4	632.9 ± 17.0	559.7	107.4 ± 1.9	100.7
80	39.7 ± 0.2	40.4	102.8 ± 3.8	100.1	655.3 ± 22.4	592.4	111.7 ± 3.1	105.3
100	39.7 ± 0.3	40.3	98.1 ± 4.5	96.2	686.0 ± 30.8	620.9	113.1 ± 5.6	109.4
120	39.8 ± 0.2	40.2	94.8 ± 2.7	93.2	707.4 ± 21.0	646.2	120.1 ± 2.5	113.2
140	39.8 ± 0.1	40.2	93.8 ± 2.8	90.9	729.5 ± 25.4	669.3	125.6 ± 5.6	116.9
160	39.9 ± 0.1	40.2	92.4 ± 1.4	88.9	745.8 ± 12.5	690.5	131.9 ± 7.9	120.3
180	39.7 ± 0.1	40.2	88.9 ± 2.3	87.3	770.6 ± 18.5	710.2	136.6 ± 8.6	123.6
200	39.9 ± 0.1	40.2	90.3 ± 2.0	85.8	777.1 ± 16.1	728.7	130.6 ± 4.0	126.8

Table S198: Densities computed from MC and MD simulations (ρ^{MC} and ρ^{MD}), densities obtained from REFPROP[10] (ρ^{REFP}), isothermal compressibilities computed from MC simulations (β_T^{MC}), isothermal compressibilities obtained from REFPROP[10] (β_T^{REFP}), thermal expansion coefficients computed from MC simulations (α_P^{MC}), thermal expansion coefficients obtained from REFPROP[10] (α_P^{REFP}), Joule Thomson coefficients computed from MC simulations ($\mu_{\mathrm{JT}}^{\mathrm{MC}}$), and Joule Thomson coefficients obtained from REFPROP[10] ($\mu_{\mathrm{JT}}^{\mathrm{REFP}}$) of CO_2 rich ternary mixture with 1% impurity of Ar and 1% impurity of CH_4 at 293 K and pressures ranging from 20 bar to 200 bar.

P/ [bar]	ρ^{MC}/ [kg/m^3]	ρ^{MD}/ [kg/m^3]	ρ^{REFP}/ [kg/m^3]	β_T^{MC}/ [10^{-5}/bar]	β_T^{REFP}/ [10^{-5}/bar]	α_P^{MC}/ [10^{-4}/K]	α_P^{REFP}/ [10^{-4}/K]	$\mu_{\mathrm{JT}}^{\mathrm{MC}}$/ [$10^{-3}$ K/bar]	$\mu_{\mathrm{JT}}^{\mathrm{REFP}}$/ [$10^{-3}$ K/bar]
20	39.8	40.6 ± 0.1	40.4	5613.8 ± 28.0	5686.9	48.4 ± 0.3	50.2	1038.6 ± 21.7	1130.9
40	92.5 ± 0.1	94.7 ± 0.3	95.8	3434.1 ± 29.6	3587.7	80.2 ± 0.8	88.2	1064.4 ± 19.5	1130.6
60	186.7 ± 1.1	572.8 ± 205.1	295.9	4522.2 ± 181.9	$-46\,250.3$	268.3 ± 13.1	-5410.7	1060.3 ± 74.7	–
80	810.9 ± 3.4	801.8 ± 1.9	796.8	183.1 ± 12.0	265.9	103.5 ± 5.2	127.7	82.4 ± 6.6	107.1
100	835.5 ± 4.2	833.2 ± 1.3	831.1	136.1 ± 11.8	170.5	83.3 ± 5.3	93.1	63.6 ± 7.2	76.2
120	856.6 ± 1.2	855.0 ± 1.6	855.9	110.7 ± 1.8	127.8	73.1 ± 0.7	76.3	52.0 ± 1.0	58.1
140	875.9 ± 2.9	873.6 ± 0.6	875.7	91.1 ± 6.2	103.1	64.0 ± 3.4	66.0	41.3 ± 4.9	45.7
160	890.2 ± 0.8	888.2 ± 0.8	892.4	76.9 ± 2.9	86.8	57.2 ± 1.6	58.9	32.9 ± 2.4	36.5
180	901.6 ± 0.8	899.7 ± 0.8	906.9	68.9 ± 2.8	75.2	53.4 ± 2.1	53.6	27.8 ± 3.1	29.2
200	913.1 ± 2.4	912.0 ± 1.0	919.8	58.7 ± 1.4	66.4	47.2 ± 1.3	49.4	19.8 ± 2.0	23.4

Table S199: Heat capacities at constant volume computed from MC simulations (c_V^{MC}), heat capacities at constant volume obtained from REFPROP[10] (c_V^{REFP}), heat capacities at constant pressure computed from MC simulations (c_P^{MC}), heat capacities at constant pressure obtained from REFPROP[10] (c_P^{REFP}), speed of sound computed from MC simulations (c^{MC}), speed of sound obtained from REFPROP[10] (c^{REFP}), viscosities computed from MD simulations (η^{MD}), and viscosities obtained from REFPROP[10] (η^{REFP}) of CO_2 rich ternary mixture with 1% impurity of Ar and 1% impurity of CH_4 at 293 K and pressures ranging from 20 bar to 200 bar.

P/ [bar]	c_V^{MC}/ [J/mol K]	c_V^{REFP}/ [J/mol K]	c_P^{MC}/ [J/mol K]	c_P^{REFP}/ [J/mol K]	c^{MC}/ [m/s]	c^{REFP}/ [m/s]	η^{MD}/ [μPa s]	η^{REFP}/ [μPa s]
20	30.8	31.0	44.3 ± 0.1	45.0	253.5 ± 0.7	251.6	10.6 ± 3.3	15.0
40	33.9 ± 0.1	34.9	59.9 ± 0.3	63.9	235.7 ± 1.2	230.8	15.0 ± 0.7	15.7
60	41.4 ± 0.5	–	151.5 ± 6.4	–	208.2 ± 6.2	–	43.0 ± 19.3	21.7
80	39.7 ± 0.2	41.9	132.9 ± 3.6	140.3	474.9 ± 16.9	397.7	73.3 ± 0.8	71.1
100	39.5 ± 0.2	40.9	118.6 ± 4.0	119.2	513.7 ± 24.0	453.5	85.1 ± 5.6	77.6
120	39.4 ± 0.2	40.5	112.1 ± 0.7	108.7	547.9 ± 4.9	495.6	86.4 ± 2.6	82.8
140	39.3 ± 0.1	40.2	105.8 ± 3.5	102.0	580.5 ± 21.9	530.3	91.3 ± 5.9	87.3
160	39.5 ± 0.1	40.0	100.8 ± 1.5	97.3	610.0 ± 12.4	560.3	101.6 ± 3.9	91.3
180	39.4 ± 0.1	39.9	98.5 ± 2.4	93.8	634.5 ± 15.2	587.0	101.4 ± 6.2	94.9
200	39.4 ± 0.1	39.9	92.9 ± 1.6	91.1	663.2 ± 9.8	611.2	105.6 ± 8.2	98.4

Table S200: Densities computed from MC and MD simulations (ρ^{MC} and ρ^{MD}), densities obtained from REFPROP[10] (ρ^{REFP}), isothermal compressibilities computed from MC simulations (β_T^{MC}), isothermal compressibilities obtained from REFPROP[10] (β_T^{REFP}), thermal expansion coefficients computed from MC simulations (α_P^{MC}), thermal expansion coefficients obtained from REFPROP[10] (α_P^{REFP}), Joule Thomson coefficients computed from MC simulations ($\mu_{\mathrm{JT}}^{\mathrm{MC}}$), and Joule Thomson coefficients obtained from REFPROP[10] ($\mu_{\mathrm{JT}}^{\mathrm{REFP}}$) of CO_2 rich ternary mixture with 1% impurity of Ar and 1% impurity of CH_4 at 313 K and pressures ranging from 20 bar to 200 bar.

P/ [bar]	ρ^{MC}/ [kg/m^3]	ρ^{MD}/ [kg/m^3]	ρ^{REFP}/ [kg/m^3]	β_T^{MC}/ [10^{-5}/bar]	β_T^{REFP}/ [10^{-5}/bar]	α_P^{MC}/ [10^{-4}/K]	α_P^{REFP}/ [10^{-4}/K]	$\mu_{\mathrm{JT}}^{\mathrm{MC}}$/ [$10^{-3}$ K/bar]	$\mu_{\mathrm{JT}}^{\mathrm{REFP}}$/ [$10^{-3}$ K/bar]
20	36.4	37.0 ± 0.1	36.8	5469.1 ± 18.8	5508.1	42.1 ± 0.2	43.1	884.9 ± 14.0	952.0
40	80.7	82.3 ± 0.2	82.6	3098.3 ± 21.6	3169.1	59.3 ± 0.5	62.8	886.5 ± 15.9	944.5
60	139.7 ± 0.1	144.1 ± 0.7	145.8	2500.2 ± 51.1	2667.3	94.6 ± 1.6	106.7	866.2 ± 23.2	912.0
80	238.0 ± 1.1	258.4 ± 5.8	259.0	3070.6 ± 198.9	3389.3	233.3 ± 15.5	285.3	809.2 ± 73.8	–
100	569.7 ± 7.0	562.5 ± 5.5	565.0	2403.8 ± 303.1	1947.0	569.8 ± 58.0	453.6	341.0 ± 46.0	–
120	685.4 ± 5.8	685.0 ± 1.6	683.7	441.8 ± 39.0	509.6	165.7 ± 14.0	173.5	161.1 ± 19.6	176.4
140	736.2 ± 2.4	737.0 ± 3.2	736.8	265.3 ± 25.3	282.1	117.5 ± 9.5	115.1	115.6 ± 14.1	120.3
160	770.1 ± 2.5	768.3 ± 2.3	772.0	177.9 ± 12.7	195.5	89.3 ± 5.9	89.9	85.1 ± 9.5	90.3
180	793.0 ± 2.1	793.0 ± 1.2	798.8	141.0 ± 9.2	150.0	76.1 ± 4.2	75.5	68.9 ± 6.9	70.9
200	816.4 ± 2.1	813.5 ± 1.6	820.6	109.3 ± 10.8	121.8	64.0 ± 5.5	65.9	52.6 ± 9.3	57.0

Table S201: Heat capacities at constant volume computed from MC simulations (c_V^{MC}), heat capacities at constant volume obtained from REFPROP[10] (c_V^{REFP}), heat capacities at constant pressure computed from MC simulations (c_P^{MC}), heat capacities at constant pressure obtained from REFPROP[10] (c_P^{REFP}), speed of sound computed from MC simulations (c^{MC}), speed of sound obtained from REFPROP[10] (c^{REFP}), viscosities computed from MD simulations (η^{MD}), and viscosities obtained from REFPROP[10] (η^{REFP}) of CO_2 rich ternary mixture with 1% impurity of Ar and 1% impurity of CH_4 at 313 K and pressures ranging from 20 bar to 200 bar.

P/ [bar]	c_V^{MC}/ [J/mol K]	c_V^{REFP}/ [J/mol K]	c_P^{MC}/ [J/mol K]	c_P^{REFP}/ [J/mol K]	c^{MC}/ [m/s]	c^{REFP}/ [m/s]	η^{MD}/ [μPa s]	η^{REFP}/ [μPa s]
20	31.1	31.2	43.3 ± 0.1	43.7	264.6 ± 0.5	263.2	10.5 ± 3.1	16.0
40	33.0	33.5	52.3 ± 0.2	54.1	251.5 ± 1.0	248.4	17.6 ± 1.6	16.6
60	35.7 ± 0.1	36.8	70.8 ± 0.5	76.9	238.4 ± 2.6	231.6	17.4 ± 1.1	17.8
80	40.2 ± 0.3	–	143.0 ± 7.0	–	220.5 ± 9.0	–	21.6 ± 1.2	21.3
100	42.4 ± 0.6	–	379.0 ± 30.4	–	255.7 ± 19.3	–	39.9 ± 1.4	41.2
120	40.2 ± 0.3	42.4	165.6 ± 10.3	160.5	368.7 ± 20.0	329.8	58.4 ± 7.1	54.5
140	39.7 ± 0.1	41.1	137.4 ± 6.8	128.2	421.1 ± 22.6	387.6	69.6 ± 11.3	61.8
160	39.5 ± 0.1	40.4	119.6 ± 5.1	113.7	470.0 ± 19.6	431.5	66.8 ± 4.6	67.4
180	39.2 ± 0.2	40.1	110.4 ± 3.5	105.1	502.3 ± 18.4	467.7	79.4 ± 3.4	72.0
200	39.1 ± 0.1	39.9	102.2 ± 4.9	99.3	541.0 ± 29.8	499.0	82.8 ± 11.8	76.0

S16.5 Data of thermodynamics and transport properties of CO_2 rich ternary mixture with 1 mole% impurity of Ar and 1 mole% impurity of H_2

Table S202: Densities computed from MC and MD simulations (ρ^{MC} and ρ^{MD}), densities obtained from REFPROP[10] (ρ^{REFP}), isothermal compressibilities computed from MC simulations (β_T^{MC}), isothermal compressibilities obtained from REFPROP[10] (β_T^{REFP}), thermal expansion coefficients computed from MC simulations (α_P^{MC}), thermal expansion coefficients obtained from REFPROP[10] (α_P^{REFP}), Joule Thomson coefficients computed from MC simulations (μ_{JT}^{MC}), and Joule Thomson coefficients obtained from REFPROP[10] (μ_{JT}^{REFP}) of CO_2 rich ternary mixture with 1% impurity of Ar and 1% impurity of H_2 at 253 K and pressures ranging from 20 bar to 200 bar.

P/ [bar]	ρ^{MC}/ [kg/m^3]	ρ^{MD}/ [kg/m^3]	ρ^{REFP}/ [kg/m^3]	β_T^{MC}/ [10^{-5}/bar]	β_T^{REFP}/ [10^{-5}/bar]	α_P^{MC}/ [10^{-4}/K]	α_P^{REFP}/ [10^{-4}/K]	μ_{JT}^{MC}/ [10^{-3} K/bar]	μ_{JT}^{REFP}/ [10^{-3} K/bar]
20	50.2	51.2	51.7	6342.4 ± 35.9	6583.7	76.0 ± 0.7	83.7	1546.0 ± 30.2	1684.9
40	1017.2 ± 1.8	1014.3 ± 0.9	989.2	41.6 ± 2.4	61.5	46.7 ± 3.0	57.6	8.0 ± 3.4	–
60	1024.4 ± 0.7	1022.7 ± 0.7	1023.5	38.8 ± 2.0	42.1	45.2 ± 2.4	44.4	6.2 ± 2.7	5.8
80	1032.3 ± 1.1	1030.0 ± 1.1	1031.8	34.4 ± 1.0	38.7	41.3 ± 1.2	42.1	2.1 ± 1.4	3.0
100	1038.8 ± 1.0	1037.4 ± 0.5	1039.6	33.6 ± 0.8	35.9	40.6 ± 1.5	40.0	1.2 ± 1.7	0.6
120	1044.9 ± 0.8	1044.4 ± 0.4	1046.8	31.6 ± 0.9	33.5	39.1 ± 1.3	38.3	-0.5 ± 1.5	-1.5
140	1052.0 ± 0.9	1050.2 ± 0.9	1053.6	29.5 ± 1.3	31.5	37.3 ± 1.7	36.7	-2.6 ± 1.9	-3.4
160	1058.7 ± 1.0	1056.9 ± 0.4	1060.1	26.9 ± 0.9	29.7	34.8 ± 1.2	35.4	-5.6 ± 1.4	-5.1
180	1063.7 ± 0.8	1062.3 ± 0.8	1066.2	24.9 ± 1.2	28.1	32.9 ± 1.8	34.1	-8.0 ± 2.2	-6.7
200	1069.1 ± 0.3	1068.1 ± 0.3	1072.1	24.6 ± 0.5	26.7	33.1 ± 1.0	33.1	-7.6 ± 1.2	-8.0

Table S203: Heat capacities at constant volume computed from MC simulations (c_V^{MC}), heat capacities at constant volume obtained from REFPROP[10] (c_V^{REFP}), heat capacities at constant pressure computed from MC simulations (c_P^{MC}), heat capacities at constant pressure obtained from REFPROP[10] (c_P^{REFP}), speed of sound computed from MC simulations (c^{MC}), speed of sound obtained from REFPROP[10] (c^{REFP}), viscosities computed from MD simulations (η^{MD}), and viscosities obtained from REFPROP[10] (η^{REFP}) of CO_2 rich ternary mixture with 1% impurity of Ar and 1% impurity of H_2 at 253 K and pressures ranging from 20 bar to 200 bar.

P/ [bar]	c_V^{MC}/ [J/mol K]	c_V^{REFP}/ [J/mol K]	c_P^{MC}/ [J/mol K]	c_P^{REFP}/ [J/mol K]	c^{MC}/ [m/s]	c^{REFP}/ [m/s]	η^{MD}/ [μPa s]	η^{REFP}/ [μPa s]
20	31.8	33.2	51.8 ± 0.3	55.9	226.3 ± 0.9	222.5	10.2 ± 2.5	13.0
40	40.5 ± 0.2	–	97.1 ± 4.4	–	753.5 ± 28.1	–	143.3 ± 6.6	122.9
60	40.4 ± 0.2	40.6	96.9 ± 3.2	91.1	776.5 ± 24.2	722.4	145.4 ± 1.3	137.2
80	40.5 ± 0.1	40.6	93.3 ± 1.6	89.3	805.5 ± 13.3	742.7	151.1 ± 7.7	141.0
100	40.3 ± 0.3	40.6	92.6 ± 2.5	87.8	812.2 ± 14.9	761.6	156.0 ± 16.1	144.6
120	40.5 ± 0.2	40.6	91.6 ± 2.2	86.5	828.1 ± 15.4	779.4	160.7 ± 16.9	148.2
140	40.6 ± 0.3	40.6	89.6 ± 2.3	85.4	842.4 ± 21.6	796.1	157.3 ± 7.0	151.6
160	40.7 ± 0.2	40.7	87.4 ± 1.6	84.4	869.1 ± 17.4	812.0	193.2 ± 52.3	154.9
180	40.6 ± 0.1	40.7	85.4 ± 2.7	83.5	891.2 ± 26.5	827.1	177.5 ± 20.9	158.2
200	40.7 ± 0.1	40.7	86.5 ± 1.7	82.7	899.7 ± 13.1	841.5	169.9 ± 11.4	161.4

Table S204: Densities computed from MC and MD simulations (ρ^{MC} and ρ^{MD}), densities obtained from REFPROP[10] (ρ^{REFP}), isothermal compressibilities computed from MC simulations (β_T^{MC}), isothermal compressibilities obtained from REFPROP[10] (β_T^{REFP}), thermal expansion coefficients computed from MC simulations (α_P^{MC}), thermal expansion coefficients obtained from REFPROP[10] (α_P^{REFP}), Joule Thomson coefficients computed from MC simulations ($\mu_{\text{JT}}^{\text{MC}}$), and Joule Thomson coefficients obtained from REFPROP[10] ($\mu_{\text{JT}}^{\text{REFP}}$) of CO_2 rich ternary mixture with 1% impurity of Ar and 1% impurity of H_2 at 273 K and pressures ranging from 20 bar to 200 bar.

P/ [bar]	ρ^{MC}/ [kg/m^3]	ρ^{MD}/ [kg/m^3]	ρ^{REFP}/ [kg/m^3]	β_T^{MC}/ [10^{-5}/bar]	β_T^{REFP}/ [10^{-5}/bar]	α_P^{MC}/ [10^{-4}/K]	α_P^{REFP}/ [10^{-4}/K]	$\mu_{\text{JT}}^{\text{MC}}$/ [$10^{-3}$ K/bar]	$\mu_{\text{JT}}^{\text{REFP}}$/ [$10^{-3}$ K/bar]
20	44.0	44.8 ± 0.1	44.9	5853.9 ± 35.3	5969.9	57.9 ± 0.3	61.0	1239.9 ± 18.3	1355.0
40	–	117.5 ± 1.3	432.4	–	633.1	–	250.9	–	–
60	924.0 ± 2.0	918.9 ± 0.7	918.1	78.9 ± 3.7	93.4	64.7 ± 2.8	68.8	33.3 ± 3.5	39.3
80	936.7 ± 2.0	932.3 ± 1.1	933.8	66.3 ± 2.8	77.7	57.2 ± 2.3	60.6	25.4 ± 2.9	30.4
100	949.5 ± 1.9	946.0 ± 0.9	947.3	59.3 ± 2.5	66.9	53.6 ± 2.1	54.8	21.1 ± 2.7	23.6
120	959.6 ± 1.5	957.2 ± 0.2	959.3	51.0 ± 3.2	59.0	47.9 ± 2.6	50.4	14.6 ± 3.4	18.2
140	969.6 ± 1.8	966.3 ± 0.6	970.1	49.0 ± 1.6	53.0	47.4 ± 1.2	46.9	13.7 ± 1.5	13.8
160	978.0 ± 1.4	976.0 ± 1.0	979.9	44.8 ± 0.6	48.1	44.6 ± 0.7	44.0	10.3 ± 0.9	10.1
180	985.9 ± 1.0	985.5 ± 0.5	989.0	40.7 ± 1.2	44.2	42.0 ± 1.4	41.6	7.0 ± 1.9	6.9
200	993.9 ± 1.1	992.5 ± 1.1	997.5	36.8 ± 1.3	40.9	38.3 ± 1.4	39.6	2.3 ± 1.9	4.1

Table S205: Heat capacities at constant volume computed from MC simulations (c_V^{MC}), heat capacities at constant volume obtained from REFPROP[10] (c_V^{REFP}), heat capacities at constant pressure computed from MC simulations (c_P^{MC}), heat capacities at constant pressure obtained from REFPROP[10] (c_P^{REFP}), speed of sound computed from MC simulations (c^{MC}), speed of sound obtained from REFPROP[10] (c^{REFP}), viscosities computed from MD simulations (η^{MD}), and viscosities obtained from REFPROP[10] (η^{REFP}) of CO_2 rich ternary mixture with 1% impurity of Ar and 1% impurity of H_2 at 273 K and pressures ranging from 20 bar to 200 bar.

P/ [bar]	c_V^{MC}/ [J/mol K]	c_V^{REFP}/ [J/mol K]	c_P^{MC}/ [J/mol K]	c_P^{REFP}/ [J/mol K]	c^{MC}/ [m/s]	c^{REFP}/ [m/s]	η^{MD}/ [μPa s]	η^{REFP}/ [μPa s]
20	30.8	31.1	46.3 ± 0.1	47.6	241.5 ± 0.8	239.1	5.9 ± 4.7	14.0
40	–	–	–	–	–	–	15.1 ± 0.8	28.2
60	39.8 ± 0.3	40.5	108.3 ± 3.4	106.1	611.2 ± 17.3	552.9	105.4 ± 2.0	98.2
80	39.7 ± 0.1	40.3	102.8 ± 2.5	100.5	645.9 ± 15.9	586.4	107.9 ± 2.7	102.8
100	39.7 ± 0.2	40.2	100.8 ± 2.5	96.4	671.7 ± 16.8	615.4	110.2 ± 7.7	106.9
120	39.7 ± 0.2	40.1	95.5 ± 3.0	93.3	700.8 ± 24.4	641.2	121.4 ± 3.8	110.7
140	39.6 ± 0.1	40.1	96.0 ± 1.0	90.9	714.2 ± 12.4	664.5	126.1 ± 9.4	114.3
160	39.9 ± 0.3	40.0	93.9 ± 1.1	88.9	732.8 ± 7.1	686.0	142.4 ± 27.7	117.8
180	39.8 ± 0.1	40.0	92.3 ± 2.2	87.2	760.4 ± 14.2	706.0	126.4 ± 6.1	121.0
200	39.8 ± 0.2	40.0	87.5 ± 1.8	85.7	775.9 ± 16.0	724.6	128.7 ± 5.8	124.2

Table S206: Densities computed from MC and MD simulations (ρ^{MC} and ρ^{MD}), densities obtained from REFPROP[10] (ρ^{REFP}), isothermal compressibilities computed from MC simulations (β_T^{MC}), isothermal compressibilities obtained from REFPROP[10] (β_T^{REFP}), thermal expansion coefficients computed from MC simulations (α_P^{MC}), thermal expansion coefficients obtained from REFPROP[10] (α_P^{REFP}), Joule Thomson coefficients computed from MC simulations ($\mu_{\mathrm{JT}}^{\mathrm{MC}}$), and Joule Thomson coefficients obtained from REFPROP[10] ($\mu_{\mathrm{JT}}^{\mathrm{REFP}}$) of CO_2 rich ternary mixture with 1% impurity of Ar and 1% impurity of H_2 at 293 K and pressures ranging from 20 bar to 200 bar.

P/ [bar]	ρ^{MC}/ [kg/m^3]	ρ^{MD}/ [kg/m^3]	ρ^{REFP}/ [kg/m^3]	β_T^{MC}/ [10^{-5}/bar]	β_T^{REFP}/ [10^{-5}/bar]	α_P^{MC}/ [10^{-4}/K]	α_P^{REFP}/ [10^{-4}/K]	$\mu_{\mathrm{JT}}^{\mathrm{MC}}$/ [$10^{-3}$ K/bar]	$\mu_{\mathrm{JT}}^{\mathrm{REFP}}$/ [$10^{-3}$ K/bar]
20	39.6	40.3 ± 0.1	40.2	5611.0 ± 32.0	5677.5	48.3 ± 0.3	49.9	1035.3 ± 21.7	1120.9
40	91.8 ± 0.2	94.0 ± 0.4	95.0	3460.6 ± 54.5	3560.2	80.0 ± 1.3	86.6	1071.2 ± 32.8	1120.4
60	181.8 ± 0.6	228.1 ± 66.7	246.1	4108.7 ± 81.2	10 440.9	236.4 ± 2.2	921.1	1033.2 ± 12.6	–
80	793.0 ± 5.0	792.3 ± 3.1	785.5	270.2 ± 31.9	289.4	139.1 ± 12.7	135.0	107.4 ± 14.2	113.7
100	827.5 ± 4.0	822.7 ± 2.8	821.7	151.0 ± 18.9	179.9	89.7 ± 7.8	96.2	69.9 ± 10.3	79.9
120	849.0 ± 3.0	846.1 ± 1.7	847.4	115.2 ± 6.9	133.1	74.0 ± 3.5	78.1	53.7 ± 5.0	60.6
140	867.3 ± 1.8	865.3 ± 1.1	867.8	98.3 ± 6.8	106.6	67.6 ± 4.0	67.2	45.4 ± 5.6	47.5
160	883.5 ± 1.0	880.0 ± 0.7	884.9	80.5 ± 4.3	89.3	58.6 ± 2.6	59.7	34.9 ± 3.8	37.9
180	896.0 ± 1.0	895.4 ± 0.8	899.7	73.9 ± 2.6	77.1	56.2 ± 1.7	54.2	31.3 ± 2.5	30.4
200	908.2 ± 1.6	907.5 ± 0.9	912.8	66.4 ± 2.8	68.0	52.3 ± 2.1	50.0	26.3 ± 3.1	24.3

Table S207: Heat capacities at constant volume computed from MC simulations (c_V^{MC}), heat capacities at constant volume obtained from REFPROP[10] (c_V^{REFP}), heat capacities at constant pressure computed from MC simulations (c_P^{MC}), heat capacities at constant pressure obtained from REFPROP[10] (c_P^{REFP}), speed of sound computed from MC simulations (c^{MC}), speed of sound obtained from REFPROP[10] (c^{REFP}), viscosities computed from MD simulations (η^{MD}), and viscosities obtained from REFPROP[10] (η^{REFP}) of CO_2 rich ternary mixture with 1% impurity of Ar and 1% impurity of H_2 at 293 K and pressures ranging from 20 bar to 200 bar.

P/ [bar]	c_V^{MC}/ [J/mol K]	c_V^{REFP}/ [J/mol K]	c_P^{MC}/ [J/mol K]	c_P^{REFP}/ [J/mol K]	c^{MC}/ [m/s]	c^{REFP}/ [m/s]	η^{MD}/ [μPa s]	η^{REFP}/ [μPa s]
20	30.8	30.8	44.2 ± 0.1	44.8	254.1 ± 0.8	252.3	6.7 ± 5.4	15.0
40	33.9 ± 0.1	34.6	59.5 ± 0.5	62.9	235.3 ± 2.1	231.8	15.4 ± 1.0	15.7
60	40.2 ± 0.3	–	137.4 ± 0.6	–	213.9 ± 2.3	–	18.0 ± 1.9	19.6
80	40.1 ± 0.1	41.8	157.3 ± 8.4	144.1	427.8 ± 27.7	389.2	72.6 ± 1.7	69.0
100	39.9 ± 0.2	40.8	122.5 ± 4.9	120.6	495.8 ± 32.7	447.2	76.0 ± 2.5	75.6
120	39.6 ± 0.2	40.3	111.6 ± 3.1	109.3	536.5 ± 17.9	490.3	83.7 ± 2.3	80.9
140	39.5 ± 0.2	40.0	108.2 ± 3.7	102.3	566.9 ± 21.9	525.6	88.5 ± 5.7	85.3
160	39.5 ± 0.2	39.9	101.3 ± 2.4	97.5	600.7 ± 17.7	556.0	87.9 ± 16.4	89.3
180	39.3 ± 0.1	39.8	100.4 ± 1.7	93.9	621.2 ± 12.2	583.0	93.7 ± 3.1	93.0
200	39.3 ± 0.1	39.7	97.5 ± 2.3	91.0	640.9 ± 15.8	607.5	96.0 ± 1.5	96.4

Table S208: Densities computed from MC and MD simulations (ρ^{MC} and ρ^{MD}), densities obtained from REFPROP[10] (ρ^{REFP}), isothermal compressibilities computed from MC simulations (β_T^{MC}), isothermal compressibilities obtained from REFPROP[10] (β_T^{REFP}), thermal expansion coefficients computed from MC simulations (α_P^{MC}), thermal expansion coefficients obtained from REFPROP[10] (α_P^{REFP}), Joule Thomson coefficients computed from MC simulations (μ_{JT}^{MC}), and Joule Thomson coefficients obtained from REFPROP[10] (μ_{JT}^{REFP}) of CO_2 rich ternary mixture with 1% impurity of Ar and 1% impurity of H_2 at 313 K and pressures ranging from 20 bar to 200 bar.

P/ [bar]	ρ^{MC}/ [kg/m^3]	ρ^{MD}/ [kg/m^3]	ρ^{REFP}/ [kg/m^3]	β_T^{MC}/ [10^{-5}/bar]	β_T^{REFP}/ [10^{-5}/bar]	α_P^{MC}/ [10^{-4}/K]	α_P^{REFP}/ [10^{-4}/K]	μ_{JT}^{MC}/ [10^{-3} K/bar]	μ_{JT}^{REFP}/ [10^{-3} K/bar]
20	36.2	36.9 ± 0.1	36.6	5443.9 ± 23.1	5502.0	41.9 ± 0.2	43.0	865.5 ± 17.5	944.4
40	80.2 ± 0.1	81.9 ± 0.2	82.2	3080.8 ± 12.3	3157.3	58.8 ± 0.3	62.2	877.0 ± 10.8	936.6
60	138.1 ± 0.2	142.0 ± 0.9	144.4	2494.7 ± 70.4	2637.2	93.2 ± 2.8	104.2	863.9 ± 42.1	905.0
80	231.0 ± 0.6	246.1 ± 2.7	252.4	2942.0 ± 123.6	3214.9	217.4 ± 11.1	261.6	805.8 ± 60.3	–
100	508.0 ± 12.4	521.4 ± 4.5	540.8	3337.7 ± 588.9	2280.8	668.8 ± 95.1	498.8	410.5 ± 76.4	366.3
120	672.8 ± 6.3	661.0 ± 5.7	671.4	536.7 ± 97.0	552.0	189.7 ± 27.1	182.3	178.7 ± 34.8	185.6
140	726.9 ± 3.4	722.8 ± 3.3	727.1	288.2 ± 47.2	296.8	121.9 ± 15.1	118.4	122.2 ± 22.2	125.2
160	759.1 ± 0.5	758.0 ± 1.8	763.5	194.9 ± 13.3	203.2	93.8 ± 6.3	91.8	91.7 ± 10.1	93.5
180	787.0 ± 2.8	786.6 ± 1.9	791.0	144.0 ± 9.3	154.8	76.4 ± 4.1	76.7	70.0 ± 6.9	73.1
200	807.4 ± 1.4	805.6 ± 1.5	813.3	118.7 ± 3.3	125.2	67.5 ± 1.7	66.8	57.5 ± 2.8	58.7

Table S209: Heat capacities at constant volume computed from MC simulations (c_V^{MC}), heat capacities at constant volume obtained from REFPROP[10] (c_V^{REFP}), heat capacities at constant pressure computed from MC simulations (c_P^{MC}), heat capacities at constant pressure obtained from REFPROP[10] (c_P^{REFP}), speed of sound computed from MC simulations (c^{MC}), speed of sound obtained from REFPROP[10] (c^{REFP}), viscosities computed from MD simulations (η^{MD}), and viscosities obtained from REFPROP[10] (η^{REFP}) of CO_2 rich ternary mixture with 1% impurity of Ar and 1% impurity of H_2 at 313 K and pressures ranging from 20 bar to 200 bar.

P/ [bar]	c_V^{MC}/ [J/mol K]	c_V^{REFP}/ [J/mol K]	c_P^{MC}/ [J/mol K]	c_P^{REFP}/ [J/mol K]	c^{MC}/ [m/s]	c^{REFP}/ [m/s]	η^{MD}/ [μPa s]	η^{REFP}/ [μPa s]
20	31.1	31.0	43.2 ± 0.1	43.6	265.6 ± 0.6	263.8	14.3 ± 3.2	16.0
40	32.9	33.3	52.0 ± 0.1	53.7	252.8 ± 0.6	249.2	17.8 ± 1.2	16.6
60	35.6 ± 0.1	36.5	70.0 ± 1.1	75.4	238.9 ± 3.9	232.9	17.7 ± 1.1	17.8
80	39.5 ± 0.4	–	135.8 ± 6.1	–	224.9 ± 7.0	–	22.1 ± 2.4	21.0
100	43.4 ± 0.5	46.3	417.1 ± 45.3	321.3	238.2 ± 24.9	237.2	35.8 ± 3.2	38.9
120	40.4 ± 0.2	42.2	178.9 ± 16.3	164.5	350.2 ± 35.5	324.1	51.2 ± 1.8	52.8
140	39.9 ± 0.3	40.9	138.1 ± 9.6	129.5	406.4 ± 36.2	383.0	61.8 ± 1.4	60.3
160	39.4 ± 0.2	40.3	121.1 ± 5.3	114.2	455.8 ± 18.6	427.5	69.3 ± 4.4	65.8
180	39.3 ± 0.1	39.9	109.9 ± 3.7	105.3	497.0 ± 18.1	464.1	74.9 ± 2.4	70.4
200	39.2 ± 0.2	39.7	104.5 ± 1.5	99.4	527.5 ± 8.4	495.7	76.7 ± 2.2	74.5

S16.6 Data of thermodynamics and transport properties of CO_2 rich ternary mixture with 1 mole% impurity of N_2 and 1 mole% impurity of CH_4

Table S210: Densities computed from MC and MD simulations (ρ^{MC} and ρ^{MD}), densities obtained from REFPROP[10] (ρ^{REFP}), isothermal compressibilities computed from MC simulations (β_T^{MC}), isothermal compressibilities obtained from REFPROP[10] (β_T^{REFP}), thermal expansion coefficients computed from MC simulations (α_P^{MC}), thermal expansion coefficients obtained from REFPROP[10] (α_P^{REFP}), Joule Thomson coefficients computed from MC simulations ($\mu_{\mathrm{JT}}^{\mathrm{MC}}$), and Joule Thomson coefficients obtained from REFPROP[10] ($\mu_{\mathrm{JT}}^{\mathrm{REFP}}$) of CO_2 rich ternary mixture with 1% impurity of N_2 and 1% impurity of CH_4 at 253 K and pressures ranging from 20 bar to 200 bar.

P/ [bar]	ρ^{MC}/ [kg/m^3]	ρ^{MD}/ [kg/m^3]	ρ^{REFP}/ [kg/m^3]	β_T^{MC}/ [10^{-5}/bar]	β_T^{REFP}/ [10^{-5}/bar]	α_P^{MC}/ [10^{-4}/K]	α_P^{REFP}/ [10^{-4}/K]	$\mu_{\mathrm{JT}}^{\mathrm{MC}}$/ [$10^{-3}$ K/bar]	$\mu_{\mathrm{JT}}^{\mathrm{REFP}}$/ [$10^{-3}$ K/bar]
20	50.4	51.5 ± 0.2	51.9	6373.7 ± 72.6	6637.0	76.6 ± 1.1	85.5	1558.5 ± 50.0	1708.7
40	1015.8 ± 1.1	1011.8 ± 1.0	1016.8	39.0 ± 1.8	45.7	44.9 ± 2.0	47.0	6.0 ± 2.3	8.7
60	1022.9 ± 1.5	1020.3 ± 0.5	1025.8	35.7 ± 1.6	41.6	41.9 ± 1.8	44.2	2.7 ± 2.1	5.5
80	1029.1 ± 1.4	1026.7 ± 1.2	1034.0	34.3 ± 2.0	38.3	41.1 ± 2.5	41.8	1.8 ± 2.8	2.7
100	1035.2 ± 0.7	1034.4 ± 0.5	1041.6	31.8 ± 1.1	35.6	39.1 ± 1.5	39.8	-0.5 ± 1.7	0.3
120	1042.6 ± 1.0	1040.9 ± 0.7	1048.8	29.2 ± 0.8	33.2	36.4 ± 1.1	38.1	-3.7 ± 1.3	-1.8
140	1049.0 ± 0.7	1047.3 ± 0.6	1055.6	27.8 ± 1.1	31.2	35.6 ± 1.6	36.6	-4.7 ± 1.9	-3.6
160	1054.5 ± 0.9	1052.7 ± 0.5	1062.0	26.5 ± 1.0	29.5	34.6 ± 1.4	35.2	-5.9 ± 1.7	-5.3
180	1059.4 ± 1.5	1058.3 ± 0.5	1068.1	25.3 ± 1.2	27.9	33.2 ± 1.8	34.0	-7.7 ± 2.2	-6.8
200	1065.2 ± 0.8	1063.6 ± 0.8	1074.0	24.3 ± 0.9	26.5	32.7 ± 1.2	32.9	-8.2 ± 1.5	-8.2

Table S211: Heat capacities at constant volume computed from MC simulations (c_V^{MC}), heat capacities at constant volume obtained from REFPROP[10] (c_V^{REFP}), heat capacities at constant pressure computed from MC simulations (c_P^{MC}), heat capacities at constant pressure obtained from REFPROP[10] (c_P^{REFP}), speed of sound computed from MC simulations (c^{MC}), speed of sound obtained from REFPROP[10] (c^{REFP}), viscosities computed from MD simulations (η^{MD}), and viscosities obtained from REFPROP[10] (η^{REFP}) of CO_2 rich ternary mixture with 1% impurity of N_2 and 1% impurity of CH_4 at 253 K and pressures ranging from 20 bar to 200 bar.

P/ [bar]	c_V^{MC}/ [J/mol K]	c_V^{REFP}/ [J/mol K]	c_P^{MC}/ [J/mol K]	c_P^{REFP}/ [J/mol K]	c^{MC}/ [m/s]	c^{REFP}/ [m/s]	η^{MD}/ [μPa s]	η^{REFP}/ [μPa s]
20	31.9	33.8	52.0 ± 0.4	57.2	225.3 ± 1.6	221.6	12.4 ± 1.3	13.0
40	40.1 ± 0.2	40.8	96.3 ± 2.5	93.3	778.4 ± 21.0	701.3	145.5 ± 7.1	135.6
60	40.6 ± 0.2	40.8	93.4 ± 2.4	91.2	793.4 ± 20.8	723.4	142.8 ± 7.7	139.6
80	40.6 ± 0.2	40.8	93.4 ± 3.5	89.4	807.8 ± 28.2	743.7	150.1 ± 0.9	143.4
100	40.7 ± 0.1	40.8	91.8 ± 2.3	87.9	827.3 ± 17.8	762.6	151.4 ± 8.9	147.1
120	40.6 ± 0.2	40.8	88.6 ± 2.1	86.7	846.3 ± 15.8	780.3	148.9 ± 12.4	150.7
140	40.8 ± 0.2	40.8	88.4 ± 2.6	85.5	862.7 ± 21.7	797.1	157.6 ± 4.1	154.1
160	40.8 ± 0.2	40.9	87.8 ± 2.3	84.5	878.1 ± 19.9	813.0	171.1 ± 14.3	157.5
180	40.8 ± 0.2	40.9	85.8 ± 2.8	83.7	886.2 ± 26.1	828.1	173.8 ± 18.8	160.8
200	40.6 ± 0.1	40.9	86.4 ± 1.9	82.9	906.7 ± 19.3	842.6	169.6 ± 14.4	164.0

Table S212: Densities computed from MC and MD simulations (ρ^{MC} and ρ^{MD}), densities obtained from REFPROP[10] (ρ^{REFP}), isothermal compressibilities computed from MC simulations (β_T^{MC}), isothermal compressibilities obtained from REFPROP[10] (β_T^{REFP}), thermal expansion coefficients computed from MC simulations (α_P^{MC}), thermal expansion coefficients obtained from REFPROP[10] (α_P^{REFP}), Joule Thomson coefficients computed from MC simulations ($\mu_{\mathrm{JT}}^{\mathrm{MC}}$), and Joule Thomson coefficients obtained from REFPROP[10] ($\mu_{\mathrm{JT}}^{\mathrm{REFP}}$) of CO_2 rich ternary mixture with 1% impurity of N_2 and 1% impurity of CH_4 at 273 K and pressures ranging from 20 bar to 200 bar.

P/ [bar]	ρ^{MC}/ [kg/m^3]	ρ^{MD}/ [kg/m^3]	ρ^{REFP}/ [kg/m^3]	β_T^{MC}/ [10^{-5}/bar]	β_T^{REFP}/ [10^{-5}/bar]	α_P^{MC}/ [10^{-4}/K]	α_P^{REFP}/ [10^{-4}/K]	$\mu_{\mathrm{JT}}^{\mathrm{MC}}$/ [$10^{-3}$ K/bar]	$\mu_{\mathrm{JT}}^{\mathrm{REFP}}$/ [$10^{-3}$ K/bar]
20	44.1	45.0 ± 0.2	45.0	5832.5 ± 49.2	5990.7	57.7 ± 0.5	61.6	1229.1 ± 31.4	1371.6
40	906.5 ± 1.8	119.2 ± 1.1	569.5	97.1 ± 4.9	-578.8	76.2 ± 3.0	549.6	43.6 ± 3.4	–
60	923.2 ± 2.2	919.0 ± 1.3	920.8	78.3 ± 4.1	92.1	64.8 ± 2.1	68.2	33.2 ± 2.5	38.4
80	935.2 ± 2.7	933.9 ± 1.2	936.3	64.3 ± 1.6	76.7	56.0 ± 1.4	60.1	24.1 ± 1.8	29.7
100	947.7 ± 1.3	944.9 ± 1.5	949.8	56.0 ± 1.2	66.2	51.1 ± 1.2	54.4	18.4 ± 1.5	23.1
120	957.3 ± 1.8	955.4 ± 1.1	961.6	50.3 ± 3.7	58.4	47.6 ± 3.3	50.1	14.2 ± 4.2	17.8
140	966.8 ± 1.6	965.4 ± 1.2	972.3	45.1 ± 0.7	52.4	43.7 ± 0.6	46.6	9.5 ± 0.8	13.4
160	975.3 ± 0.8	974.6 ± 1.0	982.1	43.8 ± 2.6	47.7	43.8 ± 2.7	43.8	9.3 ± 3.6	9.7
180	983.5 ± 1.1	982.9 ± 0.8	991.1	39.3 ± 1.6	43.8	40.6 ± 1.6	41.4	5.3 ± 2.1	6.6
200	992.4 ± 1.0	989.9 ± 0.5	999.5	35.7 ± 1.5	40.5	37.7 ± 1.8	39.4	1.5 ± 2.5	3.8

Table S213: Heat capacities at constant volume computed from MC simulations (c_V^{MC}), heat capacities at constant volume obtained from REFPROP[10] (c_V^{REFP}), heat capacities at constant pressure computed from MC simulations (c_P^{MC}), heat capacities at constant pressure obtained from REFPROP[10] (c_P^{REFP}), speed of sound computed from MC simulations (c^{MC}), speed of sound obtained from REFPROP[10] (c^{REFP}), viscosities computed from MD simulations (η^{MD}), and viscosities obtained from REFPROP[10] (η^{REFP}) of CO_2 rich ternary mixture with 1% impurity of N_2 and 1% impurity of CH_4 at 273 K and pressures ranging from 20 bar to 200 bar.

P/ [bar]	c_V^{MC}/ [J/mol K]	c_V^{REFP}/ [J/mol K]	c_P^{MC}/ [J/mol K]	c_P^{REFP}/ [J/mol K]	c^{MC}/ [m/s]	c^{REFP}/ [m/s]	η^{MD}/ [μPa s]	η^{REFP}/ [μPa s]
20	31.0	31.4	46.3 ± 0.2	48.2	241.3 ± 1.1	238.5	9.6 ± 7.5	14.0
40	39.9 ± 0.2	–	119.2 ± 2.4	–	582.4 ± 15.9	–	15.5 ± 0.9	43.7
60	39.7 ± 0.2	40.8	109.6 ± 1.4	106.0	617.4 ± 16.8	553.6	105.3 ± 1.3	99.9
80	39.8 ± 0.1	40.6	102.1 ± 1.7	100.5	652.8 ± 10.1	587.0	110.6 ± 3.1	104.5
100	40.0 ± 0.2	40.5	98.6 ± 1.3	96.5	681.3 ± 9.0	616.0	124.9 ± 19.2	108.7
120	39.8 ± 0.2	40.4	96.2 ± 3.8	93.5	708.4 ± 29.7	641.8	117.6 ± 3.5	112.5
140	39.9 ± 0.1	40.3	92.1 ± 0.9	91.0	727.1 ± 7.1	665.2	123.9 ± 5.0	116.2
160	39.9 ± 0.2	40.3	93.3 ± 3.7	89.0	739.0 ± 26.2	686.6	125.4 ± 7.1	119.6
180	39.9 ± 0.2	40.3	90.7 ± 2.2	87.4	766.7 ± 18.0	706.6	125.8 ± 6.0	123.0
200	40.1 ± 0.2	40.3	87.7 ± 2.6	85.9	785.9 ± 20.5	725.3	129.4 ± 11.9	126.2

Table S214: Densities computed from MC and MD simulations (ρ^{MC} and ρ^{MD}), densities obtained from REFPROP[10] (ρ^{REFP}), isothermal compressibilities computed from MC simulations (β_T^{MC}), isothermal compressibilities obtained from REFPROP[10] (β_T^{REFP}), thermal expansion coefficients computed from MC simulations (α_P^{MC}), thermal expansion coefficients obtained from REFPROP[10] (α_P^{REFP}), Joule Thomson coefficients computed from MC simulations ($\mu_{\mathrm{JT}}^{\mathrm{MC}}$), and Joule Thomson coefficients obtained from REFPROP[10] ($\mu_{\mathrm{JT}}^{\mathrm{REFP}}$) of CO_2 rich ternary mixture with 1% impurity of N_2 and 1% impurity of CH_4 at 293 K and pressures ranging from 20 bar to 200 bar.

P/ [bar]	ρ^{MC}/ [kg/m^3]	ρ^{MD}/ [kg/m^3]	ρ^{REFP}/ [kg/m^3]	β_T^{MC}/ [10^{-5}/bar]	β_T^{REFP}/ [10^{-5}/bar]	α_P^{MC}/ [10^{-4}/K]	α_P^{REFP}/ [10^{-4}/K]	$\mu_{\mathrm{JT}}^{\mathrm{MC}}$/ [$10^{-3}$ K/bar]	$\mu_{\mathrm{JT}}^{\mathrm{REFP}}$/ [$10^{-3}$ K/bar]
20	39.7	40.4 ± 0.1	40.3	5623.9 ± 30.4	5688.9	48.4 ± 0.2	50.3	1039.5 ± 16.9	1133.1
40	92.2 ± 0.1	94.5 ± 0.4	95.6	3444.7 ± 40.1	3591.8	80.3 ± 0.8	88.5	1066.7 ± 19.9	1131.0
60	186.2 ± 0.9	285.3 ± 75.8	281.8	4603.9 ± 391.3	138 167.3	277.8 ± 30.6	15 103.2	1058.8 ± 169.3	–
80	802.3 ± 3.6	796.7 ± 1.7	789.4	200.1 ± 18.5	280.8	110.0 ± 7.9	132.0	87.8 ± 10.0	111.0
100	830.4 ± 1.6	826.7 ± 2.5	824.9	131.1 ± 12.2	176.4	81.0 ± 6.6	94.9	61.5 ± 9.1	78.3
120	852.0 ± 2.0	846.3 ± 1.2	850.3	115.1 ± 6.9	131.1	74.7 ± 3.9	77.4	53.8 ± 5.5	59.4
140	867.2 ± 2.0	866.8 ± 0.9	870.4	91.6 ± 6.8	105.2	63.5 ± 4.1	66.7	41.2 ± 6.0	46.6
160	884.2 ± 1.7	882.1 ± 1.6	887.3	80.7 ± 4.4	88.3	59.4 ± 3.3	59.4	35.4 ± 4.8	37.2
180	896.3 ± 2.3	894.7 ± 0.7	902.0	68.0 ± 2.6	76.3	52.7 ± 1.3	53.9	27.0 ± 1.9	29.8
200	908.4 ± 1.1	906.1 ± 2.1	915.0	62.5 ± 2.1	67.4	50.3 ± 1.5	49.7	23.5 ± 2.2	23.8

Table S215: Heat capacities at constant volume computed from MC simulations (c_V^{MC}), heat capacities at constant volume obtained from REFPROP[10] (c_V^{REFP}), heat capacities at constant pressure computed from MC simulations (c_P^{MC}), heat capacities at constant pressure obtained from REFPROP[10] (c_P^{REFP}), speed of sound computed from MC simulations (c^{MC}), speed of sound obtained from REFPROP[10] (c^{REFP}), viscosities computed from MD simulations (η^{MD}), and viscosities obtained from REFPROP[10] (η^{REFP}) of CO_2 rich ternary mixture with 1% impurity of N_2 and 1% impurity of CH_4 at 293 K and pressures ranging from 20 bar to 200 bar.

P/ [bar]	c_V^{MC}/ [J/mol K]	c_V^{REFP}/ [J/mol K]	c_P^{MC}/ [J/mol K]	c_P^{REFP}/ [J/mol K]	c^{MC}/ [m/s]	c^{REFP}/ [m/s]	η^{MD}/ [μPa s]	η^{REFP}/ [μPa s]
20	30.9	31.1	44.3 ± 0.1	45.2	253.6 ± 0.7	251.8	4.5 ± 5.4	15.0
40	34.0 ± 0.1	35.1	59.9 ± 0.3	64.2	235.6 ± 1.5	230.9	16.4 ± 1.8	15.7
60	41.4 ± 0.3	–	157.8 ± 15.6	–	210.7 ± 13.8	–	19.3 ± 2.2	21.1
80	40.1 ± 0.3	42.2	137.5 ± 6.0	142.6	462.3 ± 23.7	390.3	73.5 ± 1.0	70.2
100	39.7 ± 0.2	41.2	117.2 ± 5.7	120.2	520.7 ± 27.3	447.8	86.6 ± 12.0	76.9
120	39.6 ± 0.1	40.7	113.1 ± 3.7	109.2	539.9 ± 18.6	490.8	90.0 ± 6.1	82.2
140	39.5 ± 0.1	40.4	104.8 ± 3.8	102.4	578.0 ± 24.0	526.0	93.0 ± 5.2	86.7
160	39.3 ± 0.2	40.2	103.0 ± 3.8	97.6	605.9 ± 20.0	556.4	92.6 ± 1.3	90.7
180	39.5 ± 0.1	40.1	97.9 ± 1.2	94.0	637.7 ± 12.9	583.4	95.7 ± 1.9	94.4
200	39.5 ± 0.2	40.0	96.5 ± 1.6	91.2	655.8 ± 12.4	607.9	100.1 ± 4.1	97.8

Table S216: Densities computed from MC and MD simulations (ρ^{MC} and ρ^{MD}), densities obtained from REFPROP[10] (ρ^{REFP}), isothermal compressibilities computed from MC simulations (β_T^{MC}), isothermal compressibilities obtained from REFPROP[10] (β_T^{REFP}), thermal expansion coefficients computed from MC simulations (α_P^{MC}), thermal expansion coefficients obtained from REFPROP[10] (α_P^{REFP}), Joule Thomson coefficients computed from MC simulations ($\mu_{\mathrm{JT}}^{\mathrm{MC}}$), and Joule Thomson coefficients obtained from REFPROP[10] ($\mu_{\mathrm{JT}}^{\mathrm{REFP}}$) of CO_2 rich ternary mixture with 1% impurity of N_2 and 1% impurity of CH_4 at 313 K and pressures ranging from 20 bar to 200 bar.

P/ [bar]	ρ^{MC}/ [kg/m^3]	ρ^{MD}/ [kg/m^3]	ρ^{REFP}/ [kg/m^3]	β_T^{MC}/ [10^{-5}/bar]	β_T^{REFP}/ [10^{-5}/bar]	α_P^{MC}/ [10^{-4}/K]	α_P^{REFP}/ [10^{-4}/K]	$\mu_{\mathrm{JT}}^{\mathrm{MC}}$/ [$10^{-3}$ K/bar]	$\mu_{\mathrm{JT}}^{\mathrm{REFP}}$/ [$10^{-3}$ K/bar]
20	36.3	36.9 ± 0.1	36.7	5429.9 ± 26.2	5509.2	41.9 ± 0.2	43.2	860.9 ± 16.5	953.5
40	80.5	82.2 ± 0.2	82.4	3065.6 ± 45.6	3170.3	58.7 ± 0.9	63.0	869.8 ± 28.0	944.7
60	139.0 ± 0.2	143.1 ± 0.7	145.5	2530.4 ± 35.4	2669.4	95.5 ± 1.6	107.1	873.9 ± 23.4	911.1
80	236.6 ± 0.7	254.7 ± 6.2	258.5	3097.6 ± 123.0	3380.6	236.0 ± 11.7	285.5	812.6 ± 57.6	–
100	556.8 ± 17.3	550.6 ± 6.3	554.2	2429.0 ± 445.5	2019.3	565.8 ± 76.7	457.7	345.7 ± 61.9	346.8
120	685.2 ± 6.5	677.4 ± 2.8	676.6	468.4 ± 64.0	528.6	172.8 ± 18.3	176.7	165.4 ± 24.2	180.4
140	729.9 ± 3.7	728.5 ± 2.3	730.8	279.4 ± 25.3	289.6	121.4 ± 8.5	116.5	119.7 ± 12.6	122.5
160	767.0 ± 1.9	761.9 ± 2.0	766.6	166.5 ± 5.2	199.7	83.8 ± 2.4	90.8	80.2 ± 4.1	91.7
180	789.7 ± 2.5	785.6 ± 1.9	793.8	145.9 ± 4.8	152.6	78.3 ± 1.8	76.0	71.2 ± 3.0	71.9
200	809.3 ± 2.4	807.2 ± 0.9	815.8	116.5 ± 8.1	123.7	67.7 ± 4.0	66.3	56.8 ± 6.6	57.7

Table S217: Heat capacities at constant volume computed from MC simulations (c_V^{MC}), heat capacities at constant volume obtained from REFPROP[10] (c_V^{REFP}), heat capacities at constant pressure computed from MC simulations (c_P^{MC}), heat capacities at constant pressure obtained from REFPROP[10] (c_P^{REFP}), speed of sound computed from MC simulations (c^{MC}), speed of sound obtained from REFPROP[10] (c^{REFP}), viscosities computed from MD simulations (η^{MD}), and viscosities obtained from REFPROP[10] (η^{REFP}) of CO_2 rich ternary mixture with 1% impurity of N_2 and 1% impurity of CH_4 at 313 K and pressures ranging from 20 bar to 200 bar.

P/ [bar]	c_V^{MC}/ [J/mol K]	c_V^{REFP}/ [J/mol K]	c_P^{MC}/ [J/mol K]	c_P^{REFP}/ [J/mol K]	c^{MC}/ [m/s]	c^{REFP}/ [m/s]	η^{MD}/ [μPa s]	η^{REFP}/ [μPa s]
20	31.2	31.3	43.4 ± 0.1	43.9	265.6 ± 0.7	263.4	11.1 ± 3.5	15.9
40	33.1	33.6	52.2 ± 0.3	54.3	252.8 ± 2.0	248.6	16.3 ± 1.8	16.5
60	35.9 ± 0.2	37.0	71.4 ± 0.8	77.3	237.7 ± 2.2	231.8	18.0 ± 1.1	17.7
80	40.2 ± 0.3	–	144.8 ± 6.0	–	221.5 ± 6.4	–	21.0 ± 0.9	21.2
100	41.7 ± 0.2	46.8	378.9 ± 38.9	302.1	259.4 ± 27.5	240.1	39.6 ± 3.5	40.3
120	40.0 ± 0.3	42.7	169.5 ± 11.4	161.8	363.3 ± 27.7	325.5	57.8 ± 6.4	53.8
140	39.8 ± 0.2	41.3	139.6 ± 6.3	128.8	414.8 ± 21.1	383.7	60.8 ± 1.5	61.3
160	39.5 ± 0.2	40.7	115.1 ± 2.3	114.0	477.8 ± 8.9	428.0	68.5 ± 2.9	66.8
180	39.3 ± 0.1	40.3	112.5 ± 1.7	105.4	498.3 ± 9.0	464.5	71.2 ± 10.3	71.5
200	39.3 ± 0.1	40.1	105.9 ± 3.8	99.5	534.7 ± 21.0	496.0	83.3 ± 17.1	75.5

S16.7 Data of thermodynamics and transport properties of CO_2 rich ternary mixture with 2 mole% impurity of Ar and 2 mole% impurity of N_2

Table S218: Densities computed from MC and MD simulations (ρ^{MC} and ρ^{MD}), densities obtained from REFPROP[10] (ρ^{REFP}), isothermal compressibilities computed from MC simulations (β_T^{MC}), isothermal compressibilities obtained from REFPROP[10] (β_T^{REFP}), thermal expansion coefficients computed from MC simulations (α_P^{MC}), thermal expansion coefficients obtained from REFPROP[10] (α_P^{REFP}), Joule Thomson coefficients computed from MC simulations (μ_{JT}^{MC}), and Joule Thomson coefficients obtained from REFPROP[10] (μ_{JT}^{REFP}) of CO_2 rich ternary mixture with 2% impurity of Ar and 2% impurity of N_2 at 253 K and pressures ranging from 20 bar to 200 bar.

P/ [bar]	ρ^{MC}/ [kg/m^3]	ρ^{MD}/ [kg/m^3]	ρ^{REFP}/ [kg/m^3]	β_T^{MC}/ [10^{-5}/bar]	β_T^{REFP}/ [10^{-5}/bar]	α_P^{MC}/ [10^{-4}/K]	α_P^{REFP}/ [10^{-4}/K]	μ_{JT}^{MC}/ [10^{-3} K/bar]	μ_{JT}^{REFP}/ [10^{-3} K/bar]
20	50.0	51.0 ± 0.1	51.4	6332.3 ± 22.7	6505.5	75.1 ± 0.3	81.4	1540.3 ± 13.8	1652.0
40	1005.3 ± 1.5	1001.6 ± 1.1	958.3	43.9 ± 2.3	94.5	47.3 ± 2.1	76.8	8.9 ± 2.4	–
60	1013.0 ± 1.3	1010.3 ± 0.4	1016.6	40.8 ± 1.8	46.7	45.2 ± 1.7	47.0	6.5 ± 1.9	8.9
80	1021.0 ± 1.2	1019.6 ± 0.8	1025.7	38.4 ± 1.0	42.5	44.1 ± 1.1	44.2	5.2 ± 1.2	5.6
100	1028.8 ± 1.1	1027.0 ± 1.2	1034.1	35.2 ± 2.1	39.2	41.3 ± 2.3	41.9	2.0 ± 2.7	2.8
120	1034.7 ± 0.8	1034.0 ± 0.8	1041.9	32.9 ± 1.2	36.3	39.2 ± 1.5	39.8	-0.4 ± 1.8	0.4
140	1042.8 ± 0.9	1040.6 ± 0.6	1049.3	30.9 ± 1.3	33.9	37.9 ± 1.7	38.1	-1.9 ± 2.0	-1.7
160	1048.1 ± 1.5	1047.7 ± 1.0	1056.2	29.3 ± 0.9	31.9	36.6 ± 0.9	36.6	-3.5 ± 1.0	-3.6
180	1055.0 ± 0.9	1052.8 ± 0.7	1062.7	27.4 ± 0.7	30.1	35.0 ± 1.0	35.2	-5.4 ± 1.2	-5.4
200	1060.7 ± 1.0	1059.1 ± 0.5	1069.0	25.5 ± 0.5	28.5	32.8 ± 0.5	34.0	-8.3 ± 0.7	-6.9

Table S219: Heat capacities at constant volume computed from MC simulations (c_V^{MC}), heat capacities at constant volume obtained from REFPROP[10] (c_V^{REFP}), heat capacities at constant pressure computed from MC simulations (c_P^{MC}), heat capacities at constant pressure obtained from REFPROP[10] (c_P^{REFP}), speed of sound computed from MC simulations (c^{MC}), speed of sound obtained from REFPROP[10] (c^{REFP}), viscosities computed from MD simulations (η^{MD}), and viscosities obtained from REFPROP[10] (η^{REFP}) of CO_2 rich ternary mixture with 2% impurity of Ar and 2% impurity of N_2 at 253 K and pressures ranging from 20 bar to 200 bar.

P/ [bar]	c_V^{MC}/ [J/mol K]	c_V^{REFP}/ [J/mol K]	c_P^{MC}/ [J/mol K]	c_P^{REFP}/ [J/mol K]	c^{MC}/ [m/s]	c^{REFP}/ [m/s]	η^{MD}/ [μPa s]	η^{REFP}/ [μPa s]
20	31.3 ± 0.1	32.6	50.9 ± 0.1	54.4	226.7 ± 0.5	223.6	9.6 ± 2.2	13.1
40	40.0 ± 0.1	–	96.1 ± 2.4	–	737.6 ± 21.6	–	135.2 ± 3.3	111.0
60	39.9 ± 0.3	40.2	94.5 ± 2.0	91.7	757.8 ± 19.0	693.1	137.4 ± 7.3	132.7
80	40.1 ± 0.2	40.2	94.8 ± 1.5	89.6	776.1 ± 11.7	714.9	140.0 ± 4.1	136.6
100	40.0 ± 0.2	40.2	91.9 ± 2.7	87.9	797.2 ± 26.3	735.0	144.7 ± 5.5	140.3
120	40.2 ± 0.3	40.2	90.2 ± 2.1	86.5	812.7 ± 18.3	753.8	147.3 ± 4.9	143.9
140	40.2 ± 0.2	40.2	89.3 ± 2.4	85.2	830.6 ± 20.7	771.5	156.7 ± 21.1	147.4
160	40.3 ± 0.3	40.2	88.2 ± 1.2	84.1	844.7 ± 14.5	788.1	155.1 ± 5.3	150.8
180	40.3 ± 0.2	40.3	87.0 ± 1.5	83.1	864.6 ± 13.0	804.0	166.2 ± 13.9	154.1
200	40.4 ± 0.2	40.3	84.1 ± 0.8	82.3	876.5 ± 9.2	819.1	158.9 ± 8.4	157.4

Table S220: Densities computed from MC and MD simulations (ρ^{MC} and ρ^{MD}), densities obtained from REFPROP[10] (ρ^{REFP}), isothermal compressibilities computed from MC simulations (β_T^{MC}), isothermal compressibilities obtained from REFPROP[10] (β_T^{REFP}), thermal expansion coefficients computed from MC simulations (α_P^{MC}), thermal expansion coefficients obtained from REFPROP[10] (α_P^{REFP}), Joule Thomson coefficients computed from MC simulations (μ_{JT}^{MC}), and Joule Thomson coefficients obtained from REFPROP[10] (μ_{JT}^{REFP}) of CO_2 rich ternary mixture with 2% impurity of Ar and 2% impurity of N_2 at 273 K and pressures ranging from 20 bar to 200 bar.

P/ [bar]	ρ^{MC}/ [kg/m^3]	ρ^{MD}/ [kg/m^3]	ρ^{REFP}/ [kg/m^3]	β_T^{MC}/ [10^{-5}/bar]	β_T^{REFP}/ [10^{-5}/bar]	α_P^{MC}/ [10^{-4}/K]	α_P^{REFP}/ [10^{-4}/K]	μ_{JT}^{MC}/ [10^{-3} K/bar]	μ_{JT}^{REFP}/ [10^{-3} K/bar]
20	43.9	44.8 ± 0.2	44.7	5807.9 ± 30.3	5935.9	57.0 ± 0.4	60.2	1210.2 ± 26.4	1333.3
40	110.1 ± 0.3	114.7 ± 0.4	251.2	4318.5 ± 219.8	-3548.2	133.1 ± 8.3	-514.9	1285.6 ± 124.7	–
60	905.0 ± 2.6	901.0 ± 0.8	903.4	92.8 ± 7.5	112.9	71.1 ± 4.6	77.2	40.6 ± 5.6	48.6
80	921.4 ± 1.5	918.4 ± 2.4	921.8	76.6 ± 4.9	90.5	62.3 ± 2.9	66.3	31.5 ± 3.6	37.2
100	933.8 ± 1.1	932.7 ± 0.6	937.2	69.0 ± 4.5	76.1	58.8 ± 3.5	58.9	27.3 ± 4.4	28.9
120	947.8 ± 1.7	944.6 ± 1.7	950.6	58.2 ± 3.0	66.1	51.7 ± 2.5	53.6	19.4 ± 3.2	22.5
140	956.7 ± 1.1	956.0 ± 0.9	962.4	51.2 ± 1.2	58.5	46.9 ± 0.8	49.4	13.7 ± 1.1	17.3
160	966.2 ± 1.6	965.0 ± 1.2	973.2	48.4 ± 2.3	52.7	45.6 ± 2.0	46.1	12.0 ± 2.7	13.1
180	975.9 ± 0.6	973.3 ± 0.7	983.0	44.0 ± 1.2	48.0	43.3 ± 1.2	43.4	8.8 ± 1.6	9.4
200	985.0 ± 1.1	982.2 ± 1.0	992.1	40.0 ± 1.5	44.1	40.6 ± 1.3	41.1	5.4 ± 1.8	6.3

Table S221: Heat capacities at constant volume computed from MC simulations (c_V^{MC}), heat capacities at constant volume obtained from REFPROP[10] (c_V^{REFP}), heat capacities at constant pressure computed from MC simulations (c_P^{MC}), heat capacities at constant pressure obtained from REFPROP[10] (c_P^{REFP}), speed of sound computed from MC simulations (c^{MC}), speed of sound obtained from REFPROP[10] (c^{REFP}), viscosities computed from MD simulations (η^{MD}), and viscosities obtained from REFPROP[10] (η^{REFP}) of CO_2 rich ternary mixture with 2% impurity of Ar and 2% impurity of N_2 at 273 K and pressures ranging from 20 bar to 200 bar.

P/ [bar]	c_V^{MC}/ [J/mol K]	c_V^{REFP}/ [J/mol K]	c_P^{MC}/ [J/mol K]	c_P^{REFP}/ [J/mol K]	c^{MC}/ [m/s]	c^{REFP}/ [m/s]	η^{MD}/ [μPa s]	η^{REFP}/ [μPa s]
20	30.5	30.8	45.6 ± 0.2	47.0	242.3 ± 0.8	239.8	4.9 ± 3.2	14.2
40	36.4 ± 0.3	–	81.1 ± 3.7	–	216.4 ± 7.4	–	15.1 ± 2.0	18.8
60	39.3 ± 0.1	40.4	111.8 ± 4.1	110.0	581.9 ± 25.9	516.7	97.8 ± 2.3	93.8
80	39.3 ± 0.2	40.1	105.5 ± 2.1	102.8	616.7 ± 20.7	554.3	109.1 ± 5.8	98.7
100	39.3 ± 0.1	39.9	103.6 ± 3.5	97.9	639.7 ± 23.6	586.0	107.6 ± 5.9	103.1
120	39.4 ± 0.1	39.8	97.3 ± 2.6	94.2	668.8 ± 19.3	613.8	115.7 ± 7.0	107.1
140	39.4 ± 0.2	39.7	92.9 ± 0.9	91.4	693.8 ± 9.1	638.8	110.6 ± 4.0	110.9
160	39.5 ± 0.2	39.7	92.6 ± 2.2	89.1	708.3 ± 19.0	661.5	119.0 ± 6.7	114.4
180	39.4 ± 0.1	39.7	91.5 ± 1.4	87.2	735.2 ± 11.7	682.5	124.2 ± 2.4	117.8
200	39.4 ± 0.2	39.7	89.3 ± 1.5	85.7	757.7 ± 15.5	702.1	120.6 ± 5.7	121.0

Table S222: Densities computed from MC and MD simulations (ρ^{MC} and ρ^{MD}), densities obtained from REFPROP[10] (ρ^{REFP}), isothermal compressibilities computed from MC simulations (β_T^{MC}), isothermal compressibilities obtained from REFPROP[10] (β_T^{REFP}), thermal expansion coefficients computed from MC simulations (α_P^{MC}), thermal expansion coefficients obtained from REFPROP[10] (α_P^{REFP}), Joule Thomson coefficients computed from MC simulations ($\mu_{\mathrm{JT}}^{\mathrm{MC}}$), and Joule Thomson coefficients obtained from REFPROP[10] ($\mu_{\mathrm{JT}}^{\mathrm{REFP}}$) of CO_2 rich ternary mixture with 2% impurity of Ar and 2% impurity of N_2 at 293 K and pressures ranging from 20 bar to 200 bar.

P/ [bar]	ρ^{MC}/ [kg/m^3]	ρ^{MD}/ [kg/m^3]	ρ^{REFP}/ [kg/m^3]	β_T^{MC}/ [10^{-5}/bar]	β_T^{REFP}/ [10^{-5}/bar]	α_P^{MC}/ [10^{-4}/K]	α_P^{REFP}/ [10^{-4}/K]	$\mu_{\mathrm{JT}}^{\mathrm{MC}}$/ [$10^{-3}$ K/bar]	$\mu_{\mathrm{JT}}^{\mathrm{REFP}}$/ [$10^{-3}$ K/bar]
20	39.5	40.4 ± 0.1	40.1	5608.2 ± 26.1	5656.4	48.0 ± 0.3	49.5	1024.1 ± 20.6	1104.9
40	91.2 ± 0.1	93.2 ± 0.4	94.2	3351.1 ± 30.1	3499.4	76.3 ± 0.8	83.7	1024.0 ± 19.0	1097.3
60	176.1 ± 0.5	188.7 ± 2.3	192.0	3695.1 ± 113.9	4359.1	199.2 ± 8.1	264.9	1019.7 ± 60.4	1022.6
80	762.1 ± 5.4	759.2 ± 4.1	751.7	323.7 ± 40.6	443.8	150.8 ± 14.4	180.0	122.1 ± 16.7	148.7
100	800.8 ± 1.7	801.7 ± 1.9	799.9	197.1 ± 7.8	229.5	106.4 ± 4.6	111.7	87.0 ± 6.3	96.9
120	830.5 ± 0.8	829.6 ± 0.7	830.9	144.8 ± 6.7	159.1	86.7 ± 3.2	86.7	67.5 ± 4.4	71.6
140	850.8 ± 1.3	850.4 ± 1.3	854.4	110.4 ± 8.2	123.1	71.3 ± 4.3	72.8	51.2 ± 6.2	55.5
160	868.2 ± 1.3	867.0 ± 1.3	873.6	93.8 ± 3.5	100.9	64.2 ± 2.2	63.8	42.5 ± 3.3	44.0
180	884.3 ± 1.0	881.3 ± 0.4	890.0	76.7 ± 2.5	85.8	56.0 ± 1.9	57.3	32.1 ± 2.9	35.3
200	898.0 ± 1.0	895.8 ± 1.4	904.3	66.1 ± 2.0	74.8	50.6 ± 1.6	52.4	24.8 ± 2.5	28.3

Table S223: Heat capacities at constant volume computed from MC simulations (c_V^{MC}), heat capacities at constant volume obtained from REFPROP[10] (c_V^{REFP}), heat capacities at constant pressure computed from MC simulations (c_P^{MC}), heat capacities at constant pressure obtained from REFPROP[10] (c_P^{REFP}), speed of sound computed from MC simulations (c^{MC}), speed of sound obtained from REFPROP[10] (c^{REFP}), viscosities computed from MD simulations (η^{MD}), and viscosities obtained from REFPROP[10] (η^{REFP}) of CO_2 rich ternary mixture with 2% impurity of Ar and 2% impurity of N_2 at 293 K and pressures ranging from 20 bar to 200 bar.

P/ [bar]	c_V^{MC}/ [J/mol K]	c_V^{REFP}/ [J/mol K]	c_P^{MC}/ [J/mol K]	c_P^{REFP}/ [J/mol K]	c^{MC}/ [m/s]	c^{REFP}/ [m/s]	η^{MD}/ [μPa s]	η^{REFP}/ [μPa s]
20	30.4	30.6	43.7 ± 0.1	44.4	254.4 ± 0.7	252.9	9.1 ± 3.5	15.2
40	33.3	34.2	57.7 ± 0.3	61.3	237.9 ± 1.2	233.4	15.4 ± 1.0	15.8
60	39.1 ± 0.5	43.1	117.5 ± 3.8	150.2	214.8 ± 5.0	204.2	18.1 ± 1.1	18.0
80	40.1 ± 0.5	42.7	160.2 ± 9.3	166.8	402.7 ± 28.0	342.2	66.0 ± 1.0	63.3
100	39.6 ± 0.2	41.0	132.6 ± 4.4	127.9	460.7 ± 12.0	412.3	76.4 ± 5.1	71.4
120	39.2 ± 0.1	40.3	119.7 ± 2.5	112.9	504.1 ± 12.8	460.5	80.7 ± 0.7	77.3
140	39.1 ± 0.2	39.9	108.9 ± 3.5	104.3	544.7 ± 21.9	498.8	85.8 ± 4.0	82.1
160	39.1 ± 0.2	39.7	104.3 ± 2.2	98.7	572.4 ± 12.5	531.3	92.4 ± 4.7	86.3
180	39.2 ± 0.1	39.5	98.3 ± 2.2	94.6	608.1 ± 12.1	559.8	95.3 ± 7.8	90.2
200	38.8 ± 0.1	39.4	94.4 ± 1.9	91.4	640.2 ± 11.9	585.4	95.7 ± 3.3	93.7

Table S224: Densities computed from MC and MD simulations (ρ^{MC} and ρ^{MD}), densities obtained from REFPROP[10] (ρ^{REFP}), isothermal compressibilities computed from MC simulations (β_T^{MC}), isothermal compressibilities obtained from REFPROP[10] (β_T^{REFP}), thermal expansion coefficients computed from MC simulations (α_P^{MC}), thermal expansion coefficients obtained from REFPROP[10] (α_P^{REFP}), Joule Thomson coefficients computed from MC simulations (μ_{JT}^{MC}), and Joule Thomson coefficients obtained from REFPROP[10] (μ_{JT}^{REFP}) of CO_2 rich ternary mixture with 2% impurity of Ar and 2% impurity of N_2 at 313 K and pressures ranging from 20 bar to 200 bar.

P/ [bar]	ρ^{MC}/ [kg/m^3]	ρ^{MD}/ [kg/m^3]	ρ^{REFP}/ [kg/m^3]	β_T^{MC}/ [10^{-5}/bar]	β_T^{REFP}/ [10^{-5}/bar]	α_P^{MC}/ [10^{-4}/K]	α_P^{REFP}/ [10^{-4}/K]	μ_{JT}^{MC}/ [10^{-3} K/bar]	μ_{JT}^{REFP}/ [10^{-3} K/bar]
20	36.2	36.9	36.6	5453.8 ± 23.7	5487.1	41.8 ± 0.2	42.7	865.7 ± 15.5	931.7
40	79.9 ± 0.1	81.5 ± 0.1	81.7	3088.5 ± 17.0	3128.5	58.3 ± 0.4	61.1	876.9 ± 15.3	919.5
60	136.6 ± 0.1	141.2 ± 0.5	142.4	2466.8 ± 24.4	2565.3	90.5 ± 1.3	99.0	856.9 ± 20.9	885.6
80	223.6 ± 0.7	236.8 ± 3.3	240.6	2733.7 ± 75.4	2854.2	192.9 ± 6.7	216.4	800.6 ± 40.3	781.5
100	443.1 ± 8.6	455.7 ± 10.0	167.6	3424.9 ± 159.0	2551.5	593.7 ± 42.0	121.2	472.3 ± 51.0	864.4
120	632.6 ± 5.3	627.3 ± 4.5	633.7	690.1 ± 38.0	752.2	216.4 ± 10.2	218.5	210.6 ± 13.7	224.1
140	699.5 ± 5.8	698.1 ± 2.8	702.6	360.5 ± 36.4	365.4	140.3 ± 11.8	133.0	143.4 ± 17.5	145.4
160	742.7 ± 1.7	738.3 ± 1.2	745.0	220.2 ± 15.1	238.0	100.1 ± 5.8	99.8	101.5 ± 9.3	106.4
180	771.1 ± 1.3	769.0 ± 2.3	776.0	162.5 ± 8.5	176.2	81.8 ± 3.2	81.9	78.6 ± 5.3	82.4
200	792.1 ± 1.6	791.5 ± 0.8	800.7	133.3 ± 7.3	139.9	71.6 ± 3.7	70.6	64.8 ± 6.4	65.7

Table S225: Heat capacities at constant volume computed from MC simulations (c_V^{MC}), heat capacities at constant volume obtained from REFPROP[10] (c_V^{REFP}), heat capacities at constant pressure computed from MC simulations (c_P^{MC}), heat capacities at constant pressure obtained from REFPROP[10] (c_P^{REFP}), speed of sound computed from MC simulations (c^{MC}), speed of sound obtained from REFPROP[10] (c^{REFP}), viscosities computed from MD simulations (η^{MD}), and viscosities obtained from REFPROP[10] (η^{REFP}) of CO_2 rich ternary mixture with 2% impurity of Ar and 2% impurity of N_2 at 313 K and pressures ranging from 20 bar to 200 bar.

P/ [bar]	c_V^{MC}/ [J/mol K]	c_V^{REFP}/ [J/mol K]	c_P^{MC}/ [J/mol K]	c_P^{REFP}/ [J/mol K]	c^{MC}/ [m/s]	c^{REFP}/ [m/s]	η^{MD}/ [μPa s]	η^{REFP}/ [μPa s]
20	30.7	30.8	42.8 ± 0.1	43.2	265.7 ± 0.6	264.3	10.7 ± 2.9	16.1
40	32.5	33.0	51.4 ± 0.2	52.9	253.0 ± 0.9	250.4	19.3 ± 3.2	16.7
60	35.0	36.0	68.2 ± 0.6	72.6	240.6 ± 1.6	235.1	18.0 ± 0.9	17.9
80	38.5 ± 0.2	40.8	122.7 ± 3.4	133.9	228.2 ± 4.5	218.6	22.6 ± 2.7	20.7
100	42.8 ± 0.7	37.2	366.7 ± 27.7	84.1	237.7 ± 10.9	229.9	31.2 ± 1.9	18.5
120	40.3 ± 0.6	42.6	189.0 ± 6.2	179.3	327.9 ± 10.8	297.0	53.9 ± 6.8	48.3
140	39.4 ± 0.2	41.0	147.4 ± 8.1	135.0	385.1 ± 22.2	358.3	57.3 ± 4.8	56.9
160	39.1 ± 0.1	40.2	123.4 ± 4.2	116.9	439.4 ± 16.8	405.0	72.9 ± 10.6	62.9
180	38.8 ± 0.2	39.8	112.2 ± 2.4	106.8	480.1 ± 13.6	443.1	69.6 ± 6.3	67.8
200	38.8 ± 0.1	39.5	105.6 ± 3.5	100.2	508.0 ± 16.3	475.8	74.2 ± 2.6	72.0

S16.8 Data of thermodynamics and transport properties of CO_2 rich ternary mixture with 2 mole% impurity of N_2 and 2 mole% impurity of H_2

Table S226: Densities computed from MC and MD simulations (ρ^{MC} and ρ^{MD}), densities obtained from REFPROP[10] (ρ^{REFP}), isothermal compressibilities computed from MC simulations (β_T^{MC}), isothermal compressibilities obtained from REFPROP[10] (β_T^{REFP}), thermal expansion coefficients computed from MC simulations (α_P^{MC}), thermal expansion coefficients obtained from REFPROP[10] (α_P^{REFP}), Joule Thomson coefficients computed from MC simulations (μ_{JT}^{MC}), and Joule Thomson coefficients obtained from REFPROP[10] (μ_{JT}^{REFP}) of CO_2 rich ternary mixture with 2% impurity of N_2 and 2% impurity of H_2 at 253 K and pressures ranging from 20 bar to 200 bar.

P/ [bar]	ρ^{MC}/ [kg/m^3]	ρ^{MD}/ [kg/m^3]	ρ^{REFP}/ [kg/m^3]	β_T^{MC}/ [10^{-5}/bar]	β_T^{REFP}/ [10^{-5}/bar]	α_P^{MC}/ [10^{-4}/K]	α_P^{REFP}/ [10^{-4}/K]	μ_{JT}^{MC}/ [10^{-3} K/bar]	μ_{JT}^{REFP}/ [10^{-3} K/bar]
20	48.9	49.9 ± 0.1	50.3	6235.7 ± 58.1	6474.3	73.2 ± 0.8	80.4	1480.3 ± 34.1	1630.3
40	984.2 ± 1.7	979.7 ± 0.4	679.0	47.6 ± 0.9	−51.7	50.2 ± 1.1	51.5	11.9 ± 1.2	–
60	992.1 ± 1.1	989.5 ± 1.3	947.6	45.1 ± 2.5	78.5	48.3 ± 2.0	66.2	9.9 ± 2.3	–
80	999.8 ± 1.1	996.9 ± 1.6	996.0	40.9 ± 1.7	44.8	45.2 ± 1.7	45.1	6.5 ± 2.0	6.8
100	1008.1 ± 1.3	1006.3 ± 0.8	1004.6	37.7 ± 1.5	41.1	42.8 ± 2.2	42.6	3.8 ± 2.5	3.8
120	1015.6 ± 0.8	1013.3 ± 0.4	1012.6	34.4 ± 1.8	38.0	39.7 ± 1.8	40.5	0.2 ± 2.1	1.2
140	1021.2 ± 0.6	1020.8 ± 0.5	1020.0	33.9 ± 1.1	35.4	40.2 ± 1.0	38.6	0.8 ± 1.1	−1.1
160	1028.7 ± 0.7	1027.2 ± 0.6	1027.0	29.7 ± 0.9	33.1	36.2 ± 1.1	37.0	-4.0 ± 1.4	−3.2
180	1034.1 ± 0.8	1033.7 ± 1.3	1033.7	28.3 ± 0.7	31.2	35.1 ± 1.1	35.6	-5.4 ± 1.3	−5.0
200	1040.6 ± 0.6	1039.2 ± 0.6	1040.0	27.1 ± 0.9	29.5	34.2 ± 1.5	34.4	-6.6 ± 1.8	−6.6

Table S227: Heat capacities at constant volume computed from MC simulations (c_V^{MC}), heat capacities at constant volume obtained from REFPROP[10] (c_V^{REFP}), heat capacities at constant pressure computed from MC simulations (c_P^{MC}), heat capacities at constant pressure obtained from REFPROP[10] (c_P^{REFP}), speed of sound computed from MC simulations (c^{MC}), speed of sound obtained from REFPROP[10] (c^{REFP}), viscosities computed from MD simulations (η^{MD}), and viscosities obtained from REFPROP[10] (η^{REFP}) of CO_2 rich ternary mixture with 2% impurity of N_2 and 2% impurity of H_2 at 253 K and pressures ranging from 20 bar to 200 bar.

P/ [bar]	c_V^{MC}/ [J/mol K]	c_V^{REFP}/ [J/mol K]	c_P^{MC}/ [J/mol K]	c_P^{REFP}/ [J/mol K]	c^{MC}/ [m/s]	c^{REFP}/ [m/s]	η^{MD}/ [μPa s]	η^{REFP}/ [μPa s]
20	31.5 ± 0.1	32.5	50.5 ± 0.3	54.0	229.4 ± 1.2	225.8	6.8 ± 1.6	13.0
40	40.1 ± 0.2	–	98.6 ± 1.5	–	724.7 ± 8.6	–	126.6 ± 2.3	59.5
60	40.2 ± 0.2	–	97.0 ± 2.2	–	734.7 ± 22.0	–	130.3 ± 2.8	110.9
80	40.4 ± 0.1	40.2	94.5 ± 2.0	89.6	756.3 ± 17.9	706.9	138.4 ± 1.9	129.2
100	40.3 ± 0.1	40.2	92.7 ± 3.4	87.8	777.7 ± 21.1	727.8	133.4 ± 11.4	132.9
120	40.2 ± 0.3	40.2	89.5 ± 2.0	86.3	798.2 ± 22.6	747.3	152.1 ± 9.0	136.4
140	40.3 ± 0.4	40.2	90.8 ± 1.0	85.0	806.7 ± 14.0	765.5	159.1 ± 12.6	139.8
160	40.3 ± 0.3	40.2	86.9 ± 1.8	83.8	839.7 ± 15.3	782.7	155.1 ± 2.7	143.1
180	40.4 ± 0.1	40.2	86.0 ± 1.9	82.8	852.9 ± 13.6	799.0	164.2 ± 38.5	146.3
200	40.6 ± 0.2	40.2	85.1 ± 2.5	81.9	863.1 ± 18.7	814.5	173.0 ± 20.5	149.5

Table S228: Densities computed from MC and MD simulations (ρ^{MC} and ρ^{MD}), densities obtained from REFPROP[10] (ρ^{REFP}), isothermal compressibilities computed from MC simulations (β_T^{MC}), isothermal compressibilities obtained from REFPROP[10] (β_T^{REFP}), thermal expansion coefficients computed from MC simulations (α_P^{MC}), thermal expansion coefficients obtained from REFPROP[10] (α_P^{REFP}), Joule Thomson coefficients computed from MC simulations (μ_{JT}^{MC}), and Joule Thomson coefficients obtained from REFPROP[10] (μ_{JT}^{REFP}) of CO_2 rich ternary mixture with 2% impurity of N_2 and 2% impurity of H_2 at 273 K and pressures ranging from 20 bar to 200 bar.

P/ [bar]	ρ^{MC}/ [kg/m^3]	ρ^{MD}/ [kg/m^3]	ρ^{REFP}/ [kg/m^3]	β_T^{MC}/ [10^{-5}/bar]	β_T^{REFP}/ [10^{-5}/bar]	α_P^{MC}/ [10^{-4}/K]	α_P^{REFP}/ [10^{-4}/K]	μ_{JT}^{MC}/ [10^{-3} K/bar]	μ_{JT}^{REFP}/ [10^{-3} K/bar]
20	43.0	43.9 ± 0.1	43.9	5797.4 ± 55.6	5922.9	56.8 ± 0.5	59.8	1199.3 ± 29.7	1316.8
40	107.3 ± 0.2	112.1 ± 0.9	224.8	4162.4 ± 63.3	-8081.5	125.7 ± 2.7	-883.7	1243.0 ± 41.3	–
60	880.7 ± 2.5	877.7 ± 1.1	773.4	104.7 ± 5.9	928.3	76.6 ± 3.1	387.8	46.0 ± 3.7	–
80	896.0 ± 2.3	895.9 ± 1.9	892.5	86.4 ± 3.7	97.5	67.1 ± 2.3	68.7	36.7 ± 2.8	40.6
100	910.6 ± 1.0	909.2 ± 0.5	908.4	74.2 ± 3.8	81.1	60.8 ± 2.3	60.6	29.9 ± 2.9	31.4
120	924.7 ± 1.2	921.9 ± 0.9	922.2	63.0 ± 4.0	69.8	54.4 ± 3.2	54.8	22.7 ± 4.1	24.4
140	935.7 ± 1.0	933.7 ± 0.5	934.3	56.2 ± 1.8	61.5	50.0 ± 1.6	50.4	17.5 ± 2.0	18.8
160	944.7 ± 1.6	944.0 ± 1.0	945.3	52.2 ± 0.9	55.1	47.8 ± 0.9	46.9	14.7 ± 1.2	14.2
180	953.9 ± 1.4	953.3 ± 1.0	955.2	46.7 ± 0.6	50.0	44.1 ± 0.7	44.0	10.0 ± 1.0	10.3
200	964.8 ± 1.2	962.2 ± 0.2	964.4	44.0 ± 3.6	45.9	43.6 ± 3.6	41.6	9.2 ± 4.7	7.0

Table S229: Heat capacities at constant volume computed from MC simulations (c_V^{MC}), heat capacities at constant volume obtained from REFPROP[10] (c_V^{REFP}), heat capacities at constant pressure computed from MC simulations (c_P^{MC}), heat capacities at constant pressure obtained from REFPROP[10] (c_P^{REFP}), speed of sound computed from MC simulations (c^{MC}), speed of sound obtained from REFPROP[10] (c^{REFP}), viscosities computed from MD simulations (η^{MD}), and viscosities obtained from REFPROP[10] (η^{REFP}) of CO_2 rich ternary mixture with 2% impurity of N_2 and 2% impurity of H_2 at 273 K and pressures ranging from 20 bar to 200 bar.

P/ [bar]	c_V^{MC}/ [J/mol K]	c_V^{REFP}/ [J/mol K]	c_P^{MC}/ [J/mol K]	c_P^{REFP}/ [J/mol K]	c^{MC}/ [m/s]	c^{REFP}/ [m/s]	η^{MD}/ [μPa s]	η^{REFP}/ [μPa s]
20	30.6	30.9	45.7 ± 0.1	47.0	244.8 ± 1.2	242.0	3.5 ± 5.1	14.0
40	36.4 ± 0.1	–	78.2 ± 1.1	–	219.4 ± 2.3	–	15.2 ± 0.8	17.8
60	39.7 ± 0.2	–	115.4 ± 2.4	–	561.6 ± 17.1	–	92.0 ± 1.6	67.5
80	39.9 ± 0.3	40.2	108.6 ± 2.3	103.6	593.0 ± 14.2	544.5	96.6 ± 1.6	93.2
100	39.8 ± 0.1	40.0	103.9 ± 2.1	98.3	621.4 ± 17.0	577.8	98.5 ± 4.9	97.6
120	39.6 ± 0.1	39.8	99.3 ± 3.6	94.4	656.1 ± 23.8	606.7	107.0 ± 3.9	101.5
140	39.6 ± 0.1	39.7	95.7 ± 1.6	91.4	677.9 ± 12.4	632.5	115.4 ± 11.6	105.2
160	39.6 ± 0.1	39.7	94.1 ± 1.5	89.0	693.9 ± 8.2	656.0	113.2 ± 6.4	108.7
180	39.6 ± 0.1	39.7	90.8 ± 1.1	87.0	717.4 ± 6.5	677.6	114.9 ± 2.0	112.0
200	39.7	39.7	91.9 ± 4.1	85.4	737.7 ± 34.6	697.7	120.8 ± 2.2	115.1

Table S230: Densities computed from MC and MD simulations (ρ^{MC} and ρ^{MD}), densities obtained from REFPROP[10] (ρ^{REFP}), isothermal compressibilities computed from MC simulations (β_T^{MC}), isothermal compressibilities obtained from REFPROP[10] (β_T^{REFP}), thermal expansion coefficients computed from MC simulations (α_P^{MC}), thermal expansion coefficients obtained from REFPROP[10] (α_P^{REFP}), Joule Thomson coefficients computed from MC simulations (μ_{JT}^{MC}), and Joule Thomson coefficients obtained from REFPROP[10] (μ_{JT}^{REFP}) of CO_2 rich ternary mixture with 2% impurity of N_2 and 2% impurity of H_2 at 293 K and pressures ranging from 20 bar to 200 bar.

P/ [bar]	ρ^{MC}/ [kg/m^3]	ρ^{MD}/ [kg/m^3]	ρ^{REFP}/ [kg/m^3]	β_T^{MC}/ [10^{-5}/bar]	β_T^{REFP}/ [10^{-5}/bar]	α_P^{MC}/ [10^{-4}/K]	α_P^{REFP}/ [10^{-4}/K]	μ_{JT}^{MC}/ [10^{-3} K/bar]	μ_{JT}^{REFP}/ [10^{-3} K/bar]
20	38.8	39.6 ± 0.1	39.4	5575.4 ± 22.5	5648.9	47.5 ± 0.2	49.3	988.8 ± 12.6	1091.7
40	88.9 ± 0.1	91.1 ± 0.4	92.2	3346.7 ± 41.3	3477.3	75.5 ± 0.9	82.6	1016.2 ± 22.4	1084.3
60	–	178.2 ± 2.2	185.2	–	4105.0	–	241.3	–	1015.2
80	717.2 ± 5.3	713.4 ± 4.6	716.4	574.2 ± 73.2	548.3	227.1 ± 18.5	206.8	166.5 ± 17.6	168.2
100	772.0 ± 4.1	773.9 ± 3.1	770.2	244.8 ± 33.6	255.5	121.7 ± 12.7	118.6	101.8 ± 15.9	105.2
120	804.2 ± 2.3	802.7 ± 0.3	802.8	158.8 ± 16.2	171.7	90.0 ± 7.8	89.9	72.6 ± 10.9	76.6
140	825.4 ± 1.9	823.6 ± 2.0	827.2	129.8 ± 5.6	130.8	79.9 ± 3.0	74.8	60.5 ± 4.3	58.9
160	845.2 ± 2.0	844.7 ± 1.0	846.9	103.6 ± 6.9	106.3	68.2 ± 3.8	65.1	47.5 ± 5.5	46.5
180	861.4 ± 1.3	860.3 ± 1.7	863.6	84.0 ± 2.1	89.8	59.1 ± 1.6	58.3	36.3 ± 2.4	37.1
200	875.3 ± 1.3	874.2 ± 0.8	878.1	74.8 ± 4.5	77.9	54.5 ± 3.1	53.1	30.3 ± 4.7	29.8

Table S231: Heat capacities at constant volume computed from MC simulations (c_V^{MC}), heat capacities at constant volume obtained from REFPROP[10] (c_V^{REFP}), heat capacities at constant pressure computed from MC simulations (c_P^{MC}), heat capacities at constant pressure obtained from REFPROP[10] (c_P^{REFP}), speed of sound computed from MC simulations (c^{MC}), speed of sound obtained from REFPROP[10] (c^{REFP}), viscosities computed from MD simulations (η^{MD}), and viscosities obtained from REFPROP[10] (η^{REFP}) of CO_2 rich ternary mixture with 2% impurity of N_2 and 2% impurity of H_2 at 293 K and pressures ranging from 20 bar to 200 bar.

P/ [bar]	c_V^{MC}/ [J/mol K]	c_V^{REFP}/ [J/mol K]	c_P^{MC}/ [J/mol K]	c_P^{REFP}/ [J/mol K]	c^{MC}/ [m/s]	c^{REFP}/ [m/s]	η^{MD}/ [μPa s]	η^{REFP}/ [μPa s]
20	30.6	30.7	43.7 ± 0.1	44.4	257.0 ± 0.6	255.1	2.1 ± 2.4	15.0
40	33.5	34.1	57.5 ± 0.3	60.9	240.2 ± 1.6	235.8	16.2 ± 2.7	15.7
60	–	42.2	–	138.4	–	207.7	20.1 ± 2.2	17.7
80	41.2 ± 0.4	43.2	203.0 ± 8.9	179.9	346.0 ± 23.4	325.6	60.0 ± 2.1	58.7
100	40.1 ± 0.1	41.1	140.0 ± 8.1	130.8	429.8 ± 32.1	402.1	73.5 ± 4.0	67.2
120	39.7 ± 0.4	40.3	120.3 ± 6.3	114.0	487.2 ± 28.1	452.8	76.4 ± 4.9	73.1
140	39.6 ± 0.2	39.9	115.0 ± 2.9	104.8	520.7 ± 13.0	492.5	78.4 ± 3.2	77.9
160	39.2 ± 0.1	39.7	106.6 ± 3.4	98.8	557.1 ± 20.5	526.0	90.5 ± 8.8	82.1
180	39.3 ± 0.2	39.5	100.2 ± 1.9	94.5	593.7 ± 9.4	555.3	87.6 ± 1.7	85.8
200	39.3 ± 0.2	39.4	96.5 ± 3.3	91.2	612.1 ± 21.4	581.5	91.0 ± 2.4	89.3

Table S232: Densities computed from MC and MD simulations (ρ^{MC} and ρ^{MD}), densities obtained from REFPROP[10] (ρ^{REFP}), isothermal compressibilities computed from MC simulations (β_T^{MC}), isothermal compressibilities obtained from REFPROP[10] (β_T^{REFP}), thermal expansion coefficients computed from MC simulations (α_P^{MC}), thermal expansion coefficients obtained from REFPROP[10] (α_P^{REFP}), Joule Thomson coefficients computed from MC simulations ($\mu_{\text{JT}}^{\text{MC}}$), and Joule Thomson coefficients obtained from REFPROP[10] ($\mu_{\text{JT}}^{\text{REFP}}$) of CO_2 rich ternary mixture with 2% impurity of N_2 and 2% impurity of H_2 at 313 K and pressures ranging from 20 bar to 200 bar.

P/ [bar]	ρ^{MC}/ [kg/m^3]	ρ^{MD}/ [kg/m^3]	ρ^{REFP}/ [kg/m^3]	β_T^{MC}/ [10^{-5}/bar]	β_T^{REFP}/ [10^{-5}/bar]	α_P^{MC}/ [10^{-4}/K]	α_P^{REFP}/ [10^{-4}/K]	$\mu_{\text{JT}}^{\text{MC}}$/ [$10^{-3}$ K/bar]	$\mu_{\text{JT}}^{\text{REFP}}$/ [$10^{-3}$ K/bar]
20	35.5	36.2 ± 0.1	35.9	5400.8 ± 35.8	5482.1	41.3 ± 0.2	42.6	825.3 ± 21.9	920.9
40	78.1	79.6 ± 0.3	80.1	3024.5 ± 23.4	3118.5	56.8 ± 0.5	60.6	836.4 ± 17.0	908.9
60	133.1 ± 0.2	136.4 ± 0.5	139.1	2408.4 ± 24.4	2540.0	86.7 ± 0.9	97.1	828.9 ± 14.0	875.7
80	215.0 ± 0.6	223.3 ± 2.3	233.0	2453.6 ± 37.5	2748.6	167.0 ± 3.6	203.7	764.9 ± 25.0	–
100	387.9 ± 3.5	401.0 ± 5.2	434.6	3458.6 ± 127.9	2946.2	518.4 ± 24.7	482.0	530.6 ± 40.0	480.7
120	588.1 ± 5.6	586.2 ± 3.7	603.9	943.3 ± 100.0	831.5	260.0 ± 28.3	228.8	251.0 ± 38.9	239.5
140	669.1 ± 1.9	663.4 ± 3.7	676.0	422.1 ± 33.7	393.6	155.2 ± 10.7	137.5	158.4 ± 15.7	153.6
160	712.5 ± 4.5	711.3 ± 3.0	719.7	261.7 ± 33.0	252.0	110.3 ± 10.3	102.0	115.0 ± 16.2	111.4
180	744.3 ± 1.7	742.4 ± 1.1	751.3	196.4 ± 5.7	184.8	92.1 ± 1.8	83.3	92.0 ± 2.8	85.8
200	769.8 ± 1.5	768.8 ± 0.6	776.3	144.4 ± 8.4	145.7	75.1 ± 3.6	71.4	69.9 ± 6.2	68.2

Table S233: Heat capacities at constant volume computed from MC simulations (c_V^{MC}), heat capacities at constant volume obtained from REFPROP[10] (c_V^{REFP}), heat capacities at constant pressure computed from MC simulations (c_P^{MC}), heat capacities at constant pressure obtained from REFPROP[10] (c_P^{REFP}), speed of sound computed from MC simulations (c^{MC}), speed of sound obtained from REFPROP[10] (c^{REFP}), viscosities computed from MD simulations (η^{MD}), and viscosities obtained from REFPROP[10] (η^{REFP}) of CO_2 rich ternary mixture with 2% impurity of N_2 and 2% impurity of H_2 at 313 K and pressures ranging from 20 bar to 200 bar.

P/ [bar]	c_V^{MC}/ [J/mol K]	c_V^{REFP}/ [J/mol K]	c_P^{MC}/ [J/mol K]	c_P^{REFP}/ [J/mol K]	c^{MC}/ [m/s]	c^{REFP}/ [m/s]	η^{MD}/ [μPa s]	η^{REFP}/ [μPa s]
20	30.9	30.9	42.8 ± 0.1	43.3	268.9 ± 0.9	266.6	10.9 ± 3.2	16.0
40	32.7	33.0	51.0 ± 0.2	52.8	256.9 ± 1.1	252.7	17.7 ± 1.5	16.6
60	35.2 ± 0.1	35.9	66.6 ± 0.4	71.7	243.1 ± 1.4	237.6	19.3 ± 2.6	17.7
80	38.6 ± 0.3	–	110.1 ± 2.1	–	232.4 ± 3.0	–	21.0 ± 0.9	20.3
100	42.9 ± 0.4	45.6	317.0 ± 17.4	288.9	234.8 ± 7.9	222.5	27.9 ± 1.4	30.6
120	41.2 ± 0.5	42.7	207.3 ± 19.1	182.5	301.1 ± 21.3	291.8	43.3 ± 4.2	45.3
140	39.9 ± 0.3	41.0	155.9 ± 7.4	136.3	371.9 ± 17.3	353.4	53.9 ± 3.8	53.9
160	39.4 ± 0.2	40.2	128.2 ± 6.6	117.2	417.4 ± 28.5	400.9	60.0 ± 2.3	59.8
180	39.1 ± 0.2	39.8	117.7 ± 1.1	106.8	453.6 ± 7.0	439.8	64.1 ± 5.7	64.6
200	39.0 ± 0.1	39.5	107.5 ± 3.1	100.0	498.1 ± 16.2	473.0	72.3 ± 1.7	68.8

S16.9 Data of thermodynamics and transport properties of CO_2 rich ternary mixture with 2 mole% impurity of H_2 and 2 mole% impurity of CH_4

Table S234: Densities computed from MC and MD simulations (ρ^{MC} and ρ^{MD}), densities obtained from REFPROP[10] (ρ^{REFP}), isothermal compressibilities computed from MC simulations (β_T^{MC}), isothermal compressibilities obtained from REFPROP[10] (β_T^{REFP}), thermal expansion coefficients computed from MC simulations (α_P^{MC}), thermal expansion coefficients obtained from REFPROP[10] (α_P^{REFP}), Joule Thomson coefficients computed from MC simulations (μ_{JT}^{MC}), and Joule Thomson coefficients obtained from REFPROP[10] (μ_{JT}^{REFP}) of CO_2 rich ternary mixture with 2% impurity of H_2 and 2% impurity of CH_4 at 253 K and pressures ranging from 20 bar to 200 bar.

P/ [bar]	ρ^{MC}/ [kg/m^3]	ρ^{MD}/ [kg/m^3]	ρ^{REFP}/ [kg/m^3]	β_T^{MC}/ [10^{-5}/bar]	β_T^{REFP}/ [10^{-5}/bar]	α_P^{MC}/ [10^{-4}/K]	α_P^{REFP}/ [10^{-4}/K]	μ_{JT}^{MC}/ [10^{-3} K/bar]	μ_{JT}^{REFP}/ [10^{-3} K/bar]
20	48.8	49.7 ± 0.2	50.2	6304.1 ± 45.3	6501.2	74.4 ± 0.8	81.1	1511.7 ± 34.1	1640.1
40	981.4 ± 1.0	979.4 ± 0.7	754.3	45.2 ± 1.4	−226.4	48.0 ± 1.7	−34.5	9.7 ± 1.9	–
60	990.1 ± 0.7	987.4 ± 1.0	982.4	41.0 ± 2.5	47.6	45.3 ± 3.2	47.1	6.6 ± 3.7	9.1
80	997.6 ± 0.9	996.6 ± 0.8	991.4	39.8 ± 1.6	43.3	44.8 ± 1.5	44.2	6.0 ± 1.8	5.7
100	1003.8 ± 0.7	1004.2 ± 1.3	999.6	36.5 ± 1.7	39.8	41.6 ± 1.7	41.8	2.5 ± 2.0	2.9
120	1012.5 ± 1.2	1010.3 ± 1.1	1007.3	33.7 ± 1.1	36.9	39.5 ± 1.2	39.8	0.0 ± 1.5	0.4
140	1019.9 ± 1.6	1018.1 ± 0.3	1014.5	31.0 ± 1.4	34.5	38.0 ± 1.6	38.1	-1.8 ± 1.9	−1.8
160	1026.0 ± 0.8	1024.1 ± 0.6	1021.3	28.3 ± 1.2	32.3	35.2 ± 1.6	36.5	-5.3 ± 2.0	−3.8
180	1030.8 ± 0.5	1030.3 ± 0.8	1027.8	27.7 ± 0.4	30.5	34.6 ± 0.2	35.2	-6.0 ± 0.3	−5.5
200	1037.4 ± 0.9	1035.6 ± 0.9	1033.9	26.8 ± 1.1	28.9	34.2 ± 1.3	34.0	-6.4 ± 1.6	−7.1

Table S235: Heat capacities at constant volume computed from MC simulations (c_V^{MC}), heat capacities at constant volume obtained from REFPROP[10] (c_V^{REFP}), heat capacities at constant pressure computed from MC simulations (c_P^{MC}), heat capacities at constant pressure obtained from REFPROP[10] (c_P^{REFP}), speed of sound computed from MC simulations (c^{MC}), speed of sound obtained from REFPROP[10] (c^{REFP}), viscosities computed from MD simulations (η^{MD}), and viscosities obtained from REFPROP[10] (η^{REFP}) of CO_2 rich ternary mixture with 2% impurity of H_2 and 2% impurity of CH_4 at 253 K and pressures ranging from 20 bar to 200 bar.

P/ [bar]	c_V^{MC}/ [J/mol K]	c_V^{REFP}/ [J/mol K]	c_P^{MC}/ [J/mol K]	c_P^{REFP}/ [J/mol K]	c^{MC}/ [m/s]	c^{REFP}/ [m/s]	η^{MD}/ [μPa s]	η^{REFP}/ [μPa s]
20	31.6	32.8	50.9 ± 0.3	54.5	229.1 ± 1.0	225.9	12.9 ± 1.4	12.9
40	40.2 ± 0.1	–	96.2 ± 2.3	–	733.7 ± 14.3	–	131.8 ± 1.2	66.8
60	40.2 ± 0.2	40.1	95.0 ± 4.2	91.3	763.1 ± 29.2	698.0	134.4 ± 3.0	125.8
80	40.3 ± 0.2	40.1	94.7 ± 1.7	89.2	769.3 ± 16.9	720.2	137.0 ± 4.7	129.6
100	40.2 ± 0.1	40.1	91.3 ± 2.1	87.5	787.5 ± 20.2	740.7	151.1 ± 5.1	133.2
120	40.2 ± 0.2	40.1	89.9 ± 1.7	86.0	809.9 ± 15.1	759.8	149.6 ± 9.7	136.6
140	40.5 ± 0.2	40.1	89.4 ± 2.3	84.8	835.1 ± 21.6	777.7	144.2 ± 5.4	140.0
160	40.4 ± 0.1	40.1	86.3 ± 2.5	83.7	857.0 ± 22.3	794.7	152.2 ± 7.4	143.3
180	40.3 ± 0.1	40.2	85.6 ± 0.2	82.7	862.6 ± 6.1	810.7	157.1 ± 5.1	146.4
200	40.4 ± 0.2	40.2	85.9 ± 1.7	81.8	874.3 ± 19.8	826.0	167.1 ± 12.0	149.5

Table S236: Densities computed from MC and MD simulations (ρ^{MC} and ρ^{MD}), densities obtained from REFPROP[10] (ρ^{REFP}), isothermal compressibilities computed from MC simulations (β_T^{MC}), isothermal compressibilities obtained from REFPROP[10] (β_T^{REFP}), thermal expansion coefficients computed from MC simulations (α_P^{MC}), thermal expansion coefficients obtained from REFPROP[10] (α_P^{REFP}), Joule Thomson coefficients computed from MC simulations ($\mu_{\mathrm{JT}}^{\mathrm{MC}}$), and Joule Thomson coefficients obtained from REFPROP[10] ($\mu_{\mathrm{JT}}^{\mathrm{REFP}}$) of CO_2 rich ternary mixture with 2% impurity of H_2 and 2% impurity of CH_4 at 273 K and pressures ranging from 20 bar to 200 bar.

P/ [bar]	ρ^{MC}/ [kg/m^3]	ρ^{MD}/ [kg/m^3]	ρ^{REFP}/ [kg/m^3]	β_T^{MC}/ [10^{-5}/bar]	β_T^{REFP}/ [10^{-5}/bar]	α_P^{MC}/ [10^{-4}/K]	α_P^{REFP}/ [10^{-4}/K]	$\mu_{\mathrm{JT}}^{\mathrm{MC}}$/ [$10^{-3}$ K/bar]	$\mu_{\mathrm{JT}}^{\mathrm{REFP}}$/ [$10^{-3}$ K/bar]
20	42.8	43.7 ± 0.1	43.7	5787.7 ± 29.1	5934.6	56.8 ± 0.2	60.1	1193.1 ± 14.0	1321.9
40	107.5 ± 0.2	110.7 ± 0.6	245.8	4364.4 ± 117.5	−3747.0	134.2 ± 4.8	−534.3	1293.4 ± 72.7	–
60	882.4 ± 1.1	879.0 ± 1.4	857.3	94.1 ± 4.3	141.9	71.2 ± 3.7	89.6	40.9 ± 4.6	–
80	900.2 ± 1.5	894.2 ± 1.5	891.0	76.3 ± 3.0	91.8	61.3 ± 1.9	66.2	30.7 ± 2.4	37.7
100	912.4 ± 1.0	910.7 ± 1.3	906.1	71.9 ± 3.6	77.2	60.3 ± 2.7	58.8	28.9 ± 3.4	29.2
120	925.0 ± 1.5	922.7 ± 1.1	919.2	59.8 ± 2.7	66.9	52.5 ± 2.1	53.4	20.4 ± 2.7	22.6
140	935.4 ± 1.7	933.2 ± 0.7	930.8	52.9 ± 2.7	59.2	47.9 ± 2.2	49.2	14.9 ± 2.9	17.3
160	944.8 ± 1.3	943.6 ± 0.2	941.3	47.6 ± 1.3	53.3	45.3 ± 0.9	45.9	11.5 ± 1.1	13.0
180	953.8 ± 1.7	951.6 ± 0.6	950.9	44.0 ± 2.5	48.5	42.7 ± 1.9	43.2	8.2 ± 2.6	9.3
200	961.5 ± 0.9	960.3 ± 0.5	959.8	41.5 ± 1.0	44.6	41.3 ± 1.0	40.9	6.3 ± 1.4	6.1

Table S237: Heat capacities at constant volume computed from MC simulations (c_V^{MC}), heat capacities at constant volume obtained from REFPROP[10] (c_V^{REFP}), heat capacities at constant pressure computed from MC simulations (c_P^{MC}), heat capacities at constant pressure obtained from REFPROP[10] (c_P^{REFP}), speed of sound computed from MC simulations (c^{MC}), speed of sound obtained from REFPROP[10] (c^{REFP}), viscosities computed from MD simulations (η^{MD}), and viscosities obtained from REFPROP[10] (η^{REFP}) of CO_2 rich ternary mixture with 2% impurity of H_2 and 2% impurity of CH_4 at 273 K and pressures ranging from 20 bar to 200 bar.

P/ [bar]	c_V^{MC}/ [J/mol K]	c_V^{REFP}/ [J/mol K]	c_P^{MC}/ [J/mol K]	c_P^{REFP}/ [J/mol K]	c^{MC}/ [m/s]	c^{REFP}/ [m/s]	η^{MD}/ [μPa s]	η^{REFP}/ [μPa s]
20	30.7	31.0	45.8 ± 0.1	47.2	245.2 ± 0.7	242.3	10.0 ± 4.4	13.9
40	36.9 ± 0.2	–	81.6 ± 2.2	–	217.2 ± 4.2	–	15.4 ± 2.2	18.5
60	39.8 ± 0.2	–	111.6 ± 4.1	–	581.0 ± 17.1	–	95.9 ± 0.9	85.4
80	39.7 ± 0.3	40.0	103.9 ± 1.7	102.2	617.4 ± 13.3	558.7	98.2 ± 1.0	93.9
100	39.6 ± 0.3	39.9	104.7 ± 2.6	97.3	634.7 ± 17.6	590.9	105.2 ± 6.6	98.2
120	39.7 ± 0.1	39.8	97.7 ± 2.1	93.7	666.4 ± 16.8	619.1	116.1 ± 9.9	102.0
140	39.7 ± 0.2	39.7	93.8 ± 2.3	90.9	691.2 ± 19.7	644.4	110.3 ± 4.2	105.6
160	39.7 ± 0.2	39.7	92.8 ± 0.8	88.6	720.3 ± 10.4	667.4	126.3 ± 8.6	109.0
180	39.8 ± 0.2	39.7	90.1 ± 1.8	86.7	734.3 ± 22.2	688.6	124.6 ± 6.3	112.3
200	39.9 ± 0.1	39.7	89.7 ± 1.3	85.1	750.1 ± 10.5	708.4	128.1 ± 5.0	115.4

Table S238: Densities computed from MC and MD simulations (ρ^{MC} and ρ^{MD}), densities obtained from REFPROP[10] (ρ^{REFP}), isothermal compressibilities computed from MC simulations (β_T^{MC}), isothermal compressibilities obtained from REFPROP[10] (β_T^{REFP}), thermal expansion coefficients computed from MC simulations (α_P^{MC}), thermal expansion coefficients obtained from REFPROP[10] (α_P^{REFP}), Joule Thomson coefficients computed from MC simulations ($\mu_{\mathrm{JT}}^{\mathrm{MC}}$), and Joule Thomson coefficients obtained from REFPROP[10] ($\mu_{\mathrm{JT}}^{\mathrm{REFP}}$) of CO_2 rich ternary mixture with 2% impurity of H_2 and 2% impurity of CH_4 at 293 K and pressures ranging from 20 bar to 200 bar.

P/ [bar]	ρ^{MC}/ [kg/m^3]	ρ^{MD}/ [kg/m^3]	ρ^{REFP}/ [kg/m^3]	β_T^{MC}/ [10^{-5}/bar]	β_T^{REFP}/ [10^{-5}/bar]	α_P^{MC}/ [10^{-4}/K]	α_P^{REFP}/ [10^{-4}/K]	$\mu_{\mathrm{JT}}^{\mathrm{MC}}$/ [$10^{-3}$ K/bar]	$\mu_{\mathrm{JT}}^{\mathrm{REFP}}$/ [$10^{-3}$ K/bar]
20	38.6	39.4 ± 0.1	39.2	5589.6 ± 30.3	5656.1	47.8 ± 0.3	49.4	1009.3 ± 24.2	1094.4
40	88.8	91.1 ± 0.5	92.0	3355.2 ± 20.0	3497.9	76.0 ± 0.5	83.5	1018.3 ± 12.6	1091.3
60	170.4 ± 0.7	185.8 ± 3.7	187.0	3480.3 ± 69.8	4296.6	184.8 ± 4.3	258.7	989.1 ± 34.9	1019.8
80	739.5 ± 6.5	732.7 ± 3.2	727.3	371.3 ± 38.7	444.6	164.6 ± 11.9	178.4	132.2 ± 13.2	149.7
100	778.7 ± 3.3	780.8 ± 2.3	774.1	211.7 ± 18.1	230.1	109.5 ± 7.3	110.8	91.2 ± 9.5	97.4
120	807.2 ± 2.9	806.9 ± 0.7	804.1	149.8 ± 6.6	159.6	87.2 ± 3.1	85.9	69.0 ± 4.4	71.8
140	831.4 ± 1.9	827.6 ± 1.3	826.9	109.2 ± 5.5	123.5	70.3 ± 2.5	72.2	50.3 ± 3.6	55.5
160	845.9 ± 1.8	844.9 ± 1.5	845.6	99.3 ± 3.8	101.3	66.7 ± 2.2	63.2	45.4 ± 3.2	43.9
180	862.8 ± 2.6	860.4 ± 1.6	861.5	79.4 ± 4.5	86.1	56.7 ± 3.1	56.8	33.3 ± 4.6	35.1
200	876.1 ± 1.6	873.4 ± 1.0	875.4	68.6 ± 2.4	75.1	51.4 ± 1.7	52.0	26.1 ± 2.7	28.1

Table S239: Heat capacities at constant volume computed from MC simulations (c_V^{MC}), heat capacities at constant volume obtained from REFPROP[10] (c_V^{REFP}), heat capacities at constant pressure computed from MC simulations (c_P^{MC}), heat capacities at constant pressure obtained from REFPROP[10] (c_P^{REFP}), speed of sound computed from MC simulations (c^{MC}), speed of sound obtained from REFPROP[10] (c^{REFP}), viscosities computed from MD simulations (η^{MD}), and viscosities obtained from REFPROP[10] (η^{REFP}) of CO_2 rich ternary mixture with 2% impurity of H_2 and 2% impurity of CH_4 at 293 K and pressures ranging from 20 bar to 200 bar.

P/ [bar]	c_V^{MC}/ [J/mol K]	c_V^{REFP}/ [J/mol K]	c_P^{MC}/ [J/mol K]	c_P^{REFP}/ [J/mol K]	c^{MC}/ [m/s]	c^{REFP}/ [m/s]	η^{MD}/ [μPa s]	η^{REFP}/ [μPa s]
20	30.7	30.8	43.9 ± 0.1	44.6	257.5 ± 0.8	255.5	4.9 ± 5.5	14.9
40	33.6 ± 0.1	34.3	57.8 ± 0.2	61.4	240.2 ± 0.9	235.7	15.9 ± 1.7	15.6
60	39.4 ± 0.3	43.0	111.6 ± 2.3	147.0	218.6 ± 3.3	206.2	19.3 ± 1.4	17.7
80	40.4 ± 0.3	42.5	166.7 ± 6.6	165.4	387.4 ± 21.8	346.8	64.8 ± 3.2	60.7
100	39.9 ± 0.4	40.9	132.6 ± 5.3	126.9	449.0 ± 21.4	417.3	69.6 ± 1.8	68.4
120	39.3 ± 0.1	40.2	119.1 ± 2.9	112.0	500.8 ± 12.6	465.9	84.2 ± 7.4	74.0
140	39.5 ± 0.2	39.8	107.9 ± 1.9	103.6	548.4 ± 14.8	504.5	82.9 ± 4.8	78.6
160	39.4 ± 0.2	39.6	106.0 ± 2.0	97.9	566.5 ± 12.1	537.2	87.7 ± 2.9	82.6
180	39.4 ± 0.2	39.5	98.1 ± 3.2	93.9	603.0 ± 19.8	565.9	93.9 ± 7.7	86.3
200	39.2 ± 0.1	39.4	94.5 ± 2.0	90.8	633.2 ± 13.2	591.8	95.7 ± 7.4	89.7

Table S240: Densities computed from MC and MD simulations (ρ^{MC} and ρ^{MD}), densities obtained from REFPROP[10] (ρ^{REFP}), isothermal compressibilities computed from MC simulations (β_T^{MC}), isothermal compressibilities obtained from REFPROP[10] (β_T^{REFP}), thermal expansion coefficients computed from MC simulations (α_P^{MC}), thermal expansion coefficients obtained from REFPROP[10] (α_P^{REFP}), Joule Thomson coefficients computed from MC simulations (μ_{JT}^{MC}), and Joule Thomson coefficients obtained from REFPROP[10] (μ_{JT}^{REFP}) of CO_2 rich ternary mixture with 2% impurity of H_2 and 2% impurity of CH_4 at 313 K and pressures ranging from 20 bar to 200 bar.

P/ [bar]	ρ^{MC}/ [kg/m^3]	ρ^{MD}/ [kg/m^3]	ρ^{REFP}/ [kg/m^3]	β_T^{MC}/ [10^{-5}/bar]	β_T^{REFP}/ [10^{-5}/bar]	α_P^{MC}/ [10^{-4}/K]	α_P^{REFP}/ [10^{-4}/K]	μ_{JT}^{MC}/ [10^{-3} K/bar]	μ_{JT}^{REFP}/ [10^{-3} K/bar]
20	35.3	35.9 ± 0.1	35.8	5435.4 ± 13.5	5487.4	41.6 ± 0.1	42.7	848.5 ± 7.6	922.5
40	77.9	79.5 ± 0.2	79.9	3051.9 ± 22.4	3128.9	57.5 ± 0.5	61.0	851.6 ± 18.4	913.8
60	132.9 ± 0.2	136.2 ± 0.7	139.1	2425.8 ± 28.4	2563.1	88.3 ± 1.2	98.6	837.2 ± 19.2	881.6
80	217.4 ± 1.6	228.2 ± 4.5	235.1	2621.8 ± 57.5	2838.2	183.3 ± 5.4	214.4	784.5 ± 33.7	–
100	415.8 ± 5.2	421.3 ± 6.5	454.0	3403.5 ± 162.3	2970.1	559.5 ± 29.1	518.7	492.8 ± 36.0	458.3
120	613.5 ± 1.9	602.0 ± 2.8	615.5	746.6 ± 102.4	743.0	225.8 ± 27.2	215.0	219.4 ± 36.0	223.8
140	681.5 ± 2.3	675.7 ± 1.9	681.7	347.9 ± 27.1	361.8	135.2 ± 9.2	130.9	140.8 ± 14.1	145.0
160	720.3 ± 4.7	718.6 ± 1.2	722.4	238.8 ± 12.4	236.3	105.8 ± 3.8	98.4	107.6 ± 6.2	105.9
180	750.1 ± 1.8	747.8 ± 1.2	752.3	172.4 ± 5.0	175.3	85.4 ± 1.9	80.8	82.5 ± 3.1	81.9
200	772.6 ± 1.8	769.7 ± 2.1	776.2	135.5 ± 2.1	139.3	72.3 ± 1.2	69.7	65.6 ± 2.2	65.2

Table S241: Heat capacities at constant volume computed from MC simulations (c_V^{MC}), heat capacities at constant volume obtained from REFPROP[10] (c_V^{REFP}), heat capacities at constant pressure computed from MC simulations (c_P^{MC}), heat capacities at constant pressure obtained from REFPROP[10] (c_P^{REFP}), speed of sound computed from MC simulations (c^{MC}), speed of sound obtained from REFPROP[10] (c^{REFP}), viscosities computed from MD simulations (η^{MD}), and viscosities obtained from REFPROP[10] (η^{REFP}) of CO_2 rich ternary mixture with 2% impurity of H_2 and 2% impurity of CH_4 at 313 K and pressures ranging from 20 bar to 200 bar.

P/ [bar]	c_V^{MC}/ [J/mol K]	c_V^{REFP}/ [J/mol K]	c_P^{MC}/ [J/mol K]	c_P^{REFP}/ [J/mol K]	c^{MC}/ [m/s]	c^{REFP}/ [m/s]	η^{MD}/ [μPa s]	η^{REFP}/ [μPa s]
20	31.0	31.0	43.1	43.4	268.9 ± 0.3	267.0	6.3 ± 5.0	15.9
40	32.8	33.2	51.4 ± 0.2	53.0	256.4 ± 1.1	252.8	17.7 ± 2.2	16.4
60	35.3 ± 0.1	36.1	67.6 ± 0.6	72.5	243.6 ± 1.8	237.3	18.7 ± 2.1	17.6
80	39.1 ± 0.3	–	118.4 ± 2.6	–	230.4 ± 3.8	–	19.3 ± 1.2	20.3
100	43.3 ± 1.0	45.9	343.9 ± 16.0	312.0	237.1 ± 8.5	224.6	29.3 ± 2.6	32.1
120	40.8 ± 0.2	42.4	192.1 ± 16.3	177.2	320.6 ± 25.9	302.2	47.0 ± 5.9	46.8
140	39.9 ± 0.2	40.9	143.5 ± 6.4	133.6	389.4 ± 17.6	364.0	53.8 ± 1.3	54.9
160	39.6 ± 0.3	40.1	127.1 ± 2.9	115.8	432.1 ± 12.5	411.0	64.8 ± 6.1	60.6
180	39.4 ± 0.1	39.7	115.1 ± 1.5	105.8	475.4 ± 7.6	449.4	65.2 ± 2.0	65.3
200	39.0 ± 0.1	39.5	106.1 ± 1.4	99.4	509.8 ± 5.3	482.3	70.6 ± 5.0	69.3

S16.10 Data of thermodynamics and transport properties of CO_2 rich ternary mixture with 2 mole% impurity of Ar and 2 mole% impurity of CH_4

Table S242: Densities computed from MC and MD simulations (ρ^{MC} and ρ^{MD}), densities obtained from REFPROP[10] (ρ^{REFP}), isothermal compressibilities computed from MC simulations (β_T^{MC}), isothermal compressibilities obtained from REFPROP[10] (β_T^{REFP}), thermal expansion coefficients computed from MC simulations (α_P^{MC}), thermal expansion coefficients obtained from REFPROP[10] (α_P^{REFP}), Joule Thomson coefficients computed from MC simulations (μ_{JT}^{MC}), and Joule Thomson coefficients obtained from REFPROP[10] (μ_{JT}^{REFP}) of CO_2 rich ternary mixture with 2% impurity of Ar and 2% impurity of CH_4 at 253 K and pressures ranging from 20 bar to 200 bar.

P/ [bar]	ρ^{MC}/ [kg/m^3]	ρ^{MD}/ [kg/m^3]	ρ^{REFP}/ [kg/m^3]	β_T^{MC}/ [10^{-5}/bar]	β_T^{REFP}/ [10^{-5}/bar]	α_P^{MC}/ [10^{-4}/K]	α_P^{REFP}/ [10^{-4}/K]	μ_{JT}^{MC}/ [10^{-3} K/bar]	μ_{JT}^{REFP}/ [10^{-3} K/bar]
20	49.8	50.8 ± 0.2	51.2	6317.9 ± 40.6	6533.2	74.9 ± 0.6	82.2	1526.7 ± 25.1	1661.8
40	1002.5 ± 0.9	1000.3 ± 0.7	1002.5	43.5 ± 1.5	49.8	47.6 ± 1.8	49.3	9.1 ± 2.0	11.4
60	1011.6 ± 1.8	1008.8 ± 0.8	1012.1	39.4 ± 1.5	45.0	44.4 ± 1.4	46.0	5.7 ± 1.6	7.7
80	1019.2 ± 1.0	1016.6 ± 1.5	1020.8	36.1 ± 0.9	41.2	41.8 ± 1.1	43.4	2.7 ± 1.3	4.6
100	1026.4 ± 1.0	1024.1 ± 0.8	1028.9	35.0 ± 1.5	38.0	41.2 ± 2.0	41.1	2.0 ± 2.4	2.0
120	1033.2 ± 0.3	1030.7 ± 0.5	1036.5	31.4 ± 1.2	35.3	38.2 ± 1.6	39.2	-1.6 ± 1.9	-0.4
140	1038.9 ± 1.3	1037.5 ± 0.4	1043.6	28.6 ± 0.8	33.1	35.2 ± 0.8	37.6	-5.3 ± 1.0	-2.4
160	1045.2 ± 0.5	1043.8 ± 0.7	1050.3	28.0 ± 0.6	31.1	35.4 ± 1.0	36.1	-5.0 ± 1.2	-4.3
180	1050.4 ± 1.1	1049.3 ± 1.1	1056.7	26.7 ± 0.6	29.4	34.2 ± 1.0	34.8	-6.4 ± 1.2	-5.9
200	1056.2 ± 1.1	1054.7 ± 0.8	1062.7	25.7 ± 0.8	27.9	33.6 ± 0.9	33.6	-7.1 ± 1.1	-7.4

Table S243: Heat capacities at constant volume computed from MC simulations (c_V^{MC}), heat capacities at constant volume obtained from REFPROP[10] (c_V^{REFP}), heat capacities at constant pressure computed from MC simulations (c_P^{MC}), heat capacities at constant pressure obtained from REFPROP[10] (c_P^{REFP}), speed of sound computed from MC simulations (c^{MC}), speed of sound obtained from REFPROP[10] (c^{REFP}), viscosities computed from MD simulations (η^{MD}), and viscosities obtained from REFPROP[10] (η^{REFP}) of CO_2 rich ternary mixture with 2% impurity of Ar and 2% impurity of CH_4 at 253 K and pressures ranging from 20 bar to 200 bar.

P/ [bar]	c_V^{MC}/ [J/mol K]	c_V^{REFP}/ [J/mol K]	c_P^{MC}/ [J/mol K]	c_P^{REFP}/ [J/mol K]	c^{MC}/ [m/s]	c^{REFP}/ [m/s]	η^{MD}/ [μPa s]	η^{REFP}/ [μPa s]
20	31.5	32.8	51.0 ± 0.1	55.0	227.0 ± 0.8	223.7	12.5 ± 3.0	13.0
40	40.0 ± 0.2	40.1	97.1 ± 2.8	93.5	745.5 ± 16.9	683.4	143.7 ± 6.2	129.1
60	40.2 ± 0.2	40.1	94.3 ± 1.4	91.2	767.0 ± 15.5	706.7	142.7 ± 3.6	133.1
80	40.0 ± 0.2	40.1	92.3 ± 1.8	89.2	791.4 ± 13.0	728.0	147.9 ± 5.4	137.0
100	40.3 ± 0.2	40.1	91.8 ± 3.0	87.6	797.0 ± 22.0	747.7	145.5 ± 7.4	140.6
120	40.3 ± 0.2	40.1	89.5 ± 2.3	86.2	827.2 ± 19.5	766.2	155.3 ± 11.3	144.2
140	40.2 ± 0.2	40.1	85.8 ± 0.8	85.0	847.5 ± 13.2	783.5	158.6 ± 2.8	147.6
160	40.2 ± 0.1	40.2	87.1 ± 1.9	83.9	859.5 ± 13.5	799.9	169.3 ± 18.1	151.0
180	40.4 ± 0.3	40.2	85.9 ± 1.7	83.0	871.0 ± 13.4	815.5	167.8 ± 11.4	154.2
200	40.4 ± 0.2	40.2	85.8 ± 1.0	82.2	884.4 ± 14.6	830.4	161.1 ± 6.1	157.4

Table S244: Densities computed from MC and MD simulations (ρ^{MC} and ρ^{MD}), densities obtained from REFPROP[10] (ρ^{REFP}), isothermal compressibilities computed from MC simulations (β_T^{MC}), isothermal compressibilities obtained from REFPROP[10] (β_T^{REFP}), thermal expansion coefficients computed from MC simulations (α_P^{MC}), thermal expansion coefficients obtained from REFPROP[10] (α_P^{REFP}), Joule Thomson coefficients computed from MC simulations ($\mu_{\mathrm{JT}}^{\mathrm{MC}}$), and Joule Thomson coefficients obtained from REFPROP[10] ($\mu_{\mathrm{JT}}^{\mathrm{REFP}}$) of CO_2 rich ternary mixture with 2% impurity of Ar and 2% impurity of CH_4 at 273 K and pressures ranging from 20 bar to 200 bar.

P/ [bar]	ρ^{MC}/ [kg/m^3]	ρ^{MD}/ [kg/m^3]	ρ^{REFP}/ [kg/m^3]	β_T^{MC}/ [10^{-5}/bar]	β_T^{REFP}/ [10^{-5}/bar]	α_P^{MC}/ [10^{-4}/K]	α_P^{REFP}/ [10^{-4}/K]	$\mu_{\mathrm{JT}}^{\mathrm{MC}}$/ [$10^{-3}$ K/bar]	$\mu_{\mathrm{JT}}^{\mathrm{REFP}}$/ [$10^{-3}$ K/bar]
20	43.7	44.6 ± 0.1	44.5	5820.2 ± 48.3	5947.6	57.3 ± 0.5	60.4	1220.1 ± 31.5	1338.3
40	–	116.1 ± 1.7	284.0	–	-1852.4	–	-403.4	–	–
60	907.6 ± 1.7	905.4 ± 2.8	902.7	85.2 ± 6.1	105.0	67.3 ± 3.4	73.7	36.5 ± 4.2	45.0
80	921.7 ± 1.4	920.4 ± 0.9	920.0	71.0 ± 1.0	85.5	59.1 ± 0.9	63.9	28.0 ± 1.2	34.6
100	935.4 ± 1.5	931.8 ± 1.0	934.5	61.6 ± 2.1	72.6	53.9 ± 1.8	57.2	22.0 ± 2.3	26.9
120	943.8 ± 1.6	945.0 ± 1.0	947.3	56.6 ± 1.2	63.4	51.1 ± 1.1	52.2	18.7 ± 1.4	20.9
140	956.0 ± 1.0	954.6 ± 0.6	958.7	50.7 ± 2.4	56.5	47.3 ± 2.2	48.4	14.0 ± 2.9	16.0
160	965.0 ± 1.7	964.3 ± 0.7	969.0	46.5 ± 2.3	51.0	45.2 ± 2.5	45.3	11.2 ± 3.3	11.9
180	973.5 ± 0.9	972.3 ± 1.1	978.5	42.2 ± 1.7	46.6	41.9 ± 1.7	42.7	7.1 ± 2.3	8.4
200	982.0 ± 0.6	980.1 ± 0.5	987.3	39.7 ± 1.1	42.9	40.5 ± 1.3	40.5	5.2 ± 1.7	5.4

Table S245: Heat capacities at constant volume computed from MC simulations (c_V^{MC}), heat capacities at constant volume obtained from REFPROP[10] (c_V^{REFP}), heat capacities at constant pressure computed from MC simulations (c_P^{MC}), heat capacities at constant pressure obtained from REFPROP[10] (c_P^{REFP}), speed of sound computed from MC simulations (c^{MC}), speed of sound obtained from REFPROP[10] (c^{REFP}), viscosities computed from MD simulations (η^{MD}), and viscosities obtained from REFPROP[10] (η^{REFP}) of CO_2 rich ternary mixture with 2% impurity of Ar and 2% impurity of CH_4 at 273 K and pressures ranging from 20 bar to 200 bar.

P/ [bar]	c_V^{MC}/ [J/mol K]	c_V^{REFP}/ [J/mol K]	c_P^{MC}/ [J/mol K]	c_P^{REFP}/ [J/mol K]	c^{MC}/ [m/s]	c^{REFP}/ [m/s]	η^{MD}/ [μPa s]	η^{REFP}/ [μPa s]
20	30.6	30.9	45.9 ± 0.2	47.2	242.7 ± 1.1	240.1	2.3 ± 2.5	14.1
40	–	–	–	–	–	–	15.7 ± 1.7	20.0
60	39.3 ± 0.2	40.3	109.5 ± 3.0	108.0	600.0 ± 22.9	532.0	98.1 ± 11.7	94.7
80	39.4 ± 0.2	40.0	103.0 ± 1.1	101.5	632.4 ± 6.0	568.2	106.0 ± 2.5	99.4
100	39.4 ± 0.1	39.8	99.3 ± 2.1	97.0	661.2 ± 13.4	599.0	117.7 ± 11.5	103.7
120	39.4 ± 0.2	39.7	97.4 ± 1.4	93.6	680.1 ± 8.8	626.1	128.0 ± 13.6	107.6
140	39.5 ± 0.1	39.7	94.5 ± 2.6	90.9	702.5 ± 19.3	650.5	119.7 ± 6.0	111.3
160	39.4 ± 0.1	39.7	93.2 ± 3.1	88.7	726.4 ± 21.9	672.8	130.9 ± 22.0	114.8
180	39.5 ± 0.1	39.7	90.1 ± 2.3	86.9	745.1 ± 17.7	693.4	127.7 ± 4.9	118.1
200	39.5 ± 0.1	39.7	89.6 ± 1.8	85.4	763.4 ± 13.3	712.6	124.4 ± 3.4	121.3

Table S246: Densities computed from MC and MD simulations (ρ^{MC} and ρ^{MD}), densities obtained from REFPROP[10] (ρ^{REFP}), isothermal compressibilities computed from MC simulations (β_T^{MC}), isothermal compressibilities obtained from REFPROP[10] (β_T^{REFP}), thermal expansion coefficients computed from MC simulations (α_P^{MC}), thermal expansion coefficients obtained from REFPROP[10] (α_P^{REFP}), Joule Thomson coefficients computed from MC simulations ($\mu_{\mathrm{JT}}^{\mathrm{MC}}$), and Joule Thomson coefficients obtained from REFPROP[10] ($\mu_{\mathrm{JT}}^{\mathrm{REFP}}$) of CO_2 rich ternary mixture with 2% impurity of Ar and 2% impurity of CH_4 at 293 K and pressures ranging from 20 bar to 200 bar.

P/ [bar]	ρ^{MC}/ [kg/m^3]	ρ^{MD}/ [kg/m^3]	ρ^{REFP}/ [kg/m^3]	β_T^{MC}/ [10^{-5}/bar]	β_T^{REFP}/ [10^{-5}/bar]	α_P^{MC}/ [10^{-4}/K]	α_P^{REFP}/ [10^{-4}/K]	$\mu_{\mathrm{JT}}^{\mathrm{MC}}$/ [$10^{-3}$ K/bar]	$\mu_{\mathrm{JT}}^{\mathrm{REFP}}$/ [$10^{-3}$ K/bar]
20	39.4	40.1 ± 0.1	39.9	5615.4 ± 53.9	5663.6	48.2 ± 0.5	49.6	1034.8 ± 33.9	1107.5
40	91.1 ± 0.1	93.0 ± 0.3	94.0	3388.7 ± 29.3	3520.4	77.7 ± 0.8	84.7	1041.5 ± 20.8	1104.2
60	178.8 ± 0.4	193.5 ± 2.8	194.2	3929.4 ± 153.4	4601.0	219.7 ± 11.4	287.2	1032.8 ± 80.4	1026.4
80	776.9 ± 4.4	771.6 ± 4.7	760.7	283.8 ± 27.5	370.2	141.4 ± 10.4	158.6	112.1 ± 11.9	133.6
100	811.9 ± 3.9	808.1 ± 1.6	803.2	190.1 ± 24.5	208.0	105.7 ± 10.6	104.8	84.2 ± 13.4	90.0
120	835.6 ± 0.8	832.9 ± 1.6	831.8	133.4 ± 9.9	148.3	82.6 ± 5.6	82.9	62.5 ± 7.7	67.2
140	854.7 ± 1.2	853.1 ± 0.3	853.8	97.8 ± 3.3	116.3	65.3 ± 1.7	70.4	44.4 ± 2.4	52.3
160	869.6 ± 1.6	868.1 ± 2.0	872.1	86.0 ± 3.2	96.3	60.7 ± 2.0	62.0	38.1 ± 3.0	41.5
180	884.4 ± 0.3	881.7 ± 0.7	887.7	76.2 ± 4.5	82.4	56.8 ± 3.3	56.0	32.6 ± 4.8	33.3
200	898.1 ± 1.4	895.3 ± 0.7	901.5	64.1 ± 2.2	72.1	50.1 ± 1.8	51.3	23.9 ± 2.8	26.7

Table S247: Heat capacities at constant volume computed from MC simulations (c_V^{MC}), heat capacities at constant volume obtained from REFPROP[10] (c_V^{REFP}), heat capacities at constant pressure computed from MC simulations (c_P^{MC}), heat capacities at constant pressure obtained from REFPROP[10] (c_P^{REFP}), speed of sound computed from MC simulations (c^{MC}), speed of sound obtained from REFPROP[10] (c^{REFP}), viscosities computed from MD simulations (η^{MD}), and viscosities obtained from REFPROP[10] (η^{REFP}) of CO_2 rich ternary mixture with 2% impurity of Ar and 2% impurity of CH_4 at 293 K and pressures ranging from 20 bar to 200 bar.

P/ [bar]	c_V^{MC}/ [J/mol K]	c_V^{REFP}/ [J/mol K]	c_P^{MC}/ [J/mol K]	c_P^{REFP}/ [J/mol K]	c^{MC}/ [m/s]	c^{REFP}/ [m/s]	η^{MD}/ [μPa s]	η^{REFP}/ [μPa s]
20	30.6	30.7	43.9 ± 0.1	44.5	254.9 ± 1.3	253.3	5.4 ± 4.2	15.1
40	33.5	34.4	58.4 ± 0.3	61.9	237.7 ± 1.2	233.3	17.8 ± 1.7	15.7
60	39.9 ± 0.6	44.0	127.7 ± 6.1	161.3	213.4 ± 6.8	202.5	17.6 ± 1.0	18.0
80	39.8 ± 0.3	42.2	156.6 ± 6.8	155.7	422.4 ± 22.5	362.1	68.8 ± 1.5	65.2
100	39.4 ± 0.3	40.8	133.1 ± 7.7	124.3	468.1 ± 33.1	427.2	76.8 ± 1.3	72.6
120	39.1 ± 0.2	40.1	117.7 ± 5.2	111.0	519.7 ± 22.4	473.5	82.3 ± 6.2	78.1
140	39.1 ± 0.3	39.8	104.3 ± 1.3	103.1	564.7 ± 10.4	510.7	88.3 ± 6.1	82.8
160	39.0 ± 0.2	39.6	102.0 ± 2.2	97.8	591.4 ± 12.9	542.4	90.7 ± 3.5	86.9
180	39.0 ± 0.1	39.5	100.0 ± 3.6	93.9	616.5 ± 21.2	570.4	98.8 ± 7.4	90.6
200	39.1 ± 0.2	39.4	94.5 ± 2.1	90.9	648.0 ± 13.2	595.6	102.9 ± 10.0	94.1

Table S248: Densities computed from MC and MD simulations (ρ^{MC} and ρ^{MD}), densities obtained from REFPROP[10] (ρ^{REFP}), isothermal compressibilities computed from MC simulations (β_T^{MC}), isothermal compressibilities obtained from REFPROP[10] (β_T^{REFP}), thermal expansion coefficients computed from MC simulations (α_P^{MC}), thermal expansion coefficients obtained from REFPROP[10] (α_P^{REFP}), Joule Thomson coefficients computed from MC simulations ($\mu_{\mathrm{JT}}^{\mathrm{MC}}$), and Joule Thomson coefficients obtained from REFPROP[10] ($\mu_{\mathrm{JT}}^{\mathrm{REFP}}$) of CO_2 rich ternary mixture with 2% impurity of Ar and 2% impurity of CH_4 at 313 K and pressures ranging from 20 bar to 200 bar.

P/ [bar]	ρ^{MC}/ [kg/m^3]	ρ^{MD}/ [kg/m^3]	ρ^{REFP}/ [kg/m^3]	β_T^{MC}/ [10^{-5}/bar]	β_T^{REFP}/ [10^{-5}/bar]	α_P^{MC}/ [10^{-4}/K]	α_P^{REFP}/ [10^{-4}/K]	$\mu_{\mathrm{JT}}^{\mathrm{MC}}$/ [$10^{-3}$ K/bar]	$\mu_{\mathrm{JT}}^{\mathrm{REFP}}$/ [$10^{-3}$ K/bar]
20	36.0	36.7 ± 0.1	36.4	5464.6 ± 15.5	5492.3	41.9 ± 0.1	42.8	874.2 ± 9.9	933.2
40	79.6	81.3 ± 0.4	81.5	3081.6 ± 34.0	3138.9	58.4 ± 0.6	61.4	875.1 ± 20.2	924.4
60	136.8 ± 0.3	141.1 ± 0.3	142.3	2473.2 ± 26.1	2589.1	92.0 ± 1.2	100.6	860.3 ± 19.4	891.5
80	226.7 ± 1.0	242.1 ± 6.4	243.4	2831.7 ± 104.3	2959.7	203.9 ± 9.0	229.6	806.5 ± 50.4	–
100	481.0 ± 16.2	483.8 ± 7.8	489.9	3393.7 ± 461.7	2862.2	649.8 ± 78.0	541.9	429.4 ± 73.5	427.3
120	648.5 ± 5.4	645.8 ± 2.2	645.0	567.2 ± 39.0	667.5	189.4 ± 10.2	203.7	189.0 ± 13.9	208.7
140	710.6 ± 3.5	707.0 ± 1.9	708.0	334.2 ± 40.0	336.0	134.9 ± 12.0	126.6	135.4 ± 17.1	137.2
160	746.1 ± 4.0	744.6 ± 1.8	747.6	207.2 ± 10.2	223.2	96.8 ± 3.9	96.2	96.6 ± 6.3	101.1
180	774.5 ± 1.4	771.1 ± 1.3	776.9	153.5 ± 5.7	167.2	79.2 ± 3.3	79.5	74.6 ± 5.7	78.6
200	796.2 ± 3.1	795.3 ± 0.8	800.5	122.8 ± 6.7	133.8	67.9 ± 2.9	68.8	59.3 ± 4.9	62.8

Table S249: Heat capacities at constant volume computed from MC simulations (c_V^{MC}), heat capacities at constant volume obtained from REFPROP[10] (c_V^{REFP}), heat capacities at constant pressure computed from MC simulations (c_P^{MC}), heat capacities at constant pressure obtained from REFPROP[10] (c_P^{REFP}), speed of sound computed from MC simulations (c^{MC}), speed of sound obtained from REFPROP[10] (c^{REFP}), viscosities computed from MD simulations (η^{MD}), and viscosities obtained from REFPROP[10] (η^{REFP}) of CO_2 rich ternary mixture with 2% impurity of Ar and 2% impurity of CH_4 at 313 K and pressures ranging from 20 bar to 200 bar.

P/ [bar]	c_V^{MC}/ [J/mol K]	c_V^{REFP}/ [J/mol K]	c_P^{MC}/ [J/mol K]	c_P^{REFP}/ [J/mol K]	c^{MC}/ [m/s]	c^{REFP}/ [m/s]	η^{MD}/ [μPa s]	η^{REFP}/ [μPa s]
20	30.9	30.9	43.0	43.3	266.0 ± 0.4	264.7	7.8 ± 4.0	16.0
40	32.7	33.1	51.5 ± 0.2	53.1	253.5 ± 1.5	250.5	16.7 ± 1.9	16.6
60	35.2 ± 0.1	36.2	69.3 ± 0.6	73.5	241.0 ± 1.7	234.8	16.5 ± 1.5	17.8
80	39.2 ± 0.1	–	127.7 ± 4.3	–	225.3 ± 5.6	–	21.6 ± 1.6	20.8
100	42.6 ± 0.4	46.4	406.6 ± 45.1	330.7	241.9 ± 21.6	225.5	37.3 ± 4.9	34.8
120	40.2 ± 0.4	42.4	174.5 ± 6.1	173.2	343.5 ± 13.4	308.1	52.1 ± 5.2	49.9
140	39.4 ± 0.1	40.8	145.2 ± 7.1	132.3	393.8 ± 25.5	369.1	59.2 ± 4.0	57.9
160	39.0 ± 0.1	40.1	122.3 ± 3.1	115.4	450.6 ± 12.6	415.1	67.8 ± 9.1	63.7
180	38.9 ± 0.1	39.7	111.0 ± 3.3	105.8	490.0 ± 11.7	452.8	77.1 ± 15.9	68.5
200	38.8 ± 0.2	39.5	103.2 ± 2.3	99.5	522.0 ± 15.4	485.1	72.1 ± 2.5	72.6

S16.11 Data of thermodynamics and transport properties of CO_2 rich ternary mixture with 2 mole% impurity of Ar and 2 mole% impurity of H_2

Table S250: Densities computed from MC and MD simulations (ρ^{MC} and ρ^{MD}), densities obtained from REFPROP[10] (ρ^{REFP}), isothermal compressibilities computed from MC simulations (β_T^{MC}), isothermal compressibilities obtained from REFPROP[10] (β_T^{REFP}), thermal expansion coefficients computed from MC simulations (α_P^{MC}), thermal expansion coefficients obtained from REFPROP[10] (α_P^{REFP}), Joule Thomson coefficients computed from MC simulations ($\mu_{\mathrm{JT}}^{\mathrm{MC}}$), and Joule Thomson coefficients obtained from REFPROP[10] ($\mu_{\mathrm{JT}}^{\mathrm{REFP}}$) of CO_2 rich ternary mixture with 2% impurity of Ar and 2% impurity of H_2 at 253 K and pressures ranging from 20 bar to 200 bar.

P/ [bar]	ρ^{MC}/ [kg/m^3]	ρ^{MD}/ [kg/m^3]	ρ^{REFP}/ [kg/m^3]	β_T^{MC}/ [10^{-5}/bar]	β_T^{REFP}/ [10^{-5}/bar]	α_P^{MC}/ [10^{-4}/K]	α_P^{REFP}/ [10^{-4}/K]	$\mu_{\mathrm{JT}}^{\mathrm{MC}}$/ [$10^{-3}$ K/bar]	$\mu_{\mathrm{JT}}^{\mathrm{REFP}}$/ [$10^{-3}$ K/bar]
20	49.2	50.4 ± 0.1	50.5	6249.5 ± 54.2	6457.5	73.3 ± 1.0	79.7	1487.6 ± 45.6	1623.2
40	994.0 ± 1.2	991.5 ± 1.3	703.3	48.2 ± 3.7	-74.3	50.8 ± 4.0	36.8	12.5 ± 4.4	–
60	1004.3 ± 0.9	1001.2 ± 0.7	968.2	40.5 ± 1.4	66.1	43.8 ± 1.2	59.3	5.1 ± 1.4	–
80	1011.8 ± 1.9	1009.4 ± 0.6	1006.2	39.0 ± 1.5	43.4	43.6 ± 1.2	44.5	4.7 ± 1.3	6.0
100	1019.3 ± 1.5	1017.6 ± 0.8	1014.6	35.8 ± 1.1	39.9	41.1 ± 1.1	42.1	1.9 ± 1.3	3.2
120	1026.7 ± 0.5	1025.6 ± 0.8	1022.5	34.9 ± 1.5	37.0	41.0 ± 1.4	40.1	1.8 ± 1.6	0.7
140	1032.4 ± 1.0	1032.2 ± 0.5	1029.8	31.7 ± 2.4	34.5	38.0 ± 2.9	38.3	-1.8 ± 3.4	-1.6
160	1041.0 ± 1.5	1039.2 ± 0.9	1036.7	30.4 ± 2.0	32.4	38.0 ± 2.3	36.7	-1.8 ± 2.7	-3.5
180	1047.1 ± 0.7	1044.8 ± 0.3	1043.2	28.6 ± 1.0	30.5	36.1 ± 1.2	35.4	-4.0 ± 1.4	-5.3
200	1052.2 ± 0.8	1050.8 ± 0.4	1049.5	26.9 ± 0.8	28.9	34.2 ± 0.9	34.1	-6.5 ± 1.2	-6.8

Table S251: Heat capacities at constant volume computed from MC simulations (c_V^{MC}), heat capacities at constant volume obtained from REFPROP[10] (c_V^{REFP}), heat capacities at constant pressure computed from MC simulations (c_P^{MC}), heat capacities at constant pressure obtained from REFPROP[10] (c_P^{REFP}), speed of sound computed from MC simulations (c^{MC}), speed of sound obtained from REFPROP[10] (c^{REFP}), viscosities computed from MD simulations (η^{MD}), and viscosities obtained from REFPROP[10] (η^{REFP}) of CO_2 rich ternary mixture with 2% impurity of Ar and 2% impurity of H_2 at 253 K and pressures ranging from 20 bar to 200 bar.

P/ [bar]	c_V^{MC}/ [J/mol K]	c_V^{REFP}/ [J/mol K]	c_P^{MC}/ [J/mol K]	c_P^{REFP}/ [J/mol K]	c^{MC}/ [m/s]	c^{REFP}/ [m/s]	η^{MD}/ [μPa s]	η^{REFP}/ [μPa s]
20	31.2	32.2	50.3 ± 0.4	53.4	228.8 ± 1.3	225.5	12.0 ± 3.2	13.1
40	39.8 ± 0.3	–	99.1 ± 4.9	–	720.9 ± 33.3	–	133.5 ± 3.5	60.6
60	39.8 ± 0.2	–	91.6 ± 0.8	–	752.2 ± 13.3	–	133.8 ± 2.4	115.9
80	40.0 ± 0.3	39.9	92.7 ± 1.3	89.3	766.4 ± 15.8	716.3	139.0 ± 3.6	130.8
100	40.0 ± 0.2	39.9	90.7 ± 1.6	87.6	788.5 ± 13.8	736.6	152.8 ± 4.8	134.4
120	39.9 ± 0.2	39.9	91.4 ± 1.7	86.1	799.4 ± 18.3	755.5	150.0 ± 9.5	137.9
140	40.0 ± 0.2	39.9	88.2 ± 3.8	84.9	820.9 ± 35.9	773.3	151.6 ± 10.3	141.3
160	40.3 ± 0.1	39.9	90.0 ± 3.0	83.7	840.8 ± 31.2	790.1	153.9 ± 6.9	144.6
180	40.1 ± 0.1	40.0	87.8 ± 1.7	82.8	855.2 ± 17.1	806.0	155.4 ± 9.4	147.8
200	40.3 ± 0.2	40.0	85.3 ± 1.4	81.9	865.6 ± 15.3	821.2	165.0 ± 13.1	150.9

Table S252: Densities computed from MC and MD simulations (ρ^{MC} and ρ^{MD}), densities obtained from REFPROP[10] (ρ^{REFP}), isothermal compressibilities computed from MC simulations (β_T^{MC}), isothermal compressibilities obtained from REFPROP[10] (β_T^{REFP}), thermal expansion coefficients computed from MC simulations (α_P^{MC}), thermal expansion coefficients obtained from REFPROP[10] (α_P^{REFP}), Joule Thomson coefficients computed from MC simulations (μ_{JT}^{MC}), and Joule Thomson coefficients obtained from REFPROP[10] (μ_{JT}^{REFP}) of CO_2 rich ternary mixture with 2% impurity of Ar and 2% impurity of H_2 at 273 K and pressures ranging from 20 bar to 200 bar.

P/ [bar]	ρ^{MC}/ [kg/m^3]	ρ^{MD}/ [kg/m^3]	ρ^{REFP}/ [kg/m^3]	β_T^{MC}/ [10^{-5}/bar]	β_T^{REFP}/ [10^{-5}/bar]	α_P^{MC}/ [10^{-4}/K]	α_P^{REFP}/ [10^{-4}/K]	μ_{JT}^{MC}/ [10^{-3} K/bar]	μ_{JT}^{REFP}/ [10^{-3} K/bar]
20	43.2	44.1 ± 0.1	44.1	5818.8 ± 44.1	5915.9	56.9 ± 0.4	59.5	1209.3 ± 24.3	1311.5
40	107.7 ± 0.2	112.1 ± 0.5	232.8	4196.4 ± 42.2	−6289.6	126.9 ± 1.4	−737.1	1254.4 ± 20.8	–
60	891.5 ± 1.6	889.1 ± 2.7	800.5	110.0 ± 8.9	505.0	80.0 ± 5.7	236.7	48.7 ± 6.8	–
80	909.4 ± 2.7	906.9 ± 1.1	903.5	83.4 ± 4.4	92.6	65.6 ± 3.0	66.9	35.1 ± 3.7	38.4
100	924.7 ± 2.9	922.0 ± 1.0	919.0	70.3 ± 4.1	77.7	58.5 ± 3.0	59.3	27.4 ± 3.9	29.8
120	936.3 ± 1.5	934.5 ± 0.4	932.3	62.0 ± 4.4	67.3	54.3 ± 3.5	53.9	22.3 ± 4.5	23.2
140	947.9 ± 0.9	946.1 ± 1.3	944.2	54.3 ± 2.8	59.6	49.4 ± 2.3	49.7	16.6 ± 3.0	17.8
160	957.7 ± 1.0	956.2 ± 0.9	954.9	49.7 ± 2.2	53.6	46.5 ± 1.7	46.3	13.1 ± 2.3	13.4
180	967.6 ± 1.3	964.3 ± 0.9	964.7	43.7 ± 0.4	48.7	42.5 ± 0.9	43.5	8.0 ± 1.2	9.7
200	974.4 ± 0.7	973.6 ± 0.4	973.8	42.6 ± 2.4	44.8	41.9 ± 2.3	41.2	7.1 ± 3.2	6.5

Table S253: Heat capacities at constant volume computed from MC simulations (c_V^{MC}), heat capacities at constant volume obtained from REFPROP[10] (c_V^{REFP}), heat capacities at constant pressure computed from MC simulations (c_P^{MC}), heat capacities at constant pressure obtained from REFPROP[10] (c_P^{REFP}), speed of sound computed from MC simulations (c^{MC}), speed of sound obtained from REFPROP[10] (c^{REFP}), viscosities computed from MD simulations (η^{MD}), and viscosities obtained from REFPROP[10] (η^{REFP}) of CO_2 rich ternary mixture with 2% impurity of Ar and 2% impurity of H_2 at 273 K and pressures ranging from 20 bar to 200 bar.

P/ [bar]	c_V^{MC}/ [J/mol K]	c_V^{REFP}/ [J/mol K]	c_P^{MC}/ [J/mol K]	c_P^{REFP}/ [J/mol K]	c^{MC}/ [m/s]	c^{REFP}/ [m/s]	η^{MD}/ [μPa s]	η^{REFP}/ [μPa s]
20	30.4	30.6	45.5 ± 0.1	46.6	243.9 ± 1.0	241.6	6.7 ± 3.0	14.1
40	36.6 ± 0.3	–	78.6 ± 0.5	–	217.9 ± 1.5	–	15.6 ± 2.1	18.1
60	39.5 ± 0.1	–	117.5 ± 5.2	–	550.9 ± 25.4	–	95.8 ± 3.0	71.4
80	39.3 ± 0.2	39.7	106.7 ± 2.9	102.6	598.0 ± 17.7	555.4	99.3 ± 2.1	94.6
100	39.5 ± 0.1	39.6	101.6 ± 2.9	97.6	629.0 ± 20.4	587.5	117.9 ± 32.2	98.9
120	39.4 ± 0.1	39.5	99.6 ± 3.8	93.9	659.6 ± 26.8	615.5	115.7 ± 14.3	102.8
140	39.4 ± 0.1	39.4	95.3 ± 2.3	91.0	685.8 ± 19.8	640.6	117.6 ± 7.9	106.5
160	39.6 ± 0.1	39.4	92.8 ± 1.7	88.7	701.7 ± 17.1	663.5	118.7 ± 10.3	109.9
180	39.6 ± 0.1	39.4	89.8 ± 1.8	86.8	732.1 ± 8.1	684.6	120.1 ± 4.2	113.2
200	39.3 ± 0.1	39.4	89.2 ± 2.8	85.2	739.5 ± 24.2	704.3	131.9 ± 19.4	116.3

Table S254: Densities computed from MC and MD simulations (ρ^{MC} and ρ^{MD}), densities obtained from REFPROP[10] (ρ^{REFP}), isothermal compressibilities computed from MC simulations (β_T^{MC}), isothermal compressibilities obtained from REFPROP[10] (β_T^{REFP}), thermal expansion coefficients computed from MC simulations (α_P^{MC}), thermal expansion coefficients obtained from REFPROP[10] (α_P^{REFP}), Joule Thomson coefficients computed from MC simulations ($\mu_{\mathrm{JT}}^{\mathrm{MC}}$), and Joule Thomson coefficients obtained from REFPROP[10] ($\mu_{\mathrm{JT}}^{\mathrm{REFP}}$) of CO_2 rich ternary mixture with 2% impurity of Ar and 2% impurity of H_2 at 293 K and pressures ranging from 20 bar to 200 bar.

P/ [bar]	ρ^{MC}/ [kg/m^3]	ρ^{MD}/ [kg/m^3]	ρ^{REFP}/ [kg/m^3]	β_T^{MC}/ [10^{-5}/bar]	β_T^{REFP}/ [10^{-5}/bar]	α_P^{MC}/ [10^{-4}/K]	α_P^{REFP}/ [10^{-4}/K]	$\mu_{\mathrm{JT}}^{\mathrm{MC}}$/ [$10^{-3}$ K/bar]	$\mu_{\mathrm{JT}}^{\mathrm{REFP}}$/ [$10^{-3}$ K/bar]
20	39.0	39.8	39.6	5592.9 ± 40.1	5645.4	47.7 ± 0.3	49.2	1008.3 ± 24.2	1087.8
40	89.5	91.8 ± 0.3	92.6	3319.5 ± 36.1	3471.2	74.9 ± 0.8	82.0	1007.2 ± 19.3	1083.8
60	170.9 ± 0.9	180.6 ± 2.6	185.4	3544.1 ± 64.7	4072.0	185.2 ± 5.0	237.0	1008.9 ± 39.9	1018.4
80	745.7 ± 10.0	729.5 ± 2.7	734.1	357.6 ± 75.7	468.8	158.7 ± 25.1	186.6	130.4 ± 29.0	154.2
100	789.4 ± 4.5	784.0 ± 3.8	783.2	207.1 ± 14.4	236.2	108.8 ± 5.1	113.4	90.2 ± 6.6	99.6
120	816.6 ± 2.8	813.4 ± 1.8	814.2	159.3 ± 16.0	162.5	91.0 ± 7.0	87.4	72.8 ± 9.4	73.2
140	839.2 ± 2.3	836.2 ± 1.6	837.7	118.5 ± 8.0	125.2	75.0 ± 4.4	73.2	55.3 ± 6.3	56.6
160	856.7 ± 1.7	856.0 ± 0.9	856.9	98.3 ± 2.2	102.5	65.7 ± 1.9	64.0	44.7 ± 2.8	44.8
180	871.5 ± 1.0	871.6 ± 0.4	873.2	83.5 ± 6.7	87.0	59.0 ± 4.0	57.5	36.1 ± 5.9	35.9
200	885.8 ± 1.0	884.3 ± 0.9	887.5	70.1 ± 1.6	75.8	51.9 ± 1.7	52.5	26.9 ± 2.7	28.8

Table S255: Heat capacities at constant volume computed from MC simulations (c_V^{MC}), heat capacities at constant volume obtained from REFPROP[10] (c_V^{REFP}), heat capacities at constant pressure computed from MC simulations (c_P^{MC}), heat capacities at constant pressure obtained from REFPROP[10] (c_P^{REFP}), speed of sound computed from MC simulations (c^{MC}), speed of sound obtained from REFPROP[10] (c^{REFP}), viscosities computed from MD simulations (η^{MD}), and viscosities obtained from REFPROP[10] (η^{REFP}) of CO_2 rich ternary mixture with 2% impurity of Ar and 2% impurity of H_2 at 293 K and pressures ranging from 20 bar to 200 bar.

P/ [bar]	c_V^{MC}/ [J/mol K]	c_V^{REFP}/ [J/mol K]	c_P^{MC}/ [J/mol K]	c_P^{REFP}/ [J/mol K]	c^{MC}/ [m/s]	c^{REFP}/ [m/s]	η^{MD}/ [μPa s]	η^{REFP}/ [μPa s]
20	30.4	30.4	43.6 ± 0.1	44.1	256.2 ± 1.0	254.6	4.7 ± 3.1	15.1
40	33.3 ± 0.1	33.8	57.1 ± 0.3	60.2	240.1 ± 1.4	235.4	15.9 ± 1.5	15.8
60	39.2 ± 0.4	41.7	110.7 ± 2.3	135.7	215.8 ± 3.2	207.5	17.4 ± 0.5	17.8
80	40.2 ± 0.4	42.3	161.7 ± 15.1	170.0	388.6 ± 45.0	341.9	61.3 ± 2.2	60.7
100	39.8 ± 0.1	40.6	132.4 ± 3.1	128.3	450.8 ± 16.6	413.5	71.2 ± 8.2	68.7
120	39.4 ± 0.3	39.9	120.8 ± 4.9	112.7	485.4 ± 26.4	462.2	77.6 ± 3.4	74.4
140	39.1 ± 0.1	39.5	111.2 ± 4.0	104.0	535.0 ± 20.4	500.9	88.9 ± 9.0	79.1
160	39.1 ± 0.2	39.3	104.3 ± 2.1	98.2	562.8 ± 8.5	533.5	91.7 ± 15.2	83.2
180	39.0 ± 0.1	39.2	99.8 ± 3.5	94.1	592.7 ± 26.1	562.1	93.1 ± 5.8	86.9
200	39.1 ± 0.2	39.1	94.0 ± 2.4	90.9	622.3 ± 10.7	587.9	94.5 ± 2.3	90.3

Table S256: Densities computed from MC and MD simulations (ρ^{MC} and ρ^{MD}), densities obtained from REFPROP[10] (ρ^{REFP}), isothermal compressibilities computed from MC simulations (β_T^{MC}), isothermal compressibilities obtained from REFPROP[10] (β_T^{REFP}), thermal expansion coefficients computed from MC simulations (α_P^{MC}), thermal expansion coefficients obtained from REFPROP[10] (α_P^{REFP}), Joule Thomson coefficients computed from MC simulations ($\mu_{\mathrm{JT}}^{\mathrm{MC}}$), and Joule Thomson coefficients obtained from REFPROP[10] ($\mu_{\mathrm{JT}}^{\mathrm{REFP}}$) of CO_2 rich ternary mixture with 2% impurity of Ar and 2% impurity of H_2 at 313 K and pressures ranging from 20 bar to 200 bar.

P/ [bar]	ρ^{MC}/ [kg/m^3]	ρ^{MD}/ [kg/m^3]	ρ^{REFP}/ [kg/m^3]	β_T^{MC}/ [10^{-5}/bar]	β_T^{REFP}/ [10^{-5}/bar]	α_P^{MC}/ [10^{-4}/K]	α_P^{REFP}/ [10^{-4}/K]	$\mu_{\mathrm{JT}}^{\mathrm{MC}}$/ [$10^{-3}$ K/bar]	$\mu_{\mathrm{JT}}^{\mathrm{REFP}}$/ [$10^{-3}$ K/bar]
20	35.7	36.4 ± 0.1	36.1	5425.8 ± 23.8	5480.3	41.4 ± 0.2	42.5	837.9 ± 15.7	918.2
40	78.6 ± 0.1	80.1 ± 0.3	80.5	3059.9 ± 42.8	3116.6	57.5 ± 0.9	60.3	858.7 ± 32.4	908.7
60	133.9 ± 0.2	137.3 ± 0.4	139.7	2392.0 ± 47.1	2537.3	86.1 ± 1.9	96.5	824.0 ± 30.1	877.4
80	216.4 ± 0.7	226.2 ± 1.0	233.5	2438.4 ± 113.6	2752.1	165.0 ± 7.6	202.3	766.4 ± 48.7	781.5
100	402.2 ± 8.8	413.2 ± 5.8	444.7	3775.3 ± 298.8	3114.7	587.1 ± 61.1	520.8	524.5 ± 77.5	479.4
120	604.0 ± 5.1	597.5 ± 2.1	617.8	926.2 ± 83.9	790.9	262.0 ± 20.2	225.3	245.2 ± 25.6	231.7
140	680.8 ± 3.7	676.3 ± 1.6	687.8	381.2 ± 38.4	375.2	142.4 ± 11.6	134.7	149.4 ± 17.5	148.9
160	726.4 ± 3.1	723.7 ± 1.7	730.2	243.0 ± 18.4	242.3	106.3 ± 6.6	100.4	109.3 ± 10.6	108.4
180	756.5 ± 0.9	754.6 ± 3.6	761.1	184.8 ± 14.6	178.7	88.5 ± 6.1	82.2	87.4 ± 10.1	83.7
200	779.7 ± 0.9	779.3 ± 1.2	785.7	145.1 ± 11.9	141.6	76.2 ± 5.6	70.7	70.4 ± 9.4	66.7

Table S257: Heat capacities at constant volume computed from MC simulations (c_V^{MC}), heat capacities at constant volume obtained from REFPROP[10] (c_V^{REFP}), heat capacities at constant pressure computed from MC simulations (c_P^{MC}), heat capacities at constant pressure obtained from REFPROP[10] (c_P^{REFP}), speed of sound computed from MC simulations (c^{MC}), speed of sound obtained from REFPROP[10] (c^{REFP}), viscosities computed from MD simulations (η^{MD}), and viscosities obtained from REFPROP[10] (η^{REFP}) of CO_2 rich ternary mixture with 2% impurity of Ar and 2% impurity of H_2 at 313 K and pressures ranging from 20 bar to 200 bar.

P/ [bar]	c_V^{MC}/ [J/mol K]	c_V^{REFP}/ [J/mol K]	c_P^{MC}/ [J/mol K]	c_P^{REFP}/ [J/mol K]	c^{MC}/ [m/s]	c^{REFP}/ [m/s]	η^{MD}/ [μPa s]	η^{REFP}/ [μPa s]
20	30.7	30.7	42.7	43.0	267.8 ± 0.6	266.1	7.9 ± 7.4	16.1
40	32.5	32.8	51.0 ± 0.3	52.3	255.4 ± 2.0	252.3	16.4 ± 1.1	16.6
60	34.9 ± 0.1	35.6	66.2 ± 0.8	71.0	243.2 ± 2.9	237.2	18.5 ± 1.1	17.7
80	38.5 ± 0.4	40.0	108.2 ± 3.0	125.9	230.7 ± 6.4	221.3	23.8 ± 5.3	20.4
100	42.6 ± 0.5	45.2	355.2 ± 34.1	309.2	234.3 ± 14.9	222.3	29.0 ± 2.2	31.3
120	40.8 ± 0.6	42.1	209.5 ± 11.6	182.1	303.1 ± 16.3	297.7	55.6 ± 26.8	46.5
140	39.9 ± 0.4	40.5	146.5 ± 7.5	135.4	376.2 ± 21.4	359.9	55.9 ± 5.9	54.9
160	39.2 ± 0.3	39.8	126.3 ± 4.7	116.6	427.1 ± 18.2	407.1	63.1 ± 2.6	60.8
180	39.1 ± 0.1	39.4	115.4 ± 4.8	106.3	459.7 ± 20.4	445.5	68.3 ± 9.1	65.6
200	39.0 ± 0.2	39.1	108.7 ± 4.7	99.7	496.7 ± 23.1	478.4	67.8 ± 6.0	69.7

S16.12 Data of thermodynamics and transport properties of CO_2 rich ternary mixture with 2 mole% impurity of N_2 and 2 mole% impurity of CH_4

Table S258: Densities computed from MC and MD simulations (ρ^{MC} and ρ^{MD}), densities obtained from REFPROP[10] (ρ^{REFP}), isothermal compressibilities computed from MC simulations (β_T^{MC}), isothermal compressibilities obtained from REFPROP[10] (β_T^{REFP}), thermal expansion coefficients computed from MC simulations (α_P^{MC}), thermal expansion coefficients obtained from REFPROP[10] (α_P^{REFP}), Joule Thomson coefficients computed from MC simulations (μ_{JT}^{MC}), and Joule Thomson coefficients obtained from REFPROP[10] (μ_{JT}^{REFP}) of CO_2 rich ternary mixture with 2% impurity of N_2 and 2% impurity of CH_4 at 253 K and pressures ranging from 20 bar to 200 bar.

P/ [bar]	ρ^{MC}/ [kg/m^3]	ρ^{MD}/ [kg/m^3]	ρ^{REFP}/ [kg/m^3]	β_T^{MC}/ [10^{-5}/bar]	β_T^{REFP}/ [10^{-5}/bar]	α_P^{MC}/ [10^{-4}/K]	α_P^{REFP}/ [10^{-4}/K]	μ_{JT}^{MC}/ [10^{-3} K/bar]	μ_{JT}^{REFP}/ [10^{-3} K/bar]
20	49.5	50.6 ± 0.2	51.0	6305.3 ± 104.4	6551.3	74.6 ± 1.3	82.9	1514.1 ± 58.1	1668.9
40	992.0 ± 0.7	988.8 ± 0.8	991.7	44.8 ± 1.2	51.8	48.8 ± 1.1	50.2	10.3 ± 1.2	12.5
60	1000.2 ± 1.4	997.6 ± 1.5	1001.5	40.4 ± 2.1	46.6	45.0 ± 2.3	46.8	6.3 ± 2.6	8.6
80	1008.6 ± 1.3	1005.2 ± 0.6	1010.5	36.4 ± 1.7	42.5	42.1 ± 2.3	44.0	3.0 ± 2.7	5.4
100	1015.2 ± 1.2	1012.7 ± 0.7	1018.7	34.5 ± 1.7	39.1	40.4 ± 1.9	41.6	1.1 ± 2.2	2.6
120	1021.7 ± 0.8	1019.8 ± 0.3	1026.4	31.3 ± 1.0	36.3	37.6 ± 1.3	39.6	-2.3 ± 1.6	0.1
140	1026.9 ± 0.8	1025.7 ± 0.6	1033.7	30.6 ± 0.7	33.9	37.4 ± 0.9	37.9	-2.5 ± 1.1	-2.0
160	1033.5 ± 1.2	1032.5 ± 0.8	1040.5	28.9 ± 0.9	31.8	36.0 ± 1.5	36.4	-4.2 ± 1.8	-3.9
180	1039.4 ± 1.2	1037.5 ± 0.6	1046.9	27.8 ± 0.9	30.0	35.3 ± 1.5	35.1	-5.1 ± 1.8	-5.6
200	1045.0 ± 0.8	1043.7 ± 0.4	1053.1	25.7 ± 1.3	28.4	33.2 ± 1.8	33.9	-7.7 ± 2.2	-7.1

Table S259: Heat capacities at constant volume computed from MC simulations (c_V^{MC}), heat capacities at constant volume obtained from REFPROP[10] (c_V^{REFP}), heat capacities at constant pressure computed from MC simulations (c_P^{MC}), heat capacities at constant pressure obtained from REFPROP[10] (c_P^{REFP}), speed of sound computed from MC simulations (c^{MC}), speed of sound obtained from REFPROP[10] (c^{REFP}), viscosities computed from MD simulations (η^{MD}), and viscosities obtained from REFPROP[10] (η^{REFP}) of CO_2 rich ternary mixture with 2% impurity of N_2 and 2% impurity of CH_4 at 253 K and pressures ranging from 20 bar to 200 bar.

P/ [bar]	c_V^{MC}/ [J/mol K]	c_V^{REFP}/ [J/mol K]	c_P^{MC}/ [J/mol K]	c_P^{REFP}/ [J/mol K]	c^{MC}/ [m/s]	c^{REFP}/ [m/s]	η^{MD}/ [μPa s]	η^{REFP}/ [μPa s]
20	31.6	33.2	51.1 ± 0.4	55.6	227.4 ± 2.1	224.0	11.7 ± 3.1	13.0
40	40.1 ± 0.1	40.5	98.9 ± 1.5	94.0	744.5 ± 11.5	672.3	136.2 ± 13.7	127.4
60	40.1 ± 0.1	40.4	95.1 ± 3.0	91.5	765.8 ± 23.5	696.4	147.6 ± 21.7	131.4
80	40.5 ± 0.2	40.4	93.0 ± 3.4	89.5	791.2 ± 24.0	718.5	148.3 ± 8.7	135.3
100	40.4 ± 0.2	40.4	91.2 ± 2.3	87.8	802.6 ± 22.9	738.8	153.1 ± 5.2	139.0
120	40.3 ± 0.1	40.4	88.7 ± 1.9	86.4	829.0 ± 15.9	757.8	145.3 ± 4.6	142.6
140	40.5 ± 0.2	40.4	89.0 ± 1.8	85.1	836.4 ± 12.5	775.6	151.5 ± 7.8	146.0
160	40.5 ± 0.3	40.4	87.8 ± 2.5	84.0	851.6 ± 18.1	792.4	156.1 ± 5.4	149.4
180	40.5 ± 0.2	40.4	87.5 ± 2.7	83.1	865.3 ± 19.7	808.3	172.6 ± 33.8	152.7
200	40.3 ± 0.2	40.5	85.2 ± 2.4	82.2	886.1 ± 26.0	823.5	175.1 ± 27.7	155.9

Table S260: Densities computed from MC and MD simulations (ρ^{MC} and ρ^{MD}), densities obtained from REFPROP[10] (ρ^{REFP}), isothermal compressibilities computed from MC simulations (β_T^{MC}), isothermal compressibilities obtained from REFPROP[10] (β_T^{REFP}), thermal expansion coefficients computed from MC simulations (α_P^{MC}), thermal expansion coefficients obtained from REFPROP[10] (α_P^{REFP}), Joule Thomson coefficients computed from MC simulations (μ_{JT}^{MC}), and Joule Thomson coefficients obtained from REFPROP[10] (μ_{JT}^{REFP}) of CO_2 rich ternary mixture with 2% impurity of N_2 and 2% impurity of CH_4 at 273 K and pressures ranging from 20 bar to 200 bar.

P/ [bar]	ρ^{MC}/ [kg/m^3]	ρ^{MD}/ [kg/m^3]	ρ^{REFP}/ [kg/m^3]	β_T^{MC}/ [10^{-5}/bar]	β_T^{REFP}/ [10^{-5}/bar]	α_P^{MC}/ [10^{-4}/K]	α_P^{REFP}/ [10^{-4}/K]	μ_{JT}^{MC}/ [10^{-3} K/bar]	μ_{JT}^{REFP}/ [10^{-3} K/bar]
20	43.5	44.4 ± 0.1	44.3	5852.5 ± 44.6	5955.0	57.5 ± 0.4	60.7	1228.0 ± 23.2	1343.7
40	109.9 ± 0.1	114.4 ± 0.8	269.7	4451.0 ± 122.9	−2243.0	138.9 ± 5.8	−425.1	1307.5 ± 87.9	–
60	894.6 ± 2.8	891.1 ± 1.6	890.8	90.6 ± 3.9	112.0	69.8 ± 2.5	76.4	39.3 ± 3.1	48.0
80	910.8 ± 3.0	906.9 ± 1.0	908.8	73.4 ± 4.3	89.9	60.5 ± 3.1	65.6	29.5 ± 4.0	36.7
100	924.4 ± 1.7	921.6 ± 0.5	923.9	64.3 ± 3.6	75.7	55.1 ± 2.5	58.4	23.5 ± 3.2	28.4
120	933.5 ± 0.8	932.4 ± 1.0	937.0	58.0 ± 2.4	65.7	51.8 ± 2.1	53.1	19.4 ± 2.7	22.0
140	945.9 ± 0.9	943.0 ± 0.8	948.7	50.1 ± 2.5	58.3	46.7 ± 2.5	49.0	13.4 ± 3.4	16.9
160	953.3 ± 0.9	952.8 ± 1.4	959.2	46.7 ± 1.6	52.5	44.5 ± 1.5	45.8	10.6 ± 2.0	12.6
180	962.4 ± 0.8	961.4 ± 1.4	968.9	43.0 ± 1.7	47.8	42.5 ± 1.6	43.1	7.9 ± 2.1	9.0
200	971.0 ± 0.7	968.9 ± 1.0	977.8	40.1 ± 3.3	44.0	40.4 ± 3.2	40.8	5.1 ± 4.3	5.9

Table S261: Heat capacities at constant volume computed from MC simulations (c_V^{MC}), heat capacities at constant volume obtained from REFPROP[10] (c_V^{REFP}), heat capacities at constant pressure computed from MC simulations (c_P^{MC}), heat capacities at constant pressure obtained from REFPROP[10] (c_P^{REFP}), speed of sound computed from MC simulations (c^{MC}), speed of sound obtained from REFPROP[10] (c^{REFP}), viscosities computed from MD simulations (η^{MD}), and viscosities obtained from REFPROP[10] (η^{REFP}) of CO_2 rich ternary mixture with 2% impurity of N_2 and 2% impurity of CH_4 at 273 K and pressures ranging from 20 bar to 200 bar.

P/ [bar]	c_V^{MC}/ [J/mol K]	c_V^{REFP}/ [J/mol K]	c_P^{MC}/ [J/mol K]	c_P^{REFP}/ [J/mol K]	c^{MC}/ [m/s]	c^{REFP}/ [m/s]	η^{MD}/ [μPa s]	η^{REFP}/ [μPa s]
20	30.8	31.2	46.0 ± 0.1	47.7	242.6 ± 1.0	240.5	6.4 ± 4.8	14.0
40	36.8 ± 0.2	–	83.8 ± 3.0	–	215.7 ± 4.9	–	14.8 ± 0.8	19.7
60	39.7 ± 0.3	40.7	111.1 ± 2.9	109.5	587.7 ± 14.9	519.3	99.7 ± 4.1	93.1
80	39.8 ± 0.3	40.4	104.7 ± 3.3	102.4	627.1 ± 21.1	557.2	103.5 ± 2.3	97.9
100	39.7 ± 0.1	40.2	100.1 ± 2.2	97.6	651.6 ± 19.7	589.1	117.8 ± 6.9	102.3
120	39.7 ± 0.1	40.1	98.2 ± 2.3	94.0	676.0 ± 16.1	617.1	111.6 ± 2.3	106.2
140	39.7 ± 0.2	40.0	93.8 ± 3.2	91.3	705.9 ± 21.5	642.2	120.0 ± 10.3	109.9
160	39.7 ± 0.1	40.0	92.3 ± 1.9	89.0	722.6 ± 14.9	665.1	116.8 ± 3.0	113.4
180	39.7 ± 0.1	40.0	91.2 ± 1.9	87.2	745.1 ± 16.7	686.2	117.0 ± 5.8	116.8
200	39.7 ± 0.1	39.9	89.1 ± 3.6	85.6	758.7 ± 35.1	705.9	129.8 ± 13.4	120.0

Table S262: Densities computed from MC and MD simulations (ρ^{MC} and ρ^{MD}), densities obtained from REFPROP[10] (ρ^{REFP}), isothermal compressibilities computed from MC simulations (β_T^{MC}), isothermal compressibilities obtained from REFPROP[10] (β_T^{REFP}), thermal expansion coefficients computed from MC simulations (α_P^{MC}), thermal expansion coefficients obtained from REFPROP[10] (α_P^{REFP}), Joule Thomson coefficients computed from MC simulations (μ_{JT}^{MC}), and Joule Thomson coefficients obtained from REFPROP[10] (μ_{JT}^{REFP}) of CO_2 rich ternary mixture with 2% impurity of N_2 and 2% impurity of CH_4 at 293 K and pressures ranging from 20 bar to 200 bar.

P/ [bar]	ρ^{MC}/ [kg/m^3]	ρ^{MD}/ [kg/m^3]	ρ^{REFP}/ [kg/m^3]	β_T^{MC}/ [10^{-5}/bar]	β_T^{REFP}/ [10^{-5}/bar]	α_P^{MC}/ [10^{-4}/K]	α_P^{REFP}/ [10^{-4}/K]	μ_{JT}^{MC}/ [10^{-3} K/bar]	μ_{JT}^{REFP}/ [10^{-3} K/bar]
20	39.2	40.0 ± 0.1	39.7	5580.4 ± 26.4	5667.2	47.8 ± 0.2	49.8	1005.5 ± 15.9	1111.5
40	90.5 ± 0.1	92.6 ± 0.7	93.6	3368.2 ± 21.3	3527.1	77.1 ± 0.5	85.3	1028.8 ± 13.3	1104.6
60	177.0 ± 0.7	201.2 ± 11.5	194.1	3835.0 ± 86.6	4650.1	214.0 ± 6.6	293.4	1018.5 ± 48.7	1022.7
80	758.9 ± 3.7	753.7 ± 2.9	744.1	322.1 ± 20.5	424.6	149.8 ± 8.4	173.1	121.3 ± 10.2	144.9
100	796.3 ± 3.2	795.3 ± 1.6	790.3	202.1 ± 10.2	224.4	109.8 ± 4.3	109.4	88.3 ± 5.4	95.1
120	823.2 ± 1.0	821.0 ± 1.0	820.3	134.1 ± 10.4	156.6	82.0 ± 5.6	85.3	62.8 ± 7.8	70.3
140	842.7 ± 2.9	840.7 ± 1.3	843.2	103.4 ± 4.2	121.5	68.6 ± 2.4	71.9	47.9 ± 3.5	54.4
160	859.5 ± 1.3	857.6 ± 0.9	861.9	86.6 ± 5.8	99.8	60.3 ± 3.8	63.1	38.0 ± 5.6	43.1
180	873.0 ± 1.7	871.3 ± 1.2	877.9	76.0 ± 4.7	85.0	55.2 ± 3.5	56.7	31.3 ± 5.3	34.5
200	886.8 ± 0.9	884.2 ± 1.2	892.0	67.9 ± 2.2	74.2	52.1 ± 1.5	51.9	26.6 ± 2.2	27.6

Table S263: Heat capacities at constant volume computed from MC simulations (c_V^{MC}), heat capacities at constant volume obtained from REFPROP[10] (c_V^{REFP}), heat capacities at constant pressure computed from MC simulations (c_P^{MC}), heat capacities at constant pressure obtained from REFPROP[10] (c_P^{REFP}), speed of sound computed from MC simulations (c^{MC}), speed of sound obtained from REFPROP[10] (c^{REFP}), viscosities computed from MD simulations (η^{MD}), and viscosities obtained from REFPROP[10] (η^{REFP}) of CO_2 rich ternary mixture with 2% impurity of N_2 and 2% impurity of CH_4 at 293 K and pressures ranging from 20 bar to 200 bar.

P/ [bar]	c_V^{MC}/ [J/mol K]	c_V^{REFP}/ [J/mol K]	c_P^{MC}/ [J/mol K]	c_P^{REFP}/ [J/mol K]	c^{MC}/ [m/s]	c^{REFP}/ [m/s]	η^{MD}/ [μPa s]	η^{REFP}/ [μPa s]
20	30.7	30.9	43.9 ± 0.1	44.8	255.9 ± 0.6	253.7	5.3 ± 6.1	15.0
40	33.7	34.7	58.3 ± 0.3	62.6	238.4 ± 0.9	233.7	18.4 ± 2.7	15.7
60	39.7 ± 0.3	44.5	126.1 ± 3.8	165.1	216.3 ± 4.2	202.6	16.7 ± 0.1	18.0
80	40.0 ± 0.1	43.0	158.8 ± 6.6	162.9	402.9 ± 15.3	346.3	66.6 ± 2.1	63.3
100	39.7 ± 0.1	41.3	135.9 ± 3.0	126.6	461.2 ± 12.8	415.7	85.4 ± 22.6	71.1
120	39.3 ± 0.1	40.6	117.0 ± 4.8	112.2	519.0 ± 22.8	463.8	80.7 ± 3.6	76.8
140	39.3 ± 0.2	40.2	108.0 ± 2.4	103.9	561.7 ± 13.1	502.2	88.2 ± 3.0	81.6
160	39.3 ± 0.2	40.0	101.3 ± 3.7	98.4	588.7 ± 22.6	534.7	91.7 ± 8.7	85.7
180	39.3 ± 0.1	39.8	97.5 ± 3.9	94.4	611.9 ± 22.5	563.3	95.3 ± 5.4	89.5
200	39.2 ± 0.1	39.8	96.4 ± 1.5	91.3	639.2 ± 11.6	589.0	103.6 ± 9.9	93.0

Table S264: Densities computed from MC and MD simulations (ρ^{MC} and ρ^{MD}), densities obtained from REFPROP[10] (ρ^{REFP}), isothermal compressibilities computed from MC simulations (β_T^{MC}), isothermal compressibilities obtained from REFPROP[10] (β_T^{REFP}), thermal expansion coefficients computed from MC simulations (α_P^{MC}), thermal expansion coefficients obtained from REFPROP[10] (α_P^{REFP}), Joule Thomson coefficients computed from MC simulations (μ_{JT}^{MC}), and Joule Thomson coefficients obtained from REFPROP[10] (μ_{JT}^{REFP}) of CO_2 rich ternary mixture with 2% impurity of N_2 and 2% impurity of CH_4 at 313 K and pressures ranging from 20 bar to 200 bar.

P/ [bar]	ρ^{MC}/ [kg/m^3]	ρ^{MD}/ [kg/m^3]	ρ^{REFP}/ [kg/m^3]	β_T^{MC}/ [10^{-5}/bar]	β_T^{REFP}/ [10^{-5}/bar]	α_P^{MC}/ [10^{-4}/K]	α_P^{REFP}/ [10^{-4}/K]	μ_{JT}^{MC}/ [10^{-3} K/bar]	μ_{JT}^{REFP}/ [10^{-3} K/bar]
20	35.8	36.4 ± 0.1	36.2	5433.8 ± 27.7	5494.2	41.7 ± 0.2	42.9	853.4 ± 20.9	936.0
40	79.2	80.9 ± 0.2	81.1	3043.9 ± 40.8	3141.0	57.6 ± 0.8	61.7	850.5 ± 26.6	924.6
60	136.0 ± 0.1	140.1 ± 0.7	141.7	2490.2 ± 34.0	2592.2	92.0 ± 1.7	101.3	861.9 ± 26.5	889.7
80	224.4 ± 0.9	242.1 ± 4.7	242.6	2697.3 ± 113.7	2951.3	191.7 ± 8.0	230.5	790.3 ± 44.2	–
100	464.0 ± 13.4	474.2 ± 8.1	475.4	3204.8 ± 164.3	2767.4	593.3 ± 41.9	506.4	440.6 ± 47.0	434.6
120	640.3 ± 4.5	629.7 ± 3.4	630.7	653.6 ± 68.2	710.2	213.1 ± 21.2	209.0	201.6 ± 28.4	217.0
140	701.3 ± 3.3	695.4 ± 3.1	696.1	316.1 ± 15.6	353.4	128.5 ± 6.7	129.5	131.6 ± 10.5	141.8
160	734.8 ± 3.5	733.1 ± 2.9	736.8	196.7 ± 11.5	232.5	91.1 ± 4.3	97.9	92.4 ± 7.2	104.1
180	762.9 ± 1.5	761.8 ± 1.8	766.9	160.6 ± 13.1	173.1	80.4 ± 5.3	80.6	77.1 ± 8.9	80.7
200	786.2 ± 1.9	783.6 ± 1.7	790.9	133.4 ± 2.6	137.8	72.8 ± 0.9	69.6	65.0 ± 1.6	64.4

Table S265: Heat capacities at constant volume computed from MC simulations (c_V^{MC}), heat capacities at constant volume obtained from REFPROP[10] (c_V^{REFP}), heat capacities at constant pressure computed from MC simulations (c_P^{MC}), heat capacities at constant pressure obtained from REFPROP[10] (c_P^{REFP}), speed of sound computed from MC simulations (c^{MC}), speed of sound obtained from REFPROP[10] (c^{REFP}), viscosities computed from MD simulations (η^{MD}), and viscosities obtained from REFPROP[10] (η^{REFP}) of CO_2 rich ternary mixture with 2% impurity of N_2 and 2% impurity of CH_4 at 313 K and pressures ranging from 20 bar to 200 bar.

P/ [bar]	c_V^{MC}/ [J/mol K]	c_V^{REFP}/ [J/mol K]	c_P^{MC}/ [J/mol K]	c_P^{REFP}/ [J/mol K]	c^{MC}/ [m/s]	c^{REFP}/ [m/s]	η^{MD}/ [μPa s]	η^{REFP}/ [μPa s]
20	31.1	31.1	43.1 ± 0.1	43.6	267.1 ± 0.7	265.3	9.9 ± 5.2	15.9
40	32.9	33.4	51.5 ± 0.3	53.6	254.9 ± 1.8	251.0	17.0 ± 3.0	16.5
60	35.4	36.5	69.2 ± 0.8	74.2	240.4 ± 2.1	235.2	17.8 ± 2.4	17.7
80	39.3 ± 0.3	–	121.6 ± 3.0	–	226.0 ± 5.6	–	21.3 ± 2.2	20.7
100	42.6 ± 0.5	46.9	371.1 ± 26.3	310.1	242.3 ± 11.2	224.2	33.8 ± 5.0	33.8
120	40.3 ± 0.3	43.0	189.5 ± 14.7	174.7	335.3 ± 21.8	301.2	49.4 ± 4.6	48.6
140	39.6 ± 0.4	41.4	141.3 ± 5.4	133.3	401.0 ± 12.7	362.1	58.3 ± 5.8	56.8
160	39.3 ± 0.3	40.6	117.5 ± 3.1	116.0	454.7 ± 14.8	408.6	63.3 ± 4.1	62.7
180	39.1	40.1	111.3 ± 4.0	106.3	481.9 ± 21.5	446.7	68.7 ± 4.7	67.5
200	39.1 ± 0.2	39.9	108.0 ± 1.0	99.9	512.9 ± 5.7	479.4	72.3 ± 4.3	71.6

S16.13 Data of thermodynamics and transport properties of CO_2 rich ternary mixture with 1 mole% impurity of H_2 and 3 mole% impurity of CH_4

Table S266: Densities computed from MC and MD simulations (ρ^{MC} and ρ^{MD}), densities obtained from REFPROP[10] (ρ^{REFP}), isothermal compressibilities computed from MC simulations (β_T^{MC}), isothermal compressibilities obtained from REFPROP[10] (β_T^{REFP}), thermal expansion coefficients computed from MC simulations (α_P^{MC}), thermal expansion coefficients obtained from REFPROP[10] (α_P^{REFP}), Joule Thomson coefficients computed from MC simulations (μ_{JT}^{MC}), and Joule Thomson coefficients obtained from REFPROP[10] (μ_{JT}^{REFP}) of CO_2 rich ternary mixture with 3% impurity of CH_4 and 1% impurity of H_2 at 253 K and pressures ranging from 20 bar to 200 bar.

P/ [bar]	ρ^{MC}/ [kg/m^3]	ρ^{MD}/ [kg/m^3]	ρ^{REFP}/ [kg/m^3]	β_T^{MC}/ [10^{-5}/bar]	β_T^{REFP}/ [10^{-5}/bar]	α_P^{MC}/ [10^{-4}/K]	α_P^{REFP}/ [10^{-4}/K]	μ_{JT}^{MC}/ [10^{-3} K/bar]	μ_{JT}^{REFP}/ [10^{-3} K/bar]
20	49.0	49.2 ± 0.2	50.5	6269.9 ± 64.7	6540.1	74.0 ± 0.7	82.4	1494.8 ± 31.8	1659.5
40	984.8 ± 0.9	974.9 ± 0.7	924.2	45.1 ± 0.9	107.1	48.3 ± 1.4	83.4	9.9 ± 1.6	–
60	992.3 ± 1.4	984.2 ± 0.9	989.7	41.0 ± 2.2	46.2	45.2 ± 2.4	46.4	6.5 ± 2.8	8.3
80	1002.3 ± 1.7	992.9 ± 1.3	998.5	37.1 ± 2.5	42.2	42.5 ± 2.6	43.7	3.5 ± 3.1	5.0
100	1008.1 ± 1.5	1001.3 ± 1.1	1006.6	34.0 ± 0.7	38.9	40.1 ± 0.8	41.4	0.7 ± 0.9	2.3
120	1015.7 ± 1.1	1008.1 ± 0.3	1014.2	32.9 ± 1.1	36.1	39.2 ± 1.7	39.4	-0.3 ± 1.9	-0.1
140	1022.0 ± 1.5	1014.8 ± 0.7	1021.3	29.5 ± 0.6	33.7	36.3 ± 0.7	37.7	-4.0 ± 0.8	-2.3
160	1028.3 ± 0.7	1021.5 ± 1.1	1028.0	28.0 ± 1.4	31.7	34.9 ± 1.6	36.2	-5.7 ± 2.0	-4.2
180	1034.2 ± 0.8	1027.0 ± 0.5	1034.3	28.3 ± 1.6	29.9	36.0 ± 2.0	34.9	-4.2 ± 2.4	-5.9
200	1039.1 ± 0.9	1033.0 ± 0.3	1040.3	25.8 ± 0.4	28.3	33.4 ± 0.9	33.7	-7.5 ± 1.1	-7.4

Table S267: Heat capacities at constant volume computed from MC simulations (c_V^{MC}), heat capacities at constant volume obtained from REFPROP[10] (c_V^{REFP}), heat capacities at constant pressure computed from MC simulations (c_P^{MC}), heat capacities at constant pressure obtained from REFPROP[10] (c_P^{REFP}), speed of sound computed from MC simulations (c^{MC}), speed of sound obtained from REFPROP[10] (c^{REFP}), viscosities computed from MD simulations (η^{MD}), and viscosities obtained from REFPROP[10] (η^{REFP}) of CO_2 rich ternary mixture with 3% impurity of CH_4 and 1% impurity of H_2 at 253 K and pressures ranging from 20 bar to 200 bar.

P/ [bar]	c_V^{MC}/ [J/mol K]	c_V^{REFP}/ [J/mol K]	c_P^{MC}/ [J/mol K]	c_P^{REFP}/ [J/mol K]	c^{MC}/ [m/s]	c^{REFP}/ [m/s]	η^{MD}/ [μPa s]	η^{REFP}/ [μPa s]
20	31.6	33.1	50.9 ± 0.2	55.3	229.0 ± 1.2	225.0	12.3 ± 4.1	12.9
40	40.1 ± 0.2	–	97.2 ± 2.3	–	739.3 ± 11.5	–	133.8 ± 8.5	105.0
60	40.2 ± 0.3	40.2	95.0 ± 3.1	91.1	761.9 ± 24.1	704.2	131.2 ± 2.1	128.8
80	40.0 ± 0.4	40.2	92.7 ± 3.1	89.2	789.0 ± 29.9	726.0	136.0 ± 6.5	132.6
100	40.3 ± 0.1	40.2	91.2 ± 1.1	87.5	812.3 ± 9.2	746.2	134.9 ± 6.2	136.2
120	40.4 ± 0.2	40.2	90.4 ± 2.7	86.1	818.4 ± 18.1	765.1	166.9 ± 6.7	139.7
140	40.4 ± 0.3	40.2	87.4 ± 1.1	84.8	847.2 ± 10.9	782.8	142.6 ± 4.4	143.1
160	40.5 ± 0.2	40.2	86.2 ± 2.2	83.8	859.6 ± 23.8	799.5	152.7 ± 9.0	146.4
180	40.7 ± 0.3	40.3	88.5 ± 3.1	82.8	863.1 ± 28.1	815.4	150.4 ± 1.2	149.6
200	40.6 ± 0.1	40.3	85.4 ± 1.7	82.0	885.4 ± 11.7	830.5	165.4 ± 12.6	152.7

Table S268: Densities computed from MC and MD simulations (ρ^{MC} and ρ^{MD}), densities obtained from REFPROP[10] (ρ^{REFP}), isothermal compressibilities computed from MC simulations (β_T^{MC}), isothermal compressibilities obtained from REFPROP[10] (β_T^{REFP}), thermal expansion coefficients computed from MC simulations (α_P^{MC}), thermal expansion coefficients obtained from REFPROP[10] (α_P^{REFP}), Joule Thomson coefficients computed from MC simulations ($\mu_{\mathrm{JT}}^{\mathrm{MC}}$), and Joule Thomson coefficients obtained from REFPROP[10] ($\mu_{\mathrm{JT}}^{\mathrm{REFP}}$) of CO_2 rich ternary mixture with 3% impurity of CH_4 and 1% impurity of H_2 at 273 K and pressures ranging from 20 bar to 200 bar.

P/ [bar]	ρ^{MC}/ [kg/m^3]	ρ^{MD}/ [kg/m^3]	ρ^{REFP}/ [kg/m^3]	β_T^{MC}/ [10^{-5}/bar]	β_T^{REFP}/ [10^{-5}/bar]	α_P^{MC}/ [10^{-4}/K]	α_P^{REFP}/ [10^{-4}/K]	$\mu_{\mathrm{JT}}^{\mathrm{MC}}$/ [$10^{-3}$ K/bar]	$\mu_{\mathrm{JT}}^{\mathrm{REFP}}$/ [$10^{-3}$ K/bar]
20	43.1	43.2 ± 0.1	43.9	5841.5 ± 43.3	5950.7	57.5 ± 0.5	60.5	1224.1 ± 26.9	1335.4
40	108.1 ± 0.2	851.1 ± 1.5	273.2	4339.4 ± 127.3	−2101.5	132.3 ± 4.3	−419.2	1282.8 ± 62.9	–
60	891.2 ± 1.1	870.9 ± 1.3	881.5	86.6 ± 4.3	109.4	67.4 ± 3.6	75.0	37.0 ± 4.5	46.8
80	906.0 ± 1.1	890.0 ± 1.3	899.0	75.2 ± 4.9	88.3	62.5 ± 3.6	64.7	31.2 ± 4.5	35.8
100	917.6 ± 1.5	905.5 ± 0.5	913.7	62.1 ± 5.1	74.6	53.8 ± 4.3	57.7	22.0 ± 5.7	27.8
120	929.8 ± 0.7	916.6 ± 1.1	926.5	54.9 ± 2.6	64.9	49.2 ± 2.4	52.6	16.6 ± 3.2	21.5
140	939.6 ± 2.0	927.9 ± 1.0	937.9	50.2 ± 0.5	57.7	46.7 ± 0.6	48.6	13.3 ± 0.8	16.4
160	948.3 ± 1.0	938.9 ± 1.3	948.2	46.5 ± 2.1	52.0	44.6 ± 1.6	45.4	10.6 ± 2.1	12.2
180	956.6 ± 0.7	948.2 ± 1.0	957.6	43.8 ± 1.8	47.4	43.2 ± 1.8	42.8	8.7 ± 2.4	8.6
200	964.7 ± 0.8	956.5 ± 0.9	966.4	39.6 ± 1.7	43.7	39.8 ± 1.9	40.5	4.3 ± 2.6	5.5

Table S269: Heat capacities at constant volume computed from MC simulations (c_V^{MC}), heat capacities at constant volume obtained from REFPROP[10] (c_V^{REFP}), heat capacities at constant pressure computed from MC simulations (c_P^{MC}), heat capacities at constant pressure obtained from REFPROP[10] (c_P^{REFP}), speed of sound computed from MC simulations (c^{MC}), speed of sound obtained from REFPROP[10] (c^{REFP}), viscosities computed from MD simulations (η^{MD}), and viscosities obtained from REFPROP[10] (η^{REFP}) of CO_2 rich ternary mixture with 3% impurity of CH_4 and 1% impurity of H_2 at 273 K and pressures ranging from 20 bar to 200 bar.

P/ [bar]	c_V^{MC}/ [J/mol K]	c_V^{REFP}/ [J/mol K]	c_P^{MC}/ [J/mol K]	c_P^{REFP}/ [J/mol K]	c^{MC}/ [m/s]	c^{REFP}/ [m/s]	η^{MD}/ [μPa s]	η^{REFP}/ [μPa s]
20	30.8	31.2	46.1 ± 0.1	47.5	243.9 ± 1.0	241.5	9.0 ± 4.6	13.9
40	37.1 ± 0.2	–	80.5 ± 1.6	–	215.2 ± 3.8	–	86.6 ± 0.9	19.3
60	39.6 ± 0.2	40.4	109.0 ± 4.0	108.5	596.8 ± 18.6	527.7	90.3 ± 1.4	91.6
80	39.5 ± 0.2	40.1	107.0 ± 3.6	101.7	630.0 ± 23.2	565.0	98.7 ± 8.6	96.3
100	39.8 ± 0.1	40.0	99.3 ± 4.8	97.0	661.8 ± 31.4	596.6	100.9 ± 22.7	100.5
120	39.8 ± 0.1	39.9	95.1 ± 2.8	93.5	684.4 ± 19.3	624.3	102.7 ± 5.3	104.3
140	39.5 ± 0.2	39.8	93.6 ± 1.2	90.8	708.4 ± 6.2	649.2	108.5 ± 5.9	108.0
160	39.7 ± 0.1	39.8	92.4 ± 1.5	88.6	726.1 ± 17.4	671.9	113.6 ± 4.6	111.4
180	39.6 ± 0.2	39.8	91.8 ± 2.5	86.8	743.3 ± 18.4	692.9	112.8 ± 4.2	114.6
200	39.8 ± 0.2	39.8	88.2 ± 2.5	85.2	762.6 ± 20.2	712.5	124.3 ± 7.5	117.8

Table S270: Densities computed from MC and MD simulations (ρ^{MC} and ρ^{MD}), densities obtained from REFPROP[10] (ρ^{REFP}), isothermal compressibilities computed from MC simulations (β_T^{MC}), isothermal compressibilities obtained from REFPROP[10] (β_T^{REFP}), thermal expansion coefficients computed from MC simulations (α_P^{MC}), thermal expansion coefficients obtained from REFPROP[10] (α_P^{REFP}), Joule Thomson coefficients computed from MC simulations ($\mu_{\mathrm{JT}}^{\mathrm{MC}}$), and Joule Thomson coefficients obtained from REFPROP[10] ($\mu_{\mathrm{JT}}^{\mathrm{REFP}}$) of CO_2 rich ternary mixture with 3% impurity of CH_4 and 1% impurity of H_2 at 293 K and pressures ranging from 20 bar to 200 bar.

P/ [bar]	ρ^{MC}/ [kg/m^3]	ρ^{MD}/ [kg/m^3]	ρ^{REFP}/ [kg/m^3]	β_T^{MC}/ [10^{-5}/bar]	β_T^{REFP}/ [10^{-5}/bar]	α_P^{MC}/ [10^{-4}/K]	α_P^{REFP}/ [10^{-4}/K]	$\mu_{\mathrm{JT}}^{\mathrm{MC}}$/ [$10^{-3}$ K/bar]	$\mu_{\mathrm{JT}}^{\mathrm{REFP}}$/ [$10^{-3}$ K/bar]
20	38.8	39.0 ± 0.1	39.4	5599.6 ± 19.2	5665.3	48.0 ± 0.1	49.7	1013.6 ± 8.7	1104.3
40	89.7 ± 0.1	89.7 ± 0.2	92.7	3369.6 ± 26.8	3523.1	77.0 ± 0.7	84.9	1027.0 ± 16.8	1101.5
60	174.6 ± 0.3	174.8 ± 1.2	191.5	3858.8 ± 162.0	4586.2	212.1 ± 10.8	286.4	1029.9 ± 75.7	1023.4
80	757.5 ± 2.2	712.9 ± 4.4	740.2	295.0 ± 42.5	396.0	142.2 ± 16.4	164.7	114.9 ± 19.4	139.5
100	792.0 ± 2.2	764.9 ± 2.5	783.8	170.2 ± 11.2	216.3	94.1 ± 4.5	106.6	77.0 ± 6.1	92.7
120	816.3 ± 2.3	794.3 ± 1.6	812.6	135.9 ± 8.3	152.6	82.6 ± 3.8	83.7	63.5 ± 5.3	68.8
140	838.9 ± 2.0	818.4 ± 1.5	834.7	106.2 ± 6.3	119.1	70.2 ± 3.4	70.8	49.3 ± 4.8	53.3
160	855.3 ± 1.2	838.0 ± 0.8	852.9	89.9 ± 5.0	98.2	62.5 ± 2.5	62.3	40.2 ± 3.6	42.3
180	868.0 ± 1.1	853.3 ± 0.7	868.5	77.4 ± 5.4	83.8	56.2 ± 3.7	56.1	32.4 ± 5.6	33.8
200	881.2 ± 1.1	867.5 ± 0.8	882.3	67.5 ± 1.7	73.3	51.4 ± 1.1	51.4	25.8 ± 1.6	27.0

Table S271: Heat capacities at constant volume computed from MC simulations (c_V^{MC}), heat capacities at constant volume obtained from REFPROP[10] (c_V^{REFP}), heat capacities at constant pressure computed from MC simulations (c_P^{MC}), heat capacities at constant pressure obtained from REFPROP[10] (c_P^{REFP}), speed of sound computed from MC simulations (c^{MC}), speed of sound obtained from REFPROP[10] (c^{REFP}), viscosities computed from MD simulations (η^{MD}), and viscosities obtained from REFPROP[10] (η^{REFP}) of CO_2 rich ternary mixture with 3% impurity of CH_4 and 1% impurity of H_2 at 293 K and pressures ranging from 20 bar to 200 bar.

P/ [bar]	c_V^{MC}/ [J/mol K]	c_V^{REFP}/ [J/mol K]	c_P^{MC}/ [J/mol K]	c_P^{REFP}/ [J/mol K]	c^{MC}/ [m/s]	c^{REFP}/ [m/s]	η^{MD}/ [μPa s]	η^{REFP}/ [μPa s]
20	30.8	30.9	44.0	44.8	256.6 ± 0.5	254.8	10.2 ± 2.8	14.9
40	33.7	34.6	58.3 ± 0.2	62.3	239.3 ± 1.1	234.6	15.4 ± 1.1	15.6
60	40.0 ± 0.4	44.3	124.0 ± 5.1	161.2	214.5 ± 6.4	203.6	17.0 ± 0.9	17.8
80	40.1 ± 0.3	42.5	155.6 ± 11.4	158.5	416.5 ± 33.7	356.7	60.6 ± 1.9	62.9
100	39.7 ± 0.3	41.0	123.2 ± 3.2	124.9	479.5 ± 17.0	424.1	65.6 ± 2.3	70.3
120	39.5 ± 0.2	40.3	117.1 ± 2.8	111.1	517.0 ± 17.1	471.4	76.1 ± 5.9	75.8
140	39.3 ± 0.1	40.0	109.2 ± 2.9	103.1	558.6 ± 18.3	509.3	78.7 ± 6.5	80.4
160	39.3 ± 0.1	39.8	103.3 ± 1.9	97.7	584.6 ± 17.1	541.6	84.7 ± 3.7	84.4
180	39.3 ± 0.2	39.7	98.5 ± 3.9	93.8	610.7 ± 24.4	570.0	100.2 ± 18.2	88.1
200	39.3 ± 0.1	39.6	95.2 ± 1.0	90.8	638.1 ± 8.8	595.5	94.1 ± 7.7	91.5

Table S272: Densities computed from MC and MD simulations (ρ^{MC} and ρ^{MD}), densities obtained from REFPROP[10] (ρ^{REFP}), isothermal compressibilities computed from MC simulations (β_T^{MC}), isothermal compressibilities obtained from REFPROP[10] (β_T^{REFP}), thermal expansion coefficients computed from MC simulations (α_P^{MC}), thermal expansion coefficients obtained from REFPROP[10] (α_P^{REFP}), Joule Thomson coefficients computed from MC simulations (μ_{JT}^{MC}), and Joule Thomson coefficients obtained from REFPROP[10] (μ_{JT}^{REFP}) of CO_2 rich ternary mixture with 3% impurity of CH_4 and 1% impurity of H_2 at 313 K and pressures ranging from 20 bar to 200 bar.

P/ [bar]	ρ^{MC}/ [kg/m^3]	ρ^{MD}/ [kg/m^3]	ρ^{REFP}/ [kg/m^3]	β_T^{MC}/ [10^{-5}/bar]	β_T^{REFP}/ [10^{-5}/bar]	α_P^{MC}/ [10^{-4}/K]	α_P^{REFP}/ [10^{-4}/K]	μ_{JT}^{MC}/ [10^{-3} K/bar]	μ_{JT}^{REFP}/ [10^{-3} K/bar]
20	35.5	35.7 ± 0.1	35.9	5444.4 ± 14.1	5493.4	41.8 ± 0.1	42.8	860.5 ± 10.8	930.1
40	78.4	78.7 ± 0.2	80.3	3072.2 ± 21.6	3140.2	58.2 ± 0.5	61.5	865.3 ± 18.2	921.7
60	134.6 ± 0.1	134.4 ± 0.6	140.4	2490.8 ± 49.2	2589.6	91.8 ± 2.1	100.8	860.5 ± 31.8	888.6
80	222.3 ± 0.7	219.3 ± 0.9	240.1	2748.7 ± 92.5	2945.9	196.2 ± 8.5	228.7	797.4 ± 49.2	–
100	451.0 ± 5.7	401.7 ± 7.3	475.8	3574.8 ± 216.7	2824.9	640.1 ± 37.4	524.1	461.6 ± 36.2	432.7
120	632.4 ± 3.9	577.2 ± 3.5	628.6	694.9 ± 92.9	684.4	220.3 ± 24.8	204.8	208.2 ± 31.5	212.6
140	693.8 ± 1.3	658.0 ± 1.9	691.5	325.1 ± 31.7	342.9	131.5 ± 10.6	127.0	133.9 ± 15.8	139.3
160	730.6 ± 4.1	705.0 ± 2.3	730.9	207.3 ± 15.2	227.0	95.2 ± 6.0	96.3	96.4 ± 10.0	102.4
180	758.2 ± 0.9	737.1 ± 0.8	760.0	160.2 ± 10.2	169.7	81.6 ± 4.7	79.5	77.4 ± 7.8	79.4
200	781.7 ± 2.1	760.9 ± 1.0	783.4	125.2 ± 6.1	135.5	68.8 ± 3.6	68.8	60.6 ± 6.2	63.3

Table S273: Heat capacities at constant volume computed from MC simulations (c_V^{MC}), heat capacities at constant volume obtained from REFPROP[10] (c_V^{REFP}), heat capacities at constant pressure computed from MC simulations (c_P^{MC}), heat capacities at constant pressure obtained from REFPROP[10] (c_P^{REFP}), speed of sound computed from MC simulations (c^{MC}), speed of sound obtained from REFPROP[10] (c^{REFP}), viscosities computed from MD simulations (η^{MD}), and viscosities obtained from REFPROP[10] (η^{REFP}) of CO_2 rich ternary mixture with 3% impurity of CH_4 and 1% impurity of H_2 at 313 K and pressures ranging from 20 bar to 200 bar.

P/ [bar]	c_V^{MC}/ [J/mol K]	c_V^{REFP}/ [J/mol K]	c_P^{MC}/ [J/mol K]	c_P^{REFP}/ [J/mol K]	c^{MC}/ [m/s]	c^{REFP}/ [m/s]	η^{MD}/ [μPa s]	η^{REFP}/ [μPa s]
20	31.1	31.1	43.2	43.6	268.2 ± 0.4	266.3	7.8 ± 6.4	15.9
40	32.9	33.4	51.7 ± 0.2	53.4	255.3 ± 1.0	252.0	17.4 ± 1.9	16.4
60	35.5 ± 0.1	36.4	69.1 ± 0.8	73.8	241.1 ± 2.8	236.1	19.0 ± 3.0	17.6
80	39.1 ± 0.2	–	124.0 ± 4.2	–	227.8 ± 5.5	–	20.2 ± 2.7	20.5
100	42.7 ± 0.8	46.4	391.2 ± 18.4	319.9	238.3 ± 9.6	226.4	26.2 ± 4.0	33.8
120	40.7 ± 0.3	42.6	191.5 ± 14.4	173.0	327.3 ± 25.1	307.3	46.3 ± 10.4	48.4
140	39.7 ± 0.2	41.0	143.4 ± 7.2	132.1	400.3 ± 22.0	368.5	56.2 ± 11.6	56.4
160	39.5 ± 0.1	40.3	120.0 ± 4.9	115.2	448.1 ± 18.8	414.9	59.6 ± 6.2	62.1
180	39.3 ± 0.1	39.9	113.2 ± 4.0	105.6	487.3 ± 17.8	452.9	66.5 ± 5.3	66.7
200	39.3 ± 0.2	39.7	104.2 ± 3.6	99.3	520.8 ± 15.7	485.5	67.4 ± 3.1	70.7

S16.14 Data of thermodynamics and transport properties of CO_2 rich ternary mixture with 3 mole% impurity of H_2 and 1 mole% impurity of CH_4

Table S274: Densities computed from MC and MD simulations (ρ^{MC} and ρ^{MD}), densities obtained from REFPROP[10] (ρ^{REFP}), isothermal compressibilities computed from MC simulations (β_T^{MC}), isothermal compressibilities obtained from REFPROP[10] (β_T^{REFP}), thermal expansion coefficients computed from MC simulations (α_P^{MC}), thermal expansion coefficients obtained from REFPROP[10] (α_P^{REFP}), Joule Thomson coefficients computed from MC simulations (μ_{JT}^{MC}), and Joule Thomson coefficients obtained from REFPROP[10] (μ_{JT}^{REFP}) of CO_2 rich ternary mixture with 3% impurity of H_2 and 1% impurity of CH_4 at 253 K and pressures ranging from 20 bar to 200 bar.

P/ [bar]	ρ^{MC}/ [kg/m^3]	ρ^{MD}/ [kg/m^3]	ρ^{REFP}/ [kg/m^3]	β_T^{MC}/ [10^{-5}/bar]	β_T^{REFP}/ [10^{-5}/bar]	α_P^{MC}/ [10^{-4}/K]	α_P^{REFP}/ [10^{-4}/K]	μ_{JT}^{MC}/ [10^{-3} K/bar]	μ_{JT}^{REFP}/ [10^{-3} K/bar]
20	48.4	49.2 ± 0.2	49.8	6313.5 ± 46.1	6463.5	74.1 ± 0.6	79.9	1509.4 ± 26.8	1620.7
40	977.5 ± 2.3	974.9 ± 0.7	658.9	50.6 ± 3.1	-40.6	52.8 ± 2.3	62.0	14.4 ± 2.5	–
60	985.7 ± 0.5	984.2 ± 0.9	894.8	44.6 ± 1.8	149.1	47.7 ± 2.1	105.3	9.3 ± 2.4	–
80	993.8 ± 1.2	992.9 ± 1.3	984.3	40.2 ± 2.9	44.5	44.3 ± 3.0	44.8	5.6 ± 3.5	6.5
100	1002.0 ± 0.9	1001.3 ± 1.1	992.7	37.9 ± 0.7	40.8	43.0 ± 0.7	42.3	4.1 ± 0.8	3.5
120	1008.2 ± 0.6	1008.1 ± 0.3	1000.5	37.0 ± 1.6	37.8	42.9 ± 1.8	40.2	3.9 ± 2.0	0.9
140	1016.3 ± 0.9	1014.8 ± 0.7	1007.8	32.4 ± 0.9	35.2	38.6 ± 1.1	38.4	-1.2 ± 1.3	-1.4
160	1023.2 ± 0.4	1021.5 ± 1.1	1014.7	30.6 ± 1.3	33.0	37.5 ± 1.5	36.8	-2.4 ± 1.8	-3.4
180	1029.5 ± 0.8	1027.0 ± 0.5	1021.3	28.6 ± 0.6	31.1	35.7 ± 0.9	35.4	-4.6 ± 1.1	-5.2
200	1034.8 ± 1.5	1033.0 ± 0.3	1027.4	27.6 ± 1.4	29.4	35.1 ± 1.5	34.2	-5.3 ± 1.8	-6.8

Table S275: Heat capacities at constant volume computed from MC simulations (c_V^{MC}), heat capacities at constant volume obtained from REFPROP[10] (c_V^{REFP}), heat capacities at constant pressure computed from MC simulations (c_P^{MC}), heat capacities at constant pressure obtained from REFPROP[10] (c_P^{REFP}), speed of sound computed from MC simulations (c^{MC}), speed of sound obtained from REFPROP[10] (c^{REFP}), viscosities computed from MD simulations (η^{MD}), and viscosities obtained from REFPROP[10] (η^{REFP}) of CO_2 rich ternary mixture with 3% impurity of H_2 and 1% impurity of CH_4 at 253 K and pressures ranging from 20 bar to 200 bar.

P/ [bar]	c_V^{MC}/ [J/mol K]	c_V^{REFP}/ [J/mol K]	c_P^{MC}/ [J/mol K]	c_P^{REFP}/ [J/mol K]	c^{MC}/ [m/s]	c^{REFP}/ [m/s]	η^{MD}/ [μPa s]	η^{REFP}/ [μPa s]
20	31.4	32.4	50.8 ± 0.2	53.7	229.8 ± 0.9	226.8	12.3 ± 4.1	12.9
40	40.3 ± 0.3	–	101.1 ± 1.9	–	712.0 ± 22.9	–	133.8 ± 8.5	58.2
60	40.3 ± 0.2	–	96.2 ± 2.8	–	736.4 ± 18.4	–	131.2 ± 2.1	95.7
80	40.1 ± 0.1	40.0	93.4 ± 3.6	89.3	763.3 ± 31.0	714.3	136.0 ± 6.5	126.6
100	40.3 ± 0.2	40.0	92.7 ± 1.2	87.5	778.4 ± 9.0	735.1	134.9 ± 6.2	130.2
120	40.2 ± 0.3	40.0	93.4 ± 2.2	86.0	789.1 ± 19.9	754.5	166.9 ± 6.7	133.6
140	40.5 ± 0.1	40.0	88.9 ± 1.8	84.7	816.1 ± 14.0	772.6	142.6 ± 4.4	137.0
160	40.5 ± 0.2	40.0	88.6 ± 1.8	83.6	836.9 ± 20.2	789.7	152.7 ± 9.0	140.2
180	40.4 ± 0.1	40.0	86.9 ± 1.5	82.6	854.9 ± 11.6	805.9	150.4 ± 1.2	143.4
200	40.5 ± 0.1	40.1	86.8 ± 1.9	81.7	865.7 ± 23.4	821.3	165.4 ± 12.6	146.4

Table S276: Densities computed from MC and MD simulations (ρ^{MC} and ρ^{MD}), densities obtained from REFPROP[10] (ρ^{REFP}), isothermal compressibilities computed from MC simulations (β_T^{MC}), isothermal compressibilities obtained from REFPROP[10] (β_T^{REFP}), thermal expansion coefficients computed from MC simulations (α_P^{MC}), thermal expansion coefficients obtained from REFPROP[10] (α_P^{REFP}), Joule Thomson coefficients computed from MC simulations ($\mu_{\mathrm{JT}}^{\mathrm{MC}}$), and Joule Thomson coefficients obtained from REFPROP[10] ($\mu_{\mathrm{JT}}^{\mathrm{REFP}}$) of CO_2 rich ternary mixture with 3% impurity of H_2 and 1% impurity of CH_4 at 273 K and pressures ranging from 20 bar to 200 bar.

P/ [bar]	ρ^{MC}/ [kg/m^3]	ρ^{MD}/ [kg/m^3]	ρ^{REFP}/ [kg/m^3]	β_T^{MC}/ [10^{-5}/bar]	β_T^{REFP}/ [10^{-5}/bar]	α_P^{MC}/ [10^{-4}/K]	α_P^{REFP}/ [10^{-4}/K]	$\mu_{\mathrm{JT}}^{\mathrm{MC}}$/ [$10^{-3}$ K/bar]	$\mu_{\mathrm{JT}}^{\mathrm{REFP}}$/ [$10^{-3}$ K/bar]
20	42.6	43.2 ± 0.1	43.5	5834.2 ± 49.8	5918.6	57.0 ± 0.4	59.6	1210.2 ± 23.8	1308.3
40	106.2 ± 0.2	851.1 ± 1.5	226.4	4187.8 ± 32.4	−7344.0	125.9 ± 1.6	−825.9	1250.1 ± 25.4	–
60	873.8 ± 1.7	870.9 ± 1.3	743.5	110.8 ± 7.8	3346.9	80.4 ± 5.6	1247.8	49.1 ± 6.6	–
80	893.7 ± 1.4	890.0 ± 1.3	882.9	84.9 ± 3.0	95.7	66.5 ± 2.0	67.7	36.0 ± 2.5	39.7
100	905.7 ± 0.6	905.5 ± 0.5	898.4	72.3 ± 3.6	79.9	59.1 ± 2.4	59.9	28.2 ± 3.0	30.7
120	918.1 ± 0.9	916.6 ± 1.1	911.9	64.1 ± 2.6	68.9	54.7 ± 2.8	54.2	23.1 ± 3.6	23.8
140	931.9 ± 1.0	927.9 ± 1.0	923.7	54.0 ± 1.5	60.9	48.8 ± 1.3	49.9	16.0 ± 1.7	18.3
160	939.9 ± 0.9	938.9 ± 1.3	934.4	51.8 ± 1.0	54.6	47.8 ± 1.0	46.5	14.6 ± 1.3	13.8
180	948.6 ± 0.5	948.2 ± 1.0	944.2	46.2 ± 2.5	49.6	43.7 ± 2.1	43.7	9.6 ± 2.8	10.0
200	958.5 ± 0.7	956.5 ± 0.9	953.2	43.1 ± 1.3	45.5	42.3 ± 1.1	41.3	7.6 ± 1.5	6.7

Table S277: Heat capacities at constant volume computed from MC simulations (c_V^{MC}), heat capacities at constant volume obtained from REFPROP[10] (c_V^{REFP}), heat capacities at constant pressure computed from MC simulations (c_P^{MC}), heat capacities at constant pressure obtained from REFPROP[10] (c_P^{REFP}), speed of sound computed from MC simulations (c^{MC}), speed of sound obtained from REFPROP[10] (c^{REFP}), viscosities computed from MD simulations (η^{MD}), and viscosities obtained from REFPROP[10] (η^{REFP}) of CO_2 rich ternary mixture with 3% impurity of H_2 and 1% impurity of CH_4 at 273 K and pressures ranging from 20 bar to 200 bar.

P/ [bar]	c_V^{MC}/ [J/mol K]	c_V^{REFP}/ [J/mol K]	c_P^{MC}/ [J/mol K]	c_P^{REFP}/ [J/mol K]	c^{MC}/ [m/s]	c^{REFP}/ [m/s]	η^{MD}/ [μPa s]	η^{REFP}/ [μPa s]
20	30.7	30.8	45.8 ± 0.1	46.8	245.1 ± 1.1	243.0	9.0 ± 4.6	13.9
40	36.6	–	78.0 ± 0.8	–	219.0 ± 1.4	–	86.6 ± 0.9	17.8
60	40.1 ± 0.2	–	118.3 ± 5.4	–	552.1 ± 23.2	–	90.3 ± 1.4	62.7
80	39.9 ± 0.1	39.9	107.7 ± 2.3	102.8	596.5 ± 12.3	552.2	98.7 ± 8.6	91.7
100	39.7 ± 0.2	39.7	101.9 ± 1.9	97.6	626.3 ± 16.6	585.1	100.9 ± 22.7	95.9
120	39.7 ± 0.2	39.6	99.1 ± 3.5	93.8	651.3 ± 17.5	613.8	102.7 ± 5.3	99.7
140	39.7 ± 0.1	39.6	94.8 ± 1.5	90.9	688.8 ± 10.9	639.4	108.5 ± 5.9	103.3
160	39.7 ± 0.2	39.5	94.3 ± 1.2	88.6	699.0 ± 8.3	662.7	113.6 ± 4.6	106.7
180	40.0 ± 0.4	39.5	90.4 ± 2.3	86.7	718.9 ± 21.7	684.2	112.8 ± 4.2	109.9
200	39.9 ± 0.1	39.5	90.0 ± 1.3	85.1	739.1 ± 12.4	704.2	124.3 ± 7.5	113.0

Table S278: Densities computed from MC and MD simulations (ρ^{MC} and ρ^{MD}), densities obtained from REFPROP[10] (ρ^{REFP}), isothermal compressibilities computed from MC simulations (β_T^{MC}), isothermal compressibilities obtained from REFPROP[10] (β_T^{REFP}), thermal expansion coefficients computed from MC simulations (α_P^{MC}), thermal expansion coefficients obtained from REFPROP[10] (α_P^{REFP}), Joule Thomson coefficients computed from MC simulations ($\mu_{\mathrm{JT}}^{\mathrm{MC}}$), and Joule Thomson coefficients obtained from REFPROP[10] ($\mu_{\mathrm{JT}}^{\mathrm{REFP}}$) of CO_2 rich ternary mixture with 3% impurity of H_2 and 1% impurity of CH_4 at 293 K and pressures ranging from 20 bar to 200 bar.

P/ [bar]	ρ^{MC}/ [kg/m^3]	ρ^{MD}/ [kg/m^3]	ρ^{REFP}/ [kg/m^3]	β_T^{MC}/ [10^{-5}/bar]	β_T^{REFP}/ [10^{-5}/bar]	α_P^{MC}/ [10^{-4}/K]	α_P^{REFP}/ [10^{-4}/K]	$\mu_{\mathrm{JT}}^{\mathrm{MC}}$/ [$10^{-3}$ K/bar]	$\mu_{\mathrm{JT}}^{\mathrm{REFP}}$/ [$10^{-3}$ K/bar]
20	38.4	39.0 ± 0.1	39.0	5579.3 ± 30.5	5646.9	47.6 ± 0.3	49.2	999.5 ± 20.0	1084.4
40	88.2	89.7 ± 0.2	91.3	3338.6 ± 42.9	3473.4	75.3 ± 0.8	82.2	1010.3 ± 19.7	1080.9
60	167.4 ± 0.5	174.8 ± 1.2	182.9	3558.6 ± 169.9	4062.7	186.2 ± 9.5	236.6	1006.6 ± 72.9	1015.2
80	712.8 ± 5.4	712.9 ± 4.4	713.5	715.1 ± 321.3	506.4	267.1 ± 91.8	195.3	182.4 ± 82.3	161.5
100	771.5 ± 3.5	764.9 ± 2.5	764.1	261.1 ± 45.4	245.9	128.6 ± 13.4	115.4	105.1 ± 15.9	102.6
120	798.7 ± 3.7	794.3 ± 1.6	795.4	165.1 ± 20.6	167.3	93.7 ± 9.5	88.3	75.1 ± 12.8	75.0
140	822.7 ± 2.0	818.4 ± 1.5	819.0	123.8 ± 5.2	128.2	76.3 ± 2.5	73.7	57.3 ± 3.6	57.7
160	841.8 ± 2.2	838.0 ± 0.8	838.1	100.2 ± 5.1	104.5	66.7 ± 2.9	64.3	45.6 ± 4.1	45.6
180	855.8 ± 2.5	853.3 ± 0.7	854.4	86.2 ± 5.8	88.5	60.2 ± 3.4	57.6	37.6 ± 5.0	36.4
200	871.1 ± 1.3	867.5 ± 0.8	868.6	73.1 ± 2.2	77.0	53.5 ± 1.7	52.6	29.0 ± 2.6	29.1

Table S279: Heat capacities at constant volume computed from MC simulations (c_V^{MC}), heat capacities at constant volume obtained from REFPROP[10] (c_V^{REFP}), heat capacities at constant pressure computed from MC simulations (c_P^{MC}), heat capacities at constant pressure obtained from REFPROP[10] (c_P^{REFP}), speed of sound computed from MC simulations (c^{MC}), speed of sound obtained from REFPROP[10] (c^{REFP}), viscosities computed from MD simulations (η^{MD}), and viscosities obtained from REFPROP[10] (η^{REFP}) of CO_2 rich ternary mixture with 3% impurity of H_2 and 1% impurity of CH_4 at 293 K and pressures ranging from 20 bar to 200 bar.

P/ [bar]	c_V^{MC}/ [J/mol K]	c_V^{REFP}/ [J/mol K]	c_P^{MC}/ [J/mol K]	c_P^{REFP}/ [J/mol K]	c^{MC}/ [m/s]	c^{REFP}/ [m/s]	η^{MD}/ [μPa s]	η^{REFP}/ [μPa s]
20	30.6	30.7	43.8 ± 0.1	44.3	258.3 ± 0.8	256.2	10.2 ± 2.8	14.9
40	33.5	34.1	57.5 ± 0.2	60.6	241.5 ± 1.6	236.7	15.4 ± 1.1	15.6
60	39.2 ± 0.2	41.9	112.3 ± 4.1	135.7	219.4 ± 6.6	208.7	17.0 ± 0.9	17.6
80	41.2 ± 0.6	42.6	223.1 ± 49.1	174.1	325.8 ± 81.5	336.1	60.6 ± 1.9	58.5
100	40.4 ± 0.2	40.8	145.1 ± 7.4	129.0	422.2 ± 38.3	410.3	65.6 ± 2.3	66.5
120	39.7 ± 0.3	40.1	123.5 ± 7.3	112.9	486.0 ± 33.6	460.2	76.1 ± 5.9	72.2
140	39.4 ± 0.3	39.7	111.4 ± 2.2	104.0	526.9 ± 12.4	499.5	78.7 ± 6.5	76.8
160	39.5 ± 0.1	39.5	105.6 ± 2.5	98.2	562.8 ± 16.0	532.7	84.7 ± 3.7	80.8
180	39.2 ± 0.1	39.4	100.7 ± 3.0	94.0	589.8 ± 21.7	561.8	100.2 ± 18.2	84.5
200	39.2 ± 0.2	39.3	95.3 ± 2.0	90.8	617.6 ± 11.4	587.9	94.1 ± 7.7	87.9

Table S280: Densities computed from MC and MD simulations (ρ^{MC} and ρ^{MD}), densities obtained from REFPROP[10] (ρ^{REFP}), isothermal compressibilities computed from MC simulations (β_T^{MC}), isothermal compressibilities obtained from REFPROP[10] (β_T^{REFP}), thermal expansion coefficients computed from MC simulations (α_P^{MC}), thermal expansion coefficients obtained from REFPROP[10] (α_P^{REFP}), Joule Thomson coefficients computed from MC simulations ($\mu_{\mathrm{JT}}^{\mathrm{MC}}$), and Joule Thomson coefficients obtained from REFPROP[10] ($\mu_{\mathrm{JT}}^{\mathrm{REFP}}$) of CO_2 rich ternary mixture with 3% impurity of H_2 and 1% impurity of CH_4 at 313 K and pressures ranging from 20 bar to 200 bar.

P/ [bar]	ρ^{MC}/ [kg/m^3]	ρ^{MD}/ [kg/m^3]	ρ^{REFP}/ [kg/m^3]	β_T^{MC}/ [10^{-5}/bar]	β_T^{REFP}/ [10^{-5}/bar]	α_P^{MC}/ [10^{-4}/K]	α_P^{REFP}/ [10^{-4}/K]	$\mu_{\mathrm{JT}}^{\mathrm{MC}}$/ [$10^{-3}$ K/bar]	$\mu_{\mathrm{JT}}^{\mathrm{REFP}}$/ [$10^{-3}$ K/bar]
20	35.2	35.7 ± 0.1	35.6	5411.9 ± 23.7	5481.3	41.3 ± 0.2	42.5	826.7 ± 17.4	914.9
40	77.4	78.7 ± 0.2	79.4	3052.5 ± 12.8	3117.7	57.3 ± 0.3	60.4	851.1 ± 9.2	905.8
60	131.5 ± 0.2	134.4 ± 0.6	137.8	2422.3 ± 41.0	2537.4	86.9 ± 1.6	96.6	833.0 ± 25.6	874.4
80	212.1 ± 0.5	219.3 ± 0.9	230.4	2474.0 ± 36.9	2742.1	167.0 ± 2.1	201.8	770.9 ± 15.0	–
100	375.0 ± 7.0	401.7 ± 7.3	433.3	3134.3 ± 252.4	3035.6	450.6 ± 48.5	499.8	535.4 ± 84.7	482.2
120	589.2 ± 6.8	577.2 ± 3.5	602.0	926.8 ± 127.5	807.4	258.1 ± 28.3	225.6	247.0 ± 36.5	235.6
140	659.5 ± 5.3	658.0 ± 1.9	671.7	437.9 ± 25.7	382.6	155.3 ± 5.6	135.1	162.4 ± 8.4	151.1
160	707.7 ± 2.6	705.0 ± 2.3	713.9	268.7 ± 21.1	246.3	113.2 ± 8.2	100.5	117.2 ± 13.4	109.7
180	741.3 ± 2.2	737.1 ± 0.8	744.6	174.4 ± 6.8	181.2	83.3 ± 3.0	82.2	82.8 ± 5.4	84.5
200	764.2 ± 1.9	760.9 ± 1.0	768.9	153.4 ± 10.4	143.3	79.1 ± 5.4	70.6	74.1 ± 9.2	67.2

Table S281: Heat capacities at constant volume computed from MC simulations (c_V^{MC}), heat capacities at constant volume obtained from REFPROP[10] (c_V^{REFP}), heat capacities at constant pressure computed from MC simulations (c_P^{MC}), heat capacities at constant pressure obtained from REFPROP[10] (c_P^{REFP}), speed of sound computed from MC simulations (c^{MC}), speed of sound obtained from REFPROP[10] (c^{REFP}), viscosities computed from MD simulations (η^{MD}), and viscosities obtained from REFPROP[10] (η^{REFP}) of CO_2 rich ternary mixture with 3% impurity of H_2 and 1% impurity of CH_4 at 313 K and pressures ranging from 20 bar to 200 bar.

P/ [bar]	c_V^{MC}/ [J/mol K]	c_V^{REFP}/ [J/mol K]	c_P^{MC}/ [J/mol K]	c_P^{REFP}/ [J/mol K]	c^{MC}/ [m/s]	c^{REFP}/ [m/s]	η^{MD}/ [μPa s]	η^{REFP}/ [μPa s]
20	31.0	30.9	42.9 ± 0.1	43.2	269.8 ± 0.6	267.7	7.8 ± 6.4	15.9
40	32.8	33.0	51.2 ± 0.1	52.6	257.3 ± 0.6	253.7	17.4 ± 1.9	16.5
60	35.1 ± 0.1	35.8	66.7 ± 0.6	71.3	244.1 ± 2.4	238.5	19.0 ± 3.0	17.5
80	38.9 ± 0.3	–	109.8 ± 1.3	–	232.0 ± 2.4	–	20.2 ± 2.7	20.2
100	43.3 ± 0.5	45.2	277.3 ± 29.4	297.7	233.5 ± 15.8	223.7	26.2 ± 4.0	30.5
120	41.5 ± 0.4	42.3	206.7 ± 16.2	181.4	302.2 ± 24.0	297.1	46.3 ± 10.4	45.2
140	40.2 ± 0.3	40.7	153.2 ± 3.7	135.1	363.3 ± 11.7	359.4	56.2 ± 11.6	53.5
160	39.6 ± 0.1	40.0	130.2 ± 6.9	116.4	415.8 ± 19.7	407.0	59.6 ± 6.2	59.2
180	39.3 ± 0.2	39.6	111.2 ± 3.0	106.1	467.6 ± 11.3	445.7	66.5 ± 5.3	63.9
200	39.4 ± 0.2	39.3	110.7 ± 5.2	99.4	489.7 ± 20.3	478.9	67.4 ± 3.1	67.9

S16.15 Data of thermodynamics and transport properties of CO_2 rich ternary mixture with 1 mole% impurity of N_2 and 3 mole% impurity of CH_4

Table S282: Densities computed from MC and MD simulations (ρ^{MC} and ρ^{MD}), densities obtained from REFPROP[10] (ρ^{REFP}), isothermal compressibilities computed from MC simulations (β_T^{MC}), isothermal compressibilities obtained from REFPROP[10] (β_T^{REFP}), thermal expansion coefficients computed from MC simulations (α_P^{MC}), thermal expansion coefficients obtained from REFPROP[10] (α_P^{REFP}), Joule Thomson coefficients computed from MC simulations (μ_{JT}^{MC}), and Joule Thomson coefficients obtained from REFPROP[10] (μ_{JT}^{REFP}) of CO_2 rich ternary mixture with 3% impurity of CH_4 and 1% impurity of N_2 at 253 K and pressures ranging from 20 bar to 200 bar.

P/ [bar]	ρ^{MC}/ [kg/m^3]	ρ^{MD}/ [kg/m^3]	ρ^{REFP}/ [kg/m^3]	β_T^{MC}/ [10^{-5}/bar]	β_T^{REFP}/ [10^{-5}/bar]	α_P^{MC}/ [10^{-4}/K]	α_P^{REFP}/ [10^{-4}/K]	μ_{JT}^{MC}/ [10^{-3} K/bar]	μ_{JT}^{REFP}/ [10^{-3} K/bar]
20	49.4 ± 0.1	50.4 ± 0.2	50.9	6292.3 ± 14.7	6565.7	74.9 ± 0.3	83.3	1516.4 ± 12.7	1673.8
40	988.9 ± 1.5	989.4 ± 0.5	989.7	43.5 ± 2.8	50.8	47.0 ± 2.7	49.6	8.5 ± 3.1	11.8
60	999.0 ± 1.2	999.4 ± 0.8	999.3	39.1 ± 1.9	45.8	44.2 ± 2.3	46.3	5.4 ± 2.6	8.1
80	1005.7 ± 1.2	1006.2 ± 0.7	1008.0	36.5 ± 0.8	41.8	42.2 ± 1.0	43.6	3.1 ± 1.2	4.9
100	1013.3 ± 1.0	1013.9 ± 1.1	1016.1	33.6 ± 2.0	38.5	40.1 ± 2.7	41.3	0.6 ± 3.1	2.1
120	1019.7 ± 1.3	1021.0 ± 0.5	1023.7	32.1 ± 1.7	35.8	38.8 ± 2.1	39.3	-0.8 ± 2.5	-0.3
140	1026.1 ± 1.0	1027.8 ± 1.1	1030.8	29.4 ± 0.6	33.5	36.2 ± 1.1	37.6	-4.0 ± 1.3	-2.3
160	1032.8 ± 0.5	1034.8 ± 0.7	1037.5	26.9 ± 0.3	31.5	33.9 ± 0.5	36.2	-7.0 ± 0.7	-4.2
180	1038.2 ± 0.7	1040.0 ± 0.6	1043.9	26.9 ± 0.8	29.7	34.3 ± 1.2	34.8	-6.3 ± 1.5	-5.9
200	1043.5 ± 0.8	1045.1 ± 0.6	1049.9	25.2 ± 0.7	28.1	32.9 ± 1.0	33.7	-8.1 ± 1.2	-7.4

Table S283: Heat capacities at constant volume computed from MC simulations (c_V^{MC}), heat capacities at constant volume obtained from REFPROP[10] (c_V^{REFP}), heat capacities at constant pressure computed from MC simulations (c_P^{MC}), heat capacities at constant pressure obtained from REFPROP[10] (c_P^{REFP}), speed of sound computed from MC simulations (c^{MC}), speed of sound obtained from REFPROP[10] (c^{REFP}), viscosities computed from MD simulations (η^{MD}), and viscosities obtained from REFPROP[10] (η^{REFP}) of CO_2 rich ternary mixture with 3% impurity of CH_4 and 1% impurity of N_2 at 253 K and pressures ranging from 20 bar to 200 bar.

P/ [bar]	c_V^{MC}/ [J/mol K]	c_V^{REFP}/ [J/mol K]	c_P^{MC}/ [J/mol K]	c_P^{REFP}/ [J/mol K]	c^{MC}/ [m/s]	c^{REFP}/ [m/s]	η^{MD}/ [μPa s]	η^{REFP}/ [μPa s]
20	31.7 ± 0.1	33.3	51.3 ± 0.2	55.9	228.1 ± 0.5	224.0	14.6 ± 2.2	12.9
40	40.3 ± 0.2	40.4	96.2 ± 2.9	93.7	745.4 ± 26.6	679.5	133.5 ± 4.7	127.6
60	40.3 ± 0.2	40.3	94.6 ± 3.0	91.3	775.3 ± 22.8	703.3	137.3 ± 4.3	131.7
80	40.4 ± 0.2	40.3	93.3 ± 1.9	89.3	793.2 ± 11.9	725.0	139.9 ± 2.0	135.5
100	40.3 ± 0.1	40.3	91.6 ± 3.9	87.7	817.2 ± 30.4	745.2	143.5 ± 2.8	139.1
120	40.2 ± 0.2	40.3	90.4 ± 3.2	86.2	828.5 ± 25.9	764.0	148.8 ± 5.9	142.7
140	40.5 ± 0.2	40.3	87.9 ± 2.1	85.0	848.8 ± 13.3	781.6	156.5 ± 10.5	146.1
160	40.5 ± 0.2	40.4	85.2 ± 0.8	84.0	870.0 ± 7.1	798.3	153.2 ± 7.4	149.4
180	40.6 ± 0.1	40.4	86.4 ± 2.0	83.0	872.6 ± 16.7	814.1	157.3 ± 6.4	152.7
200	40.5 ± 0.2	40.4	85.4 ± 1.5	82.2	896.1 ± 15.4	829.2	166.4 ± 7.8	155.9

Table S284: Densities computed from MC and MD simulations (ρ^{MC} and ρ^{MD}), densities obtained from REFPROP[10] (ρ^{REFP}), isothermal compressibilities computed from MC simulations (β_T^{MC}), isothermal compressibilities obtained from REFPROP[10] (β_T^{REFP}), thermal expansion coefficients computed from MC simulations (α_P^{MC}), thermal expansion coefficients obtained from REFPROP[10] (α_P^{REFP}), Joule Thomson coefficients computed from MC simulations ($\mu_{\mathrm{JT}}^{\mathrm{MC}}$), and Joule Thomson coefficients obtained from REFPROP[10] ($\mu_{\mathrm{JT}}^{\mathrm{REFP}}$) of CO_2 rich ternary mixture with 3% impurity of CH_4 and 1% impurity of N_2 at 273 K and pressures ranging from 20 bar to 200 bar.

P/ [bar]	ρ^{MC}/ [kg/m^3]	ρ^{MD}/ [kg/m^3]	ρ^{REFP}/ [kg/m^3]	β_T^{MC}/ [10^{-5}/bar]	β_T^{REFP}/ [10^{-5}/bar]	α_P^{MC}/ [10^{-4}/K]	α_P^{REFP}/ [10^{-4}/K]	$\mu_{\mathrm{JT}}^{\mathrm{MC}}$/ [$10^{-3}$ K/bar]	$\mu_{\mathrm{JT}}^{\mathrm{REFP}}$/ [$10^{-3}$ K/bar]
20	43.4	44.3	44.2	5843.7 ± 54.7	5961.0	57.6 ± 0.6	60.9	1228.2 ± 36.7	1346.3
40	110.2 ± 0.2	873.9 ± 0.8	290.0	4514.5 ± 60.4	-1644.4	142.0 ± 1.6	-398.6	1319.9 ± 21.2	–
60	897.8 ± 2.3	892.0 ± 2.4	890.5	78.1 ± 4.7	108.1	62.2 ± 3.7	74.6	31.9 ± 4.7	46.1
80	911.3 ± 2.1	907.3 ± 0.8	907.9	69.4 ± 5.5	87.4	57.5 ± 3.8	64.5	26.5 ± 5.0	35.3
100	923.5 ± 2.1	920.9 ± 0.9	922.6	61.3 ± 4.0	73.9	53.2 ± 3.5	57.6	21.4 ± 4.6	27.4
120	932.2 ± 1.3	932.1 ± 0.5	935.4	55.2 ± 1.0	64.4	49.4 ± 1.1	52.5	16.8 ± 1.4	21.2
140	943.8 ± 1.1	943.1 ± 0.9	946.8	49.0 ± 1.2	57.2	45.7 ± 1.2	48.5	12.2 ± 1.7	16.2
160	952.9 ± 1.2	953.3 ± 0.8	957.1	47.0 ± 1.4	51.6	45.5 ± 1.4	45.3	11.6 ± 1.9	12.0
180	960.2 ± 1.3	962.3 ± 1.0	966.6	42.6 ± 1.9	47.1	42.2 ± 1.9	42.7	7.5 ± 2.6	8.5
200	969.3 ± 1.1	970.6 ± 0.9	975.4	40.3 ± 2.0	43.4	40.9 ± 2.1	40.5	5.7 ± 2.8	5.5

Table S285: Heat capacities at constant volume computed from MC simulations (c_V^{MC}), heat capacities at constant volume obtained from REFPROP[10] (c_V^{REFP}), heat capacities at constant pressure computed from MC simulations (c_P^{MC}), heat capacities at constant pressure obtained from REFPROP[10] (c_P^{REFP}), speed of sound computed from MC simulations (c^{MC}), speed of sound obtained from REFPROP[10] (c^{REFP}), viscosities computed from MD simulations (η^{MD}), and viscosities obtained from REFPROP[10] (η^{REFP}) of CO_2 rich ternary mixture with 3% impurity of CH_4 and 1% impurity of N_2 at 273 K and pressures ranging from 20 bar to 200 bar.

P/ [bar]	c_V^{MC}/ [J/mol K]	c_V^{REFP}/ [J/mol K]	c_P^{MC}/ [J/mol K]	c_P^{REFP}/ [J/mol K]	c^{MC}/ [m/s]	c^{REFP}/ [m/s]	η^{MD}/ [μPa s]	η^{REFP}/ [μPa s]
20	30.8	31.3	46.1 ± 0.2	47.8	243.0 ± 1.3	240.6	10.2 ± 5.5	13.9
40	37.2 ± 0.2	–	85.0 ± 0.4	–	214.3 ± 1.7	–	93.5 ± 4.0	20.1
60	39.7 ± 0.1	40.6	104.9 ± 4.2	108.6	614.1 ± 22.3	527.0	97.6 ± 2.0	93.5
80	39.6 ± 0.1	40.3	101.5 ± 3.6	101.8	636.0 ± 27.8	564.1	107.3 ± 6.7	98.3
100	39.6 ± 0.2	40.2	98.6 ± 4.2	97.2	663.1 ± 25.7	595.6	106.6 ± 8.7	102.5
120	39.7 ± 0.2	40.0	95.5 ± 1.5	93.7	684.2 ± 8.1	623.2	110.6 ± 5.0	106.5
140	39.7 ± 0.1	40.0	92.9 ± 1.8	91.0	711.3 ± 11.1	648.0	111.4 ± 1.6	110.1
160	39.7 ± 0.1	40.0	93.9 ± 2.1	88.8	726.5 ± 13.3	670.7	118.3 ± 4.2	113.6
180	39.8 ± 0.1	39.9	91.0 ± 2.9	87.0	747.8 ± 20.2	691.7	113.7 ± 7.1	116.9
200	39.9 ± 0.2	39.9	90.3 ± 2.9	85.5	761.1 ± 22.5	711.2	125.8 ± 6.1	120.1

Table S286: Densities computed from MC and MD simulations (ρ^{MC} and ρ^{MD}), densities obtained from REFPROP[10] (ρ^{REFP}), isothermal compressibilities computed from MC simulations (β_T^{MC}), isothermal compressibilities obtained from REFPROP[10] (β_T^{REFP}), thermal expansion coefficients computed from MC simulations (α_P^{MC}), thermal expansion coefficients obtained from REFPROP[10] (α_P^{REFP}), Joule Thomson coefficients computed from MC simulations (μ_{JT}^{MC}), and Joule Thomson coefficients obtained from REFPROP[10] (μ_{JT}^{REFP}) of CO_2 rich ternary mixture with 3% impurity of CH_4 and 1% impurity of N_2 at 293 K and pressures ranging from 20 bar to 200 bar.

P/ [bar]	ρ^{MC}/ [kg/m^3]	ρ^{MD}/ [kg/m^3]	ρ^{REFP}/ [kg/m^3]	β_T^{MC}/ [10^{-5}/bar]	β_T^{REFP}/ [10^{-5}/bar]	α_P^{MC}/ [10^{-4}/K]	α_P^{REFP}/ [10^{-4}/K]	μ_{JT}^{MC}/ [10^{-3} K/bar]	μ_{JT}^{REFP}/ [10^{-3} K/bar]
20	39.1	39.8 ± 0.1	39.7	5588.4 ± 35.4	5670.9	47.9 ± 0.3	49.9	1009.5 ± 23.8	1112.8
40	90.4 ± 0.1	92.3 ± 0.4	93.5	3416.9 ± 32.3	3537.9	78.7 ± 0.9	85.8	1051.5 ± 22.5	1108.1
60	177.9 ± 1.8	230.0 ± 44.4	195.3	4088.2 ± 208.3	4795.6	231.6 ± 16.5	307.0	1044.5 ± 107.2	1024.2
80	768.3 ± 2.0	754.6 ± 4.3	748.4	268.8 ± 18.0	388.2	134.6 ± 7.3	162.7	108.4 ± 8.7	137.4
100	802.2 ± 1.0	795.0 ± 2.7	791.9	160.6 ± 7.3	213.8	92.2 ± 3.2	106.0	73.8 ± 4.3	91.6
120	824.6 ± 1.2	818.1 ± 1.9	820.7	122.1 ± 6.0	151.2	76.0 ± 3.1	83.5	57.0 ± 4.5	68.1
140	843.8 ± 1.1	839.0 ± 1.6	842.9	104.4 ± 6.2	118.2	69.5 ± 3.8	70.7	48.6 ± 5.4	52.9
160	859.6 ± 1.8	857.1 ± 1.5	861.1	87.0 ± 3.8	97.5	61.3 ± 2.8	62.2	38.7 ± 4.1	41.9
180	872.9 ± 1.2	871.8 ± 0.5	876.8	71.5 ± 2.9	83.3	52.9 ± 2.2	56.1	28.3 ± 3.4	33.5
200	885.1 ± 1.1	885.1 ± 0.8	890.5	65.4 ± 0.7	72.9	50.4 ± 0.7	51.4	24.4 ± 1.2	26.8

Table S287: Heat capacities at constant volume computed from MC simulations (c_V^{MC}), heat capacities at constant volume obtained from REFPROP[10] (c_V^{REFP}), heat capacities at constant pressure computed from MC simulations (c_P^{MC}), heat capacities at constant pressure obtained from REFPROP[10] (c_P^{REFP}), speed of sound computed from MC simulations (c^{MC}), speed of sound obtained from REFPROP[10] (c^{REFP}), viscosities computed from MD simulations (η^{MD}), and viscosities obtained from REFPROP[10] (η^{REFP}) of CO_2 rich ternary mixture with 3% impurity of CH_4 and 1% impurity of N_2 at 293 K and pressures ranging from 20 bar to 200 bar.

P/ [bar]	c_V^{MC}/ [J/mol K]	c_V^{REFP}/ [J/mol K]	c_P^{MC}/ [J/mol K]	c_P^{REFP}/ [J/mol K]	c^{MC}/ [m/s]	c^{REFP}/ [m/s]	η^{MD}/ [μPa s]	η^{REFP}/ [μPa s]
20	30.8	31.0	44.0 ± 0.1	44.9	255.9 ± 0.9	253.9	8.2 ± 6.7	14.9
40	33.7	34.8	59.1 ± 0.4	62.8	238.0 ± 1.3	233.6	16.8 ± 2.1	15.6
60	40.1 ± 0.2	45.1	133.9 ± 7.9	171.9	214.2 ± 8.4	201.7	19.5 ± 1.9	18.0
80	39.9 ± 0.2	42.7	152.0 ± 5.3	157.5	429.3 ± 16.2	356.2	70.4 ± 8.3	64.2
100	39.5 ± 0.1	41.2	123.4 ± 2.3	124.9	492.4 ± 12.1	423.1	74.2 ± 3.1	71.7
120	39.4 ± 0.2	40.5	112.2 ± 2.9	111.3	531.9 ± 14.8	470.3	80.2 ± 2.6	77.3
140	39.3 ± 0.1	40.2	108.6 ± 3.5	103.3	560.4 ± 19.0	508.1	87.4 ± 4.6	81.9
160	39.4 ± 0.1	40.0	102.9 ± 3.3	98.0	591.3 ± 15.9	540.2	85.9 ± 3.6	86.0
180	39.2 ± 0.2	39.8	95.9 ± 2.4	94.1	625.9 ± 15.1	568.6	95.2 ± 4.9	89.7
200	39.3 ± 0.1	39.7	94.9 ± 1.4	91.0	645.4 ± 6.0	594.1	102.9 ± 9.0	93.2

Table S288: Densities computed from MC and MD simulations (ρ^{MC} and ρ^{MD}), densities obtained from REFPROP[10] (ρ^{REFP}), isothermal compressibilities computed from MC simulations (β_T^{MC}), isothermal compressibilities obtained from REFPROP[10] (β_T^{REFP}), thermal expansion coefficients computed from MC simulations (α_P^{MC}), thermal expansion coefficients obtained from REFPROP[10] (α_P^{REFP}), Joule Thomson coefficients computed from MC simulations ($\mu_{\mathrm{JT}}^{\mathrm{MC}}$), and Joule Thomson coefficients obtained from REFPROP[10] ($\mu_{\mathrm{JT}}^{\mathrm{REFP}}$) of CO_2 rich ternary mixture with 3% impurity of CH_4 and 1% impurity of N_2 at 313 K and pressures ranging from 20 bar to 200 bar.

P/ [bar]	ρ^{MC}/ [kg/m^3]	ρ^{MD}/ [kg/m^3]	ρ^{REFP}/ [kg/m^3]	β_T^{MC}/ [10^{-5}/bar]	β_T^{REFP}/ [10^{-5}/bar]	α_P^{MC}/ [10^{-4}/K]	α_P^{REFP}/ [10^{-4}/K]	$\mu_{\mathrm{JT}}^{\mathrm{MC}}$/ [$10^{-3}$ K/bar]	$\mu_{\mathrm{JT}}^{\mathrm{REFP}}$/ [$10^{-3}$ K/bar]
20	35.7	36.4 ± 0.1	36.2	5441.5 ± 15.4	5496.8	41.8 ± 0.1	42.9	857.7 ± 9.3	936.8
40	79.0	80.6 ± 0.2	81.0	3058.5 ± 29.4	3146.2	58.1 ± 0.6	61.9	862.2 ± 20.8	927.0
60	135.9 ± 0.1	139.3 ± 0.4	141.7	2498.0 ± 61.5	2604.5	92.5 ± 2.3	102.1	863.9 ± 34.3	892.6
80	226.6 ± 0.7	236.3 ± 2.1	243.9	2805.1 ± 124.6	3006.7	204.7 ± 10.0	237.3	795.9 ± 55.9	–
100	482.8 ± 4.0	458.9 ± 9.0	486.5	3667.1 ± 540.0	2703.8	718.2 ± 103.4	512.9	428.3 ± 84.7	421.0
120	640.2 ± 2.8	624.3 ± 6.7	636.0	608.4 ± 49.5	670.2	200.0 ± 14.4	202.1	195.3 ± 19.4	209.6
140	705.0 ± 3.9	690.2 ± 2.3	698.6	296.6 ± 24.2	339.2	123.0 ± 8.3	126.4	125.9 ± 12.7	137.8
160	739.9 ± 2.4	731.7 ± 1.0	738.1	203.2 ± 14.9	225.3	95.0 ± 4.9	96.1	94.7 ± 7.8	101.5
180	764.5 ± 2.2	758.7 ± 1.7	767.3	157.0 ± 7.1	168.6	80.7 ± 3.3	79.5	76.2 ± 5.5	78.8
200	786.7 ± 1.8	782.4 ± 1.1	790.7	120.7 ± 4.1	134.8	67.1 ± 2.1	68.7	58.3 ± 3.6	62.9

Table S289: Heat capacities at constant volume computed from MC simulations (c_V^{MC}), heat capacities at constant volume obtained from REFPROP[10] (c_V^{REFP}), heat capacities at constant pressure computed from MC simulations (c_P^{MC}), heat capacities at constant pressure obtained from REFPROP[10] (c_P^{REFP}), speed of sound computed from MC simulations (c^{MC}), speed of sound obtained from REFPROP[10] (c^{REFP}), viscosities computed from MD simulations (η^{MD}), and viscosities obtained from REFPROP[10] (η^{REFP}) of CO_2 rich ternary mixture with 3% impurity of CH_4 and 1% impurity of N_2 at 313 K and pressures ranging from 20 bar to 200 bar.

P/ [bar]	c_V^{MC}/ [J/mol K]	c_V^{REFP}/ [J/mol K]	c_P^{MC}/ [J/mol K]	c_P^{REFP}/ [J/mol K]	c^{MC}/ [m/s]	c^{REFP}/ [m/s]	η^{MD}/ [μPa s]	η^{REFP}/ [μPa s]
20	31.1	31.2	43.2	43.7	267.2 ± 0.4	265.5	5.6 ± 4.3	15.9
40	32.9	33.5	51.8 ± 0.2	53.7	255.1 ± 1.4	251.0	16.0 ± 1.7	16.5
60	35.4 ± 0.1	36.6	69.5 ± 0.8	74.7	240.4 ± 3.3	235.0	16.8 ± 1.0	17.6
80	39.5 ± 0.4	–	128.9 ± 5.1	–	226.6 ± 6.8	–	19.9 ± 0.9	20.7
100	42.4 ± 0.5	46.9	447.2 ± 57.2	316.2	244.1 ± 23.9	226.4	32.1 ± 3.8	34.7
120	40.6 ± 0.2	42.9	180.9 ± 9.1	171.9	338.1 ± 16.2	306.7	48.9 ± 3.9	49.3
140	39.8 ± 0.2	41.3	138.1 ± 5.9	132.0	407.6 ± 18.8	367.4	60.4 ± 5.9	57.3
160	39.3 ± 0.1	40.5	121.0 ± 3.0	115.3	452.5 ± 17.5	413.6	61.1 ± 1.5	63.1
180	39.0 ± 0.1	40.1	112.8 ± 3.0	105.8	490.8 ± 12.9	451.5	69.8 ± 3.4	67.8
200	39.0 ± 0.2	39.9	103.3 ± 2.0	99.5	527.9 ± 10.4	484.0	70.9 ± 3.5	71.9

S16.16 Data of thermodynamics and transport properties of CO_2 rich ternary mixture with 3 mole% impurity of N_2 and 1 mole% impurity of CH_4

Table S290: Densities computed from MC and MD simulations (ρ^{MC} and ρ^{MD}), densities obtained from REFPROP[10] (ρ^{REFP}), isothermal compressibilities computed from MC simulations (β_T^{MC}), isothermal compressibilities obtained from REFPROP[10] (β_T^{REFP}), thermal expansion coefficients computed from MC simulations (α_P^{MC}), thermal expansion coefficients obtained from REFPROP[10] (α_P^{REFP}), Joule Thomson coefficients computed from MC simulations ($\mu_{\mathrm{JT}}^{\mathrm{MC}}$), and Joule Thomson coefficients obtained from REFPROP[10] ($\mu_{\mathrm{JT}}^{\mathrm{REFP}}$) of CO_2 rich ternary mixture with 3% impurity of N_2 and 1% impurity of CH_4 at 253 K and pressures ranging from 20 bar to 200 bar.

P/ [bar]	ρ^{MC}/ [kg/m^3]	ρ^{MD}/ [kg/m^3]	ρ^{REFP}/ [kg/m^3]	β_T^{MC}/ [10^{-5}/bar]	β_T^{REFP}/ [10^{-5}/bar]	α_P^{MC}/ [10^{-4}/K]	α_P^{REFP}/ [10^{-4}/K]	$\mu_{\mathrm{JT}}^{\mathrm{MC}}$/ [$10^{-3}$ K/bar]	$\mu_{\mathrm{JT}}^{\mathrm{REFP}}$/ [$10^{-3}$ K/bar]
20	49.6	50.4 ± 0.2	51.1	6263.7 ± 35.7	6537.1	74.1 ± 0.6	82.5	1499.3 ± 27.1	1663.9
40	992.5 ± 1.0	989.4 ± 0.5	993.7	44.8 ± 0.6	52.9	48.1 ± 1.0	50.9	9.7 ± 1.1	13.2
60	1000.6 ± 0.9	999.4 ± 0.8	1003.7	42.5 ± 2.2	47.5	46.9 ± 2.0	47.3	8.3 ± 2.3	9.2
80	1008.3 ± 1.4	1006.2 ± 0.7	1012.9	37.4 ± 1.3	43.2	42.3 ± 1.7	44.4	3.2 ± 2.0	5.9
100	1015.4 ± 1.4	1013.9 ± 1.1	1021.3	35.4 ± 0.9	39.7	40.8 ± 0.8	42.0	1.5 ± 0.9	3.0
120	1022.2 ± 1.0	1021.0 ± 0.5	1029.1	32.8 ± 0.9	36.8	39.4 ± 1.6	39.9	-0.1 ± 1.8	0.5
140	1029.4 ± 0.5	1027.8 ± 1.1	1036.5	31.5 ± 1.2	34.3	38.2 ± 1.5	38.2	-1.5 ± 1.8	-1.7
160	1034.8 ± 1.1	1034.8 ± 0.7	1043.4	29.6 ± 0.8	32.2	36.7 ± 1.1	36.6	-3.4 ± 1.3	-3.6
180	1041.1 ± 1.1	1040.0 ± 0.6	1049.9	27.9 ± 0.5	30.4	35.0 ± 0.5	35.3	-5.5 ± 0.7	-5.3
200	1046.9 ± 0.9	1045.1 ± 0.6	1056.2	26.5 ± 0.9	28.8	34.0 ± 1.3	34.1	-6.8 ± 1.6	-6.9

Table S291: Heat capacities at constant volume computed from MC simulations (c_V^{MC}), heat capacities at constant volume obtained from REFPROP[10] (c_V^{REFP}), heat capacities at constant pressure computed from MC simulations (c_P^{MC}), heat capacities at constant pressure obtained from REFPROP[10] (c_P^{REFP}), speed of sound computed from MC simulations (c^{MC}), speed of sound obtained from REFPROP[10] (c^{REFP}), viscosities computed from MD simulations (η^{MD}), and viscosities obtained from REFPROP[10] (η^{REFP}) of CO_2 rich ternary mixture with 3% impurity of N_2 and 1% impurity of CH_4 at 253 K and pressures ranging from 20 bar to 200 bar.

P/ [bar]	c_V^{MC}/ [J/mol K]	c_V^{REFP}/ [J/mol K]	c_P^{MC}/ [J/mol K]	c_P^{REFP}/ [J/mol K]	c^{MC}/ [m/s]	c^{REFP}/ [m/s]	η^{MD}/ [μPa s]	η^{REFP}/ [μPa s]
20	31.6 ± 0.1	33.0	50.9 ± 0.3	55.3	227.8 ± 0.9	223.9	14.6 ± 2.2	13.0
40	40.1 ± 0.2	40.5	97.4 ± 1.5	94.3	739.5 ± 7.8	665.2	133.5 ± 4.7	127.1
60	40.0 ± 0.2	40.5	96.9 ± 2.3	91.8	754.6 ± 21.2	689.6	137.3 ± 4.3	131.2
80	40.1 ± 0.1	40.4	92.2 ± 2.6	89.7	780.5 ± 17.5	711.9	139.9 ± 2.0	135.1
100	40.2 ± 0.1	40.4	91.0 ± 0.7	88.0	793.6 ± 10.8	732.5	143.5 ± 2.8	138.9
120	40.4 ± 0.3	40.4	91.2 ± 2.7	86.5	821.0 ± 17.0	751.6	148.8 ± 5.9	142.4
140	40.6 ± 0.2	40.4	89.7 ± 2.3	85.2	825.6 ± 18.8	769.6	156.5 ± 10.5	145.9
160	40.4 ± 0.2	40.5	88.5 ± 1.8	84.1	845.6 ± 14.9	786.5	153.2 ± 7.4	149.3
180	40.5 ± 0.3	40.5	86.5 ± 0.6	83.1	857.8 ± 9.6	802.6	157.3 ± 6.4	152.6
200	40.5 ± 0.2	40.5	85.9 ± 2.1	82.3	873.8 ± 18.6	817.9	166.4 ± 7.8	155.8

Table S292: Densities computed from MC and MD simulations (ρ^{MC} and ρ^{MD}), densities obtained from REFPROP[10] (ρ^{REFP}), isothermal compressibilities computed from MC simulations (β_T^{MC}), isothermal compressibilities obtained from REFPROP[10] (β_T^{REFP}), thermal expansion coefficients computed from MC simulations (α_P^{MC}), thermal expansion coefficients obtained from REFPROP[10] (α_P^{REFP}), Joule Thomson coefficients computed from MC simulations (μ_{JT}^{MC}), and Joule Thomson coefficients obtained from REFPROP[10] (μ_{JT}^{REFP}) of CO_2 rich ternary mixture with 3% impurity of N_2 and 1% impurity of CH_4 at 273 K and pressures ranging from 20 bar to 200 bar.

P/ [bar]	ρ^{MC}/ [kg/m^3]	ρ^{MD}/ [kg/m^3]	ρ^{REFP}/ [kg/m^3]	β_T^{MC}/ [10^{-5}/bar]	β_T^{REFP}/ [10^{-5}/bar]	α_P^{MC}/ [10^{-4}/K]	α_P^{REFP}/ [10^{-4}/K]	μ_{JT}^{MC}/ [10^{-3} K/bar]	μ_{JT}^{REFP}/ [10^{-3} K/bar]
20	43.6	44.3	44.4	5848.8 ± 56.2	5949.1	57.4 ± 0.6	60.6	1224.1 ± 36.3	1341.2
40	110.0 ± 0.1	873.9 ± 0.8	254.1	4371.1 ± 48.3	−3096.9	135.7 ± 1.0	−482.1	1291.7 ± 15.7	–
60	893.7 ± 1.5	892.0 ± 2.4	891.1	94.5 ± 2.1	116.2	72.3 ± 1.7	78.2	41.6 ± 2.1	49.9
80	910.7 ± 1.5	907.3 ± 0.8	909.7	75.9 ± 3.1	92.5	61.8 ± 2.4	66.8	31.0 ± 3.0	38.0
100	922.3 ± 1.8	920.9 ± 0.9	925.2	67.7 ± 2.3	77.6	56.9 ± 2.0	59.3	25.6 ± 2.6	29.5
120	934.4 ± 1.6	932.1 ± 0.5	938.6	57.9 ± 2.2	67.1	50.9 ± 1.9	53.8	18.7 ± 2.5	22.9
140	946.0 ± 0.7	943.1 ± 0.9	950.5	52.1 ± 1.4	59.4	47.9 ± 1.5	49.6	14.8 ± 2.0	17.6
160	954.6 ± 0.8	953.3 ± 0.8	961.3	47.0 ± 2.2	53.3	44.4 ± 1.9	46.2	10.4 ± 2.6	13.2
180	963.2 ± 1.5	962.3 ± 1.0	971.1	43.4 ± 1.0	48.5	42.4 ± 0.8	43.5	7.8 ± 1.1	9.5
200	972.0 ± 1.0	970.6 ± 0.9	980.2	41.7 ± 1.9	44.6	41.8 ± 2.5	41.2	6.9 ± 3.3	6.4

Table S293: Heat capacities at constant volume computed from MC simulations (c_V^{MC}), heat capacities at constant volume obtained from REFPROP[10] (c_V^{REFP}), heat capacities at constant pressure computed from MC simulations (c_P^{MC}), heat capacities at constant pressure obtained from REFPROP[10] (c_P^{REFP}), speed of sound computed from MC simulations (c^{MC}), speed of sound obtained from REFPROP[10] (c^{REFP}), viscosities computed from MD simulations (η^{MD}), and viscosities obtained from REFPROP[10] (η^{REFP}) of CO_2 rich ternary mixture with 3% impurity of N_2 and 1% impurity of CH_4 at 273 K and pressures ranging from 20 bar to 200 bar.

P/ [bar]	c_V^{MC}/ [J/mol K]	c_V^{REFP}/ [J/mol K]	c_P^{MC}/ [J/mol K]	c_P^{REFP}/ [J/mol K]	c^{MC}/ [m/s]	c^{REFP}/ [m/s]	η^{MD}/ [μPa s]	η^{REFP}/ [μPa s]
20	30.7	31.1	45.9 ± 0.2	47.6	242.4 ± 1.3	240.3	10.2 ± 5.5	14.0
40	36.9 ± 0.2	–	82.3 ± 0.6	–	215.5 ± 1.5	–	93.5 ± 4.0	18.9
60	39.8 ± 0.1	40.8	113.3 ± 1.7	110.6	580.5 ± 7.7	511.6	97.6 ± 2.0	92.6
80	39.6 ± 0.3	40.5	105.2 ± 2.8	103.1	619.9 ± 15.3	550.2	107.3 ± 6.7	97.6
100	39.6 ± 0.2	40.3	101.3 ± 2.3	98.1	640.1 ± 12.9	582.7	106.6 ± 8.7	102.0
120	39.6 ± 0.2	40.1	96.3 ± 1.9	94.4	670.4 ± 14.5	611.0	110.6 ± 5.0	106.0
140	39.7 ± 0.1	40.0	94.8 ± 2.1	91.5	696.3 ± 12.2	636.4	111.4 ± 1.6	109.7
160	39.6 ± 0.2	40.0	91.7 ± 2.2	89.2	719.4 ± 19.1	659.5	118.3 ± 4.2	113.3
180	39.7 ± 0.1	40.0	90.6 ± 0.9	87.3	738.9 ± 9.0	680.8	113.7 ± 7.1	116.7
200	39.8 ± 0.2	40.0	90.6 ± 3.8	85.7	749.7 ± 23.4	700.7	125.8 ± 6.1	119.9

Table S294: Densities computed from MC and MD simulations (ρ^{MC} and ρ^{MD}), densities obtained from REFPROP[10] (ρ^{REFP}), isothermal compressibilities computed from MC simulations (β_T^{MC}), isothermal compressibilities obtained from REFPROP[10] (β_T^{REFP}), thermal expansion coefficients computed from MC simulations (α_P^{MC}), thermal expansion coefficients obtained from REFPROP[10] (α_P^{REFP}), Joule Thomson coefficients computed from MC simulations ($\mu_{\mathrm{JT}}^{\mathrm{MC}}$), and Joule Thomson coefficients obtained from REFPROP[10] ($\mu_{\mathrm{JT}}^{\mathrm{REFP}}$) of CO_2 rich ternary mixture with 3% impurity of N_2 and 1% impurity of CH_4 at 293 K and pressures ranging from 20 bar to 200 bar.

P/ [bar]	ρ^{MC}/ [kg/m^3]	ρ^{MD}/ [kg/m^3]	ρ^{REFP}/ [kg/m^3]	β_T^{MC}/ [10^{-5}/bar]	β_T^{REFP}/ [10^{-5}/bar]	α_P^{MC}/ [10^{-4}/K]	α_P^{REFP}/ [10^{-4}/K]	$\mu_{\mathrm{JT}}^{\mathrm{MC}}$/ [$10^{-3}$ K/bar]	$\mu_{\mathrm{JT}}^{\mathrm{REFP}}$/ [$10^{-3}$ K/bar]
20	39.2	39.8 ± 0.1	39.8	5596.6 ± 49.3	5663.6	48.0 ± 0.5	49.8	1017.2 ± 34.0	1110.1
40	90.5 ± 0.1	92.3 ± 0.4	93.7	3386.7 ± 62.2	3516.4	77.6 ± 1.4	84.8	1040.5 ± 34.2	1101.2
60	176.2 ± 0.6	230.0 ± 44.4	192.9	3763.3 ± 144.4	4518.9	205.7 ± 7.6	281.2	1018.8 ± 51.4	1020.9
80	754.0 ± 8.1	754.6 ± 4.3	739.2	339.9 ± 56.7	467.4	156.5 ± 19.1	185.2	125.4 ± 21.5	153.2
100	793.3 ± 2.4	795.0 ± 2.7	788.6	184.2 ± 11.5	236.0	100.2 ± 5.6	113.1	82.7 ± 7.7	98.7
120	818.5 ± 2.5	818.1 ± 1.9	819.8	136.4 ± 6.4	162.3	81.5 ± 2.7	87.2	63.5 ± 3.8	72.6
140	842.0 ± 0.9	839.0 ± 1.6	843.4	109.3 ± 1.7	125.0	71.1 ± 1.0	73.1	50.9 ± 1.5	56.1
160	859.9 ± 2.8	857.1 ± 1.5	862.6	88.4 ± 4.7	102.2	61.2 ± 2.8	64.0	39.1 ± 4.2	44.4
180	873.7 ± 1.2	871.8 ± 0.5	879.0	73.1 ± 2.5	86.8	53.3 ± 1.6	57.4	29.0 ± 2.4	35.5
200	885.4 ± 1.3	885.1 ± 0.8	893.4	68.1 ± 3.2	75.5	51.2 ± 2.0	52.5	25.8 ± 3.0	28.5

Table S295: Heat capacities at constant volume computed from MC simulations (c_V^{MC}), heat capacities at constant volume obtained from REFPROP[10] (c_V^{REFP}), heat capacities at constant pressure computed from MC simulations (c_P^{MC}), heat capacities at constant pressure obtained from REFPROP[10] (c_P^{REFP}), speed of sound computed from MC simulations (c^{MC}), speed of sound obtained from REFPROP[10] (c^{REFP}), viscosities computed from MD simulations (η^{MD}), and viscosities obtained from REFPROP[10] (η^{REFP}) of CO_2 rich ternary mixture with 3% impurity of N_2 and 1% impurity of CH_4 at 293 K and pressures ranging from 20 bar to 200 bar.

P/ [bar]	c_V^{MC}/ [J/mol K]	c_V^{REFP}/ [J/mol K]	c_P^{MC}/ [J/mol K]	c_P^{REFP}/ [J/mol K]	c^{MC}/ [m/s]	c^{REFP}/ [m/s]	η^{MD}/ [μPa s]	η^{REFP}/ [μPa s]
20	30.7	30.9	43.9 ± 0.1	44.8	255.5 ± 1.2	253.5	8.2 ± 6.7	15.0
40	33.6	34.6	58.4 ± 0.4	62.3	238.3 ± 2.4	233.7	16.8 ± 2.1	15.7
60	39.7 ± 0.4	44.0	121.2 ± 2.9	159.0	214.5 ± 4.9	203.5	19.5 ± 1.9	18.0
80	40.5 ± 0.2	43.3	164.1 ± 11.7	169.1	397.9 ± 36.2	336.2	70.4 ± 8.3	62.3
100	39.7 ± 0.4	41.4	127.7 ± 4.8	128.5	469.1 ± 17.2	408.2	74.2 ± 3.1	70.5
120	39.4 ± 0.3	40.7	115.5 ± 1.8	113.2	512.7 ± 12.7	457.3	80.2 ± 2.6	76.4
140	39.2 ± 0.2	40.3	109.5 ± 1.2	104.5	551.0 ± 5.3	496.3	87.4 ± 4.6	81.2
160	39.2 ± 0.1	40.0	102.0 ± 2.8	98.8	584.9 ± 17.5	529.2	85.9 ± 3.6	85.5
180	39.3 ± 0.3	39.9	95.7 ± 1.7	94.7	617.3 ± 12.1	558.1	95.2 ± 4.9	89.3
200	39.2 ± 0.1	39.8	94.5 ± 1.7	91.5	632.1 ± 16.0	584.0	102.9 ± 9.0	92.8

Table S296: Densities computed from MC and MD simulations (ρ^{MC} and ρ^{MD}), densities obtained from REFPROP[10] (ρ^{REFP}), isothermal compressibilities computed from MC simulations (β_T^{MC}), isothermal compressibilities obtained from REFPROP[10] (β_T^{REFP}), thermal expansion coefficients computed from MC simulations (α_P^{MC}), thermal expansion coefficients obtained from REFPROP[10] (α_P^{REFP}), Joule Thomson coefficients computed from MC simulations ($\mu_{\mathrm{JT}}^{\mathrm{MC}}$), and Joule Thomson coefficients obtained from REFPROP[10] ($\mu_{\mathrm{JT}}^{\mathrm{REFP}}$) of CO_2 rich ternary mixture with 3% impurity of N_2 and 1% impurity of CH_4 at 313 K and pressures ranging from 20 bar to 200 bar.

P/ [bar]	ρ^{MC}/ [kg/m^3]	ρ^{MD}/ [kg/m^3]	ρ^{REFP}/ [kg/m^3]	β_T^{MC}/ [10^{-5}/bar]	β_T^{REFP}/ [10^{-5}/bar]	α_P^{MC}/ [10^{-4}/K]	α_P^{REFP}/ [10^{-4}/K]	$\mu_{\mathrm{JT}}^{\mathrm{MC}}$/ [$10^{-3}$ K/bar]	$\mu_{\mathrm{JT}}^{\mathrm{REFP}}$/ [$10^{-3}$ K/bar]
20	35.9	36.4 ± 0.1	36.3	5472.6 ± 19.4	5491.6	42.0 ± 0.2	42.9	877.0 ± 15.0	935.3
40	79.3	80.6 ± 0.2	81.2	3059.9 ± 19.6	3135.7	57.8 ± 0.5	61.5	858.8 ± 16.9	922.2
60	135.8 ± 0.2	139.3 ± 0.4	141.8	2474.1 ± 32.3	2580.1	91.1 ± 2.0	100.4	857.3 ± 31.4	886.8
80	223.3 ± 0.6	236.3 ± 2.1	240.7	2586.6 ± 69.0	2894.7	183.4 ± 4.5	223.0	775.0 ± 27.1	778.1
100	447.4 ± 8.5	458.9 ± 9.0	464.9	3472.2 ± 341.6	2800.8	604.4 ± 68.9	495.5	469.2 ± 75.4	446.9
120	628.4 ± 2.6	624.3 ± 6.7	625.1	671.2 ± 23.6	751.2	212.1 ± 3.5	215.8	206.8 ± 4.9	224.4
140	693.6 ± 2.1	690.2 ± 2.3	693.4	329.6 ± 19.0	368.3	131.2 ± 7.8	132.6	135.7 ± 12.3	146.0
160	732.9 ± 4.1	731.7 ± 1.0	735.5	228.7 ± 25.0	240.0	103.8 ± 9.9	99.7	104.4 ± 15.7	106.7
180	762.0 ± 1.2	758.7 ± 1.7	766.4	165.3 ± 8.3	177.6	82.4 ± 3.7	81.8	79.6 ± 6.2	82.6
200	784.7 ± 1.7	782.4 ± 1.1	791.0	126.9 ± 7.2	140.9	69.2 ± 3.4	70.5	61.5 ± 6.0	65.8

Table S297: Heat capacities at constant volume computed from MC simulations (c_V^{MC}), heat capacities at constant volume obtained from REFPROP[10] (c_V^{REFP}), heat capacities at constant pressure computed from MC simulations (c_P^{MC}), heat capacities at constant pressure obtained from REFPROP[10] (c_P^{REFP}), speed of sound computed from MC simulations (c^{MC}), speed of sound obtained from REFPROP[10] (c^{REFP}), viscosities computed from MD simulations (η^{MD}), and viscosities obtained from REFPROP[10] (η^{REFP}) of CO_2 rich ternary mixture with 3% impurity of N_2 and 1% impurity of CH_4 at 313 K and pressures ranging from 20 bar to 200 bar.

P/ [bar]	c_V^{MC}/ [J/mol K]	c_V^{REFP}/ [J/mol K]	c_P^{MC}/ [J/mol K]	c_P^{REFP}/ [J/mol K]	c^{MC}/ [m/s]	c^{REFP}/ [m/s]	η^{MD}/ [μPa s]	η^{REFP}/ [μPa s]
20	31.0	31.1	43.1 ± 0.1	43.5	266.2 ± 0.5	265.1	5.6 ± 4.3	16.0
40	32.8	33.3	51.5 ± 0.2	53.4	254.4 ± 1.0	250.9	16.0 ± 1.7	16.6
60	35.3 ± 0.1	36.4	68.8 ± 1.1	73.8	240.9 ± 2.5	235.3	16.8 ± 1.0	17.7
80	38.9 ± 0.3	41.5	118.4 ± 2.1	138.0	229.6 ± 3.8	218.6	19.9 ± 0.9	20.7
100	42.7 ± 0.3	46.8	369.4 ± 38.7	302.1	236.2 ± 17.1	222.6	32.1 ± 3.8	32.9
120	40.5 ± 0.3	43.1	187.7 ± 2.4	177.4	331.5 ± 6.3	296.0	48.9 ± 3.9	47.8
140	39.7 ± 0.2	41.4	142.8 ± 6.5	134.6	396.7 ± 14.6	356.8	60.4 ± 5.9	56.3
160	39.3 ± 0.1	40.6	127.0 ± 7.8	116.8	439.0 ± 27.6	403.6	61.1 ± 1.5	62.3
180	39.1 ± 0.1	40.1	112.6 ± 3.2	106.8	478.1 ± 13.8	441.9	69.8 ± 3.4	67.2
200	38.9 ± 0.2	39.9	104.4 ± 3.0	100.2	519.4 ± 16.5	474.8	70.9 ± 3.5	71.4

S16.17 Data of thermodynamics and transport properties of CO_2 rich ternary mixture with 1 mole% impurity of N_2 and 3 mole% impurity of H_2

Table S298: Densities computed from MC and MD simulations (ρ^{MC} and ρ^{MD}), densities obtained from REFPROP[10] (ρ^{REFP}), isothermal compressibilities computed from MC simulations (β_T^{MC}), isothermal compressibilities obtained from REFPROP[10] (β_T^{REFP}), thermal expansion coefficients computed from MC simulations (α_P^{MC}), thermal expansion coefficients obtained from REFPROP[10] (α_P^{REFP}), Joule Thomson coefficients computed from MC simulations (μ_{JT}^{MC}), and Joule Thomson coefficients obtained from REFPROP[10] (μ_{JT}^{REFP}) of CO_2 rich ternary mixture with 3% impurity of H_2 and 1% impurity of N_2 at 253 K and pressures ranging from 20 bar to 200 bar.

P/ [bar]	ρ^{MC}/ [kg/m^3]	ρ^{MD}/ [kg/m^3]	ρ^{REFP}/ [kg/m^3]	β_T^{MC}/ [10^{-5}/bar]	β_T^{REFP}/ [10^{-5}/bar]	α_P^{MC}/ [10^{-4}/K]	α_P^{REFP}/ [10^{-4}/K]	μ_{JT}^{MC}/ [10^{-3} K/bar]	μ_{JT}^{REFP}/ [10^{-3} K/bar]
20	48.5	50.2 ± 0.2	49.9	6276.4 ± 28.5	6450.4	73.8 ± 0.5	79.6	1499.7 ± 22.1	1615.8
40	977.4 ± 2.4	986.1 ± 0.6	631.3	49.3 ± 3.0	−28.1	50.8 ± 2.7	93.7	12.7 ± 3.1	–
60	987.1 ± 1.2	994.0 ± 1.4	874.3	44.5 ± 2.2	250.5	47.4 ± 1.7	156.9	9.0 ± 2.0	–
80	994.7 ± 1.7	1003.6 ± 0.3	986.5	40.9 ± 1.5	45.3	44.9 ± 1.7	45.3	6.2 ± 1.9	7.0
100	1003.7 ± 1.0	1010.5 ± 1.3	995.1	37.5 ± 2.1	41.5	42.4 ± 1.9	42.7	3.3 ± 2.2	4.0
120	1011.5 ± 1.2	1017.5 ± 0.8	1003.1	34.6 ± 0.8	38.3	40.2 ± 0.9	40.6	0.8 ± 1.1	1.3
140	1018.5 ± 0.6	1024.8 ± 0.7	1010.5	32.6 ± 0.8	35.7	38.4 ± 1.0	38.7	-1.3 ± 1.2	−1.0
160	1024.6 ± 0.8	1031.2 ± 0.6	1017.5	30.9 ± 0.9	33.4	37.7 ± 1.2	37.1	-2.2 ± 1.5	−3.1
180	1030.7 ± 0.2	1037.3 ± 0.5	1024.2	29.2 ± 1.0	31.5	36.0 ± 1.3	35.7	-4.3 ± 1.6	−4.9
200	1037.2 ± 1.2	1043.3 ± 0.3	1030.4	27.6 ± 1.1	29.7	34.8 ± 1.6	34.4	-5.8 ± 2.0	−6.5

Table S299: Heat capacities at constant volume computed from MC simulations (c_V^{MC}), heat capacities at constant volume obtained from REFPROP[10] (c_V^{REFP}), heat capacities at constant pressure computed from MC simulations (c_P^{MC}), heat capacities at constant pressure obtained from REFPROP[10] (c_P^{REFP}), speed of sound computed from MC simulations (c^{MC}), speed of sound obtained from REFPROP[10] (c^{REFP}), viscosities computed from MD simulations (η^{MD}), and viscosities obtained from REFPROP[10] (η^{REFP}) of CO_2 rich ternary mixture with 3% impurity of H_2 and 1% impurity of N_2 at 253 K and pressures ranging from 20 bar to 200 bar.

P/ [bar]	c_V^{MC}/ [J/mol K]	c_V^{REFP}/ [J/mol K]	c_P^{MC}/ [J/mol K]	c_P^{REFP}/ [J/mol K]	c^{MC}/ [m/s]	c^{REFP}/ [m/s]	η^{MD}/ [μPa s]	η^{REFP}/ [μPa s]
20	31.4	32.3	50.7 ± 0.2	53.5	230.2 ± 0.7	226.8	12.3 ± 4.5	13.0
40	40.3 ± 0.2	–	98.1 ± 3.0	–	710.2 ± 24.2	–	131.9 ± 4.8	59.4
60	40.2 ± 0.1	–	95.8 ± 1.4	–	735.9 ± 18.6	–	130.3 ± 7.6	89.5
80	40.2 ± 0.1	40.0	93.7 ± 2.2	89.5	757.1 ± 16.5	707.6	143.5 ± 3.0	126.4
100	40.1 ± 0.1	40.0	91.9 ± 2.1	87.7	780.7 ± 24.1	728.6	143.2 ± 9.7	130.0
120	40.1 ± 0.2	40.0	90.1 ± 1.2	86.1	801.2 ± 10.8	748.1	148.0 ± 10.8	133.5
140	40.3 ± 0.2	40.0	88.3 ± 1.6	84.8	811.8 ± 12.5	766.4	150.4 ± 17.8	136.9
160	40.4 ± 0.3	40.0	88.6 ± 2.0	83.7	831.9 ± 16.0	783.7	175.6 ± 25.3	140.1
180	40.3 ± 0.3	40.1	86.6 ± 2.1	82.6	845.1 ± 17.7	800.0	158.3 ± 9.5	143.3
200	40.3 ± 0.3	40.1	85.8 ± 2.4	81.7	862.0 ± 21.4	815.6	161.3 ± 19.3	146.4

Table S300: Densities computed from MC and MD simulations (ρ^{MC} and ρ^{MD}), densities obtained from REFPROP[10] (ρ^{REFP}), isothermal compressibilities computed from MC simulations (β_T^{MC}), isothermal compressibilities obtained from REFPROP[10] (β_T^{REFP}), thermal expansion coefficients computed from MC simulations (α_P^{MC}), thermal expansion coefficients obtained from REFPROP[10] (α_P^{REFP}), Joule Thomson coefficients computed from MC simulations ($\mu_{\mathrm{JT}}^{\mathrm{MC}}$), and Joule Thomson coefficients obtained from REFPROP[10] ($\mu_{\mathrm{JT}}^{\mathrm{REFP}}$) of CO_2 rich ternary mixture with 3% impurity of H_2 and 1% impurity of N_2 at 273 K and pressures ranging from 20 bar to 200 bar.

P/ [bar]	ρ^{MC}/ [kg/m^3]	ρ^{MD}/ [kg/m^3]	ρ^{REFP}/ [kg/m^3]	β_T^{MC}/ [10^{-5}/bar]	β_T^{REFP}/ [10^{-5}/bar]	α_P^{MC}/ [10^{-4}/K]	α_P^{REFP}/ [10^{-4}/K]	$\mu_{\mathrm{JT}}^{\mathrm{MC}}$/ [$10^{-3}$ K/bar]	$\mu_{\mathrm{JT}}^{\mathrm{REFP}}$/ [$10^{-3}$ K/bar]
20	42.7	44.0 ± 0.1	43.6	5796.9 ± 44.1	5912.8	56.6 ± 0.4	59.5	1192.0 ± 24.6	1305.8
40	106.2 ± 0.3	864.9 ± 2.0	217.7	4129.5 ± 114.8	−12 099.6	124.5 ± 4.1	−1221.6	1235.8 ± 63.7	–
60	874.9 ± 2.9	884.6 ± 1.8	712.8	109.4 ± 10.4	−1725.0	78.6 ± 6.6	−520.1	48.2 ± 8.0	–
80	892.8 ± 1.4	902.7 ± 2.2	883.5	86.0 ± 5.8	98.7	65.9 ± 3.3	69.1	35.9 ± 4.1	41.2
100	905.8 ± 1.9	916.4 ± 0.5	899.6	72.3 ± 5.1	82.0	58.5 ± 4.1	60.8	27.8 ± 5.3	31.9
120	920.5 ± 2.2	926.2 ± 0.6	913.3	63.2 ± 4.2	70.5	54.2 ± 3.2	54.9	22.5 ± 4.2	24.8
140	930.6 ± 1.0	939.5 ± 1.1	925.5	61.9 ± 3.9	62.0	54.3 ± 3.3	50.5	22.2 ± 4.2	19.1
160	940.9 ± 1.9	948.7 ± 0.8	936.4	51.4 ± 1.8	55.6	47.0 ± 1.9	46.9	13.8 ± 2.6	14.4
180	950.2 ± 1.5	958.8 ± 0.7	946.3	48.1 ± 1.8	50.4	45.3 ± 1.7	44.1	11.6 ± 2.3	10.5
200	959.0 ± 1.2	966.6 ± 0.7	955.5	44.3 ± 1.1	46.2	43.0 ± 0.9	41.6	8.5 ± 1.3	7.1

Table S301: Heat capacities at constant volume computed from MC simulations (c_V^{MC}), heat capacities at constant volume obtained from REFPROP[10] (c_V^{REFP}), heat capacities at constant pressure computed from MC simulations (c_P^{MC}), heat capacities at constant pressure obtained from REFPROP[10] (c_P^{REFP}), speed of sound computed from MC simulations (c^{MC}), speed of sound obtained from REFPROP[10] (c^{REFP}), viscosities computed from MD simulations (η^{MD}), and viscosities obtained from REFPROP[10] (η^{REFP}) of CO_2 rich ternary mixture with 3% impurity of H_2 and 1% impurity of N_2 at 273 K and pressures ranging from 20 bar to 200 bar.

P/ [bar]	c_V^{MC}/ [J/mol K]	c_V^{REFP}/ [J/mol K]	c_P^{MC}/ [J/mol K]	c_P^{REFP}/ [J/mol K]	c^{MC}/ [m/s]	c^{REFP}/ [m/s]	η^{MD}/ [μPa s]	η^{REFP}/ [μPa s]
20	30.6	30.8	45.6 ± 0.1	46.8	245.6 ± 1.0	242.9	11.6 ± 2.1	14.0
40	36.5 ± 0.1	–	77.8 ± 1.7	–	220.5 ± 4.0	–	88.5 ± 2.4	17.5
60	39.7 ± 0.3	–	115.6 ± 5.9	–	551.7 ± 29.9	–	95.4 ± 2.6	57.8
80	39.6 ± 0.2	40.0	106.0 ± 2.6	103.6	590.3 ± 21.2	545.0	99.8 ± 1.6	91.3
100	39.6 ± 0.2	39.8	100.9 ± 4.3	98.1	623.7 ± 25.9	578.5	102.3 ± 2.4	95.6
120	39.8 ± 0.2	39.7	98.3 ± 3.4	94.2	651.9 ± 24.4	607.5	110.2 ± 8.4	99.5
140	39.7 ± 0.2	39.6	99.4 ± 3.5	91.2	658.9 ± 23.7	633.4	120.5 ± 13.9	103.1
160	39.8 ± 0.1	39.5	93.0 ± 2.8	88.8	695.3 ± 15.9	657.0	116.2 ± 7.2	106.5
180	39.8 ± 0.2	39.5	92.0 ± 2.0	86.8	711.1 ± 15.5	678.7	129.4 ± 20.3	109.8
200	39.6 ± 0.2	39.5	90.2 ± 1.2	85.2	731.8 ± 10.6	698.8	130.3 ± 15.3	112.9

Table S302: Densities computed from MC and MD simulations (ρ^{MC} and ρ^{MD}), densities obtained from REFPROP[10] (ρ^{REFP}), isothermal compressibilities computed from MC simulations (β_T^{MC}), isothermal compressibilities obtained from REFPROP[10] (β_T^{REFP}), thermal expansion coefficients computed from MC simulations (α_P^{MC}), thermal expansion coefficients obtained from REFPROP[10] (α_P^{REFP}), Joule Thomson coefficients computed from MC simulations ($\mu_{\mathrm{JT}}^{\mathrm{MC}}$), and Joule Thomson coefficients obtained from REFPROP[10] ($\mu_{\mathrm{JT}}^{\mathrm{REFP}}$) of CO_2 rich ternary mixture with 3% impurity of H_2 and 1% impurity of N_2 at 293 K and pressures ranging from 20 bar to 200 bar.

P/ [bar]	ρ^{MC}/ [kg/m^3]	ρ^{MD}/ [kg/m^3]	ρ^{REFP}/ [kg/m^3]	β_T^{MC}/ [10^{-5}/bar]	β_T^{REFP}/ [10^{-5}/bar]	α_P^{MC}/ [10^{-4}/K]	α_P^{REFP}/ [10^{-4}/K]	$\mu_{\mathrm{JT}}^{\mathrm{MC}}$/ [$10^{-3}$ K/bar]	$\mu_{\mathrm{JT}}^{\mathrm{REFP}}$/ [$10^{-3}$ K/bar]
20	38.5	39.7 ± 0.1	39.1	5555.5 ± 28.7	5643.3	47.3 ± 0.3	49.2	980.0 ± 21.3	1083.0
40	88.2 ± 0.1	91.6 ± 0.2	91.4	3313.0 ± 49.6	3463.3	74.4 ± 1.0	81.7	997.9 ± 26.2	1077.4
60	166.9 ± 0.6	181.1 ± 1.4	182.1	3399.9 ± 82.4	3980.5	175.8 ± 6.9	229.3	985.3 ± 58.8	1012.6
80	707.8 ± 10.7	733.7 ± 3.1	681.6	581.1 ± 142.3	882.5	227.3 ± 43.8	297.3	168.4 ± 43.8	–
100	761.4 ± 4.4	778.7 ± 1.8	762.0	252.3 ± 40.4	259.5	123.6 ± 15.1	119.5	104.1 ± 19.1	106.7
120	796.0 ± 2.3	809.0 ± 2.2	794.7	166.4 ± 14.4	173.6	92.0 ± 5.1	90.3	75.2 ± 6.9	77.5
140	818.2 ± 3.1	832.4 ± 2.6	819.1	130.5 ± 5.7	132.0	78.8 ± 3.3	75.0	60.3 ± 4.7	59.5
160	839.5 ± 1.3	849.6 ± 1.2	838.7	101.1 ± 4.1	107.1	66.4 ± 2.8	65.2	45.9 ± 4.2	46.9
180	854.8 ± 0.8	865.4 ± 0.4	855.4	92.8 ± 1.7	90.4	63.0 ± 0.9	58.3	41.3 ± 1.3	37.5
200	870.3 ± 1.8	879.3 ± 0.9	869.9	74.9 ± 1.9	78.4	54.4 ± 1.5	53.2	30.3 ± 2.2	30.0

Table S303: Heat capacities at constant volume computed from MC simulations (c_V^{MC}), heat capacities at constant volume obtained from REFPROP[10] (c_V^{REFP}), heat capacities at constant pressure computed from MC simulations (c_P^{MC}), heat capacities at constant pressure obtained from REFPROP[10] (c_P^{REFP}), speed of sound computed from MC simulations (c^{MC}), speed of sound obtained from REFPROP[10] (c^{REFP}), viscosities computed from MD simulations (η^{MD}), and viscosities obtained from REFPROP[10] (η^{REFP}) of CO_2 rich ternary mixture with 3% impurity of H_2 and 1% impurity of N_2 at 293 K and pressures ranging from 20 bar to 200 bar.

P/ [bar]	c_V^{MC}/ [J/mol K]	c_V^{REFP}/ [J/mol K]	c_P^{MC}/ [J/mol K]	c_P^{REFP}/ [J/mol K]	c^{MC}/ [m/s]	c^{REFP}/ [m/s]	η^{MD}/ [μPa s]	η^{REFP}/ [μPa s]
20	30.6	30.6	43.6 ± 0.1	44.3	258.4 ± 0.7	256.0	7.4 ± 7.9	15.0
40	33.4	34.0	57.0 ± 0.4	60.3	241.7 ± 2.0	236.8	15.0 ± 0.6	15.6
60	38.9 ± 0.2	41.6	107.4 ± 3.7	132.1	220.7 ± 4.7	209.4	19.3 ± 2.4	17.6
80	41.3 ± 0.4	–	202.2 ± 25.7	–	345.1 ± 47.7	–	63.1 ± 1.2	53.9
100	40.4 ± 0.2	40.9	140.9 ± 9.9	131.1	426.1 ± 37.3	402.6	72.2 ± 5.0	65.9
120	39.6 ± 0.2	40.1	120.7 ± 2.9	113.9	479.5 ± 21.5	453.6	75.2 ± 3.5	71.7
140	39.5 ± 0.3	39.7	112.8 ± 3.2	104.6	517.3 ± 13.6	493.5	82.6 ± 5.6	76.5
160	39.3 ± 0.2	39.5	104.4 ± 3.1	98.6	559.7 ± 14.1	527.1	86.0 ± 1.7	80.6
180	39.2 ± 0.2	39.4	102.3 ± 0.9	94.3	573.3 ± 5.9	556.4	88.4 ± 8.5	84.3
200	39.4 ± 0.1	39.3	96.2 ± 1.7	91.0	612.2 ± 9.6	582.7	92.0 ± 2.6	87.7

Table S304: Densities computed from MC and MD simulations (ρ^{MC} and ρ^{MD}), densities obtained from REFPROP[10] (ρ^{REFP}), isothermal compressibilities computed from MC simulations (β_T^{MC}), isothermal compressibilities obtained from REFPROP[10] (β_T^{REFP}), thermal expansion coefficients computed from MC simulations (α_P^{MC}), thermal expansion coefficients obtained from REFPROP[10] (α_P^{REFP}), Joule Thomson coefficients computed from MC simulations ($\mu_{\mathrm{JT}}^{\mathrm{MC}}$), and Joule Thomson coefficients obtained from REFPROP[10] ($\mu_{\mathrm{JT}}^{\mathrm{REFP}}$) of CO_2 rich ternary mixture with 3% impurity of H_2 and 1% impurity of N_2 at 313 K and pressures ranging from 20 bar to 200 bar.

P/ [bar]	ρ^{MC}/ [kg/m^3]	ρ^{MD}/ [kg/m^3]	ρ^{REFP}/ [kg/m^3]	β_T^{MC}/ [10^{-5}/bar]	β_T^{REFP}/ [10^{-5}/bar]	α_P^{MC}/ [10^{-4}/K]	α_P^{REFP}/ [10^{-4}/K]	$\mu_{\mathrm{JT}}^{\mathrm{MC}}$/ [$10^{-3}$ K/bar]	$\mu_{\mathrm{JT}}^{\mathrm{REFP}}$/ [$10^{-3}$ K/bar]
20	35.2	36.3	35.7	5432.9 ± 24.5	5478.7	41.5 ± 0.2	42.5	842.1 ± 18.8	914.0
40	77.5 ± 0.1	80.0 ± 0.1	79.5	3029.2 ± 33.4	3112.5	56.7 ± 0.7	60.2	835.7 ± 24.5	903.3
60	131.7 ± 0.1	137.8 ± 0.7	137.8	2414.6 ± 50.2	2526.2	86.6 ± 2.4	95.8	831.6 ± 39.3	871.5
80	210.8 ± 0.7	227.7 ± 2.1	229.2	2437.7 ± 117.3	2699.6	162.0 ± 8.4	196.6	768.7 ± 56.3	776.6
100	370.6 ± 3.2	418.2 ± 7.0	424.3	3273.3 ± 125.7	2997.5	463.2 ± 12.1	478.4	549.0 ± 20.3	492.3
120	572.3 ± 3.3	601.3 ± 5.4	596.0	1026.6 ± 93.7	853.6	270.7 ± 20.9	232.5	264.1 ± 27.6	243.8
140	660.2 ± 2.4	675.3 ± 3.6	668.8	454.8 ± 26.5	399.2	161.7 ± 8.4	138.5	166.1 ± 12.4	155.5
160	705.6 ± 3.4	719.3 ± 0.9	712.4	264.1 ± 7.3	254.4	110.1 ± 3.4	102.4	116.1 ± 5.8	112.5
180	736.1 ± 1.7	751.4 ± 1.8	744.0	193.6 ± 21.5	186.1	89.5 ± 7.6	83.4	90.4 ± 12.7	86.5
200	764.4 ± 1.9	773.8 ± 0.7	768.9	151.4 ± 7.6	146.6	77.4 ± 3.4	71.5	73.0 ± 5.7	68.7

Table S305: Heat capacities at constant volume computed from MC simulations (c_V^{MC}), heat capacities at constant volume obtained from REFPROP[10] (c_V^{REFP}), heat capacities at constant pressure computed from MC simulations (c_P^{MC}), heat capacities at constant pressure obtained from REFPROP[10] (c_P^{REFP}), speed of sound computed from MC simulations (c^{MC}), speed of sound obtained from REFPROP[10] (c^{REFP}), viscosities computed from MD simulations (η^{MD}), and viscosities obtained from REFPROP[10] (η^{REFP}) of CO_2 rich ternary mixture with 3% impurity of H_2 and 1% impurity of N_2 at 313 K and pressures ranging from 20 bar to 200 bar.

P/ [bar]	c_V^{MC}/ [J/mol K]	c_V^{REFP}/ [J/mol K]	c_P^{MC}/ [J/mol K]	c_P^{REFP}/ [J/mol K]	c^{MC}/ [m/s]	c^{REFP}/ [m/s]	η^{MD}/ [μPa s]	η^{REFP}/ [μPa s]
20	30.9	30.9	42.9 ± 0.1	43.2	269.3 ± 0.6	267.5	3.8 ± 3.6	15.9
40	32.6	32.9	50.9 ± 0.3	52.5	257.8 ± 1.6	253.7	16.4 ± 1.2	16.5
60	35.1 ± 0.1	35.7	66.5 ± 1.1	70.9	244.2 ± 3.3	238.7	17.2 ± 1.1	17.6
80	38.3 ± 0.2	40.0	107.0 ± 3.7	123.3	233.0 ± 7.0	223.1	22.2 ± 2.6	20.2
100	42.9 ± 0.9	45.0	282.7 ± 6.4	284.9	233.2 ± 5.8	223.0	27.0 ± 1.0	29.9
120	41.4 ± 0.4	42.4	210.6 ± 11.9	184.0	294.3 ± 15.9	292.1	43.7 ± 2.9	44.5
140	40.0 ± 0.2	40.8	157.8 ± 5.7	136.5	362.6 ± 12.5	354.1	56.5 ± 4.4	52.9
160	39.6 ± 0.3	40.0	127.1 ± 3.2	117.1	414.9 ± 8.0	401.9	58.6 ± 3.6	58.8
180	39.3 ± 0.3	39.6	115.3 ± 5.3	106.6	453.6 ± 27.3	440.9	69.8 ± 6.8	63.6
200	39.3 ± 0.4	39.3	108.6 ± 2.7	99.8	488.8 ± 14.0	474.3	65.9 ± 7.0	67.6

S16.18 Data of thermodynamics and transport properties of CO_2 rich ternary mixture with 3 mole% impurity of N_2 and 1 mole% impurity of H_2

Table S306: Densities computed from MC and MD simulations (ρ^{MC} and ρ^{MD}), densities obtained from REFPROP[10] (ρ^{REFP}), isothermal compressibilities computed from MC simulations (β_T^{MC}), isothermal compressibilities obtained from REFPROP[10] (β_T^{REFP}), thermal expansion coefficients computed from MC simulations (α_P^{MC}), thermal expansion coefficients obtained from REFPROP[10] (α_P^{REFP}), Joule Thomson coefficients computed from MC simulations ($\mu_{\mathrm{JT}}^{\mathrm{MC}}$), and Joule Thomson coefficients obtained from REFPROP[10] ($\mu_{\mathrm{JT}}^{\mathrm{REFP}}$) of CO_2 rich ternary mixture with 3% impurity of N_2 and 1% impurity of H_2 at 253 K and pressures ranging from 20 bar to 200 bar.

P/ [bar]	ρ^{MC}/ [kg/m^3]	ρ^{MD}/ [kg/m^3]	ρ^{REFP}/ [kg/m^3]	β_T^{MC}/ [10^{-5}/bar]	β_T^{REFP}/ [10^{-5}/bar]	α_P^{MC}/ [10^{-4}/K]	α_P^{REFP}/ [10^{-4}/K]	$\mu_{\mathrm{JT}}^{\mathrm{MC}}$/ [$10^{-3}$ K/bar]	$\mu_{\mathrm{JT}}^{\mathrm{REFP}}$/ [$10^{-3}$ K/bar]
20	49.3	50.2 ± 0.2	50.8	6309.1 ± 74.1	6498.6	74.6 ± 1.0	81.3	1523.2 ± 45.3	1644.8
40	987.8 ± 1.4	986.1 ± 0.6	748.7	45.9 ± 2.8	-187.5	48.3 ± 3.1	-12.1	10.0 ± 3.6	–
60	996.5 ± 1.5	994.0 ± 1.4	996.3	42.6 ± 3.5	48.9	46.4 ± 3.4	48.0	7.9 ± 3.9	10.1
80	1004.6 ± 1.7	1003.6 ± 0.3	1005.6	40.7 ± 1.6	44.4	45.7 ± 1.5	45.0	7.0 ± 1.7	6.6
100	1011.7 ± 1.8	1010.5 ± 1.3	1014.2	36.0 ± 1.4	40.7	41.2 ± 1.6	42.5	1.9 ± 1.9	3.6
120	1020.0 ± 1.5	1017.5 ± 0.8	1022.1	33.6 ± 2.0	37.7	39.5 ± 2.2	40.4	-0.1 ± 2.6	1.0
140	1025.7 ± 1.3	1024.8 ± 0.7	1029.6	32.9 ± 1.3	35.1	39.4 ± 1.9	38.5	-0.2 ± 2.3	-1.2
160	1032.4 ± 0.8	1031.2 ± 0.6	1036.6	29.9 ± 1.0	32.9	36.6 ± 1.4	36.9	-3.5 ± 1.7	-3.2
180	1039.2 ± 1.1	1037.3 ± 0.5	1043.3	28.7 ± 0.9	31.0	36.3 ± 1.1	35.5	-3.8 ± 1.3	-5.0
200	1044.2 ± 1.3	1043.3 ± 0.3	1049.6	27.1 ± 1.0	29.3	34.7 ± 1.4	34.3	-5.9 ± 1.7	-6.6

Table S307: Heat capacities at constant volume computed from MC simulations (c_V^{MC}), heat capacities at constant volume obtained from REFPROP[10] (c_V^{REFP}), heat capacities at constant pressure computed from MC simulations (c_P^{MC}), heat capacities at constant pressure obtained from REFPROP[10] (c_P^{REFP}), speed of sound computed from MC simulations (c^{MC}), speed of sound obtained from REFPROP[10] (c^{REFP}), viscosities computed from MD simulations (η^{MD}), and viscosities obtained from REFPROP[10] (η^{REFP}) of CO_2 rich ternary mixture with 3% impurity of N_2 and 1% impurity of H_2 at 253 K and pressures ranging from 20 bar to 200 bar.

P/ [bar]	c_V^{MC}/ [J/mol K]	c_V^{REFP}/ [J/mol K]	c_P^{MC}/ [J/mol K]	c_P^{REFP}/ [J/mol K]	c^{MC}/ [m/s]	c^{REFP}/ [m/s]	η^{MD}/ [μPa s]	η^{REFP}/ [μPa s]
20	31.5 ± 0.1	32.7	51.0 ± 0.3	54.5	228.0 ± 1.5	224.8	12.3 ± 4.5	13.0
40	40.1 ± 0.2	–	96.5 ± 4.0	–	728.6 ± 27.3	–	131.9 ± 4.8	65.9
60	40.2 ± 0.1	40.4	96.0 ± 4.1	91.9	749.6 ± 34.7	683.5	130.3 ± 7.6	128.2
80	40.2 ± 0.2	40.3	96.0 ± 1.8	89.8	764.1 ± 16.4	706.2	143.5 ± 3.0	132.0
100	40.2 ± 0.1	40.3	91.0 ± 2.1	88.0	789.1 ± 18.1	727.0	143.2 ± 9.7	135.8
120	40.1 ± 0.2	40.3	89.7 ± 2.6	86.5	807.2 ± 26.6	746.4	148.0 ± 10.8	139.3
140	40.4 ± 0.2	40.3	90.4 ± 3.2	85.2	814.5 ± 21.5	764.6	150.4 ± 17.8	142.8
160	40.3 ± 0.2	40.3	87.7 ± 2.4	84.0	840.1 ± 18.6	781.7	175.6 ± 25.3	146.1
180	40.5 ± 0.2	40.4	88.4 ± 1.7	83.0	855.2 ± 15.5	798.0	158.3 ± 9.5	149.4
200	40.4 ± 0.1	40.4	86.6 ± 2.1	82.1	870.3 ± 19.2	813.5	161.3 ± 19.3	152.6

Table S308: Densities computed from MC and MD simulations (ρ^{MC} and ρ^{MD}), densities obtained from REFPROP[10] (ρ^{REFP}), isothermal compressibilities computed from MC simulations (β_T^{MC}), isothermal compressibilities obtained from REFPROP[10] (β_T^{REFP}), thermal expansion coefficients computed from MC simulations (α_P^{MC}), thermal expansion coefficients obtained from REFPROP[10] (α_P^{REFP}), Joule Thomson coefficients computed from MC simulations ($\mu_{\mathrm{JT}}^{\mathrm{MC}}$), and Joule Thomson coefficients obtained from REFPROP[10] ($\mu_{\mathrm{JT}}^{\mathrm{REFP}}$) of CO_2 rich ternary mixture with 3% impurity of N_2 and 1% impurity of H_2 at 273 K and pressures ranging from 20 bar to 200 bar.

P/ [bar]	ρ^{MC}/ [kg/m^3]	ρ^{MD}/ [kg/m^3]	ρ^{REFP}/ [kg/m^3]	β_T^{MC}/ [10^{-5}/bar]	β_T^{REFP}/ [10^{-5}/bar]	α_P^{MC}/ [10^{-4}/K]	α_P^{REFP}/ [10^{-4}/K]	$\mu_{\mathrm{JT}}^{\mathrm{MC}}$/ [$10^{-3}$ K/bar]	$\mu_{\mathrm{JT}}^{\mathrm{REFP}}$/ [$10^{-3}$ K/bar]
20	43.3	44.0 ± 0.1	44.2	5837.9 ± 7.7	5933.1	57.1 ± 0.1	60.2	1217.0 ± 6.4	1327.8
40	108.3 ± 0.3	864.9 ± 2.0	232.7	4161.4 ± 57.7	-5797.5	126.0 ± 2.8	-694.3	1242.8 ± 44.3	–
60	884.9 ± 2.2	884.6 ± 1.8	863.6	101.3 ± 4.2	157.3	74.4 ± 2.8	96.4	44.3 ± 3.4	–
80	905.6 ± 0.8	902.7 ± 2.2	901.4	79.5 ± 5.4	96.4	63.8 ± 3.7	68.4	33.1 ± 4.6	40.0
100	918.2 ± 1.6	916.4 ± 0.5	917.4	70.1 ± 3.2	80.3	58.6 ± 2.8	60.4	27.4 ± 3.6	31.0
120	930.1 ± 1.0	926.2 ± 0.6	931.1	61.6 ± 3.0	69.2	53.8 ± 2.0	54.6	21.9 ± 2.5	24.1
140	941.4 ± 1.0	939.5 ± 1.1	943.3	54.7 ± 1.4	61.0	49.4 ± 1.3	50.2	16.7 ± 1.7	18.6
160	952.2 ± 1.1	948.7 ± 0.8	954.2	48.7 ± 1.3	54.7	45.4 ± 1.1	46.8	11.7 ± 1.5	14.0
180	960.1 ± 0.5	958.8 ± 0.7	964.2	45.3 ± 1.7	49.6	43.9 ± 2.1	43.9	9.7 ± 2.9	10.2
200	969.0 ± 0.6	966.6 ± 0.7	973.4	43.0 ± 0.4	45.5	42.3 ± 0.3	41.5	7.7 ± 0.5	6.9

Table S309: Heat capacities at constant volume computed from MC simulations (c_V^{MC}), heat capacities at constant volume obtained from REFPROP[10] (c_V^{REFP}), heat capacities at constant pressure computed from MC simulations (c_P^{MC}), heat capacities at constant pressure obtained from REFPROP[10] (c_P^{REFP}), speed of sound computed from MC simulations (c^{MC}), speed of sound obtained from REFPROP[10] (c^{REFP}), viscosities computed from MD simulations (η^{MD}), and viscosities obtained from REFPROP[10] (η^{REFP}) of CO_2 rich ternary mixture with 3% impurity of N_2 and 1% impurity of H_2 at 273 K and pressures ranging from 20 bar to 200 bar.

P/ [bar]	c_V^{MC}/ [J/mol K]	c_V^{REFP}/ [J/mol K]	c_P^{MC}/ [J/mol K]	c_P^{REFP}/ [J/mol K]	c^{MC}/ [m/s]	c^{REFP}/ [m/s]	η^{MD}/ [μPa s]	η^{REFP}/ [μPa s]
20	30.6	31.0	45.8 ± 0.1	47.2	243.2 ± 0.2	241.1	11.6 ± 2.1	14.1
40	36.6 ± 0.2	–	78.1 ± 1.4	–	217.8 ± 2.5	–	88.5 ± 2.4	18.1
60	39.9 ± 0.4	–	113.4 ± 2.7	–	563.0 ± 13.8	–	95.4 ± 2.6	85.7
80	39.8 ± 0.4	40.3	106.6 ± 3.3	103.7	609.5 ± 22.9	543.9	99.8 ± 1.6	95.2
100	39.6 ± 0.2	40.1	102.8 ± 3.4	98.4	634.9 ± 17.7	577.0	102.3 ± 2.4	99.6
120	39.8 ± 0.3	40.0	99.1 ± 1.7	94.5	658.8 ± 17.1	605.9	110.2 ± 8.4	103.6
140	39.6 ± 0.2	39.9	95.6 ± 1.5	91.6	685.0 ± 10.5	631.6	120.5 ± 13.9	107.4
160	39.5 ± 0.1	39.9	92.3 ± 1.6	89.2	709.2 ± 11.5	655.0	116.2 ± 7.2	110.9
180	39.6 ± 0.1	39.8	91.7 ± 3.3	87.3	729.7 ± 18.9	676.6	129.4 ± 20.3	114.2
200	39.6 ± 0.1	39.8	90.3 ± 0.4	85.6	739.8 ± 4.1	696.6	130.3 ± 15.3	117.4

Table S310: Densities computed from MC and MD simulations (ρ^{MC} and ρ^{MD}), densities obtained from REFPROP[10] (ρ^{REFP}), isothermal compressibilities computed from MC simulations (β_T^{MC}), isothermal compressibilities obtained from REFPROP[10] (β_T^{REFP}), thermal expansion coefficients computed from MC simulations (α_P^{MC}), thermal expansion coefficients obtained from REFPROP[10] (α_P^{REFP}), Joule Thomson coefficients computed from MC simulations (μ_{JT}^{MC}), and Joule Thomson coefficients obtained from REFPROP[10] (μ_{JT}^{REFP}) of CO_2 rich ternary mixture with 3% impurity of N_2 and 1% impurity of H_2 at 293 K and pressures ranging from 20 bar to 200 bar.

P/ [bar]	ρ^{MC}/ [kg/m^3]	ρ^{MD}/ [kg/m^3]	ρ^{REFP}/ [kg/m^3]	β_T^{MC}/ [10^{-5}/bar]	β_T^{REFP}/ [10^{-5}/bar]	α_P^{MC}/ [10^{-4}/K]	α_P^{REFP}/ [10^{-4}/K]	μ_{JT}^{MC}/ [10^{-3} K/bar]	μ_{JT}^{REFP}/ [10^{-3} K/bar]
20	39.0	39.7 ± 0.1	39.7	5562.7 ± 29.6	5654.4	47.5 ± 0.2	49.5	991.1 ± 17.9	1100.2
40	89.8	91.6 ± 0.2	93.0	3341.0 ± 40.2	3491.5	75.8 ± 1.0	83.5	1016.1 ± 24.0	1091.1
60	172.3 ± 1.0	181.1 ± 1.4	188.4	3674.3 ± 129.5	4244.1	196.1 ± 10.1	254.9	1017.3 ± 80.0	1017.3
80	732.5 ± 6.7	733.7 ± 3.1	725.1	452.7 ± 16.7	532.2	192.7 ± 7.5	202.7	147.7 ± 8.4	165.1
100	782.2 ± 2.4	778.7 ± 1.8	778.4	209.6 ± 6.2	251.8	108.5 ± 3.0	117.7	91.3 ± 4.1	103.8
120	810.9 ± 1.6	809.0 ± 2.2	811.0	151.2 ± 5.1	169.9	87.8 ± 2.4	89.6	69.9 ± 3.2	75.7
140	832.7 ± 2.2	832.4 ± 2.6	835.3	120.6 ± 6.3	129.7	76.0 ± 3.7	74.6	56.5 ± 5.3	58.3
160	852.7 ± 2.8	849.6 ± 1.2	855.1	99.2 ± 5.4	105.4	66.4 ± 3.2	65.0	45.3 ± 4.7	46.0
180	866.8 ± 1.7	865.4 ± 0.4	871.8	83.6 ± 6.0	89.2	58.9 ± 3.6	58.2	36.0 ± 5.4	36.8
200	881.1 ± 1.5	879.3 ± 0.9	886.4	69.7 ± 1.4	77.4	52.0 ± 1.2	53.1	27.0 ± 1.9	29.5

Table S311: Heat capacities at constant volume computed from MC simulations (c_V^{MC}), heat capacities at constant volume obtained from REFPROP[10] (c_V^{REFP}), heat capacities at constant pressure computed from MC simulations (c_P^{MC}), heat capacities at constant pressure obtained from REFPROP[10] (c_P^{REFP}), speed of sound computed from MC simulations (c^{MC}), speed of sound obtained from REFPROP[10] (c^{REFP}), viscosities computed from MD simulations (η^{MD}), and viscosities obtained from REFPROP[10] (η^{REFP}) of CO_2 rich ternary mixture with 3% impurity of N_2 and 1% impurity of H_2 at 293 K and pressures ranging from 20 bar to 200 bar.

P/ [bar]	c_V^{MC}/ [J/mol K]	c_V^{REFP}/ [J/mol K]	c_P^{MC}/ [J/mol K]	c_P^{REFP}/ [J/mol K]	c^{MC}/ [m/s]	c^{REFP}/ [m/s]	η^{MD}/ [μPa s]	η^{REFP}/ [μPa s]
20	30.6	30.7	43.7 ± 0.1	44.6	256.6 ± 0.7	254.2	7.4 ± 7.9	15.0
40	33.4	34.3	57.7 ± 0.3	61.4	239.8 ± 1.6	234.8	15.0 ± 0.6	15.7
60	39.2 ± 0.6	42.9	116.8 ± 5.6	145.5	217.0 ± 6.7	206.0	19.3 ± 2.4	17.9
80	41.0 ± 0.3	43.4	185.2 ± 5.6	177.8	369.2 ± 9.1	325.8	63.1 ± 1.2	60.0
100	39.9 ± 0.3	41.3	131.7 ± 2.8	130.6	448.8 ± 8.4	401.5	72.2 ± 5.0	68.6
120	39.5 ± 0.2	40.5	119.6 ± 1.6	114.1	496.6 ± 9.2	451.9	75.2 ± 3.5	74.5
140	39.6 ± 0.3	40.1	112.4 ± 3.5	105.0	531.6 ± 16.3	491.5	82.6 ± 5.6	79.4
160	39.2 ± 0.3	39.9	105.7 ± 2.9	99.0	564.7 ± 17.4	524.9	86.0 ± 1.7	83.6
180	39.1 ± 0.2	39.7	100.1 ± 3.3	94.7	594.1 ± 23.5	554.1	88.4 ± 8.5	87.4
200	39.2 ± 0.1	39.6	95.0 ± 1.6	91.5	627.9 ± 8.4	580.3	92.0 ± 2.6	90.9

Table S312: Densities computed from MC and MD simulations (ρ^{MC} and ρ^{MD}), densities obtained from REFPROP[10] (ρ^{REFP}), isothermal compressibilities computed from MC simulations (β_T^{MC}), isothermal compressibilities obtained from REFPROP[10] (β_T^{REFP}), thermal expansion coefficients computed from MC simulations (α_P^{MC}), thermal expansion coefficients obtained from REFPROP[10] (α_P^{REFP}), Joule Thomson coefficients computed from MC simulations ($\mu_{\mathrm{JT}}^{\mathrm{MC}}$), and Joule Thomson coefficients obtained from REFPROP[10] ($\mu_{\mathrm{JT}}^{\mathrm{REFP}}$) of CO_2 rich ternary mixture with 3% impurity of N_2 and 1% impurity of H_2 at 313 K and pressures ranging from 20 bar to 200 bar.

P/ [bar]	ρ^{MC}/ [kg/m^3]	ρ^{MD}/ [kg/m^3]	ρ^{REFP}/ [kg/m^3]	β_T^{MC}/ [10^{-5}/bar]	β_T^{REFP}/ [10^{-5}/bar]	α_P^{MC}/ [10^{-4}/K]	α_P^{REFP}/ [10^{-4}/K]	$\mu_{\mathrm{JT}}^{\mathrm{MC}}$/ [$10^{-3}$ K/bar]	$\mu_{\mathrm{JT}}^{\mathrm{REFP}}$/ [$10^{-3}$ K/bar]
20	35.7	36.3	36.2	5412.0 ± 16.8	5485.6	41.5 ± 0.1	42.7	837.3 ± 12.2	927.7
40	78.7	80.0 ± 0.1	80.8	3036.2 ± 19.8	3124.5	57.1 ± 0.3	61.0	843.8 ± 10.5	914.4
60	134.4 ± 0.2	137.8 ± 0.7	140.4	2446.6 ± 25.0	2554.1	89.0 ± 0.9	98.3	845.0 ± 14.7	879.9
80	218.3 ± 1.0	227.7 ± 2.1	236.1	2585.2 ± 130.3	2795.2	179.3 ± 9.9	209.9	782.0 ± 61.9	777.0
100	410.5 ± 7.2	418.2 ± 7.0	444.9	3752.3 ± 268.4	2883.3	598.0 ± 57.7	483.1	514.5 ± 71.9	469.2
120	603.2 ± 4.8	601.3 ± 5.4	611.6	923.4 ± 130.0	811.3	265.5 ± 31.0	225.4	243.2 ± 38.1	235.6
140	678.3 ± 3.0	675.3 ± 3.6	683.3	379.6 ± 24.3	388.4	142.1 ± 7.6	136.6	149.1 ± 11.6	151.8
160	722.7 ± 2.8	719.3 ± 0.9	726.9	236.5 ± 18.6	249.8	103.7 ± 6.3	101.7	107.1 ± 10.0	110.4
180	752.4 ± 1.8	751.4 ± 1.8	758.6	184.8 ± 9.1	183.5	89.1 ± 3.8	83.2	87.7 ± 6.2	85.1
200	777.2 ± 1.6	773.8 ± 0.7	783.6	142.5 ± 7.8	144.9	75.2 ± 4.3	71.4	69.4 ± 7.4	67.7

Table S313: Heat capacities at constant volume computed from MC simulations (c_V^{MC}), heat capacities at constant volume obtained from REFPROP[10] (c_V^{REFP}), heat capacities at constant pressure computed from MC simulations (c_P^{MC}), heat capacities at constant pressure obtained from REFPROP[10] (c_P^{REFP}), speed of sound computed from MC simulations (c^{MC}), speed of sound obtained from REFPROP[10] (c^{REFP}), viscosities computed from MD simulations (η^{MD}), and viscosities obtained from REFPROP[10] (η^{REFP}) of CO_2 rich ternary mixture with 3% impurity of N_2 and 1% impurity of H_2 at 313 K and pressures ranging from 20 bar to 200 bar.

P/ [bar]	c_V^{MC}/ [J/mol K]	c_V^{REFP}/ [J/mol K]	c_P^{MC}/ [J/mol K]	c_P^{REFP}/ [J/mol K]	c^{MC}/ [m/s]	c^{REFP}/ [m/s]	η^{MD}/ [μPa s]	η^{REFP}/ [μPa s]
20	30.9	31.0	42.9	43.4	267.9 ± 0.4	265.7	3.8 ± 3.6	16.0
40	32.7	33.2	51.1 ± 0.1	53.0	255.7 ± 0.9	251.8	16.4 ± 1.2	16.6
60	35.1 ± 0.1	36.1	67.7 ± 0.5	72.5	242.1 ± 1.5	236.5	17.2 ± 1.1	17.7
80	38.9 ± 0.3	40.8	116.5 ± 4.8	130.9	230.5 ± 7.5	220.4	22.2 ± 2.6	20.5
100	42.8 ± 0.5	46.1	361.9 ± 34.0	291.6	234.4 ± 14.1	222.0	27.0 ± 1.0	31.4
120	40.8 ± 0.4	43.0	214.8 ± 17.8	181.2	307.4 ± 25.2	291.5	43.7 ± 2.9	46.2
140	39.8 ± 0.3	41.3	146.9 ± 5.1	136.1	378.8 ± 13.9	352.5	56.5 ± 4.4	54.8
160	39.5 ± 0.2	40.4	125.1 ± 4.2	117.4	430.4 ± 18.5	399.9	58.6 ± 3.6	60.8
180	39.2 ± 0.1	40.0	116.8 ± 2.9	107.0	463.0 ± 12.8	438.5	69.8 ± 6.8	65.7
200	39.1 ± 0.1	39.7	108.3 ± 4.2	100.3	500.0 ± 16.8	471.7	65.9 ± 7.0	69.9

S16.19 Data of thermodynamics and transport properties of CO_2 rich ternary mixture with 1 mole% impurity of Ar and 3 mole% impurity of CH_4

Table S314: Densities computed from MC and MD simulations (ρ^{MC} and ρ^{MD}), densities obtained from REFPROP[10] (ρ^{REFP}), isothermal compressibilities computed from MC simulations (β_T^{MC}), isothermal compressibilities obtained from REFPROP[10] (β_T^{REFP}), thermal expansion coefficients computed from MC simulations (α_P^{MC}), thermal expansion coefficients obtained from REFPROP[10] (α_P^{REFP}), Joule Thomson coefficients computed from MC simulations (μ_{JT}^{MC}), and Joule Thomson coefficients obtained from REFPROP[10] (μ_{JT}^{REFP}) of CO_2 rich ternary mixture with 3% impurity of CH_4 and 1% impurity of Ar at 253 K and pressures ranging from 20 bar to 200 bar.

P/ [bar]	ρ^{MC}/ [kg/m^3]	ρ^{MD}/ [kg/m^3]	ρ^{REFP}/ [kg/m^3]	β_T^{MC}/ [10^{-5}/bar]	β_T^{REFP}/ [10^{-5}/bar]	α_P^{MC}/ [10^{-4}/K]	α_P^{REFP}/ [10^{-4}/K]	μ_{JT}^{MC}/ [10^{-3} K/bar]	μ_{JT}^{REFP}/ [10^{-3} K/bar]
20	49.0	50.7 ± 0.2	51.0	6300.1 ± 26.0	6556.5	74.5 ± 0.3	82.9	1530.6 ± 12.0	1670.3
40	981.5 ± 1.5	1007.4 ± 1.8	995.1	43.5 ± 3.0	49.8	46.7 ± 2.9	49.2	8.4 ± 3.4	11.3
60	988.8 ± 1.5	1016.3 ± 0.8	1004.5	40.9 ± 2.2	45.0	45.5 ± 2.6	45.9	6.9 ± 3.0	7.6
80	996.9 ± 1.1	1025.3 ± 1.4	1013.2	36.4 ± 0.7	41.1	41.3 ± 1.0	43.3	2.1 ± 1.2	4.5
100	1004.1 ± 1.0	1032.0 ± 0.9	1021.2	34.0 ± 0.8	38.0	39.8 ± 1.1	41.0	0.4 ± 1.3	1.8
120	1011.5 ± 0.6	1037.7 ± 0.6	1028.7	32.4 ± 0.7	35.3	38.6 ± 1.1	39.1	-1.2 ± 1.4	-0.5
140	1017.2 ± 0.7	1044.8 ± 0.5	1035.8	29.9 ± 1.0	33.0	36.4 ± 1.1	37.5	-3.8 ± 1.4	-2.6
160	1023.1 ± 1.3	1051.4 ± 0.6	1042.4	28.8 ± 0.8	31.1	35.7 ± 1.0	36.0	-4.7 ± 1.2	-4.4
180	1029.5 ± 1.2	1056.9 ± 0.5	1048.8	27.2 ± 0.9	29.4	34.5 ± 1.3	34.7	-6.2 ± 1.6	-6.0
200	1034.5 ± 0.7	1062.4 ± 0.7	1054.8	26.2 ± 0.7	27.9	33.8 ± 0.8	33.6	-7.1 ± 0.9	-7.5

Table S315: Heat capacities at constant volume computed from MC simulations (c_V^{MC}), heat capacities at constant volume obtained from REFPROP[10] (c_V^{REFP}), heat capacities at constant pressure computed from MC simulations (c_P^{MC}), heat capacities at constant pressure obtained from REFPROP[10] (c_P^{REFP}), speed of sound computed from MC simulations (c^{MC}), speed of sound obtained from REFPROP[10] (c^{REFP}), viscosities computed from MD simulations (η^{MD}), and viscosities obtained from REFPROP[10] (η^{REFP}) of CO_2 rich ternary mixture with 3% impurity of CH_4 and 1% impurity of Ar at 253 K and pressures ranging from 20 bar to 200 bar.

P/ [bar]	c_V^{MC}/ [J/mol K]	c_V^{REFP}/ [J/mol K]	c_P^{MC}/ [J/mol K]	c_P^{REFP}/ [J/mol K]	c^{MC}/ [m/s]	c^{REFP}/ [m/s]	η^{MD}/ [μPa s]	η^{REFP}/ [μPa s]
20	31.5 ± 0.1	33.1	51.0 ± 0.1	55.6	229.0 ± 0.6	223.9	11.5 ± 2.7	13.0
40	39.9 ± 0.3	40.2	95.5 ± 3.6	93.4	749.2 ± 29.4	685.0	137.5 ± 3.3	128.5
60	40.1 ± 0.1	40.2	95.4 ± 3.4	91.1	766.7 ± 24.5	708.4	139.7 ± 8.5	132.5
80	40.1 ± 0.2	40.2	90.8 ± 1.8	89.2	789.3 ± 11.1	729.8	146.3 ± 5.2	136.3
100	40.1 ± 0.2	40.2	90.2 ± 1.5	87.6	811.5 ± 12.2	749.7	161.7 ± 14.2	140.0
120	40.2 ± 0.2	40.2	89.1 ± 2.0	86.2	822.5 ± 12.9	768.2	146.5 ± 6.2	143.5
140	40.2 ± 0.3	40.2	87.2 ± 1.4	85.0	845.0 ± 16.1	785.6	159.1 ± 16.8	146.9
160	40.2 ± 0.2	40.2	86.7 ± 1.3	83.9	855.1 ± 13.6	802.1	160.3 ± 20.0	150.2
180	40.5 ± 0.2	40.3	86.0 ± 1.9	83.0	870.4 ± 17.9	817.8	163.5 ± 5.2	153.5
200	40.3 ± 0.3	40.3	85.7 ± 1.3	82.1	885.6 ± 13.7	832.7	169.4 ± 8.5	156.7

Table S316: Densities computed from MC and MD simulations (ρ^{MC} and ρ^{MD}), densities obtained from REFPROP[10] (ρ^{REFP}), isothermal compressibilities computed from MC simulations (β_T^{MC}), isothermal compressibilities obtained from REFPROP[10] (β_T^{REFP}), thermal expansion coefficients computed from MC simulations (α_P^{MC}), thermal expansion coefficients obtained from REFPROP[10] (α_P^{REFP}), Joule Thomson coefficients computed from MC simulations ($\mu_{\text{JT}}^{\text{MC}}$), and Joule Thomson coefficients obtained from REFPROP[10] ($\mu_{\text{JT}}^{\text{REFP}}$) of CO_2 rich ternary mixture with 3% impurity of CH_4 and 1% impurity of Ar at 273 K and pressures ranging from 20 bar to 200 bar.

P/ [bar]	ρ^{MC}/ [kg/m^3]	ρ^{MD}/ [kg/m^3]	ρ^{REFP}/ [kg/m^3]	β_T^{MC}/ [10^{-5}/bar]	β_T^{REFP}/ [10^{-5}/bar]	α_P^{MC}/ [10^{-4}/K]	α_P^{REFP}/ [10^{-4}/K]	$\mu_{\text{JT}}^{\text{MC}}$/ [$10^{-3}$ K/bar]	$\mu_{\text{JT}}^{\text{REFP}}$/ [$10^{-3}$ K/bar]
20	43.0	44.7 ± 0.1	44.3	5802.8 ± 48.8	5957.2	57.0 ± 0.6	60.7	1217.2 ± 34.0	1343.5
40	–	893.6 ± 2.3	298.9	–	−1502.3	–	−396.4	–	–
60	887.0 ± 0.7	910.9 ± 1.5	896.4	92.8 ± 6.4	104.7	72.1 ± 4.5	73.3	41.4 ± 5.5	44.7
80	902.1 ± 1.0	925.2 ± 1.7	913.4	73.8 ± 2.8	85.2	60.5 ± 1.6	63.6	30.0 ± 2.1	34.3
100	913.4 ± 1.0	939.2 ± 0.8	927.9	66.0 ± 2.0	72.4	56.7 ± 1.7	57.0	25.4 ± 2.1	26.7
120	925.7 ± 0.9	949.8 ± 1.1	940.5	56.7 ± 3.8	63.3	50.5 ± 3.2	52.0	18.2 ± 4.3	20.6
140	935.8 ± 0.4	960.9 ± 1.1	951.8	51.5 ± 0.5	56.3	47.7 ± 0.6	48.2	14.8 ± 0.9	15.7
160	944.5 ± 1.2	970.8 ± 0.8	962.0	48.0 ± 3.1	50.9	45.7 ± 2.8	45.1	12.1 ± 3.7	11.7
180	952.6 ± 0.9	978.9 ± 0.6	971.4	43.4 ± 0.9	46.5	42.4 ± 0.9	42.5	7.9 ± 1.2	8.2
200	960.9 ± 0.6	986.6 ± 0.6	980.1	40.7 ± 1.2	42.9	40.7 ± 1.0	40.3	5.6 ± 1.3	5.2

Table S317: Heat capacities at constant volume computed from MC simulations (c_V^{MC}), heat capacities at constant volume obtained from REFPROP[10] (c_V^{REFP}), heat capacities at constant pressure computed from MC simulations (c_P^{MC}), heat capacities at constant pressure obtained from REFPROP[10] (c_P^{REFP}), speed of sound computed from MC simulations (c^{MC}), speed of sound obtained from REFPROP[10] (c^{REFP}), viscosities computed from MD simulations (η^{MD}), and viscosities obtained from REFPROP[10] (η^{REFP}) of CO_2 rich ternary mixture with 3% impurity of CH_4 and 1% impurity of Ar at 273 K and pressures ranging from 20 bar to 200 bar.

P/ [bar]	c_V^{MC}/ [J/mol K]	c_V^{REFP}/ [J/mol K]	c_P^{MC}/ [J/mol K]	c_P^{REFP}/ [J/mol K]	c^{MC}/ [m/s]	c^{REFP}/ [m/s]	η^{MD}/ [μPa s]	η^{REFP}/ [μPa s]
20	30.7	31.1	45.8 ± 0.2	47.5	244.7 ± 1.1	240.4	14.5 ± 4.1	14.0
40	–	–	–	–	–	–	97.9 ± 3.3	21.1
60	39.6 ± 0.4	40.4	113.7 ± 4.5	107.8	591.1 ± 23.8	533.4	110.0 ± 16.1	94.3
80	39.8 ± 0.2	40.1	103.8 ± 1.8	101.4	626.1 ± 13.1	569.6	105.2 ± 1.7	99.0
100	39.5 ± 0.3	40.0	101.9 ± 1.7	96.9	653.8 ± 11.4	600.5	107.9 ± 2.7	103.3
120	39.6 ± 0.1	39.9	96.5 ± 3.5	93.5	681.7 ± 26.2	627.7	115.5 ± 7.7	107.2
140	39.6 ± 0.1	39.8	94.7 ± 1.3	90.8	703.9 ± 6.1	652.2	121.6 ± 6.8	110.8
160	39.6 ± 0.2	39.8	93.4 ± 3.1	88.7	721.3 ± 26.3	674.6	130.8 ± 11.8	114.3
180	39.5 ± 0.2	39.8	90.3 ± 1.3	86.9	743.1 ± 9.7	695.3	124.8 ± 4.1	117.6
200	39.5 ± 0.1	39.8	88.9 ± 0.9	85.4	758.9 ± 11.8	714.6	129.0 ± 5.4	120.7

Table S318: Densities computed from MC and MD simulations (ρ^{MC} and ρ^{MD}), densities obtained from REFPROP[10] (ρ^{REFP}), isothermal compressibilities computed from MC simulations (β_T^{MC}), isothermal compressibilities obtained from REFPROP[10] (β_T^{REFP}), thermal expansion coefficients computed from MC simulations (α_P^{MC}), thermal expansion coefficients obtained from REFPROP[10] (α_P^{REFP}), Joule Thomson coefficients computed from MC simulations ($\mu_{\mathrm{JT}}^{\mathrm{MC}}$), and Joule Thomson coefficients obtained from REFPROP[10] ($\mu_{\mathrm{JT}}^{\mathrm{REFP}}$) of CO_2 rich ternary mixture with 3% impurity of CH_4 and 1% impurity of Ar at 293 K and pressures ranging from 20 bar to 200 bar.

P/ [bar]	ρ^{MC}/ [kg/m^3]	ρ^{MD}/ [kg/m^3]	ρ^{REFP}/ [kg/m^3]	β_T^{MC}/ [10^{-5}/bar]	β_T^{REFP}/ [10^{-5}/bar]	α_P^{MC}/ [10^{-4}/K]	α_P^{REFP}/ [10^{-4}/K]	$\mu_{\mathrm{JT}}^{\mathrm{MC}}$/ [$10^{-3}$ K/bar]	$\mu_{\mathrm{JT}}^{\mathrm{REFP}}$/ [$10^{-3}$ K/bar]
20	38.8	40.2 ± 0.1	39.7	5564.1 ± 15.7	5669.0	47.6 ± 0.1	49.8	1002.9 ± 11.2	1110.8
40	89.5 ± 0.1	93.3 ± 0.2	93.7	3370.5 ± 34.9	3534.5	76.9 ± 1.0	85.5	1040.9 ± 25.9	1107.8
60	–	219.2 ± 33.9	195.3	–	4768.0	–	303.5	–	1026.2
80	753.6 ± 1.5	775.9 ± 3.8	756.6	275.8 ± 7.1	363.4	133.2 ± 3.0	156.0	111.9 ± 3.8	132.0
100	791.6 ± 2.3	809.4 ± 2.4	798.3	173.3 ± 17.0	205.9	96.6 ± 7.8	103.8	79.5 ± 10.7	89.1
120	813.5 ± 2.0	836.6 ± 0.4	826.4	131.0 ± 7.4	147.2	78.9 ± 3.7	82.3	61.4 ± 5.3	66.6
140	835.4 ± 2.1	857.9 ± 1.2	848.2	104.2 ± 3.8	115.7	68.1 ± 1.7	69.9	48.4 ± 2.5	51.8
160	851.0 ± 2.2	873.5 ± 1.0	866.2	86.0 ± 2.3	95.8	59.9 ± 1.4	61.7	38.0 ± 2.1	41.1
180	864.7 ± 1.1	887.9 ± 1.0	881.7	73.9 ± 3.0	82.0	53.5 ± 2.0	55.7	29.7 ± 3.2	32.9
200	877.7 ± 1.1	900.5 ± 0.9	895.3	66.4 ± 2.2	71.9	50.4 ± 1.5	51.1	25.0 ± 2.4	26.4

Table S319: Heat capacities at constant volume computed from MC simulations (c_V^{MC}), heat capacities at constant volume obtained from REFPROP[10] (c_V^{REFP}), heat capacities at constant pressure computed from MC simulations (c_P^{MC}), heat capacities at constant pressure obtained from REFPROP[10] (c_P^{REFP}), speed of sound computed from MC simulations (c^{MC}), speed of sound obtained from REFPROP[10] (c^{REFP}), viscosities computed from MD simulations (η^{MD}), and viscosities obtained from REFPROP[10] (η^{REFP}) of CO_2 rich ternary mixture with 3% impurity of CH_4 and 1% impurity of Ar at 293 K and pressures ranging from 20 bar to 200 bar.

P/ [bar]	c_V^{MC}/ [J/mol K]	c_V^{REFP}/ [J/mol K]	c_P^{MC}/ [J/mol K]	c_P^{REFP}/ [J/mol K]	c^{MC}/ [m/s]	c^{REFP}/ [m/s]	η^{MD}/ [μPa s]	η^{REFP}/ [μPa s]
20	30.7	30.9	43.8 ± 0.1	44.8	257.4 ± 0.4	253.7	7.2 ± 0.6	15.0
40	33.6	34.6	58.1 ± 0.4	62.5	239.6 ± 1.5	233.5	16.5 ± 1.9	15.7
60	–	44.8	–	169.8	–	201.7	17.5 ± 0.8	18.0
80	40.1 ± 0.4	42.3	148.4 ± 2.2	154.2	421.8 ± 6.7	364.0	68.3 ± 0.6	65.1
100	39.6 ± 0.2	40.9	125.5 ± 5.9	123.8	480.9 ± 26.3	428.9	82.6 ± 6.0	72.4
120	39.6 ± 0.2	40.3	113.2 ± 3.2	110.7	518.2 ± 16.4	475.1	80.1 ± 2.2	77.9
140	39.3 ± 0.1	40.0	106.3 ± 1.4	102.9	557.6 ± 10.8	512.3	84.1 ± 2.4	82.5
160	39.2 ± 0.2	39.8	100.8 ± 1.3	97.7	592.7 ± 9.0	544.1	93.9 ± 10.6	86.6
180	39.2 ± 0.2	39.7	95.4 ± 2.1	93.8	617.0 ± 14.4	572.1	97.6 ± 4.2	90.3
200	39.3 ± 0.1	39.6	93.9 ± 1.6	90.9	639.8 ± 12.0	597.4	101.5 ± 10.5	93.7

Table S320: Densities computed from MC and MD simulations (ρ^{MC} and ρ^{MD}), densities obtained from REFPROP[10] (ρ^{REFP}), isothermal compressibilities computed from MC simulations (β_T^{MC}), isothermal compressibilities obtained from REFPROP[10] (β_T^{REFP}), thermal expansion coefficients computed from MC simulations (α_P^{MC}), thermal expansion coefficients obtained from REFPROP[10] (α_P^{REFP}), Joule Thomson coefficients computed from MC simulations (μ_{JT}^{MC}), and Joule Thomson coefficients obtained from REFPROP[10] (μ_{JT}^{REFP}) of CO_2 rich ternary mixture with 3% impurity of CH_4 and 1% impurity of Ar at 313 K and pressures ranging from 20 bar to 200 bar.

P/ [bar]	ρ^{MC}/ [kg/m^3]	ρ^{MD}/ [kg/m^3]	ρ^{REFP}/ [kg/m^3]	β_T^{MC}/ [10^{-5}/bar]	β_T^{REFP}/ [10^{-5}/bar]	α_P^{MC}/ [10^{-4}/K]	α_P^{REFP}/ [10^{-4}/K]	μ_{JT}^{MC}/ [10^{-3} K/bar]	μ_{JT}^{REFP}/ [10^{-3} K/bar]
20	35.4	36.7 ± 0.1	36.2	5424.8 ± 33.5	5495.9	41.6 ± 0.3	42.9	851.0 ± 22.3	935.4
40	78.4 ± 0.1	81.3 ± 0.3	81.1	3080.1 ± 36.5	3145.2	58.2 ± 0.7	61.8	878.2 ± 25.4	926.9
60	134.3 ± 0.2	140.2 ± 0.6	142.0	2499.8 ± 40.7	2602.9	92.3 ± 1.2	101.8	875.3 ± 18.5	893.5
80	222.3 ± 0.4	238.5 ± 0.6	243.7	2742.7 ± 121.4	3006.7	195.7 ± 8.7	235.8	803.2 ± 48.5	783.2
100	468.9 ± 10.1	478.3 ± 8.8	494.2	3417.6 ± 380.8	2734.2	649.8 ± 66.4	528.4	439.7 ± 64.1	416.5
120	629.8 ± 7.4	650.2 ± 3.8	643.2	671.0 ± 91.1	649.0	216.2 ± 22.0	199.3	206.8 ± 28.2	205.4
140	694.8 ± 2.5	709.4 ± 2.1	704.6	323.9 ± 15.9	330.6	130.7 ± 5.2	125.0	135.0 ± 7.8	135.5
160	728.9 ± 3.1	747.2 ± 0.7	743.4	209.8 ± 17.3	220.7	96.9 ± 7.1	95.3	98.7 ± 11.7	100.0
180	757.9 ± 1.0	776.5 ± 1.5	772.3	159.3 ± 14.0	165.8	81.1 ± 6.5	78.9	77.8 ± 11.1	77.8
200	779.3 ± 1.1	798.7 ± 1.1	795.5	128.0 ± 5.4	132.8	69.9 ± 3.0	68.3	62.8 ± 5.2	62.2

Table S321: Heat capacities at constant volume computed from MC simulations (c_V^{MC}), heat capacities at constant volume obtained from REFPROP[10] (c_V^{REFP}), heat capacities at constant pressure computed from MC simulations (c_P^{MC}), heat capacities at constant pressure obtained from REFPROP[10] (c_P^{REFP}), speed of sound computed from MC simulations (c^{MC}), speed of sound obtained from REFPROP[10] (c^{REFP}), viscosities computed from MD simulations (η^{MD}), and viscosities obtained from REFPROP[10] (η^{REFP}) of CO_2 rich ternary mixture with 3% impurity of CH_4 and 1% impurity of Ar at 313 K and pressures ranging from 20 bar to 200 bar.

P/ [bar]	c_V^{MC}/ [J/mol K]	c_V^{REFP}/ [J/mol K]	c_P^{MC}/ [J/mol K]	c_P^{REFP}/ [J/mol K]	c^{MC}/ [m/s]	c^{REFP}/ [m/s]	η^{MD}/ [μPa s]	η^{REFP}/ [μPa s]
20	31.0	31.1	43.0 ± 0.1	43.5	268.6 ± 0.9	265.2	5.2 ± 8.6	15.9
40	32.8	33.3	51.6 ± 0.2	53.5	255.2 ± 1.6	250.8	16.3 ± 0.7	16.5
60	35.3 ± 0.1	36.5	69.3 ± 0.3	74.3	241.7 ± 2.1	234.8	17.6 ± 1.4	17.7
80	39.0 ± 0.1	41.8	123.8 ± 3.6	144.2	228.2 ± 6.1	217.1	22.9 ± 3.1	20.7
100	42.8 ± 0.3	46.6	405.0 ± 39.0	325.5	243.1 ± 18.1	227.3	31.6 ± 3.7	35.2
120	40.6 ± 0.4	42.5	191.1 ± 12.4	171.0	333.6 ± 25.2	310.3	52.5 ± 6.4	50.0
140	39.8 ± 0.2	41.0	142.1 ± 3.3	131.5	398.1 ± 10.9	371.0	57.9 ± 1.6	57.9
160	39.4 ± 0.2	40.3	121.8 ± 5.7	115.0	449.5 ± 21.4	416.9	66.6 ± 5.0	63.6
180	39.2 ± 0.1	39.9	112.6 ± 5.7	105.6	487.6 ± 24.7	454.6	70.5 ± 3.0	68.3
200	39.0 ± 0.1	39.7	104.7 ± 2.9	99.4	518.6 ± 13.1	486.9	71.4 ± 2.6	72.4

S16.20 Data of thermodynamics and transport properties of CO_2 rich ternary mixture with 3 mole% impurity of Ar and 1 mole% impurity of CH_4

Table S322: Densities computed from MC and MD simulations (ρ^{MC} and ρ^{MD}), densities obtained from REFPROP[10] (ρ^{REFP}), isothermal compressibilities computed from MC simulations (β_T^{MC}), isothermal compressibilities obtained from REFPROP[10] (β_T^{REFP}), thermal expansion coefficients computed from MC simulations (α_P^{MC}), thermal expansion coefficients obtained from REFPROP[10] (α_P^{REFP}), Joule Thomson coefficients computed from MC simulations ($\mu_{\mathrm{JT}}^{\mathrm{MC}}$), and Joule Thomson coefficients obtained from REFPROP[10] ($\mu_{\mathrm{JT}}^{\mathrm{REFP}}$) of CO_2 rich ternary mixture with 3% impurity of Ar and 1% impurity of CH_4 at 253 K and pressures ranging from 20 bar to 200 bar.

P/ [bar]	ρ^{MC}/ [kg/m^3]	ρ^{MD}/ [kg/m^3]	ρ^{REFP}/ [kg/m^3]	β_T^{MC}/ [10^{-5}/bar]	β_T^{REFP}/ [10^{-5}/bar]	α_P^{MC}/ [10^{-4}/K]	α_P^{REFP}/ [10^{-4}/K]	$\mu_{\mathrm{JT}}^{\mathrm{MC}}$/ [$10^{-3}$ K/bar]	$\mu_{\mathrm{JT}}^{\mathrm{REFP}}$/ [$10^{-3}$ K/bar]
20	49.4	50.7 ± 0.2	51.4	6279.0 ± 57.1	6510.5	73.9 ± 0.7	81.4	1522.8 ± 30.8	1653.3
40	994.7 ± 1.0	1007.4 ± 1.8	1010.0	45.0 ± 2.8	49.8	47.8 ± 3.0	49.4	9.6 ± 3.5	11.5
60	1004.5 ± 0.5	1016.3 ± 0.8	1019.6	39.8 ± 1.7	45.0	43.8 ± 1.6	46.2	5.1 ± 1.9	7.9
80	1012.7 ± 0.9	1025.3 ± 1.4	1028.4	38.7 ± 2.9	41.2	43.8 ± 3.2	43.5	5.0 ± 3.8	4.8
100	1020.0 ± 0.9	1032.0 ± 0.9	1036.6	35.7 ± 2.0	38.0	41.7 ± 2.3	41.3	2.6 ± 2.7	2.1
120	1026.3 ± 0.9	1037.7 ± 0.6	1044.2	32.7 ± 2.8	35.4	38.6 ± 3.1	39.3	-1.2 ± 3.7	-0.2
140	1034.0 ± 0.7	1044.8 ± 0.5	1051.4	31.2 ± 1.1	33.1	37.8 ± 1.4	37.7	-2.1 ± 1.7	-2.3
160	1040.1 ± 1.1	1051.4 ± 0.6	1058.2	28.2 ± 0.5	31.1	34.9 ± 0.9	36.2	-5.8 ± 1.2	-4.1
180	1045.0 ± 1.2	1056.9 ± 0.5	1064.6	27.0 ± 0.3	29.4	33.8 ± 0.3	34.9	-7.1 ± 0.4	-5.8
200	1051.5 ± 0.1	1062.4 ± 0.7	1070.7	27.0 ± 1.0	27.9	35.1 ± 1.5	33.7	-5.3 ± 1.8	-7.3

Table S323: Heat capacities at constant volume computed from MC simulations (c_V^{MC}), heat capacities at constant volume obtained from REFPROP[10] (c_V^{REFP}), heat capacities at constant pressure computed from MC simulations (c_P^{MC}), heat capacities at constant pressure obtained from REFPROP[10] (c_P^{REFP}), speed of sound computed from MC simulations (c^{MC}), speed of sound obtained from REFPROP[10] (c^{REFP}), viscosities computed from MD simulations (η^{MD}), and viscosities obtained from REFPROP[10] (η^{REFP}) of CO_2 rich ternary mixture with 3% impurity of Ar and 1% impurity of CH_4 at 253 K and pressures ranging from 20 bar to 200 bar.

P/ [bar]	c_V^{MC}/ [J/mol K]	c_V^{REFP}/ [J/mol K]	c_P^{MC}/ [J/mol K]	c_P^{REFP}/ [J/mol K]	c^{MC}/ [m/s]	c^{REFP}/ [m/s]	η^{MD}/ [μPa s]	η^{REFP}/ [μPa s]
20	31.2 ± 0.1	32.6	50.4 ± 0.1	54.4	228.2 ± 1.1	223.6	11.5 ± 2.7	13.1
40	39.9 ± 0.3	40.0	95.5 ± 3.7	93.6	731.1 ± 26.9	681.8	137.5 ± 3.3	129.8
60	39.8 ± 0.2	40.0	92.2 ± 1.7	91.2	760.9 ± 17.5	704.9	139.7 ± 8.5	133.8
80	40.0 ± 0.2	40.0	93.4 ± 4.1	89.3	771.4 ± 33.1	726.1	146.3 ± 5.2	137.6
100	39.8 ± 0.1	40.0	92.0 ± 2.8	87.7	796.5 ± 25.3	745.8	161.7 ± 14.2	141.3
120	39.8 ± 0.2	40.0	88.2 ± 3.7	86.3	813.0 ± 38.8	764.2	146.5 ± 6.2	144.9
140	40.1 ± 0.1	40.0	88.2 ± 2.1	85.0	826.3 ± 17.4	781.4	159.1 ± 16.8	148.3
160	40.0 ± 0.3	40.1	85.3 ± 1.7	84.0	852.6 ± 12.0	797.8	160.3 ± 20.0	151.7
180	40.2 ± 0.2	40.1	84.2 ± 0.4	83.0	862.1 ± 5.1	813.3	163.5 ± 5.2	155.0
200	40.1 ± 0.3	40.1	87.6 ± 2.4	82.2	876.6 ± 19.9	828.1	169.4 ± 8.5	158.2

Table S324: Densities computed from MC and MD simulations (ρ^{MC} and ρ^{MD}), densities obtained from REFPROP[10] (ρ^{REFP}), isothermal compressibilities computed from MC simulations (β_T^{MC}), isothermal compressibilities obtained from REFPROP[10] (β_T^{REFP}), thermal expansion coefficients computed from MC simulations (α_P^{MC}), thermal expansion coefficients obtained from REFPROP[10] (α_P^{REFP}), Joule Thomson coefficients computed from MC simulations (μ_{JT}^{MC}), and Joule Thomson coefficients obtained from REFPROP[10] (μ_{JT}^{REFP}) of CO_2 rich ternary mixture with 3% impurity of Ar and 1% impurity of CH_4 at 273 K and pressures ranging from 20 bar to 200 bar.

P/ [bar]	ρ^{MC}/ [kg/m^3]	ρ^{MD}/ [kg/m^3]	ρ^{REFP}/ [kg/m^3]	β_T^{MC}/ [10^{-5}/bar]	β_T^{REFP}/ [10^{-5}/bar]	α_P^{MC}/ [10^{-4}/K]	α_P^{REFP}/ [10^{-4}/K]	μ_{JT}^{MC}/ [10^{-3} K/bar]	μ_{JT}^{REFP}/ [10^{-3} K/bar]
20	43.4	44.7 ± 0.1	44.7	5834.0 ± 34.9	5938.1	57.3 ± 0.4	60.1	1239.9 ± 25.9	1333.1
40	–	893.6 ± 2.3	272.0	–	−2311.4	–	−427.5	–	–
60	898.6 ± 1.5	910.9 ± 1.5	909.1	88.1 ± 8.4	105.4	67.8 ± 5.5	74.1	38.1 ± 6.9	45.3
80	913.6 ± 1.4	925.2 ± 1.7	926.5	77.7 ± 3.8	85.7	62.7 ± 2.2	64.2	32.4 ± 2.7	34.9
100	926.3 ± 1.0	939.2 ± 0.8	941.2	66.4 ± 3.8	72.8	56.6 ± 2.6	57.5	25.4 ± 3.4	27.2
120	937.4 ± 1.1	949.8 ± 1.1	954.1	60.1 ± 2.8	63.6	53.5 ± 2.6	52.5	21.5 ± 3.4	21.1
140	948.2 ± 2.3	960.9 ± 1.1	965.6	55.7 ± 2.0	56.6	50.2 ± 1.2	48.6	17.8 ± 1.5	16.2
160	958.4 ± 1.1	970.8 ± 0.8	976.0	48.6 ± 1.1	51.1	46.0 ± 0.9	45.4	12.5 ± 1.3	12.1
180	967.3 ± 1.5	978.9 ± 0.6	985.6	43.8 ± 1.8	46.7	42.6 ± 1.7	42.8	8.2 ± 2.3	8.6
200	975.1 ± 1.0	986.6 ± 0.6	994.5	40.0 ± 1.9	43.0	39.8 ± 1.8	40.6	4.4 ± 2.6	5.6

Table S325: Heat capacities at constant volume computed from MC simulations (c_V^{MC}), heat capacities at constant volume obtained from REFPROP[10] (c_V^{REFP}), heat capacities at constant pressure computed from MC simulations (c_P^{MC}), heat capacities at constant pressure obtained from REFPROP[10] (c_P^{REFP}), speed of sound computed from MC simulations (c^{MC}), speed of sound obtained from REFPROP[10] (c^{REFP}), viscosities computed from MD simulations (η^{MD}), and viscosities obtained from REFPROP[10] (η^{REFP}) of CO_2 rich ternary mixture with 3% impurity of Ar and 1% impurity of CH_4 at 273 K and pressures ranging from 20 bar to 200 bar.

P/ [bar]	c_V^{MC}/ [J/mol K]	c_V^{REFP}/ [J/mol K]	c_P^{MC}/ [J/mol K]	c_P^{REFP}/ [J/mol K]	c^{MC}/ [m/s]	c^{REFP}/ [m/s]	η^{MD}/ [μPa s]	η^{REFP}/ [μPa s]
20	30.4	30.7	45.6 ± 0.2	46.9	243.3 ± 0.8	239.8	14.5 ± 4.1	14.1
40	–	–	–	–	–	–	97.9 ± 3.3	19.8
60	39.3 ± 0.2	40.1	108.3 ± 4.9	108.3	590.1 ± 31.2	530.7	110.0 ± 16.1	95.1
80	39.4 ± 0.1	39.9	104.6 ± 1.8	101.7	611.6 ± 15.8	566.7	105.2 ± 1.7	99.8
100	39.2 ± 0.2	39.7	101.0 ± 2.5	97.1	647.2 ± 20.3	597.4	107.9 ± 2.7	104.1
120	39.2 ± 0.1	39.6	99.2 ± 3.2	93.6	670.0 ± 19.0	624.4	115.5 ± 7.7	108.1
140	39.3 ± 0.1	39.6	95.7 ± 1.1	91.0	678.8 ± 12.7	648.7	121.6 ± 6.8	111.8
160	39.1 ± 0.2	39.5	92.7 ± 1.1	88.8	712.8 ± 9.3	670.9	130.8 ± 11.8	115.2
180	39.3 ± 0.1	39.5	89.7 ± 2.1	87.0	733.7 ± 17.5	691.5	124.8 ± 4.1	118.6
200	39.2 ± 0.2	39.5	87.3 ± 2.1	85.4	755.5 ± 20.3	710.7	129.0 ± 5.4	121.8

Table S326: Densities computed from MC and MD simulations (ρ^{MC} and ρ^{MD}), densities obtained from REFPROP[10] (ρ^{REFP}), isothermal compressibilities computed from MC simulations (β_T^{MC}), isothermal compressibilities obtained from REFPROP[10] (β_T^{REFP}), thermal expansion coefficients computed from MC simulations (α_P^{MC}), thermal expansion coefficients obtained from REFPROP[10] (α_P^{REFP}), Joule Thomson coefficients computed from MC simulations ($\mu_{\mathrm{JT}}^{\mathrm{MC}}$), and Joule Thomson coefficients obtained from REFPROP[10] ($\mu_{\mathrm{JT}}^{\mathrm{REFP}}$) of CO_2 rich ternary mixture with 3% impurity of Ar and 1% impurity of CH_4 at 293 K and pressures ranging from 20 bar to 200 bar.

P/ [bar]	ρ^{MC}/ [kg/m^3]	ρ^{MD}/ [kg/m^3]	ρ^{REFP}/ [kg/m^3]	β_T^{MC}/ [10^{-5}/bar]	β_T^{REFP}/ [10^{-5}/bar]	α_P^{MC}/ [10^{-4}/K]	α_P^{REFP}/ [10^{-4}/K]	$\mu_{\mathrm{JT}}^{\mathrm{MC}}$/ [$10^{-3}$ K/bar]	$\mu_{\mathrm{JT}}^{\mathrm{REFP}}$/ [$10^{-3}$ K/bar]
20	39.1	40.2 ± 0.1	40.1	5595.4 ± 44.9	5658.2	47.8 ± 0.4	49.5	1023.4 ± 28.7	1104.2
40	90.0 ± 0.1	93.3 ± 0.2	94.3	3346.4 ± 36.9	3506.6	75.7 ± 0.8	83.9	1030.4 ± 21.7	1100.5
60	–	219.2 ± 33.9	193.1	–	4452.3	–	272.7	–	1026.3
80	755.7 ± 1.5	775.9 ± 3.8	764.7	319.3 ± 32.1	377.5	147.6 ± 12.4	161.4	123.3 ± 14.9	135.2
100	794.4 ± 4.1	809.4 ± 2.4	808.1	189.0 ± 27.4	210.3	102.1 ± 11.5	105.9	85.3 ± 15.3	90.8
120	821.3 ± 4.3	836.6 ± 0.4	837.1	140.3 ± 18.9	149.4	83.3 ± 8.9	83.6	65.9 ± 12.5	67.8
140	844.6 ± 1.1	857.9 ± 1.2	859.5	111.0 ± 1.4	117.1	72.0 ± 1.4	70.8	52.5 ± 2.1	52.8
160	860.5 ± 2.6	873.5 ± 1.0	877.9	97.1 ± 4.9	96.8	66.4 ± 2.7	62.4	45.2 ± 3.9	42.0
180	877.4 ± 2.2	887.9 ± 1.0	893.8	76.0 ± 3.1	82.7	55.2 ± 2.1	56.3	31.7 ± 3.2	33.7
200	888.7 ± 2.1	900.5 ± 0.9	907.7	72.0 ± 4.0	72.4	54.0 ± 2.4	51.6	29.5 ± 3.7	27.0

Table S327: Heat capacities at constant volume computed from MC simulations (c_V^{MC}), heat capacities at constant volume obtained from REFPROP[10] (c_V^{REFP}), heat capacities at constant pressure computed from MC simulations (c_P^{MC}), heat capacities at constant pressure obtained from REFPROP[10] (c_P^{REFP}), speed of sound computed from MC simulations (c^{MC}), speed of sound obtained from REFPROP[10] (c^{REFP}), viscosities computed from MD simulations (η^{MD}), and viscosities obtained from REFPROP[10] (η^{REFP}) of CO_2 rich ternary mixture with 3% impurity of Ar and 1% impurity of CH_4 at 293 K and pressures ranging from 20 bar to 200 bar.

P/ [bar]	c_V^{MC}/ [J/mol K]	c_V^{REFP}/ [J/mol K]	c_P^{MC}/ [J/mol K]	c_P^{REFP}/ [J/mol K]	c^{MC}/ [m/s]	c^{REFP}/ [m/s]	η^{MD}/ [μPa s]	η^{REFP}/ [μPa s]
20	30.4	30.5	43.6 ± 0.1	44.3	255.8 ± 1.1	252.9	7.2 ± 0.6	15.1
40	33.3	34.1	57.3 ± 0.3	61.3	239.0 ± 1.5	233.2	16.5 ± 1.9	15.8
60	–	43.3	–	153.8	–	203.3	17.5 ± 0.8	18.0
80	39.8 ± 0.4	42.0	155.6 ± 8.0	157.3	402.3 ± 22.8	360.1	68.3 ± 0.6	65.3
100	39.2 ± 0.2	40.6	128.3 ± 8.1	124.9	466.9 ± 37.0	425.5	82.6 ± 6.0	72.8
120	39.2 ± 0.2	40.0	116.2 ± 6.8	111.3	507.1 ± 37.3	471.8	80.1 ± 2.2	78.4
140	38.9 ± 0.2	39.6	109.0 ± 1.8	103.3	546.4 ± 5.9	509.0	84.1 ± 2.4	83.1
160	38.9 ± 0.1	39.5	106.0 ± 2.4	98.0	571.1 ± 15.8	540.7	93.9 ± 10.6	87.2
180	38.8 ± 0.1	39.3	96.8 ± 2.2	94.0	611.2 ± 14.2	568.6	97.6 ± 4.2	90.9
200	38.9 ± 0.2	39.3	96.7 ± 2.3	91.0	623.0 ± 18.8	593.7	101.5 ± 10.5	94.4

Table S328: Densities computed from MC and MD simulations (ρ^{MC} and ρ^{MD}), densities obtained from REFPROP[10] (ρ^{REFP}), isothermal compressibilities computed from MC simulations (β_T^{MC}), isothermal compressibilities obtained from REFPROP[10] (β_T^{REFP}), thermal expansion coefficients computed from MC simulations (α_P^{MC}), thermal expansion coefficients obtained from REFPROP[10] (α_P^{REFP}), Joule Thomson coefficients computed from MC simulations ($\mu_{\mathrm{JT}}^{\mathrm{MC}}$), and Joule Thomson coefficients obtained from REFPROP[10] ($\mu_{\mathrm{JT}}^{\mathrm{REFP}}$) of CO_2 rich ternary mixture with 3% impurity of Ar and 1% impurity of CH_4 at 313 K and pressures ranging from 20 bar to 200 bar.

P/ [bar]	ρ^{MC}/ [kg/m^3]	ρ^{MD}/ [kg/m^3]	ρ^{REFP}/ [kg/m^3]	β_T^{MC}/ [10^{-5}/bar]	β_T^{REFP}/ [10^{-5}/bar]	α_P^{MC}/ [10^{-4}/K]	α_P^{REFP}/ [10^{-4}/K]	$\mu_{\mathrm{JT}}^{\mathrm{MC}}$/ [$10^{-3}$ K/bar]	$\mu_{\mathrm{JT}}^{\mathrm{REFP}}$/ [$10^{-3}$ K/bar]
20	35.8	36.7 ± 0.1	36.6	5430.3 ± 28.8	5488.8	41.5 ± 0.2	42.7	853.8 ± 18.1	931.1
40	78.9 ± 0.1	81.3 ± 0.3	81.8	3043.2 ± 23.1	3132.7	57.3 ± 0.4	61.1	861.1 ± 12.8	921.9
60	134.8 ± 0.2	140.2 ± 0.6	142.6	2426.6 ± 40.9	2575.7	88.1 ± 1.7	99.5	849.2 ± 27.8	889.4
80	219.6 ± 1.0	238.5 ± 0.6	242.2	2513.7 ± 128.7	2907.8	172.6 ± 8.9	222.1	782.8 ± 57.8	785.4
100	421.8 ± 6.4	478.3 ± 8.8	485.2	3649.0 ± 441.5	2985.2	609.5 ± 76.1	553.2	498.4 ± 85.8	438.4
120	623.8 ± 9.7	650.2 ± 3.8	646.7	801.9 ± 66.8	687.1	243.5 ± 15.3	208.3	227.6 ± 19.4	212.1
140	691.8 ± 1.5	709.4 ± 2.1	711.4	358.2 ± 23.6	341.5	138.7 ± 9.1	128.3	144.8 ± 14.2	138.9
160	732.6 ± 2.2	747.2 ± 0.7	751.7	235.6 ± 20.5	225.8	105.1 ± 8.5	97.1	107.7 ± 13.9	102.2
180	765.0 ± 1.5	776.5 ± 1.5	781.6	168.8 ± 11.9	168.7	84.3 ± 5.4	80.2	82.3 ± 9.0	79.4
200	784.9 ± 1.9	798.7 ± 1.1	805.4	137.4 ± 6.2	134.8	73.6 ± 2.9	69.3	67.7 ± 5.0	63.5

Table S329: Heat capacities at constant volume computed from MC simulations (c_V^{MC}), heat capacities at constant volume obtained from REFPROP[10] (c_V^{REFP}), heat capacities at constant pressure computed from MC simulations (c_P^{MC}), heat capacities at constant pressure obtained from REFPROP[10] (c_P^{REFP}), speed of sound computed from MC simulations (c^{MC}), speed of sound obtained from REFPROP[10] (c^{REFP}), viscosities computed from MD simulations (η^{MD}), and viscosities obtained from REFPROP[10] (η^{REFP}) of CO_2 rich ternary mixture with 3% impurity of Ar and 1% impurity of CH_4 at 313 K and pressures ranging from 20 bar to 200 bar.

P/ [bar]	c_V^{MC}/ [J/mol K]	c_V^{REFP}/ [J/mol K]	c_P^{MC}/ [J/mol K]	c_P^{REFP}/ [J/mol K]	c^{MC}/ [m/s]	c^{REFP}/ [m/s]	η^{MD}/ [μPa s]	η^{REFP}/ [μPa s]
20	30.7	30.7	42.7 ± 0.1	43.1	267.4 ± 0.7	264.3	5.2 ± 8.6	16.1
40	32.5	32.9	50.9 ± 0.1	52.8	255.4 ± 1.0	250.3	16.3 ± 0.7	16.7
60	34.9	35.9	67.0 ± 0.8	72.7	242.2 ± 2.5	234.7	17.6 ± 1.4	17.9
80	38.2 ± 0.1	40.8	111.7 ± 4.2	136.4	230.2 ± 7.4	217.8	22.9 ± 3.1	20.8
100	42.5 ± 0.6	46.1	375.4 ± 41.2	334.5	239.6 ± 19.7	223.8	31.6 ± 3.7	34.3
120	40.4 ± 0.3	42.2	203.5 ± 8.7	175.5	317.6 ± 15.1	306.0	52.5 ± 6.4	49.8
140	39.5 ± 0.2	40.6	145.5 ± 7.0	133.1	385.6 ± 15.7	367.1	57.9 ± 1.6	58.0
160	39.2 ± 0.1	39.9	126.5 ± 7.0	115.8	432.6 ± 22.3	413.3	66.6 ± 5.0	63.8
180	38.7 ± 0.1	39.5	113.6 ± 4.4	106.0	476.5 ± 19.3	451.0	70.5 ± 3.0	68.6
200	38.8 ± 0.3	39.3	106.9 ± 2.4	99.7	505.3 ± 12.8	483.3	71.4 ± 2.6	72.8

S16.21 Data of thermodynamics and transport properties of CO_2 rich ternary mixture with 1 mole% impurity of Ar and 3 mole% impurity of H_2

Table S330: Densities computed from MC and MD simulations (ρ^{MC} and ρ^{MD}), densities obtained from REFPROP[10] (ρ^{REFP}), isothermal compressibilities computed from MC simulations (β_T^{MC}), isothermal compressibilities obtained from REFPROP[10] (β_T^{REFP}), thermal expansion coefficients computed from MC simulations (α_P^{MC}), thermal expansion coefficients obtained from REFPROP[10] (α_P^{REFP}), Joule Thomson coefficients computed from MC simulations (μ_{JT}^{MC}), and Joule Thomson coefficients obtained from REFPROP[10] (μ_{JT}^{REFP}) of CO_2 rich ternary mixture with 3% impurity of H_2 and 1% impurity of Ar at 253 K and pressures ranging from 20 bar to 200 bar.

P/ [bar]	ρ^{MC}/ [kg/m^3]	ρ^{MD}/ [kg/m^3]	ρ^{REFP}/ [kg/m^3]	β_T^{MC}/ [10^{-5}/bar]	β_T^{REFP}/ [10^{-5}/bar]	α_P^{MC}/ [10^{-4}/K]	α_P^{REFP}/ [10^{-4}/K]	μ_{JT}^{MC}/ [10^{-3} K/bar]	μ_{JT}^{REFP}/ [10^{-3} K/bar]
20	47.9	50.5 ± 0.1	50.0	6245.8 ± 68.4	6442.2	72.4 ± 0.9	79.2	1485.7 ± 40.5	1612.2
40	966.1 ± 2.1	1004.3 ± 0.7	640.7	53.9 ± 2.5	-29.6	53.5 ± 2.2	85.4	15.7 ± 2.5	–
60	976.3 ± 0.8	1012.8 ± 0.7	882.9	50.3 ± 1.3	220.9	51.4 ± 1.8	142.9	13.4 ± 2.1	–
80	985.1 ± 1.3	1020.2 ± 1.0	991.6	44.3 ± 2.7	44.6	47.0 ± 2.3	45.0	8.7 ± 2.7	6.6
100	992.2 ± 1.2	1028.5 ± 0.5	1000.1	40.7 ± 2.6	40.9	44.5 ± 2.8	42.5	5.9 ± 3.3	3.6
120	1002.2 ± 0.9	1036.2 ± 0.4	1008.0	37.8 ± 1.9	37.8	42.5 ± 2.6	40.4	3.5 ± 3.1	1.0
140	1009.2 ± 1.0	1042.6 ± 0.3	1015.4	33.6 ± 1.4	35.3	38.6 ± 1.6	38.5	-1.2 ± 2.0	-1.2
160	1015.6 ± 1.2	1048.9 ± 0.7	1022.3	32.3 ± 0.8	33.0	37.9 ± 0.7	37.0	-2.0 ± 0.9	-3.2
180	1022.8 ± 1.2	1054.6 ± 0.2	1028.9	31.2 ± 1.3	31.1	37.3 ± 1.3	35.6	-2.6 ± 1.6	-5.0
200	1029.0 ± 0.6	1061.0 ± 0.7	1035.1	28.8 ± 0.9	29.4	35.6 ± 1.2	34.3	-4.9 ± 1.5	-6.7

Table S331: Heat capacities at constant volume computed from MC simulations (c_V^{MC}), heat capacities at constant volume obtained from REFPROP[10] (c_V^{REFP}), heat capacities at constant pressure computed from MC simulations (c_P^{MC}), heat capacities at constant pressure obtained from REFPROP[10] (c_P^{REFP}), speed of sound computed from MC simulations (c^{MC}), speed of sound obtained from REFPROP[10] (c^{REFP}), viscosities computed from MD simulations (η^{MD}), and viscosities obtained from REFPROP[10] (η^{REFP}) of CO_2 rich ternary mixture with 3% impurity of H_2 and 1% impurity of Ar at 253 K and pressures ranging from 20 bar to 200 bar.

P/ [bar]	c_V^{MC}/ [J/mol K]	c_V^{REFP}/ [J/mol K]	c_P^{MC}/ [J/mol K]	c_P^{REFP}/ [J/mol K]	c^{MC}/ [m/s]	c^{REFP}/ [m/s]	η^{MD}/ [μPa s]	η^{REFP}/ [μPa s]
20	31.2 ± 0.1	32.1	49.9 ± 0.3	53.2	231.1 ± 1.4	226.6	11.5 ± 2.3	13.0
40	40.4 ± 0.2	–	99.4 ± 2.5	–	687.4 ± 18.4	–	143.4 ± 10.3	59.2
60	40.2 ± 0.2	–	97.9 ± 2.7	–	705.0 ± 13.4	–	140.9 ± 3.0	90.9
80	40.1 ± 0.1	39.9	94.4 ± 2.2	89.3	733.8 ± 24.0	712.2	145.9 ± 8.5	127.2
100	40.3 ± 0.1	39.9	92.7 ± 3.2	87.6	755.1 ± 27.9	733.0	166.3 ± 19.4	130.8
120	40.1 ± 0.2	39.9	91.3 ± 4.0	86.0	775.5 ± 25.6	752.3	145.0 ± 4.7	134.2
140	40.2 ± 0.2	39.9	87.1 ± 2.1	84.7	800.0 ± 19.5	770.3	153.5 ± 7.4	137.6
160	40.0 ± 0.2	39.9	87.1 ± 1.3	83.6	815.1 ± 12.2	787.4	152.1 ± 3.3	140.8
180	40.2 ± 0.1	40.0	87.0 ± 1.7	82.6	823.5 ± 18.6	803.5	160.2 ± 6.8	144.0
200	40.2 ± 0.2	40.0	85.7 ± 1.7	81.7	847.6 ± 16.0	818.9	170.7 ± 11.8	147.1

Table S332: Densities computed from MC and MD simulations (ρ^{MC} and ρ^{MD}), densities obtained from REFPROP[10] (ρ^{REFP}), isothermal compressibilities computed from MC simulations (β_T^{MC}), isothermal compressibilities obtained from REFPROP[10] (β_T^{REFP}), thermal expansion coefficients computed from MC simulations (α_P^{MC}), thermal expansion coefficients obtained from REFPROP[10] (α_P^{REFP}), Joule Thomson coefficients computed from MC simulations (μ_{JT}^{MC}), and Joule Thomson coefficients obtained from REFPROP[10] (μ_{JT}^{REFP}) of CO_2 rich ternary mixture with 3% impurity of H_2 and 1% impurity of Ar at 273 K and pressures ranging from 20 bar to 200 bar.

P/ [bar]	ρ^{MC}/ [kg/m^3]	ρ^{MD}/ [kg/m^3]	ρ^{REFP}/ [kg/m^3]	β_T^{MC}/ [10^{-5}/bar]	β_T^{REFP}/ [10^{-5}/bar]	α_P^{MC}/ [10^{-4}/K]	α_P^{REFP}/ [10^{-4}/K]	μ_{JT}^{MC}/ [10^{-3} K/bar]	μ_{JT}^{REFP}/ [10^{-3} K/bar]
20	42.3	44.4 ± 0.1	43.7	5764.0 ± 29.6	5909.4	55.9 ± 0.3	59.3	1174.6 ± 21.4	1303.1
40	104.1 ± 0.1	885.0 ± 1.5	221.4	4043.1 ± 67.2	−10 291.5	119.3 ± 2.2	−1071.3	1231.4 ± 35.7	–
60	858.3 ± 2.4	904.4 ± 1.7	723.3	132.6 ± 22.2	−2291.5	89.4 ± 11.8	−731.8	58.7 ± 13.7	–
80	875.1 ± 2.9	919.2 ± 0.4	889.0	107.0 ± 4.9	96.2	77.4 ± 3.1	68.1	47.4 ± 3.8	40.1
100	893.8 ± 2.4	932.8 ± 1.4	904.8	82.9 ± 8.7	80.2	64.4 ± 5.1	60.2	34.6 ± 6.4	31.0
120	905.6 ± 2.3	945.5 ± 1.4	918.3	70.3 ± 4.1	69.2	57.0 ± 2.8	54.5	26.5 ± 3.7	24.1
140	920.2 ± 0.9	955.7 ± 2.0	930.3	63.9 ± 3.6	61.1	54.4 ± 2.7	50.1	23.0 ± 3.6	18.6
160	931.6 ± 1.4	965.6 ± 1.4	941.1	54.0 ± 2.7	54.8	48.5 ± 2.2	46.7	15.9 ± 3.0	14.0
180	941.1 ± 0.9	975.5 ± 0.8	951.0	50.6 ± 2.9	49.8	46.7 ± 2.7	43.8	13.5 ± 3.7	10.2
200	950.8 ± 1.1	983.7 ± 0.6	960.1	46.1 ± 1.2	45.7	43.5 ± 1.0	41.5	9.5 ± 1.4	6.9

Table S333: Heat capacities at constant volume computed from MC simulations (c_V^{MC}), heat capacities at constant volume obtained from REFPROP[10] (c_V^{REFP}), heat capacities at constant pressure computed from MC simulations (c_P^{MC}), heat capacities at constant pressure obtained from REFPROP[10] (c_P^{REFP}), speed of sound computed from MC simulations (c^{MC}), speed of sound obtained from REFPROP[10] (c^{REFP}), viscosities computed from MD simulations (η^{MD}), and viscosities obtained from REFPROP[10] (η^{REFP}) of CO_2 rich ternary mixture with 3% impurity of H_2 and 1% impurity of Ar at 273 K and pressures ranging from 20 bar to 200 bar.

P/ [bar]	c_V^{MC}/ [J/mol K]	c_V^{REFP}/ [J/mol K]	c_P^{MC}/ [J/mol K]	c_P^{REFP}/ [J/mol K]	c^{MC}/ [m/s]	c^{REFP}/ [m/s]	η^{MD}/ [μPa s]	η^{REFP}/ [μPa s]
20	30.5	30.6	45.2 ± 0.1	46.5	246.8 ± 0.7	242.7	11.3 ± 1.9	14.0
40	36.1 ± 0.1	–	75.3 ± 0.9	–	222.5 ± 2.3	–	93.2 ± 2.7	17.7
60	40.1 ± 0.3	–	122.1 ± 8.3	–	517.1 ± 46.8	–	103.3 ± 13.0	59.8
80	40.0 ± 0.2	39.8	114.4 ± 2.8	103.0	553.0 ± 14.5	550.5	103.8 ± 3.8	92.0
100	39.7 ± 0.4	39.6	104.8 ± 3.9	97.8	596.7 ± 33.4	583.3	107.1 ± 4.0	96.2
120	39.6 ± 0.2	39.5	99.0 ± 2.7	93.9	626.4 ± 20.1	611.9	115.4 ± 10.1	100.1
140	39.5 ± 0.3	39.4	97.8 ± 2.8	91.0	648.9 ± 20.4	637.5	111.2 ± 2.2	103.7
160	39.6 ± 0.2	39.4	93.8 ± 2.3	88.6	685.7 ± 19.2	660.7	121.4 ± 10.5	107.1
180	39.5 ± 0.3	39.4	92.7 ± 3.4	86.7	701.4 ± 24.0	682.1	120.3 ± 4.7	110.4
200	39.5 ± 0.2	39.4	89.5 ± 1.2	85.1	719.3 ± 10.6	702.0	126.4 ± 4.1	113.5

Table S334: Densities computed from MC and MD simulations (ρ^{MC} and ρ^{MD}), densities obtained from REFPROP[10] (ρ^{REFP}), isothermal compressibilities computed from MC simulations (β_T^{MC}), isothermal compressibilities obtained from REFPROP[10] (β_T^{REFP}), thermal expansion coefficients computed from MC simulations (α_P^{MC}), thermal expansion coefficients obtained from REFPROP[10] (α_P^{REFP}), Joule Thomson coefficients computed from MC simulations (μ_{JT}^{MC}), and Joule Thomson coefficients obtained from REFPROP[10] (μ_{JT}^{REFP}) of CO_2 rich ternary mixture with 3% impurity of H_2 and 1% impurity of Ar at 293 K and pressures ranging from 20 bar to 200 bar.

P/ [bar]	ρ^{MC}/ [kg/m^3]	ρ^{MD}/ [kg/m^3]	ρ^{REFP}/ [kg/m^3]	β_T^{MC}/ [10^{-5}/bar]	β_T^{REFP}/ [10^{-5}/bar]	α_P^{MC}/ [10^{-4}/K]	α_P^{REFP}/ [10^{-4}/K]	μ_{JT}^{MC}/ [10^{-3} K/bar]	μ_{JT}^{REFP}/ [10^{-3} K/bar]
20	38.2	40.0 ± 0.1	39.2	5560.6 ± 37.2	5641.5	47.2 ± 0.3	49.1	983.8 ± 24.6	1081.1
40	86.9 ± 0.1	92.5 ± 0.3	91.6	3280.4 ± 45.0	3460.3	72.8 ± 1.0	81.4	990.4 ± 26.2	1077.2
60	161.4 ± 0.4	183.8 ± 1.6	182.2	3200.1 ± 110.4	3965.7	158.4 ± 6.8	227.3	976.4 ± 62.8	1014.2
80	645.3 ± 13.1	755.2 ± 3.9	693.7	2615.3 ± 1498.7	766.3	664.5 ± 303.2	268.8	304.1 ± 180.4	–
100	740.9 ± 5.2	797.9 ± 1.0	768.4	290.8 ± 29.0	249.4	133.1 ± 12.3	116.9	116.3 ± 16.4	103.8
120	778.2 ± 3.1	827.2 ± 2.2	800.4	205.7 ± 21.3	168.9	107.3 ± 8.2	89.1	90.4 ± 10.9	75.8
140	803.8 ± 1.6	848.4 ± 1.0	824.3	142.9 ± 8.8	129.2	82.3 ± 5.0	74.2	66.2 ± 7.5	58.4
160	825.7 ± 1.5	866.4 ± 1.0	843.7	113.3 ± 7.2	105.2	70.7 ± 4.8	64.7	52.2 ± 7.2	46.1
180	842.6 ± 2.0	882.2 ± 1.1	860.1	100.5 ± 6.6	89.0	66.0 ± 3.5	57.9	45.7 ± 5.2	36.8
200	858.9 ± 1.1	895.1 ± 0.8	874.5	81.0 ± 2.6	77.4	56.8 ± 1.7	52.9	34.1 ± 2.6	29.5

Table S335: Heat capacities at constant volume computed from MC simulations (c_V^{MC}), heat capacities at constant volume obtained from REFPROP[10] (c_V^{REFP}), heat capacities at constant pressure computed from MC simulations (c_P^{MC}), heat capacities at constant pressure obtained from REFPROP[10] (c_P^{REFP}), speed of sound computed from MC simulations (c^{MC}), speed of sound obtained from REFPROP[10] (c^{REFP}), viscosities computed from MD simulations (η^{MD}), and viscosities obtained from REFPROP[10] (η^{REFP}) of CO_2 rich ternary mixture with 3% impurity of H_2 and 1% impurity of Ar at 293 K and pressures ranging from 20 bar to 200 bar.

P/ [bar]	c_V^{MC}/ [J/mol K]	c_V^{REFP}/ [J/mol K]	c_P^{MC}/ [J/mol K]	c_P^{REFP}/ [J/mol K]	c^{MC}/ [m/s]	c^{REFP}/ [m/s]	η^{MD}/ [μPa s]	η^{REFP}/ [μPa s]
20	30.5	30.5	43.4 ± 0.1	44.1	259.2 ± 0.9	255.8	8.4 ± 4.5	15.0
40	33.2	33.8	56.2 ± 0.3	60.0	243.6 ± 1.8	236.6	18.1 ± 3.0	15.7
60	38.3 ± 0.2	41.3	98.7 ± 3.3	130.8	223.3 ± 5.4	209.3	17.6 ± 1.0	17.6
80	43.5 ± 1.2	–	403.2 ± 139.7	–	234.8 ± 78.7	–	68.0 ± 7.8	55.2
100	40.3 ± 0.1	40.6	143.7 ± 9.3	129.8	406.6 ± 24.2	408.3	72.8 ± 5.1	66.6
120	40.0 ± 0.1	39.9	130.2 ± 5.6	113.3	451.1 ± 25.3	458.3	78.3 ± 3.5	72.3
140	39.7 ± 0.3	39.5	113.4 ± 4.8	104.2	498.8 ± 18.8	497.6	94.9 ± 22.3	77.0
160	39.4 ± 0.2	39.3	106.3 ± 4.7	98.3	536.7 ± 20.9	530.8	85.4 ± 4.3	81.1
180	39.3 ± 0.1	39.2	103.5 ± 2.9	94.1	557.8 ± 20.0	559.8	96.2 ± 11.2	84.8
200	39.2 ± 0.2	39.1	97.0 ± 1.6	90.8	596.5 ± 10.9	585.9	97.6 ± 2.5	88.2

Table S336: Densities computed from MC and MD simulations (ρ^{MC} and ρ^{MD}), densities obtained from REFPROP[10] (ρ^{REFP}), isothermal compressibilities computed from MC simulations (β_T^{MC}), isothermal compressibilities obtained from REFPROP[10] (β_T^{REFP}), thermal expansion coefficients computed from MC simulations (α_P^{MC}), thermal expansion coefficients obtained from REFPROP[10] (α_P^{REFP}), Joule Thomson coefficients computed from MC simulations (μ_{JT}^{MC}), and Joule Thomson coefficients obtained from REFPROP[10] (μ_{JT}^{REFP}) of CO_2 rich ternary mixture with 3% impurity of H_2 and 1% impurity of Ar at 313 K and pressures ranging from 20 bar to 200 bar.

P/ [bar]	ρ^{MC}/ [kg/m^3]	ρ^{MD}/ [kg/m^3]	ρ^{REFP}/ [kg/m^3]	β_T^{MC}/ [10^{-5}/bar]	β_T^{REFP}/ [10^{-5}/bar]	α_P^{MC}/ [10^{-4}/K]	α_P^{REFP}/ [10^{-4}/K]	μ_{JT}^{MC}/ [10^{-3} K/bar]	μ_{JT}^{REFP}/ [10^{-3} K/bar]
20	34.9	36.5	35.8	5424.5 ± 24.2	5477.8	41.3 ± 0.2	42.5	835.4 ± 15.1	912.7
40	76.6	80.9 ± 0.1	79.7	3014.7 ± 23.6	3111.6	55.9 ± 0.5	60.1	830.5 ± 18.8	903.2
60	129.4	138.8 ± 0.3	138.1	2350.6 ± 31.2	2524.9	82.9 ± 1.2	95.5	813.6 ± 19.5	872.3
80	204.5 ± 0.9	231.2 ± 1.2	229.7	2244.7 ± 75.6	2702.2	143.5 ± 4.4	196.2	746.6 ± 32.6	778.9
100	338.9 ± 2.0	442.5 ± 3.4	428.8	2935.9 ± 237.1	3082.7	371.6 ± 35.6	496.7	586.1 ± 81.1	492.3
120	532.6 ± 6.2	624.3 ± 3.5	602.9	1328.9 ± 241.8	833.8	310.6 ± 50.0	231.0	310.5 ± 67.6	239.9
140	631.4 ± 2.1	692.5 ± 4.1	674.6	523.0 ± 32.0	390.0	169.7 ± 9.3	137.1	185.4 ± 14.4	153.2
160	684.5 ± 3.7	735.9 ± 2.9	717.6	312.8 ± 30.3	249.5	121.7 ± 9.6	101.6	132.2 ± 15.6	111.0
180	722.2 ± 2.9	766.1 ± 0.7	748.9	208.4 ± 16.7	183.1	92.8 ± 5.7	82.9	97.3 ± 9.6	85.5
200	747.9 ± 1.6	791.4 ± 1.3	773.6	174.7 ± 12.7	144.6	84.6 ± 5.2	71.1	83.9 ± 8.8	67.9

Table S337: Heat capacities at constant volume computed from MC simulations (c_V^{MC}), heat capacities at constant volume obtained from REFPROP[10] (c_V^{REFP}), heat capacities at constant pressure computed from MC simulations (c_P^{MC}), heat capacities at constant pressure obtained from REFPROP[10] (c_P^{REFP}), speed of sound computed from MC simulations (c^{MC}), speed of sound obtained from REFPROP[10] (c^{REFP}), viscosities computed from MD simulations (η^{MD}), and viscosities obtained from REFPROP[10] (η^{REFP}) of CO_2 rich ternary mixture with 3% impurity of H_2 and 1% impurity of Ar at 313 K and pressures ranging from 20 bar to 200 bar.

P/ [bar]	c_V^{MC}/ [J/mol K]	c_V^{REFP}/ [J/mol K]	c_P^{MC}/ [J/mol K]	c_P^{REFP}/ [J/mol K]	c^{MC}/ [m/s]	c^{REFP}/ [m/s]	η^{MD}/ [μPa s]	η^{REFP}/ [μPa s]
20	30.8	30.7	42.7 ± 0.1	43.0	270.4 ± 0.6	267.2	4.5 ± 5.0	16.0
40	32.5	32.8	50.4 ± 0.2	52.3	259.2 ± 1.2	253.4	17.3 ± 1.8	16.5
60	34.8 ± 0.1	35.6	64.7 ± 0.5	70.5	247.1 ± 1.9	238.5	19.1 ± 3.1	17.6
80	38.1 ± 0.3	39.8	97.7 ± 1.8	122.8	236.4 ± 4.6	222.9	21.8 ± 2.9	20.2
100	42.9 ± 0.4	44.8	228.7 ± 20.7	294.3	231.4 ± 14.1	222.8	30.4 ± 1.6	30.2
120	42.0 ± 0.7	42.1	225.3 ± 27.7	183.9	275.2 ± 30.3	294.9	50.9 ± 8.2	45.1
140	40.5 ± 0.4	40.5	157.3 ± 6.0	136.1	343.1 ± 12.5	357.3	57.3 ± 4.2	53.4
160	40.0 ± 0.3	39.8	132.6 ± 6.5	116.8	393.5 ± 21.4	405.0	61.3 ± 1.7	59.3
180	39.2 ± 0.2	39.4	115.9 ± 3.7	106.4	442.9 ± 19.2	443.8	72.4 ± 7.0	64.0
200	39.3 ± 0.1	39.2	112.2 ± 4.0	99.6	467.3 ± 18.9	476.9	73.8 ± 3.0	68.1

S16.22 Data of thermodynamics and transport properties of CO_2 rich ternary mixture with 3 mole% impurity of Ar and 1 mole% impurity of H_2

Table S338: Densities computed from MC and MD simulations (ρ^{MC} and ρ^{MD}), densities obtained from REFPROP[10] (ρ^{REFP}), isothermal compressibilities computed from MC simulations (β_T^{MC}), isothermal compressibilities obtained from REFPROP[10] (β_T^{REFP}), thermal expansion coefficients computed from MC simulations (α_P^{MC}), thermal expansion coefficients obtained from REFPROP[10] (α_P^{REFP}), Joule Thomson coefficients computed from MC simulations (μ_{JT}^{MC}), and Joule Thomson coefficients obtained from REFPROP[10] (μ_{JT}^{REFP}) of CO_2 rich ternary mixture with 3% impurity of Ar and 1% impurity of H_2 at 253 K and pressures ranging from 20 bar to 200 bar.

P/ [bar]	ρ^{MC}/ [kg/m^3]	ρ^{MD}/ [kg/m^3]	ρ^{REFP}/ [kg/m^3]	β_T^{MC}/ [10^{-5}/bar]	β_T^{REFP}/ [10^{-5}/bar]	α_P^{MC}/ [10^{-4}/K]	α_P^{REFP}/ [10^{-4}/K]	μ_{JT}^{MC}/ [10^{-3} K/bar]	μ_{JT}^{REFP}/ [10^{-3} K/bar]
20	49.7	50.5 ± 0.1	51.0	6238.0 ± 92.4	6472.9	73.2 ± 1.1	80.2	1483.6 ± 50.9	1634.1
40	1007.0 ± 1.9	1004.3 ± 0.7	801.7	43.0 ± 1.4	−299.7	46.7 ± 1.0	−80.7	8.3 ± 1.1	–
60	1014.2 ± 0.7	1012.8 ± 0.7	1012.1	41.9 ± 2.1	46.3	46.0 ± 2.5	46.8	7.4 ± 2.8	8.7
80	1023.2 ± 1.3	1020.2 ± 1.0	1021.1	39.1 ± 2.2	42.3	44.6 ± 2.4	44.1	5.8 ± 2.7	5.5
100	1030.0 ± 1.3	1028.5 ± 0.5	1029.4	36.3 ± 1.6	39.0	42.6 ± 2.0	41.7	3.5 ± 2.3	2.7
120	1037.5 ± 1.1	1036.2 ± 0.4	1037.1	33.1 ± 0.2	36.2	39.5 ± 0.5	39.7	-0.1 ± 0.6	0.3
140	1044.3 ± 0.9	1042.6 ± 0.3	1044.4	30.4 ± 0.6	33.8	37.2 ± 0.8	38.0	-2.8 ± 0.9	−1.9
160	1050.0 ± 0.9	1048.9 ± 0.7	1051.3	28.9 ± 0.9	31.8	35.8 ± 1.4	36.5	-4.5 ± 1.7	−3.8
180	1055.9 ± 1.0	1054.6 ± 0.2	1057.8	27.7 ± 0.7	30.0	35.0 ± 0.8	35.2	-5.5 ± 0.9	−5.4
200	1062.5 ± 0.6	1061.0 ± 0.7	1064.0	26.7 ± 0.8	28.4	34.5 ± 1.2	34.0	-6.0 ± 1.5	−7.0

Table S339: Heat capacities at constant volume computed from MC simulations (c_V^{MC}), heat capacities at constant volume obtained from REFPROP[10] (c_V^{REFP}), heat capacities at constant pressure computed from MC simulations (c_P^{MC}), heat capacities at constant pressure obtained from REFPROP[10] (c_P^{REFP}), speed of sound computed from MC simulations (c^{MC}), speed of sound obtained from REFPROP[10] (c^{REFP}), viscosities computed from MD simulations (η^{MD}), and viscosities obtained from REFPROP[10] (η^{REFP}) of CO_2 rich ternary mixture with 3% impurity of Ar and 1% impurity of H_2 at 253 K and pressures ranging from 20 bar to 200 bar.

P/ [bar]	c_V^{MC}/ [J/mol K]	c_V^{REFP}/ [J/mol K]	c_P^{MC}/ [J/mol K]	c_P^{REFP}/ [J/mol K]	c^{MC}/ [m/s]	c^{REFP}/ [m/s]	η^{MD}/ [μPa s]	η^{REFP}/ [μPa s]
20	31.2	32.2	50.2 ± 0.3	53.6	227.9 ± 1.8	224.5	11.5 ± 2.3	13.1
40	40.0 ± 0.2	–	95.3 ± 0.7	–	741.6 ± 12.9	–	143.4 ± 10.3	72.3
60	39.9 ± 0.1	39.9	94.8 ± 3.1	91.4	748.2 ± 22.7	698.7	140.9 ± 3.0	130.6
80	40.1 ± 0.1	39.9	94.5 ± 3.2	89.3	767.1 ± 25.4	720.3	145.9 ± 8.5	134.5
100	40.0 ± 0.3	39.9	93.3 ± 2.7	87.7	789.7 ± 21.1	740.2	166.3 ± 19.4	138.1
120	39.9 ± 0.1	39.9	90.0 ± 1.3	86.2	809.7 ± 6.4	758.8	145.0 ± 4.7	141.7
140	40.1 ± 0.2	39.9	87.7 ± 1.5	85.0	829.6 ± 11.0	776.3	153.5 ± 7.4	145.1
160	40.1 ± 0.1	40.0	86.5 ± 2.4	83.9	844.2 ± 17.5	792.9	152.1 ± 3.3	148.4
180	40.0 ± 0.2	40.0	86.1 ± 1.2	82.9	858.3 ± 12.0	808.6	160.2 ± 6.8	151.7
200	40.2 ± 0.2	40.0	86.3 ± 2.0	82.1	869.4 ± 16.2	823.5	170.7 ± 11.8	154.9

Table S340: Densities computed from MC and MD simulations (ρ^{MC} and ρ^{MD}), densities obtained from REFPROP[10] (ρ^{REFP}), isothermal compressibilities computed from MC simulations (β_T^{MC}), isothermal compressibilities obtained from REFPROP[10] (β_T^{REFP}), thermal expansion coefficients computed from MC simulations (α_P^{MC}), thermal expansion coefficients obtained from REFPROP[10] (α_P^{REFP}), Joule Thomson coefficients computed from MC simulations ($\mu_{\mathrm{JT}}^{\mathrm{MC}}$), and Joule Thomson coefficients obtained from REFPROP[10] ($\mu_{\mathrm{JT}}^{\mathrm{REFP}}$) of CO_2 rich ternary mixture with 3% impurity of Ar and 1% impurity of H_2 at 273 K and pressures ranging from 20 bar to 200 bar.

P/ [bar]	ρ^{MC}/ [kg/m^3]	ρ^{MD}/ [kg/m^3]	ρ^{REFP}/ [kg/m^3]	β_T^{MC}/ [10^{-5}/bar]	β_T^{REFP}/ [10^{-5}/bar]	α_P^{MC}/ [10^{-4}/K]	α_P^{REFP}/ [10^{-4}/K]	$\mu_{\mathrm{JT}}^{\mathrm{MC}}$/ [$10^{-3}$ K/bar]	$\mu_{\mathrm{JT}}^{\mathrm{REFP}}$/ [$10^{-3}$ K/bar]
20	43.7	44.4 ± 0.1	44.5	5817.3 ± 34.3	5922.4	57.0 ± 0.4	59.7	1213.0 ± 21.2	1319.8
40	109.4 ± 0.2	885.0 ± 1.5	246.2	4353.5 ± 152.4	−4189.7	134.2 ± 5.5	−566.1	1298.0 ± 83.1	–
60	905.3 ± 1.1	904.4 ± 1.7	900.2	96.7 ± 7.0	110.8	74.3 ± 4.2	76.3	43.0 ± 5.0	47.8
80	919.8 ± 2.8	919.2 ± 0.4	918.2	77.4 ± 4.5	89.2	62.8 ± 2.7	65.7	32.0 ± 3.3	36.7
100	936.4 ± 2.0	932.8 ± 1.4	933.3	64.6 ± 2.0	75.3	54.9 ± 1.3	58.5	23.5 ± 1.7	28.6
120	946.9 ± 0.6	945.5 ± 1.4	946.5	59.8 ± 2.8	65.5	52.8 ± 2.4	53.3	20.7 ± 3.1	22.2
140	959.9 ± 0.7	955.7 ± 2.0	958.3	51.9 ± 1.5	58.1	47.8 ± 1.4	49.2	14.7 ± 1.8	17.1
160	967.9 ± 2.0	965.6 ± 1.4	968.9	48.5 ± 2.1	52.4	46.0 ± 1.9	45.9	12.4 ± 2.5	12.9
180	977.1 ± 0.9	975.5 ± 0.8	978.6	42.7 ± 1.7	47.8	42.2 ± 2.0	43.3	7.6 ± 2.7	9.2
200	985.7 ± 0.8	983.7 ± 0.6	987.6	40.7 ± 0.8	43.9	40.9 ± 0.9	41.0	5.8 ± 1.2	6.1

Table S341: Heat capacities at constant volume computed from MC simulations (c_V^{MC}), heat capacities at constant volume obtained from REFPROP[10] (c_V^{REFP}), heat capacities at constant pressure computed from MC simulations (c_P^{MC}), heat capacities at constant pressure obtained from REFPROP[10] (c_P^{REFP}), speed of sound computed from MC simulations (c^{MC}), speed of sound obtained from REFPROP[10] (c^{REFP}), viscosities computed from MD simulations (η^{MD}), and viscosities obtained from REFPROP[10] (η^{REFP}) of CO_2 rich ternary mixture with 3% impurity of Ar and 1% impurity of H_2 at 273 K and pressures ranging from 20 bar to 200 bar.

P/ [bar]	c_V^{MC}/ [J/mol K]	c_V^{REFP}/ [J/mol K]	c_P^{MC}/ [J/mol K]	c_P^{REFP}/ [J/mol K]	c^{MC}/ [m/s]	c^{REFP}/ [m/s]	η^{MD}/ [μPa s]	η^{REFP}/ [μPa s]
20	30.4	30.6	45.5 ± 0.1	46.6	242.8 ± 0.8	240.5	11.3 ± 1.9	14.2
40	36.3 ± 0.1	–	81.5 ± 2.4	–	217.1 ± 5.0	–	93.2 ± 2.7	18.6
60	39.2 ± 0.2	40.0	114.7 ± 3.5	109.3	578.1 ± 22.8	523.3	103.3 ± 13.0	92.6
80	39.4 ± 0.2	39.7	105.4 ± 2.2	102.2	613.3 ± 19.1	560.3	103.8 ± 3.8	97.4
100	39.3 ± 0.2	39.6	98.7 ± 1.3	97.4	644.1 ± 10.7	591.6	107.1 ± 4.0	101.7
120	39.4 ± 0.1	39.5	98.1 ± 2.7	93.8	663.2 ± 18.3	619.1	115.4 ± 10.1	105.6
140	39.3 ± 0.1	39.4	93.7 ± 1.9	91.0	691.5 ± 12.3	643.8	111.2 ± 2.2	109.3
160	39.3 ± 0.2	39.4	92.8 ± 2.1	88.8	709.0 ± 17.7	666.3	121.4 ± 10.5	112.8
180	39.2 ± 0.1	39.4	90.1 ± 2.8	86.9	741.7 ± 18.9	687.1	120.3 ± 4.7	116.1
200	39.5 ± 0.2	39.4	88.9 ± 1.4	85.3	749.6 ± 9.7	706.5	126.4 ± 4.1	119.3

Table S342: Densities computed from MC and MD simulations (ρ^{MC} and ρ^{MD}), densities obtained from REFPROP[10] (ρ^{REFP}), isothermal compressibilities computed from MC simulations (β_T^{MC}), isothermal compressibilities obtained from REFPROP[10] (β_T^{REFP}), thermal expansion coefficients computed from MC simulations (α_P^{MC}), thermal expansion coefficients obtained from REFPROP[10] (α_P^{REFP}), Joule Thomson coefficients computed from MC simulations (μ_{JT}^{MC}), and Joule Thomson coefficients obtained from REFPROP[10] (μ_{JT}^{REFP}) of CO_2 rich ternary mixture with 3% impurity of Ar and 1% impurity of H_2 at 293 K and pressures ranging from 20 bar to 200 bar.

P/ [bar]	ρ^{MC}/ [kg/m^3]	ρ^{MD}/ [kg/m^3]	ρ^{REFP}/ [kg/m^3]	β_T^{MC}/ [10^{-5}/bar]	β_T^{REFP}/ [10^{-5}/bar]	α_P^{MC}/ [10^{-4}/K]	α_P^{REFP}/ [10^{-4}/K]	μ_{JT}^{MC}/ [10^{-3} K/bar]	μ_{JT}^{REFP}/ [10^{-3} K/bar]
20	39.4	40.0 ± 0.1	39.9	5583.3 ± 19.6	5649.1	47.7 ± 0.2	49.3	1011.7 ± 12.3	1094.4
40	90.6 ± 0.1	92.5 ± 0.3	93.6	3326.2 ± 23.1	3482.1	75.7 ± 0.6	82.6	1016.7 ± 15.9	1090.4
60	173.9 ± 0.6	183.8 ± 1.6	188.7	3588.0 ± 94.2	4189.1	191.6 ± 5.0	247.8	1004.1 ± 36.2	1022.3
80	766.4 ± 8.0	755.2 ± 3.9	751.4	383.5 ± 86.2	423.7	172.5 ± 28.7	174.7	133.3 ± 30.7	145.2
100	802.7 ± 3.0	797.9 ± 1.0	798.0	195.2 ± 20.4	224.0	106.1 ± 8.7	110.1	86.6 ± 11.2	95.6
120	829.0 ± 3.0	827.2 ± 2.2	828.2	138.3 ± 11.5	156.4	82.1 ± 5.2	85.8	64.2 ± 7.3	70.8
140	853.0 ± 2.4	848.4 ± 1.0	851.3	110.3 ± 4.4	121.5	71.6 ± 2.4	72.2	51.4 ± 3.4	54.9
160	868.3 ± 2.9	866.4 ± 1.0	870.2	90.5 ± 6.6	99.8	62.1 ± 3.9	63.4	40.3 ± 5.9	43.6
180	885.2 ± 0.8	882.2 ± 1.1	886.4	78.9 ± 3.7	85.0	57.1 ± 2.5	57.0	33.5 ± 3.7	34.9
200	896.6 ± 1.0	895.1 ± 0.8	900.6	69.8 ± 3.1	74.2	52.9 ± 2.1	52.2	27.8 ± 3.2	28.1

Table S343: Heat capacities at constant volume computed from MC simulations (c_V^{MC}), heat capacities at constant volume obtained from REFPROP[10] (c_V^{REFP}), heat capacities at constant pressure computed from MC simulations (c_P^{MC}), heat capacities at constant pressure obtained from REFPROP[10] (c_P^{REFP}), speed of sound computed from MC simulations (c^{MC}), speed of sound obtained from REFPROP[10] (c^{REFP}), viscosities computed from MD simulations (η^{MD}), and viscosities obtained from REFPROP[10] (η^{REFP}) of CO_2 rich ternary mixture with 3% impurity of Ar and 1% impurity of H_2 at 293 K and pressures ranging from 20 bar to 200 bar.

P/ [bar]	c_V^{MC}/ [J/mol K]	c_V^{REFP}/ [J/mol K]	c_P^{MC}/ [J/mol K]	c_P^{REFP}/ [J/mol K]	c^{MC}/ [m/s]	c^{REFP}/ [m/s]	η^{MD}/ [μPa s]	η^{REFP}/ [μPa s]
20	30.4	30.4	43.5 ± 0.1	44.1	255.4 ± 0.5	253.6	8.4 ± 4.5	15.2
40	33.2 ± 0.1	33.8	57.5 ± 0.2	60.5	239.7 ± 1.0	234.3	18.1 ± 3.0	15.8
60	39.6 ± 0.3	42.1	114.8 ± 1.9	141.0	215.6 ± 3.5	205.8	17.6 ± 1.0	17.9
80	40.2 ± 0.4	42.0	172.5 ± 17.2	164.1	382.1 ± 47.0	350.2	68.0 ± 7.8	63.0
100	39.6 ± 0.3	40.5	131.9 ± 6.3	126.8	460.7 ± 26.5	418.7	72.8 ± 5.1	70.8
120	39.0 ± 0.1	39.8	114.9 ± 3.8	112.2	506.6 ± 22.7	466.2	78.3 ± 3.5	76.4
140	39.1 ± 0.2	39.5	109.1 ± 2.2	103.8	544.2 ± 12.1	504.1	94.9 ± 22.3	81.2
160	38.7 ± 0.2	39.3	101.7 ± 3.8	98.2	578.0 ± 23.8	536.2	85.4 ± 4.3	85.3
180	39.1 ± 0.2	39.2	98.8 ± 2.5	94.1	601.8 ± 16.0	564.5	96.2 ± 11.2	89.0
200	38.9 ± 0.1	39.1	96.2 ± 2.2	91.0	628.2 ± 15.8	589.9	97.6 ± 2.5	92.5

Table S344: Densities computed from MC and MD simulations (ρ^{MC} and ρ^{MD}), densities obtained from REFPROP[10] (ρ^{REFP}), isothermal compressibilities computed from MC simulations (β_T^{MC}), isothermal compressibilities obtained from REFPROP[10] (β_T^{REFP}), thermal expansion coefficients computed from MC simulations (α_P^{MC}), thermal expansion coefficients obtained from REFPROP[10] (α_P^{REFP}), Joule Thomson coefficients computed from MC simulations ($\mu_{\mathrm{JT}}^{\mathrm{MC}}$), and Joule Thomson coefficients obtained from REFPROP[10] ($\mu_{\mathrm{JT}}^{\mathrm{REFP}}$) of CO_2 rich ternary mixture with 3% impurity of Ar and 1% impurity of H_2 at 313 K and pressures ranging from 20 bar to 200 bar.

P/ [bar]	ρ^{MC}/ [kg/m^3]	ρ^{MD}/ [kg/m^3]	ρ^{REFP}/ [kg/m^3]	β_T^{MC}/ [10^{-5}/bar]	β_T^{REFP}/ [10^{-5}/bar]	α_P^{MC}/ [10^{-4}/K]	α_P^{REFP}/ [10^{-4}/K]	$\mu_{\mathrm{JT}}^{\mathrm{MC}}$/ [$10^{-3}$ K/bar]	$\mu_{\mathrm{JT}}^{\mathrm{REFP}}$/ [$10^{-3}$ K/bar]
20	36.0	36.5	36.5	5429.7 ± 29.9	5482.8	41.5 ± 0.3	42.6	850.0 ± 24.4	923.6
40	79.5	80.9 ± 0.1	81.3	3047.9 ± 21.4	3121.6	57.5 ± 0.4	60.6	856.2 ± 13.5	914.1
60	135.6 ± 0.2	138.8 ± 0.3	141.3	2448.3 ± 48.4	2549.8	89.0 ± 2.1	97.4	850.0 ± 33.8	882.4
80	220.9 ± 1.2	231.2 ± 1.2	237.5	2638.5 ± 142.4	2805.1	182.1 ± 11.0	208.8	794.7 ± 66.7	784.0
100	428.1 ± 5.3	442.5 ± 3.4	461.8	3559.8 ± 272.5	3123.7	596.0 ± 55.0	543.1	489.4 ± 65.1	465.2
120	624.7 ± 6.1	624.3 ± 3.5	633.0	823.6 ± 98.2	748.8	245.1 ± 22.0	219.3	228.6 ± 27.3	223.6
140	699.8 ± 2.2	692.5 ± 4.1	701.2	380.7 ± 10.9	361.1	145.1 ± 4.2	132.4	147.9 ± 6.6	144.7
160	738.5 ± 1.5	735.9 ± 2.9	742.9	234.0 ± 10.6	235.3	104.2 ± 4.4	99.3	106.1 ± 7.2	105.9
180	770.7 ± 1.9	766.1 ± 0.7	773.5	169.1 ± 8.5	174.5	84.2 ± 3.2	81.5	81.3 ± 5.4	82.0
200	793.9 ± 1.6	791.4 ± 1.3	797.9	133.0 ± 6.9	138.7	71.8 ± 3.9	70.2	64.6 ± 6.7	65.4

Table S345: Heat capacities at constant volume computed from MC simulations (c_V^{MC}), heat capacities at constant volume obtained from REFPROP[10] (c_V^{REFP}), heat capacities at constant pressure computed from MC simulations (c_P^{MC}), heat capacities at constant pressure obtained from REFPROP[10] (c_P^{REFP}), speed of sound computed from MC simulations (c^{MC}), speed of sound obtained from REFPROP[10] (c^{REFP}), viscosities computed from MD simulations (η^{MD}), and viscosities obtained from REFPROP[10] (η^{REFP}) of CO_2 rich ternary mixture with 3% impurity of Ar and 1% impurity of H_2 at 313 K and pressures ranging from 20 bar to 200 bar.

P/ [bar]	c_V^{MC}/ [J/mol K]	c_V^{REFP}/ [J/mol K]	c_P^{MC}/ [J/mol K]	c_P^{REFP}/ [J/mol K]	c^{MC}/ [m/s]	c^{REFP}/ [m/s]	η^{MD}/ [μPa s]	η^{REFP}/ [μPa s]
20	30.7	30.6	42.7 ± 0.1	43.0	266.7 ± 0.8	264.9	4.5 ± 5.0	16.1
40	32.4	32.7	51.0 ± 0.1	52.4	254.7 ± 1.0	251.1	17.3 ± 1.8	16.7
60	34.9 ± 0.1	35.6	67.3 ± 0.9	71.4	241.0 ± 2.9	236.0	19.1 ± 3.1	17.8
80	38.6 ± 0.3	40.2	116.4 ± 4.8	129.3	227.5 ± 7.8	219.7	21.8 ± 2.9	20.6
100	42.2 ± 0.9	45.5	366.5 ± 32.8	323.7	238.7 ± 14.4	222.1	30.4 ± 1.6	32.5
120	40.3 ± 0.2	42.0	203.2 ± 12.0	180.1	313.0 ± 20.9	300.6	50.9 ± 8.2	48.1
140	39.4 ± 0.4	40.5	148.8 ± 3.6	134.7	376.5 ± 7.3	362.5	57.3 ± 4.2	56.4
160	39.1 ± 0.2	39.8	125.5 ± 4.0	116.4	431.0 ± 12.0	409.3	61.3 ± 1.7	62.4
180	38.8 ± 0.1	39.4	113.3 ± 2.6	106.3	473.3 ± 13.0	447.3	72.4 ± 7.0	67.1
200	38.8 ± 0.1	39.1	105.6 ± 3.8	99.7	507.6 ± 16.0	479.9	73.8 ± 3.0	71.3

S16.23 Data of thermodynamics and transport properties of CO_2 rich ternary mixture with 1 mole% impurity of Ar and 3 mole% impurity of N_2

Table S346: Densities computed from MC and MD simulations (ρ^{MC} and ρ^{MD}), densities obtained from REFPROP[10] (ρ^{REFP}), isothermal compressibilities computed from MC simulations (β_T^{MC}), isothermal compressibilities obtained from REFPROP[10] (β_T^{REFP}), thermal expansion coefficients computed from MC simulations (α_P^{MC}), thermal expansion coefficients obtained from REFPROP[10] (α_P^{REFP}), Joule Thomson coefficients computed from MC simulations ($\mu_{\mathrm{JT}}^{\mathrm{MC}}$), and Joule Thomson coefficients obtained from REFPROP[10] ($\mu_{\mathrm{JT}}^{\mathrm{REFP}}$) of CO_2 rich ternary mixture with 3% impurity of N_2 and 1% impurity of Ar at 253 K and pressures ranging from 20 bar to 200 bar.

P/ [bar]	ρ^{MC}/ [kg/m^3]	ρ^{MD}/ [kg/m^3]	ρ^{REFP}/ [kg/m^3]	β_T^{MC}/ [10^{-5}/bar]	β_T^{REFP}/ [10^{-5}/bar]	α_P^{MC}/ [10^{-4}/K]	α_P^{REFP}/ [10^{-4}/K]	$\mu_{\mathrm{JT}}^{\mathrm{MC}}$/ [$10^{-3}$ K/bar]	$\mu_{\mathrm{JT}}^{\mathrm{REFP}}$/ [$10^{-3}$ K/bar]
20	49.1	50.7 ± 0.1	51.3	6205.0 ± 42.9	6514.3	72.6 ± 0.6	81.8	1476.1 ± 29.5	1655.5
40	981.8 ± 1.7	996.5 ± 1.0	917.7	48.9 ± 2.7	179.1	50.4 ± 2.3	122.7	12.5 ± 2.7	–
60	991.1 ± 0.5	1005.6 ± 1.2	1011.3	43.7 ± 2.3	47.5	47.0 ± 2.4	47.4	8.7 ± 2.8	9.3
80	998.1 ± 0.8	1013.9 ± 1.3	1020.5	41.7 ± 1.2	43.2	46.0 ± 1.1	44.5	7.5 ± 1.3	6.0
100	1006.1 ± 1.4	1021.3 ± 1.2	1029.0	37.8 ± 2.4	39.7	42.7 ± 2.7	42.1	3.8 ± 3.2	3.1
120	1013.9 ± 0.7	1028.5 ± 0.8	1036.9	36.5 ± 1.3	36.8	42.5 ± 1.7	40.0	3.4 ± 1.9	0.6
140	1020.1 ± 1.1	1034.8 ± 0.5	1044.3	33.6 ± 0.8	34.4	39.6 ± 0.9	38.3	0.1 ± 1.1	-1.5
160	1026.7 ± 1.2	1041.2 ± 0.3	1051.2	29.5 ± 0.9	32.2	35.7 ± 1.1	36.7	-4.8 ± 1.4	-3.5
180	1033.4 ± 1.6	1047.4 ± 0.8	1057.8	29.7 ± 0.8	30.4	37.0 ± 1.1	35.4	-3.0 ± 1.4	-5.2
200	1038.2 ± 0.6	1053.2 ± 0.5	1064.1	28.4 ± 1.6	28.8	35.5 ± 2.0	34.1	-4.9 ± 2.4	-6.8

Table S347: Heat capacities at constant volume computed from MC simulations (c_V^{MC}), heat capacities at constant volume obtained from REFPROP[10] (c_V^{REFP}), heat capacities at constant pressure computed from MC simulations (c_P^{MC}), heat capacities at constant pressure obtained from REFPROP[10] (c_P^{REFP}), speed of sound computed from MC simulations (c^{MC}), speed of sound obtained from REFPROP[10] (c^{REFP}), viscosities computed from MD simulations (η^{MD}), and viscosities obtained from REFPROP[10] (η^{REFP}) of CO_2 rich ternary mixture with 3% impurity of N_2 and 1% impurity of Ar at 253 K and pressures ranging from 20 bar to 200 bar.

P/ [bar]	c_V^{MC}/ [J/mol K]	c_V^{REFP}/ [J/mol K]	c_P^{MC}/ [J/mol K]	c_P^{REFP}/ [J/mol K]	c^{MC}/ [m/s]	c^{REFP}/ [m/s]	η^{MD}/ [μPa s]	η^{REFP}/ [μPa s]
20	31.4	32.7	50.2 ± 0.3	54.8	229.1 ± 1.0	223.7	13.6 ± 2.4	13.1
40	40.1 ± 0.2	–	97.8 ± 2.6	–	712.8 ± 22.3	–	134.5 ± 3.0	99.4
60	40.1 ± 0.1	40.4	95.6 ± 2.8	91.9	741.7 ± 22.2	688.0	135.4 ± 3.6	131.9
80	39.9 ± 0.1	40.4	95.5 ± 1.2	89.8	757.9 ± 12.2	710.1	144.1 ± 4.2	135.8
100	39.9 ± 0.3	40.3	92.4 ± 3.6	88.0	780.8 ± 29.8	730.6	147.9 ± 10.1	139.5
120	40.2 ± 0.1	40.3	93.3 ± 2.3	86.6	792.6 ± 17.6	749.7	147.7 ± 6.9	143.1
140	40.3 ± 0.2	40.3	90.3 ± 1.9	85.3	808.8 ± 13.1	767.5	160.8 ± 12.6	146.6
160	40.2 ± 0.2	40.4	85.9 ± 1.8	84.2	839.6 ± 15.2	784.4	152.0 ± 6.0	150.0
180	40.4 ± 0.3	40.4	88.8 ± 1.9	83.2	847.0 ± 15.0	800.4	166.5 ± 26.9	153.3
200	40.3 ± 0.4	40.4	86.8 ± 2.5	82.3	854.2 ± 27.4	815.7	157.3 ± 7.9	156.6

Table S348: Densities computed from MC and MD simulations (ρ^{MC} and ρ^{MD}), densities obtained from REFPROP[10] (ρ^{REFP}), isothermal compressibilities computed from MC simulations (β_T^{MC}), isothermal compressibilities obtained from REFPROP[10] (β_T^{REFP}), thermal expansion coefficients computed from MC simulations (α_P^{MC}), thermal expansion coefficients obtained from REFPROP[10] (α_P^{REFP}), Joule Thomson coefficients computed from MC simulations ($\mu_{\mathrm{JT}}^{\mathrm{MC}}$), and Joule Thomson coefficients obtained from REFPROP[10] ($\mu_{\mathrm{JT}}^{\mathrm{REFP}}$) of CO_2 rich ternary mixture with 3% impurity of N_2 and 1% impurity of Ar at 273 K and pressures ranging from 20 bar to 200 bar.

P/ [bar]	ρ^{MC}/ [kg/m^3]	ρ^{MD}/ [kg/m^3]	ρ^{REFP}/ [kg/m^3]	β_T^{MC}/ [10^{-5}/bar]	β_T^{REFP}/ [10^{-5}/bar]	α_P^{MC}/ [10^{-4}/K]	α_P^{REFP}/ [10^{-4}/K]	$\mu_{\mathrm{JT}}^{\mathrm{MC}}$/ [$10^{-3}$ K/bar]	$\mu_{\mathrm{JT}}^{\mathrm{REFP}}$/ [$10^{-3}$ K/bar]
20	43.2	44.5 ± 0.2	44.6	5805.5 ± 28.2	5939.5	56.7 ± 0.3	60.4	1210.1 ± 17.1	1336.0
40	107.8 ± 0.2	878.3 ± 1.0	246.2	4183.2 ± 69.6	-3925.6	126.5 ± 2.3	-543.9	1270.0 ± 36.2	–
60	878.5 ± 0.5	897.0 ± 1.8	897.3	108.1 ± 11.4	116.7	77.5 ± 5.8	78.7	48.1 ± 7.1	50.2
80	896.4 ± 1.2	913.6 ± 0.6	916.1	86.8 ± 1.8	92.8	67.4 ± 1.5	67.2	37.6 ± 2.0	38.3
100	910.7 ± 1.3	926.6 ± 1.3	931.8	71.9 ± 3.6	77.8	58.9 ± 3.1	59.5	28.4 ± 4.0	29.7
120	923.8 ± 1.1	938.8 ± 1.1	945.4	62.1 ± 3.2	67.3	53.2 ± 2.6	54.0	21.8 ± 3.4	23.1
140	934.2 ± 1.6	949.1 ± 0.7	957.4	56.7 ± 4.0	59.5	49.9 ± 2.7	49.8	17.8 ± 3.7	17.8
160	944.9 ± 1.5	959.6 ± 0.9	968.2	51.5 ± 1.1	53.5	47.6 ± 0.8	46.4	14.6 ± 1.0	13.4
180	953.6 ± 1.0	968.5 ± 0.9	978.2	46.1 ± 1.5	48.6	43.7 ± 1.4	43.6	9.8 ± 1.9	9.7
200	962.4 ± 1.0	976.5 ± 0.4	987.3	43.2 ± 1.9	44.7	42.2 ± 2.0	41.3	7.6 ± 2.7	6.6

Table S349: Heat capacities at constant volume computed from MC simulations (c_V^{MC}), heat capacities at constant volume obtained from REFPROP[10] (c_V^{REFP}), heat capacities at constant pressure computed from MC simulations (c_P^{MC}), heat capacities at constant pressure obtained from REFPROP[10] (c_P^{REFP}), speed of sound computed from MC simulations (c^{MC}), speed of sound obtained from REFPROP[10] (c^{REFP}), viscosities computed from MD simulations (η^{MD}), and viscosities obtained from REFPROP[10] (η^{REFP}) of CO_2 rich ternary mixture with 3% impurity of N_2 and 1% impurity of Ar at 273 K and pressures ranging from 20 bar to 200 bar.

P/ [bar]	c_V^{MC}/ [J/mol K]	c_V^{REFP}/ [J/mol K]	c_P^{MC}/ [J/mol K]	c_P^{REFP}/ [J/mol K]	c^{MC}/ [m/s]	c^{REFP}/ [m/s]	η^{MD}/ [μPa s]	η^{REFP}/ [μPa s]
20	30.5	30.9	45.5 ± 0.1	47.2	243.8 ± 0.6	240.0	11.3 ± 3.2	14.1
40	36.4 ± 0.2	–	77.9 ± 0.9	–	218.0 ± 2.3	–	95.7 ± 5.3	18.6
60	39.6 ± 0.2	40.7	114.8 ± 4.2	110.8	552.6 ± 30.8	510.3	98.1 ± 2.3	93.0
80	39.5 ± 0.2	40.3	108.6 ± 1.8	103.3	594.4 ± 8.2	548.8	104.3 ± 6.4	97.9
100	39.5 ± 0.1	40.1	102.1 ± 3.5	98.2	627.9 ± 19.1	581.1	106.5 ± 4.4	102.4
120	39.4 ± 0.1	40.0	97.6 ± 2.9	94.5	657.1 ± 19.4	609.4	115.3 ± 7.9	106.4
140	39.5 ± 0.2	39.9	95.2 ± 2.4	91.6	674.3 ± 25.6	634.7	124.5 ± 18.9	110.2
160	39.5 ± 0.1	39.9	94.6 ± 0.8	89.3	701.6 ± 8.4	657.7	115.9 ± 8.2	113.8
180	39.4 ± 0.1	39.8	90.6 ± 1.7	87.4	723.3 ± 13.8	679.0	126.6 ± 19.1	117.2
200	39.6 ± 0.2	39.8	89.9 ± 2.6	85.8	739.4 ± 19.8	698.8	127.1 ± 6.9	120.4

Table S350: Densities computed from MC and MD simulations (ρ^{MC} and ρ^{MD}), densities obtained from REFPROP[10] (ρ^{REFP}), isothermal compressibilities computed from MC simulations (β_T^{MC}), isothermal compressibilities obtained from REFPROP[10] (β_T^{REFP}), thermal expansion coefficients computed from MC simulations (α_P^{MC}), thermal expansion coefficients obtained from REFPROP[10] (α_P^{REFP}), Joule Thomson coefficients computed from MC simulations ($\mu_{\mathrm{JT}}^{\mathrm{MC}}$), and Joule Thomson coefficients obtained from REFPROP[10] ($\mu_{\mathrm{JT}}^{\mathrm{REFP}}$) of CO_2 rich ternary mixture with 3% impurity of N_2 and 1% impurity of Ar at 293 K and pressures ranging from 20 bar to 200 bar.

P/ [bar]	ρ^{MC}/ [kg/m^3]	ρ^{MD}/ [kg/m^3]	ρ^{REFP}/ [kg/m^3]	β_T^{MC}/ [10^{-5}/bar]	β_T^{REFP}/ [10^{-5}/bar]	α_P^{MC}/ [10^{-4}/K]	α_P^{REFP}/ [10^{-4}/K]	$\mu_{\mathrm{JT}}^{\mathrm{MC}}$/ [$10^{-3}$ K/bar]	$\mu_{\mathrm{JT}}^{\mathrm{REFP}}$/ [$10^{-3}$ K/bar]
20	39.0	40.0	40.0	5549.9 ± 31.2	5658.2	47.4 ± 0.3	49.6	994.0 ± 20.2	1106.8
40	89.5 ± 0.1	92.7 ± 0.4	94.0	3276.9 ± 50.6	3502.6	73.9 ± 1.2	84.1	996.7 ± 31.9	1097.5
60	170.4 ± 0.3	185.6 ± 2.0	191.9	3536.7 ± 59.6	4379.3	184.2 ± 3.4	267.5	1016.6 ± 28.7	1020.8
80	727.5 ± 6.2	756.2 ± 4.1	742.8	463.6 ± 84.9	478.7	192.7 ± 29.8	189.1	152.0 ± 33.0	155.2
100	774.1 ± 4.5	795.7 ± 1.4	793.3	242.7 ± 42.9	238.7	119.9 ± 15.7	114.3	102.1 ± 20.0	99.7
120	804.3 ± 2.4	823.0 ± 2.0	825.0	162.3 ± 11.6	163.6	92.3 ± 6.5	87.9	75.3 ± 9.2	73.2
140	826.0 ± 1.0	843.8 ± 1.7	849.0	118.4 ± 6.6	125.8	73.5 ± 3.7	73.6	55.6 ± 5.6	56.6
160	846.1 ± 3.3	861.4 ± 1.6	868.4	101.5 ± 5.7	102.8	66.6 ± 2.0	64.3	46.6 ± 3.0	44.8
180	861.1 ± 1.2	876.1 ± 1.2	885.0	86.1 ± 4.3	87.2	60.1 ± 2.6	57.7	38.1 ± 3.9	35.9
200	875.4 ± 1.2	889.1 ± 1.2	899.5	72.5 ± 2.8	75.8	52.6 ± 1.5	52.8	28.5 ± 2.4	28.8

Table S351: Heat capacities at constant volume computed from MC simulations (c_V^{MC}), heat capacities at constant volume obtained from REFPROP[10] (c_V^{REFP}), heat capacities at constant pressure computed from MC simulations (c_P^{MC}), heat capacities at constant pressure obtained from REFPROP[10] (c_P^{REFP}), speed of sound computed from MC simulations (c^{MC}), speed of sound obtained from REFPROP[10] (c^{REFP}), viscosities computed from MD simulations (η^{MD}), and viscosities obtained from REFPROP[10] (η^{REFP}) of CO_2 rich ternary mixture with 3% impurity of N_2 and 1% impurity of Ar at 293 K and pressures ranging from 20 bar to 200 bar.

P/ [bar]	c_V^{MC}/ [J/mol K]	c_V^{REFP}/ [J/mol K]	c_P^{MC}/ [J/mol K]	c_P^{REFP}/ [J/mol K]	c^{MC}/ [m/s]	c^{REFP}/ [m/s]	η^{MD}/ [μPa s]	η^{REFP}/ [μPa s]
20	30.5	30.7	43.6 ± 0.1	44.5	257.1 ± 0.8	253.1	3.7 ± 3.5	15.1
40	33.3 ± 0.1	34.3	56.8 ± 0.4	61.7	240.9 ± 2.1	233.6	15.1 ± 1.0	15.8
60	39.1 ± 0.3	43.3	110.4 ± 1.8	151.8	216.5 ± 2.7	204.2	18.8 ± 1.4	18.0
80	40.6 ± 0.4	43.2	182.8 ± 19.8	171.3	365.4 ± 38.9	334.1	65.0 ± 1.1	62.3
100	40.1 ± 0.6	41.3	138.3 ± 9.6	129.1	428.3 ± 40.8	406.5	77.5 ± 4.7	70.7
120	39.5 ± 0.4	40.5	122.4 ± 5.9	113.5	487.6 ± 21.1	455.7	82.7 ± 5.5	76.6
140	39.4 ± 0.3	40.1	109.3 ± 3.6	104.7	532.8 ± 17.3	494.6	81.0 ± 1.9	81.5
160	39.0 ± 0.3	39.8	104.7 ± 1.6	99.0	558.9 ± 16.3	527.5	84.6 ± 1.0	85.8
180	39.1 ± 0.2	39.7	100.8 ± 2.6	94.8	589.6 ± 16.6	556.3	92.5 ± 3.9	89.6
200	39.0 ± 0.1	39.6	94.4 ± 1.3	91.6	617.2 ± 12.8	582.2	96.1 ± 4.5	93.2

Table S352: Densities computed from MC and MD simulations (ρ^{MC} and ρ^{MD}), densities obtained from REFPROP[10] (ρ^{REFP}), isothermal compressibilities computed from MC simulations (β_T^{MC}), isothermal compressibilities obtained from REFPROP[10] (β_T^{REFP}), thermal expansion coefficients computed from MC simulations (α_P^{MC}), thermal expansion coefficients obtained from REFPROP[10] (α_P^{REFP}), Joule Thomson coefficients computed from MC simulations ($\mu_{\mathrm{JT}}^{\mathrm{MC}}$), and Joule Thomson coefficients obtained from REFPROP[10] ($\mu_{\mathrm{JT}}^{\mathrm{REFP}}$) of CO_2 rich ternary mixture with 3% impurity of N_2 and 1% impurity of Ar at 313 K and pressures ranging from 20 bar to 200 bar.

P/ [bar]	ρ^{MC}/ [kg/m^3]	ρ^{MD}/ [kg/m^3]	ρ^{REFP}/ [kg/m^3]	β_T^{MC}/ [10^{-5}/bar]	β_T^{REFP}/ [10^{-5}/bar]	α_P^{MC}/ [10^{-4}/K]	α_P^{REFP}/ [10^{-4}/K]	$\mu_{\mathrm{JT}}^{\mathrm{MC}}$/ [$10^{-3}$ K/bar]	$\mu_{\mathrm{JT}}^{\mathrm{REFP}}$/ [$10^{-3}$ K/bar]
20	35.7	36.6	36.5	5417.7 ± 30.0	5488.1	41.4 ± 0.3	42.8	842.0 ± 25.2	933.1
40	78.6	81.1 ± 0.2	81.5	3048.5 ± 18.4	3129.5	57.4 ± 0.4	61.2	863.6 ± 12.6	919.6
60	133.8 ± 0.2	139.5 ± 0.6	142.1	2409.1 ± 66.0	2566.8	87.1 ± 2.2	99.3	841.7 ± 35.5	884.8
80	216.8 ± 1.3	233.3 ± 1.0	240.6	2530.9 ± 108.7	2853.6	171.6 ± 6.9	217.6	789.4 ± 43.2	–
100	394.5 ± 1.0	448.6 ± 9.3	460.8	3382.8 ± 231.4	2880.3	518.3 ± 35.5	499.8	525.6 ± 50.8	456.6
120	595.6 ± 4.3	623.0 ± 1.8	626.4	879.0 ± 83.1	773.1	249.8 ± 19.4	220.6	244.5 ± 25.7	228.1
140	672.5 ± 5.1	693.8 ± 2.1	696.5	388.2 ± 51.6	374.6	142.9 ± 15.8	134.4	153.4 ± 24.2	147.8
160	713.4 ± 5.1	733.1 ± 0.5	739.5	265.9 ± 21.9	242.8	111.3 ± 6.9	100.6	118.0 ± 11.1	107.9
180	747.6 ± 2.4	763.3 ± 1.7	770.9	184.5 ± 12.8	179.3	88.6 ± 5.6	82.5	88.7 ± 9.5	83.4
200	771.0 ± 0.4	786.5 ± 1.6	795.9	147.1 ± 9.1	142.0	75.6 ± 3.7	71.0	71.8 ± 6.5	66.5

Table S353: Heat capacities at constant volume computed from MC simulations (c_V^{MC}), heat capacities at constant volume obtained from REFPROP[10] (c_V^{REFP}), heat capacities at constant pressure computed from MC simulations (c_P^{MC}), heat capacities at constant pressure obtained from REFPROP[10] (c_P^{REFP}), speed of sound computed from MC simulations (c^{MC}), speed of sound obtained from REFPROP[10] (c^{REFP}), viscosities computed from MD simulations (η^{MD}), and viscosities obtained from REFPROP[10] (η^{REFP}) of CO_2 rich ternary mixture with 3% impurity of N_2 and 1% impurity of Ar at 313 K and pressures ranging from 20 bar to 200 bar.

P/ [bar]	c_V^{MC}/ [J/mol K]	c_V^{REFP}/ [J/mol K]	c_P^{MC}/ [J/mol K]	c_P^{REFP}/ [J/mol K]	c^{MC}/ [m/s]	c^{REFP}/ [m/s]	η^{MD}/ [μPa s]	η^{REFP}/ [μPa s]
20	30.8	30.9	42.7 ± 0.1	43.3	267.9 ± 0.8	264.6	4.6 ± 5.2	16.1
40	32.6	33.1	51.1 ± 0.1	53.1	255.9 ± 0.8	250.6	17.4 ± 2.3	16.7
60	35.0 ± 0.1	36.1	66.6 ± 0.8	72.9	243.0 ± 3.7	235.3	18.3 ± 1.7	17.8
80	38.3 ± 0.2	–	111.1 ± 2.5	–	229.8 ± 5.7	–	21.2 ± 1.6	20.7
100	42.5 ± 0.6	46.5	319.4 ± 20.3	302.7	237.4 ± 11.2	221.5	31.0 ± 1.5	32.5
120	41.3 ± 0.3	43.0	203.7 ± 11.2	179.7	306.9 ± 16.9	293.9	48.5 ± 2.4	47.7
140	39.6 ± 0.2	41.2	146.4 ± 10.0	135.5	376.4 ± 28.2	354.9	55.8 ± 3.3	56.3
160	39.2 ± 0.1	40.4	128.3 ± 4.4	117.2	415.4 ± 18.6	401.8	67.7 ± 5.5	62.4
180	39.2 ± 0.2	40.0	116.3 ± 4.7	107.0	463.8 ± 18.7	440.2	68.3 ± 2.4	67.3
200	38.8 ± 0.3	39.7	107.4 ± 2.9	100.4	494.3 ± 16.8	473.0	80.1 ± 5.1	71.6

S16.24 Data of thermodynamics and transport properties of CO_2 rich ternary mixture with 3 mole% impurity of Ar and 1 mole% impurity of N_2

Table S354: Densities computed from MC and MD simulations (ρ^{MC} and ρ^{MD}), densities obtained from REFPROP[10] (ρ^{REFP}), isothermal compressibilities computed from MC simulations (β_T^{MC}), isothermal compressibilities obtained from REFPROP[10] (β_T^{REFP}), thermal expansion coefficients computed from MC simulations (α_P^{MC}), thermal expansion coefficients obtained from REFPROP[10] (α_P^{REFP}), Joule Thomson coefficients computed from MC simulations (μ_{JT}^{MC}), and Joule Thomson coefficients obtained from REFPROP[10] (μ_{JT}^{REFP}) of CO_2 rich ternary mixture with 3% impurity of Ar and 1% impurity of N_2 at 253 K and pressures ranging from 20 bar to 200 bar.

P/ [bar]	ρ^{MC}/ [kg/m^3]	ρ^{MD}/ [kg/m^3]	ρ^{REFP}/ [kg/m^3]	β_T^{MC}/ [10^{-5}/bar]	β_T^{REFP}/ [10^{-5}/bar]	α_P^{MC}/ [10^{-4}/K]	α_P^{REFP}/ [10^{-4}/K]	μ_{JT}^{MC}/ [10^{-3} K/bar]	μ_{JT}^{REFP}/ [10^{-3} K/bar]
20	50.1	51.0 ± 0.1	51.5	6310.2 ± 59.7	6496.9	74.7 ± 1.0	81.1	1530.2 ± 44.2	1648.4
40	1011.0 ± 0.7	1007.9 ± 0.9	1006.0	42.9 ± 1.1	54.3	46.6 ± 1.1	52.3	8.1 ± 1.2	–
60	1019.7 ± 1.1	1017.2 ± 0.5	1021.9	39.4 ± 2.0	45.8	44.0 ± 2.4	46.7	5.2 ± 2.8	8.4
80	1027.5 ± 1.9	1025.1 ± 1.0	1030.9	35.9 ± 2.6	41.9	41.1 ± 2.7	43.9	1.8 ± 3.2	5.3
100	1035.5 ± 1.8	1033.2 ± 0.7	1039.2	33.8 ± 1.3	38.6	39.9 ± 1.5	41.6	0.4 ± 1.8	2.5
120	1042.0 ± 1.0	1040.2 ± 0.2	1047.0	33.0 ± 1.2	35.8	39.7 ± 1.4	39.6	0.2 ± 1.6	0.1
140	1047.0 ± 1.0	1046.8 ± 0.6	1054.3	30.6 ± 0.8	33.5	37.5 ± 1.1	37.9	-2.4 ± 1.3	−2.0
160	1054.3 ± 0.8	1053.0 ± 0.6	1061.1	27.6 ± 1.5	31.5	34.5 ± 2.0	36.4	-6.1 ± 2.5	−3.8
180	1059.6 ± 1.1	1058.6 ± 0.8	1067.6	27.7 ± 1.4	29.7	35.3 ± 2.1	35.1	-5.1 ± 2.5	−5.5
200	1066.5 ± 1.6	1064.1 ± 0.5	1073.8	25.3 ± 0.9	28.2	32.9 ± 1.4	33.9	-8.2 ± 1.7	−7.0

Table S355: Heat capacities at constant volume computed from MC simulations (c_V^{MC}), heat capacities at constant volume obtained from REFPROP[10] (c_V^{REFP}), heat capacities at constant pressure computed from MC simulations (c_P^{MC}), heat capacities at constant pressure obtained from REFPROP[10] (c_P^{REFP}), speed of sound computed from MC simulations (c^{MC}), speed of sound obtained from REFPROP[10] (c^{REFP}), viscosities computed from MD simulations (η^{MD}), and viscosities obtained from REFPROP[10] (η^{REFP}) of CO_2 rich ternary mixture with 3% impurity of Ar and 1% impurity of N_2 at 253 K and pressures ranging from 20 bar to 200 bar.

P/ [bar]	c_V^{MC}/ [J/mol K]	c_V^{REFP}/ [J/mol K]	c_P^{MC}/ [J/mol K]	c_P^{REFP}/ [J/mol K]	c^{MC}/ [m/s]	c^{REFP}/ [m/s]	η^{MD}/ [μPa s]	η^{REFP}/ [μPa s]
20	31.3	32.4	50.8 ± 0.3	54.1	226.5 ± 1.3	223.5	12.0 ± 3.3	13.2
40	39.9 ± 0.1	–	95.3 ± 1.2	–	742.3 ± 10.5	–	134.8 ± 3.5	127.0
60	39.9 ± 0.3	40.1	93.1 ± 3.1	91.5	761.8 ± 23.5	698.1	142.9 ± 8.7	133.6
80	39.8 ± 0.1	40.1	90.3 ± 3.1	89.5	784.0 ± 31.3	719.6	150.8 ± 15.9	137.4
100	39.8 ± 0.2	40.1	90.1 ± 2.1	87.8	804.6 ± 18.0	739.4	150.8 ± 2.4	141.2
120	40.0 ± 0.2	40.1	90.7 ± 1.9	86.4	812.1 ± 16.7	758.0	168.4 ± 13.4	144.7
140	39.9 ± 0.2	40.1	88.8 ± 1.5	85.2	834.2 ± 13.0	775.4	163.7 ± 13.0	148.2
160	40.0 ± 0.2	40.1	85.3 ± 3.1	84.1	856.0 ± 27.5	791.9	164.4 ± 21.3	151.6
180	40.0 ± 0.2	40.1	86.9 ± 3.1	83.1	861.1 ± 27.3	807.5	174.6 ± 33.4	154.9
200	40.2 ± 0.2	40.2	84.4 ± 2.0	82.3	882.6 ± 19.6	822.4	193.3 ± 35.9	158.2

Table S356: Densities computed from MC and MD simulations (ρ^{MC} and ρ^{MD}), densities obtained from REFPROP[10] (ρ^{REFP}), isothermal compressibilities computed from MC simulations (β_T^{MC}), isothermal compressibilities obtained from REFPROP[10] (β_T^{REFP}), thermal expansion coefficients computed from MC simulations (α_P^{MC}), thermal expansion coefficients obtained from REFPROP[10] (α_P^{REFP}), Joule Thomson coefficients computed from MC simulations ($\mu_{\mathrm{JT}}^{\mathrm{MC}}$), and Joule Thomson coefficients obtained from REFPROP[10] ($\mu_{\mathrm{JT}}^{\mathrm{REFP}}$) of CO_2 rich ternary mixture with 3% impurity of Ar and 1% impurity of N_2 at 273 K and pressures ranging from 20 bar to 200 bar.

P/ [bar]	ρ^{MC}/ [kg/m^3]	ρ^{MD}/ [kg/m^3]	ρ^{REFP}/ [kg/m^3]	β_T^{MC}/ [10^{-5}/bar]	β_T^{REFP}/ [10^{-5}/bar]	α_P^{MC}/ [10^{-4}/K]	α_P^{REFP}/ [10^{-4}/K]	$\mu_{\mathrm{JT}}^{\mathrm{MC}}$/ [$10^{-3}$ K/bar]	$\mu_{\mathrm{JT}}^{\mathrm{REFP}}$/ [$10^{-3}$ K/bar]
20	44.0	44.7 ± 0.1	44.8	5806.1 ± 72.4	5932.3	57.0 ± 0.7	60.0	1212.3 ± 43.0	1330.7
40	110.6 ± 0.3	890.4 ± 1.4	256.5	4389.1 ± 86.2	-3209.6	135.4 ± 2.4	-489.4	1304.4 ± 34.7	–
60	912.4 ± 4.8	911.4 ± 1.7	909.4	90.5 ± 15.0	109.2	70.2 ± 8.9	75.8	39.5 ± 10.8	47.1
80	926.2 ± 1.6	925.5 ± 1.4	927.4	74.3 ± 4.9	88.2	61.0 ± 3.4	65.4	30.2 ± 4.4	36.2
100	940.4 ± 1.6	940.2 ± 1.4	942.6	66.9 ± 4.7	74.6	57.1 ± 3.6	58.3	25.6 ± 4.6	28.2
120	951.4 ± 0.7	950.7 ± 1.6	955.7	58.9 ± 1.5	64.9	52.8 ± 0.9	53.1	20.5 ± 1.1	21.9
140	962.8 ± 0.9	960.7 ± 0.8	967.5	52.8 ± 1.7	57.6	48.9 ± 1.7	49.1	15.9 ± 2.2	16.9
160	972.0 ± 0.7	970.3 ± 1.3	978.1	45.8 ± 2.1	52.0	43.8 ± 1.8	45.9	9.7 ± 2.5	12.7
180	980.8 ± 0.9	979.4 ± 1.1	987.9	44.0 ± 0.8	47.4	43.5 ± 0.8	43.2	9.1 ± 1.1	9.1
200	989.4 ± 0.5	986.7 ± 0.5	996.9	40.5 ± 0.8	43.6	40.9 ± 1.0	40.9	5.8 ± 1.4	6.0

Table S357: Heat capacities at constant volume computed from MC simulations (c_V^{MC}), heat capacities at constant volume obtained from REFPROP[10] (c_V^{REFP}), heat capacities at constant pressure computed from MC simulations (c_P^{MC}), heat capacities at constant pressure obtained from REFPROP[10] (c_P^{REFP}), speed of sound computed from MC simulations (c^{MC}), speed of sound obtained from REFPROP[10] (c^{REFP}), viscosities computed from MD simulations (η^{MD}), and viscosities obtained from REFPROP[10] (η^{REFP}) of CO_2 rich ternary mixture with 3% impurity of Ar and 1% impurity of N_2 at 273 K and pressures ranging from 20 bar to 200 bar.

P/ [bar]	c_V^{MC}/ [J/mol K]	c_V^{REFP}/ [J/mol K]	c_P^{MC}/ [J/mol K]	c_P^{REFP}/ [J/mol K]	c^{MC}/ [m/s]	c^{REFP}/ [m/s]	η^{MD}/ [μPa s]	η^{REFP}/ [μPa s]
20	30.4	30.7	45.5 ± 0.2	46.8	242.2 ± 1.6	239.7	12.6 ± 4.0	14.2
40	36.6	–	81.8 ± 0.8	–	214.5 ± 2.4	–	95.3 ± 1.7	19.0
60	39.3 ± 0.2	40.2	111.3 ± 7.3	109.2	586.1 ± 52.2	523.0	100.7 ± 3.6	94.6
80	39.3 ± 0.2	39.9	104.0 ± 3.3	102.3	619.8 ± 22.6	559.8	104.6 ± 1.6	99.5
100	39.2 ± 0.1	39.7	101.5 ± 3.8	97.5	641.2 ± 25.6	590.9	124.7 ± 27.4	103.8
120	39.2 ± 0.1	39.6	98.9 ± 0.6	94.0	670.8 ± 8.8	618.3	121.4 ± 22.7	107.8
140	39.2 ± 0.1	39.6	95.5 ± 2.1	91.2	691.8 ± 13.6	642.9	115.6 ± 4.5	111.6
160	39.2 ± 0.1	39.6	90.6 ± 2.0	89.0	719.9 ± 18.6	665.3	122.9 ± 5.6	115.1
180	39.3 ± 0.2	39.5	91.5 ± 1.3	87.1	734.4 ± 8.9	686.1	138.0 ± 7.8	118.5
200	39.6 ± 0.1	39.5	89.3 ± 1.7	85.6	749.9 ± 10.2	705.4	127.7 ± 6.9	121.7

Table S358: Densities computed from MC and MD simulations (ρ^{MC} and ρ^{MD}), densities obtained from REFPROP[10] (ρ^{REFP}), isothermal compressibilities computed from MC simulations (β_T^{MC}), isothermal compressibilities obtained from REFPROP[10] (β_T^{REFP}), thermal expansion coefficients computed from MC simulations (α_P^{MC}), thermal expansion coefficients obtained from REFPROP[10] (α_P^{REFP}), Joule Thomson coefficients computed from MC simulations ($\mu_{\mathrm{JT}}^{\mathrm{MC}}$), and Joule Thomson coefficients obtained from REFPROP[10] ($\mu_{\mathrm{JT}}^{\mathrm{REFP}}$) of CO_2 rich ternary mixture with 3% impurity of Ar and 1% impurity of N_2 at 293 K and pressures ranging from 20 bar to 200 bar.

P/ [bar]	ρ^{MC}/ [kg/m^3]	ρ^{MD}/ [kg/m^3]	ρ^{REFP}/ [kg/m^3]	β_T^{MC}/ [10^{-5}/bar]	β_T^{REFP}/ [10^{-5}/bar]	α_P^{MC}/ [10^{-4}/K]	α_P^{REFP}/ [10^{-4}/K]	$\mu_{\mathrm{JT}}^{\mathrm{MC}}$/ [$10^{-3}$ K/bar]	$\mu_{\mathrm{JT}}^{\mathrm{REFP}}$/ [$10^{-3}$ K/bar]
20	39.7	40.3 ± 0.1	40.2	5596.2 ± 25.7	5654.6	47.9 ± 0.2	49.4	1020.8 ± 16.2	1102.9
40	91.4	93.5 ± 0.4	94.4	3350.3 ± 22.5	3496.2	76.3 ± 0.6	83.4	1025.8 ± 14.4	1097.1
60	177.4 ± 0.6	248.1 ± 58.7	192.1	3686.2 ± 71.7	4338.9	200.8 ± 2.8	262.3	1010.9 ± 22.2	1024.3
80	775.3 ± 5.5	768.5 ± 1.4	760.3	304.7 ± 50.8	412.9	146.6 ± 19.1	171.8	117.4 ± 22.0	142.6
100	809.5 ± 2.0	807.1 ± 1.4	806.5	181.2 ± 14.7	220.8	99.3 ± 6.8	109.3	81.6 ± 9.1	94.3
120	837.7 ± 2.0	833.9 ± 2.1	836.7	132.6 ± 7.7	154.8	81.2 ± 4.4	85.4	62.2 ± 6.2	70.0
140	856.8 ± 2.4	854.2 ± 2.1	859.7	111.6 ± 10.0	120.4	72.4 ± 5.3	72.1	52.2 ± 7.5	54.4
160	875.0 ± 2.7	872.4 ± 1.1	878.7	89.3 ± 6.9	99.1	61.6 ± 3.8	63.3	39.6 ± 5.6	43.2
180	889.6 ± 1.1	888.9 ± 0.7	894.9	74.3 ± 4.0	84.4	54.2 ± 2.7	57.0	30.1 ± 4.1	34.6
200	902.5 ± 0.6	900.2 ± 1.2	909.1	67.3 ± 2.2	73.8	51.1 ± 1.9	52.1	25.6 ± 2.9	27.8

Table S359: Heat capacities at constant volume computed from MC simulations (c_V^{MC}), heat capacities at constant volume obtained from REFPROP[10] (c_V^{REFP}), heat capacities at constant pressure computed from MC simulations (c_P^{MC}), heat capacities at constant pressure obtained from REFPROP[10] (c_P^{REFP}), speed of sound computed from MC simulations (c^{MC}), speed of sound obtained from REFPROP[10] (c^{REFP}), viscosities computed from MD simulations (η^{MD}), and viscosities obtained from REFPROP[10] (η^{REFP}) of CO_2 rich ternary mixture with 3% impurity of Ar and 1% impurity of N_2 at 293 K and pressures ranging from 20 bar to 200 bar.

P/ [bar]	c_V^{MC}/ [J/mol K]	c_V^{REFP}/ [J/mol K]	c_P^{MC}/ [J/mol K]	c_P^{REFP}/ [J/mol K]	c^{MC}/ [m/s]	c^{REFP}/ [m/s]	η^{MD}/ [μPa s]	η^{REFP}/ [μPa s]
20	30.4	30.4	43.6 ± 0.1	44.2	254.4 ± 0.6	252.7	11.5 ± 3.6	15.2
40	33.2	34.0	57.6 ± 0.2	61.0	237.8 ± 0.9	233.3	15.8 ± 0.9	15.9
60	39.1 ± 0.3	42.8	119.1 ± 1.6	148.6	215.7 ± 2.7	204.1	18.8 ± 2.3	18.1
80	39.7 ± 0.3	42.3	158.4 ± 12.6	162.7	410.9 ± 38.0	350.2	66.8 ± 1.5	64.3
100	39.3 ± 0.1	40.7	126.5 ± 5.3	126.7	468.1 ± 21.4	418.1	81.1 ± 10.4	72.2
120	39.0 ± 0.1	40.0	115.7 ± 3.9	112.2	516.8 ± 17.4	465.3	83.3 ± 6.5	77.9
140	38.9 ± 0.1	39.7	109.7 ± 4.3	103.9	542.5 ± 26.5	503.1	88.1 ± 3.2	82.7
160	39.0 ± 0.1	39.5	101.5 ± 3.3	98.4	577.3 ± 24.2	535.1	89.4 ± 1.4	86.9
180	38.9 ± 0.1	39.3	96.2 ± 2.7	94.3	611.9 ± 18.6	563.3	97.3 ± 4.5	90.7
200	38.9 ± 0.1	39.3	94.0 ± 2.3	91.2	630.5 ± 12.7	588.7	95.9 ± 4.4	94.2

Table S360: Densities computed from MC and MD simulations (ρ^{MC} and ρ^{MD}), densities obtained from REFPROP[10] (ρ^{REFP}), isothermal compressibilities computed from MC simulations (β_T^{MC}), isothermal compressibilities obtained from REFPROP[10] (β_T^{REFP}), thermal expansion coefficients computed from MC simulations (α_P^{MC}), thermal expansion coefficients obtained from REFPROP[10] (α_P^{REFP}), Joule Thomson coefficients computed from MC simulations (μ_{JT}^{MC}), and Joule Thomson coefficients obtained from REFPROP[10] (μ_{JT}^{REFP}) of CO_2 rich ternary mixture with 3% impurity of Ar and 1% impurity of N_2 at 313 K and pressures ranging from 20 bar to 200 bar.

P/ [bar]	ρ^{MC}/ [kg/m^3]	ρ^{MD}/ [kg/m^3]	ρ^{REFP}/ [kg/m^3]	β_T^{MC}/ [10^{-5}/bar]	β_T^{REFP}/ [10^{-5}/bar]	α_P^{MC}/ [10^{-4}/K]	α_P^{REFP}/ [10^{-4}/K]	μ_{JT}^{MC}/ [10^{-3} K/bar]	μ_{JT}^{REFP}/ [10^{-3} K/bar]
20	36.3	36.8	36.7	5430.5 ± 11.5	5486.2	41.6 ± 0.1	42.7	852.7 ± 5.3	930.4
40	80.1 ± 0.1	81.4 ± 0.2	81.9	3060.9 ± 29.5	3127.5	57.9 ± 0.5	60.9	865.8 ± 17.3	919.4
60	137.1 ± 0.1	140.6 ± 0.6	142.7	2456.2 ± 22.6	2563.9	89.8 ± 1.0	98.7	852.7 ± 15.0	886.5
80	224.5 ± 0.6	235.0 ± 0.9	241.4	2623.0 ± 137.1	2860.3	183.9 ± 9.8	216.7	788.1 ± 58.7	–
100	454.9 ± 11.3	462.1 ± 7.4	473.3	3636.5 ± 316.0	3025.7	645.7 ± 70.0	539.8	466.2 ± 73.1	452.5
120	635.8 ± 9.4	637.4 ± 7.3	641.0	776.3 ± 140.3	730.5	238.3 ± 32.9	216.0	220.5 ± 40.4	219.9
140	710.5 ± 5.5	704.7 ± 1.9	708.7	334.4 ± 26.6	356.4	134.6 ± 9.0	131.6	136.7 ± 13.6	143.0
160	745.3 ± 1.3	744.6 ± 4.5	750.4	220.9 ± 5.4	233.2	99.7 ± 2.5	99.0	101.6 ± 4.3	104.9
180	774.3 ± 2.3	773.3 ± 1.5	781.1	164.4 ± 6.5	173.2	82.9 ± 2.1	81.4	79.5 ± 3.4	81.3
200	799.2 ± 1.9	797.1 ± 1.8	805.5	129.2 ± 6.3	137.8	70.3 ± 3.5	70.2	62.8 ± 6.1	64.9

Table S361: Heat capacities at constant volume computed from MC simulations (c_V^{MC}), heat capacities at constant volume obtained from REFPROP[10] (c_V^{REFP}), heat capacities at constant pressure computed from MC simulations (c_P^{MC}), heat capacities at constant pressure obtained from REFPROP[10] (c_P^{REFP}), speed of sound computed from MC simulations (c^{MC}), speed of sound obtained from REFPROP[10] (c^{REFP}), viscosities computed from MD simulations (η^{MD}), and viscosities obtained from REFPROP[10] (η^{REFP}) of CO_2 rich ternary mixture with 3% impurity of Ar and 1% impurity of N_2 at 313 K and pressures ranging from 20 bar to 200 bar.

P/ [bar]	c_V^{MC}/ [J/mol K]	c_V^{REFP}/ [J/mol K]	c_P^{MC}/ [J/mol K]	c_P^{REFP}/ [J/mol K]	c^{MC}/ [m/s]	c^{REFP}/ [m/s]	η^{MD}/ [μPa s]	η^{REFP}/ [μPa s]
20	30.7	30.7	42.7	43.0	265.9 ± 0.3	264.1	2.1 ± 2.5	16.2
40	32.5	32.8	51.1 ± 0.1	52.7	253.5 ± 1.3	250.2	16.9 ± 2.1	16.8
60	34.9 ± 0.1	35.8	67.8 ± 0.4	72.2	240.1 ± 1.4	234.9	17.6 ± 1.8	17.9
80	38.6 ± 0.2	–	117.6 ± 4.3	–	227.3 ± 7.3	–	20.8 ± 2.2	20.8
100	42.1 ± 0.4	46.0	396.5 ± 41.5	324.5	238.8 ± 16.5	221.9	32.1 ± 2.7	33.4
120	40.1 ± 0.3	42.3	201.5 ± 18.1	178.7	319.1 ± 32.3	300.3	46.2 ± 3.0	49.0
140	39.3 ± 0.3	40.7	144.6 ± 6.6	134.5	393.4 ± 18.2	361.7	57.4 ± 2.7	57.4
160	38.9 ± 0.2	40.0	122.5 ± 2.6	116.5	437.3 ± 7.2	408.2	67.6 ± 6.1	63.4
180	38.7 ± 0.2	39.6	113.2 ± 1.2	106.5	479.5 ± 10.0	446.1	68.1 ± 4.3	68.3
200	38.8 ± 0.1	39.3	104.7 ± 3.5	100.0	511.3 ± 15.1	478.6	73.8 ± 2.2	72.5

S17 Data of thermodynamic and transport properties of quaternary systems

This section shows the thermodynamic and transport properties with uncertainties computed from Monte Carlo (MC) and Molecular Dynamics (MD) simulations, respectively, for CO_2 rich quaternary mixtures with impurities (Ar, N_2, H_2, listed in Table S362, where compositions are in mole%. The thermodynamic properties computed from MC simulations are (1) densities (ρ), (2) isothermal compressibilities at constant temperature (β_T), (3) thermal expansion coefficients (α_P), (4) heat capacities at constant volume (c_V), (5) heat capacities at constant pressure (c_P), (6) Joule-Thomson Coefficients (μ_{JT}), and (7) speed of sound (c). Transport property, (8) viscosities (η) are computed from MD simulations. Densities computed from MD simulations are also reported in this section. The thermodynamic and transport properties are computed at temperatures 253 K, 273 K, 293 K, and 313K, and pressure ranging from 20 bar to 200 bar in intervals of 20 bar. For each condition (concentration, temperature, and pressure), 10 independent simulations are performed, and each simulation starts with a different initial configuration. These 10 simulations are divided into 5 blocks from which average values and uncertainties of thermodynamic and transport properties are calculated as the standard deviation of 5 blocks. The thermodynamic and transport properties uncertainty values with more than one significant decimal figure are not shown. Properties computed from molecular simulations are compared with data calculated from the GERG-2008 EoS,[30] which are obtained from REFPROP.[10] At conditions close to the critical point, REFPROP[10] fails to calculate heat capacities at constant volume (c_V), heat capacities at constant pressure (c_P), Joule-Thomson Coefficients (μ_{JT}), and speed of sound (c). A similar issue was also encountered during simulations, so the unavailable EoS data and simulation data are represented by ”-”.

Table S362: Compositions of CO_2 rich quaternary mixtures in mole% with impurities (Ar, N_2, H_2, and CH_4) for which the thermodynamics and transport properties are computed at temperatures 253 K, 273 K, 293 K, and 313K, and pressure ranging from 20 bar to 200 bar in intervals of 20 bar. Component with 0 mole% are denoted by -.

No.	Mixture - ID	CO_2 (mole%)	Ar (mole%)	N_2 (mole%)	H_2 (mole%)	CH_4 (mole%)
1	QM-96-1	96	-	1	1	2
2	QM-96-2	96	-	1	2	1
3	QM-96-3	96	-	2	1	1
4	QM-96-4	96	2	-	1	1
5	QM-96-5	96	1	-	1	2
6	QM-96-6	96	1	-	2	1
7	QM-96-7	96	1	1	-	2
8	QM-96-8	96	1	2	-	1
9	QM-96-9	96	2	1	-	1
10	QM-96-10	96	1	1	2	-
11	QM-96-11	96	1	2	1	-
12	QM-96-12	96	2	1	1	-

S17.1 Data of thermodynamics and transport properties of CO_2 rich quaternary mixture with 1 mole% impurity of N_2, 1 mole% impurity of H_2, and 2 mole% impurity of CH_4

Table S363: Densities computed from MC and MD simulations (ρ^{MC} and ρ^{MD}), densities obtained from REFPROP[10] (ρ^{REFP}), isothermal compressibilities computed from MC simulations (β_T^{MC}), isothermal compressibilities obtained from REFPROP[10] (β_T^{REFP}), thermal expansion coefficients computed from MC simulations (α_P^{MC}), thermal expansion coefficients obtained from REFPROP[10] (α_P^{REFP}), Joule Thomson coefficients computed from MC simulations ($\mu_{\mathrm{JT}}^{\mathrm{MC}}$), and Joule Thomson coefficients obtained from REFPROP[10] ($\mu_{\mathrm{JT}}^{\mathrm{REFP}}$) of CO_2 rich quaternary mixture with 2% impurity of CH_4, 1% impurity of N_2 and 1% impurity of H_2 at 253 K and pressures ranging from 20 bar to 200 bar.

P/ [bar]	ρ^{MC}/ [kg/m^3]	ρ^{MD}/ [kg/m^3]	ρ^{REFP}/ [kg/m^3]	β_T^{MC}/ [10^{-5}/bar]	β_T^{REFP}/ [10^{-5}/bar]	α_P^{MC}/ [10^{-4}/K]	α_P^{REFP}/ [10^{-4}/K]	$\mu_{\mathrm{JT}}^{\mathrm{MC}}$/ [$10^{-3}$ K/bar]	$\mu_{\mathrm{JT}}^{\mathrm{REFP}}$/ [$10^{-3}$ K/bar]
20	49.1	49.8 ± 0.1	50.6	6272.3 ± 87.5	6526.1	74.0 ± 1.0	82.0	1496.7 ± 43.4	1654.6
40	986.6 ± 0.9	984.6 ± 1.2	860.1	42.6 ± 1.0	686.0	45.9 ± 0.7	373.5	7.4 ± 0.8	–
60	994.1 ± 1.2	993.7 ± 1.0	991.9	42.0 ± 0.5	47.1	46.1 ± 0.6	46.9	7.6 ± 0.7	8.9
80	1002.0 ± 1.1	1000.2 ± 1.1	1000.9	38.8 ± 0.7	42.9	43.8 ± 0.9	44.1	5.0 ± 1.0	5.5
100	1010.2 ± 1.6	1008.8 ± 0.9	1009.1	36.1 ± 1.7	39.5	41.8 ± 1.6	41.7	2.7 ± 1.9	2.7
120	1017.2 ± 1.1	1015.4 ± 0.4	1016.8	33.2 ± 2.3	36.6	39.7 ± 3.1	39.7	0.2 ± 3.7	0.2
140	1024.4 ± 0.9	1021.9 ± 0.8	1024.1	31.3 ± 0.7	34.2	38.1 ± 1.0	38.0	-1.7 ± 1.2	-1.9
160	1029.2 ± 1.1	1028.5 ± 0.9	1030.9	29.5 ± 0.7	32.1	36.8 ± 0.8	36.5	-3.2 ± 0.9	-3.9
180	1034.9 ± 0.6	1034.3 ± 0.9	1037.3	27.6 ± 1.1	30.3	35.0 ± 1.5	35.1	-5.5 ± 1.8	-5.6
200	1041.6 ± 1.2	1039.7 ± 0.8	1043.4	26.1 ± 0.5	28.7	33.5 ± 0.6	33.9	-7.3 ± 0.7	-7.1

Table S364: Heat capacities at constant volume computed from MC simulations (c_V^{MC}), heat capacities at constant volume obtained from REFPROP[10] (c_V^{REFP}), heat capacities at constant pressure computed from MC simulations (c_P^{MC}), heat capacities at constant pressure obtained from REFPROP[10] (c_P^{REFP}), speed of sound computed from MC simulations (c^{MC}), speed of sound obtained from REFPROP[10] (c^{REFP}), viscosities computed from MD simulations (η^{MD}), and viscosities obtained from REFPROP[10] (η^{REFP}) of CO_2 rich quaternary mixture with 2% impurity of CH_4, 1% impurity of N_2 and 1% impurity of H_2 at 253 K and pressures ranging from 20 bar to 200 bar.

P/ [bar]	c_V^{MC}/ [J/mol K]	c_V^{REFP}/ [J/mol K]	c_P^{MC}/ [J/mol K]	c_P^{REFP}/ [J/mol K]	c^{MC}/ [m/s]	c^{REFP}/ [m/s]	η^{MD}/ [μPa s]	η^{REFP}/ [μPa s]
20	31.6	33.0	50.9 ± 0.2	55.1	228.8 ± 1.7	224.9	13.3 ± 1.7	12.9
40	40.1 ± 0.2	–	94.8 ± 0.7	–	749.7 ± 9.2	–	137.6 ± 11.1	86.6
60	40.3 ± 0.2	40.2	95.4 ± 1.2	91.4	752.5 ± 6.6	697.2	134.8 ± 3.1	128.6
80	40.3 ± 0.3	40.2	94.2 ± 1.2	89.4	774.8 ± 9.0	719.4	137.5 ± 1.9	132.4
100	40.2 ± 0.3	40.2	92.3 ± 1.6	87.7	793.9 ± 19.9	739.8	166.1 ± 19.5	136.1
120	40.4 ± 0.2	40.2	91.0 ± 4.7	86.2	817.4 ± 35.3	758.8	177.0 ± 34.3	139.6
140	40.4 ± 0.2	40.2	89.5 ± 1.7	85.0	831.7 ± 12.2	776.7	153.3 ± 9.6	143.0
160	40.4 ± 0.2	40.3	89.0 ± 1.3	83.9	851.6 ± 11.5	793.5	159.1 ± 15.5	146.3
180	40.8 ± 0.3	40.3	86.8 ± 2.2	82.9	863.5 ± 20.9	809.5	158.1 ± 11.4	149.5
200	40.6 ± 0.1	40.3	85.3 ± 0.7	82.0	880.0 ± 9.7	824.8	158.3 ± 6.7	152.7

Table S365: Densities computed from MC and MD simulations (ρ^{MC} and ρ^{MD}), densities obtained from REFPROP[10] (ρ^{REFP}), isothermal compressibilities computed from MC simulations (β_T^{MC}), isothermal compressibilities obtained from REFPROP[10] (β_T^{REFP}), thermal expansion coefficients computed from MC simulations (α_P^{MC}), thermal expansion coefficients obtained from REFPROP[10] (α_P^{REFP}), Joule Thomson coefficients computed from MC simulations ($\mu_{\mathrm{JT}}^{\mathrm{MC}}$), and Joule Thomson coefficients obtained from REFPROP[10] ($\mu_{\mathrm{JT}}^{\mathrm{REFP}}$) of CO_2 rich quaternary mixture with 2% impurity of CH_4, 1% impurity of N_2 and 1% impurity of H_2 at 273 K and pressures ranging from 20 bar to 200 bar.

P/ [bar]	ρ^{MC}/ [kg/m^3]	ρ^{MD}/ [kg/m^3]	ρ^{REFP}/ [kg/m^3]	β_T^{MC}/ [10^{-5}/bar]	β_T^{REFP}/ [10^{-5}/bar]	α_P^{MC}/ [10^{-4}/K]	α_P^{REFP}/ [10^{-4}/K]	$\mu_{\mathrm{JT}}^{\mathrm{MC}}$/ [$10^{-3}$ K/bar]	$\mu_{\mathrm{JT}}^{\mathrm{REFP}}$/ [$10^{-3}$ K/bar]
20	43.1	43.8 ± 0.1	44.0	5780.5 ± 9.3	5944.8	56.6 ± 0.1	60.4	1186.3 ± 3.1	1332.9
40	108.7 ± 0.2	865.9 ± 0.9	256.8	4423.2 ± 119.9	−2887.2	135.6 ± 4.6	−469.1	1303.3 ± 69.4	–
60	888.1 ± 1.1	887.0 ± 2.7	881.8	92.1 ± 4.7	113.4	70.0 ± 3.7	76.8	39.7 ± 4.6	48.7
80	903.5 ± 1.3	903.4 ± 2.0	899.9	81.8 ± 4.1	90.8	65.4 ± 2.8	65.9	34.5 ± 3.5	37.2
100	917.9 ± 0.4	915.4 ± 0.8	915.0	68.5 ± 4.2	76.4	58.2 ± 3.3	58.6	26.7 ± 4.2	28.8
120	928.4 ± 1.5	928.1 ± 1.3	928.1	59.8 ± 5.9	66.3	51.9 ± 4.2	53.2	19.9 ± 5.6	22.3
140	940.3 ± 2.6	938.7 ± 0.6	939.7	53.8 ± 3.8	58.7	49.6 ± 3.3	49.1	16.7 ± 4.4	17.1
160	949.2 ± 0.8	947.7 ± 0.6	950.2	48.4 ± 4.5	52.9	45.8 ± 3.9	45.9	12.1 ± 5.2	12.8
180	958.4 ± 0.8	956.5 ± 1.3	959.9	42.7 ± 3.3	48.2	41.9 ± 3.1	43.1	7.2 ± 4.2	9.1
200	965.8 ± 1.5	964.3 ± 1.1	968.8	40.4 ± 2.4	44.3	40.9 ± 2.5	40.9	5.7 ± 3.4	6.0

Table S366: Heat capacities at constant volume computed from MC simulations (c_V^{MC}), heat capacities at constant volume obtained from REFPROP[10] (c_V^{REFP}), heat capacities at constant pressure computed from MC simulations (c_P^{MC}), heat capacities at constant pressure obtained from REFPROP[10] (c_P^{REFP}), speed of sound computed from MC simulations (c^{MC}), speed of sound obtained from REFPROP[10] (c^{REFP}), viscosities computed from MD simulations (η^{MD}), and viscosities obtained from REFPROP[10] (η^{REFP}) of CO_2 rich quaternary mixture with 2% impurity of CH_4, 1% impurity of N_2 and 1% impurity of H_2 at 273 K and pressures ranging from 20 bar to 200 bar.

P/ [bar]	c_V^{MC}/ [J/mol K]	c_V^{REFP}/ [J/mol K]	c_P^{MC}/ [J/mol K]	c_P^{REFP}/ [J/mol K]	c^{MC}/ [m/s]	c^{REFP}/ [m/s]	η^{MD}/ [μPa s]	η^{REFP}/ [μPa s]
20	30.7	31.1	45.8	47.4	244.4 ± 0.2	241.4	11.8 ± 1.9	14.0
40	36.8 ± 0.2	–	81.8 ± 2.2	–	214.8 ± 4.1	–	92.4 ± 2.9	19.0
60	39.6 ± 0.2	40.5	110.7 ± 4.0	109.5	584.4 ± 18.3	519.9	97.6 ± 1.6	91.1
80	39.7 ± 0.1	40.2	108.1 ± 2.8	102.3	606.9 ± 17.2	558.0	102.1 ± 3.0	95.9
100	39.6 ± 0.2	40.0	103.1 ± 3.7	97.5	644.0 ± 23.0	590.1	101.2 ± 4.0	100.2
120	39.8 ± 0.1	39.9	96.8 ± 4.1	93.9	662.2 ± 35.4	618.1	110.5 ± 2.3	104.1
140	39.8 ± 0.1	39.9	96.6 ± 4.0	91.1	693.0 ± 28.2	643.3	115.8 ± 7.4	107.8
160	39.7 ± 0.2	39.8	93.4 ± 4.5	88.8	716.0 ± 37.5	666.3	113.9 ± 5.8	111.2
180	39.6 ± 0.1	39.8	90.1 ± 3.8	86.9	746.4 ± 32.6	687.5	170.9 ± 59.6	114.5
200	39.8 ± 0.1	39.8	90.0 ± 3.2	85.4	760.9 ± 26.3	707.2	123.9 ± 5.0	117.7

Table S367: Densities computed from MC and MD simulations (ρ^{MC} and ρ^{MD}), densities obtained from REFPROP[10] (ρ^{REFP}), isothermal compressibilities computed from MC simulations (β_T^{MC}), isothermal compressibilities obtained from REFPROP[10] (β_T^{REFP}), thermal expansion coefficients computed from MC simulations (α_P^{MC}), thermal expansion coefficients obtained from REFPROP[10] (α_P^{REFP}), Joule Thomson coefficients computed from MC simulations ($\mu_{\mathrm{JT}}^{\mathrm{MC}}$), and Joule Thomson coefficients obtained from REFPROP[10] ($\mu_{\mathrm{JT}}^{\mathrm{REFP}}$) of CO_2 rich quaternary mixture with 2% impurity of CH_4, 1% impurity of N_2 and 1% impurity of H_2 at 293 K and pressures ranging from 20 bar to 200 bar.

P/ [bar]	ρ^{MC}/ [kg/m^3]	ρ^{MD}/ [kg/m^3]	ρ^{REFP}/ [kg/m^3]	β_T^{MC}/ [10^{-5}/bar]	β_T^{REFP}/ [10^{-5}/bar]	α_P^{MC}/ [10^{-4}/K]	α_P^{REFP}/ [10^{-4}/K]	$\mu_{\mathrm{JT}}^{\mathrm{MC}}$/ [$10^{-3}$ K/bar]	$\mu_{\mathrm{JT}}^{\mathrm{REFP}}$/ [$10^{-3}$ K/bar]
20	38.9	39.5 ± 0.1	39.5	5598.3 ± 22.7	5661.7	48.0 ± 0.3	49.6	1016.9 ± 21.6	1103.0
40	89.7 ± 0.1	91.4 ± 0.2	92.8	3419.9 ± 48.6	3512.4	78.1 ± 1.6	84.4	1050.4 ± 39.9	1098.0
60	173.5 ± 0.9	182.0 ± 4.0	190.4	3670.9 ± 121.2	4461.6	198.0 ± 7.0	274.8	1011.0 ± 51.8	1021.5
80	754.5 ± 5.4	746.6 ± 3.6	735.7	296.5 ± 31.9	433.9	141.4 ± 10.4	175.6	115.8 ± 12.2	147.2
100	788.1 ± 1.5	787.5 ± 2.6	782.2	211.5 ± 33.3	227.1	112.4 ± 13.5	110.0	91.5 ± 16.9	96.2
120	815.7 ± 2.7	814.4 ± 2.0	812.2	140.1 ± 12.0	158.0	83.9 ± 5.4	85.6	65.2 ± 7.4	71.0
140	836.6 ± 0.9	835.4 ± 0.6	835.0	100.3 ± 4.9	122.5	65.5 ± 2.7	72.0	45.3 ± 4.1	54.9
160	855.0 ± 1.0	851.2 ± 0.7	853.7	88.8 ± 2.6	100.5	61.7 ± 2.0	63.1	39.6 ± 3.1	43.5
180	867.3 ± 2.5	864.8 ± 0.8	869.7	80.4 ± 4.1	85.5	57.6 ± 2.3	56.8	34.3 ± 3.5	34.8
200	881.3 ± 1.2	878.6 ± 0.5	883.7	70.0 ± 3.3	74.6	52.9 ± 2.1	52.0	27.8 ± 3.2	27.8

Table S368: Heat capacities at constant volume computed from MC simulations (c_V^{MC}), heat capacities at constant volume obtained from REFPROP[10] (c_V^{REFP}), heat capacities at constant pressure computed from MC simulations (c_P^{MC}), heat capacities at constant pressure obtained from REFPROP[10] (c_P^{REFP}), speed of sound computed from MC simulations (c^{MC}), speed of sound obtained from REFPROP[10] (c^{REFP}), viscosities computed from MD simulations (η^{MD}), and viscosities obtained from REFPROP[10] (η^{REFP}) of CO_2 rich quaternary mixture with 2% impurity of CH_4, 1% impurity of N_2 and 1% impurity of H_2 at 293 K and pressures ranging from 20 bar to 200 bar.

P/ [bar]	c_V^{MC}/ [J/mol K]	c_V^{REFP}/ [J/mol K]	c_P^{MC}/ [J/mol K]	c_P^{REFP}/ [J/mol K]	c^{MC}/ [m/s]	c^{REFP}/ [m/s]	η^{MD}/ [μPa s]	η^{REFP}/ [μPa s]
20	30.7	30.9	44.0 ± 0.1	44.7	256.4 ± 0.6	254.6	3.8 ± 5.6	14.9
40	33.6	34.5	58.6 ± 0.7	62.0	238.4 ± 2.2	234.7	17.0 ± 3.1	15.6
60	39.6 ± 0.2	43.8	117.4 ± 3.2	155.4	215.9 ± 4.7	204.5	17.8 ± 1.7	17.8
80	40.4 ± 0.3	42.8	154.3 ± 6.3	164.1	412.9 ± 23.8	346.6	66.7 ± 4.3	62.0
100	39.7 ± 0.2	41.1	136.3 ± 9.0	126.7	453.7 ± 38.7	416.6	74.1 ± 3.1	69.8
120	39.6 ± 0.1	40.4	117.5 ± 3.9	112.1	509.5 ± 23.5	464.9	79.8 ± 2.5	75.4
140	39.4 ± 0.2	40.0	104.2 ± 2.6	103.7	561.7 ± 15.4	503.4	83.3 ± 2.1	80.1
160	39.3 ± 0.1	39.8	102.3 ± 2.4	98.2	585.7 ± 11.0	536.0	89.2 ± 2.9	84.2
180	39.4 ± 0.1	39.7	99.4 ± 2.3	94.1	601.9 ± 16.7	564.7	115.4 ± 29.4	87.9
200	39.3 ± 0.1	39.6	96.5 ± 2.0	91.0	630.4 ± 16.3	590.4	97.4 ± 5.8	91.3

Table S369: Densities computed from MC and MD simulations (ρ^{MC} and ρ^{MD}), densities obtained from REFPROP[10] (ρ^{REFP}), isothermal compressibilities computed from MC simulations (β_T^{MC}), isothermal compressibilities obtained from REFPROP[10] (β_T^{REFP}), thermal expansion coefficients computed from MC simulations (α_P^{MC}), thermal expansion coefficients obtained from REFPROP[10] (α_P^{REFP}), Joule Thomson coefficients computed from MC simulations ($\mu_{\mathrm{JT}}^{\mathrm{MC}}$), and Joule Thomson coefficients obtained from REFPROP[10] ($\mu_{\mathrm{JT}}^{\mathrm{REFP}}$) of CO_2 rich quaternary mixture with 2% impurity of CH_4, 1% impurity of N_2 and 1% impurity of H_2 at 313 K and pressures ranging from 20 bar to 200 bar.

P/ [bar]	ρ^{MC}/ [kg/m^3]	ρ^{MD}/ [kg/m^3]	ρ^{REFP}/ [kg/m^3]	β_T^{MC}/ [10^{-5}/bar]	β_T^{REFP}/ [10^{-5}/bar]	α_P^{MC}/ [10^{-4}/K]	α_P^{REFP}/ [10^{-4}/K]	$\mu_{\mathrm{JT}}^{\mathrm{MC}}$/ [$10^{-3}$ K/bar]	$\mu_{\mathrm{JT}}^{\mathrm{REFP}}$/ [$10^{-3}$ K/bar]
20	35.6	36.1 ± 0.1	36.0	5425.2 ± 10.7	5490.8	41.6 ± 0.1	42.8	845.6 ± 9.2	929.3
40	78.5	79.8 ± 0.3	80.5	3040.1 ± 29.9	3135.0	57.5 ± 0.7	61.3	848.6 ± 24.6	919.2
60	134.4 ± 0.2	137.4 ± 0.5	140.4	2478.4 ± 33.3	2577.6	90.6 ± 1.4	100.0	856.8 ± 21.1	885.7
80	220.9 ± 0.5	233.4 ± 3.3	238.8	2728.7 ± 34.1	2893.6	191.7 ± 2.5	222.3	798.2 ± 15.5	–
100	430.5 ± 3.6	447.8 ± 4.7	464.8	3417.2 ± 420.9	2871.9	582.9 ± 64.8	513.8	476.8 ± 71.5	446.2
120	624.1 ± 4.9	620.1 ± 5.4	623.2	727.2 ± 45.8	725.8	225.3 ± 11.6	211.9	215.1 ± 15.3	220.2
140	689.7 ± 4.2	686.1 ± 3.6	688.9	347.5 ± 38.9	357.4	135.5 ± 12.3	130.2	140.0 ± 18.4	143.4
160	728.1 ± 2.1	725.1 ± 2.0	729.6	228.0 ± 16.0	234.3	102.9 ± 5.7	98.1	103.9 ± 8.8	105.0
180	757.7 ± 1.9	754.3 ± 0.6	759.6	162.2 ± 10.4	174.1	81.4 ± 5.7	80.7	78.1 ± 9.8	81.2
200	778.2 ± 1.4	777.9 ± 1.5	783.5	129.1 ± 1.9	138.5	69.2 ± 1.1	69.6	62.2 ± 2.0	64.8

Table S370: Heat capacities at constant volume computed from MC simulations (c_V^{MC}), heat capacities at constant volume obtained from REFPROP[10] (c_V^{REFP}), heat capacities at constant pressure computed from MC simulations (c_P^{MC}), heat capacities at constant pressure obtained from REFPROP[10] (c_P^{REFP}), speed of sound computed from MC simulations (c^{MC}), speed of sound obtained from REFPROP[10] (c^{REFP}), viscosities computed from MD simulations (η^{MD}), and viscosities obtained from REFPROP[10] (η^{REFP}) of CO_2 rich quaternary mixture with 2% impurity of CH_4, 1% impurity of N_2 and 1% impurity of H_2 at 313 K and pressures ranging from 20 bar to 200 bar.

P/ [bar]	c_V^{MC}/ [J/mol K]	c_V^{REFP}/ [J/mol K]	c_P^{MC}/ [J/mol K]	c_P^{REFP}/ [J/mol K]	c^{MC}/ [m/s]	c^{REFP}/ [m/s]	η^{MD}/ [μPa s]	η^{REFP}/ [μPa s]
20	31.1	31.1	43.1 ± 0.1	43.5	268.1 ± 0.3	266.1	0.9 ± 0.1	15.9
40	32.9	33.3	51.4 ± 0.3	53.3	256.1 ± 1.5	251.9	18.0 ± 0.9	16.5
60	35.3 ± 0.1	36.3	68.4 ± 0.6	73.4	241.2 ± 1.9	236.2	18.0 ± 0.9	17.6
80	39.2 ± 0.1	–	121.6 ± 1.4	–	226.7 ± 2.0	–	20.1 ± 0.9	20.5
100	42.8 ± 0.7	46.4	360.4 ± 33.4	311.7	239.3 ± 18.6	224.4	34.9 ± 8.0	32.9
120	40.5 ± 0.2	42.7	193.4 ± 7.4	175.9	324.3 ± 12.0	301.8	50.8 ± 8.7	47.7
140	39.7 ± 0.3	41.1	143.9 ± 7.9	133.4	388.8 ± 24.3	363.1	54.3 ± 1.0	55.8
160	39.3 ± 0.1	40.4	126.0 ± 3.7	115.9	439.5 ± 16.7	409.8	66.1 ± 2.0	61.7
180	39.4 ± 0.3	39.9	112.1 ± 5.5	106.1	481.1 ± 19.5	448.1	68.9 ± 8.1	66.4
200	39.2 ± 0.2	39.7	103.4 ± 1.2	99.6	512.7 ± 5.0	480.9	71.8 ± 3.6	70.5

S17.2 Data of thermodynamics and transport properties of CO_2 rich quaternary mixture with 1 mole% impurity of N_2, 2 mole% impurity of H_2, and 1 mole% impurity of CH_4

Table S371: Densities computed from MC and MD simulations (ρ^{MC} and ρ^{MD}), densities obtained from REFPROP[10] (ρ^{REFP}), isothermal compressibilities computed from MC simulations (β_T^{MC}), isothermal compressibilities obtained from REFPROP[10] (β_T^{REFP}), thermal expansion coefficients computed from MC simulations (α_P^{MC}), thermal expansion coefficients obtained from REFPROP[10] (α_P^{REFP}), Joule Thomson coefficients computed from MC simulations (μ_{JT}^{MC}), and Joule Thomson coefficients obtained from REFPROP[10] (μ_{JT}^{REFP}) of CO_2 rich quaternary mixture with 2% impurity of H_2, 1% impurity of N_2 and 1% impurity of CH_4 at 253 K and pressures ranging from 20 bar to 200 bar.

P/ [bar]	ρ^{MC}/ [kg/m^3]	ρ^{MD}/ [kg/m^3]	ρ^{REFP}/ [kg/m^3]	β_T^{MC}/ [10^{-5}/bar]	β_T^{REFP}/ [10^{-5}/bar]	α_P^{MC}/ [10^{-4}/K]	α_P^{REFP}/ [10^{-4}/K]	μ_{JT}^{MC}/ [10^{-3} K/bar]	μ_{JT}^{REFP}/ [10^{-3} K/bar]
20	48.8 ± 0.1	49.5 ± 0.1	50.3	6275.9 ± 21.3	6487.6	73.8 ± 0.4	80.8	1496.0 ± 16.6	1635.2
40	982.0 ± 3.0	979.7 ± 1.2	714.8	45.9 ± 3.2	−117.3	48.1 ± 2.8	18.3	9.9 ± 3.2	–
60	990.8 ± 1.7	989.3 ± 0.5	972.5	43.1 ± 1.7	55.4	46.7 ± 1.8	52.1	8.2 ± 2.1	–
80	999.1 ± 1.3	997.5 ± 1.0	993.7	38.6 ± 2.4	44.1	42.9 ± 2.7	44.7	4.0 ± 3.2	6.3
100	1006.3 ± 1.2	1004.8 ± 1.1	1002.1	38.8 ± 2.4	40.4	44.1 ± 2.8	42.2	5.2 ± 3.2	3.3
120	1013.1 ± 0.6	1012.6 ± 0.7	1010.0	34.3 ± 1.3	37.4	40.2 ± 1.5	40.1	0.8 ± 1.7	0.8
140	1021.5 ± 0.8	1018.8 ± 0.6	1017.3	32.4 ± 1.1	34.9	38.9 ± 1.2	38.3	-0.7 ± 1.4	−1.5
160	1026.3 ± 0.9	1025.2 ± 0.9	1024.2	30.2 ± 0.6	32.7	37.3 ± 1.0	36.8	-2.7 ± 1.2	−3.5
180	1033.7 ± 1.4	1031.4 ± 0.5	1030.7	28.3 ± 0.4	30.8	35.7 ± 0.5	35.4	-4.6 ± 0.6	−5.2
200	1038.9 ± 1.4	1037.2 ± 0.8	1036.9	26.8 ± 1.0	29.2	34.1 ± 1.2	34.2	-6.6 ± 1.4	−6.8

Table S372: Heat capacities at constant volume computed from MC simulations (c_V^{MC}), heat capacities at constant volume obtained from REFPROP[10] (c_V^{REFP}), heat capacities at constant pressure computed from MC simulations (c_P^{MC}), heat capacities at constant pressure obtained from REFPROP[10] (c_P^{REFP}), speed of sound computed from MC simulations (c^{MC}), speed of sound obtained from REFPROP[10] (c^{REFP}), viscosities computed from MD simulations (η^{MD}), and viscosities obtained from REFPROP[10] (η^{REFP}) of CO_2 rich quaternary mixture with 2% impurity of H_2, 1% impurity of N_2 and 1% impurity of CH_4 at 253 K and pressures ranging from 20 bar to 200 bar.

P/ [bar]	c_V^{MC}/ [J/mol K]	c_V^{REFP}/ [J/mol K]	c_P^{MC}/ [J/mol K]	c_P^{REFP}/ [J/mol K]	c^{MC}/ [m/s]	c^{REFP}/ [m/s]	η^{MD}/ [μPa s]	η^{REFP}/ [μPa s]
20	31.5	32.6	50.7 ± 0.2	54.3	228.9 ± 0.6	225.8	12.4 ± 3.4	13.0
40	40.3 ± 0.4	–	95.7 ± 2.8	–	726.5 ± 27.8	–	133.2 ± 7.3	61.8
60	40.1 ± 0.3	–	95.2 ± 2.1	–	745.0 ± 17.2	–	135.5 ± 4.2	120.8
80	40.2 ± 0.2	40.1	92.0 ± 3.3	89.4	771.1 ± 28.0	713.5	140.1 ± 5.1	129.4
100	40.4 ± 0.2	40.1	94.4 ± 4.0	87.7	773.6 ± 28.7	734.2	143.8 ± 2.8	133.0
120	40.2 ± 0.3	40.1	90.5 ± 1.6	86.2	804.9 ± 17.3	753.5	145.2 ± 9.9	136.5
140	40.5 ± 0.2	40.1	89.7 ± 1.7	84.9	817.5 ± 16.1	771.6	154.6 ± 11.7	139.9
160	40.5 ± 0.2	40.2	89.0 ± 1.6	83.8	842.8 ± 12.0	788.7	151.5 ± 11.2	143.2
180	40.4 ± 0.2	40.2	87.4 ± 0.9	82.8	859.0 ± 7.9	804.8	160.5 ± 11.9	146.4
200	40.4 ± 0.2	40.2	85.6 ± 1.4	81.9	873.2 ± 18.4	820.2	161.6 ± 12.8	149.5

Table S373: Densities computed from MC and MD simulations (ρ^{MC} and ρ^{MD}), densities obtained from REFPROP[10] (ρ^{REFP}), isothermal compressibilities computed from MC simulations (β_T^{MC}), isothermal compressibilities obtained from REFPROP[10] (β_T^{REFP}), thermal expansion coefficients computed from MC simulations (α_P^{MC}), thermal expansion coefficients obtained from REFPROP[10] (α_P^{REFP}), Joule Thomson coefficients computed from MC simulations (μ_{JT}^{MC}), and Joule Thomson coefficients obtained from REFPROP[10] (μ_{JT}^{REFP}) of CO_2 rich quaternary mixture with 2% impurity of H_2, 1% impurity of N_2 and 1% impurity of CH_4 at 273 K and pressures ranging from 20 bar to 200 bar.

P/ [bar]	ρ^{MC}/ [kg/m^3]	ρ^{MD}/ [kg/m^3]	ρ^{REFP}/ [kg/m^3]	β_T^{MC}/ [10^{-5}/bar]	β_T^{REFP}/ [10^{-5}/bar]	α_P^{MC}/ [10^{-4}/K]	α_P^{REFP}/ [10^{-4}/K]	μ_{JT}^{MC}/ [10^{-3} K/bar]	μ_{JT}^{REFP}/ [10^{-3} K/bar]
20	42.9	43.5 ± 0.1	43.8	5798.0 ± 37.1	5928.8	56.8 ± 0.5	59.9	1197.5 ± 27.2	1319.4
40	107.3 ± 0.2	857.6 ± 1.6	234.4	4276.8 ± 96.2	-5357.2	130.4 ± 2.8	-661.2	1274.9 ± 40.2	–
60	881.0 ± 0.5	879.8 ± 0.5	813.8	105.8 ± 11.6	303.0	78.1 ± 8.3	156.6	47.0 ± 9.9	–
80	897.4 ± 2.9	896.0 ± 1.0	891.8	81.9 ± 9.2	94.6	64.9 ± 6.4	67.4	34.4 ± 8.0	39.1
100	912.0 ± 1.4	909.6 ± 0.7	907.3	69.6 ± 3.3	79.1	58.2 ± 2.8	59.7	27.0 ± 3.6	30.3
120	923.4 ± 1.6	922.7 ± 0.9	920.7	61.4 ± 1.0	68.3	52.8 ± 1.2	54.1	21.0 ± 1.6	23.5
140	935.2 ± 1.5	933.2 ± 1.5	932.6	54.7 ± 4.2	60.3	49.1 ± 3.7	49.8	16.4 ± 5.0	18.1
160	945.5 ± 2.1	943.7 ± 0.4	943.3	48.7 ± 1.4	54.2	45.4 ± 0.9	46.4	11.8 ± 1.3	13.6
180	953.5 ± 1.1	952.0 ± 1.6	953.1	45.4 ± 1.1	49.2	43.6 ± 1.0	43.6	9.4 ± 1.3	9.8
200	962.5 ± 0.8	961.2 ± 1.0	962.1	42.8 ± 1.2	45.2	42.2 ± 1.3	41.2	7.5 ± 1.7	6.6

Table S374: Heat capacities at constant volume computed from MC simulations (c_V^{MC}), heat capacities at constant volume obtained from REFPROP[10] (c_V^{REFP}), heat capacities at constant pressure computed from MC simulations (c_P^{MC}), heat capacities at constant pressure obtained from REFPROP[10] (c_P^{REFP}), speed of sound computed from MC simulations (c^{MC}), speed of sound obtained from REFPROP[10] (c^{REFP}), viscosities computed from MD simulations (η^{MD}), and viscosities obtained from REFPROP[10] (η^{REFP}) of CO_2 rich quaternary mixture with 2% impurity of H_2, 1% impurity of N_2 and 1% impurity of CH_4 at 273 K and pressures ranging from 20 bar to 200 bar.

P/ [bar]	c_V^{MC}/ [J/mol K]	c_V^{REFP}/ [J/mol K]	c_P^{MC}/ [J/mol K]	c_P^{REFP}/ [J/mol K]	c^{MC}/ [m/s]	c^{REFP}/ [m/s]	η^{MD}/ [μPa s]	η^{REFP}/ [μPa s]
20	30.7	30.9	45.7 ± 0.1	47.1	244.8 ± 0.9	242.1	13.0 ± 2.7	14.0
40	36.4 ± 0.2	–	79.9 ± 0.9	–	218.7 ± 2.8	–	87.1 ± 2.0	18.1
60	39.8 ± 0.2	–	117.0 ± 8.1	–	561.9 ± 36.4	–	95.7 ± 2.3	75.4
80	39.7 ± 0.1	40.1	107.0 ± 6.2	102.9	605.2 ± 38.3	551.6	98.6 ± 1.1	93.6
100	39.7 ± 0.2	39.9	102.5 ± 3.0	97.8	637.8 ± 18.1	584.4	102.4 ± 5.8	97.9
120	39.9 ± 0.2	39.8	97.4 ± 1.7	94.0	656.5 ± 8.2	612.9	105.6 ± 4.7	101.8
140	39.7 ± 0.1	39.7	94.8 ± 4.5	91.1	683.2 ± 30.9	638.5	120.9 ± 9.5	105.4
160	39.7 ± 0.2	39.7	92.1 ± 0.9	88.8	709.5 ± 11.2	661.7	116.7 ± 5.6	108.9
180	39.7 ± 0.1	39.7	91.2 ± 1.2	86.9	728.8 ± 10.5	683.1	120.8 ± 7.1	112.1
200	39.7 ± 0.2	39.7	90.3 ± 1.8	85.3	743.2 ± 13.1	703.0	121.4 ± 7.0	115.3

Table S375: Densities computed from MC and MD simulations (ρ^{MC} and ρ^{MD}), densities obtained from REFPROP[10] (ρ^{REFP}), isothermal compressibilities computed from MC simulations (β_T^{MC}), isothermal compressibilities obtained from REFPROP[10] (β_T^{REFP}), thermal expansion coefficients computed from MC simulations (α_P^{MC}), thermal expansion coefficients obtained from REFPROP[10] (α_P^{REFP}), Joule Thomson coefficients computed from MC simulations ($\mu_{\mathrm{JT}}^{\mathrm{MC}}$), and Joule Thomson coefficients obtained from REFPROP[10] ($\mu_{\mathrm{JT}}^{\mathrm{REFP}}$) of CO_2 rich quaternary mixture with 2% impurity of H_2, 1% impurity of N_2 and 1% impurity of CH_4 at 293 K and pressures ranging from 20 bar to 200 bar.

P/ [bar]	ρ^{MC}/ [kg/m^3]	ρ^{MD}/ [kg/m^3]	ρ^{REFP}/ [kg/m^3]	β_T^{MC}/ [10^{-5}/bar]	β_T^{REFP}/ [10^{-5}/bar]	α_P^{MC}/ [10^{-4}/K]	α_P^{REFP}/ [10^{-4}/K]	$\mu_{\mathrm{JT}}^{\mathrm{MC}}$/ [$10^{-3}$ K/bar]	$\mu_{\mathrm{JT}}^{\mathrm{REFP}}$/ [$10^{-3}$ K/bar]
20	38.7	39.3 ± 0.1	39.3	5589.7 ± 27.7	5652.5	47.8 ± 0.3	49.4	1007.2 ± 19.7	1093.0
40	89.0 ± 0.1	90.7 ± 0.3	92.1	3348.2 ± 42.3	3487.6	75.9 ± 0.7	83.0	1018.2 ± 18.7	1087.8
60	169.8 ± 0.4	179.1 ± 1.1	186.1	3598.4 ± 143.7	4196.9	189.6 ± 8.4	249.6	1011.5 ± 65.3	1017.5
80	731.4 ± 3.8	726.5 ± 3.3	722.1	485.3 ± 184.1	491.7	198.4 ± 54.7	191.5	152.0 ± 55.9	158.5
100	777.7 ± 1.8	774.2 ± 2.1	772.2	222.2 ± 18.7	242.3	114.0 ± 9.0	114.5	94.9 ± 11.9	101.2
120	807.3 ± 2.5	804.7 ± 1.6	803.5	148.2 ± 7.9	165.5	86.3 ± 3.3	87.9	68.5 ± 4.6	74.1
140	827.1 ± 2.5	825.0 ± 1.8	827.1	112.8 ± 14.5	127.1	70.9 ± 8.0	73.5	51.7 ± 11.8	57.1
160	845.7 ± 2.7	843.4 ± 1.8	846.3	102.3 ± 5.8	103.7	68.0 ± 2.9	64.1	47.0 ± 4.1	45.2
180	861.7 ± 1.2	860.5 ± 0.3	862.5	85.2 ± 4.6	87.9	59.8 ± 3.1	57.5	37.1 ± 4.7	36.1
200	875.4 ± 1.2	873.5 ± 1.8	876.8	71.4 ± 3.2	76.5	52.8 ± 2.7	52.5	28.0 ± 4.2	28.9

Table S376: Heat capacities at constant volume computed from MC simulations (c_V^{MC}), heat capacities at constant volume obtained from REFPROP[10] (c_V^{REFP}), heat capacities at constant pressure computed from MC simulations (c_P^{MC}), heat capacities at constant pressure obtained from REFPROP[10] (c_P^{REFP}), speed of sound computed from MC simulations (c^{MC}), speed of sound obtained from REFPROP[10] (c^{REFP}), viscosities computed from MD simulations (η^{MD}), and viscosities obtained from REFPROP[10] (η^{REFP}) of CO_2 rich quaternary mixture with 2% impurity of H_2, 1% impurity of N_2 and 1% impurity of CH_4 at 293 K and pressures ranging from 20 bar to 200 bar.

P/ [bar]	c_V^{MC}/ [J/mol K]	c_V^{REFP}/ [J/mol K]	c_P^{MC}/ [J/mol K]	c_P^{REFP}/ [J/mol K]	c^{MC}/ [m/s]	c^{REFP}/ [m/s]	η^{MD}/ [μPa s]	η^{REFP}/ [μPa s]
20	30.6	30.7	43.8 ± 0.1	44.5	257.2 ± 0.7	255.3	8.6 ± 1.2	15.0
40	33.5 ± 0.1	34.2	57.8 ± 0.2	61.1	240.5 ± 1.6	235.7	15.8 ± 1.3	15.6
60	39.0 ± 0.2	42.6	113.3 ± 4.0	142.5	218.0 ± 5.8	207.0	17.4 ± 1.3	17.7
80	40.4 ± 0.2	42.9	185.0 ± 28.9	172.1	359.1 ± 73.7	336.4	66.9 ± 11.2	59.8
100	40.0 ± 0.2	41.0	135.5 ± 7.2	128.8	442.6 ± 22.1	409.7	73.2 ± 3.2	67.8
120	39.8 ± 0.2	40.3	118.2 ± 2.6	113.0	498.5 ± 14.4	459.3	76.9 ± 7.5	73.6
140	39.6 ± 0.3	39.9	107.5 ± 7.0	104.2	539.4 ± 39.0	498.5	78.8 ± 0.8	78.3
160	39.3 ± 0.2	39.7	106.8 ± 2.2	98.4	560.3 ± 17.1	531.6	94.2 ± 9.4	82.4
180	39.3 ± 0.1	39.5	100.7 ± 3.2	94.2	590.6 ± 18.5	560.6	85.0 ± 1.4	86.1
200	39.4 ± 0.3	39.4	95.4 ± 3.2	91.0	622.9 ± 17.8	586.6	99.0 ± 7.4	89.5

Table S377: Densities computed from MC and MD simulations (ρ^{MC} and ρ^{MD}), densities obtained from REFPROP[10] (ρ^{REFP}), isothermal compressibilities computed from MC simulations (β_T^{MC}), isothermal compressibilities obtained from REFPROP[10] (β_T^{REFP}), thermal expansion coefficients computed from MC simulations (α_P^{MC}), thermal expansion coefficients obtained from REFPROP[10] (α_P^{REFP}), Joule Thomson coefficients computed from MC simulations ($\mu_{\mathrm{JT}}^{\mathrm{MC}}$), and Joule Thomson coefficients obtained from REFPROP[10] ($\mu_{\mathrm{JT}}^{\mathrm{REFP}}$) of CO_2 rich quaternary mixture with 2% impurity of H_2, 1% impurity of N_2 and 1% impurity of CH_4 at 313 K and pressures ranging from 20 bar to 200 bar.

P/ [bar]	ρ^{MC}/ [kg/m^3]	ρ^{MD}/ [kg/m^3]	ρ^{REFP}/ [kg/m^3]	β_T^{MC}/ [10^{-5}/bar]	β_T^{REFP}/ [10^{-5}/bar]	α_P^{MC}/ [10^{-4}/K]	α_P^{REFP}/ [10^{-4}/K]	$\mu_{\mathrm{JT}}^{\mathrm{MC}}$/ [$10^{-3}$ K/bar]	$\mu_{\mathrm{JT}}^{\mathrm{REFP}}$/ [$10^{-3}$ K/bar]
20	35.4	35.9 ± 0.1	35.9	5420.7 ± 25.4	5484.8	41.4 ± 0.2	42.7	835.5 ± 16.6	921.7
40	78.0 ± 0.1	79.3 ± 0.1	80.0	3036.3 ± 18.9	3123.7	57.1 ± 0.5	60.8	843.2 ± 16.8	911.3
60	133.0 ± 0.2	136.3 ± 0.6	139.1	2453.4 ± 58.8	2551.5	88.9 ± 2.3	97.8	846.4 ± 35.9	878.7
80	214.9 ± 0.4	224.3 ± 0.8	233.6	2453.5 ± 40.4	2789.5	165.8 ± 4.1	208.2	766.2 ± 28.6	778.6
100	402.2 ± 3.7	412.6 ± 5.0	443.9	3615.3 ± 236.0	2967.9	573.2 ± 37.7	501.0	512.9 ± 48.4	470.2
120	601.1 ± 4.4	596.0 ± 7.1	609.8	883.5 ± 90.6	787.0	255.0 ± 22.4	222.1	239.0 ± 28.4	231.7
140	677.9 ± 2.8	672.8 ± 0.8	678.9	388.0 ± 18.1	377.4	145.8 ± 4.9	134.2	150.3 ± 7.4	149.2
160	717.2 ± 2.1	714.2 ± 2.7	721.1	247.1 ± 18.7	244.0	106.9 ± 6.7	100.2	110.5 ± 10.7	108.7
180	747.9 ± 3.2	745.9 ± 1.9	751.8	174.9 ± 11.7	179.9	85.2 ± 5.5	82.0	83.4 ± 9.4	83.8
200	771.8 ± 0.9	770.5 ± 1.0	776.3	142.7 ± 7.0	142.5	74.9 ± 3.4	70.5	69.0 ± 5.9	66.7

Table S378: Heat capacities at constant volume computed from MC simulations (c_V^{MC}), heat capacities at constant volume obtained from REFPROP[10] (c_V^{REFP}), heat capacities at constant pressure computed from MC simulations (c_P^{MC}), heat capacities at constant pressure obtained from REFPROP[10] (c_P^{REFP}), speed of sound computed from MC simulations (c^{MC}), speed of sound obtained from REFPROP[10] (c^{REFP}), viscosities computed from MD simulations (η^{MD}), and viscosities obtained from REFPROP[10] (η^{REFP}) of CO_2 rich quaternary mixture with 2% impurity of H_2, 1% impurity of N_2 and 1% impurity of CH_4 at 313 K and pressures ranging from 20 bar to 200 bar.

P/ [bar]	c_V^{MC}/ [J/mol K]	c_V^{REFP}/ [J/mol K]	c_P^{MC}/ [J/mol K]	c_P^{REFP}/ [J/mol K]	c^{MC}/ [m/s]	c^{REFP}/ [m/s]	η^{MD}/ [μPa s]	η^{REFP}/ [μPa s]
20	31.0	31.0	42.9 ± 0.1	43.3	268.7 ± 0.7	266.8	3.9 ± 3.6	15.9
40	32.8	33.1	51.2 ± 0.2	52.9	256.7 ± 1.0	252.8	16.2 ± 1.0	16.5
60	35.2 ± 0.1	36.0	67.7 ± 0.9	72.1	242.8 ± 3.4	237.5	17.6 ± 2.8	17.6
80	38.7 ± 0.4	40.6	108.7 ± 2.4	129.6	231.0 ± 3.4	221.2	20.5 ± 2.5	20.3
100	43.1 ± 0.3	45.7	351.1 ± 22.1	300.6	236.9 ± 10.8	223.4	33.7 ± 4.9	31.3
120	40.8 ± 0.4	42.6	207.7 ± 13.2	180.0	309.8 ± 18.8	296.9	53.6 ± 17.5	46.1
140	40.0 ± 0.2	41.0	149.4 ± 3.6	134.9	376.7 ± 10.0	358.6	55.3 ± 4.6	54.4
160	39.5 ± 0.2	40.2	126.4 ± 4.9	116.5	424.7 ± 18.0	405.9	57.4 ± 0.9	60.2
180	39.2 ± 0.2	39.8	114.2 ± 4.9	106.3	471.7 ± 18.9	444.5	70.1 ± 4.6	65.0
200	39.1 ± 0.1	39.5	107.9 ± 3.1	99.7	500.8 ± 14.2	477.6	70.9 ± 3.2	69.0

S17.3 Data of thermodynamics and transport properties of CO_2 rich quaternary mixture with 2 mole% impurity of N_2, 1 mole% impurity of H_2, and 1 mole% impurity of CH_4

Table S379: Densities computed from MC and MD simulations (ρ^{MC} and ρ^{MD}), densities obtained from REFPROP[10] (ρ^{REFP}), isothermal compressibilities computed from MC simulations (β_T^{MC}), isothermal compressibilities obtained from REFPROP[10] (β_T^{REFP}), thermal expansion coefficients computed from MC simulations (α_P^{MC}), thermal expansion coefficients obtained from REFPROP[10] (α_P^{REFP}), Joule Thomson coefficients computed from MC simulations (μ_{JT}^{MC}), and Joule Thomson coefficients obtained from REFPROP[10] (μ_{JT}^{REFP}) of CO_2 rich quaternary mixture with 2% impurity of N_2, 1% impurity of H_2 and 1% impurity of CH_4 at 253 K and pressures ranging from 20 bar to 200 bar.

P/ [bar]	ρ^{MC}/ [kg/m^3]	ρ^{MD}/ [kg/m^3]	ρ^{REFP}/ [kg/m^3]	β_T^{MC}/ [10^{-5}/bar]	β_T^{REFP}/ [10^{-5}/bar]	α_P^{MC}/ [10^{-4}/K]	α_P^{REFP}/ [10^{-4}/K]	μ_{JT}^{MC}/ [10^{-3} K/bar]	μ_{JT}^{REFP}/ [10^{-3} K/bar]
20	49.2	50.0 ± 0.1	50.7	6318.4 ± 47.5	6512.3	74.9 ± 0.7	81.6	1527.3 ± 30.2	1649.7
40	987.1 ± 0.9	985.5 ± 0.8	800.8	44.3 ± 1.8	-372.3	47.4 ± 1.7	-115.6	9.0 ± 1.9	–
60	996.4 ± 1.7	994.4 ± 0.5	994.1	43.3 ± 0.6	48.0	47.3 ± 0.8	47.5	8.7 ± 0.9	9.5
80	1004.6 ± 0.8	1002.7 ± 1.1	1003.3	39.3 ± 2.9	43.6	44.3 ± 3.1	44.5	5.5 ± 3.5	6.1
100	1011.5 ± 1.2	1010.4 ± 1.0	1011.7	35.9 ± 1.1	40.1	41.4 ± 1.6	42.1	2.2 ± 1.9	3.1
120	1019.0 ± 1.3	1017.2 ± 0.5	1019.5	32.9 ± 2.0	37.1	38.7 ± 2.1	40.0	-1.0 ± 2.6	0.6
140	1024.0 ± 1.4	1024.4 ± 0.5	1026.8	31.8 ± 1.6	34.6	38.4 ± 1.7	38.3	-1.4 ± 2.1	-1.6
160	1030.7 ± 1.2	1030.0 ± 0.6	1033.7	30.1 ± 1.1	32.5	37.2 ± 1.4	36.7	-2.7 ± 1.7	-3.5
180	1037.2 ± 1.6	1035.3 ± 0.8	1040.3	28.6 ± 0.9	30.6	36.1 ± 1.4	35.3	-4.1 ± 1.6	-5.3
200	1043.2 ± 1.3	1041.8 ± 0.9	1046.5	26.9 ± 1.0	29.0	34.6 ± 1.3	34.1	-5.9 ± 1.6	-6.9

Table S380: Heat capacities at constant volume computed from MC simulations (c_V^{MC}), heat capacities at constant volume obtained from REFPROP[10] (c_V^{REFP}), heat capacities at constant pressure computed from MC simulations (c_P^{MC}), heat capacities at constant pressure obtained from REFPROP[10] (c_P^{REFP}), speed of sound computed from MC simulations (c^{MC}), speed of sound obtained from REFPROP[10] (c^{REFP}), viscosities computed from MD simulations (η^{MD}), and viscosities obtained from REFPROP[10] (η^{REFP}) of CO_2 rich quaternary mixture with 2% impurity of N_2, 1% impurity of H_2 and 1% impurity of CH_4 at 253 K and pressures ranging from 20 bar to 200 bar.

P/ [bar]	c_V^{MC}/ [J/mol K]	c_V^{REFP}/ [J/mol K]	c_P^{MC}/ [J/mol K]	c_P^{REFP}/ [J/mol K]	c^{MC}/ [m/s]	c^{REFP}/ [m/s]	η^{MD}/ [μPa s]	η^{REFP}/ [μPa s]
20	31.5	32.8	51.1 ± 0.2	54.8	228.3 ± 1.0	224.9	13.6 ± 2.6	13.0
40	40.2 ± 0.3	–	96.2 ± 2.0	–	739.8 ± 16.7	–	138.9 ± 7.5	73.8
60	40.2 ± 0.1	40.3	96.7 ± 1.2	91.7	746.6 ± 7.0	690.4	137.8 ± 3.7	128.4
80	40.3 ± 0.1	40.3	94.6 ± 3.7	89.6	770.8 ± 32.2	712.7	148.1 ± 20.1	132.2
100	40.1 ± 0.1	40.3	91.7 ± 2.5	87.8	794.5 ± 16.1	733.4	141.2 ± 7.1	135.9
120	40.3 ± 0.2	40.3	88.9 ± 2.3	86.3	810.5 ± 26.9	752.6	154.0 ± 13.4	139.5
140	40.5 ± 0.3	40.3	89.8 ± 2.1	85.1	825.6 ± 22.5	770.6	153.3 ± 11.8	142.9
160	40.4 ± 0.2	40.3	88.9 ± 2.3	83.9	841.3 ± 18.7	787.6	148.5 ± 11.5	146.2
180	40.5 ± 0.2	40.3	88.2 ± 2.2	83.0	857.3 ± 17.9	803.7	158.9 ± 7.6	149.5
200	40.4 ± 0.2	40.4	87.0 ± 2.2	82.1	876.6 ± 19.7	819.1	167.2 ± 17.2	152.6

Table S381: Densities computed from MC and MD simulations (ρ^{MC} and ρ^{MD}), densities obtained from REFPROP[10] (ρ^{REFP}), isothermal compressibilities computed from MC simulations (β_T^{MC}), isothermal compressibilities obtained from REFPROP[10] (β_T^{REFP}), thermal expansion coefficients computed from MC simulations (α_P^{MC}), thermal expansion coefficients obtained from REFPROP[10] (α_P^{REFP}), Joule Thomson coefficients computed from MC simulations (μ_{JT}^{MC}), and Joule Thomson coefficients obtained from REFPROP[10] (μ_{JT}^{REFP}) of CO_2 rich quaternary mixture with 2% impurity of N_2, 1% impurity of H_2 and 1% impurity of CH_4 at 273 K and pressures ranging from 20 bar to 200 bar.

P/ [bar]	ρ^{MC}/ [kg/m^3]	ρ^{MD}/ [kg/m^3]	ρ^{REFP}/ [kg/m^3]	β_T^{MC}/ [10^{-5}/bar]	β_T^{REFP}/ [10^{-5}/bar]	α_P^{MC}/ [10^{-4}/K]	α_P^{REFP}/ [10^{-4}/K]	μ_{JT}^{MC}/ [10^{-3} K/bar]	μ_{JT}^{REFP}/ [10^{-3} K/bar]
20	43.2	43.9 ± 0.1	44.1	5792.1 ± 6.4	5938.9	56.8 ± 0.2	60.3	1196.9 ± 9.9	1330.3
40	108.5 ± 0.2	864.3 ± 0.7	243.6	4202.9 ± 77.7	-4028.8	128.9 ± 3.2	-554.0	1253.3 ± 49.6	–
60	887.4 ± 0.8	885.5 ± 0.6	882.0	96.7 ± 2.7	117.7	72.7 ± 2.4	78.7	42.3 ± 3.1	50.6
80	904.1 ± 1.2	901.9 ± 0.7	900.7	80.1 ± 2.0	93.5	64.4 ± 1.6	67.1	33.5 ± 2.0	38.6
100	916.8 ± 1.6	915.5 ± 1.1	916.2	68.8 ± 3.8	78.3	57.7 ± 3.1	59.5	26.5 ± 4.0	29.9
120	930.2 ± 1.5	927.3 ± 1.3	929.6	58.8 ± 2.4	67.7	51.8 ± 2.0	53.9	19.6 ± 2.7	23.2
140	940.3 ± 1.4	939.5 ± 0.9	941.5	52.8 ± 2.8	59.8	48.5 ± 2.5	49.7	15.5 ± 3.3	17.8
160	949.1 ± 1.5	948.0 ± 0.9	952.3	47.5 ± 1.6	53.8	44.9 ± 1.3	46.3	11.1 ± 1.7	13.4
180	959.1 ± 1.1	957.5 ± 0.9	962.1	46.0 ± 2.8	48.9	44.7 ± 2.7	43.5	10.6 ± 3.6	9.7
200	967.2 ± 1.3	965.9 ± 0.6	971.1	41.7 ± 1.0	44.9	41.3 ± 0.7	41.2	6.3 ± 0.9	6.4

Table S382: Heat capacities at constant volume computed from MC simulations (c_V^{MC}), heat capacities at constant volume obtained from REFPROP[10] (c_V^{REFP}), heat capacities at constant pressure computed from MC simulations (c_P^{MC}), heat capacities at constant pressure obtained from REFPROP[10] (c_P^{REFP}), speed of sound computed from MC simulations (c^{MC}), speed of sound obtained from REFPROP[10] (c^{REFP}), viscosities computed from MD simulations (η^{MD}), and viscosities obtained from REFPROP[10] (η^{REFP}) of CO_2 rich quaternary mixture with 2% impurity of N_2, 1% impurity of H_2 and 1% impurity of CH_4 at 273 K and pressures ranging from 20 bar to 200 bar.

P/ [bar]	c_V^{MC}/ [J/mol K]	c_V^{REFP}/ [J/mol K]	c_P^{MC}/ [J/mol K]	c_P^{REFP}/ [J/mol K]	c^{MC}/ [m/s]	c^{REFP}/ [m/s]	η^{MD}/ [μPa s]	η^{REFP}/ [μPa s]
20	30.7	31.0	45.8 ± 0.1	47.3	244.2 ± 0.3	241.2	10.7 ± 2.9	14.0
40	36.9 ± 0.1	–	79.6 ± 1.5	–	217.3 ± 2.9	–	91.1 ± 3.1	18.5
60	39.8 ± 0.1	40.6	113.0 ± 2.8	110.6	575.1 ± 10.8	512.1	99.3 ± 4.1	90.7
80	39.8	40.3	107.3 ± 1.8	103.0	610.2 ± 9.3	550.9	100.5 ± 3.0	95.6
100	39.8 ± 0.1	40.1	101.8 ± 3.5	97.9	636.7 ± 20.6	583.5	106.6 ± 10.9	99.9
120	39.6 ± 0.2	40.0	97.5 ± 2.4	94.2	671.1 ± 16.1	612.0	110.0 ± 6.2	103.9
140	39.6 ± 0.1	39.9	95.5 ± 2.8	91.3	696.4 ± 21.2	637.5	116.0 ± 14.2	107.6
160	39.6 ± 0.1	39.8	92.5 ± 1.5	89.0	720.2 ± 13.7	660.6	111.9 ± 4.6	111.1
180	39.6 ± 0.1	39.8	92.9 ± 3.2	87.1	729.6 ± 25.2	682.0	132.4 ± 17.8	114.4
200	39.8 ± 0.2	39.8	89.5 ± 0.8	85.5	746.4 ± 9.3	701.9	130.9 ± 22.2	117.6

Table S383: Densities computed from MC and MD simulations (ρ^{MC} and ρ^{MD}), densities obtained from REFPROP[10] (ρ^{REFP}), isothermal compressibilities computed from MC simulations (β_T^{MC}), isothermal compressibilities obtained from REFPROP[10] (β_T^{REFP}), thermal expansion coefficients computed from MC simulations (α_P^{MC}), thermal expansion coefficients obtained from REFPROP[10] (α_P^{REFP}), Joule Thomson coefficients computed from MC simulations (μ_{JT}^{MC}), and Joule Thomson coefficients obtained from REFPROP[10] (μ_{JT}^{REFP}) of CO_2 rich quaternary mixture with 2% impurity of N_2, 1% impurity of H_2 and 1% impurity of CH_4 at 293 K and pressures ranging from 20 bar to 200 bar.

P/ [bar]	ρ^{MC}/ [kg/m^3]	ρ^{MD}/ [kg/m^3]	ρ^{REFP}/ [kg/m^3]	β_T^{MC}/ [10^{-5}/bar]	β_T^{REFP}/ [10^{-5}/bar]	α_P^{MC}/ [10^{-4}/K]	α_P^{REFP}/ [10^{-4}/K]	μ_{JT}^{MC}/ [10^{-3} K/bar]	μ_{JT}^{REFP}/ [10^{-3} K/bar]
20	39.0	39.5 ± 0.1	39.6	5569.4 ± 38.5	5658.1	47.7 ± 0.3	49.6	999.1 ± 24.0	1101.6
40	89.7 ± 0.1	91.5 ± 0.1	92.9	3386.9 ± 38.0	3501.9	77.0 ± 1.0	83.9	1034.8 ± 25.4	1094.5
60	172.6 ± 0.9	182.9 ± 1.1	189.4	3622.6 ± 263.3	4348.1	195.4 ± 16.5	264.4	1006.0 ± 121.6	1019.5
80	741.6 ± 6.2	737.8 ± 2.1	730.7	513.4 ± 134.9	478.7	213.0 ± 41.9	188.1	154.4 ± 40.7	155.7
100	788.4 ± 2.7	783.8 ± 1.8	780.4	198.8 ± 14.1	238.9	106.6 ± 5.3	113.7	87.7 ± 6.8	99.9
120	814.3 ± 1.8	812.6 ± 1.7	811.7	135.5 ± 8.2	163.8	80.7 ± 4.4	87.5	63.0 ± 6.4	73.3
140	834.3 ± 1.3	833.2 ± 0.9	835.2	114.5 ± 2.0	126.0	72.6 ± 1.9	73.3	53.1 ± 2.8	56.6
160	850.5 ± 2.1	851.5 ± 0.8	854.4	93.5 ± 6.2	102.9	63.0 ± 3.3	64.0	41.6 ± 4.8	44.7
180	867.5 ± 2.5	865.8 ± 0.7	870.8	81.1 ± 4.5	87.3	58.0 ± 3.2	57.5	34.7 ± 4.7	35.8
200	880.8 ± 1.1	879.3 ± 0.7	885.0	71.7 ± 2.8	76.0	53.4 ± 2.2	52.5	28.6 ± 3.4	28.7

Table S384: Heat capacities at constant volume computed from MC simulations (c_V^{MC}), heat capacities at constant volume obtained from REFPROP[10] (c_V^{REFP}), heat capacities at constant pressure computed from MC simulations (c_P^{MC}), heat capacities at constant pressure obtained from REFPROP[10] (c_P^{REFP}), speed of sound computed from MC simulations (c^{MC}), speed of sound obtained from REFPROP[10] (c^{REFP}), viscosities computed from MD simulations (η^{MD}), and viscosities obtained from REFPROP[10] (η^{REFP}) of CO_2 rich quaternary mixture with 2% impurity of N_2, 1% impurity of H_2 and 1% impurity of CH_4 at 293 K and pressures ranging from 20 bar to 200 bar.

P/ [bar]	c_V^{MC}/ [J/mol K]	c_V^{REFP}/ [J/mol K]	c_P^{MC}/ [J/mol K]	c_P^{REFP}/ [J/mol K]	c^{MC}/ [m/s]	c^{REFP}/ [m/s]	η^{MD}/ [μPa s]	η^{REFP}/ [μPa s]
20	30.7	30.8	43.8 ± 0.1	44.6	256.7 ± 0.9	254.4	9.4 ± 4.8	15.0
40	33.6 ± 0.1	34.4	58.2 ± 0.4	61.7	238.7 ± 1.6	234.7	15.5 ± 1.3	15.7
60	39.3 ± 0.4	43.3	116.9 ± 7.5	150.2	218.2 ± 10.7	205.3	18.0 ± 1.0	17.8
80	40.5 ± 0.4	43.1	196.9 ± 23.9	170.5	357.4 ± 51.8	336.4	64.6 ± 1.8	61.0
100	39.9 ± 0.1	41.2	132.1 ± 3.3	128.6	459.8 ± 17.3	409.0	72.2 ± 4.0	69.2
120	39.3 ± 0.2	40.5	114.4 ± 4.1	113.1	513.4 ± 18.0	458.4	76.2 ± 3.6	75.0
140	39.4 ± 0.1	40.1	109.4 ± 2.4	104.3	539.0 ± 7.5	497.4	86.0 ± 6.8	79.7
160	39.2 ± 0.2	39.8	102.6 ± 2.4	98.6	573.4 ± 20.3	530.4	86.8 ± 2.8	83.9
180	39.2 ± 0.1	39.7	99.8 ± 3.3	94.4	601.5 ± 19.5	559.4	90.6 ± 4.4	87.7
200	39.2 ± 0.1	39.6	96.3 ± 2.5	91.2	623.8 ± 14.6	585.3	96.4 ± 10.5	91.1

Table S385: Densities computed from MC and MD simulations (ρ^{MC} and ρ^{MD}), densities obtained from REFPROP[10] (ρ^{REFP}), isothermal compressibilities computed from MC simulations (β_T^{MC}), isothermal compressibilities obtained from REFPROP[10] (β_T^{REFP}), thermal expansion coefficients computed from MC simulations (α_P^{MC}), thermal expansion coefficients obtained from REFPROP[10] (α_P^{REFP}), Joule Thomson coefficients computed from MC simulations ($\mu_{\mathrm{JT}}^{\mathrm{MC}}$), and Joule Thomson coefficients obtained from REFPROP[10] ($\mu_{\mathrm{JT}}^{\mathrm{REFP}}$) of CO_2 rich quaternary mixture with 2% impurity of N_2, 1% impurity of H_2 and 1% impurity of CH_4 at 313 K and pressures ranging from 20 bar to 200 bar.

P/ [bar]	ρ^{MC}/ [kg/m^3]	ρ^{MD}/ [kg/m^3]	ρ^{REFP}/ [kg/m^3]	β_T^{MC}/ [10^{-5}/bar]	β_T^{REFP}/ [10^{-5}/bar]	α_P^{MC}/ [10^{-4}/K]	α_P^{REFP}/ [10^{-4}/K]	$\mu_{\mathrm{JT}}^{\mathrm{MC}}$/ [$10^{-3}$ K/bar]	$\mu_{\mathrm{JT}}^{\mathrm{REFP}}$/ [$10^{-3}$ K/bar]
20	35.7	36.2 ± 0.1	36.1	5434.8 ± 15.4	5488.2	41.6 ± 0.1	42.8	850.6 ± 13.2	928.5
40	78.7 ± 0.1	80.0 ± 0.2	80.6	3053.7 ± 21.9	3129.7	57.6 ± 0.4	61.2	854.7 ± 14.6	916.8
60	134.4 ± 0.1	137.4 ± 0.6	140.4	2446.9 ± 41.7	2565.7	89.7 ± 1.7	99.1	847.2 ± 25.8	882.8
80	218.7 ± 1.3	227.7 ± 2.3	237.1	2570.7 ± 81.0	2841.0	178.5 ± 5.1	215.4	778.3 ± 30.2	778.5
100	416.0 ± 4.3	429.6 ± 3.7	454.5	3727.0 ± 274.3	2888.5	611.4 ± 46.0	499.5	498.9 ± 52.5	458.3
120	610.2 ± 7.0	611.6 ± 1.4	617.5	855.8 ± 94.1	768.2	250.4 ± 19.0	218.8	234.4 ± 23.1	227.9
140	680.2 ± 1.8	682.1 ± 1.7	686.2	374.2 ± 32.4	372.6	141.0 ± 10.8	133.3	147.5 ± 16.4	147.5
160	724.5 ± 3.2	722.0 ± 1.3	728.3	236.1 ± 17.2	241.9	103.5 ± 6.9	99.9	106.5 ± 11.2	107.7
180	756.2 ± 0.8	753.1 ± 1.9	759.1	173.6 ± 5.4	178.7	85.2 ± 2.4	81.9	82.9 ± 4.1	83.2
200	778.8 ± 2.1	775.6 ± 1.5	783.6	141.2 ± 4.4	141.7	74.9 ± 1.9	70.5	68.6 ± 3.2	66.2

Table S386: Heat capacities at constant volume computed from MC simulations (c_V^{MC}), heat capacities at constant volume obtained from REFPROP[10] (c_V^{REFP}), heat capacities at constant pressure computed from MC simulations (c_P^{MC}), heat capacities at constant pressure obtained from REFPROP[10] (c_P^{REFP}), speed of sound computed from MC simulations (c^{MC}), speed of sound obtained from REFPROP[10] (c^{REFP}), viscosities computed from MD simulations (η^{MD}), and viscosities obtained from REFPROP[10] (η^{REFP}) of CO_2 rich quaternary mixture with 2% impurity of N_2, 1% impurity of H_2 and 1% impurity of CH_4 at 313 K and pressures ranging from 20 bar to 200 bar.

P/ [bar]	c_V^{MC}/ [J/mol K]	c_V^{REFP}/ [J/mol K]	c_P^{MC}/ [J/mol K]	c_P^{REFP}/ [J/mol K]	c^{MC}/ [m/s]	c^{REFP}/ [m/s]	η^{MD}/ [μPa s]	η^{REFP}/ [μPa s]
20	31.0	31.0	43.0 ± 0.1	43.4	267.7 ± 0.4	265.9	5.6 ± 3.8	16.0
40	32.8	33.2	51.4 ± 0.2	53.2	255.4 ± 1.0	251.8	17.0 ± 2.8	16.5
60	35.2	36.2	68.2 ± 0.7	72.9	242.6 ± 2.4	236.4	18.4 ± 2.5	17.7
80	38.8 ± 0.2	41.0	115.8 ± 1.9	133.7	230.6 ± 4.2	219.9	21.2 ± 1.3	20.5
100	43.5 ± 0.4	46.3	375.8 ± 25.6	302.0	236.1 ± 12.0	223.0	32.4 ± 4.1	32.1
120	41.0 ± 0.4	42.9	205.6 ± 9.2	178.7	310.0 ± 18.6	296.5	47.9 ± 4.9	47.0
140	39.9 ± 0.2	41.2	146.3 ± 7.4	134.8	379.7 ± 19.1	357.8	53.2 ± 2.0	55.3
160	39.5 ± 0.2	40.4	124.7 ± 5.4	116.6	429.7 ± 18.3	404.8	64.6 ± 6.1	61.3
180	39.3 ± 0.2	40.0	114.2 ± 2.3	106.5	470.7 ± 8.8	443.3	64.4 ± 3.8	66.1
200	39.1 ± 0.2	39.7	108.2 ± 1.7	99.9	501.9 ± 8.9	476.3	70.4 ± 2.4	70.2

S17.4 Data of thermodynamics and transport properties of CO_2 rich quaternary mixture with 2 mole% impurity of Ar, 1 mole% impurity of H_2, and 1 mole% impurity of CH_4

Table S387: Densities computed from MC and MD simulations (ρ^{MC} and ρ^{MD}), densities obtained from REFPROP[10] (ρ^{REFP}), isothermal compressibilities computed from MC simulations (β_T^{MC}), isothermal compressibilities obtained from REFPROP[10] (β_T^{REFP}), thermal expansion coefficients computed from MC simulations (α_P^{MC}), thermal expansion coefficients obtained from REFPROP[10] (α_P^{REFP}), Joule Thomson coefficients computed from MC simulations (μ_{JT}^{MC}), and Joule Thomson coefficients obtained from REFPROP[10] (μ_{JT}^{REFP}) of CO_2 rich quaternary mixture with 2% impurity of Ar, 1% impurity of H_2 and 1% impurity of CH_4 at 253 K and pressures ranging from 20 bar to 200 bar.

P/ [bar]	ρ^{MC}/ [kg/m^3]	ρ^{MD}/ [kg/m^3]	ρ^{REFP}/ [kg/m^3]	β_T^{MC}/ [10^{-5}/bar]	β_T^{REFP}/ [10^{-5}/bar]	α_P^{MC}/ [10^{-4}/K]	α_P^{REFP}/ [10^{-4}/K]	μ_{JT}^{MC}/ [10^{-3} K/bar]	μ_{JT}^{REFP}/ [10^{-3} K/bar]
20	49.5	50.3 ± 0.2	50.9	6229.6 ± 77.8	6494.8	73.2 ± 0.8	80.9	1479.7 ± 36.0	1642.5
40	999.6 ± 1.7	997.3 ± 0.4	840.9	44.0 ± 2.3	−1174.2	47.8 ± 1.9	−520.1	9.3 ± 2.2	–
60	1007.8 ± 0.9	1006.2 ± 0.9	1004.6	40.5 ± 0.9	46.3	45.0 ± 1.1	46.7	6.3 ± 1.3	8.6
80	1015.2 ± 1.3	1014.4 ± 0.9	1013.5	37.7 ± 1.5	42.2	43.1 ± 1.8	43.9	4.1 ± 2.0	5.3
100	1023.5 ± 0.6	1021.6 ± 0.7	1021.8	34.8 ± 1.6	38.9	41.1 ± 1.9	41.6	1.9 ± 2.2	2.5
120	1029.7 ± 1.6	1028.3 ± 0.9	1029.5	33.1 ± 0.6	36.1	39.4 ± 0.7	39.6	-0.1 ± 0.8	0.1
140	1036.6 ± 1.4	1035.4 ± 0.2	1036.7	32.0 ± 0.9	33.8	39.4 ± 1.4	37.9	-0.2 ± 1.7	−2.0
160	1042.4 ± 1.1	1041.5 ± 0.8	1043.5	29.3 ± 1.3	31.7	36.8 ± 1.6	36.4	-3.2 ± 1.9	−3.9
180	1048.7 ± 1.0	1047.3 ± 0.5	1049.9	27.0 ± 0.6	30.0	34.2 ± 1.1	35.1	-6.5 ± 1.4	−5.6
200	1054.8 ± 0.4	1052.7 ± 0.5	1056.1	25.7 ± 0.5	28.4	33.3 ± 0.9	33.9	-7.6 ± 1.1	−7.1

Table S388: Heat capacities at constant volume computed from MC simulations (c_V^{MC}), heat capacities at constant volume obtained from REFPROP[10] (c_V^{REFP}), heat capacities at constant pressure computed from MC simulations (c_P^{MC}), heat capacities at constant pressure obtained from REFPROP[10] (c_P^{REFP}), speed of sound computed from MC simulations (c^{MC}), speed of sound obtained from REFPROP[10] (c^{REFP}), viscosities computed from MD simulations (η^{MD}), and viscosities obtained from REFPROP[10] (η^{REFP}) of CO_2 rich quaternary mixture with 2% impurity of Ar, 1% impurity of H_2 and 1% impurity of CH_4 at 253 K and pressures ranging from 20 bar to 200 bar.

P/ [bar]	c_V^{MC}/ [J/mol K]	c_V^{REFP}/ [J/mol K]	c_P^{MC}/ [J/mol K]	c_P^{REFP}/ [J/mol K]	c^{MC}/ [m/s]	c^{REFP}/ [m/s]	η^{MD}/ [μPa s]	η^{REFP}/ [μPa s]
20	31.4 ± 0.1	32.5	50.3 ± 0.2	54.2	228.2 ± 1.5	224.6	12.7 ± 4.2	13.1
40	40.0 ± 0.2	–	97.0 ± 1.8	–	742.2 ± 20.6	–	128.6 ± 13.3	80.6
60	40.0 ± 0.2	40.0	94.5 ± 1.6	91.3	760.8 ± 11.2	700.5	142.9 ± 8.7	130.0
80	40.0 ± 0.2	40.0	93.3 ± 2.3	89.3	781.2 ± 18.7	722.2	140.3 ± 2.3	133.8
100	40.1 ± 0.3	40.0	91.9 ± 2.3	87.6	802.4 ± 21.0	742.2	145.4 ± 4.4	137.5
120	40.0 ± 0.2	40.0	90.1 ± 1.1	86.2	814.0 ± 8.6	760.9	146.2 ± 4.7	141.0
140	40.2 ± 0.2	40.0	91.1 ± 2.2	84.9	826.6 ± 15.7	778.5	147.7 ± 10.1	144.4
160	40.2 ± 0.3	40.1	88.5 ± 2.3	83.8	848.9 ± 21.7	795.1	159.3 ± 10.7	147.7
180	40.0 ± 0.1	40.1	85.3 ± 2.1	82.9	867.0 ± 14.6	810.8	159.7 ± 6.0	151.0
200	40.4 ± 0.3	40.1	85.0 ± 1.7	82.0	880.8 ± 12.8	825.8	163.8 ± 7.0	154.2

Table S389: Densities computed from MC and MD simulations (ρ^{MC} and ρ^{MD}), densities obtained from REFPROP[10] (ρ^{REFP}), isothermal compressibilities computed from MC simulations (β_T^{MC}), isothermal compressibilities obtained from REFPROP[10] (β_T^{REFP}), thermal expansion coefficients computed from MC simulations (α_P^{MC}), thermal expansion coefficients obtained from REFPROP[10] (α_P^{REFP}), Joule Thomson coefficients computed from MC simulations ($\mu_{\mathrm{JT}}^{\mathrm{MC}}$), and Joule Thomson coefficients obtained from REFPROP[10] ($\mu_{\mathrm{JT}}^{\mathrm{REFP}}$) of CO_2 rich quaternary mixture with 2% impurity of Ar, 1% impurity of H_2 and 1% impurity of CH_4 at 273 K and pressures ranging from 20 bar to 200 bar.

P/ [bar]	ρ^{MC}/ [kg/m^3]	ρ^{MD}/ [kg/m^3]	ρ^{REFP}/ [kg/m^3]	β_T^{MC}/ [10^{-5}/bar]	β_T^{REFP}/ [10^{-5}/bar]	α_P^{MC}/ [10^{-4}/K]	α_P^{REFP}/ [10^{-4}/K]	$\mu_{\mathrm{JT}}^{\mathrm{MC}}$/ [$10^{-3}$ K/bar]	$\mu_{\mathrm{JT}}^{\mathrm{REFP}}$/ [$10^{-3}$ K/bar]
20	43.5	44.2 ± 0.1	44.3	5866.9 ± 53.8	5931.7	57.5 ± 0.4	60.0	1235.0 ± 25.1	1325.0
40	109.1 ± 0.2	881.7 ± 2.1	253.8	4341.1 ± 57.9	-3292.6	132.6 ± 2.8	-497.7	1290.1 ± 44.7	–
60	902.2 ± 2.8	898.6 ± 1.0	894.0	90.0 ± 3.6	110.3	69.6 ± 2.4	75.8	39.0 ± 3.0	47.5
80	917.1 ± 1.8	914.1 ± 0.8	911.8	75.5 ± 4.1	88.9	61.8 ± 3.1	65.3	30.9 ± 4.0	36.4
100	928.2 ± 0.7	928.3 ± 0.6	926.8	67.1 ± 2.6	75.1	57.0 ± 2.0	58.2	25.7 ± 2.6	28.3
120	941.6 ± 1.4	940.2 ± 1.0	939.8	57.5 ± 3.6	65.3	51.2 ± 3.2	53.0	18.8 ± 4.2	22.0
140	952.1 ± 1.3	949.8 ± 1.3	951.5	53.9 ± 2.1	58.0	49.8 ± 1.4	49.0	16.9 ± 1.9	16.9
160	961.6 ± 0.7	959.4 ± 0.5	962.0	47.2 ± 1.4	52.2	45.1 ± 1.5	45.8	11.2 ± 2.0	12.6
180	970.5 ± 1.9	969.4 ± 0.9	971.6	42.7 ± 1.5	47.6	42.0 ± 1.4	43.1	7.3 ± 1.9	9.0
200	978.6 ± 2.0	977.8 ± 0.6	980.5	40.7 ± 0.7	43.8	40.8 ± 1.1	40.8	5.7 ± 1.5	5.9

Table S390: Heat capacities at constant volume computed from MC simulations (c_V^{MC}), heat capacities at constant volume obtained from REFPROP[10] (c_V^{REFP}), heat capacities at constant pressure computed from MC simulations (c_P^{MC}), heat capacities at constant pressure obtained from REFPROP[10] (c_P^{REFP}), speed of sound computed from MC simulations (c^{MC}), speed of sound obtained from REFPROP[10] (c^{REFP}), viscosities computed from MD simulations (η^{MD}), and viscosities obtained from REFPROP[10] (η^{REFP}) of CO_2 rich quaternary mixture with 2% impurity of Ar, 1% impurity of H_2 and 1% impurity of CH_4 at 273 K and pressures ranging from 20 bar to 200 bar.

P/ [bar]	c_V^{MC}/ [J/mol K]	c_V^{REFP}/ [J/mol K]	c_P^{MC}/ [J/mol K]	c_P^{REFP}/ [J/mol K]	c^{MC}/ [m/s]	c^{REFP}/ [m/s]	η^{MD}/ [μPa s]	η^{REFP}/ [μPa s]
20	30.5	30.8	45.8 ± 0.1	46.9	242.6 ± 1.1	240.9	10.8 ± 6.4	14.1
40	36.6 ± 0.1	–	80.5 ± 1.4	–	215.6 ± 2.4	–	95.1 ± 4.9	18.9
60	39.5 ± 0.1	40.2	110.4 ± 2.6	109.0	586.3 ± 13.6	524.8	97.4 ± 1.9	92.3
80	39.5 ± 0.2	39.9	104.7 ± 3.3	102.0	618.7 ± 19.5	561.9	106.1 ± 3.6	97.0
100	39.4 ± 0.1	39.7	101.1 ± 2.4	97.2	641.3 ± 14.6	593.3	111.0 ± 10.5	101.3
120	39.3 ± 0.2	39.6	96.9 ± 3.7	93.7	674.5 ± 24.9	620.9	125.8 ± 21.2	105.2
140	39.3 ± 0.1	39.6	96.7 ± 1.1	90.9	692.1 ± 14.3	645.6	111.4 ± 3.3	108.9
160	39.4 ± 0.1	39.5	92.4 ± 1.9	88.7	718.2 ± 13.0	668.2	129.1 ± 24.5	112.3
180	39.5 ± 0.1	39.5	89.7 ± 1.7	86.9	739.6 ± 14.8	689.1	121.1 ± 3.0	115.6
200	39.5 ± 0.2	39.5	89.0 ± 1.8	85.3	752.0 ± 10.3	708.5	136.3 ± 18.7	118.8

Table S391: Densities computed from MC and MD simulations (ρ^{MC} and ρ^{MD}), densities obtained from REFPROP[10] (ρ^{REFP}), isothermal compressibilities computed from MC simulations (β_T^{MC}), isothermal compressibilities obtained from REFPROP[10] (β_T^{REFP}), thermal expansion coefficients computed from MC simulations (α_P^{MC}), thermal expansion coefficients obtained from REFPROP[10] (α_P^{REFP}), Joule Thomson coefficients computed from MC simulations ($\mu_{\mathrm{JT}}^{\mathrm{MC}}$), and Joule Thomson coefficients obtained from REFPROP[10] ($\mu_{\mathrm{JT}}^{\mathrm{REFP}}$) of CO_2 rich quaternary mixture with 2% impurity of Ar, 1% impurity of H_2 and 1% impurity of CH_4 at 293 K and pressures ranging from 20 bar to 200 bar.

P/ [bar]	ρ^{MC}/ [kg/m^3]	ρ^{MD}/ [kg/m^3]	ρ^{REFP}/ [kg/m^3]	β_T^{MC}/ [10^{-5}/bar]	β_T^{REFP}/ [10^{-5}/bar]	α_P^{MC}/ [10^{-4}/K]	α_P^{REFP}/ [10^{-4}/K]	$\mu_{\mathrm{JT}}^{\mathrm{MC}}$/ [$10^{-3}$ K/bar]	$\mu_{\mathrm{JT}}^{\mathrm{REFP}}$/ [$10^{-3}$ K/bar]
20	39.2	39.8 ± 0.1	39.8	5568.5 ± 30.0	5654.5	47.6 ± 0.2	49.4	998.5 ± 15.6	1097.7
40	90.3 ± 0.1	92.1 ± 0.3	93.3	3372.2 ± 28.8	3495.5	76.7 ± 0.9	83.3	1032.5 ± 24.0	1094.1
60	173.9 ± 0.4	206.3 ± 46.2	189.6	3589.0 ± 115.8	4308.2	192.0 ± 7.3	259.3	1003.0 ± 55.6	1022.9
80	757.4 ± 6.9	755.1 ± 5.0	747.8	384.7 ± 66.0	413.7	172.9 ± 22.4	171.2	133.9 ± 24.0	143.2
100	801.4 ± 2.4	797.7 ± 2.2	793.3	186.3 ± 12.0	221.2	102.9 ± 5.9	108.9	83.5 ± 7.8	94.6
120	824.6 ± 2.5	823.1 ± 1.7	823.1	139.6 ± 13.0	155.1	83.8 ± 5.0	85.1	65.1 ± 6.8	70.1
140	846.5 ± 0.7	845.3 ± 1.2	845.8	109.9 ± 5.9	120.6	71.2 ± 4.0	71.7	51.1 ± 5.8	54.4
160	862.2 ± 0.8	862.8 ± 1.9	864.5	93.2 ± 6.4	99.3	63.7 ± 3.8	63.0	42.0 ± 5.5	43.1
180	879.4 ± 1.4	877.6 ± 1.2	880.5	76.7 ± 2.4	84.6	55.9 ± 1.4	56.7	32.0 ± 2.2	34.5
200	892.6 ± 1.8	890.0 ± 0.7	894.5	66.6 ± 2.3	73.9	51.1 ± 1.6	51.9	25.3 ± 2.4	27.7

Table S392: Heat capacities at constant volume computed from MC simulations (c_V^{MC}), heat capacities at constant volume obtained from REFPROP[10] (c_V^{REFP}), heat capacities at constant pressure computed from MC simulations (c_P^{MC}), heat capacities at constant pressure obtained from REFPROP[10] (c_P^{REFP}), speed of sound computed from MC simulations (c^{MC}), speed of sound obtained from REFPROP[10] (c^{REFP}), viscosities computed from MD simulations (η^{MD}), and viscosities obtained from REFPROP[10] (η^{REFP}) of CO_2 rich quaternary mixture with 2% impurity of Ar, 1% impurity of H_2 and 1% impurity of CH_4 at 293 K and pressures ranging from 20 bar to 200 bar.

P/ [bar]	c_V^{MC}/ [J/mol K]	c_V^{REFP}/ [J/mol K]	c_P^{MC}/ [J/mol K]	c_P^{REFP}/ [J/mol K]	c^{MC}/ [m/s]	c^{REFP}/ [m/s]	η^{MD}/ [μPa s]	η^{REFP}/ [μPa s]
20	30.5	30.6	43.6 ± 0.1	44.3	256.1 ± 0.7	254.0	7.0 ± 5.5	15.1
40	33.4 ± 0.1	34.1	57.8 ± 0.4	61.1	238.3 ± 1.3	234.4	16.4 ± 2.2	15.8
60	39.5 ± 0.5	42.8	114.7 ± 3.5	147.1	215.6 ± 5.0	205.1	17.5 ± 3.1	17.9
80	40.0 ± 0.3	42.2	173.3 ± 13.5	162.1	385.8 ± 36.4	352.4	65.5 ± 1.9	63.0
100	39.5 ± 0.1	40.7	130.1 ± 5.0	126.2	470.0 ± 17.7	420.5	78.3 ± 9.2	70.6
120	39.4 ± 0.2	40.0	117.2 ± 2.9	111.8	508.3 ± 24.5	468.0	75.9 ± 1.6	76.2
140	39.3 ± 0.2	39.7	108.7 ± 4.0	103.6	545.1 ± 17.8	505.8	85.4 ± 6.5	80.9
160	39.1 ± 0.2	39.5	103.4 ± 3.4	98.0	573.6 ± 21.8	538.0	88.3 ± 6.3	85.0
180	39.2 ± 0.1	39.3	97.8 ± 1.3	94.0	608.2 ± 10.4	566.3	90.9 ± 2.3	88.7
200	39.0 ± 0.2	39.3	94.9 ± 1.6	90.9	639.7 ± 12.3	591.8	115.5 ± 42.2	92.2

Table S393: Densities computed from MC and MD simulations (ρ^{MC} and ρ^{MD}), densities obtained from REFPROP[10] (ρ^{REFP}), isothermal compressibilities computed from MC simulations (β_T^{MC}), isothermal compressibilities obtained from REFPROP[10] (β_T^{REFP}), thermal expansion coefficients computed from MC simulations (α_P^{MC}), thermal expansion coefficients obtained from REFPROP[10] (α_P^{REFP}), Joule Thomson coefficients computed from MC simulations ($\mu_{\mathrm{JT}}^{\mathrm{MC}}$), and Joule Thomson coefficients obtained from REFPROP[10] ($\mu_{\mathrm{JT}}^{\mathrm{REFP}}$) of CO_2 rich quaternary mixture with 2% impurity of Ar, 1% impurity of H_2 and 1% impurity of CH_4 at 313 K and pressures ranging from 20 bar to 200 bar.

P/ [bar]	ρ^{MC}/ [kg/m^3]	ρ^{MD}/ [kg/m^3]	ρ^{REFP}/ [kg/m^3]	β_T^{MC}/ [10^{-5}/bar]	β_T^{REFP}/ [10^{-5}/bar]	α_P^{MC}/ [10^{-4}/K]	α_P^{REFP}/ [10^{-4}/K]	$\mu_{\mathrm{JT}}^{\mathrm{MC}}$/ [$10^{-3}$ K/bar]	$\mu_{\mathrm{JT}}^{\mathrm{REFP}}$/ [$10^{-3}$ K/bar]
20	35.9	36.3 ± 0.1	36.3	5417.5 ± 18.8	5486.3	41.5 ± 0.1	42.7	842.7 ± 10.5	925.8
40	79.1	80.4 ± 0.2	81.0	3047.0 ± 22.5	3127.7	57.4 ± 0.3	60.9	852.1 ± 11.3	916.6
60	135.4 ± 0.2	138.4 ± 0.8	141.0	2456.3 ± 34.1	2562.8	90.0 ± 1.7	98.5	851.7 ± 27.7	884.5
80	221.2 ± 0.7	231.4 ± 2.3	238.4	2603.2 ± 71.3	2850.3	180.7 ± 5.8	215.2	784.9 ± 38.8	–
100	432.0 ± 2.9	459.3 ± 7.4	466.8	3434.8 ± 236.2	3034.6	585.0 ± 46.8	539.5	478.7 ± 55.5	454.3
120	627.0 ± 7.2	625.3 ± 7.1	631.7	729.6 ± 39.3	726.0	224.1 ± 6.1	214.2	216.2 ± 8.7	219.8
140	698.5 ± 1.8	692.5 ± 1.1	698.0	338.5 ± 34.7	354.7	134.4 ± 11.4	130.5	137.8 ± 16.9	142.8
160	735.0 ± 2.6	731.0 ± 2.2	739.0	226.4 ± 13.6	232.4	102.3 ± 5.2	98.2	103.7 ± 8.3	104.7
180	764.3 ± 2.1	763.9 ± 1.7	769.1	164.9 ± 3.8	172.8	82.1 ± 2.1	80.8	79.2 ± 3.7	81.1
200	789.2 ± 1.3	786.5 ± 1.7	793.1	128.8 ± 6.7	137.6	69.6 ± 3.0	69.7	62.3 ± 5.1	64.7

Table S394: Heat capacities at constant volume computed from MC simulations (c_V^{MC}), heat capacities at constant volume obtained from REFPROP[10] (c_V^{REFP}), heat capacities at constant pressure computed from MC simulations (c_P^{MC}), heat capacities at constant pressure obtained from REFPROP[10] (c_P^{REFP}), speed of sound computed from MC simulations (c^{MC}), speed of sound obtained from REFPROP[10] (c^{REFP}), viscosities computed from MD simulations (η^{MD}), and viscosities obtained from REFPROP[10] (η^{REFP}) of CO_2 rich quaternary mixture with 2% impurity of Ar, 1% impurity of H_2 and 1% impurity of CH_4 at 313 K and pressures ranging from 20 bar to 200 bar.

P/ [bar]	c_V^{MC}/ [J/mol K]	c_V^{REFP}/ [J/mol K]	c_P^{MC}/ [J/mol K]	c_P^{REFP}/ [J/mol K]	c^{MC}/ [m/s]	c^{REFP}/ [m/s]	η^{MD}/ [μPa s]	η^{REFP}/ [μPa s]
20	30.8	30.8	42.8	43.2	267.4 ± 0.5	265.4	2.3 ± 2.7	16.0
40	32.6	32.9	51.1 ± 0.1	52.7	255.0 ± 1.0	251.4	17.8 ± 1.7	16.6
60	35.0 ± 0.1	35.9	68.2 ± 0.9	72.2	241.9 ± 2.4	236.0	21.7 ± 4.3	17.8
80	38.6 ± 0.3	–	115.9 ± 3.5	–	228.3 ± 4.7	–	21.5 ± 2.1	20.6
100	42.7 ± 0.4	45.8	362.1 ± 28.6	323.9	239.0 ± 12.6	223.4	31.6 ± 1.7	32.9
120	40.6 ± 0.4	42.2	191.9 ± 4.2	177.6	321.4 ± 9.7	302.9	48.6 ± 3.8	48.2
140	39.5 ± 0.3	40.7	144.1 ± 7.5	133.8	392.7 ± 22.6	364.5	73.0 ± 26.0	56.4
160	39.1 ± 0.1	39.9	124.8 ± 3.9	116.0	437.8 ± 14.9	411.2	60.9 ± 2.0	62.3
180	39.0 ± 0.2	39.6	112.1 ± 2.4	106.1	477.7 ± 7.7	449.2	69.5 ± 3.6	67.0
200	38.8 ± 0.1	39.3	103.7 ± 2.3	99.6	512.8 ± 14.5	481.8	72.5 ± 4.7	71.1

S17.5 Data of thermodynamics and transport properties of CO_2 rich quaternary mixture with 1 mole% impurity of Ar, 1 mole% impurity of H_2, and 2 mole% impurity of CH_4

Table S395: Densities computed from MC and MD simulations (ρ^{MC} and ρ^{MD}), densities obtained from REFPROP[10] (ρ^{REFP}), isothermal compressibilities computed from MC simulations (β_T^{MC}), isothermal compressibilities obtained from REFPROP[10] (β_T^{REFP}), thermal expansion coefficients computed from MC simulations (α_P^{MC}), thermal expansion coefficients obtained from REFPROP[10] (α_P^{REFP}), Joule Thomson coefficients computed from MC simulations (μ_{JT}^{MC}), and Joule Thomson coefficients obtained from REFPROP[10] (μ_{JT}^{REFP}) of CO_2 rich quaternary mixture with 2% impurity of CH_4, 1% impurity of Ar and 1% impurity of H_2 at 253 K and pressures ranging from 20 bar to 200 bar.

P/ [bar]	ρ^{MC}/ [kg/m^3]	ρ^{MD}/ [kg/m^3]	ρ^{REFP}/ [kg/m^3]	β_T^{MC}/ [10^{-5}/bar]	β_T^{REFP}/ [10^{-5}/bar]	α_P^{MC}/ [10^{-4}/K]	α_P^{REFP}/ [10^{-4}/K]	μ_{JT}^{MC}/ [10^{-3} K/bar]	μ_{JT}^{REFP}/ [10^{-3} K/bar]
20	49.3	50.0 ± 0.1	50.7	6293.9 ± 50.4	6517.2	74.3 ± 0.6	81.6	1510.1 ± 27.6	1651.0
40	993.5 ± 1.2	990.6 ± 0.7	882.2	43.9 ± 1.8	309.3	47.9 ± 2.0	189.9	9.4 ± 2.2	–
60	1000.7 ± 0.8	999.1 ± 1.0	997.2	41.1 ± 1.4	46.3	45.6 ± 1.4	46.6	6.9 ± 1.5	8.4
80	1009.2 ± 1.7	1006.9 ± 0.7	1006.0	37.7 ± 1.4	42.2	43.3 ± 1.2	43.8	4.3 ± 1.4	5.2
100	1015.7 ± 0.8	1013.1 ± 0.5	1014.2	35.5 ± 2.2	38.9	41.4 ± 2.6	41.5	2.2 ± 3.0	2.4
120	1022.7 ± 1.3	1021.7 ± 0.5	1021.8	32.9 ± 1.5	36.1	39.2 ± 1.6	39.5	-0.4 ± 1.9	0.0
140	1029.2 ± 0.7	1027.8 ± 0.5	1029.0	30.5 ± 1.6	33.8	37.2 ± 2.0	37.8	-2.8 ± 2.4	−2.1
160	1035.5 ± 1.1	1034.2 ± 0.9	1035.7	29.2 ± 1.1	31.7	36.5 ± 1.5	36.3	-3.7 ± 1.8	−4.0
180	1041.2 ± 1.0	1039.9 ± 0.3	1042.1	27.2 ± 1.0	29.9	34.3 ± 1.6	35.0	-6.3 ± 1.9	−5.7
200	1045.9 ± 0.9	1045.8 ± 0.3	1048.2	25.8 ± 0.8	28.4	33.3 ± 1.1	33.8	-7.6 ± 1.4	−7.2

Table S396: Heat capacities at constant volume computed from MC simulations (c_V^{MC}), heat capacities at constant volume obtained from REFPROP[10] (c_V^{REFP}), heat capacities at constant pressure computed from MC simulations (c_P^{MC}), heat capacities at constant pressure obtained from REFPROP[10] (c_P^{REFP}), speed of sound computed from MC simulations (c^{MC}), speed of sound obtained from REFPROP[10] (c^{REFP}), viscosities computed from MD simulations (η^{MD}), and viscosities obtained from REFPROP[10] (η^{REFP}) of CO_2 rich quaternary mixture with 2% impurity of CH_4, 1% impurity of Ar and 1% impurity of H_2 at 253 K and pressures ranging from 20 bar to 200 bar.

P/ [bar]	c_V^{MC}/ [J/mol K]	c_V^{REFP}/ [J/mol K]	c_P^{MC}/ [J/mol K]	c_P^{REFP}/ [J/mol K]	c^{MC}/ [m/s]	c^{REFP}/ [m/s]	η^{MD}/ [μPa s]	η^{REFP}/ [μPa s]
20	31.5	32.8	50.9 ± 0.2	54.8	228.2 ± 1.1	224.8	11.1 ± 2.2	13.0
40	40.3 ± 0.3	–	97.5 ± 2.4	–	744.5 ± 18.0	–	130.4 ± 3.1	91.4
60	40.1 ± 0.2	40.1	95.4 ± 1.6	91.2	760.9 ± 14.4	702.4	143.0 ± 6.4	129.4
80	40.2 ± 0.3	40.1	93.8 ± 1.3	89.2	783.2 ± 15.7	724.1	142.9 ± 5.9	133.2
100	40.2 ± 0.2	40.1	91.8 ± 3.3	87.5	795.6 ± 28.9	744.2	172.3 ± 39.4	136.8
120	40.1 ± 0.2	40.1	90.1 ± 2.2	86.1	817.0 ± 20.9	763.0	151.1 ± 7.0	140.4
140	40.2 ± 0.1	40.1	88.3 ± 2.6	84.9	837.4 ± 24.9	780.6	151.9 ± 7.3	143.7
160	40.4 ± 0.3	40.1	88.1 ± 2.3	83.8	849.5 ± 20.1	797.3	154.1 ± 8.4	147.1
180	40.3 ± 0.3	40.2	85.5 ± 2.6	82.9	864.8 ± 21.2	813.1	161.1 ± 2.8	150.3
200	40.5 ± 0.1	40.2	85.1 ± 1.9	82.0	882.2 ± 16.3	828.2	163.5 ± 13.6	153.4

Table S397: Densities computed from MC and MD simulations (ρ^{MC} and ρ^{MD}), densities obtained from REFPROP[10] (ρ^{REFP}), isothermal compressibilities computed from MC simulations (β_T^{MC}), isothermal compressibilities obtained from REFPROP[10] (β_T^{REFP}), thermal expansion coefficients computed from MC simulations (α_P^{MC}), thermal expansion coefficients obtained from REFPROP[10] (α_P^{REFP}), Joule Thomson coefficients computed from MC simulations ($\mu_{\mathrm{JT}}^{\mathrm{MC}}$), and Joule Thomson coefficients obtained from REFPROP[10] ($\mu_{\mathrm{JT}}^{\mathrm{REFP}}$) of CO_2 rich quaternary mixture with 2% impurity of CH_4, 1% impurity of Ar and 1% impurity of H_2 at 273 K and pressures ranging from 20 bar to 200 bar.

P/ [bar]	ρ^{MC}/ [kg/m^3]	ρ^{MD}/ [kg/m^3]	ρ^{REFP}/ [kg/m^3]	β_T^{MC}/ [10^{-5}/bar]	β_T^{REFP}/ [10^{-5}/bar]	α_P^{MC}/ [10^{-4}/K]	α_P^{REFP}/ [10^{-4}/K]	$\mu_{\mathrm{JT}}^{\mathrm{MC}}$/ [$10^{-3}$ K/bar]	$\mu_{\mathrm{JT}}^{\mathrm{REFP}}$/ [$10^{-3}$ K/bar]
20	43.3	44.0 ± 0.1	44.1	5843.3 ± 27.9	5941.2	57.4 ± 0.2	60.2	1224.7 ± 11.5	1330.2
40	108.7 ± 0.2	874.5 ± 1.4	262.7	4403.7 ± 88.6	-2619.5	135.3 ± 3.3	-450.3	1301.7 ± 48.1	–
60	895.1 ± 3.1	895.0 ± 1.1	887.8	95.2 ± 5.7	109.8	73.1 ± 3.6	75.4	42.0 ± 4.4	47.1
80	910.9 ± 1.5	907.7 ± 1.6	905.4	76.6 ± 7.0	88.6	62.5 ± 5.0	65.0	31.5 ± 6.3	36.1
100	923.4 ± 1.2	921.3 ± 0.7	920.2	65.2 ± 3.8	74.8	55.6 ± 2.9	58.0	24.1 ± 3.7	28.0
120	936.2 ± 1.5	933.6 ± 0.7	933.1	56.5 ± 4.0	65.1	50.3 ± 3.7	52.8	17.9 ± 4.9	21.7
140	945.6 ± 0.7	943.4 ± 1.0	944.7	52.9 ± 1.1	57.8	49.2 ± 1.0	48.8	16.2 ± 1.3	16.6
160	954.3 ± 1.4	954.1 ± 0.8	955.1	48.9 ± 2.7	52.1	46.4 ± 2.1	45.6	12.8 ± 2.8	12.4
180	963.1 ± 0.5	962.3 ± 1.0	964.6	43.1 ± 1.3	47.5	42.4 ± 1.3	42.9	7.8 ± 1.7	8.8
200	972.8 ± 0.9	970.6 ± 0.5	973.5	39.8 ± 2.7	43.8	40.2 ± 2.7	40.7	4.9 ± 3.7	5.7

Table S398: Heat capacities at constant volume computed from MC simulations (c_V^{MC}), heat capacities at constant volume obtained from REFPROP[10] (c_V^{REFP}), heat capacities at constant pressure computed from MC simulations (c_P^{MC}), heat capacities at constant pressure obtained from REFPROP[10] (c_P^{REFP}), speed of sound computed from MC simulations (c^{MC}), speed of sound obtained from REFPROP[10] (c^{REFP}), viscosities computed from MD simulations (η^{MD}), and viscosities obtained from REFPROP[10] (η^{REFP}) of CO_2 rich quaternary mixture with 2% impurity of CH_4, 1% impurity of Ar and 1% impurity of H_2 at 273 K and pressures ranging from 20 bar to 200 bar.

P/ [bar]	c_V^{MC}/ [J/mol K]	c_V^{REFP}/ [J/mol K]	c_P^{MC}/ [J/mol K]	c_P^{REFP}/ [J/mol K]	c^{MC}/ [m/s]	c^{REFP}/ [m/s]	η^{MD}/ [μPa s]	η^{REFP}/ [μPa s]
20	30.7	31.0	45.9 ± 0.1	47.2	243.3 ± 0.6	241.2	13.9 ± 3.0	14.0
40	36.6 ± 0.2	–	81.9 ± 1.3	–	216.3 ± 2.8	–	94.7 ± 3.4	19.3
60	39.5 ± 0.1	40.3	113.9 ± 3.6	108.8	581.5 ± 19.7	526.3	100.3 ± 2.2	91.9
80	39.5 ± 0.2	40.0	105.7 ± 4.6	101.9	619.7 ± 31.6	563.5	102.8 ± 0.8	96.6
100	39.6 ± 0.1	39.8	100.2 ± 3.0	97.1	649.0 ± 21.4	595.0	122.3 ± 35.5	100.9
120	39.6 ± 0.2	39.7	96.0 ± 4.5	93.6	677.6 ± 28.9	622.6	106.4 ± 4.0	104.8
140	39.6 ± 0.2	39.7	96.4 ± 1.1	90.9	697.1 ± 8.6	647.4	117.7 ± 7.9	108.4
160	39.7 ± 0.2	39.7	93.9 ± 2.4	88.7	712.7 ± 21.8	670.1	124.6 ± 6.2	111.8
180	39.8 ± 0.1	39.7	90.4 ± 1.7	86.8	739.9 ± 13.1	691.0	120.7 ± 3.7	115.1
200	39.6 ± 0.1	39.7	88.6 ± 3.4	85.3	760.3 ± 29.9	710.5	131.3 ± 9.1	118.3

Table S399: Densities computed from MC and MD simulations (ρ^{MC} and ρ^{MD}), densities obtained from REFPROP[10] (ρ^{REFP}), isothermal compressibilities computed from MC simulations (β_T^{MC}), isothermal compressibilities obtained from REFPROP[10] (β_T^{REFP}), thermal expansion coefficients computed from MC simulations (α_P^{MC}), thermal expansion coefficients obtained from REFPROP[10] (α_P^{REFP}), Joule Thomson coefficients computed from MC simulations (μ_{JT}^{MC}), and Joule Thomson coefficients obtained from REFPROP[10] (μ_{JT}^{REFP}) of CO_2 rich quaternary mixture with 2% impurity of CH_4, 1% impurity of Ar and 1% impurity of H_2 at 293 K and pressures ranging from 20 bar to 200 bar.

P/ [bar]	ρ^{MC}/ [kg/m^3]	ρ^{MD}/ [kg/m^3]	ρ^{REFP}/ [kg/m^3]	β_T^{MC}/ [10^{-5}/bar]	β_T^{REFP}/ [10^{-5}/bar]	α_P^{MC}/ [10^{-4}/K]	α_P^{REFP}/ [10^{-4}/K]	μ_{JT}^{MC}/ [10^{-3} K/bar]	μ_{JT}^{REFP}/ [10^{-3} K/bar]
20	39.0	39.5 ± 0.1	39.6	5600.6 ± 43.4	5659.9	48.0 ± 0.4	49.5	1020.4 ± 27.7	1101.0
40	90.0 ± 0.1	91.6 ± 0.4	93.0	3317.9 ± 17.1	3509.2	75.5 ± 0.5	84.1	1006.3 ± 12.9	1097.8
60	174.7 ± 0.4	193.7 ± 15.8	190.5	3790.5 ± 121.6	4439.7	205.4 ± 8.1	272.1	1022.3 ± 58.6	1023.3
80	756.8 ± 5.3	753.6 ± 3.0	744.0	315.1 ± 39.9	404.5	147.4 ± 11.4	167.8	119.7 ± 12.9	141.3
100	793.6 ± 4.5	791.3 ± 2.4	788.6	210.2 ± 36.0	218.7	112.9 ± 15.0	107.7	91.1 ± 18.7	93.6
120	821.0 ± 1.2	819.1 ± 3.2	817.9	131.6 ± 7.1	153.8	79.3 ± 4.6	84.4	61.1 ± 6.7	69.4
140	841.7 ± 1.3	840.3 ± 1.0	840.3	105.4 ± 2.8	119.8	69.0 ± 1.9	71.3	48.6 ± 2.9	53.9
160	859.4 ± 1.3	858.6 ± 0.5	858.7	91.3 ± 3.4	98.7	62.9 ± 2.5	62.6	40.9 ± 3.8	42.7
180	873.5 ± 1.4	871.5 ± 1.4	874.5	76.3 ± 2.4	84.2	55.5 ± 1.7	56.4	31.5 ± 2.7	34.2
200	888.2 ± 1.8	884.6 ± 0.9	888.4	68.7 ± 3.7	73.6	52.4 ± 2.4	51.7	27.0 ± 3.6	27.4

Table S400: Heat capacities at constant volume computed from MC simulations (c_V^{MC}), heat capacities at constant volume obtained from REFPROP[10] (c_V^{REFP}), heat capacities at constant pressure computed from MC simulations (c_P^{MC}), heat capacities at constant pressure obtained from REFPROP[10] (c_P^{REFP}), speed of sound computed from MC simulations (c^{MC}), speed of sound obtained from REFPROP[10] (c^{REFP}), viscosities computed from MD simulations (η^{MD}), and viscosities obtained from REFPROP[10] (η^{REFP}) of CO_2 rich quaternary mixture with 2% impurity of CH_4, 1% impurity of Ar and 1% impurity of H_2 at 293 K and pressures ranging from 20 bar to 200 bar.

P/ [bar]	c_V^{MC}/ [J/mol K]	c_V^{REFP}/ [J/mol K]	c_P^{MC}/ [J/mol K]	c_P^{REFP}/ [J/mol K]	c^{MC}/ [m/s]	c^{REFP}/ [m/s]	η^{MD}/ [μPa s]	η^{REFP}/ [μPa s]
20	30.6	30.7	43.9 ± 0.1	44.5	256.2 ± 1.0	254.4	7.2 ± 3.5	15.0
40	33.5 ± 0.1	34.4	57.6 ± 0.2	61.6	239.9 ± 0.8	234.5	16.4 ± 2.5	15.7
60	39.3 ± 0.3	43.5	120.8 ± 3.9	153.7	215.6 ± 5.0	204.4	18.7 ± 3.7	17.8
80	39.9 ± 0.3	42.3	157.6 ± 5.9	160.2	406.8 ± 26.9	354.6	67.2 ± 1.3	62.9
100	39.6 ± 0.2	40.8	137.3 ± 10.4	125.5	455.8 ± 42.7	422.3	76.1 ± 7.5	70.5
120	39.4 ± 0.1	40.2	113.5 ± 4.5	111.5	516.6 ± 17.4	469.7	84.3 ± 1.4	76.0
140	39.2 ± 0.3	39.8	107.2 ± 1.9	103.3	555.1 ± 9.1	507.6	75.5 ± 13.1	80.7
160	39.4 ± 0.2	39.6	103.1 ± 2.8	97.9	577.4 ± 13.4	539.8	93.4 ± 6.3	84.7
180	39.1 ± 0.2	39.5	97.5 ± 1.9	93.9	611.2 ± 11.6	568.1	90.8 ± 2.8	88.4
200	39.1 ± 0.1	39.4	96.1 ± 2.4	90.8	634.4 ± 18.8	593.7	91.2 ± 13.3	91.8

Table S401: Densities computed from MC and MD simulations (ρ^{MC} and ρ^{MD}), densities obtained from REFPROP[10] (ρ^{REFP}), isothermal compressibilities computed from MC simulations (β_T^{MC}), isothermal compressibilities obtained from REFPROP[10] (β_T^{REFP}), thermal expansion coefficients computed from MC simulations (α_P^{MC}), thermal expansion coefficients obtained from REFPROP[10] (α_P^{REFP}), Joule Thomson coefficients computed from MC simulations ($\mu_{\mathrm{JT}}^{\mathrm{MC}}$), and Joule Thomson coefficients obtained from REFPROP[10] ($\mu_{\mathrm{JT}}^{\mathrm{REFP}}$) of CO_2 rich quaternary mixture with 2% impurity of CH_4, 1% impurity of Ar and 1% impurity of H_2 at 313 K and pressures ranging from 20 bar to 200 bar.

P/ [bar]	ρ^{MC}/ [kg/m^3]	ρ^{MD}/ [kg/m^3]	ρ^{REFP}/ [kg/m^3]	β_T^{MC}/ [10^{-5}/bar]	β_T^{REFP}/ [10^{-5}/bar]	α_P^{MC}/ [10^{-4}/K]	α_P^{REFP}/ [10^{-4}/K]	$\mu_{\mathrm{JT}}^{\mathrm{MC}}$/ [$10^{-3}$ K/bar]	$\mu_{\mathrm{JT}}^{\mathrm{REFP}}$/ [$10^{-3}$ K/bar]
20	35.7	36.2	36.1	5445.2 ± 40.2	5489.9	41.7 ± 0.4	42.7	859.1 ± 33.2	927.9
40	78.8 ± 0.1	80.1 ± 0.1	80.7	3046.6 ± 17.1	3133.9	57.6 ± 0.4	61.2	853.5 ± 13.1	919.1
60	134.9 ± 0.1	137.8 ± 0.8	140.7	2427.1 ± 36.6	2576.1	89.1 ± 2.0	99.6	837.9 ± 32.7	886.6
80	222.9 ± 1.0	231.7 ± 3.3	238.7	2790.4 ± 152.6	2893.7	200.7 ± 13.4	220.9	801.3 ± 76.3	782.4
100	449.2 ± 4.0	454.2 ± 5.1	471.4	3259.3 ± 167.8	2934.2	583.4 ± 25.9	533.1	456.2 ± 27.7	443.4
120	628.0 ± 3.7	624.9 ± 3.7	630.2	767.6 ± 103.3	704.5	238.7 ± 27.0	209.4	218.7 ± 33.6	216.2
140	695.9 ± 3.8	691.1 ± 2.7	694.8	323.5 ± 9.5	348.6	129.0 ± 4.1	128.8	133.8 ± 6.4	141.0
160	732.1 ± 1.7	730.9 ± 1.6	734.9	235.0 ± 21.4	229.7	105.2 ± 7.8	97.3	106.1 ± 12.1	103.5
180	761.9 ± 2.3	761.3 ± 1.1	764.6	162.6 ± 11.0	171.2	82.3 ± 5.4	80.2	78.6 ± 9.1	80.2
200	784.9 ± 1.9	782.9 ± 1.1	788.2	134.3 ± 9.1	136.5	72.7 ± 4.1	69.2	65.3 ± 6.9	64.0

Table S402: Heat capacities at constant volume computed from MC simulations (c_V^{MC}), heat capacities at constant volume obtained from REFPROP[10] (c_V^{REFP}), heat capacities at constant pressure computed from MC simulations (c_P^{MC}), heat capacities at constant pressure obtained from REFPROP[10] (c_P^{REFP}), speed of sound computed from MC simulations (c^{MC}), speed of sound obtained from REFPROP[10] (c^{REFP}), viscosities computed from MD simulations (η^{MD}), and viscosities obtained from REFPROP[10] (η^{REFP}) of CO_2 rich quaternary mixture with 2% impurity of CH_4, 1% impurity of Ar and 1% impurity of H_2 at 313 K and pressures ranging from 20 bar to 200 bar.

P/ [bar]	c_V^{MC}/ [J/mol K]	c_V^{REFP}/ [J/mol K]	c_P^{MC}/ [J/mol K]	c_P^{REFP}/ [J/mol K]	c^{MC}/ [m/s]	c^{REFP}/ [m/s]	η^{MD}/ [μPa s]	η^{REFP}/ [μPa s]
20	31.0	31.0	43.0 ± 0.1	43.4	267.5 ± 1.1	265.9	3.9 ± 3.7	16.0
40	32.8	33.2	51.3 ± 0.1	53.1	255.5 ± 0.8	251.7	17.7 ± 2.4	16.5
60	35.3	36.2	68.1 ± 1.1	73.0	242.6 ± 2.6	236.0	17.5 ± 0.6	17.7
80	39.0 ± 0.2	41.1	127.2 ± 6.7	136.1	229.1 ± 8.7	219.1	21.0 ± 2.2	20.5
100	43.0 ± 0.7	46.1	362.3 ± 13.7	322.6	240.0 ± 8.0	224.8	31.5 ± 2.1	33.4
120	40.5 ± 0.6	42.4	202.6 ± 16.2	175.3	322.1 ± 25.3	305.1	45.0 ± 1.4	48.3
140	39.7 ± 0.4	40.9	140.3 ± 3.0	132.9	396.4 ± 7.6	366.5	53.7 ± 1.0	56.4
160	39.4 ± 0.2	40.1	126.9 ± 5.3	115.6	432.4 ± 21.7	413.1	64.0 ± 2.2	62.2
180	39.1 ± 0.2	39.7	113.2 ± 5.0	105.8	483.2 ± 19.5	451.1	72.7 ± 9.1	66.9
200	39.0 ± 0.1	39.5	107.0 ± 3.4	99.4	509.9 ± 19.2	483.7	73.3 ± 8.9	70.9

S17.6 Data of thermodynamics and transport properties of CO_2 rich quaternary mixture with 1 mole% impurity of Ar, 2 mole% impurity of H_2, and 1 mole% impurity of CH_4

Table S403: Densities computed from MC and MD simulations (ρ^{MC} and ρ^{MD}), densities obtained from REFPROP[10] (ρ^{REFP}), isothermal compressibilities computed from MC simulations (β_T^{MC}), isothermal compressibilities obtained from REFPROP[10] (β_T^{REFP}), thermal expansion coefficients computed from MC simulations (α_P^{MC}), thermal expansion coefficients obtained from REFPROP[10] (α_P^{REFP}), Joule Thomson coefficients computed from MC simulations (μ_{JT}^{MC}), and Joule Thomson coefficients obtained from REFPROP[10] (μ_{JT}^{REFP}) of CO_2 rich quaternary mixture with 2% impurity of H_2, 1% impurity of Ar and 1% impurity of CH_4 at 253 K and pressures ranging from 20 bar to 200 bar.

P/ [bar]	ρ^{MC}/ [kg/m^3]	ρ^{MD}/ [kg/m^3]	ρ^{REFP}/ [kg/m^3]	β_T^{MC}/ [10^{-5}/bar]	β_T^{REFP}/ [10^{-5}/bar]	α_P^{MC}/ [10^{-4}/K]	α_P^{REFP}/ [10^{-4}/K]	μ_{JT}^{MC}/ [10^{-3} K/bar]	μ_{JT}^{REFP}/ [10^{-3} K/bar]
20	49.0	49.6 ± 0.1	50.3	6268.6 ± 80.3	6479.1	73.8 ± 1.1	80.4	1498.1 ± 47.1	1631.7
40	989.9 ± 1.3	986.7 ± 0.7	728.2	46.4 ± 1.8	-141.4	49.9 ± 1.5	7.2	11.5 ± 1.7	–
60	997.0 ± 1.3	995.0 ± 1.3	982.8	42.8 ± 1.1	51.4	46.8 ± 1.0	49.7	8.2 ± 1.1	–
80	1005.8 ± 1.6	1002.8 ± 1.2	998.8	38.6 ± 1.6	43.3	43.3 ± 2.1	44.4	4.4 ± 2.4	5.9
100	1013.5 ± 0.9	1010.3 ± 1.1	1007.1	35.8 ± 2.3	39.9	41.5 ± 3.1	42.0	2.3 ± 3.6	3.0
120	1020.2 ± 0.2	1018.7 ± 0.5	1014.9	33.3 ± 0.9	37.0	39.3 ± 1.5	39.9	-0.3 ± 1.8	0.5
140	1026.8 ± 1.3	1025.9 ± 0.6	1022.2	31.6 ± 0.6	34.5	38.2 ± 1.3	38.2	-1.6 ± 1.5	-1.7
160	1032.2 ± 0.7	1032.0 ± 0.3	1029.0	29.8 ± 1.0	32.4	36.5 ± 1.1	36.6	-3.7 ± 1.3	-3.6
180	1039.4 ± 1.8	1036.7 ± 0.8	1035.5	27.7 ± 1.1	30.5	35.0 ± 1.4	35.3	-5.5 ± 1.7	-5.4
200	1044.7 ± 1.3	1043.3 ± 0.2	1041.7	27.2 ± 1.1	28.9	34.8 ± 1.5	34.0	-5.6 ± 1.9	-7.0

Table S404: Heat capacities at constant volume computed from MC simulations (c_V^{MC}), heat capacities at constant volume obtained from REFPROP[10] (c_V^{REFP}), heat capacities at constant pressure computed from MC simulations (c_P^{MC}), heat capacities at constant pressure obtained from REFPROP[10] (c_P^{REFP}), speed of sound computed from MC simulations (c^{MC}), speed of sound obtained from REFPROP[10] (c^{REFP}), viscosities computed from MD simulations (η^{MD}), and viscosities obtained from REFPROP[10] (η^{REFP}) of CO_2 rich quaternary mixture with 2% impurity of H_2, 1% impurity of Ar and 1% impurity of CH_4 at 253 K and pressures ranging from 20 bar to 200 bar.

P/ [bar]	c_V^{MC}/ [J/mol K]	c_V^{REFP}/ [J/mol K]	c_P^{MC}/ [J/mol K]	c_P^{REFP}/ [J/mol K]	c^{MC}/ [m/s]	c^{REFP}/ [m/s]	η^{MD}/ [μPa s]	η^{REFP}/ [μPa s]
20	31.4	32.5	50.6 ± 0.3	54.0	229.2 ± 1.6	225.7	10.8 ± 2.8	13.0
40	40.3 ± 0.2	–	99.1 ± 1.8	–	731.6 ± 15.7	–	129.1 ± 3.0	62.9
60	40.1 ± 0.3	–	95.7 ± 1.4	–	747.6 ± 11.3	–	133.7 ± 2.6	123.6
80	40.0 ± 0.2	40.0	92.6 ± 2.8	89.3	771.5 ± 20.2	718.3	141.1 ± 1.9	130.2
100	40.3 ± 0.2	40.0	91.6 ± 4.6	87.5	791.1 ± 32.6	738.7	140.3 ± 7.0	133.8
120	40.3 ± 0.3	40.0	89.6 ± 2.6	86.1	809.1 ± 16.2	757.7	151.0 ± 10.1	137.3
140	40.1 ± 0.3	40.0	89.0 ± 2.5	84.8	827.3 ± 14.1	775.5	142.8 ± 2.6	140.6
160	40.2 ± 0.2	40.0	87.1 ± 1.4	83.7	839.6 ± 15.8	792.4	164.2 ± 16.3	143.9
180	40.4 ± 0.2	40.1	86.3 ± 1.9	82.7	861.1 ± 19.4	808.4	156.8 ± 9.0	147.1
200	40.3 ± 0.3	40.1	86.5 ± 2.4	81.9	868.5 ± 21.6	823.6	163.9 ± 8.5	150.2

Table S405: Densities computed from MC and MD simulations (ρ^{MC} and ρ^{MD}), densities obtained from REFPROP[10] (ρ^{REFP}), isothermal compressibilities computed from MC simulations (β_T^{MC}), isothermal compressibilities obtained from REFPROP[10] (β_T^{REFP}), thermal expansion coefficients computed from MC simulations (α_P^{MC}), thermal expansion coefficients obtained from REFPROP[10] (α_P^{REFP}), Joule Thomson coefficients computed from MC simulations (μ_{JT}^{MC}), and Joule Thomson coefficients obtained from REFPROP[10] (μ_{JT}^{REFP}) of CO_2 rich quaternary mixture with 2% impurity of H_2, 1% impurity of Ar and 1% impurity of CH_4 at 273 K and pressures ranging from 20 bar to 200 bar.

P/ [bar]	ρ^{MC}/ [kg/m^3]	ρ^{MD}/ [kg/m^3]	ρ^{REFP}/ [kg/m^3]	β_T^{MC}/ [10^{-5}/bar]	β_T^{REFP}/ [10^{-5}/bar]	α_P^{MC}/ [10^{-4}/K]	α_P^{REFP}/ [10^{-4}/K]	μ_{JT}^{MC}/ [10^{-3} K/bar]	μ_{JT}^{REFP}/ [10^{-3} K/bar]
20	43.0	43.7 ± 0.1	43.9	5794.7 ± 28.0	5925.2	56.7 ± 0.5	59.8	1194.3 ± 27.8	1316.7
40	107.6 ± 0.2	865.4 ± 2.6	238.9	4235.3 ± 97.9	-4795.6	129.3 ± 3.5	-616.2	1265.4 ± 53.9	–
60	886.0 ± 1.7	886.9 ± 0.9	828.3	104.5 ± 2.8	246.5	77.2 ± 1.3	134.7	46.1 ± 1.6	–
80	904.1 ± 2.2	902.7 ± 1.0	897.3	80.4 ± 3.7	92.2	64.3 ± 2.3	66.5	33.6 ± 2.9	38.0
100	917.8 ± 2.3	916.0 ± 1.1	912.5	70.9 ± 3.8	77.4	59.1 ± 2.5	59.0	27.8 ± 3.2	29.5
120	930.0 ± 1.5	928.4 ± 0.6	925.8	61.4 ± 3.7	67.1	53.2 ± 2.7	53.6	21.3 ± 3.6	22.9
140	940.2 ± 2.1	939.8 ± 0.9	937.5	54.8 ± 3.1	59.4	49.4 ± 2.3	49.5	16.7 ± 3.0	17.6
160	950.1 ± 1.7	949.3 ± 0.8	948.1	48.8 ± 1.6	53.4	45.9 ± 1.9	46.1	12.2 ± 2.5	13.2
180	959.7 ± 1.8	958.2 ± 0.5	957.8	45.1 ± 2.1	48.6	43.6 ± 1.9	43.4	9.3 ± 2.5	9.5
200	968.1 ± 1.0	966.7 ± 0.6	966.8	41.7 ± 2.4	44.7	41.4 ± 2.4	41.1	6.5 ± 3.3	6.3

Table S406: Heat capacities at constant volume computed from MC simulations (c_V^{MC}), heat capacities at constant volume obtained from REFPROP[10] (c_V^{REFP}), heat capacities at constant pressure computed from MC simulations (c_P^{MC}), heat capacities at constant pressure obtained from REFPROP[10] (c_P^{REFP}), speed of sound computed from MC simulations (c^{MC}), speed of sound obtained from REFPROP[10] (c^{REFP}), viscosities computed from MD simulations (η^{MD}), and viscosities obtained from REFPROP[10] (η^{REFP}) of CO_2 rich quaternary mixture with 2% impurity of H_2, 1% impurity of Ar and 1% impurity of CH_4 at 273 K and pressures ranging from 20 bar to 200 bar.

P/ [bar]	c_V^{MC}/ [J/mol K]	c_V^{REFP}/ [J/mol K]	c_P^{MC}/ [J/mol K]	c_P^{REFP}/ [J/mol K]	c^{MC}/ [m/s]	c^{REFP}/ [m/s]	η^{MD}/ [μPa s]	η^{REFP}/ [μPa s]
20	30.6	30.8	45.6 ± 0.2	46.9	244.6 ± 0.8	241.9	10.0 ± 3.0	14.0
40	36.7 ± 0.2	–	79.6 ± 1.5	–	218.2 ± 3.3	–	90.1 ± 2.1	18.3
60	39.9 ± 0.2	–	116.1 ± 1.2	–	560.8 ± 8.3	–	97.5 ± 3.7	77.9
80	39.5 ± 0.1	39.9	106.5 ± 2.3	102.4	609.3 ± 15.5	557.1	100.2 ± 2.1	94.3
100	39.5 ± 0.3	39.7	102.9 ± 2.6	97.4	632.7 ± 19.0	589.2	100.5 ± 3.2	98.5
120	39.6 ± 0.1	39.6	97.9 ± 2.8	93.8	658.1 ± 22.0	617.3	108.6 ± 5.1	102.4
140	39.6 ± 0.2	39.6	95.2 ± 2.2	90.9	683.6 ± 21.2	642.5	114.4 ± 5.7	106.1
160	39.5 ± 0.1	39.5	93.1 ± 2.8	88.6	713.1 ± 16.0	665.5	114.1 ± 4.9	109.5
180	39.5 ± 0.2	39.5	91.1 ± 2.3	86.8	730.2 ± 19.3	686.6	113.8 ± 7.5	112.7
200	39.6 ± 0.1	39.5	89.5 ± 3.1	85.2	748.5 ± 25.0	706.3	121.6 ± 6.5	115.9

Table S407: Densities computed from MC and MD simulations (ρ^{MC} and ρ^{MD}), densities obtained from REFPROP[10] (ρ^{REFP}), isothermal compressibilities computed from MC simulations (β_T^{MC}), isothermal compressibilities obtained from REFPROP[10] (β_T^{REFP}), thermal expansion coefficients computed from MC simulations (α_P^{MC}), thermal expansion coefficients obtained from REFPROP[10] (α_P^{REFP}), Joule Thomson coefficients computed from MC simulations ($\mu_{\mathrm{JT}}^{\mathrm{MC}}$), and Joule Thomson coefficients obtained from REFPROP[10] ($\mu_{\mathrm{JT}}^{\mathrm{REFP}}$) of CO_2 rich quaternary mixture with 2% impurity of H_2, 1% impurity of Ar and 1% impurity of CH_4 at 293 K and pressures ranging from 20 bar to 200 bar.

P/ [bar]	ρ^{MC}/ [kg/m^3]	ρ^{MD}/ [kg/m^3]	ρ^{REFP}/ [kg/m^3]	β_T^{MC}/ [10^{-5}/bar]	β_T^{REFP}/ [10^{-5}/bar]	α_P^{MC}/ [10^{-4}/K]	α_P^{REFP}/ [10^{-4}/K]	$\mu_{\mathrm{JT}}^{\mathrm{MC}}$/ [$10^{-3}$ K/bar]	$\mu_{\mathrm{JT}}^{\mathrm{REFP}}$/ [$10^{-3}$ K/bar]
20	38.8	39.4 ± 0.1	39.4	5608.1 ± 13.8	5650.7	47.9 ± 0.1	49.3	1014.4 ± 10.0	1091.1
40	89.2 ± 0.1	90.9 ± 0.3	92.3	3332.8 ± 42.9	3484.4	75.4 ± 1.0	82.7	1010.6 ± 25.9	1087.5
60	170.5 ± 0.6	179.3 ± 1.3	186.2	3553.5 ± 98.5	4179.1	187.1 ± 6.1	247.3	1004.8 ± 49.0	1019.2
80	744.2 ± 10.6	733.0 ± 3.5	730.7	446.0 ± 172.2	456.1	189.1 ± 55.1	182.4	144.4 ± 57.4	151.9
100	783.5 ± 5.9	781.2 ± 2.6	778.7	226.0 ± 29.7	233.1	117.5 ± 10.8	112.0	95.7 ± 13.2	98.5
120	814.0 ± 2.0	809.6 ± 2.0	809.2	153.9 ± 11.4	161.0	89.9 ± 5.4	86.6	70.9 ± 7.4	72.5
140	834.5 ± 2.0	833.1 ± 1.2	832.3	117.6 ± 3.0	124.3	74.6 ± 1.7	72.7	54.8 ± 2.5	56.0
160	852.3 ± 2.1	850.4 ± 1.2	851.2	97.1 ± 5.0	101.9	65.2 ± 2.8	63.6	43.9 ± 4.1	44.3
180	868.8 ± 0.5	867.2 ± 0.7	867.3	83.5 ± 3.7	86.6	59.3 ± 2.6	57.2	36.3 ± 3.8	35.5
200	880.1 ± 2.8	880.1 ± 1.1	881.5	73.0 ± 2.0	75.4	53.9 ± 1.4	52.3	29.3 ± 2.1	28.4

Table S408: Heat capacities at constant volume computed from MC simulations (c_V^{MC}), heat capacities at constant volume obtained from REFPROP[10] (c_V^{REFP}), heat capacities at constant pressure computed from MC simulations (c_P^{MC}), heat capacities at constant pressure obtained from REFPROP[10] (c_P^{REFP}), speed of sound computed from MC simulations (c^{MC}), speed of sound obtained from REFPROP[10] (c^{REFP}), viscosities computed from MD simulations (η^{MD}), and viscosities obtained from REFPROP[10] (η^{REFP}) of CO_2 rich quaternary mixture with 2% impurity of H_2, 1% impurity of Ar and 1% impurity of CH_4 at 293 K and pressures ranging from 20 bar to 200 bar.

P/ [bar]	c_V^{MC}/ [J/mol K]	c_V^{REFP}/ [J/mol K]	c_P^{MC}/ [J/mol K]	c_P^{REFP}/ [J/mol K]	c^{MC}/ [m/s]	c^{REFP}/ [m/s]	η^{MD}/ [μPa s]	η^{REFP}/ [μPa s]
20	30.5	30.6	43.8	44.3	256.6 ± 0.4	255.1	7.2 ± 3.7	15.0
40	33.5	34.1	57.4 ± 0.3	60.8	240.3 ± 1.7	235.5	16.2 ± 2.6	15.7
60	38.8 ± 0.3	42.4	112.0 ± 3.2	141.1	218.2 ± 4.4	206.9	19.8 ± 4.0	17.7
80	40.6 ± 0.5	42.4	181.1 ± 32.1	167.7	366.7 ± 78.0	344.4	63.9 ± 5.0	60.7
100	39.9 ± 0.4	40.7	139.7 ± 6.6	127.6	444.9 ± 31.2	415.4	68.3 ± 2.2	68.6
120	39.4 ± 0.2	40.0	121.4 ± 4.4	112.3	495.9 ± 20.5	464.1	75.2 ± 3.2	74.2
140	39.4 ± 0.3	39.7	111.1 ± 1.9	103.8	536.2 ± 8.4	502.7	84.1 ± 4.8	78.8
160	39.2 ± 0.2	39.5	104.3 ± 2.4	98.1	567.2 ± 16.2	535.4	90.5 ± 9.6	82.9
180	39.3 ± 0.3	39.3	100.3 ± 2.8	94.0	593.4 ± 15.5	564.1	96.2 ± 7.0	86.6
200	39.1 ± 0.1	39.3	96.2 ± 1.6	90.8	618.7 ± 9.9	589.8	90.0 ± 11.4	90.0

Table S409: Densities computed from MC and MD simulations (ρ^{MC} and ρ^{MD}), densities obtained from REFPROP[10] (ρ^{REFP}), isothermal compressibilities computed from MC simulations (β_T^{MC}), isothermal compressibilities obtained from REFPROP[10] (β_T^{REFP}), thermal expansion coefficients computed from MC simulations (α_P^{MC}), thermal expansion coefficients obtained from REFPROP[10] (α_P^{REFP}), Joule Thomson coefficients computed from MC simulations ($\mu_{\mathrm{JT}}^{\mathrm{MC}}$), and Joule Thomson coefficients obtained from REFPROP[10] ($\mu_{\mathrm{JT}}^{\mathrm{REFP}}$) of CO_2 rich quaternary mixture with 2% impurity of H_2, 1% impurity of Ar and 1% impurity of CH_4 at 313 K and pressures ranging from 20 bar to 200 bar.

P/ [bar]	ρ^{MC}/ [kg/m^3]	ρ^{MD}/ [kg/m^3]	ρ^{REFP}/ [kg/m^3]	β_T^{MC}/ [10^{-5}/bar]	β_T^{REFP}/ [10^{-5}/bar]	α_P^{MC}/ [10^{-4}/K]	α_P^{REFP}/ [10^{-4}/K]	$\mu_{\mathrm{JT}}^{\mathrm{MC}}$/ [$10^{-3}$ K/bar]	$\mu_{\mathrm{JT}}^{\mathrm{REFP}}$/ [$10^{-3}$ K/bar]
20	35.5	36.0 ± 0.1	35.9	5434.1 ± 29.9	5483.8	41.6 ± 0.2	42.6	847.3 ± 21.2	920.4
40	78.2 ± 0.1	79.5 ± 0.2	80.2	3061.2 ± 15.7	3122.7	57.5 ± 0.3	60.7	856.7 ± 11.6	911.2
60	133.5 ± 0.1	136.1 ± 0.7	139.4	2428.0 ± 55.1	2550.1	88.1 ± 2.2	97.5	839.3 ± 34.6	879.5
80	216.9 ± 0.6	224.9 ± 1.2	234.3	2532.8 ± 108.9	2794.4	174.3 ± 10.2	208.3	775.7 ± 67.2	–
100	405.3 ± 7.1	421.7 ± 10.4	449.4	3809.8 ± 280.9	3048.5	605.1 ± 53.9	521.0	514.6 ± 66.1	468.9
120	608.8 ± 6.8	605.3 ± 4.6	616.8	796.0 ± 83.0	766.2	235.0 ± 19.9	220.0	227.8 ± 26.5	227.7
140	676.5 ± 3.0	676.0 ± 2.0	684.8	406.0 ± 41.8	368.4	150.7 ± 13.4	132.8	154.2 ± 20.1	146.9
160	722.9 ± 3.0	719.8 ± 1.0	726.3	252.2 ± 17.5	239.2	110.3 ± 6.3	99.4	112.0 ± 9.8	107.2
180	752.0 ± 2.0	751.1 ± 1.9	756.7	176.5 ± 5.9	177.0	85.8 ± 2.8	81.5	84.2 ± 4.8	82.8
200	776.6 ± 1.7	775.8 ± 1.6	780.9	137.5 ± 8.1	140.4	73.0 ± 3.9	70.2	66.7 ± 6.7	65.9

Table S410: Heat capacities at constant volume computed from MC simulations (c_V^{MC}), heat capacities at constant volume obtained from REFPROP[10] (c_V^{REFP}), heat capacities at constant pressure computed from MC simulations (c_P^{MC}), heat capacities at constant pressure obtained from REFPROP[10] (c_P^{REFP}), speed of sound computed from MC simulations (c^{MC}), speed of sound obtained from REFPROP[10] (c^{REFP}), viscosities computed from MD simulations (η^{MD}), and viscosities obtained from REFPROP[10] (η^{REFP}) of CO_2 rich quaternary mixture with 2% impurity of H_2, 1% impurity of Ar and 1% impurity of CH_4 at 313 K and pressures ranging from 20 bar to 200 bar.

P/ [bar]	c_V^{MC}/ [J/mol K]	c_V^{REFP}/ [J/mol K]	c_P^{MC}/ [J/mol K]	c_P^{REFP}/ [J/mol K]	c^{MC}/ [m/s]	c^{REFP}/ [m/s]	η^{MD}/ [μPa s]	η^{REFP}/ [μPa s]
20	30.9	30.9	42.9 ± 0.1	43.2	268.3 ± 0.8	266.5	7.9 ± 4.9	16.0
40	32.7	33.0	51.2 ± 0.1	52.7	255.8 ± 0.7	252.6	17.1 ± 1.5	16.5
60	35.1 ± 0.1	35.9	67.3 ± 0.9	71.7	243.2 ± 3.3	237.2	21.1 ± 6.5	17.7
80	38.9 ± 0.3	–	113.4 ± 5.5	–	230.4 ± 7.5	–	21.9 ± 1.9	20.4
100	42.7 ± 0.5	45.5	368.7 ± 31.6	311.3	236.5 ± 13.6	223.4	28.1 ± 2.8	31.7
120	41.1 ± 0.4	42.3	196.5 ± 12.0	179.6	314.0 ± 19.1	299.9	46.1 ± 1.9	46.7
140	39.7 ± 0.2	40.7	152.6 ± 9.8	134.5	374.2 ± 22.8	361.9	55.8 ± 4.4	54.9
160	39.4 ± 0.2	40.0	129.7 ± 4.4	116.2	425.1 ± 16.5	409.1	64.7 ± 6.9	60.7
180	39.1 ± 0.2	39.6	114.2 ± 2.5	106.1	469.3 ± 9.5	447.5	68.8 ± 6.2	65.4
200	38.9 ± 0.2	39.3	106.3 ± 3.4	99.5	505.9 ± 17.0	480.4	68.4 ± 3.3	69.5

S17.7 Data of thermodynamics and transport properties of CO_2 rich quaternary mixture with 1 mole% impurity of Ar, 1 mole% impurity of N_2, and 2 mole% impurity of CH_4

Table S411: Densities computed from MC and MD simulations (ρ^{MC} and ρ^{MD}), densities obtained from REFPROP[10] (ρ^{REFP}), isothermal compressibilities computed from MC simulations (β_T^{MC}), isothermal compressibilities obtained from REFPROP[10] (β_T^{REFP}), thermal expansion coefficients computed from MC simulations (α_P^{MC}), thermal expansion coefficients obtained from REFPROP[10] (α_P^{REFP}), Joule Thomson coefficients computed from MC simulations (μ_{JT}^{MC}), and Joule Thomson coefficients obtained from REFPROP[10] (μ_{JT}^{REFP}) of CO_2 rich quaternary mixture with 2% impurity of CH_4, 1% impurity of Ar and 1% impurity of N_2 at 253 K and pressures ranging from 20 bar to 200 bar.

P/ [bar]	ρ^{MC}/ [kg/m^3]	ρ^{MD}/ [kg/m^3]	ρ^{REFP}/ [kg/m^3]	β_T^{MC}/ [10^{-5}/bar]	β_T^{REFP}/ [10^{-5}/bar]	α_P^{MC}/ [10^{-4}/K]	α_P^{REFP}/ [10^{-4}/K]	μ_{JT}^{MC}/ [10^{-3} K/bar]	μ_{JT}^{REFP}/ [10^{-3} K/bar]
20	49.7	50.5 ± 0.1	51.1	6252.8 ± 49.7	6542.2	74.3 ± 0.8	82.5	1501.0 ± 34.2	1665.3
40	997.5 ± 1.1	995.3 ± 0.5	997.1	44.0 ± 1.3	50.8	48.2 ± 1.2	49.7	9.7 ± 1.4	12.0
60	1004.4 ± 0.9	1003.0 ± 1.1	1006.8	40.8 ± 3.4	45.8	45.4 ± 3.6	46.4	6.7 ± 4.1	8.2
80	1013.5 ± 1.4	1011.3 ± 0.7	1015.6	35.5 ± 1.3	41.8	41.0 ± 1.7	43.7	1.8 ± 2.0	5.0
100	1021.2 ± 1.8	1018.5 ± 0.7	1023.8	33.9 ± 0.8	38.5	40.0 ± 1.3	41.4	0.5 ± 1.6	2.3
120	1027.2 ± 0.9	1025.2 ± 0.5	1031.5	32.2 ± 1.5	35.8	39.1 ± 1.7	39.4	-0.6 ± 2.0	-0.1
140	1033.0 ± 1.0	1031.8 ± 0.9	1038.6	30.9 ± 0.7	33.5	38.2 ± 0.9	37.7	-1.5 ± 1.1	-2.2
160	1040.0 ± 0.8	1038.0 ± 0.8	1045.4	28.5 ± 1.0	31.5	36.0 ± 1.2	36.2	-4.2 ± 1.4	-4.1
180	1045.9 ± 0.8	1043.5 ± 0.6	1051.8	26.2 ± 0.5	29.7	33.6 ± 0.8	34.9	-7.2 ± 1.0	-5.8
200	1050.4 ± 1.3	1049.6 ± 0.7	1057.9	25.9 ± 1.2	28.2	33.7 ± 1.4	33.7	-7.0 ± 1.7	-7.3

Table S412: Heat capacities at constant volume computed from MC simulations (c_V^{MC}), heat capacities at constant volume obtained from REFPROP[10] (c_V^{REFP}), heat capacities at constant pressure computed from MC simulations (c_P^{MC}), heat capacities at constant pressure obtained from REFPROP[10] (c_P^{REFP}), speed of sound computed from MC simulations (c^{MC}), speed of sound obtained from REFPROP[10] (c^{REFP}), viscosities computed from MD simulations (η^{MD}), and viscosities obtained from REFPROP[10] (η^{REFP}) of CO_2 rich quaternary mixture with 2% impurity of CH_4, 1% impurity of Ar and 1% impurity of N_2 at 253 K and pressures ranging from 20 bar to 200 bar.

P/ [bar]	c_V^{MC}/ [J/mol K]	c_V^{REFP}/ [J/mol K]	c_P^{MC}/ [J/mol K]	c_P^{REFP}/ [J/mol K]	c^{MC}/ [m/s]	c^{REFP}/ [m/s]	η^{MD}/ [μPa s]	η^{REFP}/ [μPa s]
20	31.5 ± 0.1	33.0	51.0 ± 0.3	55.3	228.1 ± 1.1	223.8	12.1 ± 4.0	13.0
40	40.0 ± 0.1	40.3	98.1 ± 1.5	93.8	746.9 ± 12.5	677.9	137.3 ± 3.1	128.3
60	39.9 ± 0.1	40.2	95.1 ± 4.3	91.3	762.4 ± 36.5	701.6	147.8 ± 15.1	132.3
80	40.1 ± 0.2	40.2	91.2 ± 2.3	89.4	795.1 ± 18.2	723.2	144.9 ± 5.7	136.1
100	40.3 ± 0.1	40.2	90.6 ± 2.1	87.7	805.9 ± 13.6	743.3	158.2 ± 29.6	139.8
120	40.5 ± 0.3	40.2	90.6 ± 2.3	86.3	823.1 ± 22.2	762.0	146.2 ± 6.9	143.4
140	40.5 ± 0.1	40.3	90.5 ± 1.4	85.1	837.5 ± 11.3	779.5	155.3 ± 28.2	146.8
160	40.4 ± 0.2	40.3	88.2 ± 1.4	84.0	858.9 ± 17.2	796.2	172.0 ± 23.9	150.2
180	40.4 ± 0.2	40.3	85.5 ± 1.5	83.0	878.5 ± 11.6	811.9	172.4 ± 14.6	153.4
200	40.3 ± 0.4	40.4	86.2 ± 1.9	82.2	886.0 ± 22.7	827.0	168.2 ± 12.0	156.7

Table S413: Densities computed from MC and MD simulations (ρ^{MC} and ρ^{MD}), densities obtained from REFPROP[10] (ρ^{REFP}), isothermal compressibilities computed from MC simulations (β_T^{MC}), isothermal compressibilities obtained from REFPROP[10] (β_T^{REFP}), thermal expansion coefficients computed from MC simulations (α_P^{MC}), thermal expansion coefficients obtained from REFPROP[10] (α_P^{REFP}), Joule Thomson coefficients computed from MC simulations (μ_{JT}^{MC}), and Joule Thomson coefficients obtained from REFPROP[10] (μ_{JT}^{REFP}) of CO_2 rich quaternary mixture with 2% impurity of CH_4, 1% impurity of Ar and 1% impurity of N_2 at 273 K and pressures ranging from 20 bar to 200 bar.

P/ [bar]	ρ^{MC}/ [kg/m^3]	ρ^{MD}/ [kg/m^3]	ρ^{REFP}/ [kg/m^3]	β_T^{MC}/ [10^{-5}/bar]	β_T^{REFP}/ [10^{-5}/bar]	α_P^{MC}/ [10^{-4}/K]	α_P^{REFP}/ [10^{-4}/K]	μ_{JT}^{MC}/ [10^{-3} K/bar]	μ_{JT}^{REFP}/ [10^{-3} K/bar]
20	43.6	44.3	44.4	5813.4 ± 50.2	5951.3	57.3 ± 0.5	60.6	1217.5 ± 29.5	1341.0
40	109.7 ± 0.1	882.3 ± 1.2	276.6	4373.5 ± 98.0	-2038.3	134.8 ± 2.5	-413.0	1296.6 ± 34.9	–
60	901.1 ± 2.6	899.4 ± 1.4	896.8	86.4 ± 5.4	108.4	67.4 ± 3.0	75.0	37.0 ± 3.8	46.4
80	917.4 ± 1.2	913.5 ± 1.0	914.4	69.9 ± 3.6	87.6	58.0 ± 3.0	64.8	27.0 ± 3.9	35.6
100	929.3 ± 0.9	927.2 ± 1.3	929.2	62.6 ± 3.9	74.1	53.9 ± 2.8	57.8	22.3 ± 3.7	27.7
120	941.0 ± 1.3	938.4 ± 1.7	942.2	57.5 ± 1.7	64.6	52.0 ± 1.7	52.7	19.6 ± 2.2	21.4
140	950.8 ± 1.3	949.0 ± 0.7	953.7	50.1 ± 4.2	57.4	46.9 ± 3.7	48.7	13.5 ± 4.9	16.4
160	959.9 ± 1.3	958.3 ± 0.6	964.1	46.0 ± 2.2	51.7	44.6 ± 2.4	45.5	10.5 ± 3.2	12.2
180	968.0 ± 0.8	967.0 ± 0.6	973.7	42.7 ± 2.5	47.2	42.2 ± 2.6	42.9	7.5 ± 3.5	8.7
200	976.2 ± 1.0	975.0 ± 1.0	982.6	39.6 ± 0.8	43.4	40.3 ± 1.0	40.6	5.0 ± 1.3	5.6

Table S414: Heat capacities at constant volume computed from MC simulations (c_V^{MC}), heat capacities at constant volume obtained from REFPROP[10] (c_V^{REFP}), heat capacities at constant pressure computed from MC simulations (c_P^{MC}), heat capacities at constant pressure obtained from REFPROP[10] (c_P^{REFP}), speed of sound computed from MC simulations (c^{MC}), speed of sound obtained from REFPROP[10] (c^{REFP}), viscosities computed from MD simulations (η^{MD}), and viscosities obtained from REFPROP[10] (η^{REFP}) of CO_2 rich quaternary mixture with 2% impurity of CH_4, 1% impurity of Ar and 1% impurity of N_2 at 273 K and pressures ranging from 20 bar to 200 bar.

P/ [bar]	c_V^{MC}/ [J/mol K]	c_V^{REFP}/ [J/mol K]	c_P^{MC}/ [J/mol K]	c_P^{REFP}/ [J/mol K]	c^{MC}/ [m/s]	c^{REFP}/ [m/s]	η^{MD}/ [μPa s]	η^{REFP}/ [μPa s]
20	30.7	31.1	46.0 ± 0.1	47.4	243.1 ± 1.1	240.3	10.9 ± 6.2	14.0
40	36.8 ± 0.2	–	81.5 ± 0.7	–	214.8 ± 2.6	–	96.6 ± 3.1	19.9
60	39.5 ± 0.1	40.5	108.9 ± 2.9	108.8	594.9 ± 20.3	525.7	101.4 ± 4.1	93.9
80	39.5 ± 0.2	40.2	101.7 ± 3.4	102.0	633.8 ± 19.7	562.7	104.1 ± 3.9	98.7
100	39.3 ± 0.2	40.0	98.7 ± 2.7	97.3	656.8 ± 22.6	594.0	111.5 ± 4.5	103.0
120	39.5 ± 0.1	39.9	98.6 ± 2.2	93.8	679.1 ± 12.8	621.6	108.7 ± 4.2	106.9
140	39.6 ± 0.1	39.9	94.3 ± 4.3	91.1	707.0 ± 33.6	646.3	123.3 ± 15.8	110.6
160	39.5 ± 0.2	39.8	92.9 ± 3.3	88.9	729.7 ± 22.2	668.9	116.0 ± 8.3	114.1
180	39.6 ± 0.1	39.8	90.6 ± 3.5	87.0	744.1 ± 26.2	689.8	122.3 ± 5.1	117.4
200	39.6 ± 0.2	39.8	89.4 ± 1.7	85.5	763.5 ± 10.5	709.3	122.4 ± 4.5	120.6

Table S415: Densities computed from MC and MD simulations (ρ^{MC} and ρ^{MD}), densities obtained from REFPROP[10] (ρ^{REFP}), isothermal compressibilities computed from MC simulations (β_T^{MC}), isothermal compressibilities obtained from REFPROP[10] (β_T^{REFP}), thermal expansion coefficients computed from MC simulations (α_P^{MC}), thermal expansion coefficients obtained from REFPROP[10] (α_P^{REFP}), Joule Thomson coefficients computed from MC simulations ($\mu_{\mathrm{JT}}^{\mathrm{MC}}$), and Joule Thomson coefficients obtained from REFPROP[10] ($\mu_{\mathrm{JT}}^{\mathrm{REFP}}$) of CO_2 rich quaternary mixture with 2% impurity of CH_4, 1% impurity of Ar and 1% impurity of N_2 at 293 K and pressures ranging from 20 bar to 200 bar.

P/ [bar]	ρ^{MC}/ [kg/m^3]	ρ^{MD}/ [kg/m^3]	ρ^{REFP}/ [kg/m^3]	β_T^{MC}/ [10^{-5}/bar]	β_T^{REFP}/ [10^{-5}/bar]	α_P^{MC}/ [10^{-4}/K]	α_P^{REFP}/ [10^{-4}/K]	$\mu_{\mathrm{JT}}^{\mathrm{MC}}$/ [$10^{-3}$ K/bar]	$\mu_{\mathrm{JT}}^{\mathrm{REFP}}$/ [$10^{-3}$ K/bar]
20	39.3	39.8	39.8	5607.0 ± 31.8	5665.4	48.1 ± 0.3	49.7	1023.1 ± 20.0	1109.5
40	90.7 ± 0.1	92.3 ± 0.5	93.8	3375.9 ± 52.3	3523.7	77.3 ± 1.4	85.0	1034.6 ± 34.6	1104.4
60	177.4 ± 0.5	320.8 ± 141.9	194.1	3933.1 ± 161.5	4625.5	218.8 ± 11.3	290.3	1034.9 ± 82.0	1024.5
80	766.0 ± 3.6	766.5 ± 3.4	752.5	282.3 ± 19.1	395.8	137.4 ± 8.7	165.5	112.3 ± 10.9	139.0
100	804.1 ± 2.6	802.1 ± 1.0	796.8	168.0 ± 11.0	216.0	94.8 ± 5.4	107.1	76.6 ± 7.4	92.5
120	829.2 ± 1.3	826.8 ± 0.9	826.1	127.7 ± 5.6	152.3	79.3 ± 2.9	84.1	59.9 ± 4.1	68.7
140	848.8 ± 1.7	846.1 ± 1.1	848.5	102.4 ± 9.5	118.9	67.6 ± 5.2	71.1	46.9 ± 7.5	53.3
160	864.2 ± 1.5	863.0 ± 1.5	867.0	88.3 ± 2.8	98.0	61.7 ± 1.2	62.5	39.3 ± 1.8	42.3
180	877.6 ± 1.7	877.2 ± 0.6	882.8	74.2 ± 2.7	83.7	54.1 ± 2.0	56.3	29.9 ± 3.2	33.9
200	892.9 ± 0.4	889.9 ± 0.9	896.7	65.0 ± 1.6	73.1	50.0 ± 0.9	51.6	24.1 ± 1.4	27.2

Table S416: Heat capacities at constant volume computed from MC simulations (c_V^{MC}), heat capacities at constant volume obtained from REFPROP[10] (c_V^{REFP}), heat capacities at constant pressure computed from MC simulations (c_P^{MC}), heat capacities at constant pressure obtained from REFPROP[10] (c_P^{REFP}), speed of sound computed from MC simulations (c^{MC}), speed of sound obtained from REFPROP[10] (c^{REFP}), viscosities computed from MD simulations (η^{MD}), and viscosities obtained from REFPROP[10] (η^{REFP}) of CO_2 rich quaternary mixture with 2% impurity of CH_4, 1% impurity of Ar and 1% impurity of N_2 at 293 K and pressures ranging from 20 bar to 200 bar.

P/ [bar]	c_V^{MC}/ [J/mol K]	c_V^{REFP}/ [J/mol K]	c_P^{MC}/ [J/mol K]	c_P^{REFP}/ [J/mol K]	c^{MC}/ [m/s]	c^{REFP}/ [m/s]	η^{MD}/ [μPa s]	η^{REFP}/ [μPa s]
20	30.6	30.8	43.9 ± 0.1	44.7	255.2 ± 0.8	253.5	8.2 ± 4.4	15.0
40	33.6 ± 0.1	34.5	58.3 ± 0.5	62.2	238.0 ± 2.1	233.5	15.4 ± 1.2	15.7
60	39.8 ± 0.5	44.3	127.5 ± 6.4	163.2	214.2 ± 7.1	202.6	21.1 ± 7.0	18.0
80	39.8 ± 0.3	42.6	152.2 ± 7.2	159.2	420.4 ± 17.5	354.3	71.2 ± 4.6	64.3
100	39.5 ± 0.1	41.0	124.9 ± 4.7	125.4	483.5 ± 18.3	421.5	76.5 ± 3.8	71.9
120	39.1 ± 0.2	40.4	115.1 ± 2.9	111.6	527.0 ± 13.4	468.6	81.5 ± 6.6	77.5
140	39.2 ± 0.1	40.0	106.4 ± 4.5	103.5	558.8 ± 28.4	506.4	84.3 ± 3.7	82.2
160	39.2 ± 0.1	39.8	103.0 ± 1.3	98.1	587.2 ± 10.0	538.5	93.1 ± 6.6	86.3
180	39.2 ± 0.1	39.7	96.4 ± 2.5	94.1	614.7 ± 13.7	566.8	94.7 ± 4.0	90.1
200	39.2 ± 0.1	39.6	93.9 ± 0.8	91.1	642.4 ± 8.4	592.3	93.8 ± 2.9	93.5

Table S417: Densities computed from MC and MD simulations (ρ^{MC} and ρ^{MD}), densities obtained from REFPROP[10] (ρ^{REFP}), isothermal compressibilities computed from MC simulations (β_T^{MC}), isothermal compressibilities obtained from REFPROP[10] (β_T^{REFP}), thermal expansion coefficients computed from MC simulations (α_P^{MC}), thermal expansion coefficients obtained from REFPROP[10] (α_P^{REFP}), Joule Thomson coefficients computed from MC simulations (μ_{JT}^{MC}), and Joule Thomson coefficients obtained from REFPROP[10] (μ_{JT}^{REFP}) of CO_2 rich quaternary mixture with 2% impurity of CH_4, 1% impurity of Ar and 1% impurity of N_2 at 313 K and pressures ranging from 20 bar to 200 bar.

P/ [bar]	ρ^{MC}/ [kg/m^3]	ρ^{MD}/ [kg/m^3]	ρ^{REFP}/ [kg/m^3]	β_T^{MC}/ [10^{-5}/bar]	β_T^{REFP}/ [10^{-5}/bar]	α_P^{MC}/ [10^{-4}/K]	α_P^{REFP}/ [10^{-4}/K]	μ_{JT}^{MC}/ [10^{-3} K/bar]	μ_{JT}^{REFP}/ [10^{-3} K/bar]
20	35.9	36.3	36.3	5435.7 ± 21.8	5493.3	41.7 ± 0.2	42.9	853.2 ± 15.5	934.6
40	79.4 ± 0.1	80.8 ± 0.1	81.3	3024.5 ± 17.4	3139.9	57.3 ± 0.3	61.6	842.9 ± 9.5	924.5
60	136.5 ± 0.1	139.2 ± 0.7	142.0	2486.6 ± 12.8	2590.7	92.1 ± 0.6	101.0	862.7 ± 10.5	890.6
80	225.9 ± 0.4	235.3 ± 2.3	243.0	2743.5 ± 121.0	2955.8	199.0 ± 10.4	230.1	792.0 ± 59.2	–
100	470.8 ± 10.3	474.5 ± 8.0	482.4	3275.8 ± 329.5	2817.3	616.2 ± 40.1	524.1	436.6 ± 36.7	431.3
120	643.3 ± 2.2	641.2 ± 3.2	637.8	628.4 ± 19.3	689.0	206.5 ± 7.6	206.5	198.2 ± 10.7	212.9
140	706.7 ± 4.2	700.5 ± 1.8	702.1	289.7 ± 24.4	344.6	119.6 ± 8.7	128.0	124.5 ± 13.6	139.5
160	740.0 ± 3.1	739.8 ± 1.5	742.2	208.8 ± 10.6	227.8	96.4 ± 4.5	97.0	97.1 ± 7.5	102.6
180	770.2 ± 1.5	767.1 ± 0.8	771.9	144.2 ± 4.4	170.1	74.8 ± 2.3	80.1	70.1 ± 4.0	79.6
200	791.7 ± 1.3	789.6 ± 2.4	795.7	128.1 ± 1.7	135.8	70.7 ± 1.0	69.2	62.4 ± 1.7	63.6

Table S418: Heat capacities at constant volume computed from MC simulations (c_V^{MC}), heat capacities at constant volume obtained from REFPROP[10] (c_V^{REFP}), heat capacities at constant pressure computed from MC simulations (c_P^{MC}), heat capacities at constant pressure obtained from REFPROP[10] (c_P^{REFP}), speed of sound computed from MC simulations (c^{MC}), speed of sound obtained from REFPROP[10] (c^{REFP}), viscosities computed from MD simulations (η^{MD}), and viscosities obtained from REFPROP[10] (η^{REFP}) of CO_2 rich quaternary mixture with 2% impurity of CH_4, 1% impurity of Ar and 1% impurity of N_2 at 313 K and pressures ranging from 20 bar to 200 bar.

P/ [bar]	c_V^{MC}/ [J/mol K]	c_V^{REFP}/ [J/mol K]	c_P^{MC}/ [J/mol K]	c_P^{REFP}/ [J/mol K]	c^{MC}/ [m/s]	c^{REFP}/ [m/s]	η^{MD}/ [μPa s]	η^{REFP}/ [μPa s]
20	31.0	31.0	43.0 ± 0.1	43.5	266.8 ± 0.6	265.0	2.2 ± 2.6	16.0
40	32.8	33.3	51.3 ± 0.1	53.4	255.3 ± 0.8	250.8	16.3 ± 1.0	16.6
60	35.3 ± 0.1	36.4	69.2 ± 0.4	73.9	240.4 ± 1.0	235.0	17.9 ± 2.7	17.7
80	39.1 ± 0.2	–	126.4 ± 5.3	–	228.3 ± 7.0	–	20.2 ± 0.7	20.7
100	42.4 ± 0.6	46.6	385.2 ± 16.7	320.2	242.9 ± 13.7	224.8	31.1 ± 1.0	34.3
120	40.3 ± 0.3	42.7	185.4 ± 6.0	174.0	337.2 ± 7.6	304.6	50.3 ± 5.5	49.2
140	39.3 ± 0.1	41.1	134.9 ± 6.2	132.8	409.7 ± 19.7	365.5	61.6 ± 3.8	57.4
160	39.2 ± 0.4	40.3	121.4 ± 3.9	115.7	447.8 ± 13.6	411.8	61.7 ± 2.7	63.2
180	39.0 ± 0.2	39.9	107.6 ± 2.1	106.0	498.2 ± 9.1	449.7	72.9 ± 2.4	68.0
200	39.0 ± 0.1	39.7	106.0 ± 1.0	99.7	517.9 ± 4.3	482.2	78.5 ± 7.7	72.1

S17.8 Data of thermodynamics and transport properties of CO_2 rich quaternary mixture with 1 mole% impurity of Ar, 2 mole% impurity of N_2, and 1 mole% impurity of CH_4

Table S419: Densities computed from MC and MD simulations (ρ^{MC} and ρ^{MD}), densities obtained from REFPROP[10] (ρ^{REFP}), isothermal compressibilities computed from MC simulations (β_T^{MC}), isothermal compressibilities obtained from REFPROP[10] (β_T^{REFP}), thermal expansion coefficients computed from MC simulations (α_P^{MC}), thermal expansion coefficients obtained from REFPROP[10] (α_P^{REFP}), Joule Thomson coefficients computed from MC simulations ($\mu_{\mathrm{JT}}^{\mathrm{MC}}$), and Joule Thomson coefficients obtained from REFPROP[10] ($\mu_{\mathrm{JT}}^{\mathrm{REFP}}$) of CO_2 rich quaternary mixture with 2% impurity of N_2, 1% impurity of Ar and 1% impurity of CH_4 at 253 K and pressures ranging from 20 bar to 200 bar.

P/ [bar]	ρ^{MC}/ [kg/m^3]	ρ^{MD}/ [kg/m^3]	ρ^{REFP}/ [kg/m^3]	β_T^{MC}/ [10^{-5}/bar]	β_T^{REFP}/ [10^{-5}/bar]	α_P^{MC}/ [10^{-4}/K]	α_P^{REFP}/ [10^{-4}/K]	$\mu_{\mathrm{JT}}^{\mathrm{MC}}$/ [$10^{-3}$ K/bar]	$\mu_{\mathrm{JT}}^{\mathrm{REFP}}$/ [$10^{-3}$ K/bar]
20	49.7	50.6 ± 0.1	51.2	6342.5 ± 62.6	6528.2	75.0 ± 1.0	82.2	1533.7 ± 42.3	1660.4
40	997.3 ± 0.4	996.1 ± 0.4	999.2	43.1 ± 2.5	51.9	46.5 ± 2.5	50.4	8.1 ± 2.9	12.7
60	1007.4 ± 2.3	1004.1 ± 0.9	1009.1	38.6 ± 1.5	46.6	43.0 ± 1.4	46.9	4.1 ± 1.6	8.8
80	1013.8 ± 1.8	1012.0 ± 1.5	1018.1	37.9 ± 2.1	42.5	43.3 ± 2.2	44.1	4.4 ± 2.6	5.5
100	1022.4 ± 0.5	1020.1 ± 0.4	1026.4	34.1 ± 1.7	39.1	40.1 ± 2.1	41.7	0.7 ± 2.5	2.7
120	1028.0 ± 1.4	1026.9 ± 0.3	1034.2	33.7 ± 0.4	36.3	40.5 ± 0.6	39.7	1.1 ± 0.7	0.3
140	1034.7 ± 1.0	1033.4 ± 0.8	1041.5	29.7 ± 0.5	33.9	36.3 ± 0.7	38.0	-3.9 ± 0.9	-1.9
160	1040.2 ± 1.1	1039.6 ± 0.5	1048.3	28.8 ± 0.9	31.8	35.8 ± 1.2	36.5	-4.5 ± 1.4	-3.8
180	1047.1 ± 1.2	1045.8 ± 0.4	1054.8	27.4 ± 0.9	30.0	35.0 ± 1.0	35.1	-5.4 ± 1.2	-5.5
200	1052.1 ± 0.4	1051.2 ± 0.6	1061.0	25.9 ± 0.4	28.5	33.6 ± 0.5	33.9	-7.2 ± 0.6	-7.0

Table S420: Heat capacities at constant volume computed from MC simulations (c_V^{MC}), heat capacities at constant volume obtained from REFPROP[10] (c_V^{REFP}), heat capacities at constant pressure computed from MC simulations (c_P^{MC}), heat capacities at constant pressure obtained from REFPROP[10] (c_P^{REFP}), speed of sound computed from MC simulations (c^{MC}), speed of sound obtained from REFPROP[10] (c^{REFP}), viscosities computed from MD simulations (η^{MD}), and viscosities obtained from REFPROP[10] (η^{REFP}) of CO_2 rich quaternary mixture with 2% impurity of N_2, 1% impurity of Ar and 1% impurity of CH_4 at 253 K and pressures ranging from 20 bar to 200 bar.

P/ [bar]	c_V^{MC}/ [J/mol K]	c_V^{REFP}/ [J/mol K]	c_P^{MC}/ [J/mol K]	c_P^{REFP}/ [J/mol K]	c^{MC}/ [m/s]	c^{REFP}/ [m/s]	η^{MD}/ [μPa s]	η^{REFP}/ [μPa s]
20	31.5	32.9	51.1 ± 0.3	55.0	226.8 ± 1.3	223.8	10.4 ± 3.7	13.1
40	40.2 ± 0.3	40.4	95.4 ± 2.9	94.1	743.7 ± 24.6	670.8	134.1 ± 1.8	128.0
60	40.0 ± 0.1	40.3	92.2 ± 1.7	91.6	769.5 ± 16.8	694.8	136.7 ± 3.5	132.1
80	40.1 ± 0.1	40.3	93.7 ± 2.9	89.6	780.1 ± 24.5	716.7	140.0 ± 2.9	136.0
100	40.3 ± 0.2	40.3	90.8 ± 2.8	87.9	804.1 ± 23.3	736.9	156.5 ± 11.2	139.7
120	40.2 ± 0.1	40.3	92.3 ± 1.4	86.4	814.2 ± 7.6	755.8	152.0 ± 10.8	143.3
140	40.1 ± 0.3	40.3	87.2 ± 1.5	85.2	840.7 ± 10.8	773.5	141.9 ± 27.4	146.7
160	40.4 ± 0.2	40.3	87.3 ± 1.7	84.1	849.6 ± 15.8	790.3	152.6 ± 5.8	150.1
180	40.2 ± 0.2	40.4	87.2 ± 1.2	83.1	869.5 ± 15.2	806.2	177.1 ± 21.4	153.4
200	40.3 ± 0.2	40.4	85.8 ± 0.9	82.2	883.6 ± 7.9	821.3	159.7 ± 4.1	156.6

Table S421: Densities computed from MC and MD simulations (ρ^{MC} and ρ^{MD}), densities obtained from REFPROP[10] (ρ^{REFP}), isothermal compressibilities computed from MC simulations (β_T^{MC}), isothermal compressibilities obtained from REFPROP[10] (β_T^{REFP}), thermal expansion coefficients computed from MC simulations (α_P^{MC}), thermal expansion coefficients obtained from REFPROP[10] (α_P^{REFP}), Joule Thomson coefficients computed from MC simulations (μ_{JT}^{MC}), and Joule Thomson coefficients obtained from REFPROP[10] (μ_{JT}^{REFP}) of CO_2 rich quaternary mixture with 2% impurity of N_2, 1% impurity of Ar and 1% impurity of CH_4 at 273 K and pressures ranging from 20 bar to 200 bar.

P/ [bar]	ρ^{MC}/ [kg/m^3]	ρ^{MD}/ [kg/m^3]	ρ^{REFP}/ [kg/m^3]	β_T^{MC}/ [10^{-5}/bar]	β_T^{REFP}/ [10^{-5}/bar]	α_P^{MC}/ [10^{-4}/K]	α_P^{REFP}/ [10^{-4}/K]	μ_{JT}^{MC}/ [10^{-3} K/bar]	μ_{JT}^{REFP}/ [10^{-3} K/bar]
20	43.7	44.4 ± 0.1	44.5	5824.7 ± 45.4	5945.4	57.2 ± 0.4	60.5	1216.4 ± 24.7	1338.5
40	110.2 ± 0.2	880.3 ± 1.1	259.7	4415.0 ± 111.0	−2809.1	136.6 ± 3.2	−461.4	1303.6 ± 44.9	–
60	903.8 ± 2.5	899.6 ± 1.6	897.1	86.7 ± 4.2	112.4	68.4 ± 3.0	76.8	37.7 ± 3.7	48.3
80	916.3 ± 2.3	914.0 ± 1.1	915.3	71.9 ± 3.4	90.2	58.9 ± 2.4	65.9	28.2 ± 3.1	36.9
100	929.2 ± 0.8	927.7 ± 1.2	930.5	70.0 ± 3.8	75.9	59.5 ± 2.7	58.7	27.9 ± 3.3	28.7
120	940.7 ± 1.8	939.0 ± 0.5	943.8	58.7 ± 3.7	65.9	51.8 ± 3.0	53.3	19.6 ± 4.0	22.3
140	950.9 ± 1.3	948.1 ± 0.9	955.6	52.1 ± 1.2	58.4	48.1 ± 0.8	49.2	15.1 ± 1.1	17.1
160	960.1 ± 0.6	958.7 ± 0.9	966.2	48.2 ± 2.1	52.6	45.8 ± 1.8	46.0	12.1 ± 2.4	12.8
180	969.0 ± 1.1	967.9 ± 1.6	975.9	43.8 ± 0.9	47.9	43.3 ± 1.0	43.3	8.9 ± 1.3	9.2
200	976.8 ± 1.0	975.5 ± 1.0	985.0	40.6 ± 1.5	44.1	41.1 ± 1.4	41.0	6.0 ± 1.9	6.1

Table S422: Heat capacities at constant volume computed from MC simulations (c_V^{MC}), heat capacities at constant volume obtained from REFPROP[10] (c_V^{REFP}), heat capacities at constant pressure computed from MC simulations (c_P^{MC}), heat capacities at constant pressure obtained from REFPROP[10] (c_P^{REFP}), speed of sound computed from MC simulations (c^{MC}), speed of sound obtained from REFPROP[10] (c^{REFP}), viscosities computed from MD simulations (η^{MD}), and viscosities obtained from REFPROP[10] (η^{REFP}) of CO_2 rich quaternary mixture with 2% impurity of N_2, 1% impurity of Ar and 1% impurity of CH_4 at 273 K and pressures ranging from 20 bar to 200 bar.

P/ [bar]	c_V^{MC}/ [J/mol K]	c_V^{REFP}/ [J/mol K]	c_P^{MC}/ [J/mol K]	c_P^{REFP}/ [J/mol K]	c^{MC}/ [m/s]	c^{REFP}/ [m/s]	η^{MD}/ [μPa s]	η^{REFP}/ [μPa s]
20	30.6	31.0	45.8 ± 0.1	47.3	242.6 ± 1.0	240.2	11.3 ± 4.5	14.1
40	36.6 ± 0.2	–	82.4 ± 1.0	–	215.3 ± 3.1	–	94.9 ± 3.6	19.1
60	39.5 ± 0.2	40.6	110.5 ± 3.0	109.8	597.3 ± 16.6	518.0	98.3 ± 2.9	93.4
80	39.5 ± 0.2	40.3	102.3 ± 2.1	102.6	626.8 ± 16.4	555.8	109.0 ± 6.0	98.3
100	39.4 ± 0.2	40.1	104.6 ± 2.5	97.7	639.4 ± 19.0	587.6	107.9 ± 7.0	102.7
120	39.3 ± 0.2	40.0	97.2 ± 3.4	94.1	669.6 ± 24.3	615.5	125.9 ± 25.9	106.7
140	39.4 ± 0.2	39.9	95.0 ± 0.9	91.3	697.2 ± 8.6	640.5	114.7 ± 2.1	110.4
160	39.5 ± 0.3	39.8	93.3 ± 2.0	89.1	714.8 ± 17.9	663.3	120.2 ± 5.5	113.9
180	39.6 ± 0.2	39.8	91.9 ± 1.4	87.2	739.3 ± 9.8	684.4	126.5 ± 16.3	117.3
200	39.6 ± 0.2	39.8	90.1 ± 1.7	85.6	757.4 ± 15.9	704.0	126.1 ± 5.6	120.5

Table S423: Densities computed from MC and MD simulations (ρ^{MC} and ρ^{MD}), densities obtained from REFPROP[10] (ρ^{REFP}), isothermal compressibilities computed from MC simulations (β_T^{MC}), isothermal compressibilities obtained from REFPROP[10] (β_T^{REFP}), thermal expansion coefficients computed from MC simulations (α_P^{MC}), thermal expansion coefficients obtained from REFPROP[10] (α_P^{REFP}), Joule Thomson coefficients computed from MC simulations (μ_{JT}^{MC}), and Joule Thomson coefficients obtained from REFPROP[10] (μ_{JT}^{REFP}) of CO_2 rich quaternary mixture with 2% impurity of N_2, 1% impurity of Ar and 1% impurity of CH_4 at 293 K and pressures ranging from 20 bar to 200 bar.

P/ [bar]	ρ^{MC}/ [kg/m^3]	ρ^{MD}/ [kg/m^3]	ρ^{REFP}/ [kg/m^3]	β_T^{MC}/ [10^{-5}/bar]	β_T^{REFP}/ [10^{-5}/bar]	α_P^{MC}/ [10^{-4}/K]	α_P^{REFP}/ [10^{-4}/K]	μ_{JT}^{MC}/ [10^{-3} K/bar]	μ_{JT}^{REFP}/ [10^{-3} K/bar]
20	39.4	40.1	39.9	5588.2 ± 26.3	5661.8	47.8 ± 0.3	49.7	1009.5 ± 19.4	1108.1
40	90.8 ± 0.1	92.5 ± 0.2	93.9	3388.5 ± 33.3	3513.1	77.3 ± 0.6	84.5	1038.0 ± 14.6	1100.9
60	176.7 ± 0.9	205.9 ± 21.0	193.0	3802.8 ± 181.6	4496.6	207.4 ± 11.6	278.3	1027.4 ± 82.2	1022.7
80	763.5 ± 4.5	756.5 ± 4.2	747.9	291.2 ± 35.9	433.8	139.4 ± 15.0	176.5	114.7 ± 18.2	146.7
100	799.2 ± 4.9	797.4 ± 3.1	795.2	183.6 ± 13.2	226.9	100.7 ± 5.7	110.6	82.4 ± 7.4	96.0
120	827.5 ± 1.8	826.7 ± 1.6	825.6	138.2 ± 6.0	157.8	83.4 ± 3.1	86.0	64.5 ± 4.3	70.9
140	846.1 ± 1.5	846.1 ± 1.7	848.8	111.5 ± 2.9	122.3	71.6 ± 1.7	72.3	51.7 ± 2.4	54.9
160	863.8 ± 1.9	861.5 ± 1.3	867.8	88.5 ± 3.8	100.4	61.3 ± 1.7	63.4	39.3 ± 2.5	43.5
180	877.6 ± 0.8	877.6 ± 0.4	883.9	81.5 ± 5.0	85.4	59.3 ± 2.9	57.0	35.8 ± 4.2	34.9
200	891.6 ± 1.3	889.4 ± 0.9	898.1	67.5 ± 2.7	74.5	51.4 ± 2.1	52.2	25.9 ± 3.1	28.0

Table S424: Heat capacities at constant volume computed from MC simulations (c_V^{MC}), heat capacities at constant volume obtained from REFPROP[10] (c_V^{REFP}), heat capacities at constant pressure computed from MC simulations (c_P^{MC}), heat capacities at constant pressure obtained from REFPROP[10] (c_P^{REFP}), speed of sound computed from MC simulations (c^{MC}), speed of sound obtained from REFPROP[10] (c^{REFP}), viscosities computed from MD simulations (η^{MD}), and viscosities obtained from REFPROP[10] (η^{REFP}) of CO_2 rich quaternary mixture with 2% impurity of N_2, 1% impurity of Ar and 1% impurity of CH_4 at 293 K and pressures ranging from 20 bar to 200 bar.

P/ [bar]	c_V^{MC}/ [J/mol K]	c_V^{REFP}/ [J/mol K]	c_P^{MC}/ [J/mol K]	c_P^{REFP}/ [J/mol K]	c^{MC}/ [m/s]	c^{REFP}/ [m/s]	η^{MD}/ [μPa s]	η^{REFP}/ [μPa s]
20	30.6	30.7	43.8 ± 0.1	44.6	255.2 ± 0.7	253.3	11.3 ± 4.6	15.1
40	33.6	34.4	58.2 ± 0.2	61.9	237.3 ± 1.2	233.6	16.4 ± 2.3	15.8
60	39.6 ± 0.6	43.8	121.3 ± 5.3	157.2	213.5 ± 7.1	203.4	18.0 ± 1.1	18.0
80	40.0 ± 0.3	42.8	152.8 ± 10.9	164.8	414.3 ± 29.6	344.3	68.2 ± 2.7	63.3
100	39.6 ± 0.2	41.1	128.5 ± 3.9	127.2	470.1 ± 18.4	414.0	81.8 ± 6.0	71.3
120	39.3 ± 0.2	40.4	117.4 ± 2.8	112.5	510.8 ± 12.7	462.2	85.8 ± 11.9	77.1
140	39.2 ± 0.2	40.1	108.9 ± 1.6	104.1	542.8 ± 8.3	500.5	91.2 ± 7.2	81.8
160	39.1 ± 0.1	39.8	102.0 ± 1.4	98.5	584.2 ± 13.1	533.0	92.0 ± 5.9	86.0
180	39.1 ± 0.1	39.7	102.0 ± 2.6	94.5	603.9 ± 20.2	561.6	95.8 ± 10.2	89.8
200	39.1 ± 0.1	39.6	95.1 ± 2.3	91.3	635.7 ± 15.0	587.2	96.7 ± 2.6	93.4

Table S425: Densities computed from MC and MD simulations (ρ^{MC} and ρ^{MD}), densities obtained from REFPROP[10] (ρ^{REFP}), isothermal compressibilities computed from MC simulations (β_T^{MC}), isothermal compressibilities obtained from REFPROP[10] (β_T^{REFP}), thermal expansion coefficients computed from MC simulations (α_P^{MC}), thermal expansion coefficients obtained from REFPROP[10] (α_P^{REFP}), Joule Thomson coefficients computed from MC simulations ($\mu_{\mathrm{JT}}^{\mathrm{MC}}$), and Joule Thomson coefficients obtained from REFPROP[10] ($\mu_{\mathrm{JT}}^{\mathrm{REFP}}$) of CO_2 rich quaternary mixture with 2% impurity of N_2, 1% impurity of Ar and 1% impurity of CH_4 at 313 K and pressures ranging from 20 bar to 200 bar.

P/ [bar]	ρ^{MC}/ [kg/m^3]	ρ^{MD}/ [kg/m^3]	ρ^{REFP}/ [kg/m^3]	β_T^{MC}/ [10^{-5}/bar]	β_T^{REFP}/ [10^{-5}/bar]	α_P^{MC}/ [10^{-4}/K]	α_P^{REFP}/ [10^{-4}/K]	$\mu_{\mathrm{JT}}^{\mathrm{MC}}$/ [$10^{-3}$ K/bar]	$\mu_{\mathrm{JT}}^{\mathrm{REFP}}$/ [$10^{-3}$ K/bar]
20	36.0	36.5 ± 0.1	36.4	5444.5 ± 7.1	5490.7	41.7 ± 0.1	42.8	859.2 ± 7.8	933.9
40	79.5 ± 0.1	80.8 ± 0.1	81.4	3050.3 ± 33.0	3134.7	57.7 ± 0.6	61.4	856.6 ± 20.3	922.1
60	136.4 ± 0.2	139.3 ± 0.7	142.1	2433.5 ± 50.4	2578.6	89.6 ± 2.0	100.1	842.4 ± 30.5	887.7
80	225.4 ± 0.8	234.0 ± 1.5	241.8	2743.3 ± 36.0	2903.1	195.5 ± 3.6	223.6	796.9 ± 21.7	–
100	450.1 ± 3.8	463.7 ± 4.8	471.3	3264.7 ± 148.1	2863.4	583.6 ± 29.2	513.9	455.6 ± 34.6	444.6
120	638.4 ± 6.6	632.6 ± 7.2	632.2	712.7 ± 127.2	730.5	227.0 ± 34.0	213.6	210.6 ± 43.2	220.4
140	700.5 ± 0.7	697.6 ± 2.2	699.4	324.8 ± 18.8	359.3	129.9 ± 7.2	131.2	134.1 ± 10.9	143.6
160	738.8 ± 3.9	736.7 ± 2.2	740.9	225.9 ± 31.1	235.2	101.9 ± 11.0	98.8	103.1 ± 17.5	105.2
180	769.4 ± 1.1	764.0 ± 1.0	771.5	158.3 ± 5.6	174.6	80.4 ± 2.1	81.3	76.7 ± 3.5	81.5
200	791.6 ± 2.2	789.6 ± 1.4	795.8	129.4 ± 9.4	138.8	70.9 ± 4.0	70.1	63.1 ± 6.7	65.0

Table S426: Heat capacities at constant volume computed from MC simulations (c_V^{MC}), heat capacities at constant volume obtained from REFPROP[10] (c_V^{REFP}), heat capacities at constant pressure computed from MC simulations (c_P^{MC}), heat capacities at constant pressure obtained from REFPROP[10] (c_P^{REFP}), speed of sound computed from MC simulations (c^{MC}), speed of sound obtained from REFPROP[10] (c^{REFP}), viscosities computed from MD simulations (η^{MD}), and viscosities obtained from REFPROP[10] (η^{REFP}) of CO_2 rich quaternary mixture with 2% impurity of N_2, 1% impurity of Ar and 1% impurity of CH_4 at 313 K and pressures ranging from 20 bar to 200 bar.

P/ [bar]	c_V^{MC}/ [J/mol K]	c_V^{REFP}/ [J/mol K]	c_P^{MC}/ [J/mol K]	c_P^{REFP}/ [J/mol K]	c^{MC}/ [m/s]	c^{REFP}/ [m/s]	η^{MD}/ [μPa s]	η^{REFP}/ [μPa s]
20	30.9	30.9	43.0	43.4	266.3 ± 0.2	264.8	3.6 ± 3.3	16.0
40	32.7	33.2	51.3 ± 0.2	53.2	254.3 ± 1.5	250.7	16.1 ± 0.9	16.6
60	35.2 ± 0.1	36.2	68.1 ± 0.8	73.4	241.2 ± 2.9	235.1	17.3 ± 0.4	17.8
80	38.9 ± 0.3	–	123.6 ± 2.0	–	226.8 ± 2.5	–	21.1 ± 1.6	20.7
100	42.4 ± 0.3	46.6	365.5 ± 19.6	312.2	242.4 ± 8.6	222.9	31.0 ± 2.1	33.3
120	40.2 ± 0.1	42.8	197.0 ± 21.1	177.0	328.2 ± 34.2	299.1	53.8 ± 7.7	48.5
140	39.4 ± 0.1	41.2	141.5 ± 5.1	134.2	397.3 ± 13.6	360.2	59.3 ± 3.3	56.8
160	39.2 ± 0.1	40.4	124.6 ± 7.7	116.4	436.2 ± 33.0	406.8	72.0 ± 10.9	62.8
180	39.0 ± 0.1	39.9	111.5 ± 1.4	106.5	484.8 ± 9.2	444.9	69.6 ± 6.9	67.7
200	38.9 ± 0.2	39.7	106.0 ± 3.0	100.0	515.4 ± 20.2	477.6	72.8 ± 3.4	71.8

S17.9 Data of thermodynamics and transport properties of CO_2 rich quaternary mixture with 2 mole% impurity of Ar, 1 mole% impurity of N_2, and 1 mole% impurity of CH_4

Table S427: Densities computed from MC and MD simulations (ρ^{MC} and ρ^{MD}), densities obtained from REFPROP[10] (ρ^{REFP}), isothermal compressibilities computed from MC simulations (β_T^{MC}), isothermal compressibilities obtained from REFPROP[10] (β_T^{REFP}), thermal expansion coefficients computed from MC simulations (α_P^{MC}), thermal expansion coefficients obtained from REFPROP[10] (α_P^{REFP}), Joule Thomson coefficients computed from MC simulations (μ_{JT}^{MC}), and Joule Thomson coefficients obtained from REFPROP[10] (μ_{JT}^{REFP}) of CO_2 rich quaternary mixture with 2% impurity of Ar, 1% impurity of N_2 and 1% impurity of CH_4 at 253 K and pressures ranging from 20 bar to 200 bar.

P/ [bar]	ρ^{MC}/ [kg/m^3]	ρ^{MD}/ [kg/m^3]	ρ^{REFP}/ [kg/m^3]	β_T^{MC}/ [10^{-5}/bar]	β_T^{REFP}/ [10^{-5}/bar]	α_P^{MC}/ [10^{-4}/K]	α_P^{REFP}/ [10^{-4}/K]	μ_{JT}^{MC}/ [10^{-3} K/bar]	μ_{JT}^{REFP}/ [10^{-3} K/bar]
20	49.9	50.7 ± 0.2	51.3	6348.8 ± 75.0	6519.3	75.3 ± 1.0	81.8	1542.2 ± 41.7	1656.9
40	1003.4 ± 1.0	1001.4 ± 1.1	1004.6	42.5 ± 2.6	50.8	46.2 ± 2.4	49.9	7.7 ± 2.8	12.1
60	1012.9 ± 0.5	1010.7 ± 0.7	1014.3	38.3 ± 1.1	45.8	43.0 ± 1.1	46.5	4.1 ± 1.3	8.3
80	1020.0 ± 0.7	1018.8 ± 0.9	1023.3	36.9 ± 1.7	41.8	42.3 ± 1.9	43.8	3.3 ± 2.2	5.1
100	1026.8 ± 0.4	1025.5 ± 0.3	1031.5	34.4 ± 1.2	38.6	40.4 ± 1.5	41.5	1.0 ± 1.7	2.4
120	1034.4 ± 0.8	1032.6 ± 0.9	1039.2	31.7 ± 1.1	35.8	38.3 ± 1.6	39.5	-1.4 ± 1.9	0.0
140	1040.7 ± 1.2	1039.3 ± 0.5	1046.4	31.0 ± 0.6	33.5	37.9 ± 0.9	37.8	-1.9 ± 1.0	−2.1
160	1047.3 ± 1.2	1044.9 ± 0.7	1053.2	28.4 ± 1.0	31.5	35.7 ± 1.3	36.3	-4.6 ± 1.5	−4.0
180	1051.9 ± 1.3	1051.0 ± 0.8	1059.7	26.4 ± 0.8	29.7	33.6 ± 1.1	35.0	-7.3 ± 1.4	−5.6
200	1058.5 ± 0.7	1057.0 ± 0.9	1065.9	26.3 ± 0.6	28.2	34.2 ± 0.9	33.8	-6.4 ± 1.1	−7.1

Table S428: Heat capacities at constant volume computed from MC simulations (c_V^{MC}), heat capacities at constant volume obtained from REFPROP[10] (c_V^{REFP}), heat capacities at constant pressure computed from MC simulations (c_P^{MC}), heat capacities at constant pressure obtained from REFPROP[10] (c_P^{REFP}), speed of sound computed from MC simulations (c^{MC}), speed of sound obtained from REFPROP[10] (c^{REFP}), viscosities computed from MD simulations (η^{MD}), and viscosities obtained from REFPROP[10] (η^{REFP}) of CO_2 rich quaternary mixture with 2% impurity of Ar, 1% impurity of N_2 and 1% impurity of CH_4 at 253 K and pressures ranging from 20 bar to 200 bar.

P/ [bar]	c_V^{MC}/ [J/mol K]	c_V^{REFP}/ [J/mol K]	c_P^{MC}/ [J/mol K]	c_P^{REFP}/ [J/mol K]	c^{MC}/ [m/s]	c^{REFP}/ [m/s]	η^{MD}/ [μPa s]	η^{REFP}/ [μPa s]
20	31.4	32.7	51.1 ± 0.3	54.7	226.6 ± 1.5	223.7	12.1 ± 2.4	13.1
40	40.1 ± 0.3	40.2	95.1 ± 2.6	93.8	745.4 ± 25.4	676.3	133.1 ± 6.9	128.9
60	39.9 ± 0.2	40.2	92.3 ± 1.6	91.4	772.1 ± 13.1	699.9	146.8 ± 6.1	132.9
80	40.0 ± 0.2	40.1	92.6 ± 2.7	89.4	784.7 ± 21.9	721.4	146.9 ± 3.6	136.8
100	40.1 ± 0.2	40.1	90.9 ± 2.0	87.8	801.2 ± 16.6	741.4	162.8 ± 30.0	140.5
120	40.3 ± 0.2	40.2	89.4 ± 2.8	86.3	822.8 ± 19.6	760.0	154.8 ± 4.4	144.1
140	40.2 ± 0.2	40.2	89.3 ± 1.6	85.1	830.2 ± 11.3	777.5	152.3 ± 9.4	147.5
160	40.2 ± 0.1	40.2	87.5 ± 1.8	84.0	856.2 ± 17.5	794.0	158.4 ± 12.4	150.9
180	40.4 ± 0.2	40.2	85.0 ± 1.7	83.1	871.3 ± 15.3	809.7	185.4 ± 41.0	154.2
200	40.3 ± 0.3	40.3	86.3 ± 1.5	82.2	877.3 ± 13.6	824.7	160.3 ± 4.3	157.4

Table S429: Densities computed from MC and MD simulations (ρ^{MC} and ρ^{MD}), densities obtained from REFPROP[10] (ρ^{REFP}), isothermal compressibilities computed from MC simulations (β_T^{MC}), isothermal compressibilities obtained from REFPROP[10] (β_T^{REFP}), thermal expansion coefficients computed from MC simulations (α_P^{MC}), thermal expansion coefficients obtained from REFPROP[10] (α_P^{REFP}), Joule Thomson coefficients computed from MC simulations (μ_{JT}^{MC}), and Joule Thomson coefficients obtained from REFPROP[10] (μ_{JT}^{REFP}) of CO_2 rich quaternary mixture with 2% impurity of Ar, 1% impurity of N_2 and 1% impurity of CH_4 at 273 K and pressures ranging from 20 bar to 200 bar.

P/ [bar]	ρ^{MC}/ [kg/m^3]	ρ^{MD}/ [kg/m^3]	ρ^{REFP}/ [kg/m^3]	β_T^{MC}/ [10^{-5}/bar]	β_T^{REFP}/ [10^{-5}/bar]	α_P^{MC}/ [10^{-4}/K]	α_P^{REFP}/ [10^{-4}/K]	μ_{JT}^{MC}/ [10^{-3} K/bar]	μ_{JT}^{REFP}/ [10^{-3} K/bar]
20	43.8	44.5 ± 0.1	44.6	5821.8 ± 42.8	5941.8	57.2 ± 0.4	60.3	1218.6 ± 25.9	1335.8
40	110.2 ± 0.1	886.7 ± 1.6	265.6	4461.5 ± 114.3	−2548.1	137.9 ± 3.9	−443.2	1316.8 ± 55.5	–
60	906.6 ± 2.0	905.1 ± 1.2	903.1	90.8 ± 5.1	108.8	70.8 ± 3.5	75.4	39.8 ± 4.3	46.7
80	921.8 ± 1.9	920.7 ± 0.9	920.9	76.4 ± 1.9	87.9	62.8 ± 1.0	65.1	31.7 ± 1.3	35.9
100	934.5 ± 0.8	933.7 ± 1.6	935.9	63.1 ± 1.3	74.3	54.7 ± 1.5	58.1	23.0 ± 2.0	27.9
120	945.7 ± 2.3	945.5 ± 1.2	948.9	58.3 ± 1.7	64.7	52.3 ± 1.3	52.9	20.0 ± 1.7	21.7
140	955.8 ± 0.8	955.0 ± 0.5	960.6	51.4 ± 2.5	57.5	47.9 ± 2.7	48.9	14.7 ± 3.6	16.6
160	966.8 ± 1.5	964.6 ± 0.4	971.1	45.5 ± 1.8	51.8	44.0 ± 1.9	45.7	9.9 ± 2.6	12.5
180	975.0 ± 0.9	973.2 ± 1.2	980.8	42.0 ± 2.1	47.3	41.6 ± 2.0	43.0	6.7 ± 2.8	8.9
200	983.4 ± 0.6	981.8 ± 0.5	989.7	37.9 ± 2.1	43.5	38.6 ± 1.9	40.8	2.7 ± 2.6	5.8

Table S430: Heat capacities at constant volume computed from MC simulations (c_V^{MC}), heat capacities at constant volume obtained from REFPROP[10] (c_V^{REFP}), heat capacities at constant pressure computed from MC simulations (c_P^{MC}), heat capacities at constant pressure obtained from REFPROP[10] (c_P^{REFP}), speed of sound computed from MC simulations (c^{MC}), speed of sound obtained from REFPROP[10] (c^{REFP}), viscosities computed from MD simulations (η^{MD}), and viscosities obtained from REFPROP[10] (η^{REFP}) of CO_2 rich quaternary mixture with 2% impurity of Ar, 1% impurity of N_2 and 1% impurity of CH_4 at 273 K and pressures ranging from 20 bar to 200 bar.

P/ [bar]	c_V^{MC}/ [J/mol K]	c_V^{REFP}/ [J/mol K]	c_P^{MC}/ [J/mol K]	c_P^{REFP}/ [J/mol K]	c^{MC}/ [m/s]	c^{REFP}/ [m/s]	η^{MD}/ [μPa s]	η^{REFP}/ [μPa s]
20	30.5	30.9	45.7 ± 0.1	47.1	242.6 ± 1.0	240.0	10.5 ± 3.1	14.1
40	36.5 ± 0.3	–	82.8 ± 1.4	–	214.9 ± 3.4	–	94.8 ± 2.6	19.5
60	39.7 ± 0.2	40.3	112.2 ± 3.2	109.0	586.2 ± 18.6	524.4	99.0 ± 1.8	94.2
80	39.3 ± 0.1	40.1	106.5 ± 1.6	102.1	620.5 ± 9.3	561.2	113.3 ± 11.0	99.1
100	39.4 ± 0.2	39.9	99.6 ± 2.1	97.4	654.9 ± 9.8	592.5	110.6 ± 10.4	103.4
120	39.4 ± 0.1	39.8	98.6 ± 1.4	93.9	673.9 ± 10.9	619.9	110.6 ± 3.2	107.4
140	39.3 ± 0.1	39.7	95.0 ± 3.6	91.1	700.9 ± 21.6	644.6	118.5 ± 9.8	111.1
160	39.5 ± 0.2	39.7	91.9 ± 2.6	88.9	727.6 ± 17.7	667.1	123.2 ± 8.2	114.6
180	39.3 ± 0.1	39.7	89.6 ± 2.6	87.1	746.1 ± 21.4	687.9	120.9 ± 3.6	117.9
200	39.6 ± 0.2	39.7	87.0 ± 2.1	85.5	767.9 ± 22.9	707.3	120.6 ± 7.3	121.2

Table S431: Densities computed from MC and MD simulations (ρ^{MC} and ρ^{MD}), densities obtained from REFPROP[10] (ρ^{REFP}), isothermal compressibilities computed from MC simulations (β_T^{MC}), isothermal compressibilities obtained from REFPROP[10] (β_T^{REFP}), thermal expansion coefficients computed from MC simulations (α_P^{MC}), thermal expansion coefficients obtained from REFPROP[10] (α_P^{REFP}), Joule Thomson coefficients computed from MC simulations (μ_{JT}^{MC}), and Joule Thomson coefficients obtained from REFPROP[10] (μ_{JT}^{REFP}) of CO_2 rich quaternary mixture with 2% impurity of Ar, 1% impurity of N_2 and 1% impurity of CH_4 at 293 K and pressures ranging from 20 bar to 200 bar.

P/ [bar]	ρ^{MC}/ [kg/m^3]	ρ^{MD}/ [kg/m^3]	ρ^{REFP}/ [kg/m^3]	β_T^{MC}/ [10^{-5}/bar]	β_T^{REFP}/ [10^{-5}/bar]	α_P^{MC}/ [10^{-4}/K]	α_P^{REFP}/ [10^{-4}/K]	μ_{JT}^{MC}/ [10^{-3} K/bar]	μ_{JT}^{REFP}/ [10^{-3} K/bar]
20	39.5	40.0 ± 0.1	40.0	5599.9 ± 32.1	5660.0	48.0 ± 0.4	49.6	1021.2 ± 27.1	1106.2
40	91.1 ± 0.1	92.9 ± 0.2	94.1	3420.8 ± 60.0	3509.8	78.3 ± 1.4	84.2	1056.8 ± 33.6	1100.7
60	177.3 ± 0.6	240.8 ± 67.8	193.0	3810.3 ± 222.7	4474.4	208.2 ± 15.4	275.5	1027.9 ± 111.5	1024.6
80	769.6 ± 2.8	766.7 ± 3.5	756.4	298.1 ± 37.6	404.0	142.4 ± 14.0	168.6	116.0 ± 16.7	140.8
100	805.2 ± 2.0	806.3 ± 1.4	801.7	193.3 ± 19.6	218.3	105.1 ± 9.6	108.2	85.5 ± 12.7	93.4
120	832.0 ± 1.8	830.4 ± 1.9	831.4	131.4 ± 6.8	153.5	80.0 ± 2.7	84.7	61.5 ± 3.8	69.3
140	850.8 ± 1.9	850.1 ± 1.2	854.1	109.0 ± 5.4	119.6	70.9 ± 2.6	71.6	50.6 ± 3.7	53.8
160	870.3 ± 1.0	868.0 ± 1.1	872.8	87.4 ± 2.4	98.5	61.4 ± 1.9	62.9	39.0 ± 2.8	42.7
180	884.4 ± 1.2	881.7 ± 0.8	888.9	75.1 ± 4.3	84.0	55.1 ± 3.4	56.6	31.0 ± 5.2	34.3
200	898.1 ± 1.0	895.1 ± 1.0	902.9	67.1 ± 2.4	73.4	51.7 ± 1.8	51.9	26.0 ± 2.6	27.5

Table S432: Heat capacities at constant volume computed from MC simulations (c_V^{MC}), heat capacities at constant volume obtained from REFPROP[10] (c_V^{REFP}), heat capacities at constant pressure computed from MC simulations (c_P^{MC}), heat capacities at constant pressure obtained from REFPROP[10] (c_P^{REFP}), speed of sound computed from MC simulations (c^{MC}), speed of sound obtained from REFPROP[10] (c^{REFP}), viscosities computed from MD simulations (η^{MD}), and viscosities obtained from REFPROP[10] (η^{REFP}) of CO_2 rich quaternary mixture with 2% impurity of Ar, 1% impurity of N_2 and 1% impurity of CH_4 at 293 K and pressures ranging from 20 bar to 200 bar.

P/ [bar]	c_V^{MC}/ [J/mol K]	c_V^{REFP}/ [J/mol K]	c_P^{MC}/ [J/mol K]	c_P^{REFP}/ [J/mol K]	c^{MC}/ [m/s]	c^{REFP}/ [m/s]	η^{MD}/ [μPa s]	η^{REFP}/ [μPa s]
20	30.5	30.6	43.8 ± 0.1	44.4	254.8 ± 0.8	253.1	10.5 ± 5.9	15.1
40	33.4	34.3	58.5 ± 0.5	61.6	237.1 ± 2.3	233.4	15.7 ± 0.7	15.8
60	39.5 ± 0.3	43.5	121.7 ± 7.6	155.5	213.5 ± 9.2	203.4	19.3 ± 1.9	18.0
80	40.0 ± 0.4	42.4	154.6 ± 9.8	160.9	410.2 ± 29.0	352.3	71.3 ± 4.4	64.3
100	39.3 ± 0.2	40.9	131.4 ± 7.8	126.1	463.4 ± 27.3	419.8	82.8 ± 11.5	72.0
120	39.4 ± 0.2	40.2	114.4 ± 1.9	111.9	515.2 ± 14.1	467.0	80.6 ± 9.0	77.7
140	39.0 ± 0.1	39.8	108.7 ± 2.0	103.7	548.2 ± 14.5	504.8	84.2 ± 2.7	82.4
160	39.0 ± 0.2	39.6	102.3 ± 2.3	98.2	587.1 ± 10.5	536.8	100.9 ± 13.7	86.6
180	39.0 ± 0.1	39.5	97.4 ± 3.9	94.2	613.0 ± 21.6	565.1	94.2 ± 6.8	90.4
200	39.1 ± 0.2	39.4	95.7 ± 1.9	91.2	637.4 ± 13.1	590.5	96.8 ± 3.4	93.9

Table S433: Densities computed from MC and MD simulations (ρ^{MC} and ρ^{MD}), densities obtained from REFPROP[10] (ρ^{REFP}), isothermal compressibilities computed from MC simulations (β_T^{MC}), isothermal compressibilities obtained from REFPROP[10] (β_T^{REFP}), thermal expansion coefficients computed from MC simulations (α_P^{MC}), thermal expansion coefficients obtained from REFPROP[10] (α_P^{REFP}), Joule Thomson coefficients computed from MC simulations (μ_{JT}^{MC}), and Joule Thomson coefficients obtained from REFPROP[10] (μ_{JT}^{REFP}) of CO_2 rich quaternary mixture with 2% impurity of Ar, 1% impurity of N_2 and 1% impurity of CH_4 at 313 K and pressures ranging from 20 bar to 200 bar.

P/ [bar]	ρ^{MC}/ [kg/m^3]	ρ^{MD}/ [kg/m^3]	ρ^{REFP}/ [kg/m^3]	β_T^{MC}/ [10^{-5}/bar]	β_T^{REFP}/ [10^{-5}/bar]	α_P^{MC}/ [10^{-4}/K]	α_P^{REFP}/ [10^{-4}/K]	μ_{JT}^{MC}/ [10^{-3} K/bar]	μ_{JT}^{REFP}/ [10^{-3} K/bar]
20	36.1	36.6 ± 0.1	36.5	5454.1 ± 41.5	5489.7	41.8 ± 0.3	42.8	866.2 ± 27.4	932.5
40	79.8 ± 0.1	81.1 ± 0.2	81.6	3084.6 ± 23.6	3133.7	58.4 ± 0.4	61.2	877.2 ± 14.5	922.0
60	136.7 ± 0.1	139.7 ± 0.6	142.4	2473.9 ± 31.4	2577.2	90.8 ± 1.5	99.8	856.7 ± 22.7	888.6
80	225.3 ± 1.2	237.2 ± 2.6	242.1	2758.4 ± 90.5	2906.8	196.2 ± 7.8	223.1	801.7 ± 47.3	–
100	471.6 ± 8.5	471.1 ± 9.4	167.1	3335.8 ± 382.0	2567.7	627.4 ± 76.9	122.0	438.8 ± 75.1	867.7
120	640.0 ± 5.9	637.9 ± 6.2	639.5	689.4 ± 99.7	709.1	219.1 ± 24.8	211.1	208.8 ± 31.7	216.3
140	707.0 ± 1.7	703.5 ± 1.2	705.4	307.1 ± 8.5	350.3	124.1 ± 3.0	129.8	129.5 ± 5.1	141.2
160	746.6 ± 3.3	742.6 ± 1.3	746.3	211.0 ± 6.5	230.5	98.4 ± 2.0	98.0	98.1 ± 3.2	103.7
180	772.8 ± 2.1	771.1 ± 1.3	776.5	158.5 ± 9.5	171.7	80.4 ± 4.3	80.7	76.6 ± 7.2	80.4
200	793.1 ± 2.7	793.9 ± 0.9	800.6	132.0 ± 10.0	136.8	71.8 ± 4.7	69.7	64.4 ± 8.0	64.2

Table S434: Heat capacities at constant volume computed from MC simulations (c_V^{MC}), heat capacities at constant volume obtained from REFPROP[10] (c_V^{REFP}), heat capacities at constant pressure computed from MC simulations (c_P^{MC}), heat capacities at constant pressure obtained from REFPROP[10] (c_P^{REFP}), speed of sound computed from MC simulations (c^{MC}), speed of sound obtained from REFPROP[10] (c^{REFP}), viscosities computed from MD simulations (η^{MD}), and viscosities obtained from REFPROP[10] (η^{REFP}) of CO_2 rich quaternary mixture with 2% impurity of Ar, 1% impurity of N_2 and 1% impurity of CH_4 at 313 K and pressures ranging from 20 bar to 200 bar.

P/ [bar]	c_V^{MC}/ [J/mol K]	c_V^{REFP}/ [J/mol K]	c_P^{MC}/ [J/mol K]	c_P^{REFP}/ [J/mol K]	c^{MC}/ [m/s]	c^{REFP}/ [m/s]	η^{MD}/ [μPa s]	η^{REFP}/ [μPa s]
20	30.8	30.8	42.9 ± 0.1	43.3	266.0 ± 1.1	264.5	5.6 ± 4.3	16.1
40	32.6	33.0	51.5 ± 0.1	53.0	253.3 ± 1.0	250.5	16.7 ± 0.7	16.7
60	35.2	36.1	68.4 ± 0.7	73.0	239.8 ± 1.9	234.9	19.2 ± 2.6	17.8
80	38.7 ± 0.2	–	123.8 ± 4.3	–	226.9 ± 5.5	–	20.5 ± 1.2	20.8
100	42.4 ± 0.6	37.3	392.1 ± 43.5	84.5	242.6 ± 19.5	229.8	30.4 ± 1.6	18.4
120	40.0 ± 0.2	42.5	190.7 ± 14.0	176.3	328.6 ± 26.7	302.5	44.2 ± 5.7	49.1
140	39.3 ± 0.2	40.9	137.0 ± 3.0	133.7	400.6 ± 7.1	363.6	56.6 ± 1.2	57.4
160	39.1 ± 0.2	40.2	123.6 ± 1.6	116.1	447.7 ± 7.6	410.0	66.4 ± 3.7	63.3
180	38.9 ± 0.2	39.7	111.4 ± 3.6	106.3	483.4 ± 16.5	447.9	69.3 ± 1.8	68.1
200	38.8 ± 0.1	39.5	106.3 ± 4.2	99.8	511.5 ± 21.9	480.4	75.1 ± 3.3	72.3

S17.10 Data of thermodynamics and transport properties of CO_2 rich quaternary mixture with 1 mole% impurity of N_2, 1 mole% impurity of N_2, and 2 mole% impurity of H_2

Table S435: Densities computed from MC and MD simulations (ρ^{MC} and ρ^{MD}), densities obtained from REFPROP[10] (ρ^{REFP}), isothermal compressibilities computed from MC simulations (β_T^{MC}), isothermal compressibilities obtained from REFPROP[10] (β_T^{REFP}), thermal expansion coefficients computed from MC simulations (α_P^{MC}), thermal expansion coefficients obtained from REFPROP[10] (α_P^{REFP}), Joule Thomson coefficients computed from MC simulations (μ_{JT}^{MC}), and Joule Thomson coefficients obtained from REFPROP[10] (μ_{JT}^{REFP}) of CO_2 rich quaternary mixture with 2% impurity of H_2, 1% impurity of Ar and 1% impurity of N_2 at 253 K and pressures ranging from 20 bar to 200 bar.

P/ [bar]	ρ^{MC}/ [kg/m^3]	ρ^{MD}/ [kg/m^3]	ρ^{REFP}/ [kg/m^3]	β_T^{MC}/ [10^{-5}/bar]	β_T^{REFP}/ [10^{-5}/bar]	α_P^{MC}/ [10^{-4}/K]	α_P^{REFP}/ [10^{-4}/K]	μ_{JT}^{MC}/ [10^{-3} K/bar]	μ_{JT}^{REFP}/ [10^{-3} K/bar]
20	49.0	49.8 ± 0.2	50.4	6227.0 ± 56.9	6465.9	73.0 ± 0.8	80.1	1474.1 ± 36.9	1626.8
40	987.4 ± 0.8	986.7 ± 1.4	691.0	48.6 ± 1.7	-61.6	50.7 ± 1.7	44.4	12.5 ± 2.0	–
60	997.6 ± 1.2	996.1 ± 0.8	957.8	45.3 ± 1.9	72.0	48.8 ± 1.8	62.6	10.4 ± 2.1	–
80	1006.9 ± 0.9	1004.7 ± 1.6	1001.1	39.0 ± 2.0	44.1	43.6 ± 2.4	44.8	4.8 ± 2.8	6.4
100	1014.7 ± 1.0	1012.1 ± 0.5	1009.6	36.3 ± 0.7	40.5	41.9 ± 0.8	42.3	2.7 ± 1.0	3.5
120	1021.0 ± 1.0	1019.6 ± 0.5	1017.5	33.2 ± 1.5	37.5	39.0 ± 1.9	40.3	-0.7 ± 2.3	0.9
140	1027.9 ± 1.4	1026.9 ± 0.9	1024.9	33.2 ± 1.1	34.9	39.6 ± 1.3	38.5	0.1 ± 1.6	-1.3
160	1034.2 ± 1.1	1033.0 ± 0.4	1031.9	30.9 ± 1.6	32.8	37.9 ± 2.0	36.9	-1.9 ± 2.4	-3.3
180	1040.8 ± 0.8	1038.1 ± 0.4	1038.5	28.2 ± 0.7	30.9	35.0 ± 0.9	35.5	-5.5 ± 1.1	-5.1
200	1045.8 ± 0.4	1045.4 ± 1.0	1044.7	27.6 ± 0.7	29.2	35.2 ± 1.1	34.2	-5.2 ± 1.4	-6.7

Table S436: Heat capacities at constant volume computed from MC simulations (c_V^{MC}), heat capacities at constant volume obtained from REFPROP[10] (c_V^{REFP}), heat capacities at constant pressure computed from MC simulations (c_P^{MC}), heat capacities at constant pressure obtained from REFPROP[10] (c_P^{REFP}), speed of sound computed from MC simulations (c^{MC}), speed of sound obtained from REFPROP[10] (c^{REFP}), viscosities computed from MD simulations (η^{MD}), and viscosities obtained from REFPROP[10] (η^{REFP}) of CO_2 rich quaternary mixture with 2% impurity of H_2, 1% impurity of Ar and 1% impurity of N_2 at 253 K and pressures ranging from 20 bar to 200 bar.

P/ [bar]	c_V^{MC}/ [J/mol K]	c_V^{REFP}/ [J/mol K]	c_P^{MC}/ [J/mol K]	c_P^{REFP}/ [J/mol K]	c^{MC}/ [m/s]	c^{REFP}/ [m/s]	η^{MD}/ [μPa s]	η^{REFP}/ [μPa s]
20	31.4	32.3	50.3 ± 0.3	53.7	229.2 ± 1.3	225.7	13.3 ± 5.8	13.0
40	40.0 ± 0.1	–	98.6 ± 2.0	–	716.4 ± 14.6	–	131.7 ± 3.3	60.0
60	40.1 ± 0.2	–	97.6 ± 2.3	–	733.5 ± 17.4	–	134.8 ± 3.0	113.3
80	40.0 ± 0.1	40.0	93.0 ± 3.5	89.5	769.8 ± 24.8	711.6	134.3 ± 6.7	130.0
100	40.2 ± 0.3	40.0	92.0 ± 1.0	87.7	788.3 ± 9.0	732.2	145.7 ± 18.0	133.6
120	40.1 ± 0.2	40.0	88.9 ± 2.7	86.2	808.9 ± 22.4	751.4	146.0 ± 4.4	137.1
140	40.4 ± 0.3	40.0	90.1 ± 2.0	84.9	808.1 ± 16.9	769.4	143.7 ± 7.9	140.5
160	40.3 ± 0.2	40.1	89.1 ± 3.0	83.8	830.6 ± 25.3	786.4	168.6 ± 20.9	143.8
180	40.4 ± 0.3	40.1	85.4 ± 1.3	82.8	848.8 ± 13.1	802.5	156.6 ± 4.4	147.1
200	40.3 ± 0.2	40.1	87.0 ± 1.9	81.9	864.3 ± 14.5	817.9	154.5 ± 3.0	150.2

Table S437: Densities computed from MC and MD simulations (ρ^{MC} and ρ^{MD}), densities obtained from REFPROP[10] (ρ^{REFP}), isothermal compressibilities computed from MC simulations (β_T^{MC}), isothermal compressibilities obtained from REFPROP[10] (β_T^{REFP}), thermal expansion coefficients computed from MC simulations (α_P^{MC}), thermal expansion coefficients obtained from REFPROP[10] (α_P^{REFP}), Joule Thomson coefficients computed from MC simulations ($\mu_{\mathrm{JT}}^{\mathrm{MC}}$), and Joule Thomson coefficients obtained from REFPROP[10] ($\mu_{\mathrm{JT}}^{\mathrm{REFP}}$) of CO_2 rich quaternary mixture with 2% impurity of H_2, 1% impurity of Ar and 1% impurity of N_2 at 273 K and pressures ranging from 20 bar to 200 bar.

P/ [bar]	ρ^{MC}/ [kg/m^3]	ρ^{MD}/ [kg/m^3]	ρ^{REFP}/ [kg/m^3]	β_T^{MC}/ [10^{-5}/bar]	β_T^{REFP}/ [10^{-5}/bar]	α_P^{MC}/ [10^{-4}/K]	α_P^{REFP}/ [10^{-4}/K]	$\mu_{\mathrm{JT}}^{\mathrm{MC}}$/ [$10^{-3}$ K/bar]	$\mu_{\mathrm{JT}}^{\mathrm{REFP}}$/ [$10^{-3}$ K/bar]
20	43.1	43.8 ± 0.1	44.0	5814.7 ± 38.2	5919.4	56.8 ± 0.5	59.7	1203.8 ± 28.4	1314.2
40	107.7 ± 0.4	863.1 ± 1.6	228.7	4222.6 ± 70.2	−7115.6	128.5 ± 3.2	−804.6	1264.5 ± 50.1	–
60	883.9 ± 2.9	884.7 ± 0.9	786.9	104.1 ± 6.8	668.7	76.6 ± 5.0	296.1	46.0 ± 6.1	–
80	903.6 ± 1.8	902.3 ± 1.1	898.0	87.8 ± 14.5	95.0	67.9 ± 8.3	67.8	37.4 ± 10.1	39.5
100	916.6 ± 0.8	915.9 ± 1.4	913.7	74.4 ± 5.8	79.4	61.4 ± 4.0	60.0	30.2 ± 5.0	30.6
120	929.1 ± 0.9	928.4 ± 1.6	927.3	64.8 ± 3.1	68.5	55.5 ± 2.3	54.3	23.9 ± 3.0	23.8
140	940.1 ± 1.8	939.5 ± 0.4	939.3	55.2 ± 1.9	60.5	49.1 ± 1.5	50.0	16.5 ± 2.1	18.3
160	952.1 ± 1.7	949.4 ± 0.3	950.1	50.5 ± 2.1	54.3	47.1 ± 2.2	46.6	13.7 ± 2.9	13.8
180	960.6 ± 1.6	958.6 ± 1.0	960.0	46.2 ± 1.1	49.4	44.2 ± 1.4	43.8	10.1 ± 1.8	10.0
200	969.4 ± 0.4	968.3 ± 0.6	969.1	42.5 ± 1.7	45.3	42.0 ± 1.5	41.4	7.2 ± 2.0	6.8

Table S438: Heat capacities at constant volume computed from MC simulations (c_V^{MC}), heat capacities at constant volume obtained from REFPROP[10] (c_V^{REFP}), heat capacities at constant pressure computed from MC simulations (c_P^{MC}), heat capacities at constant pressure obtained from REFPROP[10] (c_P^{REFP}), speed of sound computed from MC simulations (c^{MC}), speed of sound obtained from REFPROP[10] (c^{REFP}), viscosities computed from MD simulations (η^{MD}), and viscosities obtained from REFPROP[10] (η^{REFP}) of CO_2 rich quaternary mixture with 2% impurity of H_2, 1% impurity of Ar and 1% impurity of N_2 at 273 K and pressures ranging from 20 bar to 200 bar.

P/ [bar]	c_V^{MC}/ [J/mol K]	c_V^{REFP}/ [J/mol K]	c_P^{MC}/ [J/mol K]	c_P^{REFP}/ [J/mol K]	c^{MC}/ [m/s]	c^{REFP}/ [m/s]	η^{MD}/ [μPa s]	η^{REFP}/ [μPa s]
20	30.5	30.7	45.6 ± 0.2	46.8	244.1 ± 0.9	241.8	13.3 ± 0.6	14.1
40	36.5 ± 0.3	–	79.2 ± 1.5	–	218.4 ± 2.8	–	88.9 ± 3.9	17.9
60	40.0 ± 0.3	–	115.3 ± 4.9	–	560.1 ± 22.0	–	95.6 ± 3.4	69.4
80	39.6 ± 0.1	40.0	108.6 ± 6.4	103.1	588.1 ± 51.7	550.0	99.9 ± 2.1	93.9
100	39.5 ± 0.1	39.8	105.2 ± 3.7	97.9	624.7 ± 27.0	582.6	107.2 ± 1.5	98.2
120	39.4 ± 0.2	39.7	99.8 ± 2.5	94.1	648.6 ± 17.8	611.1	109.3 ± 6.1	102.2
140	39.5 ± 0.1	39.6	94.2 ± 2.2	91.2	677.5 ± 14.1	636.6	117.1 ± 6.2	105.9
160	39.4 ± 0.1	39.5	93.9 ± 3.1	88.8	703.9 ± 18.5	659.8	115.4 ± 8.7	109.3
180	39.5 ± 0.2	39.5	91.2 ± 2.0	86.9	721.3 ± 11.6	681.1	121.9 ± 10.2	112.6
200	39.5 ± 0.3	39.5	89.9 ± 1.8	85.3	743.4 ± 16.4	701.0	126.6 ± 12.6	115.7

Table S439: Densities computed from MC and MD simulations (ρ^{MC} and ρ^{MD}), densities obtained from REFPROP[10] (ρ^{REFP}), isothermal compressibilities computed from MC simulations (β_T^{MC}), isothermal compressibilities obtained from REFPROP[10] (β_T^{REFP}), thermal expansion coefficients computed from MC simulations (α_P^{MC}), thermal expansion coefficients obtained from REFPROP[10] (α_P^{REFP}), Joule Thomson coefficients computed from MC simulations ($\mu_{\mathrm{JT}}^{\mathrm{MC}}$), and Joule Thomson coefficients obtained from REFPROP[10] ($\mu_{\mathrm{JT}}^{\mathrm{REFP}}$) of CO_2 rich quaternary mixture with 2% impurity of H_2, 1% impurity of Ar and 1% impurity of N_2 at 293 K and pressures ranging from 20 bar to 200 bar.

P/ [bar]	ρ^{MC}/ [kg/m^3]	ρ^{MD}/ [kg/m^3]	ρ^{REFP}/ [kg/m^3]	β_T^{MC}/ [10^{-5}/bar]	β_T^{REFP}/ [10^{-5}/bar]	α_P^{MC}/ [10^{-4}/K]	α_P^{REFP}/ [10^{-4}/K]	$\mu_{\mathrm{JT}}^{\mathrm{MC}}$/ [$10^{-3}$ K/bar]	$\mu_{\mathrm{JT}}^{\mathrm{REFP}}$/ [$10^{-3}$ K/bar]
20	38.9	39.4 ± 0.1	39.5	5564.7 ± 26.6	5647.1	47.4 ± 0.3	49.3	987.1 ± 19.7	1089.7
40	89.3 ± 0.1	91.1 ± 0.3	92.4	3375.7 ± 37.2	3474.2	76.6 ± 1.0	82.3	1034.2 ± 26.4	1084.0
60	170.0 ± 0.9	177.1 ± 1.6	185.3	3458.0 ± 190.7	4088.3	183.1 ± 11.7	239.2	989.9 ± 91.8	1016.8
80	732.1 ± 4.9	726.3 ± 4.8	725.3	433.7 ± 73.4	506.0	185.4 ± 24.7	196.2	144.6 ± 26.7	161.0
100	780.3 ± 4.6	776.5 ± 1.4	776.7	227.9 ± 18.6	245.5	115.2 ± 7.4	115.9	96.6 ± 9.6	102.3
120	811.0 ± 0.9	807.1 ± 1.3	808.5	155.9 ± 10.9	167.0	90.5 ± 6.1	88.6	71.9 ± 8.5	74.9
140	830.9 ± 2.0	830.4 ± 1.4	832.4	123.6 ± 7.2	128.0	76.1 ± 3.7	74.0	57.3 ± 5.4	57.7
160	851.4 ± 1.8	849.7 ± 0.6	851.9	97.2 ± 2.2	104.3	64.9 ± 1.6	64.6	43.9 ± 2.4	45.6
180	866.7 ± 1.7	865.3 ± 2.0	868.4	85.1 ± 1.8	88.4	60.1 ± 1.1	57.9	37.3 ± 1.6	36.5
200	880.4 ± 1.8	879.5 ± 0.5	882.8	72.3 ± 3.0	76.8	53.4 ± 2.4	52.8	28.8 ± 3.7	29.3

Table S440: Heat capacities at constant volume computed from MC simulations (c_V^{MC}), heat capacities at constant volume obtained from REFPROP[10] (c_V^{REFP}), heat capacities at constant pressure computed from MC simulations (c_P^{MC}), heat capacities at constant pressure obtained from REFPROP[10] (c_P^{REFP}), speed of sound computed from MC simulations (c^{MC}), speed of sound obtained from REFPROP[10] (c^{REFP}), viscosities computed from MD simulations (η^{MD}), and viscosities obtained from REFPROP[10] (η^{REFP}) of CO_2 rich quaternary mixture with 2% impurity of H_2, 1% impurity of Ar and 1% impurity of N_2 at 293 K and pressures ranging from 20 bar to 200 bar.

P/ [bar]	c_V^{MC}/ [J/mol K]	c_V^{REFP}/ [J/mol K]	c_P^{MC}/ [J/mol K]	c_P^{REFP}/ [J/mol K]	c^{MC}/ [m/s]	c^{REFP}/ [m/s]	η^{MD}/ [μPa s]	η^{REFP}/ [μPa s]
20	30.5	30.6	43.6 ± 0.1	44.3	256.9 ± 0.7	254.9	12.4 ± 2.5	15.0
40	33.3	34.0	57.8 ± 0.5	60.5	239.8 ± 1.6	235.6	16.0 ± 2.5	15.7
60	39.0 ± 0.5	41.9	111.5 ± 5.5	137.0	220.4 ± 8.3	207.6	20.5 ± 4.7	17.8
80	40.5 ± 0.4	42.7	179.8 ± 15.4	174.8	374.1 ± 35.5	333.9	60.4 ± 3.3	59.7
100	40.0 ± 0.5	40.8	135.4 ± 5.5	129.5	436.5 ± 20.1	407.8	72.0 ± 2.5	68.0
120	39.5 ± 0.2	40.1	121.8 ± 5.5	113.4	493.7 ± 20.6	457.5	75.4 ± 3.0	73.7
140	39.4 ± 0.2	39.7	111.0 ± 3.3	104.4	523.7 ± 17.1	496.7	79.9 ± 4.6	78.5
160	39.2 ± 0.1	39.5	103.8 ± 2.0	98.5	565.7 ± 8.3	529.8	85.2 ± 3.2	82.6
180	39.2 ± 0.1	39.4	101.2 ± 1.1	94.3	591.8 ± 7.3	558.7	92.7 ± 6.3	86.3
200	39.1 ± 0.2	39.3	95.6 ± 2.8	91.1	619.3 ± 15.8	584.7	95.0 ± 3.2	89.8

Table S441: Densities computed from MC and MD simulations (ρ^{MC} and ρ^{MD}), densities obtained from REFPROP[10] (ρ^{REFP}), isothermal compressibilities computed from MC simulations (β_T^{MC}), isothermal compressibilities obtained from REFPROP[10] (β_T^{REFP}), thermal expansion coefficients computed from MC simulations (α_P^{MC}), thermal expansion coefficients obtained from REFPROP[10] (α_P^{REFP}), Joule Thomson coefficients computed from MC simulations (μ_{JT}^{MC}), and Joule Thomson coefficients obtained from REFPROP[10] (μ_{JT}^{REFP}) of CO_2 rich quaternary mixture with 2% impurity of H_2, 1% impurity of Ar and 1% impurity of N_2 at 313 K and pressures ranging from 20 bar to 200 bar.

P/ [bar]	ρ^{MC}/ [kg/m^3]	ρ^{MD}/ [kg/m^3]	ρ^{REFP}/ [kg/m^3]	β_T^{MC}/ [10^{-5}/bar]	β_T^{REFP}/ [10^{-5}/bar]	α_P^{MC}/ [10^{-4}/K]	α_P^{REFP}/ [10^{-4}/K]	μ_{JT}^{MC}/ [10^{-3} K/bar]	μ_{JT}^{REFP}/ [10^{-3} K/bar]
20	35.6	36.1 ± 0.1	36.0	5436.2 ± 18.1	5481.2	41.6 ± 0.1	42.6	849.2 ± 13.2	919.6
40	78.4 ± 0.1	79.7 ± 0.2	80.3	3060.5 ± 20.3	3117.6	57.6 ± 0.3	60.5	860.0 ± 10.2	908.8
60	133.4 ± 0.2	136.8 ± 0.4	139.4	2407.7 ± 52.1	2538.7	87.0 ± 2.1	96.8	830.9 ± 34.1	876.5
80	215.5 ± 0.5	224.0 ± 2.1	233.1	2401.4 ± 64.8	2749.2	162.2 ± 4.5	202.7	756.3 ± 30.5	779.2
100	392.4 ± 4.2	407.6 ± 7.2	439.5	3473.7 ± 257.3	3027.8	525.6 ± 42.9	500.5	527.7 ± 61.2	480.2
120	596.7 ± 2.9	590.3 ± 6.7	610.8	814.8 ± 37.4	811.8	231.8 ± 6.9	227.2	234.9 ± 9.3	235.7
140	678.6 ± 2.4	672.8 ± 2.1	681.9	377.8 ± 27.5	384.4	141.2 ± 9.0	136.1	148.7 ± 13.9	151.3
160	719.7 ± 3.4	717.7 ± 2.8	724.9	249.7 ± 18.0	247.1	107.9 ± 6.5	101.2	111.5 ± 10.4	109.9
180	750.7 ± 1.6	747.8 ± 1.8	756.2	182.8 ± 4.6	181.7	87.9 ± 2.2	82.7	86.8 ± 3.8	84.8
200	776.5 ± 1.0	772.2 ± 0.8	781.0	144.3 ± 5.4	143.6	75.4 ± 2.1	71.0	69.9 ± 3.5	67.4

Table S442: Heat capacities at constant volume computed from MC simulations (c_V^{MC}), heat capacities at constant volume obtained from REFPROP[10] (c_V^{REFP}), heat capacities at constant pressure computed from MC simulations (c_P^{MC}), heat capacities at constant pressure obtained from REFPROP[10] (c_P^{REFP}), speed of sound computed from MC simulations (c^{MC}), speed of sound obtained from REFPROP[10] (c^{REFP}), viscosities computed from MD simulations (η^{MD}), and viscosities obtained from REFPROP[10] (η^{REFP}) of CO_2 rich quaternary mixture with 2% impurity of H_2, 1% impurity of Ar and 1% impurity of N_2 at 313 K and pressures ranging from 20 bar to 200 bar.

P/ [bar]	c_V^{MC}/ [J/mol K]	c_V^{REFP}/ [J/mol K]	c_P^{MC}/ [J/mol K]	c_P^{REFP}/ [J/mol K]	c^{MC}/ [m/s]	c^{REFP}/ [m/s]	η^{MD}/ [μPa s]	η^{REFP}/ [μPa s]
20	30.8	30.8	42.8 ± 0.1	43.1	268.0 ± 0.5	266.3	9.0 ± 7.1	16.0
40	32.6	32.9	51.2 ± 0.1	52.5	255.9 ± 0.9	252.5	16.9 ± 2.1	16.6
60	35.0 ± 0.1	35.7	66.9 ± 0.9	71.3	243.8 ± 3.1	237.4	18.1 ± 0.9	17.7
80	38.9 ± 0.4	40.2	107.5 ± 2.2	126.4	231.0 ± 4.1	221.5	20.3 ± 1.5	20.4
100	42.5 ± 0.6	45.4	320.8 ± 24.4	298.6	235.5 ± 12.7	222.4	27.9 ± 2.2	30.9
120	41.0 ± 0.2	42.4	191.8 ± 3.5	182.4	310.2 ± 7.7	294.7	42.0 ± 2.2	45.9
140	39.8 ± 0.4	40.8	145.6 ± 6.4	135.9	377.9 ± 16.2	356.6	54.6 ± 2.4	54.4
160	39.5 ± 0.4	40.0	127.4 ± 4.9	116.9	423.6 ± 17.5	404.0	66.7 ± 15.8	60.3
180	39.2 ± 0.2	39.6	115.4 ± 2.2	106.6	463.1 ± 7.4	442.6	62.7 ± 3.0	65.1
200	38.9 ± 0.2	39.3	107.6 ± 1.5	99.8	496.8 ± 9.9	475.7	67.8 ± 5.4	69.2

S17.11 Data of thermodynamics and transport properties of CO_2 rich quaternary mixture with 1 mole% impurity of N_2, 2 mole% impurity of N_2, and 1 mole% impurity of H_2

Table S443: Densities computed from MC and MD simulations (ρ^{MC} and ρ^{MD}), densities obtained from REFPROP[10] (ρ^{REFP}), isothermal compressibilities computed from MC simulations (β_T^{MC}), isothermal compressibilities obtained from REFPROP[10] (β_T^{REFP}), thermal expansion coefficients computed from MC simulations (α_P^{MC}), thermal expansion coefficients obtained from REFPROP[10] (α_P^{REFP}), Joule Thomson coefficients computed from MC simulations (μ_{JT}^{MC}), and Joule Thomson coefficients obtained from REFPROP[10] (μ_{JT}^{REFP}) of CO_2 rich quaternary mixture with 2% impurity of N_2, 1% impurity of Ar and 1% impurity of H_2 at 253 K and pressures ranging from 20 bar to 200 bar.

P/ [bar]	ρ^{MC}/ [kg/m^3]	ρ^{MD}/ [kg/m^3]	ρ^{REFP}/ [kg/m^3]	β_T^{MC}/ [10^{-5}/bar]	β_T^{REFP}/ [10^{-5}/bar]	α_P^{MC}/ [10^{-4}/K]	α_P^{REFP}/ [10^{-4}/K]	μ_{JT}^{MC}/ [10^{-3} K/bar]	μ_{JT}^{REFP}/ [10^{-3} K/bar]
20	49.4 ± 0.1	50.1 ± 0.1	50.9	6324.4 ± 9.6	6490.0	74.6 ± 0.3	80.9	1526.7 ± 12.6	1641.2
40	994.1 ± 2.1	991.8 ± 1.0	765.7	45.8 ± 2.9	−220.1	47.9 ± 2.7	−30.5	9.6 ± 3.1	–
60	1002.6 ± 1.4	1000.1 ± 0.9	1001.6	42.1 ± 2.8	48.0	45.9 ± 3.4	47.6	7.4 ± 3.9	9.6
80	1010.5 ± 1.8	1009.4 ± 0.6	1010.8	39.3 ± 2.6	43.7	44.5 ± 2.9	44.7	5.6 ± 3.3	6.2
100	1018.1 ± 1.6	1016.8 ± 0.9	1019.3	36.9 ± 3.0	40.1	42.3 ± 2.9	42.2	3.2 ± 3.4	3.3
120	1025.7 ± 1.0	1024.1 ± 0.9	1027.1	33.4 ± 1.1	37.2	39.0 ± 1.1	40.1	-0.6 ± 1.3	0.8
140	1033.7 ± 0.6	1030.0 ± 1.0	1034.5	30.7 ± 1.0	34.7	37.3 ± 1.4	38.4	-2.7 ± 1.6	−1.4
160	1039.1 ± 0.6	1036.6 ± 0.6	1041.5	29.2 ± 0.8	32.5	35.7 ± 1.4	36.8	-4.6 ± 1.7	−3.4
180	1044.6 ± 0.4	1042.9 ± 0.6	1048.1	28.9 ± 0.9	30.6	36.3 ± 0.9	35.4	-3.8 ± 1.1	−5.2
200	1050.5 ± 1.0	1049.0 ± 1.1	1054.4	26.9 ± 0.9	29.0	34.3 ± 1.3	34.2	-6.4 ± 1.6	−6.7

Table S444: Heat capacities at constant volume computed from MC simulations (c_V^{MC}), heat capacities at constant volume obtained from REFPROP[10] (c_V^{REFP}), heat capacities at constant pressure computed from MC simulations (c_P^{MC}), heat capacities at constant pressure obtained from REFPROP[10] (c_P^{REFP}), speed of sound computed from MC simulations (c^{MC}), speed of sound obtained from REFPROP[10] (c^{REFP}), viscosities computed from MD simulations (η^{MD}), and viscosities obtained from REFPROP[10] (η^{REFP}) of CO_2 rich quaternary mixture with 2% impurity of N_2, 1% impurity of Ar and 1% impurity of H_2 at 253 K and pressures ranging from 20 bar to 200 bar.

P/ [bar]	c_V^{MC}/ [J/mol K]	c_V^{REFP}/ [J/mol K]	c_P^{MC}/ [J/mol K]	c_P^{REFP}/ [J/mol K]	c^{MC}/ [m/s]	c^{REFP}/ [m/s]	η^{MD}/ [μPa s]	η^{REFP}/ [μPa s]
20	31.4 ± 0.1	32.5	50.8 ± 0.2	54.2	227.6 ± 0.5	224.7	10.7 ± 1.7	13.1
40	39.9 ± 0.1	–	95.4 ± 3.2	–	724.5 ± 25.8	–	133.1 ± 2.5	67.7
60	40.1 ± 0.1	40.2	94.8 ± 4.4	91.7	747.9 ± 30.1	688.6	158.3 ± 36.0	129.0
80	40.2 ± 0.2	40.2	94.6 ± 3.8	89.6	770.3 ± 29.6	710.9	140.6 ± 5.6	132.8
100	40.1 ± 0.2	40.2	92.4 ± 3.4	87.9	783.2 ± 34.9	731.4	141.1 ± 4.0	136.5
120	40.1 ± 0.2	40.2	88.9 ± 1.1	86.4	804.8 ± 14.2	750.6	161.1 ± 26.2	140.1
140	40.3 ± 0.1	40.2	88.0 ± 2.1	85.1	828.8 ± 16.5	768.5	148.0 ± 3.2	143.6
160	40.2 ± 0.3	40.2	86.3 ± 2.2	84.0	841.2 ± 16.0	785.4	156.2 ± 14.5	146.9
180	40.4 ± 0.1	40.2	88.3 ± 1.0	83.0	851.2 ± 14.0	801.5	154.1 ± 5.7	150.2
200	40.1 ± 0.1	40.3	85.7 ± 1.8	82.1	868.3 ± 17.9	816.8	160.0 ± 6.8	153.4

Table S445: Densities computed from MC and MD simulations (ρ^{MC} and ρ^{MD}), densities obtained from REFPROP[10] (ρ^{REFP}), isothermal compressibilities computed from MC simulations (β_T^{MC}), isothermal compressibilities obtained from REFPROP[10] (β_T^{REFP}), thermal expansion coefficients computed from MC simulations (α_P^{MC}), thermal expansion coefficients obtained from REFPROP[10] (α_P^{REFP}), Joule Thomson coefficients computed from MC simulations ($\mu_{\mathrm{JT}}^{\mathrm{MC}}$), and Joule Thomson coefficients obtained from REFPROP[10] ($\mu_{\mathrm{JT}}^{\mathrm{REFP}}$) of CO_2 rich quaternary mixture with 2% impurity of N_2, 1% impurity of Ar and 1% impurity of H_2 at 273 K and pressures ranging from 20 bar to 200 bar.

P/ [bar]	ρ^{MC}/ [kg/m^3]	ρ^{MD}/ [kg/m^3]	ρ^{REFP}/ [kg/m^3]	β_T^{MC}/ [10^{-5}/bar]	β_T^{REFP}/ [10^{-5}/bar]	α_P^{MC}/ [10^{-4}/K]	α_P^{REFP}/ [10^{-4}/K]	$\mu_{\mathrm{JT}}^{\mathrm{MC}}$/ [$10^{-3}$ K/bar]	$\mu_{\mathrm{JT}}^{\mathrm{REFP}}$/ [$10^{-3}$ K/bar]
20	43.5	44.2 ± 0.1	44.3	5802.4 ± 19.4	5929.5	56.8 ± 0.2	60.0	1202.2 ± 11.8	1325.1
40	109.0 ± 0.2	870.1 ± 2.7	237.0	4336.3 ± 98.7	-5192.1	132.8 ± 3.2	-645.7	1286.7 ± 46.6	–
60	893.8 ± 3.1	891.1 ± 1.8	883.4	101.6 ± 7.1	125.8	74.5 ± 1.9	82.7	44.2 ± 2.3	–
80	909.5 ± 3.0	906.0 ± 0.6	907.0	79.9 ± 5.7	93.9	63.5 ± 3.6	67.5	33.0 ± 4.6	38.9
100	923.1 ± 0.8	921.6 ± 0.9	922.7	66.4 ± 4.1	78.6	55.6 ± 3.9	59.7	24.4 ± 5.2	30.1
120	935.6 ± 0.9	935.0 ± 0.7	936.3	63.2 ± 1.2	67.9	55.4 ± 1.4	54.2	23.4 ± 1.8	23.4
140	946.4 ± 0.8	944.3 ± 1.1	948.3	53.1 ± 0.4	60.0	47.9 ± 0.4	49.9	15.1 ± 0.5	18.1
160	958.0 ± 1.3	954.3 ± 1.0	959.1	49.8 ± 0.9	53.9	47.0 ± 1.2	46.5	13.5 ± 1.6	13.6
180	965.4 ± 0.2	964.1 ± 1.1	969.0	44.8 ± 1.9	49.0	43.3 ± 1.9	43.7	9.0 ± 2.5	9.9
200	973.5 ± 0.8	972.6 ± 0.7	978.2	41.5 ± 2.3	45.0	41.2 ± 2.2	41.3	6.2 ± 3.0	6.7

Table S446: Heat capacities at constant volume computed from MC simulations (c_V^{MC}), heat capacities at constant volume obtained from REFPROP[10] (c_V^{REFP}), heat capacities at constant pressure computed from MC simulations (c_P^{MC}), heat capacities at constant pressure obtained from REFPROP[10] (c_P^{REFP}), speed of sound computed from MC simulations (c^{MC}), speed of sound obtained from REFPROP[10] (c^{REFP}), viscosities computed from MD simulations (η^{MD}), and viscosities obtained from REFPROP[10] (η^{REFP}) of CO_2 rich quaternary mixture with 2% impurity of N_2, 1% impurity of Ar and 1% impurity of H_2 at 273 K and pressures ranging from 20 bar to 200 bar.

P/ [bar]	c_V^{MC}/ [J/mol K]	c_V^{REFP}/ [J/mol K]	c_P^{MC}/ [J/mol K]	c_P^{REFP}/ [J/mol K]	c^{MC}/ [m/s]	c^{REFP}/ [m/s]	η^{MD}/ [μPa s]	η^{REFP}/ [μPa s]
20	30.5	30.8	45.6 ± 0.1	47.0	243.4 ± 0.5	240.9	10.6 ± 3.8	14.1
40	36.8 ± 0.2	–	81.0 ± 1.2	–	215.9 ± 3.0	–	94.2 ± 4.9	18.3
60	39.8 ± 0.1	–	113.2 ± 1.4	–	559.9 ± 19.8	–	96.4 ± 4.0	89.8
80	39.5 ± 0.2	40.1	105.4 ± 3.3	103.2	605.7 ± 23.8	549.4	100.5 ± 3.8	95.9
100	39.6 ± 0.3	39.9	99.2 ± 4.8	98.0	639.5 ± 25.3	581.9	104.2 ± 4.1	100.3
120	39.4 ± 0.2	39.8	101.0 ± 2.0	94.3	658.5 ± 9.2	610.3	107.7 ± 14.7	104.3
140	39.6 ± 0.2	39.7	93.4 ± 0.6	91.4	685.0 ± 3.7	635.7	113.8 ± 9.6	108.0
160	39.4 ± 0.2	39.7	94.3 ± 2.0	89.1	708.3 ± 10.2	658.8	131.9 ± 39.4	111.5
180	39.6 ± 0.2	39.7	90.8 ± 2.3	87.1	728.1 ± 17.9	680.1	119.1 ± 5.2	114.9
200	39.6 ± 0.1	39.7	89.4 ± 2.7	85.5	747.9 ± 23.6	699.9	125.4 ± 8.5	118.1

Table S447: Densities computed from MC and MD simulations (ρ^{MC} and ρ^{MD}), densities obtained from REFPROP[10] (ρ^{REFP}), isothermal compressibilities computed from MC simulations (β_T^{MC}), isothermal compressibilities obtained from REFPROP[10] (β_T^{REFP}), thermal expansion coefficients computed from MC simulations (α_P^{MC}), thermal expansion coefficients obtained from REFPROP[10] (α_P^{REFP}), Joule Thomson coefficients computed from MC simulations ($\mu_{\mathrm{JT}}^{\mathrm{MC}}$), and Joule Thomson coefficients obtained from REFPROP[10] ($\mu_{\mathrm{JT}}^{\mathrm{REFP}}$) of CO_2 rich quaternary mixture with 2% impurity of N_2, 1% impurity of Ar and 1% impurity of H_2 at 293 K and pressures ranging from 20 bar to 200 bar.

P/ [bar]	ρ^{MC}/ [kg/m^3]	ρ^{MD}/ [kg/m^3]	ρ^{REFP}/ [kg/m^3]	β_T^{MC}/ [10^{-5}/bar]	β_T^{REFP}/ [10^{-5}/bar]	α_P^{MC}/ [10^{-4}/K]	α_P^{REFP}/ [10^{-4}/K]	$\mu_{\mathrm{JT}}^{\mathrm{MC}}$/ [$10^{-3}$ K/bar]	$\mu_{\mathrm{JT}}^{\mathrm{REFP}}$/ [$10^{-3}$ K/bar]
20	39.2	39.7 ± 0.1	39.8	5593.8 ± 33.7	5652.7	47.8 ± 0.3	49.4	1009.5 ± 23.8	1098.3
40	90.0 ± 0.1	91.8 ± 0.1	93.2	3394.2 ± 52.4	3488.4	76.9 ± 1.1	83.2	1039.4 ± 26.4	1090.8
60	172.5 ± 0.7	180.4 ± 1.4	188.5	3514.2 ± 117.8	4225.6	185.4 ± 6.6	252.5	1000.3 ± 51.7	1019.0
80	748.7 ± 3.8	740.0 ± 4.8	734.1	372.0 ± 82.7	491.4	165.4 ± 24.1	192.4	132.0 ± 26.0	157.9
100	789.2 ± 2.5	786.4 ± 2.9	785.0	201.0 ± 7.5	242.0	105.9 ± 2.5	115.0	88.4 ± 3.6	101.0
120	816.6 ± 1.8	815.3 ± 2.1	816.8	150.0 ± 9.8	165.2	87.5 ± 4.8	88.3	69.4 ± 6.7	74.0
140	839.0 ± 3.2	836.2 ± 2.3	840.7	111.1 ± 6.9	126.9	70.4 ± 3.0	73.8	51.1 ± 4.3	57.1
160	857.4 ± 1.2	855.1 ± 1.2	860.1	97.2 ± 2.4	103.5	65.4 ± 2.2	64.4	44.2 ± 3.3	45.2
180	872.0 ± 0.4	870.6 ± 0.9	876.7	82.7 ± 4.3	87.8	58.8 ± 2.7	57.8	35.7 ± 4.0	36.2
200	887.1 ± 1.0	884.3 ± 0.9	891.1	69.9 ± 4.4	76.3	52.2 ± 2.9	52.8	27.2 ± 4.4	29.0

Table S448: Heat capacities at constant volume computed from MC simulations (c_V^{MC}), heat capacities at constant volume obtained from REFPROP[10] (c_V^{REFP}), heat capacities at constant pressure computed from MC simulations (c_P^{MC}), heat capacities at constant pressure obtained from REFPROP[10] (c_P^{REFP}), speed of sound computed from MC simulations (c^{MC}), speed of sound obtained from REFPROP[10] (c^{REFP}), viscosities computed from MD simulations (η^{MD}), and viscosities obtained from REFPROP[10] (η^{REFP}) of CO_2 rich quaternary mixture with 2% impurity of N_2, 1% impurity of Ar and 1% impurity of H_2 at 293 K and pressures ranging from 20 bar to 200 bar.

P/ [bar]	c_V^{MC}/ [J/mol K]	c_V^{REFP}/ [J/mol K]	c_P^{MC}/ [J/mol K]	c_P^{REFP}/ [J/mol K]	c^{MC}/ [m/s]	c^{REFP}/ [m/s]	η^{MD}/ [μPa s]	η^{REFP}/ [μPa s]
20	30.5	30.6	43.7 ± 0.1	44.4	255.6 ± 0.8	254.0	4.8 ± 3.1	15.1
40	33.4	34.1	57.9 ± 0.3	61.1	238.2 ± 1.9	234.6	17.9 ± 4.2	15.8
60	39.1 ± 0.3	42.6	111.0 ± 3.0	144.0	216.5 ± 4.7	205.9	17.2 ± 0.6	17.9
80	40.1 ± 0.3	42.9	168.2 ± 12.1	172.9	388.0 ± 45.3	334.1	62.6 ± 3.0	61.0
100	39.8 ± 0.4	41.0	130.3 ± 2.6	129.3	454.2 ± 9.9	407.2	73.5 ± 3.2	69.3
120	39.3 ± 0.2	40.3	119.3 ± 4.1	113.4	498.1 ± 18.5	456.7	77.5 ± 3.9	75.2
140	39.2 ± 0.2	39.9	107.0 ± 2.1	104.6	541.2 ± 17.7	495.7	99.5 ± 27.2	80.0
160	39.2 ± 0.2	39.7	104.7 ± 2.5	98.7	565.8 ± 10.0	528.7	86.7 ± 6.3	84.2
180	39.2 ± 0.1	39.5	100.2 ± 2.6	94.5	595.7 ± 17.1	557.5	87.1 ± 5.7	88.0
200	39.3 ± 0.1	39.4	94.9 ± 2.8	91.3	624.7 ± 21.8	583.5	92.7 ± 4.1	91.5

Table S449: Densities computed from MC and MD simulations (ρ^{MC} and ρ^{MD}), densities obtained from REFPROP[10] (ρ^{REFP}), isothermal compressibilities computed from MC simulations (β_T^{MC}), isothermal compressibilities obtained from REFPROP[10] (β_T^{REFP}), thermal expansion coefficients computed from MC simulations (α_P^{MC}), thermal expansion coefficients obtained from REFPROP[10] (α_P^{REFP}), Joule Thomson coefficients computed from MC simulations ($\mu_{\mathrm{JT}}^{\mathrm{MC}}$), and Joule Thomson coefficients obtained from REFPROP[10] ($\mu_{\mathrm{JT}}^{\mathrm{REFP}}$) of CO_2 rich quaternary mixture with 2% impurity of N_2, 1% impurity of Ar and 1% impurity of H_2 at 313 K and pressures ranging from 20 bar to 200 bar.

P/ [bar]	ρ^{MC}/ [kg/m^3]	ρ^{MD}/ [kg/m^3]	ρ^{REFP}/ [kg/m^3]	β_T^{MC}/ [10^{-5}/bar]	β_T^{REFP}/ [10^{-5}/bar]	α_P^{MC}/ [10^{-4}/K]	α_P^{REFP}/ [10^{-4}/K]	$\mu_{\mathrm{JT}}^{\mathrm{MC}}$/ [$10^{-3}$ K/bar]	$\mu_{\mathrm{JT}}^{\mathrm{REFP}}$/ [$10^{-3}$ K/bar]
20	35.8	36.3	36.3	5443.9 ± 18.3	5484.7	41.7 ± 0.1	42.7	856.8 ± 11.7	926.4
40	79.0 ± 0.1	80.1 ± 0.1	80.9	3054.2 ± 19.3	3123.5	57.5 ± 0.3	60.8	856.0 ± 11.5	914.3
60	134.8 ± 0.2	138.5 ± 0.4	140.7	2422.2 ± 17.5	2552.7	88.3 ± 0.7	98.0	838.8 ± 11.6	880.7
80	219.0 ± 0.7	227.7 ± 2.2	236.5	2517.0 ± 113.5	2798.7	172.7 ± 8.6	209.5	773.0 ± 54.7	779.3
100	407.9 ± 11.5	435.3 ± 6.8	450.4	3438.7 ± 353.0	2957.8	541.1 ± 66.9	501.5	510.6 ± 89.3	–
120	611.8 ± 6.0	610.5 ± 6.2	618.7	826.6 ± 78.0	791.4	244.4 ± 17.3	223.8	231.0 ± 21.7	231.7
140	686.0 ± 3.5	679.1 ± 3.8	689.2	385.6 ± 27.0	379.2	143.7 ± 9.3	135.2	150.0 ± 14.2	149.5
160	726.1 ± 1.3	724.5 ± 1.8	732.2	245.9 ± 11.9	244.9	106.6 ± 4.6	100.9	110.1 ± 7.4	108.9
180	758.9 ± 1.8	755.3 ± 2.3	763.6	173.6 ± 12.3	180.4	84.4 ± 5.6	82.6	83.0 ± 9.5	84.1
200	781.6 ± 1.8	780.3 ± 1.5	788.4	138.7 ± 8.9	142.8	73.5 ± 4.2	71.0	67.3 ± 7.3	67.0

Table S450: Heat capacities at constant volume computed from MC simulations (c_V^{MC}), heat capacities at constant volume obtained from REFPROP[10] (c_V^{REFP}), heat capacities at constant pressure computed from MC simulations (c_P^{MC}), heat capacities at constant pressure obtained from REFPROP[10] (c_P^{REFP}), speed of sound computed from MC simulations (c^{MC}), speed of sound obtained from REFPROP[10] (c^{REFP}), viscosities computed from MD simulations (η^{MD}), and viscosities obtained from REFPROP[10] (η^{REFP}) of CO_2 rich quaternary mixture with 2% impurity of N_2, 1% impurity of Ar and 1% impurity of H_2 at 313 K and pressures ranging from 20 bar to 200 bar.

P/ [bar]	c_V^{MC}/ [J/mol K]	c_V^{REFP}/ [J/mol K]	c_P^{MC}/ [J/mol K]	c_P^{REFP}/ [J/mol K]	c^{MC}/ [m/s]	c^{REFP}/ [m/s]	η^{MD}/ [μPa s]	η^{REFP}/ [μPa s]
20	30.8	30.8	42.9 ± 0.1	43.2	267.1 ± 0.5	265.5	5.3 ± 3.7	16.1
40	32.6	33.0	51.2 ± 0.1	52.8	255.0 ± 0.9	251.6	15.5 ± 1.0	16.6
60	35.0 ± 0.1	35.9	67.4 ± 0.3	72.1	242.8 ± 1.2	236.3	17.9 ± 1.2	17.8
80	38.7 ± 0.3	40.6	112.5 ± 4.1	130.4	229.6 ± 6.7	220.2	23.3 ± 4.7	20.5
100	42.0 ± 0.2	–	331.1 ± 37.1	–	237.2 ± 18.3	–	30.9 ± 5.4	31.7
120	40.8 ± 0.4	42.7	203.4 ± 9.3	181.0	314.0 ± 16.6	294.4	48.1 ± 4.8	46.8
140	39.9 ± 0.1	41.0	147.0 ± 6.7	135.7	373.2 ± 15.6	355.8	56.1 ± 5.6	55.3
160	39.3	40.2	126.4 ± 3.5	117.1	424.6 ± 11.8	403.0	59.4 ± 2.5	61.4
180	39.0 ± 0.1	39.8	112.7 ± 4.6	106.8	468.4 ± 19.2	441.4	64.8 ± 2.9	66.2
200	38.9 ± 0.2	39.5	106.8 ± 3.7	100.1	503.1 ± 18.3	474.4	73.0 ± 3.1	70.4

S17.12 Data of thermodynamics and transport properties of CO_2 rich quaternary mixture with 2 mole% impurity of N_2, 1 mole% impurity of N_2, and 1 mole% impurity of H_2

Table S451: Densities computed from MC and MD simulations (ρ^{MC} and ρ^{MD}), densities obtained from REFPROP[10] (ρ^{REFP}), isothermal compressibilities computed from MC simulations (β_T^{MC}), isothermal compressibilities obtained from REFPROP[10] (β_T^{REFP}), thermal expansion coefficients computed from MC simulations (α_P^{MC}), thermal expansion coefficients obtained from REFPROP[10] (α_P^{REFP}), Joule Thomson coefficients computed from MC simulations (μ_{JT}^{MC}), and Joule Thomson coefficients obtained from REFPROP[10] (μ_{JT}^{REFP}) of CO_2 rich quaternary mixture with 2% impurity of Ar, 1% impurity of N_2 and 1% impurity of H_2 at 253 K and pressures ranging from 20 bar to 200 bar.

P/ [bar]	ρ^{MC}/ [kg/m^3]	ρ^{MD}/ [kg/m^3]	ρ^{REFP}/ [kg/m^3]	β_T^{MC}/ [10^{-5}/bar]	β_T^{REFP}/ [10^{-5}/bar]	α_P^{MC}/ [10^{-4}/K]	α_P^{REFP}/ [10^{-4}/K]	μ_{JT}^{MC}/ [10^{-3} K/bar]	μ_{JT}^{REFP}/ [10^{-3} K/bar]
20	49.6	50.5 ± 0.2	51.0	6320.6 ± 54.6	6481.4	74.5 ± 0.7	80.6	1526.2 ± 29.8	1637.7
40	999.8 ± 1.3	998.3 ± 0.7	783.4	45.3 ± 2.5	−254.4	47.8 ± 2.1	−52.2	9.5 ± 2.4	–
60	1008.6 ± 1.4	1006.4 ± 1.5	1006.8	40.7 ± 0.9	47.2	44.9 ± 0.8	47.2	6.2 ± 1.0	9.2
80	1014.9 ± 1.5	1014.8 ± 1.1	1015.9	40.2 ± 2.1	43.0	44.8 ± 2.3	44.4	6.0 ± 2.7	5.8
100	1022.6 ± 1.3	1022.4 ± 0.5	1024.3	36.8 ± 1.3	39.5	42.4 ± 1.3	42.0	3.4 ± 1.6	3.0
120	1032.2 ± 0.8	1029.4 ± 0.6	1032.1	33.5 ± 1.3	36.7	40.0 ± 1.6	39.9	0.5 ± 1.9	0.5
140	1038.6 ± 0.7	1036.3 ± 0.5	1039.5	31.0 ± 1.5	34.2	37.5 ± 1.7	38.2	-2.4 ± 2.1	−1.7
160	1043.5 ± 1.1	1042.8 ± 0.5	1046.4	30.4 ± 0.8	32.1	37.8 ± 1.5	36.7	-2.1 ± 1.8	−3.6
180	1049.9 ± 1.0	1049.1 ± 1.0	1052.9	28.7 ± 1.3	30.3	36.2 ± 1.5	35.3	-3.9 ± 1.8	−5.3
200	1056.0 ± 0.9	1054.5 ± 0.6	1059.2	26.7 ± 0.6	28.7	34.3 ± 1.0	34.1	-6.4 ± 1.2	−6.9

Table S452: Heat capacities at constant volume computed from MC simulations (c_V^{MC}), heat capacities at constant volume obtained from REFPROP[10] (c_V^{REFP}), heat capacities at constant pressure computed from MC simulations (c_P^{MC}), heat capacities at constant pressure obtained from REFPROP[10] (c_P^{REFP}), speed of sound computed from MC simulations (c^{MC}), speed of sound obtained from REFPROP[10] (c^{REFP}), viscosities computed from MD simulations (η^{MD}), and viscosities obtained from REFPROP[10] (η^{REFP}) of CO_2 rich quaternary mixture with 2% impurity of Ar, 1% impurity of N_2 and 1% impurity of H_2 at 253 K and pressures ranging from 20 bar to 200 bar.

P/ [bar]	c_V^{MC}/ [J/mol K]	c_V^{REFP}/ [J/mol K]	c_P^{MC}/ [J/mol K]	c_P^{REFP}/ [J/mol K]	c^{MC}/ [m/s]	c^{REFP}/ [m/s]	η^{MD}/ [μPa s]	η^{REFP}/ [μPa s]
20	31.3	32.4	50.7 ± 0.2	53.9	227.4 ± 1.1	224.6	12.1 ± 3.0	13.1
40	39.9	–	95.4 ± 2.2	–	726.6 ± 21.7	–	134.0 ± 5.2	71.9
60	40.1 ± 0.1	40.1	93.9 ± 0.9	91.5	755.6 ± 9.3	693.7	132.8 ± 3.4	129.8
80	39.9 ± 0.2	40.0	94.4 ± 3.1	89.5	761.4 ± 23.7	715.6	149.8 ± 13.4	133.7
100	40.0 ± 0.3	40.0	92.7 ± 1.6	87.8	785.0 ± 15.6	735.8	162.7 ± 37.8	137.3
120	40.1 ± 0.3	40.0	90.6 ± 2.5	86.3	807.8 ± 19.1	754.7	151.1 ± 7.3	140.9
140	40.2 ± 0.2	40.1	88.0 ± 2.2	85.0	824.7 ± 22.7	772.4	158.6 ± 12.5	144.3
160	40.0 ± 0.2	40.1	89.4 ± 2.6	83.9	839.3 ± 16.9	789.1	152.6 ± 5.2	147.7
180	40.3 ± 0.3	40.1	88.0 ± 2.1	83.0	850.8 ± 22.1	805.0	164.1 ± 7.2	150.9
200	40.1 ± 0.2	40.2	85.7 ± 1.9	82.1	870.1 ± 14.1	820.1	164.1 ± 5.1	154.1

Table S453: Densities computed from MC and MD simulations (ρ^{MC} and ρ^{MD}), densities obtained from REFPROP[10] (ρ^{REFP}), isothermal compressibilities computed from MC simulations (β_T^{MC}), isothermal compressibilities obtained from REFPROP[10] (β_T^{REFP}), thermal expansion coefficients computed from MC simulations (α_P^{MC}), thermal expansion coefficients obtained from REFPROP[10] (α_P^{REFP}), Joule Thomson coefficients computed from MC simulations (μ_{JT}^{MC}), and Joule Thomson coefficients obtained from REFPROP[10] (μ_{JT}^{REFP}) of CO_2 rich quaternary mixture with 2% impurity of Ar, 1% impurity of N_2 and 1% impurity of H_2 at 273 K and pressures ranging from 20 bar to 200 bar.

P/ [bar]	ρ^{MC}/ [kg/m^3]	ρ^{MD}/ [kg/m^3]	ρ^{REFP}/ [kg/m^3]	β_T^{MC}/ [10^{-5}/bar]	β_T^{REFP}/ [10^{-5}/bar]	α_P^{MC}/ [10^{-4}/K]	α_P^{REFP}/ [10^{-4}/K]	μ_{JT}^{MC}/ [10^{-3} K/bar]	μ_{JT}^{REFP}/ [10^{-3} K/bar]
20	43.6	44.3 ± 0.1	44.4	5828.7 ± 25.0	5925.9	57.0 ± 0.3	59.9	1213.9 ± 18.1	1322.5
40	109.2 ± 0.2	877.0 ± 2.3	241.4	4326.5 ± 103.6	−4660.8	132.6 ± 3.1	−603.3	1290.9 ± 45.0	–
60	898.5 ± 4.2	898.8 ± 2.8	894.2	104.8 ± 8.9	114.4	78.6 ± 4.7	77.7	46.8 ± 5.5	49.4
80	916.4 ± 2.2	912.4 ± 1.3	912.6	80.8 ± 5.2	91.5	65.3 ± 3.6	66.6	34.3 ± 4.4	37.8
100	929.7 ± 2.2	927.9 ± 1.6	928.0	67.3 ± 2.2	76.9	56.8 ± 1.4	59.1	25.5 ± 1.8	29.4
120	940.2 ± 1.7	939.7 ± 0.9	941.4	59.9 ± 3.5	66.7	52.6 ± 2.6	53.7	20.5 ± 3.4	22.8
140	953.0 ± 1.4	950.2 ± 0.8	953.3	52.9 ± 2.1	59.1	48.8 ± 1.9	49.5	15.9 ± 2.5	17.6
160	961.1 ± 0.8	960.5 ± 1.0	964.0	48.9 ± 2.4	53.1	46.2 ± 2.2	46.2	12.7 ± 2.9	13.2
180	970.5 ± 1.1	969.3 ± 0.7	973.8	46.1 ± 1.9	48.4	44.9 ± 1.9	43.5	10.9 ± 2.5	9.6
200	980.2 ± 1.0	977.5 ± 1.5	982.9	41.4 ± 0.9	44.5	41.3 ± 0.7	41.2	6.3 ± 0.9	6.4

Table S454: Heat capacities at constant volume computed from MC simulations (c_V^{MC}), heat capacities at constant volume obtained from REFPROP[10] (c_V^{REFP}), heat capacities at constant pressure computed from MC simulations (c_P^{MC}), heat capacities at constant pressure obtained from REFPROP[10] (c_P^{REFP}), speed of sound computed from MC simulations (c^{MC}), speed of sound obtained from REFPROP[10] (c^{REFP}), viscosities computed from MD simulations (η^{MD}), and viscosities obtained from REFPROP[10] (η^{REFP}) of CO_2 rich quaternary mixture with 2% impurity of Ar, 1% impurity of N_2 and 1% impurity of H_2 at 273 K and pressures ranging from 20 bar to 200 bar.

P/ [bar]	c_V^{MC}/ [J/mol K]	c_V^{REFP}/ [J/mol K]	c_P^{MC}/ [J/mol K]	c_P^{REFP}/ [J/mol K]	c^{MC}/ [m/s]	c^{REFP}/ [m/s]	η^{MD}/ [μPa s]	η^{REFP}/ [μPa s]
20	30.5	30.7	45.5 ± 0.1	46.8	242.6 ± 0.6	240.7	11.8 ± 2.1	14.1
40	36.6 ± 0.2	–	80.6 ± 1.1	–	215.8 ± 3.1	–	91.7 ± 2.9	18.4
60	39.4 ± 0.2	40.2	118.1 ± 3.8	110.0	564.1 ± 25.8	517.0	98.3 ± 4.5	91.8
80	39.2 ± 0.2	39.9	107.9 ± 3.2	102.7	609.6 ± 21.5	554.9	99.3 ± 2.5	96.7
100	39.3 ± 0.2	39.8	100.6 ± 1.5	97.7	639.9 ± 11.7	586.8	110.0 ± 6.9	101.0
120	39.4 ± 0.1	39.6	97.9 ± 2.7	94.0	664.7 ± 21.3	614.7	121.9 ± 25.1	105.0
140	39.3 ± 0.1	39.6	95.3 ± 2.3	91.2	693.6 ± 16.2	639.7	116.1 ± 3.2	108.7
160	39.5 ± 0.2	39.5	93.4 ± 2.7	88.9	709.7 ± 20.1	662.5	119.3 ± 6.8	112.2
180	39.4 ± 0.1	39.5	93.0 ± 2.5	87.0	726.2 ± 18.1	683.6	119.7 ± 3.0	115.5
200	39.5 ± 0.1	39.5	89.2 ± 0.9	85.4	746.0 ± 8.6	703.2	127.8 ± 13.3	118.7

Table S455: Densities computed from MC and MD simulations (ρ^{MC} and ρ^{MD}), densities obtained from REFPROP[10] (ρ^{REFP}), isothermal compressibilities computed from MC simulations (β_T^{MC}), isothermal compressibilities obtained from REFPROP[10] (β_T^{REFP}), thermal expansion coefficients computed from MC simulations (α_P^{MC}), thermal expansion coefficients obtained from REFPROP[10] (α_P^{REFP}), Joule Thomson coefficients computed from MC simulations ($\mu_{\mathrm{JT}}^{\mathrm{MC}}$), and Joule Thomson coefficients obtained from REFPROP[10] ($\mu_{\mathrm{JT}}^{\mathrm{REFP}}$) of CO_2 rich quaternary mixture with 2% impurity of Ar, 1% impurity of N_2 and 1% impurity of H_2 at 293 K and pressures ranging from 20 bar to 200 bar.

P/ [bar]	ρ^{MC}/ [kg/m^3]	ρ^{MD}/ [kg/m^3]	ρ^{REFP}/ [kg/m^3]	β_T^{MC}/ [10^{-5}/bar]	β_T^{REFP}/ [10^{-5}/bar]	α_P^{MC}/ [10^{-4}/K]	α_P^{REFP}/ [10^{-4}/K]	$\mu_{\mathrm{JT}}^{\mathrm{MC}}$/ [$10^{-3}$ K/bar]	$\mu_{\mathrm{JT}}^{\mathrm{REFP}}$/ [$10^{-3}$ K/bar]
20	39.3	39.8	39.8	5560.1 ± 32.6	5650.9	47.5 ± 0.3	49.3	996.1 ± 21.7	1096.4
40	90.5 ± 0.1	91.7 ± 0.4	93.4	3371.5 ± 74.2	3485.2	76.6 ± 1.7	82.9	1032.7 ± 41.5	1090.6
60	173.1 ± 0.5	181.6 ± 1.5	188.6	3582.5 ± 131.0	4207.2	192.8 ± 5.5	250.1	999.8 ± 37.9	1020.7
80	750.3 ± 4.4	748.1 ± 2.9	742.9	435.0 ± 55.8	455.5	187.5 ± 18.1	183.1	143.5 ± 18.8	151.3
100	794.8 ± 3.0	792.3 ± 2.5	791.6	199.2 ± 9.1	232.7	106.8 ± 3.6	112.5	88.0 ± 4.7	98.2
120	822.5 ± 1.3	821.0 ± 1.4	822.5	147.1 ± 8.2	160.7	86.2 ± 4.3	87.0	68.1 ± 6.0	72.4
140	845.5 ± 1.3	843.4 ± 1.2	846.0	113.1 ± 4.9	124.1	72.8 ± 3.3	73.0	52.8 ± 4.8	56.0
160	862.4 ± 1.9	861.6 ± 1.2	865.2	98.1 ± 5.7	101.7	66.5 ± 3.0	63.9	45.1 ± 4.3	44.4
180	877.6 ± 2.3	876.6 ± 1.6	881.5	81.5 ± 5.3	86.4	57.9 ± 3.2	57.4	34.7 ± 4.8	35.6
200	891.8 ± 1.0	890.4 ± 1.3	895.9	70.2 ± 3.1	75.3	52.5 ± 2.2	52.5	27.5 ± 3.3	28.5

Table S456: Heat capacities at constant volume computed from MC simulations (c_V^{MC}), heat capacities at constant volume obtained from REFPROP[10] (c_V^{REFP}), heat capacities at constant pressure computed from MC simulations (c_P^{MC}), heat capacities at constant pressure obtained from REFPROP[10] (c_P^{REFP}), speed of sound computed from MC simulations (c^{MC}), speed of sound obtained from REFPROP[10] (c^{REFP}), viscosities computed from MD simulations (η^{MD}), and viscosities obtained from REFPROP[10] (η^{REFP}) of CO_2 rich quaternary mixture with 2% impurity of Ar, 1% impurity of N_2 and 1% impurity of H_2 at 293 K and pressures ranging from 20 bar to 200 bar.

P/ [bar]	c_V^{MC}/ [J/mol K]	c_V^{REFP}/ [J/mol K]	c_P^{MC}/ [J/mol K]	c_P^{REFP}/ [J/mol K]	c^{MC}/ [m/s]	c^{REFP}/ [m/s]	η^{MD}/ [μPa s]	η^{REFP}/ [μPa s]
20	30.4	30.5	43.6 ± 0.1	44.2	256.1 ± 0.8	253.8	6.8 ± 5.3	15.1
40	33.3	34.0	57.7 ± 0.5	60.8	238.5 ± 2.9	234.4	17.5 ± 3.1	15.8
60	38.8 ± 0.3	42.4	116.4 ± 1.7	142.5	220.0 ± 4.4	205.9	18.2 ± 1.9	17.9
80	40.4 ± 0.2	42.5	180.9 ± 10.2	168.3	370.3 ± 26.0	342.2	65.2 ± 4.0	62.0
100	39.6 ± 0.3	40.8	132.1 ± 2.6	128.1	458.8 ± 11.6	413.0	74.2 ± 5.1	70.0
120	39.0 ± 0.1	40.1	118.2 ± 3.7	112.8	500.8 ± 16.0	461.4	79.1 ± 4.5	75.8
140	39.1 ± 0.2	39.7	110.0 ± 3.4	104.2	542.4 ± 14.6	499.9	79.6 ± 1.3	80.6
160	39.1 ± 0.2	39.5	105.8 ± 2.4	98.4	565.8 ± 17.8	532.4	92.7 ± 9.7	84.7
180	39.0 ± 0.1	39.4	99.1 ± 3.1	94.3	596.4 ± 21.4	561.0	88.7 ± 11.4	88.5
200	38.9 ± 0.1	39.3	95.3 ± 2.3	91.1	625.4 ± 15.9	586.7	94.6 ± 4.4	92.0

Table S457: Densities computed from MC and MD simulations (ρ^{MC} and ρ^{MD}), densities obtained from REFPROP[10] (ρ^{REFP}), isothermal compressibilities computed from MC simulations (β_T^{MC}), isothermal compressibilities obtained from REFPROP[10] (β_T^{REFP}), thermal expansion coefficients computed from MC simulations (α_P^{MC}), thermal expansion coefficients obtained from REFPROP[10] (α_P^{REFP}), Joule Thomson coefficients computed from MC simulations ($\mu_{\mathrm{JT}}^{\mathrm{MC}}$), and Joule Thomson coefficients obtained from REFPROP[10] ($\mu_{\mathrm{JT}}^{\mathrm{REFP}}$) of CO_2 rich quaternary mixture with 2% impurity of Ar, 1% impurity of N_2 and 1% impurity of H_2 at 313 K and pressures ranging from 20 bar to 200 bar.

P/ [bar]	ρ^{MC}/ [kg/m^3]	ρ^{MD}/ [kg/m^3]	ρ^{REFP}/ [kg/m^3]	β_T^{MC}/ [10^{-5}/bar]	β_T^{REFP}/ [10^{-5}/bar]	α_P^{MC}/ [10^{-4}/K]	α_P^{REFP}/ [10^{-4}/K]	$\mu_{\mathrm{JT}}^{\mathrm{MC}}$/ [$10^{-3}$ K/bar]	$\mu_{\mathrm{JT}}^{\mathrm{REFP}}$/ [$10^{-3}$ K/bar]
20	35.9	36.4 ± 0.1	36.4	5433.1 ± 24.4	5483.7	41.6 ± 0.2	42.6	854.7 ± 16.7	925.0
40	79.3	80.8 ± 0.2	81.1	3040.5 ± 27.0	3122.5	57.4 ± 0.6	60.7	852.2 ± 19.6	914.2
60	135.3 ± 0.2	138.2 ± 0.9	141.0	2432.7 ± 42.3	2551.2	88.5 ± 1.6	97.7	843.0 ± 25.0	881.6
80	219.7 ± 0.8	229.8 ± 0.9	237.0	2563.7 ± 36.0	2802.0	176.2 ± 2.8	209.2	782.6 ± 18.9	781.6
100	415.1 ± 9.3	436.6 ± 8.6	455.8	3456.8 ± 208.5	3039.6	554.1 ± 29.3	521.5	502.7 ± 40.4	467.0
120	618.9 ± 3.8	613.0 ± 6.6	625.8	840.0 ± 97.0	770.6	252.4 ± 27.2	221.7	229.8 ± 34.3	227.7
140	691.1 ± 3.9	685.9 ± 2.5	695.2	358.6 ± 37.4	370.1	137.4 ± 12.0	133.8	143.7 ± 18.4	147.1
160	734.7 ± 2.9	730.4 ± 1.6	737.6	215.5 ± 12.1	240.1	96.8 ± 4.1	100.1	99.8 ± 6.7	107.4
180	762.5 ± 3.8	762.5 ± 1.7	768.5	178.7 ± 11.4	177.4	87.3 ± 4.8	82.0	85.3 ± 8.1	83.0
200	787.9 ± 1.4	786.0 ± 1.0	793.2	138.9 ± 7.7	140.7	74.3 ± 3.9	70.6	67.7 ± 6.7	66.2

Table S458: Heat capacities at constant volume computed from MC simulations (c_V^{MC}), heat capacities at constant volume obtained from REFPROP[10] (c_V^{REFP}), heat capacities at constant pressure computed from MC simulations (c_P^{MC}), heat capacities at constant pressure obtained from REFPROP[10] (c_P^{REFP}), speed of sound computed from MC simulations (c^{MC}), speed of sound obtained from REFPROP[10] (c^{REFP}), viscosities computed from MD simulations (η^{MD}), and viscosities obtained from REFPROP[10] (η^{REFP}) of CO_2 rich quaternary mixture with 2% impurity of Ar, 1% impurity of N_2 and 1% impurity of H_2 at 313 K and pressures ranging from 20 bar to 200 bar.

P/ [bar]	c_V^{MC}/ [J/mol K]	c_V^{REFP}/ [J/mol K]	c_P^{MC}/ [J/mol K]	c_P^{REFP}/ [J/mol K]	c^{MC}/ [m/s]	c^{REFP}/ [m/s]	η^{MD}/ [μPa s]	η^{REFP}/ [μPa s]
20	30.7	30.7	42.8 ± 0.1	43.1	267.0 ± 0.6	265.2	3.3 ± 3.0	16.1
40	32.5	32.9	51.0 ± 0.2	52.6	255.2 ± 1.3	251.3	16.6 ± 1.6	16.7
60	35.0 ± 0.1	35.8	67.3 ± 0.6	71.8	241.8 ± 2.4	236.2	17.8 ± 1.4	17.8
80	38.6 ± 0.3	40.4	113.8 ± 1.6	129.8	228.8 ± 2.5	220.0	19.9 ± 0.8	20.6
100	42.9 ± 0.9	45.7	339.8 ± 18.1	312.1	235.1 ± 10.1	222.0	30.9 ± 4.2	32.1
120	40.5 ± 0.6	42.4	210.4 ± 17.6	180.7	316.2 ± 22.7	297.4	48.4 ± 3.1	47.4
140	39.5 ± 0.2	40.7	144.1 ± 8.3	135.2	383.6 ± 22.9	359.1	55.0 ± 5.4	55.9
160	39.2 ± 0.1	40.0	120.1 ± 2.8	116.7	439.8 ± 13.4	406.1	65.3 ± 7.7	61.9
180	38.9 ± 0.1	39.6	115.5 ± 4.1	106.6	466.7 ± 17.1	444.4	66.7 ± 2.3	66.7
200	38.7 ± 0.2	39.3	107.6 ± 3.6	99.9	504.4 ± 16.4	477.2	73.7 ± 5.6	70.8

Literature Cited

(1) Potoff, J. J.; Siepmann, J. I. Vapor–liquid equilibria of mixtures containing alkanes, carbon dioxide, and nitrogen. *AIChE Journal* **2001**, *47*, 1676–1682.

(2) García-Pérez, E.; Parra, J. B.; Ania, C. O.; Dubbeldam, D.; Vlugt, T. J. H.; Castillo, J. M.; Merkling, P. J.; Calero, S. Unraveling the argon adsorption processes in MFI-type zeolite. *Journal of Physical Chemistry C* **2008**, *112*, 9976–9979.

(3) Köster, A.; Thol, M.; Vrabec, J. Molecular models for the hydrogen age: hydrogen, nitrogen, oxygen, argon, and water. *Journal of Chemical and Engineering Data* **2018**, *63*, 305–320.

(4) Martin, M. G.; Siepmann, J. I. Transferable potentials for phase equilibria. 1. United-atom description of n-alkanes. *Journal of Physical Chemistry B* **1998**, *102*, 2569–2577.

(5) Hens, R.; Rahbari, A.; Caro-Ortiz, S.; Dawass, N.; Erdös, M.; Poursaeidesfahani, A.; Salehi, H. S.; Celebi, A. T.; Ramdin, M.; Moultos, O. A.; Dubbeldam, D.; Vlugt, T. J. H. Brick-CFCMC: Open source software for Monte Carlo simulations of phase and reaction equilibria using the Continuous Fractional Component method. *Journal of Chemical Information and Modeling* **2020**, *60*, 2678–2682.

(6) Polat, H. M.; Salehi, H. S.; Hens, R.; Wasik, D. O.; Rahbari, A.; De Meyer, F.; Houriez, C.; Coquelet, C.; Calero, S.; Dubbeldam, D.; Vlugt, T. J. H. New features of the open source Monte Carlo software Brick-CFCMC: Thermodynamic integration and hybrid trial moves. *Journal of Chemical Information and Modeling* **2021**, *61*, 3752–3757.

(7) Rahbari, A.; Hens, R.; Ramdin, M.; Moultos, O.; Dubbeldam, D.; Vlugt, T. J. H. Recent advances in the Continuous Fractional Component Monte Carlo methodology. *Molecular Simulation* **2021**, *47*, 804–823.

(8) Shi, W.; Maginn, E. J. Continuous Fractional Component Monte Carlo: an adaptive biasing method for open system atomistic simulations. *Journal of Chemical Theory and Computation* **2007**, *3*, 1451–1463.

(9) Shi, W.; Maginn, E. J. Improvement in molecule exchange efficiency in Gibbs ensemble Monte Carlo: development and implementation of the Continuous Fractional Component move. *Journal of Computational Chemistry* **2008**, *29*, 2520–2530.

(10) Lemmon, E. W.; Bell, I. H.; Huber, M.; McLinden, M. NIST standard reference database 23: reference fluid thermodynamic and transport properties-REFPROP, Version 10.0, National Institute of Standards and Technology. *Standard Reference Data Program, Gaithersburg* **2018**,

(11) Rowlinson, J. S.; Swinton, F. *Liquids and liquid mixtures: Butterworths monographs in chemistry*, 3rd ed.; Butterworth-Heinemann, Elsevier, UK, 2013.

(12) Creton, B.; Nieto-Draghi, C.; De Bruin, T.; Lachet, V.; El Ahmar, E.; Valtz, A.; Coquelet, C.; Lasala, S.; Privat, R.; Jaubert, J.-N. Thermodynamic study of binary systems containing sulphur dioxide and nitric oxide: Measurements and modelling. *Fluid Phase Equilibria* **2018**, *461*, 84–100.

(13) Rowlinson, J. S.; Widom, B. *Molecular theory of capillarity*, 1st ed.; Courier Corporation, US, 2013.

(14) Span, R.; Wagner, W. A new equation of state for carbon dioxide covering the fluid region from the triple-point temperature to 1100 K at pressures up to 800 MPa. *Journal of Physical and Chemical Reference Data* **1996**, *25*, 1509–1596.

(15) Laesecke, A.; Muzny, C. D. Reference correlation for the viscosity of carbon dioxide. *Journal of Physical and Chemical Reference Data* **2017**, *46*.

(16) Span, R.; Lemmon, E. W.; Jacobsen, R. T.; Wagner, W.; Yokozeki, A. A reference equation of state for the thermodynamic properties of nitrogen for temperatures from 63.151 to 1000 K and pressures to 2200 MPa. *Journal of Physical and Chemical Reference Data* **2000**, *29*, 1361–1433.

(17) Lemmon, E. W.; Jacobsen, R. T. Viscosity and thermal conductivity equations for nitrogen, oxygen, argon, and air. *International Journal of Thermophysics* **2004**, *25*, 21–69.

(18) Tegeler, C.; Span, R.; Wagner, W. A new equation of state for argon covering the fluid region for temperatures from the melting line to 700 K at pressures up to 1000 MPa. *Journal of Physical and Chemical Reference Data* **1999**, *28*, 779–850.

(19) Leachman, J. W.; Jacobsen, R. T.; Penoncello, S.; Lemmon, E. W. Fundamental equations of state for parahydrogen, normal hydrogen, and orthohydrogen. *Journal of Physical and Chemical Reference Data* **2009**, *38*, 721–748.

(20) Muzny, C. D.; Huber, M. L.; Kazakov, A. F. Correlation for the viscosity of normal hydrogen obtained from symbolic regression. *Journal of Chemical and Engineering Data* **2013**, *58*, 969–979.

(21) Setzmann, U.; Wagner, W. A new equation of state and tables of thermodynamic properties for methane covering the range from the melting line to 625 K at pressures up to 1000 MPa. *Journal of Physical and Chemical Reference Data* **1991**, *20*, 1061–1155.

(22) Quiñones-Cisneros, S.; Huber, M.; Deiters, U. Reference correlation for the viscosity of methane. *unpublished work* **2011**,

(23) Deeg, K. S.; Gutiérrez-Sevillano, J. J.; Bueno-Pérez, R.; Parra, J. B.; Ania, C. O.; Doblaré, M.; Calero, S. Insights on the molecular mechanisms of hydrogen adsorption in zeolites. *The Journal of Physical Chemistry C* **2013**, *117*, 14374–14380.

(24) Poursaeidesfahani, A.; Torres-Knoop, A.; Dubbeldam, D.; Vlugt, T. J. H. Direct free energy calculation in the Continuous Fractional Component Gibbs ensemble. *Journal of Chemical Theory and Computation* **2016**, *12*, 1481–1490.

(25) Brown, T.; Niesen, V.; Sloan, E.; Kidnay, A. Vapor-liquid equilibria for the binary systems of nitrogen, carbon dioxide, and n-butane at temperatures from 220 to 344 K. *Fluid Phase Equilibria* **1989**, *53*, 7–14.

(26) Coquelet, C.; Valtz, A.; Dieu, F.; Richon, D.; Arpentinier, P.; Lockwood, F. Isothermal P, x, y data for the argon + carbon dioxide system at six temperatures from 233.32 to 299.21 K and pressures up to 14 MPa. *Fluid Phase Equilibria* **2008**, *273*, 38–43.

(27) Tsang, C.; Street, W. Phase equilibria in the H_2/CO_2 system at temperatures from 220 to 290 K and pressures to 172 MPa. *Chemical Engineering Science* **1981**, *36*, 993–1000.

(28) Davalos, J.; Anderson, W. R.; Phelps, R. E.; Kidnay, A. J. Liquid-vapor equilibria at 250.00K for systems containing methane, ethane, and carbon dioxide. *Journal of Chemical and Engineering Data* **1976**, *21*, 81–84.

(29) Wei, M. S.-W.; Brown, T. S.; Kidnay, A. J.; Sloan, E. D. Vapor+ liquid equilibria for the ternary system methane+ ethane+ carbon dioxide at 230 K and its constituent binaries at temperatures from 207 to 270 K. *Journal of Chemical and Engineering Data* **1995**, *40*, 726–731.

(30) Kunz, O.; Wagner, W. The GERG-2008 wide-range equation of state for natural gases and other mixtures: an expansion of GERG-2004. *Journal of Chemical and Engineering Data* **2012**, *57*, 3032–3091.

(31) Lagache, M.; Ungerer, P.; Boutin, A.; Fuchs, A. Prediction of thermodynamic derivative properties of fluids by Monte Carlo simulation. *Physical Chemistry Chemical Physics* **2001**, *3*, 4333–4339.

(32) Fazelabdolabadi, B.; Bahramian, A. Prediction of sound velocity in normal alkanes: A configurational-bias Monte Carlo simulation approach. *Fluid Phase Equilibria* **2009**, *284*, 129–136.

(33) Fazelabdolabadi, B.; Bahramian, A. Prediction of sound velocity and compressibility via molecular simulation at fixed entropy. *Fluid Phase Equilibria* **2010**, *293*, 262–264.

(34) Moran M. J, H. N., Shapiro *Fundamentals of engineering thermodynamics*, 5th ed.; John Wiley and Sons: West Sussex, U.K., 2006.

(35) Reid R.C, P. B., Prausnitz J.M *The properties of gases and liquids*, 5th ed.; McGraw-Hill: New York, 2001.

(36) Frisch, M. J.; Trucks, G. W.; Schlegel, H. B.; Scuseria, G. E.; Robb, M. A.; Cheeseman, J. R.; Scalmani, G.; Barone, V.; Mennucci, B.; Petersson, G. A.; Nakatsuji, H.; Caricato, M.; Li, X.; Hratchian, H. P.; Izmaylov, A. F.; Bloino, J.; Zheng, G.; Sonnenberg, J. L.; Hada, M.; Ehara, M.; Toyota, K.; Fukuda, R.; Hasegawa, J.; Ishida, M.; Nakajima, T.; Honda, Y.; Kitao, O.; Nakai, H.; Vreven, T.; Montgomery, J. J.; Peralta, J. E.; Ogliaro, F.; Bearpark, M.; Heyd, J. J.; Brothers, E.; Kudin, K. N.; Staroverov, V. N.; Kobayashi, R.; Normand, J.; Raghavachari, K.; Rendell, A.; Burant, J. C.; Iyengar, S. S.; Tomasi, J.; Cossi, M.; Rega, N.; Millam, J. M.; Klene, M.; Knox, J. E.; Cross, J. B.; Bakken, V.; Adamo, C.; Jaramillo, J.; Gomperts, R.; Stratmann, R. E.; Yazyev, O.; Austin, A. J.; Cammi, R.; Pomelli, C.; Ochterski, J. W.; Martin, R. L.; Morokuma, K.; Zakrzewski, V. G.; Voth, G. A.; Salvador, P.; Dannenberg, J. J.; Dapprich, S.; Daniels, A. D.; Farkas, O.; Foresman, J. B.; Ortiz, J. V.; Cioslowski, J.; Fox, D. J. *Gaussian 09*; Gaussian Inc Wallingford, 2009.

(37) Rahbari, A.; Garcia-Navarro, J. C.; Ramdin, M.; Van Den Broeke, L. J.; Moultos, O. A.; Dubbeldam, D.; Vlugt, T. J. H. Effect of water content on thermodynamic properties of compressed hydrogen. *Journal of Chemical and Engineering Data* **2021**, *66*, 2071–2087.

(38) Ishmael, M. P.; Stutzman, L. B.; Lukawski, M. Z.; Escobedo, F. A.; Tester, J. W. Heat capacities of supercritical fluid mixtures: Comparing experimental measurements with Monte Carlo molecular simulations for carbon dioxide-methanol mixtures. *The Journal of Supercritical Fluids* **2017**, *123*, 40–49.

(39) Aimoli, C. G.; Maginn, E. J.; Abreu, C. R. Force field comparison and thermodynamic property calculation of supercritical CO_2 and CH_4 using molecular dynamics simulations. *Fluid Phase Equilibria* **2014**, *368*, 80–90.

(40) Colina, C.; Olivera-Fuentes, C.; Siperstein, F.; Lisal, M.; Gubbins, K. Thermal properties of supercritical carbon dioxide by Monte Carlo simulations. *Molecular Simulation* **2003**, *29*, 405–412.
